# Effects of phosphate mining-induced P and F pollution on soil fungal communities: Spatial variations in structure and function

**DOI:** 10.1016/j.crmicr.2025.100506

**Published:** 2025-11-08

**Authors:** Lingzi Meng, Limin Zhou, Meiyue Xu, Kejin Ding, Bailun Liu, Ying Wang, Zhijun Wang, Feng Guo, Zhen Li

**Affiliations:** a College of Resources and Environmental Sciences, Nanjing Agricultural University, Nanjing, Jiangsu 210095, China; b Jiangsu Academy of Environmental Industry and Technology Corp, Nanjing, Jiangsu 210095, China; c Jiangsu Provincial Key Lab of Organic Solid Waste Utilization, Nanjing Agricultural University, Nanjing, Jiangsu 210095, China; d Jiangsu Provincial Key Laboratory of Coastal Saline Soil Resources Utilization and Ecological Conservation, Nanjing, Jiangsu 210095, China; e Gezhouba Group Eco-Environmental Protection Co., LTD., Qiaokou District, Wuhan, Hubei 430030, China

**Keywords:** Phosphate ore, Surrounding environment, Fluorine, Fungal community, Spatial scales

## Abstract

Phosphate ore is the primary phosphorus (P) source for ecosystem. Meanwhile, the intense exploitation of phosphate ore results in an escalating release of fluorine (F) into soil, posing potential risk to both the ecological system and public health. Thus, this study collected soil samples from distinct distances from phosphate ore. Mild F contamination (∼9 mg/kg, water-soluble) was detected in the areas near phosphate ore. Elevated concentrations of F and P were associated with an evident increase of fungal community richness and Ascomycota abundance. Additionally, F stress may weaken the co-occurrence networks and decrease the centralization of fungal communities. These responses of fungal communities enhanced their stress resistance capabilities. Meanwhile, P accumulation was observed for the soils near phosphate ore, which was consistent with the elevation of the abundance of phosphate-solubilizing fungi (PSF), enhancing the ability of this community to drive P cycle. This study offered a new perspective for sustainable management of phosphate mining, especially considering the pollution balance of P and F, and the microbial response to them.

## Highlights

• Geological apatite provides both P and F to the surrounding environment.
• The abundance of phosphate-solubilizing fungi was enhanced near phosphate ore.
• Phosphate ore did not significantly change the assembly process of fungal community.
• Slightly elevated F showed more significant influences on fungal community composition than P.
• The stress resistance of soil fungi community near phosphate ore was enhanced.

## Graphical abstract

**Figure:**
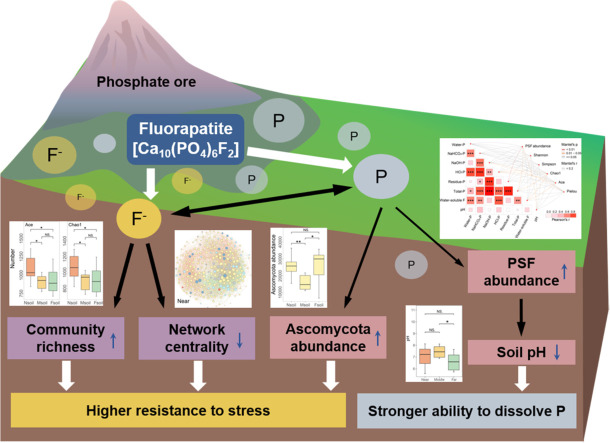

## 1 Introduction

With the rising demand for phosphates, the intensity of phosphate mining has been greatly enhanced (Li et al., 2022). The mining and smelting process of phosphates has led to serious environmental problems in surrounding areas (El Zrelli et al., 2018). For example, soil erosion and surface runoff will cause dissolution of phosphorus (P) from exposed phosphate ores. The excessive P could affect the absorption and utilization of other trace elements by plants (Magnan and Duvat, 2015; Stapanian et al., 2016). In the natural environment, geological fluorapatite [Ca_10_(PO_4_)_6_F_2_] is the most common P-bearing mineral (Pan and Fleet, 2002). Thus, industrial activities related to phosphate mining and processing became a major source of soil fluorine (F) (Butler et al., 2018). For example, the average background total F concentration (855 mg/kg) in topsoil in Guizhou Province, China is much higher than the national average (453 mg/kg) (Wang et al., 2021). In addition, compared with the average level of the surface soil, the concentration of water-soluble F also increased significantly (by up to 20 times, ∼6 μg/g) (Wang et al., 2012; Yang et al., 2020). The daily intake of F for humans typically ranges between 1 and 4 mg (EPA, 2000), and chronic exposure exceeding 6 mg/day increases the potential risk of skeletal poisoning (WHO, 2002).

Microorganisms are highly sensitive to environmental changes and play critical active roles in ecosystem function (Delgado-Baquerizo et al., 2020; Li et al., 2023), including nutrient cycling and the detoxification of pollutants (Maqsood et al., 2023; Chen et al., 2024). Under normal circumstances, the PO_4_^3−^ release rate of phosphate minerals is low (Tang et al., 2019). For instance, the dissolution rate constant of fluorapatite is as low as 1.0 × 10^−9^ (Manecki et al., 2000). Nevertheless, human activities (such as applying phosphate fertilizers) can cause a sharp increase in soil P concentration which led to the increase of microbial biomass and activity in soil (Turner and Wright, 2014; Meng et al., 2022). In addition, it further changed the microbial community composition (Ducousso-Détrez et al., 2022) and diversity (Yao et al., 2018). Previous study has proposed that F released from fluorapatite could degrade bioactivity of microorganisms (Shao et al., 2021). Meanwhile, the F inhibit the activity of enzymes and change the distribution of various microorganisms in soil (Wang et al., 2019). However, most current studies have predominantly focused on bacterial communities, often overlooking the pronounced sensitivity of fungal communities to soil environmental changes (Liang et al., 2020; Chen et al., 2023; Meng et al., 2024a; Pramanik et al., 2025). Moreover, few studies have explored the combined effects of concurrently elevated F and P on microbial communities under natural weather conditions. Therefore, it is necessary to elucidate the response of fungal communities to the changes accompanied of both F and P.

With the development of the phosphate mining industry and the growth in the number of tailings ponds, the environmental problem of phosphate ore continues to attract attention. This study focused on the soils surrounding a typical phosphate mine in Guizhou Province, China. The effects of phosphate mining on soil P and F concentrations were evaluated by physicochemical characterization. The composition and diversity of fungal communities were analyzed using high-throughput sequencing methods. The relationship between the fungal community and the comprehensive effect of P and F was further investigated.

## 2 Materials and methods

### 2.1 Field description and soil sample collection

**Fig. 1:**
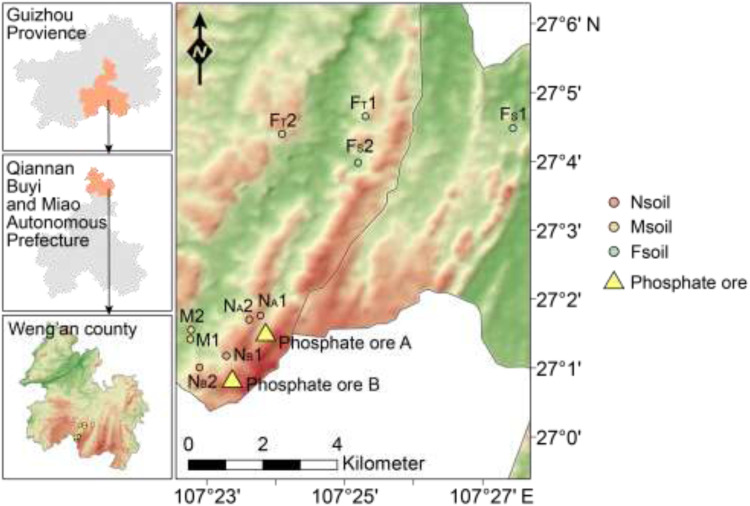
Location of the Weng’an mining area and the sampling points. N_A_1 and N_A_2 represent the sites near the phosphate ore A. N_B_1 and N_B_2 represent the sites near the phosphate ore B. M1 and M2 represent the sites at a medium distance from the phosphate ores. F_S_1 and F_S_2 represent the sites on the mountain slope. F_T_1 and F_T_2 represent sites on the mountaintop.

The study area is located in Weng'an County, south-central Guizhou Province, China (Fig. 1), which hosts one of the largest phosphate ore deposits in China. The predominant phosphate mineral is fluorapatite (Table S1). The region experiences a subtropical humid monsoon climate, with a mean annual temperature of 13.6 °C and mean annual precipitation of 1150 mm (CMA, 2024).

Soil sampling was conducted at varying distances from an active phosphate ore mining site (see Fig. 1, specific geographic coordinates are provided in Table S2). Sampling sites were categorized based on distance, i.e., Near (N) within 1 km, Middle (M) between 1 and 5 km, and Far (F) exceeding 5 km from the ore. A total of ten sampling sites were established as N_A_1, N_A_2, N_B_1, N_B_2 (representing proximity to ores A and B, respectively), M1, M2, F_S_1, F_S_2 (sites on the mountain slope), and F_T_1, F_T_2 (sites on the mountain top). At each site, three replicate soil cores were collected at 5 m intervals, yielding a total of 30 samples. The chemical properties of sample Msoil were shown in Table S4. Furthermore, due to the almost no significant difference in chemical properties of N_A_1, N_A_2, N_B_1 and N_B_2 (Table S3), these four sampling sites (12 samples) were combined into Nsoil when calculating the microbiome data. Msoil contained six samples in total from two sampling sites (M1 and M2). Similarly, the F_S_1, F_S_2, F_T_1 and F_T_2 (Table S5), 4 sampling sites (total 12 samples) were combined into Fsoil.

Soil sampling occurred in October 2022. At each sampling point, a soil core (diameter of 2 cm, depth of 0–20 cm) was randomly collected. Each composite sample was homogenized using sterile implements and subdivided. One portion was air-dried at ambient temperature for chemical analysis. The remaining portion was stored at −80 °C for subsequent DNA extraction and Illumina MiSeq sequencing.

### 2.2 Soil chemical properties analyses

Soil chemical composition was determined using an ARL Perform'X X-ray fluorescence spectrometer (XRF) (ThermoFisher Scientific Inc.) with an effective analysis diameter of 25 mm.

Soil pH was measured potentiometrically in a soil/water suspension (1:5 w/v) using an SG98 InLab pH meter (Mettler Toledo Int. Inc.) equipped with an Expert Pro-ISM-IP67 probe.

Water-soluble fluoride (F), chloride (Cl), nitrate (N), and sulfate (S) concentrations were extracted using ultrapure water (soil/water ratio 1:5 w/v) at 25 °C with ultrasonication for 30 min. Extracts were analyzed by ion chromatography (ICS-1100 system with conductivity detector) (Saha and Kundu, 2003).

Total carbon (TC) and total nitrogen (TN) content were quantified using a CN analyzer (Elementar Vario Micro cube, Germany). Soil organic carbon (SOC) was determined via the Walkley-Black wet oxidation method, based on unreduced potassium dichromate K_2_Cr_2_O_7_. Soil organic matter (SOM) concentration was derived by multiplying SOC by the Van Bemmelen factor (1.724) (Minasny et al., 2020).

Five inorganic P fractions were extracted sequentially (Tiessen and Moir, 1993) from air-dried soil ground to pass a 1-mm sieve. The water-P is the most labile and can be immediately utilized by microorganisms. The exchangeable and loosely adsorbed NaHCO_3_-P can be utilized by microorganisms and plants. The left three forms are the NaOH-P combined with aluminum and iron oxides (considered moderately recalcitrant), the calcium-bounded HCl-P (especially relevant in calcareous soils), and highly stable mineral-bounded residual P. Extractions were performed at 25 °C. The water-P fraction was extracted using 30 mL of ultrapure water with 16 h of shaking. The NaHCO_3_-P fraction was extracted with 0.5 M NaHCO_3_ at pH 8.5 under 16 h shaking. The NaOH-P fraction was obtained using 0.1 M NaOH with 16 h of shaking. The HCl-P fraction, which includes calcium-bound P and is especially relevant in calcareous soils, was extracted with 1 M HCl under 16 h shaking. Finally, the residual P was extracted by acid digestion (8 mL H_2_SO_4_ and 10 drops 70 % HClO_4_, heating ∼1 h). P concentrations were determined by using molybdenum-blue method (Murphy and Riley, 1986), at 880 nm by Shimadzu UV mini-1240.

### 2.3 DNA extraction and high-throughput sequencing

Microbial genomic DNA was extracted from 10 g (fresh weight equivalent) of soil using the E.Z.N.A.® Soil DNA Kit (Omega Bio-Tek, Norcross, GA, U.S.). The fungal ITS1 region was amplified using primers ITS1F (5′-CTTGGTCATTTAGAGGAAGTAA-3′) and ITS2 (5′-GCTGCGTTCTTCATCGATGC-3′), selected for their established classification accuracy and fungal sequence coverage (Mello et al., 2011). PCR reactions were performed in triplicate 20 μL mixture containing 4 μL of 5 × FastPfu Buffer, 2 μL of 2.5 mM dNTPs, 0.8 μL of each primer (5 μM), 0.4 μL of FastPfu Polymerase, and 1 μL of template DNA. The amplification program included 95 °C for 2 min, followed by 25 cycles at 95 °C for 30 s, 55 °C for 30 s, 72 °C for 30 s, and a final extension at 72 °C for 5 min. Amplicons were extracted from 2 % agarose gels and purified. Purified PCR products were quantified by Qubit®3.0 and every twenty-four amplicons whose barcodes were different were mixed equally, and served as DNA samples to be sequenced on the Illumina MiSeq System platform.

### 2.4 Bioinformatical analyses

Raw FASTQ files were demultiplexed and quality-filtered using QIIME (version 1.17). Reads (250 bp) were truncated at positions where the average quality score fell below 20 within a 10-bp sliding window. Reads shorter than 50 bp after truncation were discarded. Reads with exact barcode matches, allowing ≤2 nucleotide mismatches in primer sequences, or containing ambiguous characters, were removed. Following chimera removal and quality filtering, 1116,841 high-quality sequences remained for analysis (sequence length distribution in Table S6). Operational Taxonomic Units (OTUs) were clustered at 97 % sequence similarity using Usearch (version 10, http://drive5.com/uparse/) to generate an OTU table.

### 2.5 Statistical analysis

Alpha diversity of fungal communities was assessed using the following indices: richness (Chao1, ACE), diversity (Shannon, Simpson), and evenness (Pielou).

Beta diversity was evaluated using non-metric multidimensional scaling (NMDS) and hierarchical clustering based on Bray-Curtis dissimilarity matrices. Results were visualized in two-dimensional ordination plots.

The relationship between microbial community structure and soil environmental variables was examined using distance-based redundancy analysis (db-RDA). Subsequently, variance partitioning analysis (VPA) quantified the proportion of variation in fungal community structure explained by specific environmental factor groups.

Mantel tests (Pearson correlation) assessed correlations between fungal species composition profiles, environmental factors, abundance, and community diversity.

Community assembly processes were inferred using a null model approach. Specifically, the beta-nearest taxon index (βNTI) was calculated to assess taxonomic and phylogenetic diversity divergence patterns.

Co-occurrence networks (among OTUs, or between OTUs and environmental factors) were constructed based on significant (*p* < 0.05) Spearman correlation coefficients (|r| ≥ 0.6). Network topology metrics, including node number, edge number, average degree, average path length, centralization, and modularity, were calculated. Network visualization was performed using Gephi.

Structural equation modeling (SEM) was employed to test hypothesized pathways linking phosphate ore proximity to soil fungal community resistance and function, using the piecewiseSEM package (version 2.1.0) in R. The model incorporated three soil environmental factors (water-soluble F, total P, pH) and four microbial indicators (Ascomycota abundance, phosphate-solubilizing fungi (PSF) abundance, richness, network centrality).

Differences in microbial diversity between distance categories were assessed using paired *t*-tests. Non-significant differences (*p* ≥ 0.05) are denoted as "NS" in figures. All statistical analyses were performed using R software (version 4.0.4).

## 3 Results

### 3.1 Soil chemical properties

**Fig. 2:**
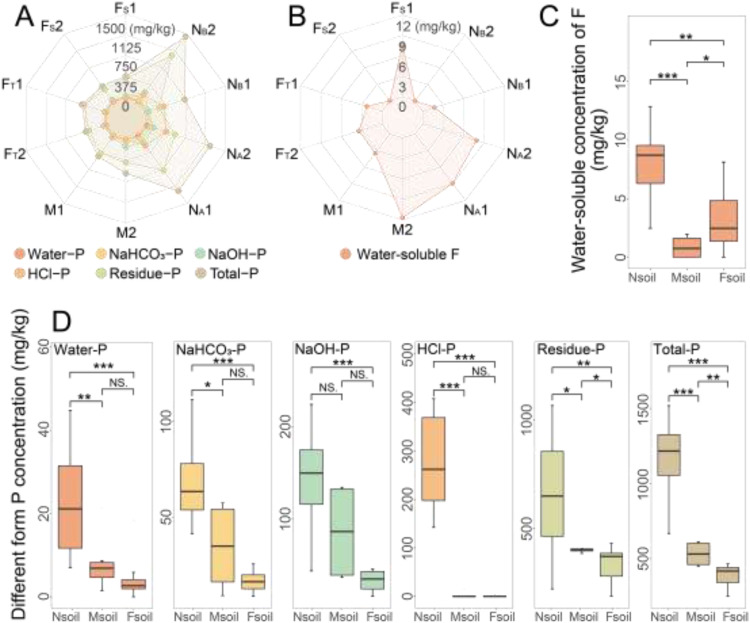
The concentrations of different P forms (A) and water-soluble F (B). The concentrations of water-soluble F (C) and different P forms (D) in the Nsoil, Msoil, and Fsoil. Note: “***” : *p* < 0.001, “**” : 0.001 < *p* < 0.01, “*” : 0.01 < *p* < 0.05, “NS.” : *p* > 0.05.

The concentrations of all forms of P (i.e., water-P, NaHCO_3_-P, HCl-P, and total P) in the soil samples also decreased with distance from the phosphate ore (Fig. 2A). The water-P concentrations in the Nsoil, Msoil, and Fsoil were 23.75, 8.21, 4.02 mg/kg, respectively. The NaHCO_3_-P concentrations in Nsoil, Msoil, and Fsoil were 68.49, 34.68, and 16.78 mg/kg, respectively. The HCl-P concentrations in the Nsoil, Msoil, and Fsoil showed the largest variation, i.e., 273.53, 2.24, and 0.05 mg/kg, respectively (Fig. 2D). For NaOH-P concentration, only the Nsoil (138.86 mg/kg) and Fsoil (31.75 mg/kg) showed significant differences (Fig. 2D). The residual P concentrations in Nsoil, Msoil, and Fsoil were 648.34, 401.65, and 336.19 mg/kg, respectively. In conclusion, the concentrations of all forms of P, especially the total P concentration, were the maximum in Nsoil.

The concentration of water-soluble F in the Nsoil was 8.00 mg/kg, which was significantly higher than those for Msoil (0.86 mg/kg) and Fsoil (3.33 mg/kg) (Fig. 2C). Despite the overall low water-soluble F concentrations in Msoil and Fsoil, considerable variation was observed within each group (see Fig. 2B).

For the other chemical properties of soil, the Msoil was generally significantly different from Nsoil and Fsoil. For instance, the pH value of Msoil is the highest (∼7.5) among the three soils, which is significantly higher than that of Fsoil (6.59). The concentrations of water-soluble Cl and S in Msoil were 8.79 and 94.21 mg/kg, respectively, which were significantly higher than those in Nsoil and Fsoil. In contrast, the concentration of water-soluble N in Msoil was 40.47 mg/kg, which was significantly lower than that in the Nsoil (128.31 mg/kg) and Fsoil (119.69 mg/kg). The TC and TS concentrations in Msoil were 31.73 and 4 g/kg, respectively (Table 1). Similarly, these concentrations were significantly lower than those in Nsoil and Fsoil. Additionally, the TN concentration in Msoil (2.85 g/kg) was significantly lower than that in Fsoil (3.61 g/kg) (Table 1). However, there was no statistically significant difference in SOC and SOM among the Nsoil (31.83 and 54.87 mg/kg), Msoil (22.58 and 38.93 mg/kg), and Fsoil (32.38 and 55.83 mg/kg) (Fig. S1). The contents of other elements of Nsoil, Msoil, and Fsoil were shown in Table S7.

**Table 1:** The chemical properties in the Nsoil, Msoil, and Fsoil (mean ± SE). Note: “***” : *p* < 0.001, “**” : 0.001 < *p* < 0.01, “*” : 0.01< *p* < 0.05, “NS.” : *p* > 0.05.

Chemical properties	Nsoil	Msoil	Fsoil	Nsoil*Msoil	Msoil*Fsoil	Nsoil*Fsoil
pH	7.01 ± 0.9	7.46 ± 0.55	6.59 ± 0.68	NS.	*	NS.
Water-soluble Cl (mg/kg)	0.00 ± 0.00	8.97 ± 2.1	0.00 ± 0.00	***	***	NS.
Water-soluble N (mg/kg)	128.31 ± 100.79	40.47 ± 27.62	119.69 ± 73.33	*	**	NS.
Water-soluble S (mg/kg)	38.47 ± 15.79	94.21 ± 35.8	29.24 ± 11.3	*	**	NS.
TN (g/kg)	3.94 ± 2.28	2.85 ± 0.4	3.61 ± 0.61	NS.	**	NS.
TS (g/kg)	8.87 ± 2.36	4.81 ± 0.21	6.86 ± 3.21	***	*	NS.
TC (g/kg)	50.47 ± 22.81	31.73 ± 3.46	37.37 ± 2.82	*	**	NS.

### 3.2 Composition of fungal community

The flattening pattern of rarefaction curves indicated sufficient sequencing depth for each sample (Fig. S2). 4644 operational taxonomic units (OTUs) were classified into 9 phyla, 37 class, 123 orders, 309 families, 867 genera, and 2006 species.

**Fig. 3:**
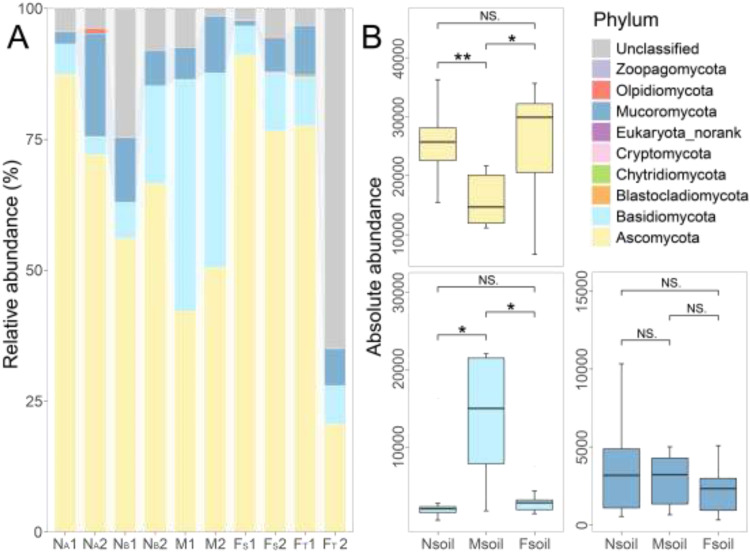
Average relative abundance of top ten fungal phyla in each sample (A). The absolute abundance of dominant fungal phylum in Nsoil, Msoil, and Fsoil (B). Note: “***” : *p* < 0.001, “**” : 0.001 < *p* < 0.01, “*” : 0.01 < *p* < 0.05, “NS.” : *p* > 0.05.

Ascomycota, Basidiomycota, and Mucoromycota were the three most dominant fungal phyla (Fig. 3A). The number of OTU for Ascomycota in the Nsoil and Fsoil were 25,703 and 25,197, respectively. They were significantly higher than that in the Msoil (15,852) (see Fig. 3B). In contrast, the number of OTU belonged to Basidiomycota in the Nsoil and Fsoil were 3156 and 3150, which were significantly lower than that (13,932) in the Msoil. Mucoromycota has the third highest relative abundance among all fungal phyla, with no difference among the Nsoil (3693), Msoil (2875), and Fsoil (2274). In addition, the abundances of the top ten genera in the Ascomycota and Basidiomycota phyla were presented in Tables S9 and S10.

No significant associations were observed between SOC concentrations and Ascomycota abundance in either the Nsoil (*p* = 0.95) or all soils (*p* = 0.20) (Fig. S3). Notably, water-soluble F demonstrated a significant negative correlation (*p* < 0.05) with Basidiomycota abundance across all the soil samples (Fig. S4).

### 3.3 Alpha and beta diversity of fungal community

**Fig. 4:**
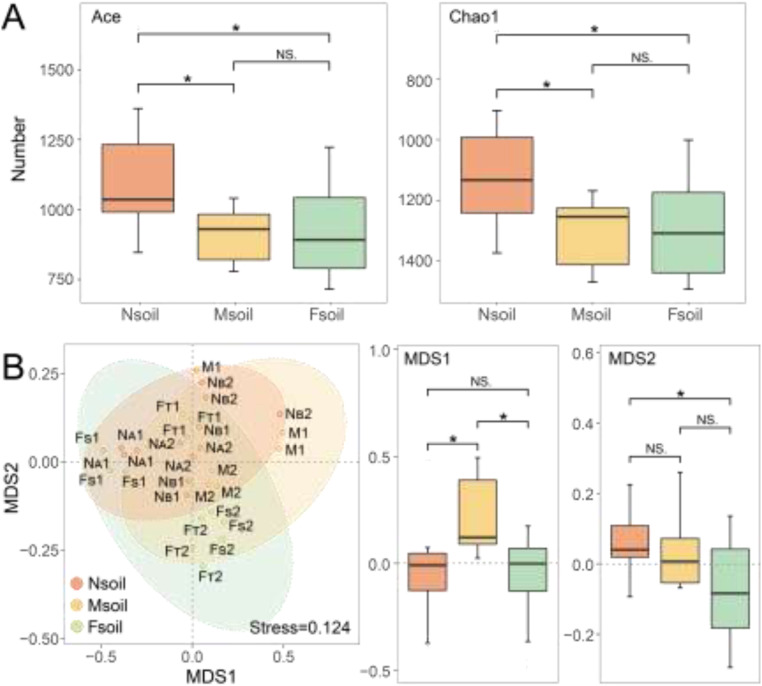
Fungal community alpha diversity index of the Nsoil, Msoil, and Fsoil (A). The dissimilarities of fungal community composition (beta diversity) among the Nsoil, Msoil, and Fsoil (B). Note: “***” : *p* < 0.001, “**” : 0.001 < *p* < 0.01, “*” : 0.01 < *p* < 0.05, “NS.” : *p* > 0.05.

The alpha diversity was characterized in terms of community richness (Chao1 and ACE), diversity (Shannon and Simpson), and evenness (Pielou). The richness (whether Chao1 = 1098.70 or ACE = 1118.23) of Nsoil was significantly higher than that of Msoil (Chao1 = 894.64 and ACE = 910.44) and Fsoil (Chao1 = 915.04 and ACE = 930.93) (Fig. 4A). Nevertheless, there was no significant difference in diversity and evenness among the Nsoil, Msoil, and Fsoil groups (Fig. S5). The specific alpha diversity data for each sample were shown in Table S8.

The NMDS results showed that the composition of fungal communities at the Nsoil, Msoil, and Fsoil were significantly different (stress = 0.124) (Fig. 4B). Specifically, in the NMDS1 dimension, the Msoil had significant difference from the Nsoil and Fsoil (Fig. 4B). In the NMDS2 dimension, there was significant difference between the Nsoil and Fsoil (Fig. 4B).

### 3.4 Fungal community assembly processes

**Fig. 5:**
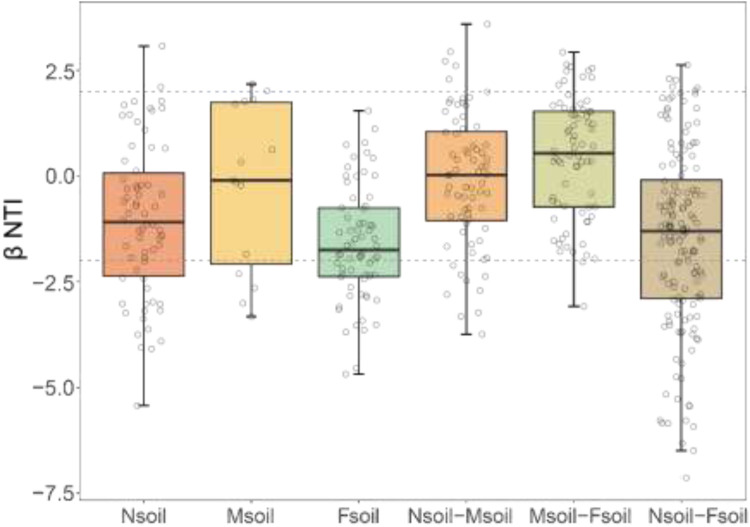
βNTI analysis showed the assembly processes of the fungal community in the Nsoil, Msoil, and Fsoil. Note: |βNTI| < 2 means stochastic community assembly process, and |βNTI| > 2 means deterministic community assembly process.

βNTI values for the fungal community were within the range of −2 and 2 in Nsoil, Msoil, and Fsoil (Fig. 5). This suggested that stochastic processes were dominant during the assembly of community. However, a subset of sample pairs exhibited |βNTI| > 2, which suggested a shift toward deterministic processes. Specifically, 21, 6, and 25 such pairs were identified within Nsoil, Msoil, and Fsoil, respectively. Furthermore, between groups, 14 (Nsoil–Msoil), 12 (Msoil–Fsoil), and 61 (Nsoil–Fsoil) pairs showed |βNTI| > 2. The pairs with |βNTI| > 2 between Nsoil–Fsoil suggested that distance from the phosphate ore increasingly promotes deterministic assembly in the fungal community (Fig. 5).

### 3.5 Influence of environmental factors on fungal community

**Fig. 6:**
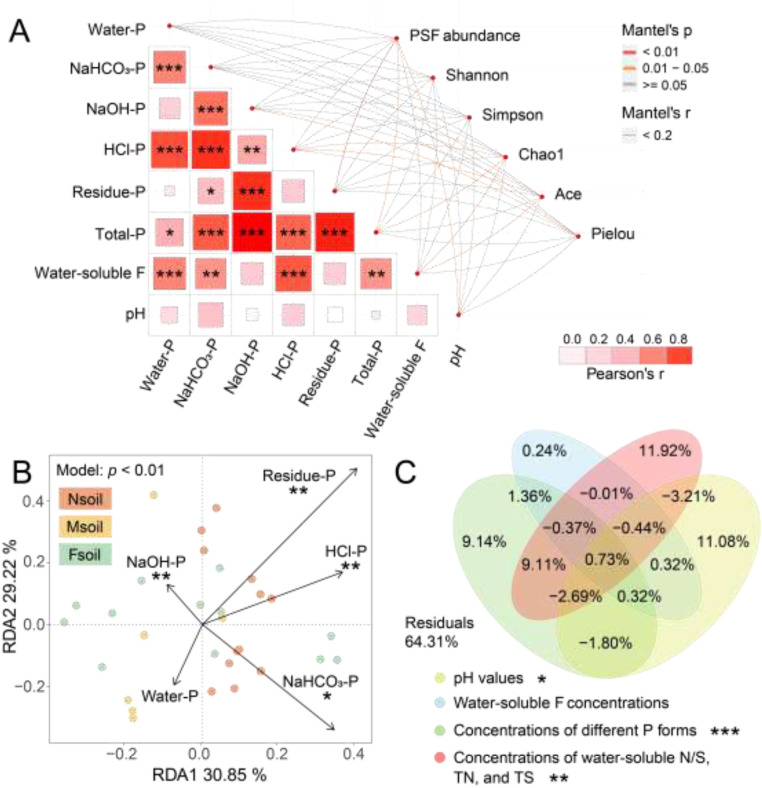
Relationships between fungal communities and environmental factors in all samples were analyzed by mantel analysis (A). DB-RDA analysis (B) showed the influence of environmental factors on the structure of fungal communities in Nsoil, Msoil, and Fsoil (environmental factor of concentrations of different P forms). VPA analysis (C) showed the correlation between four specific environmental factors and community structure. Note: “PSF abundance” represents the abundance of phosphate-solubilizing fungi. The different P forms contained the water-P, NaHCO3-P, NaOH-P, HCl-P, and residue-P. “***” : *p* < 0.001, “**” : 0.001 < *p* < 0.01, “*” : 0.01 < *p* < 0.05.

Mantel analysis showed significant positive correlations between water-soluble F and various P forms (Fig. 6A). Additionally, *Penicillium, Aspergillus*, and *Fusarium* were observed (Tian et al., 2019; Sarmah and Sarma, 2023), and their abundances were summarized as PSF abundances (Fig. 6A). The abundance of PSF was significantly correlated with residue P, total P, and pH (Fig. 6A). In addition, fungal community richness and evenness were significantly correlated with concentrations of NaOH-P, HCl-P, total P, water-soluble F, and pH value.

DB-RDA identified NaHCO₃-P, NaOH-P, HCl-P, and residual P as the main factors influencing fungal community structure (Fig. 6B). The overall model was significant, and the interpretabilities of the two axes (sum of RDA1 and RDA2) were higher than 60 %. Total P was excluded due to high expansion (expansion factors factor >10). VPA results further showed that different P forms, pH, and different N/S forms explained the community variation, with explanatory powers of 15.8 %, 4.31 %, and 15.04 %, respectively (Fig. 6C). Specifically, the P forms contained the water-P, NaHCO_3_-P, NaOH-P, HCl-P, and residue P concentrations. In addition, the different N/S forms contained the water-soluble N, water-soluble S, TN and TS concentrations.

**Fig. 7:**
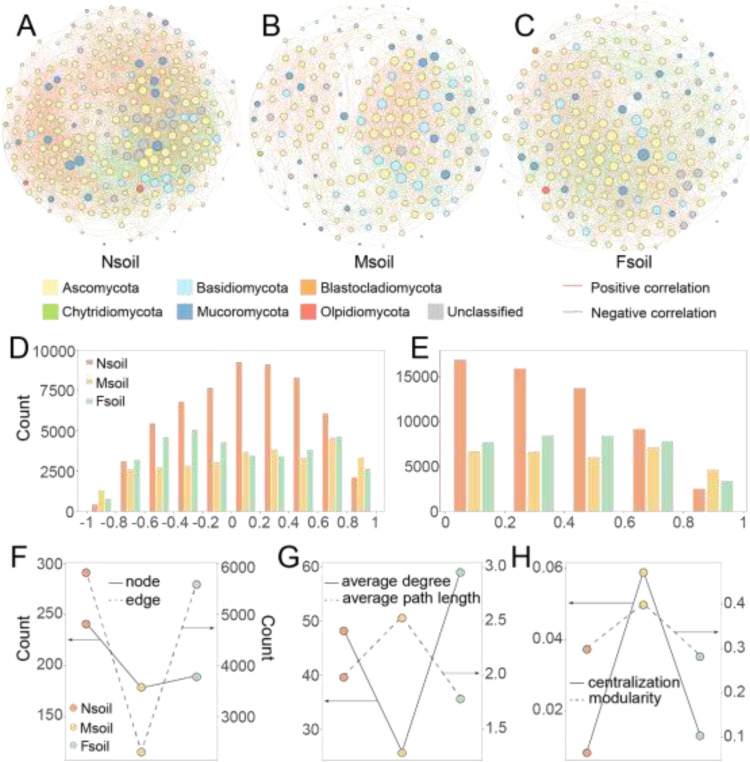
The fungal co-occurrence networks of Nsoil (A), Msoil (B), and Fsoil (C). The nodes with different colors represent the phylum they belong to (A-C). Count distributions of correlations in fungal co-occurrence networks (D: strength, E: absolute strength). Topological properties of co-occurrence networks (F: nodes and edges, G: average degree and average path length, H: centralization and modularity).

The co-occurrence networks were predominantly composed of Ascomycota, Basidiomycota, Blastocladiomycota, Chytridiomycota, Mucoromycota, and Olpidiomycota (Fig. 7A–C). Across the Nsoil, Msoil, and Fsoil, positive correlations accounted for 59.75 %, 59.98 %, and 50.06 % of all network connections, respectively, while negative correlations represented 40.25 %, 40.02 %, and 49.94 % (Fig. 7D). Overall, fungal OTUs in Nsoil showed weaker connections compared with those in Msoil and Fsoil (Fig. 7D, E), indicating their stronger potential fungal interactions. The topologies of three co-occurrence networks were displayed in Figs 7F–7H. The networks contained 242, 178, and 190 nodes and 5854, 2304, and 5621 edges under Nsoil, Msoil, and Fsoil, respectively (Fig. 7F). Furthermore, the Msoil network exhibited higher average path length, centralization, and modularity, but a lower average degree relative to Nsoil and Fsoil (Fig. 7G and 7H).

**Fig. 8:**
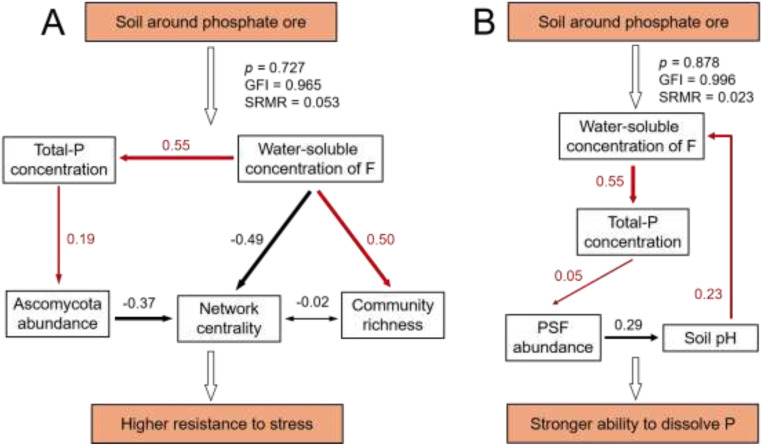
SEM analysis of soils around the phosphate rock on resistance to stress (A) and P dissolve ability (B) of fungal communities. Values near the arrows were path coefficients. Note: “PSF abundance” represents phosphate-solubilizing fungi abundance; network centrality the values of the network center were multiplied by 1000 in order to avoid large differences with other variables; red and black arrows denote significant positive and negative relationships (*p* < 0.05), respectively.

SEM quantified the effects of increasing water-soluble concentration of F caused by phosphate ore on the resistance to stress (Fig. 8A) and P solubility (Fig. 8B) of fungal communities. These results indicated that the increase of soil total P was observed alongside the rise in water-soluble concentration of F. The total-P further promoted the Ascomycota abundance. Additionally, water-soluble concentration of F has a significant negative effect (−0.49) on the centrality of the community network but has a positive impact (0.50) on the richness of the community (Fig. 8A). Fig. 8B showed that the total-P promoted the increase of the abundance of PSF and reduced the soil pH, thereby enhancing the phosphorus-solubilizing ability of the fungal community.

## 4 Discussion

The spatial distribution of soil P showed an increasing trend in concentrations of its various forms with the decreasing distance from phosphate ore (Fig. 2D). NaHCO_3_-P, NaOH-P, HCl-P, and residue-P may further emerge as critical environmental drivers in fungal communities. However, the changes correlated with the increase of P concentrations did not cause an imbalance in fungal community (Fig. 4B). This may be due to the low solubility of fluorapatite (Ksp = 1.0 × 10^−61^), which does not induce a sharp increase in soil P. Thus, the P concentrations in Nsoil was just relatively higher with respect to Msoil and Fsoil. In addition, the microbial community has a wide range of acceptance for P in the habitat (Magnan and Duvat, 2015; Stapanian et al., 2016). For example, the NaHCO_3_-P in this study (Nsoil up to ∼70 mg/kg) were analogical to that in agricultural soil with long-term fertilization (Ling et al., 2017), indicating that there was no evident non-point P pollution.

Fungal community was highly susceptible to dynamic P changes (Kaspari et al., 2008). Their richness was usually decreased with elevated P (Zhou et al., 2020; Ma et al., 2022). However, this study showed that the richness of the fungal community was enhanced in the soil near the phosphate ore (Figs. 2D, 4A, and 6). This may be due to those soils with high P concentrations near phosphate ore were often accompanied by elevated F. The water-soluble F in the Nsoil was ∼9 mg/kg (see Fig. 2C), which was significantly higher than background water-soluble F (∼2 mg/kg) in Guizhou (Wang et al., 2021). The elevated F may decrease the abundance of dominant fungi, thereby increasing the community richness (see Fig. 8). These data indicated that the effect of F on richness was greater than that of P supplement.

Fluorine, as an environmental stress, was also closely related to the stability of fungal communities. The strength of relationship between fungal OTUs was weak in the Nsoil (see Fig. 7D and 7E). Processes that weaken interactions between species will generally promote stability, as these processes reduce the coupling that drives instability (Coyte et al., 2015). Moreover, this enhanced stability was further evidenced by a reduced centralization within fungal network (Fig. 7). Previous modeling indicated that the decreased strength of some key interactions could enhance the stability of network (de Vries et al., 2018). This stability further affected the fungal community assembly process. Intensified environmental stress can cause community assembly to change from stochastic to deterministic, and the importance of deterministic processes increases with stress (Ning et al., 2024). Nevertheless, regardless of the distance from phosphate ore, the fungal community assembly was stochastic processes in this study (Fig. 5). This may be due to some stresses (such as drought) are so harmful or even lethal. Then, no species can sustain or gain any advantage (Jiao et al., 2021). This could also be ascribed to that the community has some resistance to stress and the assembly process at low stress remains random. Considering the environmental factors in this study, the stochastic assembly process of the community (see Fig. 5) should be caused by its own stress resistance. Therefore, the experimental evidence demonstrated that low-level F pollution may contribute to the enhanced structural stability in fungal communities.

Ascomycota, as one of the most common phyla of fungi, plays an important role in ecological balance (Maharachchikumbura et al., 2021). For instance, Ascomycota can form mycorrhizal relationships with plants (Halbwachs et al., 2021). However, SOC did not increase its abundance (Fig. S3). It was the concentration of P that significantly contributed to the abundance of Ascomycota (see Fig. 8A). Simultaneously, the fungal communities showed high resistance to F stress, which may also be related to the extensive degradation and metabolic characteristics of Ascomycota (Egidi et al., 2019). Additionally, Basidiomycota had a higher abundance in Msoil with the lowest F concentration (Fig. 3), and there was a significant negative correlation between them (Fig. S4). These evidences demonstrated that Basidiomycota, in contrast to Ascomycota, has an advantage in relatively lightly stressed environments (Fig. 2C) (Tian et al., 2017).

Beyond characterizing fungal community responses to phosphate ore resulted in F stress, this study further demonstrated their phosphate-solubilizing ability was improved near phosphate ore. The functional fungi in the soil that can solubilize PO_4_^3−^ from insoluble phosphate minerals are collectively referred to PSF (Tian et al., 2021). The PSF can secret of a variety of low molecular weight organic acids, e.g., gluconic acid, citric acid, oxalic acid, etc. (Su et al., 2021) and these organic acids can increase the solubility of geological fluorapatite (Meng et al., 2024b). Therefore, the PSF abundance was enhanced in soil fungi community near phosphate ore (see Fig. 8B). Subsequently, the secretion of more organic acids could further decrease the soil pH (Fig. 8B). As a positive feedback, the P released into the environment further promoted the growth of PSF (Zhang et al., 2019), which enhanced the potential of fungal communities to release P in the ecosystem near the phosphate ore, as well as speeded up the biogeochemical cycle of P in this region.

## 5 Conclusion

This study provided an analysis of the spatial distribution characteristics of P and F in soils surrounding phosphate mining areas. The results elucidated the impact of phosphate mining on the structure and function of soil fungal communities. It indicated that the soil near the phosphate ore has high concentrations of various P, and their concentrations decreased with the increase of distance from the phosphate ore. For the fungal community, the richness and Ascomycota abundance increased, and the correlation of co-occurrence networks was weak near phosphate ore, which indicated that these enhanced their resistance to environmental stress. Additionally, the abundance of PSF in soils near the phosphate ore significantly increased, and the soil pH decreased, thereby improving the phosphate-solubilizing capability of the fungal community. This study investigated fungal roles as both stress buffers and P-cycle drivers in the ecosystems around phosphate ore, highlighting the importance of microbial-informed mining management and monitoring of F pollution impacts.

## Authors contributions

Lingzi Meng, Limin Zhou, Meiyue Xu, and Zhen Li designed the research. Lingzi Meng, Kejin Ding, Bailun Liu, and Ying Wang conducted the experiments. Lingzi Meng, Zhijun Wang, Feng Guo, and Meiyue Xu analyzed the data. The first draft of the manuscript was written by Lingzi Meng, Limin Zhou, Zhen Li, and all authors commented on previous versions of the manuscript. All authors read and approved the final manuscript.

## Declaration of competing interest

The authors have no conflict of interest.

## Appendix Supplementary materials

Image, application 1
