# Supplementary material for: Effects of phosphate mining-induced P and F pollution on soil fungal communities: Spatial variations in structure and function
Source: Curr Res Microb Sci. 2025 Nov 8;9:100506. doi: 10.1016/j.crmicr.2025.100506 (PMC12657603; doi:10.1016/j.crmicr.2025.100506)
Supplement: Supplementary file 1 [file mmc1.docx]

Supporting Information

**Table S1.** Elemental contents of phosphate ore in Guizhou Province analyzed by X-ray fluorescence spectrometer (XRF).

| **Compound** | CaO | SiO_2_ | P_2_O_5_ | MgO | Al_2_O_3_ | Fe_2_O_3_ |
| --- | --- | --- | --- | --- | --- | --- |
| **m/m%** | 24.26 | 14.91 | 12.40 | 7.62 | 4.26 | 1.08 |
| **StdErr%** | 0.25 | 0.19 | 0.18 | 0.14 | 0.10 | 0.05 |
| **Compound** | K_2_O | F | Na_2_O | TiO_2_ | MnO |  |
| **m/m%** | 0.63 | 0.57 | 0.18 | 0.15 | 0.11 |  |
| **StdErr%** | 0.03 | 0.03 | 0.009 | 0.007 | 0.005 |  |

**Table S2.** The coordinates of sampling sites for this study.

| **Point** | **Longitude (°E)** | **Latitude (°N)** | **Point** | **Longitude (°E)** | **Latitude (°N)** |
| --- | --- | --- | --- | --- | --- |
| N_A_1 | 107.396426 | 27.029286 | M2 | 107.379568 | 27.025808 |
| N_A_2 | 107.393658 | 27.028291 | F_S_1 | 107.457416 | 27.074585 |
| N_B_1 | 107.388061 | 27.019518 | F_S_2 | 107.401535 | 27.073202 |
| N_B_2 | 107.381631 | 27.016739 | F_T_1 | 107.420783 | 27.076534 |
| M1 | 107.379403 | 27.023515 | F_T_2 | 107.419962 | 27.066273 |

**Table S3.** The soil chemical properties of the N_A_ and N_B_ sampling sites.

| **Treatment** | **N_A_** | **N_B_** |
| --- | --- | --- |
| Water-P (mg/kg) | 20.24 ± 5.41 a | 27.25 ± 5.92 a |
| **NaHCO_3_-P (mg/kg)** | **85.39 ± 7.44 a** | **51.58 ± 3.32 b** |
| NaOH-P (mg/kg) | 150.50 ± 4.25 a | 127.22 ± 35.92 a |
| HCl-P (mg/kg) | 301.59 ± 37.50 a | 245.47 ± 43.04 a |
| Residue-P (mg/kg) | 659.89 ± 52.78 a | 636.79 ± 172.43 a |
| Total-P (mg/kg) | 1217.62 ± 23.95 a | 1088.31 ± 160.43 a |
| **pH** | **7.71 ± 0.15 a** | **6.31 ± 0.27 b** |
| SOC (mg/kg) | 40.20 ± 5.25 a | 23.45 ± 5.75 a |
| SOM (mg/kg) | 69.30 ± 9.04 a | 40.43 ± 9.91 a |
| TC (g/kg) | 59.72 ± 5.05 a | 41.22 ± 11.45 a |
| TN (g/kg) | 4.73 ± 0.71 a | 3.16 ± 1.07 a |
| **TS (g/kg)** | **6.78 ± 0.38 b** | **10.96 ± 0.39 a** |
| Water-soluble F (mg/kg) | 7.58 ± 1.89 a | 8.42 ± 0.35 a |
| Water-soluble Cl (mg/kg) | 0.00 ± 0.00 a | 0.00 ± 0.00 a |
| Water-soluble N (mg/kg) | 154.63 ± 58.62 a | 101.99 ± 3.50 a |
| Water-soluble S (mg/kg) | 35.34 ± 6.43 a | 41.59 ± 6.80 a |

Note: values are means of three replicates ± SE.

**Table S4.** The soil chemical properties of the Msoil.

| **Treatment** | **Msoil** |
| --- | --- |
| Water-P (mg/kg) | 8.21 ± 2.84 |
| NaHCO_3_-P (mg/kg) | 34.68 ± 9.09 |
| NaOH-P (mg/kg) | 85.05 ± 21.36 |
| HCl-P (mg/kg) | 2.24 ± 2.24 |
| Residue-P (mg/kg) | 401.65 ± 4.74 |
| Total-P (mg/kg) | 531.84 ± 32.58 |
| pH | 7.46 ± 0.22 |
| SOC (mg/kg) | 34.87 ± 2.99 |
| SOM (mg/kg) | 60.12 ± 5.16 |
| TC (g/kg) | 575.01 ± 543.52 |
| TN (g/kg) | 2.85 ± 0.16 |
| TS (g/kg) | 4.81 ± 0.08 |
| Water-soluble F (mg/kg) | 0.86 ± 0.39 |
| Water-soluble Cl (mg/kg) | 2.85 ± 0.16 |
| Water-soluble N (mg/kg) | 40.47 ± 11.28 |
| Water-soluble S (mg/kg) | 94.21 ± 14.61 |

**Table S5.** The soil chemical properties of the F_S_ and F_T_ sampling sites.

| **Treatment** | **F_S_** | **F_T_** |
| --- | --- | --- |
| Water-P (mg/kg) | 2.09 ± 0.53 a | 5.95 ± 2.51 a |
| NaHCO_3_-P (mg/kg) | 16.34 ± 2.04 a | 17.21 ± 2.12 a |
| **NaOH-P (mg/kg)** | **22.51 ± 3.21 b** | **40.99 ± 1.39 a** |
| HCl-P (mg/kg) | 0.10 ± 0.10 a | 0.00 ± 0.00 a |
| Residue-P (mg/kg) | 376.18 ± 21.73 a | 296.20 ± 31.24 a |
| Total-P (mg/kg) | 417.23 ± 21.49 a | 360.35 ± 31.18 a |
| **pH** | **7.20 ± 0.12 a** | **5.99 ± 0.10 b** |
| SOC (mg/kg) | 29.47 ± 1.30 a | 35.30 ± 2.34 a |
| SOM (mg/kg) | 50.80 ± 2.25 a | 60.85 ± 4.04 a |
| TC (g/kg) | 36.13 ± 1.39 a | 38.62 ± 0.59 a |
| **TN (g/kg)** | **3.09 ± 0.16 b** | **4.13 ± 0.03 a** |
| **TS (g/kg)** | **9.02 ± 1.27 a** | **4.70 ± 0.54 b** |
| Water-soluble F (mg/kg) | 4.95 ± 1.22 a | 1.71 ± 0.83 a |
| Water-soluble Cl (mg/kg) | 0.00 ± 0.00 a | 0.00 ± 0.00 a |
| Water-soluble N (mg/kg) | 115.96 ± 7.03 a | 123.43 ± 43.78 a |
| **Water-soluble S (mg/kg)** | **21.52 ± 3.11 b** | **36.95 ± 3.65 a** |

Note: values are means of three replicates ± SE.

**Table S6.** Length distribution of valid sequences

| **Length** | **Sequences** | **Percent** |
| --- | --- | --- |
| 1-150 | 2 | 0.00% |
| 151-200 | 281258 | 22.92% |
| 201-250 | 731100 | 59.58% |
| 251-300 | 199941 | 16.29% |
| 301-350 | 14854 | 1.21% |

**Table S7.** Elemental contents of each soil sample analyzed by X-ray fluorescence spectrometer (XRF).

| **N_A_1** | | | **N_A_2** | | | **N_B_1** | | | **N_B_2** | | | **M1** | | |
| --- | --- | --- | --- | --- | --- | --- | --- | --- | --- | --- | --- | --- | --- | --- |
| **Compd** | **m/m%** | **StdErr%** | **Compd** | **m/m%** | **StdErr%** | **Compd** | **m/m%** | **StdErr%** | **Compd** | **m/m%** | **StdErr%** | **Compd** | **m/m%** | **StdErr%** |
| SiO_2_ | 40.030 | 0.320 | SiO_2_ | 34.060 | 0.290 | SiO_2_ | 31.750 | 0.280 | SiO_2_ | 45.380 | 0.340 | SiO_2_ | 46.180 | 0.340 |
| Al_2_O_3_ | 12.540 | 0.180 | Al_2_O_3_ | 12.880 | 0.180 | Al_2_O_3_ | 13.020 | 0.180 | Al_2_O_3_ | 10.040 | 0.160 | Al_2_O_3_ | 14.050 | 0.190 |
| Fe_2_O_3_ | 4.270 | 0.100 | CaO | 3.010 | 0.090 | CaO | 6.050 | 0.120 | Fe_2_O_3_ | 3.940 | 0.100 | Fe_2_O_3_ | 4.580 | 0.110 |
| CaO | 3.850 | 0.100 | Fe_2_O_3_ | 2.960 | 0.090 | K_2_O | 3.110 | 0.090 | CaO | 1.580 | 0.060 | K_2_O | 1.880 | 0.070 |
| MgO | 3.440 | 0.090 | K_2_O | 2.530 | 0.080 | P_2_O_5_ | 2.660 | 0.080 | MgO | 1.470 | 0.060 | MgO | 1.450 | 0.060 |
| P_2_O_5_ | 0.674 | 0.034 | MgO | 1.590 | 0.060 | MgO | 2.510 | 0.080 | K_2_O | 0.864 | 0.043 | TiO_2_ | 0.878 | 0.044 |
| K_2_O | 0.491 | 0.025 | P_2_O_5_ | 1.250 | 0.060 | Fe_2_O_3_ | 2.270 | 0.080 | P_2_O_5_ | 0.673 | 0.034 | CaO | 0.590 | 0.029 |
| TiO_2_ | 0.467 | 0.023 | TiO_2_ | 0.341 | 0.017 | TiO_2_ | 0.398 | 0.020 | TiO_2_ | 0.324 | 0.016 | Na_2_O | 0.269 | 0.013 |
| MnO | 0.207 | 0.010 | Na_2_O | 0.115 | 0.006 | Na_2_O | 0.204 | 0.010 | MnO | 0.134 | 0.007 | P_2_O_5_ | 0.248 | 0.012 |
| Na_2_O | 0.189 | 0.009 | MnO | 0.114 | 0.006 | MnO | 0.084 | 0.004 | Na_2_O | 0.115 | 0.006 | MnO | 0.102 | 0.005 |
| S | 0.042 | 0.002 | S | 0.059 | 0.003 | S | 0.081 | 0.004 | ZnO | 0.061 | 0.003 | S | 0.036 | 0.002 |
| ZnO | 0.022 | 0.001 | ZrO_2_ | 0.024 | 0.001 | ZrO_2_ | 0.025 | 0.001 | PbO | 0.027 | 0.001 | V_2_O_5_ | 0.023 | 0.001 |
| V_2_O_5_ | 0.013 | 0.001 | SrO | 0.014 | 0.001 | Au | 0.014 | 0.001 | V_2_O_5_ | 0.018 | 0.001 | ZrO_2_ | 0.019 | 0.001 |
| ZrO_2_ | 0.013 | 0.001 | ZnO | 0.008 | 0.000 | PtO_2_ | 0.009 | 0.001 | BaO | 0.014 | 0.004 | ZnO | 0.015 | 0.001 |
| Cr_2_O_3_ | 0.011 | 0.001 | Rb_2_O | 0.007 | 0.001 | Rb_2_O | 0.009 | 0.001 | As_2_O_3_ | 0.014 | 0.003 | Au | 0.012 | 0.001 |
| Au | 0.009 | 0.001 | CeO_2_ | 0.007 | 0.002 | ZnO | 0.008 | 0.000 | S | 0.013 | 0.001 | PtO_2_ | 0.010 | 0.001 |
| SrO | 0.007 | 0.001 | RuO_4_ | 0.005 | 0.001 | SrO | 0.007 | 0.001 | NiO | 0.013 | 0.001 | Cr_2_O_3_ | 0.009 | 0.000 |
| MoO_3_ | 0.006 | 0.001 | Au | 0.005 | 0.001 | RuO_4_ | 0.007 | 0.001 | Au | 0.010 | 0.001 | Rb_2_O | 0.008 | 0.001 |
| CeO_2_ | 0.006 | 0.002 | La_2_O_3_ | 0.004 | 0.001 | MoO_3_ | 0.007 | 0.001 | ZrO_2_ | 0.010 | 0.001 | SrO | 0.007 | 0.001 |
| PtO_2_ | 0.005 | 0.001 | Y_2_O_3_ | 0.004 | 0.001 | V_2_O_5_ | 0.006 | 0.001 | SrO | 0.007 | 0.001 | Co_3_O_4_ | 0.006 | 0.000 |
| NiO | 0.005 | 0.000 | Ar | 0.004 | 0.001 | Y_2_O_3_ | 0.005 | 0.001 | Co_3_O_4_ | 0.007 | 0.000 | NiO | 0.005 | 0.000 |
| Co_3_O_4_ | 0.005 | 0.000 | Bi_2_O_3_ | 0.004 | 0.001 | Ar | 0.004 | 0.001 | MoO_3_ | 0.007 | 0.001 | CuO | 0.005 | 0.000 |
| RuO_4_ | 0.005 | 0.001 | MoO_3_ | 0.004 | 0.001 | Co_3_O_4_ | 0.003 | 0.000 | PtO_2_ | 0.005 | 0.001 | La_2_O_3_ | 0.004 | 0.001 |
| Ar | 0.004 | 0.001 | V_2_O_5_ | 0.004 | 0.001 | CuO | 0.002 | 0.000 | Cr_2_O_3_ | 0.005 | 0.000 | Ar | 0.004 | 0.001 |
| Rb_2_O | 0.004 | 0.001 | Co_3_O_4_ | 0.003 | 0.000 | GeO_2_ | 0.001 | 0.001 | CuO | 0.005 | 0.000 | CeO_2_ | 0.003 | 0.001 |
| Y_2_O_3_ | 0.003 | 0.001 | CuO | 0.002 | 0.000 |  |  |  | Rb_2_O | 0.005 | 0.001 | Y_2_O_3_ | 0.002 | 0.001 |
| PbO | 0.003 | 0.001 | Yb_2_O_3_ | 0.003 | 0.001 |  |  |  | RuO_4_ | 0.004 | 0.001 | GeO_2_ | 0.001 | 0.001 |
| Nd_2_O_3_ | 0.003 | 0.001 |  |  |  |  |  |  | Y_2_O_3_ | 0.003 | 0.001 | Nd_2_O_3_ | 0.002 | 0.001 |
| Bi_2_O_3_ | 0.003 | 0.001 |  |  |  |  |  |  | Yb_2_O_3_ | 0.003 | 0.001 | Yb_2_O_3_ | 0.002 | 0.001 |
| CuO | 0.003 | 0.000 |  |  |  |  |  |  | Ar | 0.004 | 0.001 | Ga_2_O_3_ | 0.002 | 0.000 |
| GeO_2_ | 0.001 | 0.001 |  |  |  |  |  |  | La_2_O_3_ | 0.002 | 0.001 |  |  |  |
| Yb_2_O_3_ | 0.002 | 0.001 |  |  |  |  |  |  | Nd_2_O_3_ | 0.002 | 0.001 |  |  |  |
|  |  |  |  |  |  |  |  |  | Bi_2_O_3_ | 0.004 | 0.001 |  |  |  |
| **M2** | | | **F_S_1** | | | **F_S_2** | | | **F_T_1** | | | **F_T_2** | | |
| **Compd** | **m/m%** | **StdErr%** | **Compd** | **m/m%** | **StdErr%** | **Compd** | **m/m%** | **StdErr%** | **Compd** | **m/m%** | **StdErr%** | **Compd** | **m/m%** | **StdErr%** |
| SiO_2_ | 43.970 | 0.330 | SiO_2_ | 43.500 | 0.330 | SiO_2_ | 43.160 | 0.330 | SiO_2_ | 42.390 | 0.330 | SiO_2_ | 46.150 | 0.340 |
| Al_2_O_3_ | 16.370 | 0.200 | Al_2_O_3_ | 14.750 | 0.190 | Al_2_O_3_ | 15.130 | 0.190 | Al_2_O_3_ | 10.140 | 0.160 | Al_2_O_3_ | 11.800 | 0.170 |
| Fe_2_O_3_ | 4.890 | 0.110 | Fe_2_O_3_ | 5.860 | 0.120 | Fe_2_O_3_ | 5.460 | 0.120 | Fe_2_O_3_ | 3.960 | 0.100 | Fe_2_O_3_ | 3.470 | 0.090 |
| K_2_O | 2.050 | 0.070 | K_2_O | 2.400 | 0.080 | K_2_O | 2.440 | 0.080 | MgO | 1.690 | 0.070 | K_2_O | 1.560 | 0.060 |
| MgO | 1.490 | 0.060 | MgO | 1.610 | 0.060 | MgO | 1.790 | 0.070 | CaO | 1.070 | 0.050 | MgO | 1.030 | 0.050 |
| TiO_2_ | 0.741 | 0.037 | CaO | 1.340 | 0.060 | TiO_2_ | 0.741 | 0.037 | TiO_2_ | 0.424 | 0.021 | TiO_2_ | 0.806 | 0.040 |
| CaO | 0.450 | 0.023 | TiO_2_ | 1.080 | 0.050 | Na_2_O | 0.301 | 0.015 | K_2_O | 0.289 | 0.014 | CaO | 0.264 | 0.013 |
| P_2_O_5_ | 0.350 | 0.018 | Na_2_O | 0.245 | 0.012 | CaO | 0.245 | 0.012 | P_2_O_5_ | 0.236 | 0.012 | Na_2_O | 0.209 | 0.010 |
| Na_2_O | 0.330 | 0.017 | P_2_O_5_ | 0.145 | 0.007 | P_2_O_5_ | 0.203 | 0.010 | Na_2_O | 0.174 | 0.009 | P_2_O_5_ | 0.196 | 0.010 |
| MnO | 0.100 | 0.005 | MnO | 0.134 | 0.007 | MnO | 0.122 | 0.006 | MnO | 0.119 | 0.006 | MnO | 0.074 | 0.004 |
| S | 0.051 | 0.003 | S | 0.030 | 0.002 | S | 0.039 | 0.002 | S | 0.033 | 0.002 | S | 0.033 | 0.002 |
| Cl | 0.026 | 0.002 | ZrO_2_ | 0.028 | 0.001 | V_2_O_5_ | 0.025 | 0.001 | ZnO | 0.020 | 0.001 | V_2_O_5_ | 0.020 | 0.001 |
| V_2_O_5_ | 0.024 | 0.001 | V_2_O_5_ | 0.024 | 0.001 | ZrO_2_ | 0.019 | 0.001 | V_2_O_5_ | 0.013 | 0.001 | ZrO_2_ | 0.017 | 0.001 |
| ZrO_2_ | 0.021 | 0.001 | SrO | 0.017 | 0.001 | ZnO | 0.015 | 0.001 | ZrO_2_ | 0.012 | 0.001 | ZnO | 0.010 | 0.001 |
| ZnO | 0.014 | 0.001 | Rb_2_O | 0.011 | 0.001 | Rb_2_O | 0.012 | 0.001 | Au | 0.010 | 0.001 | BaO | 0.008 | 0.004 |
| Rb_2_O | 0.010 | 0.001 | ZnO | 0.011 | 0.001 | Au | 0.011 | 0.001 | PbO | 0.009 | 0.001 | Rb_2_O | 0.007 | 0.001 |
| Au | 0.009 | 0.001 | CeO_2_ | 0.010 | 0.002 | Cr_2_O_3_ | 0.010 | 0.001 | MoO_3_ | 0.006 | 0.001 | Au | 0.007 | 0.001 |
| Cr_2_O_3_ | 0.009 | 0.000 | Au | 0.010 | 0.001 | PtO_2_ | 0.010 | 0.001 | PtO_2_ | 0.006 | 0.001 | SrO | 0.006 | 0.001 |
| Co_3_O_4_ | 0.007 | 0.000 | Cr_2_O_3_ | 0.010 | 0.001 | BaO | 0.009 | 0.004 | Cr_2_O_3_ | 0.006 | 0.000 | Cr_2_O_3_ | 0.005 | 0.000 |
| SrO | 0.007 | 0.001 | CuO | 0.009 | 0.001 | Co_3_O_4_ | 0.007 | 0.000 | Ar | 0.005 | 0.001 | Re_2_O_7_ | 0.005 | 0.002 |
| PtO_2_ | 0.007 | 0.001 | Co_3_O_4_ | 0.007 | 0.000 | SrO | 0.007 | 0.001 | NiO | 0.005 | 0.000 | Co_3_O_4_ | 0.005 | 0.000 |
| MoO_3_ | 0.006 | 0.001 | PtO_2_ | 0.007 | 0.001 | NiO | 0.006 | 0.000 | RuO_4_ | 0.005 | 0.001 | NiO | 0.004 | 0.000 |
| CuO | 0.005 | 0.000 | NiO | 0.007 | 0.000 | CuO | 0.006 | 0.000 | Co_3_O_4_ | 0.004 | 0.000 | Ar | 0.004 | 0.001 |
| NiO | 0.005 | 0.000 | MoO_3_ | 0.006 | 0.001 | MoO_3_ | 0.005 | 0.001 | SrO | 0.003 | 0.001 | Bi_2_O_3_ | 0.004 | 0.001 |
| CeO_2_ | 0.005 | 0.002 | La_2_O_3_ | 0.005 | 0.001 | RuO_4_ | 0.005 | 0.001 | CeO_2_ | 0.003 | 0.001 | La_2_O_3_ | 0.003 | 0.001 |
| La_2_O_3_ | 0.003 | 0.001 | Y_2_O_3_ | 0.004 | 0.001 | La_2_O_3_ | 0.003 | 0.001 | La_2_O_3_ | 0.002 | 0.001 | RuO_4_ | 0.003 | 0.001 |
| Re_2_O_7_ | 0.004 | 0.002 | RuO_4_ | 0.004 | 0.001 | Ar | 0.004 | 0.001 | Y_2_O_3_ | 0.002 | 0.001 | Yb_2_O_3_ | 0.001 | 0.001 |
| RuO_4_ | 0.003 | 0.001 | Ar | 0.003 | 0.001 | PbO | 0.002 | 0.001 | CuO | 0.002 | 0.000 | MoO_3_ | 0.003 | 0.001 |
| Ar | 0.005 | 0.001 | Nd_2_O_3_ | 0.003 | 0.001 | Re_2_O_7_ | 0.004 | 0.001 | Bi_2_O_3_ | 0.003 | 0.001 | Y_2_O_3_ | 0.002 | 0.001 |
| Y_2_O_3_ | 0.003 | 0.001 | Bi_2_O_3_ | 0.003 | 0.001 | Y_2_O_3_ | 0.003 | 0.001 | Yb_2_O_3_ | 0.002 | 0.001 | CuO | 0.003 | 0.000 |
| GeO_2_ | 0.001 | 0.001 | PbO | 0.003 | 0.001 | GeO_2_ | 0.001 | 0.000 | GeO_2_ | 0.001 | 0.001 |  |  |  |
| Nd_2_O_3_ | 0.003 | 0.001 | Gd_2_O_3_ | 0.002 | 0.001 | Ga_2_O_3_ | 0.002 | 0.000 | Rb_2_O | 0.002 | 0.001 |  |  |  |
|  |  |  | Ga_2_O_3_ | 0.002 | 0.000 |  |  |  |  |  |  |  |  |  |
|  |  |  | Nb_2_O_5_ | 0.002 | 0.001 |  |  |  |  |  |  |  |  |  |

**Table S8.** Microbial alpha diversity indexes.

| **Treatment** | **Shannon** | **Simpson** | **Chao1** | **Ace** | **Pielou** | **Richness** | **Coverage** |
| --- | --- | --- | --- | --- | --- | --- | --- |
| N_A_1 | 2.84 ± 0.23 | 0.81 ± 0.02 | 921.14 ± 50.05 | 943.74 ± 69.2 | 0.3 ± 0.02 | 700.33 ± 57.5 | 99% |
| N_A_2 | 4.67 ± 0.18 | 0.96 ± 0 | 1184.99 ± 68.33 | 1202.95 ± 80.4 | 0.47 ± 0.02 | 1053 ± 47.51 | 99% |
| N_B_1 | 4.65 ± 0.41 | 0.96 ± 0.03 | 1334 ± 256.87 | 1369.1 ± 316.01 | 0.46 ± 0.05 | 1158.33 ± 205 | 99% |
| N_B_2 | 4.23 ± 1.27 | 0.94 ± 0.08 | 954.68 ± 113.17 | 957.14 ± 96.15 | 0.44 ± 0.12 | 785 ± 182.04 | 99% |
| M1 | 3.45 ± 1.1 | 0.9 ± 0.06 | 800.24 ± 116.77 | 830.04 ± 84.37 | 0.37 ± 0.1 | 647.67 ± 145.78 | 99% |
| M2 | 3.99 ± 0.38 | 0.92 ± 0.04 | 989.04 ± 38.51 | 990.84 ± 54.54 | 0.41 ± 0.04 | 840.67 ± 56.89 | 100% |
| F_S_1 | 2.93 ± 0.41 | 0.87 ± 0.05 | 789.62 ± 71.32 | 811.14 ± 51.74 | 0.32 ± 0.04 | 584 ± 79.9 | 99% |
| F_S_2 | 4.23 ± 0.19 | 0.92 ± 0.02 | 1036.18 ± 147.63 | 1034.75 ± 136.69 | 0.43 ± 0.02 | 878.67 ± 77.78 | 100% |
| F_T_1 | 3.97 ± 0.25 | 0.93 ± 0.02 | 745.56 ± 63.42 | 760.61 ± 50.43 | 0.43 ± 0.02 | 627.33 ± 58.05 | 99% |
| F_T_2 | 2.95 ± 0.53 | 0.81 ± 0.07 | 1088.79 ± 92.72 | 1117.23 ± 124.2 | 0.31 ± 0.05 | 812.67 ± 95.04 | 99% |

Values are means of three replicates ± SE.

**Table S9.** Abundance of phylum.

| **Phylum** | **F_S_1** | **F_S_2** | **F_T_1** | **F_T_2** | **N_A_1** | **N_A_2** | **N_B_1** | **N_B_2** | **M1** | **M2** |
| --- | --- | --- | --- | --- | --- | --- | --- | --- | --- | --- |
| Ascomycota | 26995.67 ± 586.12 | 6104 ± 763.96 | 22588.67 ± 1883.65 | 22919.33 ± 294.09 | 25812 ± 140.01 | 21456.67 ± 1059.79 | 16475 ± 1938.7 | 19664 ± 2196.31 | 12402 ± 2777.85 | 15091.67 ± 2278.81 |
| Basidiomycota | 1609 ± 249.2 | 2127 ± 237.23 | 3318 ± 1332.31 | 2769 ± 475.31 | 1720 ± 145.32 | 1065.67 ± 485.71 | 2024.67 ± 193.53 | 5516.67 ± 3950.14 | 13189.67 ± 5791.78 | 10762.67 ± 2777.97 |
| Blastocladiomycota | 0 ± 0 | 1.33 ± 0.88 | 0 ± 0 | 105.67 ± 25.77 | 2.33 ± 1.2 | 34 ± 27.57 | 0.33 ± 0.33 | 0.33 ± 0.33 | 0 ± 0 | 3 ± 3 |
| Chytridiomycota | 4.67 ± 4.18 | 9.33 ± 7.42 | 16 ± 8.5 | 7 ± 1.53 | 0.33 ± 0.33 | 2.67 ± 1.45 | 8 ± 4.73 | 3 ± 1.73 | 31 ± 30.5 | 5.67 ± 2.91 |
| Cryptomycota | 1.33 ± 0.88 | 0 ± 0 | 0 ± 0 | 0 ± 0 | 0 ± 0 | 0.33 ± 0.33 | 0 ± 0 | 0 ± 0 | 0 ± 0 | 0 ± 0 |
| Eukaryota_norank | 0 ± 0 | 2.67 ± 2.19 | 54 ± 30.89 | 0 ± 0 | 0 ± 0 | 0.33 ± 0.33 | 6.33 ± 6.33 | 0 ± 0 | 0.67 ± 0.67 | 0 ± 0 |
| Mucoromycota | 286 ± 65.13 | 2078.33 ± 980.3 | 1890.33 ± 150.59 | 2776 ± 414.07 | 703.33 ± 156.17 | 5604.67 ± 1408.96 | 3602.33 ± 946.88 | 1975.67 ± 793.78 | 1757.67 ± 1184.53 | 3240 ± 476.39 |
| Olpidiomycota | 37 ± 8.5 | 12.33 ± 2.85 | 75.67 ± 10.17 | 3.33 ± 2.4 | 19.33 ± 3.84 | 255.33 ± 126.65 | 45 ± 11 | 2 ± 1.15 | 2.67 ± 1.76 | 13.67 ± 11.26 |
| Unclassified | 619.67 ± 282.27 | 19219 ± 1922.36 | 1611.33 ± 506.5 | 971.33 ± 162.73 | 1293.67 ± 101.41 | 1133.33 ± 159.31 | 7377.67 ± 2745.31 | 2392 ± 1032.17 | 2165 ± 1800.38 | 437.33 ± 83.59 |
| Zoopagomycota | 0.67 ± 0.67 | 0 ± 0 | 0 ± 0 | 2.33 ± 2.33 | 3 ± 2.52 | 1 ± 0.58 | 14.67 ± 14.17 | 0.33 ± 0.33 | 5.33 ± 3.38 | 0 ± 0 |

**Table S10.** Abundance of class.

| **Class** | **F_S_1** | **F_S_2** | **F_T_1** | **F_T_2** | **N_A_1** | **N_A_2** | **N_B_1** | **N_B_2** | **M1** | **M2** |
| --- | --- | --- | --- | --- | --- | --- | --- | --- | --- | --- |
| Agaricomycetes | 1474.67 ± 210.17 | 693.33 ± 250.01 | 732.67 ± 134.69 | 345.33 ± 108.02 | 848.33 ± 339.75 | 337 ± 83.47 | 769 ± 112.12 | 4727.33 ± 4259.83 | 12531.67 ± 6018.88 | 3764 ± 2707.88 |
| Agaricostilbomycetes | 1.67 ± 1.67 | 1 ± 0.58 | 0.67 ± 0.33 | 0 ± 0 | 1.67 ± 1.2 | 1.33 ± 0.88 | 1.33 ± 1.33 | 8.33 ± 6.01 | 0 ± 0 | 5 ± 0.58 |
| Archaeorhizomycetes | 24282.67 ± 1343.46 | 1637.67 ± 112.48 | 687.67 ± 78.27 | 1902 ± 1079.9 | 22225.33 ± 741.27 | 622 ± 333.52 | 2052.33 ± 929.68 | 892 ± 866.53 | 2692 ± 1372.29 | 105 ± 66.68 |
| Arthoniomycetes | 1.67 ± 1.67 | 0 ± 0 | 0 ± 0 | 0 ± 0 | 0 ± 0 | 0 ± 0 | 0 ± 0 | 0 ± 0 | 0 ± 0 | 0 ± 0 |
| Ascomycota_norank | 155 ± 124.71 | 23.33 ± 5.61 | 82.67 ± 12.77 | 165.33 ± 71.42 | 70.67 ± 25.22 | 174.67 ± 43.18 | 128.33 ± 59.58 | 773.67 ± 587.9 | 123.67 ± 112.17 | 147 ± 89.23 |
| Atractiellomycetes | 1 ± 1 | 19.67 ± 5.84 | 0 ± 0 | 281.67 ± 149.45 | 0 ± 0 | 0.67 ± 0.67 | 53.67 ± 38.37 | 2.33 ± 2.33 | 0 ± 0 | 0.33 ± 0.33 |
| Basidiobolomycetes | 0 ± 0 | 0 ± 0 | 0 ± 0 | 0 ± 0 | 2.33 ± 2.33 | 0 ± 0 | 13.67 ± 13.67 | 0 ± 0 | 1.33 ± 0.88 | 0 ± 0 |
| Blastocladiomycetes | 0 ± 0 | 0 ± 0 | 0 ± 0 | 0 ± 0 | 0 ± 0 | 0.67 ± 0.67 | 0 ± 0 | 0 ± 0 | 0 ± 0 | 0.67 ± 0.67 |
| Chytridiomycetes | 4.67 ± 4.18 | 9.33 ± 7.42 | 7 ± 1.53 | 16 ± 8.5 | 0.33 ± 0.33 | 2.67 ± 1.45 | 8 ± 4.73 | 3 ± 1.73 | 31 ± 30.5 | 5.67 ± 2.91 |
| Classiculomycetes | 0 ± 0 | 0.67 ± 0.67 | 0 ± 0 | 0 ± 0 | 2 ± 1.53 | 0 ± 0 | 1 ± 1 | 0.33 ± 0.33 | 0 ± 0 | 0 ± 0 |
| Cryptomycota_norank | 1.33 ± 0.88 | 0 ± 0 | 0 ± 0 | 0 ± 0 | 0 ± 0 | 0.33 ± 0.33 | 0 ± 0 | 0 ± 0 | 0 ± 0 | 0 ± 0 |
| Cystobasidiomycetes | 0.33 ± 0.33 | 0 ± 0 | 17.33 ± 8.95 | 0 ± 0 | 0.67 ± 0.67 | 1.67 ± 1.2 | 2.67 ± 0.33 | 4.33 ± 2.96 | 0.33 ± 0.33 | 9.33 ± 5.61 |
| Dacrymycetes | 0.67 ± 0.67 | 0 ± 0 | 0 ± 0 | 0 ± 0 | 0 ± 0 | 0 ± 0 | 0 ± 0 | 0 ± 0 | 0 ± 0 | 0 ± 0 |
| Dothideomycetes | 694.67 ± 375.5 | 597.33 ± 203.13 | 2171 ± 561.69 | 4289 ± 1409.97 | 779.67 ± 210.9 | 2199.33 ± 713.06 | 2267 ± 164.21 | 1871 ± 722.39 | 1112.33 ± 626.79 | 1750.67 ± 360.26 |
| Entorrhizomycetes | 0 ± 0 | 2.67 ± 2.19 | 0 ± 0 | 54 ± 30.89 | 0 ± 0 | 0.33 ± 0.33 | 6.33 ± 6.33 | 0 ± 0 | 0 ± 0 | 0 ± 0 |
| Eukaryota_norank | 0 ± 0 | 0 ± 0 | 0 ± 0 | 0 ± 0 | 0 ± 0 | 0 ± 0 | 0 ± 0 | 0 ± 0 | 0.67 ± 0.67 | 0 ± 0 |
| Eurotiomycetes | 271.67 ± 83.31 | 634 ± 168.09 | 1327.33 ± 59.45 | 2363 ± 802.05 | 235 ± 53.7 | 1066.67 ± 131.7 | 1522.33 ± 335.39 | 6956.33 ± 1209.75 | 3528.33 ± 1473.83 | 2870.67 ± 752.77 |
| Exobasidiomycetes | 0 ± 0 | 0 ± 0 | 0 ± 0 | 0 ± 0 | 0 ± 0 | 0 ± 0 | 0 ± 0 | 0.67 ± 0.67 | 0 ± 0 | 0 ± 0 |
| Geminibasidiomycetes | 0.67 ± 0.67 | 3.67 ± 0.88 | 770.33 ± 181.55 | 4.33 ± 1.2 | 19.33 ± 5.84 | 270.67 ± 201.74 | 10 ± 4.16 | 0 ± 0 | 0 ± 0 | 7.33 ± 3.53 |
| Geoglossomycetes | 42.33 ± 16.22 | 0.33 ± 0.33 | 12.33 ± 9.4 | 0.33 ± 0.33 | 12.33 ± 4.33 | 2.67 ± 2.19 | 31.67 ± 30.17 | 93.67 ± 92.17 | 408.33 ± 348.53 | 3.33 ± 1.33 |
| Glomeromycetes | 92.33 ± 9.21 | 332.33 ± 62.25 | 48.33 ± 17.29 | 569.67 ± 128.36 | 185.67 ± 41.48 | 81.67 ± 10.97 | 215 ± 91.22 | 257.33 ± 45.82 | 213.67 ± 24.67 | 449.67 ± 63.88 |
| Kickxellomycetes | 0 ± 0 | 0 ± 0 | 2.33 ± 2.33 | 0 ± 0 | 0 ± 0 | 0 ± 0 | 0.33 ± 0.33 | 0 ± 0 | 0 ± 0 | 0 ± 0 |
| Laboulbeniomycetes | 1.33 ± 1.33 | 0.67 ± 0.67 | 2.33 ± 1.2 | 0 ± 0 | 0.67 ± 0.67 | 41 ± 22.14 | 0.33 ± 0.33 | 1.33 ± 1.33 | 1.33 ± 1.33 | 1.67 ± 0.88 |
| Lecanoromycetes | 4.33 ± 2.6 | 8 ± 4.16 | 15 ± 7.37 | 8.67 ± 4.18 | 1 ± 0.58 | 7 ± 6.03 | 31.67 ± 24.21 | 65.67 ± 58.71 | 18.67 ± 3.33 | 16 ± 3.51 |
| Leotiomycetes | 106.67 ± 26.98 | 315.33 ± 39.18 | 2091.67 ± 494.97 | 375.67 ± 119.47 | 62.67 ± 11.46 | 796 ± 242.92 | 453.33 ± 29.9 | 951.33 ± 420.05 | 485.67 ± 378.15 | 929.67 ± 348.27 |
| Malasseziomycetes | 0 ± 0 | 0.67 ± 0.67 | 2.33 ± 1.45 | 0 ± 0 | 0 ± 0 | 0.67 ± 0.67 | 2.67 ± 2.19 | 0 ± 0 | 1.33 ± 0.88 | 2.67 ± 1.33 |
| Microbotryomycetes | 16 ± 8.14 | 17.67 ± 6.36 | 27 ± 19.55 | 21.33 ± 11.67 | 7.33 ± 1.33 | 13 ± 9.17 | 16.33 ± 1.33 | 52 ± 38.89 | 12 ± 5.86 | 21.33 ± 6.57 |
| Mortierellomycetes | 193.67 ± 67.35 | 1746 ± 935.61 | 2725.67 ± 397.85 | 1320.67 ± 111.76 | 517.67 ± 116.12 | 5523 ± 1415.37 | 3387.33 ± 1035.21 | 1718.33 ± 754.42 | 1544 ± 1175.52 | 2783 ± 423.62 |
| Mucoromycetes | 0 ± 0 | 0 ± 0 | 2 ± 1.15 | 0 ± 0 | 0 ± 0 | 0 ± 0 | 0 ± 0 | 0 ± 0 | 0 ± 0 | 6 ± 6 |
| Mucoromycota_norank | 0 ± 0 | 0 ± 0 | 0 ± 0 | 0 ± 0 | 0 ± 0 | 0 ± 0 | 0 ± 0 | 0 ± 0 | 0 ± 0 | 1.33 ± 0.88 |
| Olpidiomycetes | 37 ± 8.5 | 12.33 ± 2.85 | 3.33 ± 2.4 | 75.67 ± 10.17 | 19.33 ± 3.84 | 255.33 ± 126.65 | 45 ± 11 | 2 ± 1.15 | 2.67 ± 1.76 | 13.67 ± 11.26 |
| Orbiliomycetes | 22.33 ± 13.35 | 15.67 ± 4.48 | 86 ± 8.02 | 114.33 ± 30.8 | 13 ± 6.35 | 110.67 ± 24.44 | 66.67 ± 11.1 | 403 ± 185.36 | 181.67 ± 140.2 | 76.67 ± 26.74 |
| Pezizomycetes | 57.33 ± 25.89 | 270.67 ± 51.22 | 42.67 ± 11.86 | 18 ± 3.51 | 18 ± 3.46 | 36 ± 7.81 | 116.67 ± 19.95 | 503.33 ± 415.37 | 577 ± 305.95 | 35.33 ± 19.62 |
| Physodermatomycetes | 0 ± 0 | 1.33 ± 0.88 | 105.67 ± 25.77 | 0 ± 0 | 2.33 ± 1.2 | 33.33 ± 27.95 | 0.33 ± 0.33 | 0.33 ± 0.33 | 0 ± 0 | 2.33 ± 2.33 |
| Pucciniomycetes | 0.67 ± 0.33 | 5.67 ± 2.4 | 2.67 ± 2.67 | 0.67 ± 0.33 | 18.33 ± 6.49 | 0.33 ± 0.33 | 2.33 ± 2.33 | 89 ± 49.74 | 120.33 ± 118.84 | 4.33 ± 2.33 |
| Saccharomycetes | 0 ± 0 | 2 ± 1.15 | 0.33 ± 0.33 | 0 ± 0 | 0 ± 0 | 2 ± 1.15 | 3.33 ± 2.4 | 6.33 ± 3.18 | 6 ± 4.58 | 4.67 ± 3.28 |
| Sordariomycetes | 1355.67 ± 283.41 | 2599 ± 659.97 | 16400.33 ± 883.2 | 13352.33 ± 2281.69 | 2392.67 ± 435.89 | 16397.67 ± 700.05 | 9795 ± 2489.97 | 7146.33 ± 3088.55 | 3267 ± 2239.74 | 9149.67 ± 1771.83 |
| Taphrinomycetes | 0 ± 0 | 0 ± 0 | 0 ± 0 | 0 ± 0 | 1 ± 1 | 1 ± 1 | 6.33 ± 6.33 | 0 ± 0 | 0 ± 0 | 1.33 ± 1.33 |
| Tremellomycetes | 113.33 ± 34.57 | 1384.67 ± 92.84 | 1215 ± 316.49 | 2664.67 ± 1279.76 | 822 ± 257.85 | 440 ± 193.76 | 1165.67 ± 217.55 | 630.67 ± 298.26 | 523.67 ± 118.11 | 6944.67 ± 1382.47 |
| Unclassified | 619.67 ± 282.27 | 19219 ± 1922.36 | 971.33 ± 162.73 | 1611.33 ± 506.5 | 1293.67 ± 101.41 | 1133.33 ± 159.31 | 7377.67 ± 2745.31 | 2392 ± 1032.17 | 2165 ± 1800.38 | 437.33 ± 83.59 |
| Ustilaginomycetes | 0 ± 0 | 0 ± 0 | 1 ± 1 | 0 ± 0 | 0.33 ± 0.33 | 0.33 ± 0.33 | 0 ± 0 | 1.67 ± 1.67 | 0.33 ± 0.33 | 3.67 ± 3.67 |
| Zoopagomycetes | 0.67 ± 0.67 | 0 ± 0 | 0 ± 0 | 0 ± 0 | 0.67 ± 0.33 | 1 ± 0.58 | 0.67 ± 0.67 | 0.33 ± 0.33 | 0.33 ± 0.33 | 0 ± 0 |
| Zoopagomycota_norank | 0 ± 0 | 0 ± 0 | 0 ± 0 | 0 ± 0 | 0 ± 0 | 0 ± 0 | 0 ± 0 | 0 ± 0 | 3.67 ± 3.67 | 0 ± 0 |

**Table S11.** Abundance of order.

| **Order** | **F_S_1** | **F_S_2** | **F_T_1** | **F_T_2** | **N_A_1** | **N_A_2** | **N_B_1** | **N_B_2** | **M1** | **M2** |
| --- | --- | --- | --- | --- | --- | --- | --- | --- | --- | --- |
| Abrothallales | 0 ± 0 | 0 ± 0 | 13.33 ± 13.33 | 0 ± 0 | 0.67 ± 0.67 | 1 ± 0.58 | 2.67 ± 1.45 | 0 ± 0 | 0 ± 0 | 0 ± 0 |
| Agaricales | 1119 ± 169.55 | 240.67 ± 96.87 | 409 ± 18.08 | 114 ± 63.22 | 69.33 ± 3.84 | 154.33 ± 55.53 | 164.67 ± 58.08 | 627.33 ± 503.07 | 2460.33 ± 2315.33 | 361 ± 88.84 |
| Agaricomycetes_norank | 0 ± 0 | 0 ± 0 | 0 ± 0 | 0 ± 0 | 0.67 ± 0.67 | 0 ± 0 | 0 ± 0 | 0 ± 0 | 0 ± 0 | 0 ± 0 |
| Agaricostilbales | 1.67 ± 1.67 | 0 ± 0 | 0.67 ± 0.33 | 1 ± 0.58 | 0.33 ± 0.33 | 1.33 ± 0.88 | 1.33 ± 1.33 | 8.33 ± 6.01 | 0 ± 0 | 4 ± 0.58 |
| Agaricostilbomycetes_norank | 0 ± 0 | 0 ± 0 | 0 ± 0 | 0 ± 0 | 1.33 ± 1.33 | 0 ± 0 | 0 ± 0 | 0 ± 0 | 0 ± 0 | 1 ± 1 |
| Amylocorticiales | 0 ± 0 | 0 ± 0 | 0 ± 0 | 1 ± 1 | 0 ± 0 | 0 ± 0 | 1.33 ± 1.33 | 0.33 ± 0.33 | 0 ± 0 | 0 ± 0 |
| Annulatascales | 0 ± 0 | 0.67 ± 0.67 | 0 ± 0 | 0 ± 0 | 0 ± 0 | 2.67 ± 1.76 | 2 ± 2 | 0 ± 0 | 0 ± 0 | 0.67 ± 0.67 |
| Archaeorhizomycetales | 24282.67 ± 1343.46 | 1902 ± 1079.9 | 687.67 ± 78.27 | 1637.67 ± 112.48 | 22225.33 ± 741.27 | 622 ± 333.52 | 2052.33 ± 929.68 | 892 ± 866.53 | 2692 ± 1372.29 | 105 ± 66.68 |
| Archaeosporales | 0 ± 0 | 9.33 ± 3.93 | 0 ± 0 | 1.67 ± 0.88 | 0 ± 0 | 1 ± 1 | 1.67 ± 1.2 | 0 ± 0 | 1 ± 1 | 2.33 ± 1.2 |
| Ascomycota_norank | 155 ± 124.71 | 165.33 ± 71.42 | 82.67 ± 12.77 | 23.33 ± 5.61 | 70.67 ± 25.22 | 174.67 ± 43.18 | 128.33 ± 59.58 | 773.67 ± 587.9 | 121.33 ± 113.36 | 147 ± 89.23 |
| Atheliales | 0 ± 0 | 0.67 ± 0.67 | 1 ± 1 | 0 ± 0 | 0 ± 0 | 1.67 ± 0.33 | 0 ± 0 | 0.33 ± 0.33 | 1 ± 0 | 23 ± 19.04 |
| Atractiellales | 1 ± 1 | 281.67 ± 149.45 | 0 ± 0 | 19.67 ± 5.84 | 0 ± 0 | 0.67 ± 0.67 | 53.67 ± 38.37 | 2.33 ± 2.33 | 0 ± 0 | 0.33 ± 0.33 |
| Auriculariales | 130.33 ± 29.36 | 8.67 ± 4.18 | 21 ± 17.09 | 7 ± 1 | 39.67 ± 14.44 | 7.33 ± 4.1 | 10.33 ± 4.67 | 5.33 ± 2.73 | 5 ± 2.52 | 8 ± 1.15 |
| Basidiobolales | 0 ± 0 | 0 ± 0 | 0 ± 0 | 0 ± 0 | 2.33 ± 2.33 | 0 ± 0 | 13.67 ± 13.67 | 0 ± 0 | 1.33 ± 0.88 | 0 ± 0 |
| Blastocladiales | 0 ± 0 | 0 ± 0 | 0 ± 0 | 0 ± 0 | 0 ± 0 | 0.67 ± 0.67 | 0 ± 0 | 0 ± 0 | 0 ± 0 | 0.67 ± 0.67 |
| Boletales | 0 ± 0 | 0 ± 0 | 1 ± 0.58 | 0 ± 0 | 0 ± 0 | 4 ± 2.65 | 4.67 ± 4.67 | 84.67 ± 84.67 | 247.33 ± 141.47 | 2.33 ± 1.86 |
| Boliniales | 0 ± 0 | 0 ± 0 | 0 ± 0 | 0 ± 0 | 0 ± 0 | 0 ± 0 | 0 ± 0 | 0 ± 0 | 0 ± 0 | 0 ± 0 |
| Botryosphaeriales | 6.67 ± 4.81 | 1.33 ± 0.88 | 93.67 ± 35.67 | 8.67 ± 3.18 | 38.33 ± 1.45 | 37 ± 24.06 | 13.67 ± 8.41 | 11 ± 2.52 | 5.33 ± 1.86 | 2 ± 0.58 |
| Calcarisporiellales | 0 ± 0 | 0 ± 0 | 0 ± 0 | 0 ± 0 | 0 ± 0 | 0 ± 0 | 0 ± 0 | 0 ± 0 | 0 ± 0 | 1.33 ± 0.88 |
| Caliciales | 0 ± 0 | 0 ± 0 | 0 ± 0 | 0 ± 0 | 0 ± 0 | 0 ± 0 | 0 ± 0 | 0 ± 0 | 0 ± 0 | 2 ± 2 |
| Calosphaeriales | 0 ± 0 | 2 ± 2 | 0.33 ± 0.33 | 0 ± 0 | 0.67 ± 0.67 | 3.67 ± 3.67 | 1 ± 1 | 0.33 ± 0.33 | 0 ± 0 | 474 ± 474 |
| Candelariales | 0 ± 0 | 0 ± 0 | 0 ± 0 | 0 ± 0 | 0 ± 0 | 0 ± 0 | 0 ± 0 | 0 ± 0 | 2.33 ± 2.33 | 0 ± 0 |
| Cantharellales | 60.67 ± 5.46 | 8.33 ± 1.45 | 122.33 ± 109.84 | 246.33 ± 100.52 | 221.67 ± 78.61 | 27.67 ± 14.44 | 222.67 ± 27.57 | 17.67 ± 8.97 | 11.33 ± 3.84 | 34 ± 12.22 |
| Capnodiales | 95.33 ± 63.4 | 800.67 ± 204.09 | 755.67 ± 264.72 | 153.33 ± 46.28 | 60.33 ± 12.68 | 371 ± 142.1 | 510.67 ± 98.34 | 595 ± 183.51 | 359 ± 115.38 | 907.33 ± 311.36 |
| Chaetosphaeriales | 5.67 ± 0.67 | 66.33 ± 16.05 | 178.33 ± 70.64 | 62 ± 33.41 | 10.67 ± 3.53 | 117.67 ± 35.48 | 44 ± 17.79 | 5 ± 1.15 | 5 ± 2.52 | 17.33 ± 8.88 |
| Chaetothyriales | 151 ± 20.98 | 1599 ± 576.67 | 1075.33 ± 50.72 | 337.33 ± 74.63 | 143 ± 53.78 | 479.67 ± 184.88 | 772.67 ± 181.58 | 2193 ± 1098 | 1456.33 ± 1122.26 | 2074 ± 962.58 |
| Chytridiales | 0 ± 0 | 0 ± 0 | 0.67 ± 0.67 | 0 ± 0 | 0 ± 0 | 0 ± 0 | 0 ± 0 | 0 ± 0 | 0 ± 0 | 0 ± 0 |
| Classiculales | 0 ± 0 | 0 ± 0 | 0 ± 0 | 0.67 ± 0.67 | 2 ± 1.53 | 0 ± 0 | 1 ± 1 | 0.33 ± 0.33 | 0 ± 0 | 0 ± 0 |
| Coniochaetales | 0 ± 0 | 24.33 ± 8.99 | 5.67 ± 3.71 | 1 ± 1 | 3.33 ± 1.86 | 29.33 ± 12.55 | 2 ± 1.53 | 3.33 ± 1.2 | 1.67 ± 1.67 | 4 ± 3.06 |
| Conioscyphales | 0 ± 0 | 1 ± 1 | 0 ± 0 | 0 ± 0 | 0 ± 0 | 0 ± 0 | 0 ± 0 | 0 ± 0 | 1.33 ± 1.33 | 0 ± 0 |
| Coronophorales | 1 ± 1 | 0 ± 0 | 0 ± 0 | 0 ± 0 | 0 ± 0 | 0 ± 0 | 0 ± 0 | 0 ± 0 | 0 ± 0 | 0 ± 0 |
| Corticiales | 14 ± 7.51 | 4.33 ± 1.67 | 34 ± 10.54 | 84.67 ± 26.67 | 471.33 ± 420.91 | 74 ± 21.28 | 141.67 ± 15.92 | 26 ± 12.66 | 26.33 ± 23.84 | 16.33 ± 10.4 |
| Cryptomycota_norank | 1.33 ± 0.88 | 0 ± 0 | 0 ± 0 | 0 ± 0 | 0 ± 0 | 0.33 ± 0.33 | 0 ± 0 | 0 ± 0 | 0 ± 0 | 0 ± 0 |
| Cystobasidiales | 0 ± 0 | 0 ± 0 | 0 ± 0 | 0 ± 0 | 0 ± 0 | 0.67 ± 0.33 | 0 ± 0 | 0 ± 0 | 0 ± 0 | 1 ± 1 |
| Cystobasidiomycetes_norank | 0.33 ± 0.33 | 0 ± 0 | 13.67 ± 5.78 | 0 ± 0 | 0.33 ± 0.33 | 0.33 ± 0.33 | 0.67 ± 0.67 | 1.67 ± 1.2 | 0.33 ± 0.33 | 4.67 ± 2.73 |
| Cystofilobasidiales | 1.33 ± 0.67 | 1.67 ± 1.2 | 158.67 ± 76.52 | 2.67 ± 0.88 | 5 ± 1.73 | 54.67 ± 14.05 | 126.67 ± 111.17 | 63.67 ± 29.04 | 12.67 ± 3.38 | 27.67 ± 5.24 |
| Dacrymycetales | 0.67 ± 0.67 | 0 ± 0 | 0 ± 0 | 0 ± 0 | 0 ± 0 | 0 ± 0 | 0 ± 0 | 0 ± 0 | 0 ± 0 | 0 ± 0 |
| Diaporthales | 12.33 ± 6.84 | 59 ± 34.53 | 13.33 ± 11.35 | 5.33 ± 3.84 | 6.67 ± 2.73 | 101 ± 54.88 | 34.67 ± 15.51 | 11 ± 4.73 | 5.67 ± 3.71 | 125 ± 87.51 |
| Diversisporales | 49.33 ± 13.42 | 175.33 ± 61.66 | 46 ± 17.24 | 241 ± 54.11 | 97.33 ± 20.99 | 70 ± 8.39 | 138.67 ± 58.81 | 171.33 ± 44.89 | 99.33 ± 20.54 | 190.33 ± 43.51 |
| Dothideales | 0.33 ± 0.33 | 0 ± 0 | 1 ± 1 | 0 ± 0 | 0 ± 0 | 0.33 ± 0.33 | 0 ± 0 | 3 ± 1.73 | 0 ± 0 | 0 ± 0 |
| Dothideomycetes_norank | 10.33 ± 2.91 | 675 ± 633.76 | 94 ± 71.49 | 39.67 ± 20.95 | 6.33 ± 1.45 | 15.33 ± 6.74 | 298 ± 275 | 83.67 ± 41.43 | 58 ± 54.05 | 76 ± 7.55 |
| Entorrhizales | 0 ± 0 | 54 ± 30.89 | 0 ± 0 | 2.67 ± 2.19 | 0 ± 0 | 0.33 ± 0.33 | 6.33 ± 6.33 | 0 ± 0 | 0 ± 0 | 0 ± 0 |
| Erysiphales | 9.67 ± 4.18 | 0.67 ± 0.33 | 24 ± 11.68 | 9.67 ± 6.89 | 0.33 ± 0.33 | 16.67 ± 5.81 | 13.67 ± 6.06 | 45.67 ± 25.41 | 9.33 ± 9.33 | 17.67 ± 4.67 |
| Erythrobasidiales | 0 ± 0 | 0 ± 0 | 3.67 ± 3.18 | 0 ± 0 | 0.33 ± 0.33 | 0.67 ± 0.67 | 2 ± 0.58 | 2.67 ± 1.76 | 0 ± 0 | 3.67 ± 2.03 |
| Eukaryota_norank | 0 ± 0 | 0 ± 0 | 0 ± 0 | 0 ± 0 | 0 ± 0 | 0 ± 0 | 0 ± 0 | 0 ± 0 | 0.67 ± 0.67 | 0 ± 0 |
| Eurotiales | 118 ± 64.39 | 754.67 ± 227.81 | 235 ± 17.62 | 139.33 ± 43.36 | 81.67 ± 19.97 | 540 ± 55.72 | 640.33 ± 154.77 | 1723 ± 855.74 | 679.67 ± 628.67 | 758.33 ± 372.1 |
| Eurotiomycetes_norank | 0 ± 0 | 0 ± 0 | 0 ± 0 | 0 ± 0 | 0 ± 0 | 0 ± 0 | 0 ± 0 | 0.67 ± 0.67 | 3 ± 0.58 | 0 ± 0 |
| Exobasidiales | 0 ± 0 | 0 ± 0 | 0 ± 0 | 0 ± 0 | 0 ± 0 | 0 ± 0 | 0 ± 0 | 0.67 ± 0.67 | 0 ± 0 | 0 ± 0 |
| Filobasidiales | 2 ± 1 | 187.33 ± 58.33 | 488 ± 309.8 | 50 ± 29.26 | 2 ± 0.58 | 118.67 ± 99.17 | 38.33 ± 16.38 | 103.67 ± 60.38 | 93.33 ± 85.34 | 174.33 ± 87.61 |
| Geminibasidiales | 0.67 ± 0.67 | 4.33 ± 1.2 | 770.33 ± 181.55 | 3.67 ± 0.88 | 19.33 ± 5.84 | 270.67 ± 201.74 | 10 ± 4.16 | 0 ± 0 | 0 ± 0 | 7.33 ± 3.53 |
| Geoglossales | 42.33 ± 16.22 | 0.33 ± 0.33 | 12.33 ± 9.4 | 0.33 ± 0.33 | 12.33 ± 4.33 | 2.67 ± 2.19 | 31.67 ± 30.17 | 93.67 ± 92.17 | 408.33 ± 348.53 | 3.33 ± 1.33 |
| Glomerales | 42.67 ± 12.71 | 357.33 ± 63.26 | 1.67 ± 0.33 | 70 ± 22.3 | 86 ± 23.44 | 9.67 ± 2.4 | 66.67 ± 27.79 | 57.67 ± 14.4 | 89.67 ± 34.69 | 229.67 ± 60.63 |
| Glomerellales | 26.67 ± 5.21 | 165.67 ± 67.02 | 579 ± 74.48 | 174.33 ± 29.49 | 205.67 ± 37.02 | 1701.67 ± 451.75 | 475.67 ± 61.01 | 141 ± 68.15 | 82.33 ± 67.34 | 1364.33 ± 1083.27 |
| Gomphales | 0 ± 0 | 0 ± 0 | 0 ± 0 | 0 ± 0 | 0 ± 0 | 0 ± 0 | 0 ± 0 | 1.67 ± 1.2 | 4 ± 3.51 | 53 ± 53 |
| Helotiales | 93 ± 23.59 | 303.67 ± 96.56 | 905 ± 110.77 | 111.67 ± 53.72 | 49.33 ± 8.57 | 571.33 ± 144 | 283.33 ± 70.82 | 874.33 ± 406.42 | 458.33 ± 372.1 | 874.33 ± 351.86 |
| Hymenochaetales | 0 ± 0 | 8.67 ± 4.91 | 0.33 ± 0.33 | 0.33 ± 0.33 | 0 ± 0 | 0.33 ± 0.33 | 1 ± 0.58 | 8 ± 7.51 | 0.67 ± 0.67 | 0.67 ± 0.33 |
| Hypocreales | 1100 ± 231.16 | 8664.67 ± 1991.01 | 4381.33 ± 659.69 | 1822 ± 542.74 | 1798.67 ± 408.39 | 8436.67 ± 2096.79 | 6284.33 ± 1552.68 | 5281.33 ± 2352.45 | 2556.67 ± 1756.94 | 5354 ± 911.96 |
| Kickxellales | 0 ± 0 | 0 ± 0 | 2.33 ± 2.33 | 0 ± 0 | 0 ± 0 | 0 ± 0 | 0.33 ± 0.33 | 0 ± 0 | 0 ± 0 | 0 ± 0 |
| Lecanorales | 0.67 ± 0.67 | 0 ± 0 | 0.67 ± 0.67 | 3.33 ± 3.33 | 0.33 ± 0.33 | 0 ± 0 | 0.67 ± 0.67 | 0.67 ± 0.67 | 8 ± 6.11 | 0.33 ± 0.33 |
| Lecanoromycetes_norank | 0.67 ± 0.33 | 4 ± 2.08 | 7 ± 3.46 | 1 ± 0.58 | 0 ± 0 | 4.67 ± 4.67 | 3.67 ± 0.88 | 64 ± 59.53 | 6.33 ± 5.36 | 1 ± 1 |
| Leotiomycetes_norank | 1.33 ± 0.33 | 70.33 ± 30.07 | 30.33 ± 16.58 | 184.67 ± 30.12 | 4.33 ± 2.4 | 65.67 ± 6.17 | 115.33 ± 57.92 | 30 ± 6.66 | 16.67 ± 6.67 | 36 ± 3.21 |
| Leucosporidiales | 0 ± 0 | 11.33 ± 8.51 | 11.33 ± 11.33 | 1 ± 1 | 0 ± 0 | 4.67 ± 4.67 | 1 ± 1 | 1 ± 1 | 0.33 ± 0.33 | 0 ± 0 |
| Lichenostigmatales | 1.67 ± 1.67 | 0 ± 0 | 0 ± 0 | 0 ± 0 | 0 ± 0 | 0 ± 0 | 0 ± 0 | 0 ± 0 | 0 ± 0 | 0 ± 0 |
| Lulworthiales | 7.67 ± 5.17 | 0 ± 0 | 3 ± 3 | 0 ± 0 | 15.33 ± 8.45 | 3.67 ± 3.18 | 0.67 ± 0.67 | 1 ± 1 | 0 ± 0 | 0 ± 0 |
| Magnaporthales | 0 ± 0 | 1 ± 1 | 0.33 ± 0.33 | 0 ± 0 | 0 ± 0 | 0 ± 0 | 0.67 ± 0.33 | 2.67 ± 2.67 | 0 ± 0 | 0.33 ± 0.33 |
| Malasseziales | 0 ± 0 | 0 ± 0 | 2.33 ± 1.45 | 0.67 ± 0.67 | 0 ± 0 | 0.67 ± 0.67 | 2.67 ± 2.19 | 0 ± 0 | 1.33 ± 0.88 | 2.67 ± 1.33 |
| Microascales | 1.33 ± 0.88 | 5.33 ± 1.86 | 46 ± 15.53 | 5 ± 2 | 5 ± 2.31 | 76.67 ± 27.96 | 123.33 ± 96.34 | 8.33 ± 3.53 | 4.33 ± 1.86 | 12 ± 8.02 |
| Microbotryales | 0 ± 0 | 0 ± 0 | 0 ± 0 | 0 ± 0 | 0 ± 0 | 0 ± 0 | 0.33 ± 0.33 | 0 ± 0 | 0 ± 0 | 0 ± 0 |
| Microbotryomycetes_norank | 7 ± 5.51 | 7.67 ± 3.53 | 2.67 ± 1.45 | 11.67 ± 3.93 | 1.33 ± 1.33 | 1.67 ± 1.67 | 5.33 ± 2.33 | 45.33 ± 37.89 | 10 ± 5.86 | 2 ± 1.53 |
| Minutisphaerales | 0 ± 0 | 0 ± 0 | 0 ± 0 | 0 ± 0 | 0 ± 0 | 0 ± 0 | 0 ± 0 | 0 ± 0 | 0 ± 0 | 0 ± 0 |
| Monoblastiales | 0 ± 0 | 0 ± 0 | 0 ± 0 | 0 ± 0 | 0 ± 0 | 5 ± 4.51 | 0.33 ± 0.33 | 0 ± 0 | 0 ± 0 | 0 ± 0 |
| Mortierellales | 193.67 ± 67.35 | 1320.67 ± 111.76 | 2725.67 ± 397.85 | 1746 ± 935.61 | 517.67 ± 116.12 | 5523 ± 1415.37 | 3387.33 ± 1035.21 | 1718.33 ± 754.42 | 1544 ± 1175.52 | 2783 ± 423.62 |
| Mucorales | 0 ± 0 | 0 ± 0 | 2 ± 1.15 | 0 ± 0 | 0 ± 0 | 0 ± 0 | 0 ± 0 | 0 ± 0 | 0 ± 0 | 6 ± 6 |
| Myriangiales | 3 ± 2.08 | 0 ± 0 | 0 ± 0 | 0.33 ± 0.33 | 0.67 ± 0.67 | 2 ± 1.15 | 0 ± 0 | 0.33 ± 0.33 | 0.67 ± 0.67 | 7.67 ± 5.7 |
| Myrmecridiales | 16.67 ± 4.81 | 167 ± 159.5 | 1.67 ± 0.33 | 11.33 ± 11.33 | 2.33 ± 1.45 | 3.33 ± 1.45 | 17 ± 12 | 9.67 ± 5.93 | 2 ± 1.53 | 5.67 ± 0.33 |
| Olpidiales | 37 ± 8.5 | 75.67 ± 10.17 | 3.33 ± 2.4 | 12.33 ± 2.85 | 19.33 ± 3.84 | 255.33 ± 126.65 | 45 ± 11 | 2 ± 1.15 | 2.67 ± 1.76 | 13.67 ± 11.26 |
| Onygenales | 1 ± 0.58 | 0.67 ± 0.33 | 1.33 ± 0.88 | 1 ± 0.58 | 3.67 ± 0.88 | 2 ± 0 | 13.33 ± 10.33 | 15 ± 12.12 | 7.33 ± 6.36 | 24.33 ± 5.55 |
| Ophiostomatales | 0 ± 0 | 0 ± 0 | 1 ± 1 | 0 ± 0 | 0 ± 0 | 8.33 ± 5.61 | 5.33 ± 4.84 | 1 ± 0.58 | 2 ± 2 | 7.67 ± 2.96 |
| Orbiliales | 22.33 ± 13.35 | 114.33 ± 30.8 | 86 ± 8.02 | 15.67 ± 4.48 | 13 ± 6.35 | 110.67 ± 24.44 | 66.67 ± 11.1 | 403 ± 185.36 | 181.67 ± 140.2 | 76.67 ± 26.74 |
| Ostropales | 0 ± 0 | 2 ± 1.15 | 0 ± 0 | 0 ± 0 | 0 ± 0 | 0 ± 0 | 0 ± 0 | 0 ± 0 | 0 ± 0 | 0 ± 0 |
| Paraglomerales | 0.33 ± 0.33 | 27.67 ± 13.35 | 0.67 ± 0.67 | 19.67 ± 1.45 | 2.33 ± 0.88 | 1 ± 0.58 | 8 ± 5.13 | 28.33 ± 13.59 | 23.67 ± 14.71 | 27.33 ± 7.88 |
| Peltigerales | 1 ± 1 | 2.33 ± 2.33 | 6 ± 2.65 | 3 ± 0.58 | 0 ± 0 | 2 ± 1.15 | 27 ± 25.03 | 0.33 ± 0.33 | 0 ± 0 | 0.33 ± 0.33 |
| Pezizales | 57.33 ± 25.89 | 18 ± 3.51 | 42.67 ± 11.86 | 270.67 ± 51.22 | 18 ± 3.46 | 36 ± 7.81 | 116.67 ± 19.95 | 503.33 ± 415.37 | 577 ± 305.95 | 35.33 ± 19.62 |
| Phaeomoniellales | 1 ± 0.58 | 3 ± 2.52 | 1.67 ± 0.33 | 0 ± 0 | 0 ± 0 | 33.33 ± 20.85 | 4.67 ± 2.91 | 6.67 ± 3.76 | 2.67 ± 1.2 | 1.67 ± 0.88 |
| Physodermatomycetes_norank | 0 ± 0 | 0 ± 0 | 105.67 ± 25.77 | 1.33 ± 0.88 | 2.33 ± 1.2 | 33.33 ± 27.95 | 0.33 ± 0.33 | 0.33 ± 0.33 | 0 ± 0 | 2.33 ± 2.33 |
| Pisorisporiales | 0 ± 0 | 0 ± 0 | 0.67 ± 0.67 | 0 ± 0 | 0 ± 0 | 4 ± 2.31 | 4 ± 4 | 4.67 ± 4.67 | 1.67 ± 1.2 | 0 ± 0 |
| Platygloeales | 0.33 ± 0.33 | 0.67 ± 0.33 | 0 ± 0 | 5.67 ± 2.4 | 13 ± 3.61 | 0 ± 0 | 2 ± 2 | 89 ± 49.74 | 120 ± 119 | 0.67 ± 0.33 |
| Pleosporales | 576 ± 308.48 | 2797.33 ± 1362.58 | 1162.67 ± 208.24 | 389.33 ± 186.16 | 671.67 ± 200.67 | 1760.67 ± 539.93 | 1434.67 ± 149.07 | 1125.33 ± 570.56 | 674.33 ± 526.95 | 755.33 ± 37.9 |
| Pleurotheciales | 0 ± 0 | 0 ± 0 | 0.67 ± 0.33 | 0 ± 0 | 0.33 ± 0.33 | 1.33 ± 1.33 | 2.33 ± 1.45 | 0 ± 0 | 0.67 ± 0.67 | 1.33 ± 1.33 |
| Polyporales | 40 ± 5.86 | 35 ± 2.65 | 31 ± 11.59 | 14 ± 6.51 | 21.67 ± 1.76 | 20 ± 6.93 | 44 ± 20.82 | 59.67 ± 30.03 | 16 ± 13.05 | 42 ± 12.5 |
| Pyxidiophorales | 1.33 ± 1.33 | 0 ± 0 | 2.33 ± 1.2 | 0.67 ± 0.67 | 0.67 ± 0.67 | 41 ± 22.14 | 0.33 ± 0.33 | 1.33 ± 1.33 | 1.33 ± 1.33 | 1.67 ± 0.88 |
| Ramicandelaberales | 0 ± 0 | 0 ± 0 | 0 ± 0 | 0 ± 0 | 0 ± 0 | 0 ± 0 | 0 ± 0 | 0 ± 0 | 3.67 ± 3.67 | 0 ± 0 |
| Rhizocarpales | 0 ± 0 | 0.33 ± 0.33 | 0 ± 0 | 0 ± 0 | 0 ± 0 | 0 ± 0 | 0 ± 0 | 0 ± 0 | 0 ± 0 | 0 ± 0 |
| Rhizophlyctidales | 4.67 ± 4.18 | 13.33 ± 8.45 | 1.33 ± 0.88 | 1.67 ± 0.88 | 0.33 ± 0.33 | 0.67 ± 0.33 | 2 ± 2 | 0 ± 0 | 29 ± 29 | 5.33 ± 3.18 |
| Rhizophydiales | 0 ± 0 | 2.67 ± 2.67 | 3 ± 0 | 7.67 ± 6.69 | 0 ± 0 | 1.67 ± 1.2 | 5 ± 3.06 | 3 ± 1.73 | 1.67 ± 1.67 | 0 ± 0 |
| Russulales | 105 ± 17.35 | 1 ± 0.58 | 22.67 ± 6.64 | 31 ± 15.95 | 12 ± 4.04 | 13 ± 6.56 | 62.67 ± 46.19 | 3251.33 ± 3191.84 | 9204.67 ± 4897.53 | 34 ± 13.61 |
| Saccharomycetales | 0 ± 0 | 0 ± 0 | 0.33 ± 0.33 | 2 ± 1.15 | 0 ± 0 | 2 ± 1.15 | 3.33 ± 2.4 | 6.33 ± 3.18 | 6 ± 4.58 | 4.67 ± 3.28 |
| Sarrameanales | 0 ± 0 | 0 ± 0 | 0 ± 0 | 0 ± 0 | 0 ± 0 | 0 ± 0 | 0 ± 0 | 0.67 ± 0.67 | 1.67 ± 1.67 | 0 ± 0 |
| Savoryellales | 0.33 ± 0.33 | 0 ± 0 | 11.67 ± 4.37 | 0.33 ± 0.33 | 0.33 ± 0.33 | 13.33 ± 9.94 | 3 ± 2.08 | 2.67 ± 2.67 | 0 ± 0 | 2 ± 1.15 |
| Sebacinales | 1.67 ± 0.67 | 5.33 ± 2.4 | 36.67 ± 9.74 | 2.33 ± 0.88 | 6 ± 3.21 | 8.67 ± 4.67 | 4 ± 2.08 | 53.67 ± 16.01 | 23.33 ± 16.33 | 358 ± 166.17 |
| Septobasidiales | 0.33 ± 0.33 | 0 ± 0 | 2.67 ± 2.67 | 0 ± 0 | 5.33 ± 3.93 | 0.33 ± 0.33 | 0.33 ± 0.33 | 0 ± 0 | 0.33 ± 0.33 | 3.67 ± 2.03 |
| Sordariales | 136.67 ± 26.91 | 3776.67 ± 560.13 | 9352.33 ± 1133.49 | 414 ± 101.73 | 246 ± 18.34 | 2935 ± 1620.81 | 1750 ± 258.54 | 642.67 ± 158.31 | 150 ± 35.93 | 1491.67 ± 350.67 |
| Sordariomycetes_norank | 15.33 ± 7.26 | 184.67 ± 58.07 | 1392.33 ± 1058.91 | 71.33 ± 15.71 | 39 ± 3.46 | 2769.67 ± 1176.51 | 845.67 ± 555.05 | 555 ± 264.76 | 294.67 ± 240.22 | 212.33 ± 33.89 |
| Spizellomycetales | 0 ± 0 | 0 ± 0 | 2 ± 1.15 | 0 ± 0 | 0 ± 0 | 0.33 ± 0.33 | 1 ± 1 | 0 ± 0 | 0.33 ± 0.33 | 0.33 ± 0.33 |
| Sporidiobolales | 9 ± 9 | 2.33 ± 0.33 | 13 ± 8 | 5 ± 2.31 | 6 ± 0 | 6.67 ± 3.18 | 9.67 ± 2.03 | 5.67 ± 2.85 | 1.67 ± 0.33 | 19.33 ± 7.88 |
| Strigulales | 0 ± 0 | 0 ± 0 | 0 ± 0 | 0 ± 0 | 1.33 ± 1.33 | 0 ± 0 | 0 ± 0 | 0 ± 0 | 0 ± 0 | 0 ± 0 |
| Taphrinales | 0 ± 0 | 0 ± 0 | 0 ± 0 | 0 ± 0 | 1 ± 1 | 1 ± 1 | 6.33 ± 6.33 | 0 ± 0 | 0 ± 0 | 1.33 ± 1.33 |
| Thelebolales | 2.67 ± 1.76 | 1 ± 0.58 | 1132.33 ± 579.9 | 9.33 ± 3.18 | 8.67 ± 2.91 | 142.33 ± 109.22 | 41 ± 23.39 | 1.33 ± 0.88 | 1.33 ± 0.88 | 1.67 ± 1.67 |
| Thelephorales | 2 ± 0.58 | 3 ± 1.15 | 41.33 ± 6.44 | 18 ± 9.02 | 4.33 ± 2.4 | 18 ± 4.51 | 14.33 ± 6.57 | 557.67 ± 545.68 | 512.33 ± 258.92 | 17 ± 3.21 |
| Togniniales | 0 ± 0 | 0 ± 0 | 0.67 ± 0.67 | 0 ± 0 | 0.67 ± 0.33 | 0 ± 0 | 0 ± 0 | 0 ± 0 | 0 ± 0 | 0 ± 0 |
| Trechisporales | 2 ± 1 | 29.67 ± 10.17 | 12.33 ± 5.24 | 174.67 ± 136.56 | 1.67 ± 0.88 | 8 ± 4.73 | 97.67 ± 59.61 | 33.67 ± 9.28 | 19.33 ± 2.19 | 2814 ± 2726.27 |
| Tremellales | 93.67 ± 39.19 | 2475.67 ± 1330.22 | 560.67 ± 187.59 | 1243 ± 105.37 | 209.67 ± 58.77 | 246 ± 70.68 | 895 ± 258.25 | 458 ± 206.18 | 413.33 ± 66.96 | 6735 ± 1403.01 |
| Tremellodendropsidales | 0 ± 0 | 0 ± 0 | 0 ± 0 | 0 ± 0 | 0 ± 0 | 0 ± 0 | 0 ± 0 | 0 ± 0 | 0 ± 0 | 0.67 ± 0.67 |
| Trichosphaeriales | 7.67 ± 2.33 | 182.33 ± 169.38 | 53.33 ± 10.11 | 13.33 ± 6.89 | 8.67 ± 3.76 | 48.67 ± 17.17 | 81.33 ± 34.62 | 63 ± 35.93 | 14.67 ± 12.17 | 12 ± 5 |
| Trichosporonales | 16.33 ± 7.54 | 0 ± 0 | 7.67 ± 3.18 | 89 ± 17.69 | 605.33 ± 261.02 | 20.67 ± 12.2 | 105.67 ± 53.01 | 5.33 ± 4.84 | 4.33 ± 2.33 | 7.67 ± 4.98 |
| Tubeufiales | 1.67 ± 0.33 | 0 ± 0 | 0 ± 0 | 0 ± 0 | 0 ± 0 | 0 ± 0 | 0 ± 0 | 0 ± 0 | 0 ± 0 | 0 ± 0 |
| Umbilicariales | 2 ± 1 | 0 ± 0 | 1.33 ± 0.88 | 0.67 ± 0.67 | 0.67 ± 0.67 | 0.33 ± 0.33 | 0.33 ± 0.33 | 0 ± 0 | 2.67 ± 2.67 | 12.33 ± 5.24 |
| Unclassified | 619.67 ± 282.27 | 1611.33 ± 506.5 | 971.33 ± 162.73 | 19219 ± 1922.36 | 1293.67 ± 101.41 | 1133.33 ± 159.31 | 7377.67 ± 2745.31 | 2392 ± 1032.17 | 2165 ± 1800.38 | 437.33 ± 83.59 |
| Ustilaginales | 0 ± 0 | 0 ± 0 | 1 ± 1 | 0 ± 0 | 0.33 ± 0.33 | 0.33 ± 0.33 | 0 ± 0 | 1.67 ± 1.67 | 0.33 ± 0.33 | 3.67 ± 3.67 |
| Venturiales | 1.33 ± 1.33 | 14.67 ± 3.53 | 50.67 ± 6.49 | 6 ± 3.61 | 0.33 ± 0.33 | 7 ± 4.73 | 7 ± 4.73 | 52.67 ± 44.67 | 15 ± 9.85 | 2.33 ± 1.2 |
| Verrucariales | 0.67 ± 0.67 | 5.67 ± 3.84 | 14 ± 8.39 | 156.33 ± 73.75 | 6.67 ± 2.33 | 11.67 ± 10.17 | 91.33 ± 20.51 | 3018 ± 2983 | 1379.33 ± 1249.98 | 12.33 ± 5.49 |
| Xenospadicoidales | 0 ± 0 | 0 ± 0 | 0 ± 0 | 0 ± 0 | 0 ± 0 | 0.67 ± 0.67 | 0 ± 0 | 0 ± 0 | 0 ± 0 | 0 ± 0 |
| Xylariales | 24.33 ± 6.39 | 51.67 ± 14.88 | 378.67 ± 2.73 | 19 ± 9.02 | 49.33 ± 8.45 | 140.33 ± 53.01 | 118 ± 69.53 | 413.67 ± 206.14 | 144.33 ± 117.34 | 65.33 ± 15.81 |
| Zoopagales | 0.67 ± 0.67 | 0 ± 0 | 0 ± 0 | 0 ± 0 | 0.67 ± 0.33 | 1 ± 0.58 | 0.67 ± 0.67 | 0.33 ± 0.33 | 0.33 ± 0.33 | 0 ± 0 |

**Table S12.** Abundance of family.

| Family | **F_S_1** | **F_S_2** | **F_T_1** | **F_T_2** | **N_A_1** | **N_A_2** | **N_B_1** | **N_B_2** | **M1** | **M2** |
| --- | --- | --- | --- | --- | --- | --- | --- | --- | --- | --- |
| Abrothallaceae | 0 ± 0 | 0 ± 0 | 13.33 ± 13.33 | 0 ± 0 | 0.67 ± 0.67 | 1 ± 0.58 | 2.67 ± 1.45 | 0 ± 0 | 0 ± 0 | 0 ± 0 |
| Agaricaceae | 0 ± 0 | 0.33 ± 0.33 | 0 ± 0 | 0 ± 0 | 0.33 ± 0.33 | 2 ± 2 | 6.33 ± 6.33 | 11.33 ± 2.19 | 2298 ± 2286.5 | 2.33 ± 1.45 |
| Agaricales_norank | 0 ± 0 | 0.33 ± 0.33 | 0 ± 0 | 0 ± 0 | 0 ± 0 | 3.67 ± 3.67 | 2 ± 2 | 0 ± 0 | 0 ± 0 | 0.33 ± 0.33 |
| Agaricomycetes_norank | 0 ± 0 | 0 ± 0 | 0 ± 0 | 0 ± 0 | 0.67 ± 0.67 | 0 ± 0 | 0 ± 0 | 0 ± 0 | 0 ± 0 | 0 ± 0 |
| Agaricostilbomycetes_norank | 0 ± 0 | 0 ± 0 | 0 ± 0 | 0 ± 0 | 1.33 ± 1.33 | 0 ± 0 | 0 ± 0 | 0 ± 0 | 0 ± 0 | 1 ± 1 |
| Alphamycetaceae | 0 ± 0 | 2.67 ± 2.67 | 2.33 ± 0.67 | 1.33 ± 0.67 | 0 ± 0 | 1.33 ± 1.33 | 0.67 ± 0.67 | 0.33 ± 0.33 | 0 ± 0 | 0 ± 0 |
| Amanitaceae | 0 ± 0 | 0 ± 0 | 0 ± 0 | 0 ± 0 | 0 ± 0 | 0 ± 0 | 0.67 ± 0.67 | 2.33 ± 2.33 | 10.33 ± 6.74 | 0 ± 0 |
| Ambisporaceae | 0 ± 0 | 1 ± 1 | 0 ± 0 | 0 ± 0 | 0 ± 0 | 0 ± 0 | 0 ± 0 | 0 ± 0 | 0 ± 0 | 0 ± 0 |
| Amphisphaeriaceae | 0 ± 0 | 0 ± 0 | 0.33 ± 0.33 | 0 ± 0 | 0 ± 0 | 5.33 ± 3.53 | 0.67 ± 0.67 | 0 ± 0 | 0 ± 0 | 0 ± 0 |
| Amylocorticiaceae | 0 ± 0 | 0 ± 0 | 0 ± 0 | 1 ± 1 | 0 ± 0 | 0 ± 0 | 1.33 ± 1.33 | 0.33 ± 0.33 | 0 ± 0 | 0 ± 0 |
| Annulatascaceae | 0 ± 0 | 0.67 ± 0.67 | 0 ± 0 | 0 ± 0 | 0 ± 0 | 2.67 ± 1.76 | 2 ± 2 | 0 ± 0 | 0 ± 0 | 0.67 ± 0.67 |
| Apiosporaceae | 0.33 ± 0.33 | 0 ± 0 | 0.67 ± 0.67 | 0 ± 0 | 0 ± 0 | 0 ± 0 | 13.33 ± 13.33 | 14 ± 9.07 | 8 ± 7.51 | 8.67 ± 1.86 |
| Aplosporellaceae | 0 ± 0 | 0 ± 0 | 0 ± 0 | 0 ± 0 | 0 ± 0 | 0 ± 0 | 0 ± 0 | 1.67 ± 1.67 | 0 ± 0 | 0 ± 0 |
| Archaeorhizomycetaceae | 24282.67 ± 1343.46 | 1902 ± 1079.9 | 687.67 ± 78.27 | 1637.67 ± 112.48 | 22225.33 ± 741.27 | 622 ± 333.52 | 2052.33 ± 929.68 | 892 ± 866.53 | 2692 ± 1372.29 | 105 ± 66.68 |
| Archaeosporaceae | 0 ± 0 | 8.33 ± 4.33 | 0 ± 0 | 1.67 ± 0.88 | 0 ± 0 | 1 ± 1 | 1.67 ± 1.2 | 0 ± 0 | 1 ± 1 | 2.33 ± 1.2 |
| Arizonaphlyctidaceae | 0.33 ± 0.33 | 3.67 ± 3.67 | 0 ± 0 | 0.67 ± 0.67 | 0 ± 0 | 0 ± 0 | 0 ± 0 | 0 ± 0 | 0 ± 0 | 0 ± 0 |
| Arthopyreniaceae | 0 ± 0 | 0 ± 0 | 0.67 ± 0.67 | 0 ± 0 | 0 ± 0 | 0 ± 0 | 0 ± 0 | 0 ± 0 | 0 ± 0 | 0 ± 0 |
| Arthrodermataceae | 0 ± 0 | 0.67 ± 0.33 | 1.33 ± 0.88 | 0 ± 0 | 0 ± 0 | 0 ± 0 | 1 ± 0.58 | 9.33 ± 9.33 | 2.67 ± 2.19 | 1 ± 0 |
| Ascobolaceae | 0 ± 0 | 0 ± 0 | 0 ± 0 | 0 ± 0 | 0 ± 0 | 1.67 ± 1.67 | 0 ± 0 | 0 ± 0 | 0 ± 0 | 0 ± 0 |
| Ascodesmidaceae | 0 ± 0 | 1 ± 0.58 | 0 ± 0 | 0 ± 0 | 2 ± 2 | 0 ± 0 | 0 ± 0 | 2.33 ± 2.33 | 1.67 ± 1.2 | 0 ± 0 |
| Ascomycota_norank | 155 ± 124.71 | 165.33 ± 71.42 | 82.67 ± 12.77 | 23.33 ± 5.61 | 70.67 ± 25.22 | 174.67 ± 43.18 | 128.33 ± 59.58 | 773.67 ± 587.9 | 121.33 ± 113.36 | 147 ± 89.23 |
| Ascosphaeraceae | 0 ± 0 | 0 ± 0 | 0 ± 0 | 0 ± 0 | 0 ± 0 | 0 ± 0 | 0 ± 0 | 4 ± 2.65 | 0 ± 0 | 0 ± 0 |
| Aspergillaceae | 116.33 ± 64.54 | 538.67 ± 201.77 | 209.33 ± 19.06 | 120.67 ± 39.56 | 79.67 ± 19.8 | 490.33 ± 41.01 | 561.33 ± 155.6 | 1637 ± 818.77 | 638.33 ± 592.85 | 749.33 ± 372.07 |
| Astraeaceae | 0 ± 0 | 0 ± 0 | 0.67 ± 0.67 | 0 ± 0 | 0 ± 0 | 0 ± 0 | 0 ± 0 | 0 ± 0 | 0 ± 0 | 0 ± 0 |
| Astrosphaeriellaceae | 289.33 ± 265.36 | 393.33 ± 180.07 | 49 ± 28.05 | 39.67 ± 25 | 53.67 ± 33.79 | 53.33 ± 16.83 | 129.67 ± 82.66 | 19.67 ± 11.72 | 30.33 ± 28.83 | 13 ± 6.81 |
| Atheliaceae | 0 ± 0 | 0.67 ± 0.67 | 1 ± 1 | 0 ± 0 | 0 ± 0 | 1.67 ± 0.33 | 0 ± 0 | 0.33 ± 0.33 | 1 ± 0 | 23 ± 19.04 |
| Atractosporaceae | 0 ± 0 | 0 ± 0 | 6 ± 3.06 | 4.67 ± 3.67 | 0 ± 0 | 49.67 ± 27.91 | 5 ± 2.89 | 23.67 ± 23.67 | 0.33 ± 0.33 | 44 ± 21.36 |
| Auriculariales_norank | 130.33 ± 29.36 | 6 ± 5.51 | 21 ± 17.09 | 0.33 ± 0.33 | 37.67 ± 13.92 | 5.33 ± 4.84 | 6.67 ± 3.18 | 5.33 ± 2.73 | 4.67 ± 2.33 | 2.67 ± 1.76 |
| Auriscalpiaceae | 101 ± 17.06 | 1 ± 0.58 | 13 ± 5.29 | 27.67 ± 13.93 | 11.67 ± 4.18 | 5.67 ± 2.85 | 37.33 ± 22.88 | 0.33 ± 0.33 | 0.67 ± 0.67 | 1 ± 1 |
| Barrmaeliaceae | 0 ± 0 | 0 ± 0 | 0 ± 0 | 0 ± 0 | 0 ± 0 | 1 ± 1 | 0 ± 0 | 0 ± 0 | 0 ± 0 | 0 ± 0 |
| Basidiobolaceae | 0 ± 0 | 0 ± 0 | 0 ± 0 | 0 ± 0 | 2.33 ± 2.33 | 0 ± 0 | 13.67 ± 13.67 | 0 ± 0 | 1.33 ± 0.88 | 0 ± 0 |
| Beltraniaceae | 0 ± 0 | 0 ± 0 | 0 ± 0 | 0 ± 0 | 0 ± 0 | 5.67 ± 5.67 | 1 ± 1 | 1.33 ± 1.33 | 1 ± 1 | 6.67 ± 5.17 |
| Biatriosporaceae | 0 ± 0 | 0 ± 0 | 0 ± 0 | 0 ± 0 | 0 ± 0 | 30 ± 16.52 | 0 ± 0 | 0 ± 0 | 0 ± 0 | 0 ± 0 |
| Bionectriaceae | 48.67 ± 7.17 | 180.67 ± 72.78 | 164.67 ± 93.19 | 20.67 ± 7.36 | 37 ± 7 | 78.67 ± 6.84 | 70.67 ± 20.34 | 301.33 ± 134.59 | 88.67 ± 79.23 | 22.67 ± 5.78 |
| Bolbitiaceae | 0 ± 0 | 0 ± 0 | 5.33 ± 2.33 | 0 ± 0 | 0 ± 0 | 2 ± 1.53 | 0.33 ± 0.33 | 0 ± 0 | 0 ± 0 | 0 ± 0 |
| Boletaceae | 0 ± 0 | 0 ± 0 | 0.33 ± 0.33 | 0 ± 0 | 0 ± 0 | 0 ± 0 | 0 ± 0 | 0 ± 0 | 0 ± 0 | 2 ± 2 |
| Boliniaceae | 0 ± 0 | 0 ± 0 | 0 ± 0 | 0 ± 0 | 0 ± 0 | 0 ± 0 | 0 ± 0 | 0 ± 0 | 0 ± 0 | 0 ± 0 |
| Botryobasidiaceae | 0 ± 0 | 0 ± 0 | 0 ± 0 | 0 ± 0 | 0 ± 0 | 0 ± 0 | 0 ± 0 | 3.33 ± 3.33 | 8.67 ± 5.55 | 0 ± 0 |
| Botryosphaeriaceae | 6.67 ± 4.81 | 1.33 ± 0.88 | 93.67 ± 35.67 | 8.33 ± 2.85 | 37.67 ± 1.67 | 37 ± 24.06 | 13.67 ± 8.41 | 9.33 ± 0.88 | 5.33 ± 1.86 | 2 ± 0.58 |
| Bulleraceae | 1.67 ± 1.67 | 0 ± 0 | 22.33 ± 22.33 | 1 ± 1 | 0.33 ± 0.33 | 0.67 ± 0.67 | 2 ± 1.15 | 0 ± 0 | 0 ± 0 | 0.67 ± 0.67 |
| Bulleribasidiaceae | 16.67 ± 11.68 | 24 ± 4.04 | 219 ± 143.54 | 60.67 ± 27.67 | 85 ± 32.7 | 60 ± 11 | 93 ± 38.02 | 25 ± 11.79 | 28.67 ± 26.17 | 423.33 ± 166.3 |
| Cainiaceae | 0 ± 0 | 0 ± 0 | 0 ± 0 | 0 ± 0 | 0.33 ± 0.33 | 0 ± 0 | 0 ± 0 | 0.33 ± 0.33 | 0 ± 0 | 0 ± 0 |
| Calcarisporiellaceae | 0 ± 0 | 0 ± 0 | 0 ± 0 | 0 ± 0 | 0 ± 0 | 0 ± 0 | 0 ± 0 | 0 ± 0 | 0 ± 0 | 1.33 ± 0.88 |
| Calosphaeriaceae | 0 ± 0 | 2 ± 2 | 0.33 ± 0.33 | 0 ± 0 | 0.67 ± 0.67 | 3.67 ± 3.67 | 1 ± 1 | 0.33 ± 0.33 | 0 ± 0 | 474 ± 474 |
| Candelariaceae | 0 ± 0 | 0 ± 0 | 0 ± 0 | 0 ± 0 | 0 ± 0 | 0 ± 0 | 0 ± 0 | 0 ± 0 | 2.33 ± 2.33 | 0 ± 0 |
| Cantharellales_norank | 1.67 ± 1.67 | 0.33 ± 0.33 | 0.33 ± 0.33 | 0.33 ± 0.33 | 1.33 ± 0.67 | 17 ± 13.53 | 2.67 ± 1.33 | 0 ± 0 | 0 ± 0 | 1.67 ± 1.67 |
| Capnodiaceae | 0 ± 0 | 0 ± 0 | 1.33 ± 1.33 | 0 ± 0 | 0 ± 0 | 0 ± 0 | 0 ± 0 | 0 ± 0 | 0 ± 0 | 0 ± 0 |
| Catenariaceae | 0 ± 0 | 0 ± 0 | 0 ± 0 | 0 ± 0 | 0 ± 0 | 0.67 ± 0.67 | 0 ± 0 | 0 ± 0 | 0 ± 0 | 0.67 ± 0.67 |
| Cenangiaceae | 0 ± 0 | 0 ± 0 | 0 ± 0 | 0 ± 0 | 0 ± 0 | 0.67 ± 0.67 | 0 ± 0 | 1.67 ± 1.67 | 3.67 ± 3.67 | 1.33 ± 1.33 |
| Cephalothecaceae | 0 ± 0 | 0.33 ± 0.33 | 4.33 ± 3.38 | 3.67 ± 2.19 | 1.33 ± 0.33 | 30.33 ± 14.26 | 8.33 ± 4.18 | 2.67 ± 2.19 | 1 ± 1 | 3.67 ± 3.18 |
| Ceratobasidiaceae | 57 ± 3.51 | 1.33 ± 1.33 | 118 ± 111.53 | 15.33 ± 5.24 | 220 ± 79.15 | 8 ± 4.04 | 120.67 ± 76.21 | 14.33 ± 10.35 | 2.67 ± 2.19 | 32.33 ± 13.37 |
| Chaetomellaceae | 0 ± 0 | 29 ± 3.79 | 2.33 ± 2.33 | 0 ± 0 | 0 ± 0 | 0.67 ± 0.67 | 3.67 ± 2.03 | 3.67 ± 2.73 | 7 ± 7 | 0.67 ± 0.67 |
| Chaetomiaceae | 35.33 ± 12.67 | 3212 ± 699.95 | 975.33 ± 446.61 | 193.67 ± 52.01 | 49 ± 11.79 | 542 ± 111.12 | 1147.67 ± 245.94 | 338 ± 75.01 | 51.67 ± 11.26 | 261 ± 55.34 |
| Chaetosphaeriaceae | 1.67 ± 0.33 | 66.33 ± 16.05 | 178.33 ± 70.64 | 62 ± 33.41 | 10.67 ± 3.53 | 117.67 ± 35.48 | 40.33 ± 21.23 | 5 ± 1.15 | 5 ± 2.52 | 17.33 ± 8.88 |
| Chaetosphaeriales_norank | 4 ± 0.58 | 0 ± 0 | 0 ± 0 | 0 ± 0 | 0 ± 0 | 0 ± 0 | 3.67 ± 3.67 | 0 ± 0 | 0 ± 0 | 0 ± 0 |
| Chaetothyriaceae | 0 ± 0 | 9 ± 4.93 | 0.67 ± 0.67 | 3 ± 2.08 | 0 ± 0 | 1 ± 1 | 4 ± 2.31 | 60.33 ± 35.57 | 29.67 ± 29.17 | 31.33 ± 17.37 |
| Chaetothyriales_norank | 1.67 ± 1.2 | 7.33 ± 7.33 | 22 ± 10.26 | 4.33 ± 2.4 | 0 ± 0 | 4.33 ± 2.85 | 8.67 ± 2.19 | 2 ± 1.15 | 4 ± 3.51 | 17 ± 7.77 |
| Chionosphaeraceae | 1.67 ± 1.67 | 0 ± 0 | 0.33 ± 0.33 | 1 ± 0.58 | 0.33 ± 0.33 | 1.33 ± 0.88 | 1.33 ± 1.33 | 8 ± 6.11 | 0 ± 0 | 0 ± 0 |
| Chrysozymaceae | 7 ± 5.51 | 7.67 ± 3.53 | 2.67 ± 1.45 | 11.67 ± 3.93 | 1.33 ± 1.33 | 1.33 ± 1.33 | 5 ± 2.65 | 44 ± 36.56 | 10 ± 5.86 | 0.33 ± 0.33 |
| Chytriomycetaceae | 0 ± 0 | 0 ± 0 | 0.67 ± 0.67 | 0 ± 0 | 0 ± 0 | 0 ± 0 | 0 ± 0 | 0 ± 0 | 0 ± 0 | 0 ± 0 |
| Cladosporiaceae | 81.33 ± 60.84 | 114.67 ± 67.85 | 497.33 ± 296.47 | 56 ± 21.63 | 34 ± 7.81 | 230.33 ± 69.68 | 220 ± 75.02 | 189 ± 91.09 | 81.67 ± 63.11 | 240.33 ± 97.11 |
| Claroideoglomeraceae | 0 ± 0 | 1.33 ± 1.33 | 0.33 ± 0.33 | 0 ± 0 | 3 ± 1.15 | 0.33 ± 0.33 | 1 ± 0.58 | 1.33 ± 0.33 | 1 ± 0.58 | 0 ± 0 |
| Classiculaceae | 0 ± 0 | 0 ± 0 | 0 ± 0 | 0.67 ± 0.67 | 2 ± 1.53 | 0 ± 0 | 1 ± 1 | 0.33 ± 0.33 | 0 ± 0 | 0 ± 0 |
| Clavariaceae | 790.33 ± 167.69 | 34.67 ± 20.34 | 43.67 ± 8.67 | 52.67 ± 26.82 | 38 ± 5.13 | 18.33 ± 10.87 | 42 ± 15.31 | 506 ± 503 | 69.33 ± 42.53 | 12 ± 8.02 |
| Clavicipitaceae | 71.67 ± 19.68 | 103 ± 4.58 | 884.67 ± 466.68 | 209 ± 85.78 | 93.67 ± 20.95 | 653.33 ± 183.05 | 382.67 ± 126.72 | 559.67 ± 286.16 | 285 ± 243.1 | 862.67 ± 159.85 |
| Clavulinaceae | 2 ± 1 | 6.67 ± 1.76 | 4 ± 1.73 | 230.67 ± 98.38 | 0.33 ± 0.33 | 2.67 ± 2.19 | 99.33 ± 51.56 | 0 ± 0 | 0 ± 0 | 0 ± 0 |
| Coniochaetaceae | 0 ± 0 | 24.33 ± 8.99 | 5.67 ± 3.71 | 1 ± 1 | 3.33 ± 1.86 | 29.33 ± 12.55 | 2 ± 1.53 | 3.33 ± 1.2 | 1.67 ± 1.67 | 4 ± 3.06 |
| Coniophoraceae | 0 ± 0 | 0 ± 0 | 0 ± 0 | 0 ± 0 | 0 ± 0 | 0 ± 0 | 0 ± 0 | 0 ± 0 | 0.33 ± 0.33 | 0 ± 0 |
| Conioscyphaceae | 0 ± 0 | 1 ± 1 | 0 ± 0 | 0 ± 0 | 0 ± 0 | 0 ± 0 | 0 ± 0 | 0 ± 0 | 1.33 ± 1.33 | 0 ± 0 |
| Coniothyriaceae | 2.67 ± 0.88 | 2.67 ± 1.67 | 43 ± 11.93 | 6.67 ± 2.96 | 1.33 ± 0.67 | 42.67 ± 13.78 | 35.67 ± 21.5 | 4.67 ± 2.4 | 5.67 ± 4.7 | 14 ± 5 |
| Conlariaceae | 0 ± 0 | 8.33 ± 2.73 | 1 ± 1 | 0.33 ± 0.33 | 0 ± 0 | 0 ± 0 | 1.33 ± 1.33 | 0 ± 0 | 0.33 ± 0.33 | 0 ± 0 |
| Cordycipitaceae | 53 ± 23.26 | 25 ± 10.02 | 118 ± 6.03 | 190 ± 89.76 | 48.67 ± 11.26 | 153.67 ± 23.21 | 317 ± 39.31 | 93.67 ± 43.78 | 61.33 ± 49.9 | 193.67 ± 32.98 |
| Corticiaceae | 14 ± 7.51 | 4.33 ± 1.67 | 34 ± 10.54 | 84.67 ± 26.67 | 471.33 ± 420.91 | 74 ± 21.28 | 141.67 ± 15.92 | 26 ± 12.66 | 26.33 ± 23.84 | 16.33 ± 10.4 |
| Cortinariaceae | 0 ± 0 | 0 ± 0 | 1.33 ± 0.88 | 0 ± 0 | 0 ± 0 | 1 ± 1 | 0.33 ± 0.33 | 0 ± 0 | 0.67 ± 0.67 | 10 ± 10 |
| Corynesporascaceae | 0.33 ± 0.33 | 0 ± 0 | 0 ± 0 | 0 ± 0 | 1.33 ± 1.33 | 0 ± 0 | 0 ± 0 | 0 ± 0 | 0 ± 0 | 0 ± 0 |
| Crepidotaceae | 0.67 ± 0.67 | 0 ± 0 | 0.33 ± 0.33 | 0 ± 0 | 0 ± 0 | 0.33 ± 0.33 | 0 ± 0 | 0.67 ± 0.67 | 4.33 ± 4.33 | 0 ± 0 |
| Cryptococcaceae | 3 ± 1.53 | 1.33 ± 0.88 | 4.67 ± 1.45 | 15.67 ± 11.67 | 70 ± 29.5 | 8 ± 0 | 7 ± 1.53 | 16.67 ± 7.84 | 15.33 ± 12.84 | 310.33 ± 65.79 |
| Cryptomycota_norank | 1.33 ± 0.88 | 0 ± 0 | 0 ± 0 | 0 ± 0 | 0 ± 0 | 0.33 ± 0.33 | 0 ± 0 | 0 ± 0 | 0 ± 0 | 0 ± 0 |
| Cucurbitariaceae | 9.67 ± 5.17 | 53.33 ± 19.97 | 32.33 ± 6.67 | 25.67 ± 18.75 | 52.33 ± 34.19 | 58.33 ± 9.24 | 68.67 ± 39.1 | 8.67 ± 6.23 | 17.67 ± 3.48 | 74.33 ± 10.48 |
| Cuniculitremaceae | 0 ± 0 | 0 ± 0 | 0 ± 0 | 0.33 ± 0.33 | 0 ± 0 | 0 ± 0 | 0 ± 0 | 1.33 ± 0.88 | 0 ± 0 | 0.67 ± 0.67 |
| Cunninghamellaceae | 0 ± 0 | 0 ± 0 | 0 ± 0 | 0 ± 0 | 0 ± 0 | 0 ± 0 | 0 ± 0 | 0 ± 0 | 0 ± 0 | 6 ± 6 |
| Cyphellophoraceae | 19.67 ± 9.24 | 1.33 ± 0.88 | 17.67 ± 10.68 | 3 ± 1.73 | 6.33 ± 1.86 | 31.67 ± 7.22 | 26.33 ± 10.73 | 164 ± 107.82 | 78.67 ± 72.69 | 13.67 ± 6.64 |
| Cystobasidiaceae | 0 ± 0 | 0 ± 0 | 0 ± 0 | 0 ± 0 | 0 ± 0 | 0.67 ± 0.33 | 0 ± 0 | 0 ± 0 | 0 ± 0 | 1 ± 1 |
| Cystobasidiomycetes_norank | 0.33 ± 0.33 | 0 ± 0 | 0.33 ± 0.33 | 0 ± 0 | 0 ± 0 | 0.33 ± 0.33 | 0.67 ± 0.67 | 1.33 ± 1.33 | 0 ± 0 | 0.33 ± 0.33 |
| Cystofilobasidiaceae | 0 ± 0 | 0 ± 0 | 39 ± 12.42 | 1 ± 0.58 | 2.67 ± 2.19 | 7 ± 5.51 | 37.67 ± 36.17 | 31.33 ± 19.81 | 5.67 ± 4.18 | 16.67 ± 8.35 |
| Cystostereaceae | 0 ± 0 | 0 ± 0 | 0 ± 0 | 0 ± 0 | 0 ± 0 | 0 ± 0 | 2.33 ± 2.33 | 0 ± 0 | 0 ± 0 | 0 ± 0 |
| Dacrymycetaceae | 0.67 ± 0.67 | 0 ± 0 | 0 ± 0 | 0 ± 0 | 0 ± 0 | 0 ± 0 | 0 ± 0 | 0 ± 0 | 0 ± 0 | 0 ± 0 |
| Debaryomycetaceae | 0 ± 0 | 0 ± 0 | 0 ± 0 | 0 ± 0 | 0 ± 0 | 0.33 ± 0.33 | 0 ± 0 | 1.33 ± 1.33 | 0.67 ± 0.67 | 0 ± 0 |
| Delonicicolaceae | 0 ± 0 | 0 ± 0 | 0 ± 0 | 0 ± 0 | 0 ± 0 | 0 ± 0 | 0 ± 0 | 8 ± 4.62 | 1.67 ± 1.67 | 0 ± 0 |
| Dermateaceae | 5 ± 2.65 | 3 ± 3 | 32.67 ± 10.2 | 7.33 ± 1.45 | 5.67 ± 1.2 | 44.67 ± 17.07 | 22.33 ± 5.78 | 30 ± 15.72 | 5.33 ± 1.45 | 15.33 ± 6.89 |
| Diaporthaceae | 1 ± 1 | 8 ± 7.02 | 11.33 ± 11.33 | 0.33 ± 0.33 | 0 ± 0 | 0.67 ± 0.67 | 3 ± 3 | 0 ± 0 | 0.67 ± 0.33 | 97 ± 95 |
| Diatrypaceae | 10 ± 5.13 | 0 ± 0 | 2.67 ± 1.76 | 0.67 ± 0.33 | 1.33 ± 0.88 | 8 ± 4.16 | 2.67 ± 1.2 | 0 ± 0 | 0 ± 0 | 1.67 ± 0.88 |
| Dictyosporiaceae | 0.67 ± 0.67 | 1 ± 1 | 2.67 ± 1.2 | 1.33 ± 0.67 | 3.33 ± 1.33 | 11.67 ± 1.2 | 6 ± 1.53 | 20.67 ± 13.3 | 12.33 ± 12.33 | 13.67 ± 7.22 |
| Didymellaceae | 132 ± 45 | 1823.33 ± 1127.46 | 774.67 ± 160.93 | 234.33 ± 156.33 | 289 ± 90.12 | 973.33 ± 518.65 | 623.67 ± 146.72 | 408 ± 227.21 | 264.67 ± 238.69 | 372.67 ± 18.66 |
| Didymosphaeriaceae | 1 ± 0.58 | 9 ± 4.93 | 5.33 ± 3.93 | 1.67 ± 1.2 | 0.67 ± 0.67 | 13 ± 0.58 | 8 ± 4.04 | 29.33 ± 15.07 | 21.33 ± 18.37 | 1.67 ± 0.67 |
| Dissoconiaceae | 0.67 ± 0.67 | 0 ± 0 | 0 ± 0 | 0.67 ± 0.67 | 0 ± 0 | 0 ± 0 | 1 ± 1 | 4.67 ± 0.33 | 4 ± 1.15 | 0 ± 0 |
| Diversisporaceae | 0 ± 0 | 0.67 ± 0.67 | 0 ± 0 | 0 ± 0 | 0 ± 0 | 0 ± 0 | 0.33 ± 0.33 | 0 ± 0 | 0 ± 0 | 0 ± 0 |
| Diversisporales_norank | 25 ± 6.03 | 170.33 ± 62.03 | 40.33 ± 14.77 | 219.33 ± 46.84 | 53 ± 19.63 | 56 ± 12.42 | 128.33 ± 55.71 | 117 ± 34.02 | 77 ± 2.65 | 172.33 ± 37.12 |
| Dothideomycetes_norank | 10.33 ± 2.91 | 675 ± 633.76 | 94 ± 71.49 | 37 ± 21.07 | 6.33 ± 1.45 | 13.33 ± 7.36 | 297 ± 274.5 | 83.33 ± 41.25 | 56 ± 54.51 | 46 ± 20.11 |
| Elaphomycetaceae | 0 ± 0 | 0 ± 0 | 0 ± 0 | 0.67 ± 0.67 | 0 ± 0 | 0 ± 0 | 0.67 ± 0.67 | 0 ± 0 | 0 ± 0 | 0 ± 0 |
| Elsinoaceae | 3 ± 2.08 | 0 ± 0 | 0 ± 0 | 0 ± 0 | 0.67 ± 0.67 | 2 ± 1.15 | 0 ± 0 | 0.33 ± 0.33 | 0 ± 0 | 3.67 ± 3.67 |
| Entolomataceae | 12 ± 11 | 181.67 ± 72.16 | 52 ± 24.83 | 47.33 ± 33.39 | 4.33 ± 2.4 | 14 ± 2.52 | 45.67 ± 14.72 | 29 ± 9.07 | 46 ± 4.93 | 253 ± 109.25 |
| Entorrhizaceae | 0 ± 0 | 54 ± 30.89 | 0 ± 0 | 2.67 ± 2.19 | 0 ± 0 | 0.33 ± 0.33 | 6.33 ± 6.33 | 0 ± 0 | 0 ± 0 | 0 ± 0 |
| Eocronartiaceae | 0.33 ± 0.33 | 0.67 ± 0.33 | 0 ± 0 | 5.67 ± 2.4 | 13 ± 3.61 | 0 ± 0 | 2 ± 2 | 89 ± 49.74 | 120 ± 119 | 0.67 ± 0.33 |
| Epibryaceae | 0 ± 0 | 0.33 ± 0.33 | 0 ± 0 | 0 ± 0 | 0 ± 0 | 0 ± 0 | 0.33 ± 0.33 | 0.67 ± 0.67 | 3.33 ± 2.85 | 0 ± 0 |
| Erysiphaceae | 9.67 ± 4.18 | 0.67 ± 0.33 | 24 ± 11.68 | 9.67 ± 6.89 | 0.33 ± 0.33 | 16.67 ± 5.81 | 13.67 ± 6.06 | 45.67 ± 25.41 | 9.33 ± 9.33 | 17.67 ± 4.67 |
| Erythrobasidiaceae | 0 ± 0 | 0 ± 0 | 3.67 ± 3.18 | 0 ± 0 | 0.33 ± 0.33 | 0.67 ± 0.67 | 2 ± 0.58 | 2.67 ± 1.76 | 0 ± 0 | 3.67 ± 2.03 |
| Eurotiomycetes_norank | 0 ± 0 | 0 ± 0 | 0 ± 0 | 0 ± 0 | 0 ± 0 | 0 ± 0 | 0 ± 0 | 0.67 ± 0.67 | 3 ± 0.58 | 0 ± 0 |
| Exidiaceae | 0 ± 0 | 2.67 ± 1.45 | 0 ± 0 | 6.67 ± 0.88 | 2 ± 1.15 | 2 ± 2 | 3.67 ± 2.33 | 0 ± 0 | 0.33 ± 0.33 | 5.33 ± 2.91 |
| Exobasidiaceae | 0 ± 0 | 0 ± 0 | 0 ± 0 | 0 ± 0 | 0 ± 0 | 0 ± 0 | 0 ± 0 | 0.67 ± 0.67 | 0 ± 0 | 0 ± 0 |
| Extremaceae | 0 ± 0 | 0 ± 0 | 0 ± 0 | 0 ± 0 | 0 ± 0 | 0 ± 0 | 1.33 ± 1.33 | 0 ± 0 | 0 ± 0 | 0 ± 0 |
| Filobasidiaceae | 0.67 ± 0.33 | 1 ± 1 | 0.67 ± 0.67 | 0.33 ± 0.33 | 0 ± 0 | 5.33 ± 2.67 | 0.67 ± 0.33 | 0.33 ± 0.33 | 1.33 ± 0.88 | 1.33 ± 1.33 |
| Geminibasidiaceae | 0.67 ± 0.67 | 4.33 ± 1.2 | 770.33 ± 181.55 | 3.67 ± 0.88 | 19.33 ± 5.84 | 270.67 ± 201.74 | 10 ± 4.16 | 0 ± 0 | 0 ± 0 | 7.33 ± 3.53 |
| Geoglossaceae | 42.33 ± 16.22 | 0.33 ± 0.33 | 12.33 ± 9.4 | 0.33 ± 0.33 | 12.33 ± 4.33 | 2.67 ± 2.19 | 31.67 ± 30.17 | 93.67 ± 92.17 | 408.33 ± 348.53 | 3.33 ± 1.33 |
| Gigasporaceae | 24.33 ± 7.45 | 4.33 ± 1.86 | 5.67 ± 5.17 | 21.67 ± 18.17 | 44.33 ± 8.17 | 14 ± 10.15 | 10 ± 3.51 | 54.33 ± 31.62 | 22.33 ± 19.84 | 18 ± 6.51 |
| Glomeraceae | 42.67 ± 12.71 | 356 ± 63.32 | 1.33 ± 0.33 | 70 ± 22.3 | 83 ± 22.3 | 9.33 ± 2.73 | 65.67 ± 27.23 | 56.33 ± 14.62 | 88.67 ± 34.91 | 229.67 ± 60.63 |
| Glomerellaceae | 0 ± 0 | 8.33 ± 0.88 | 12 ± 2 | 13 ± 3.79 | 0.33 ± 0.33 | 71.33 ± 28.62 | 12 ± 2.89 | 2.67 ± 2.19 | 6.33 ± 2.6 | 22 ± 11.53 |
| Gloniaceae | 0 ± 0 | 0 ± 0 | 0 ± 0 | 2.67 ± 1.76 | 0 ± 0 | 0 ± 0 | 1 ± 0.58 | 0.33 ± 0.33 | 1 ± 0.58 | 30 ± 22.59 |
| Gomphaceae | 0 ± 0 | 0 ± 0 | 0 ± 0 | 0 ± 0 | 0 ± 0 | 0 ± 0 | 0 ± 0 | 1.67 ± 1.2 | 4 ± 3.51 | 53 ± 53 |
| Graphiaceae | 0 ± 0 | 0.33 ± 0.33 | 0.33 ± 0.33 | 0 ± 0 | 0 ± 0 | 6 ± 2.65 | 0 ± 0 | 0 ± 0 | 0 ± 0 | 0.33 ± 0.33 |
| Gymnoascaceae | 0 ± 0 | 0 ± 0 | 0 ± 0 | 0 ± 0 | 0.67 ± 0.67 | 0 ± 0 | 0 ± 0 | 0 ± 0 | 0 ± 0 | 0 ± 0 |
| Halosphaeriaceae | 0 ± 0 | 0 ± 0 | 0 ± 0 | 0 ± 0 | 0 ± 0 | 0 ± 0 | 0 ± 0 | 0 ± 0 | 0 ± 0 | 1.33 ± 1.33 |
| Helminthosphaeriaceae | 0.33 ± 0.33 | 24.33 ± 18.34 | 0 ± 0 | 1 ± 1 | 0 ± 0 | 3 ± 1.53 | 2 ± 1.53 | 0 ± 0 | 0 ± 0 | 0 ± 0 |
| Helotiaceae | 3 ± 2 | 82 ± 40.51 | 42.67 ± 11.17 | 20.67 ± 12.02 | 2.33 ± 1.45 | 107 ± 68.5 | 46.67 ± 10.4 | 51 ± 26.35 | 20.33 ± 12.91 | 101 ± 51.79 |
| Helotiales_norank | 23.33 ± 10.48 | 66.33 ± 23.31 | 553.33 ± 89.14 | 24.67 ± 10.91 | 12 ± 4.04 | 165 ± 130.85 | 78.33 ± 32.27 | 377.33 ± 179.6 | 320.33 ± 276.96 | 544.67 ± 252.15 |
| Herpotrichiellaceae | 123.67 ± 20.2 | 954 ± 382.57 | 1022.33 ± 40.32 | 291.67 ± 78.32 | 134.67 ± 50.99 | 429.67 ± 181.67 | 604.67 ± 144.81 | 1822 ± 917 | 1189 ± 896.56 | 370.33 ± 133.32 |
| Hoehnelomycetaceae | 1 ± 1 | 281.67 ± 149.45 | 0 ± 0 | 19.67 ± 5.84 | 0 ± 0 | 0.67 ± 0.67 | 53.67 ± 38.37 | 2.33 ± 2.33 | 0 ± 0 | 0.33 ± 0.33 |
| Hyaloscyphaceae | 7.67 ± 2.33 | 95.67 ± 40.35 | 205.67 ± 59.96 | 53.67 ± 31.18 | 6.33 ± 2.85 | 212.33 ± 15.96 | 73 ± 33.72 | 200 ± 179.06 | 23 ± 5.2 | 194 ± 54.56 |
| Hydnodontaceae | 2 ± 1 | 29.67 ± 10.17 | 12.33 ± 5.24 | 174.67 ± 136.56 | 1.67 ± 0.88 | 8 ± 4.73 | 97.67 ± 59.61 | 33.67 ± 9.28 | 19.33 ± 2.19 | 2814 ± 2726.27 |
| Hygrophoraceae | 265.67 ± 19.24 | 2 ± 1 | 5 ± 1 | 0.33 ± 0.33 | 16 ± 0.58 | 5.33 ± 2.67 | 4.33 ± 2.4 | 0.33 ± 0.33 | 0 ± 0 | 2.67 ± 2.67 |
| Hymenochaetales_norank | 0 ± 0 | 8.67 ± 4.91 | 0.33 ± 0.33 | 0.33 ± 0.33 | 0 ± 0 | 0.33 ± 0.33 | 1 ± 0.58 | 8 ± 7.51 | 0.67 ± 0.67 | 0.67 ± 0.33 |
| Hymenogastraceae | 0 ± 0 | 0 ± 0 | 1 ± 0.58 | 4.33 ± 3.38 | 0 ± 0 | 11 ± 4.58 | 22 ± 14.01 | 6.33 ± 2.19 | 1.67 ± 0.67 | 2 ± 2 |
| Hypocreaceae | 16 ± 2.65 | 371.67 ± 132.52 | 144.67 ± 48.95 | 65.33 ± 27.91 | 17.33 ± 13.38 | 115.67 ± 31.07 | 154 ± 63.79 | 77.33 ± 27.18 | 109.67 ± 64.76 | 113 ± 8.39 |
| Hypocreales_norank | 12.33 ± 5.84 | 17 ± 6.03 | 41.33 ± 15.92 | 9.33 ± 0.33 | 14 ± 3.21 | 128 ± 57.24 | 89 ± 64 | 96.67 ± 37.25 | 114 ± 86.02 | 34 ± 4.73 |
| Hypoxylaceae | 0 ± 0 | 0.33 ± 0.33 | 0.33 ± 0.33 | 1.33 ± 1.33 | 1 ± 1 | 0 ± 0 | 0.67 ± 0.67 | 1 ± 1 | 0.33 ± 0.33 | 1.67 ± 1.67 |
| Inocybaceae | 2.33 ± 2.33 | 0.33 ± 0.33 | 57.67 ± 22.64 | 0 ± 0 | 0 ± 0 | 9 ± 1.73 | 2 ± 1 | 0.67 ± 0.67 | 0.33 ± 0.33 | 12.33 ± 9.4 |
| Irpicaceae | 0 ± 0 | 12.67 ± 2.91 | 0 ± 0 | 0 ± 0 | 0 ± 0 | 0 ± 0 | 1.33 ± 1.33 | 0 ± 0 | 0 ± 0 | 0 ± 0 |
| Ischnodermataceae | 0 ± 0 | 0 ± 0 | 2.67 ± 2.19 | 0 ± 0 | 0 ± 0 | 0 ± 0 | 0 ± 0 | 0 ± 0 | 0 ± 0 | 0 ± 0 |
| Kickxellaceae | 0 ± 0 | 0 ± 0 | 2.33 ± 2.33 | 0 ± 0 | 0 ± 0 | 0 ± 0 | 0.33 ± 0.33 | 0 ± 0 | 0 ± 0 | 0 ± 0 |
| Kondoaceae | 0 ± 0 | 0 ± 0 | 0.33 ± 0.33 | 0 ± 0 | 0 ± 0 | 0 ± 0 | 0 ± 0 | 0.33 ± 0.33 | 0 ± 0 | 4 ± 0.58 |
| Lachnocladiaceae | 4 ± 1 | 0 ± 0 | 0 ± 0 | 0 ± 0 | 0.33 ± 0.33 | 0 ± 0 | 0 ± 0 | 0 ± 0 | 0 ± 0 | 0 ± 0 |
| Lasiosphaeriaceae | 31 ± 12.49 | 521.33 ± 300.37 | 8351.33 ± 716.57 | 212.33 ± 60.29 | 133 ± 20.79 | 2329 ± 1520.89 | 582 ± 13.08 | 292.67 ± 110.07 | 83 ± 15.53 | 1217.33 ± 394.26 |
| Lecanoromycetes_norank | 0.67 ± 0.33 | 4 ± 2.08 | 7 ± 3.46 | 1 ± 0.58 | 0 ± 0 | 4.67 ± 4.67 | 3.67 ± 0.88 | 64 ± 59.53 | 6.33 ± 5.36 | 1 ± 1 |
| Lentitheciaceae | 0.33 ± 0.33 | 48.33 ± 16.76 | 0 ± 0 | 2 ± 0.58 | 0 ± 0 | 2 ± 1.53 | 12 ± 7.94 | 90 ± 84.56 | 4 ± 1.73 | 4.33 ± 3.84 |
| Leotiaceae | 2.33 ± 1.45 | 0.33 ± 0.33 | 33.33 ± 9.96 | 1 ± 0.58 | 3.67 ± 0.67 | 4.33 ± 3.38 | 0.67 ± 0.33 | 14 ± 11.02 | 7 ± 5.57 | 0 ± 0 |
| Leotiomycetes_norank | 0 ± 0 | 26.67 ± 11.35 | 20 ± 14.05 | 26 ± 8.33 | 3 ± 1.73 | 32.67 ± 8.99 | 13.33 ± 7.22 | 4.33 ± 2.4 | 2.33 ± 1.45 | 15.67 ± 4.06 |
| Leptosphaeriaceae | 0 ± 0 | 6.33 ± 2.6 | 5.67 ± 5.17 | 8 ± 4.16 | 0.67 ± 0.33 | 11.67 ± 7.06 | 4.33 ± 2.85 | 1 ± 1 | 0 ± 0 | 0.33 ± 0.33 |
| Leucosporidiaceae | 0 ± 0 | 11.33 ± 8.51 | 11.33 ± 11.33 | 1 ± 1 | 0 ± 0 | 4.67 ± 4.67 | 1 ± 1 | 1 ± 1 | 0.33 ± 0.33 | 0 ± 0 |
| Lipomycetaceae | 0 ± 0 | 0 ± 0 | 0.33 ± 0.33 | 2 ± 1.15 | 0 ± 0 | 0.33 ± 0.33 | 2 ± 1.15 | 5 ± 2.89 | 5.33 ± 3.93 | 4.67 ± 3.28 |
| Lophiostomataceae | 2.33 ± 1.86 | 8.33 ± 3.48 | 21 ± 8.39 | 6 ± 2.31 | 4 ± 2 | 9 ± 4.73 | 30.67 ± 17.23 | 11.67 ± 5.36 | 4 ± 1.73 | 4.67 ± 0.88 |
| Lophiotremataceae | 0.67 ± 0.67 | 0 ± 0 | 0 ± 0 | 0 ± 0 | 0 ± 0 | 130 ± 118.63 | 1.67 ± 1.67 | 12.33 ± 5.67 | 3 ± 1.73 | 2 ± 2 |
| Lulworthiaceae | 7.67 ± 5.17 | 0 ± 0 | 3 ± 3 | 0 ± 0 | 15.33 ± 8.45 | 3.67 ± 3.18 | 0.67 ± 0.67 | 1 ± 1 | 0 ± 0 | 0 ± 0 |
| Lycoperdaceae | 0 ± 0 | 0 ± 0 | 0 ± 0 | 0.67 ± 0.67 | 0 ± 0 | 0 ± 0 | 0 ± 0 | 0 ± 0 | 0 ± 0 | 0 ± 0 |
| Lyophyllaceae | 0.33 ± 0.33 | 2.67 ± 1.45 | 1.67 ± 0.33 | 0.67 ± 0.67 | 0.33 ± 0.33 | 0.67 ± 0.67 | 0 ± 0 | 0 ± 0 | 1.67 ± 0.88 | 2 ± 0.58 |
| Magnaporthaceae | 0 ± 0 | 1 ± 1 | 0.33 ± 0.33 | 0 ± 0 | 0 ± 0 | 0 ± 0 | 0.67 ± 0.33 | 2.67 ± 2.67 | 0 ± 0 | 0.33 ± 0.33 |
| Malasseziaceae | 0 ± 0 | 0 ± 0 | 2.33 ± 1.45 | 0.67 ± 0.67 | 0 ± 0 | 0.67 ± 0.67 | 2.67 ± 2.19 | 0 ± 0 | 1.33 ± 0.88 | 2.67 ± 1.33 |
| Marasmiaceae | 0 ± 0 | 1.33 ± 1.33 | 181.67 ± 28.5 | 1.33 ± 0.88 | 0.33 ± 0.33 | 44.33 ± 34.82 | 3 ± 1.73 | 0 ± 0 | 0 ± 0 | 0.67 ± 0.33 |
| Massarinaceae | 11 ± 6.56 | 1.33 ± 1.33 | 11.67 ± 6.89 | 5.67 ± 1.2 | 32 ± 4.04 | 24.33 ± 1.2 | 44.67 ± 31.39 | 16.67 ± 10.2 | 11 ± 1.53 | 19.67 ± 4.91 |
| Melanconiellaceae | 11.33 ± 7.36 | 50.67 ± 35.47 | 1 ± 0.58 | 4.67 ± 3.67 | 5.33 ± 2.85 | 2.67 ± 1.67 | 26 ± 13.8 | 9 ± 4.51 | 5 ± 3.61 | 13.33 ± 8.35 |
| Melanommataceae | 1.67 ± 0.88 | 3 ± 1.53 | 10 ± 5.86 | 5.33 ± 1.86 | 5.67 ± 2.03 | 12 ± 8.02 | 17 ± 9 | 13.33 ± 5.36 | 24 ± 11.37 | 61.67 ± 15.24 |
| Meripilaceae | 0.33 ± 0.33 | 0 ± 0 | 0 ± 0 | 0 ± 0 | 4.33 ± 1.86 | 0 ± 0 | 0 ± 0 | 24.67 ± 24.67 | 1.67 ± 1.67 | 0 ± 0 |
| Meruliaceae | 0 ± 0 | 0 ± 0 | 0.33 ± 0.33 | 0 ± 0 | 0 ± 0 | 0 ± 0 | 0 ± 0 | 0 ± 0 | 0 ± 0 | 0 ± 0 |
| Microascaceae | 1.33 ± 0.88 | 4.33 ± 2.03 | 45.67 ± 15.21 | 3.33 ± 1.45 | 0.67 ± 0.33 | 62 ± 30.51 | 113.33 ± 100.36 | 8.33 ± 3.53 | 4.33 ± 1.86 | 10 ± 7 |
| Microascales_norank | 0 ± 0 | 0.67 ± 0.67 | 0 ± 0 | 1.67 ± 1.2 | 4.33 ± 2.33 | 8.67 ± 6.33 | 10 ± 4.16 | 0 ± 0 | 0 ± 0 | 0.33 ± 0.33 |
| Microbotryaceae | 0 ± 0 | 0 ± 0 | 0 ± 0 | 0 ± 0 | 0 ± 0 | 0 ± 0 | 0.33 ± 0.33 | 0 ± 0 | 0 ± 0 | 0 ± 0 |
| Microbotryomycetes_norank | 0 ± 0 | 0 ± 0 | 0 ± 0 | 0 ± 0 | 0 ± 0 | 0.33 ± 0.33 | 0.33 ± 0.33 | 1.33 ± 1.33 | 0 ± 0 | 1.67 ± 1.2 |
| Microdochiaceae | 5 ± 2.08 | 22.67 ± 14.85 | 60 ± 24.98 | 4 ± 1.53 | 31.33 ± 8.41 | 21.33 ± 5.36 | 35.67 ± 24.18 | 8 ± 4.36 | 19 ± 19 | 6.33 ± 4.37 |
| Micropeltidaceae | 0 ± 0 | 0 ± 0 | 0 ± 0 | 0 ± 0 | 0 ± 0 | 2 ± 2 | 0 ± 0 | 0 ± 0 | 0 ± 0 | 0 ± 0 |
| Minutisphaeraceae | 0 ± 0 | 0 ± 0 | 0 ± 0 | 0 ± 0 | 0 ± 0 | 0 ± 0 | 0 ± 0 | 0 ± 0 | 0 ± 0 | 0 ± 0 |
| Monoblastiaceae | 0 ± 0 | 0 ± 0 | 0 ± 0 | 0 ± 0 | 0 ± 0 | 5 ± 4.51 | 0.33 ± 0.33 | 0 ± 0 | 0 ± 0 | 0 ± 0 |
| Morosphaeriaceae | 3.33 ± 1.33 | 2 ± 1.53 | 3.33 ± 2.4 | 2 ± 2 | 9 ± 4.04 | 12.67 ± 4.91 | 2.67 ± 2.19 | 13.67 ± 6.12 | 11.33 ± 9.33 | 6.67 ± 1.76 |
| Mortierellaceae | 193.67 ± 67.35 | 1320.67 ± 111.76 | 2725.67 ± 397.85 | 1745 ± 935.94 | 517.67 ± 116.12 | 5523 ± 1415.37 | 3386.67 ± 1034.61 | 1718.33 ± 754.42 | 1544 ± 1175.52 | 2783 ± 423.62 |
| Mortierellales_norank | 0 ± 0 | 0 ± 0 | 0 ± 0 | 1 ± 1 | 0 ± 0 | 0 ± 0 | 0.67 ± 0.67 | 0 ± 0 | 0 ± 0 | 0 ± 0 |
| Mrakiaceae | 1.33 ± 0.67 | 1.67 ± 1.2 | 119.67 ± 64.6 | 1.67 ± 1.2 | 2.33 ± 0.88 | 47.67 ± 9.26 | 89 ± 75 | 32.33 ± 24.85 | 7 ± 4.58 | 11 ± 4.36 |
| Mucoraceae | 0 ± 0 | 0 ± 0 | 2 ± 1.15 | 0 ± 0 | 0 ± 0 | 0 ± 0 | 0 ± 0 | 0 ± 0 | 0 ± 0 | 0 ± 0 |
| Mycenaceae | 0.67 ± 0.33 | 13 ± 10.15 | 2.67 ± 2.67 | 4 ± 2.08 | 0 ± 0 | 0 ± 0 | 1.33 ± 0.67 | 0 ± 0 | 0.67 ± 0.67 | 14.67 ± 13.68 |
| Mycosphaerellaceae | 7.67 ± 2.4 | 1.67 ± 0.88 | 232 ± 33.81 | 26 ± 12.34 | 25 ± 5.77 | 117.67 ± 65.79 | 131.33 ± 34.94 | 284 ± 204.82 | 59.67 ± 54.17 | 72.67 ± 24.55 |
| Myriangiaceae | 0 ± 0 | 0 ± 0 | 0 ± 0 | 0 ± 0 | 0 ± 0 | 0 ± 0 | 0 ± 0 | 0 ± 0 | 0 ± 0 | 0.33 ± 0.33 |
| Myriangiales_norank | 0 ± 0 | 0 ± 0 | 0 ± 0 | 0.33 ± 0.33 | 0 ± 0 | 0 ± 0 | 0 ± 0 | 0 ± 0 | 0.67 ± 0.67 | 3.67 ± 2.33 |
| Myrmecridiaceae | 16.67 ± 4.81 | 167 ± 159.5 | 1.67 ± 0.33 | 11.33 ± 11.33 | 2.33 ± 1.45 | 3.33 ± 1.45 | 17 ± 12 | 9.67 ± 5.93 | 2 ± 1.53 | 5.67 ± 0.33 |
| Myxotrichaceae | 0 ± 0 | 0.33 ± 0.33 | 2.67 ± 2.67 | 7 ± 3 | 0 ± 0 | 5.67 ± 1.76 | 6.33 ± 2.96 | 25.33 ± 8.67 | 11.33 ± 6.57 | 13.67 ± 3.28 |
| Nectriaceae | 848.33 ± 171.05 | 7560.67 ± 1709.87 | 2753.33 ± 190.41 | 1159.67 ± 292.94 | 1302 ± 386.04 | 6997.33 ± 1977.97 | 4752.33 ± 1175.31 | 2718.67 ± 1180.68 | 1066.67 ± 642.12 | 3758 ± 655.37 |
| Neodevriesiaceae | 0 ± 0 | 0 ± 0 | 1.67 ± 1.67 | 1 ± 1 | 0 ± 0 | 1 ± 0.58 | 1.67 ± 0.33 | 9 ± 4.73 | 5.33 ± 5.33 | 0.33 ± 0.33 |
| Neohendersoniaceae | 0 ± 0 | 0 ± 0 | 0 ± 0 | 0 ± 0 | 0 ± 0 | 0 ± 0 | 2 ± 2 | 0.33 ± 0.33 | 0 ± 0 | 0 ± 0 |
| Neopyrenochaetaceae | 2.33 ± 2.33 | 4 ± 3.06 | 0 ± 0 | 0 ± 0 | 8.33 ± 1.76 | 22.67 ± 8.33 | 4.33 ± 1.33 | 64.33 ± 38.2 | 20 ± 20 | 1 ± 0 |
| Niessliaceae | 0 ± 0 | 0 ± 0 | 3.33 ± 2.85 | 28 ± 12.77 | 0 ± 0 | 13.67 ± 8.69 | 14.67 ± 9.28 | 13 ± 6.43 | 4.67 ± 4.18 | 0.67 ± 0.67 |
| Nigrogranaceae | 0 ± 0 | 0 ± 0 | 3.33 ± 3.33 | 0.33 ± 0.33 | 0.33 ± 0.33 | 15.67 ± 6.36 | 11.33 ± 10.35 | 3 ± 3 | 1 ± 1 | 1 ± 0.58 |
| Olpidiaceae | 37 ± 8.5 | 75.67 ± 10.17 | 3.33 ± 2.4 | 12.33 ± 2.85 | 19.33 ± 3.84 | 255.33 ± 126.65 | 45 ± 11 | 2 ± 1.15 | 2.67 ± 1.76 | 13.67 ± 11.26 |
| Omphalotaceae | 0 ± 0 | 0 ± 0 | 0 ± 0 | 0 ± 0 | 0 ± 0 | 0 ± 0 | 0 ± 0 | 0 ± 0 | 0.33 ± 0.33 | 20 ± 8.14 |
| Onygenaceae | 1 ± 0.58 | 0 ± 0 | 0 ± 0 | 0.33 ± 0.33 | 2.33 ± 0.88 | 0.33 ± 0.33 | 6 ± 5.03 | 1.67 ± 0.88 | 4.33 ± 3.84 | 20.33 ± 6.01 |
| Onygenales_norank | 0 ± 0 | 0 ± 0 | 0 ± 0 | 0.67 ± 0.67 | 0.67 ± 0.33 | 1.67 ± 0.33 | 6.33 ± 4.91 | 0 ± 0 | 0.33 ± 0.33 | 3 ± 2.52 |
| Ophiocordycipitaceae | 27 ± 6.43 | 302.67 ± 80.37 | 139.33 ± 27 | 117 ± 50.54 | 76.67 ± 20.2 | 211 ± 59.43 | 390.33 ± 104.99 | 1347 ± 931.69 | 785.67 ± 685.23 | 224 ± 59.14 |
| Ophiostomataceae | 0 ± 0 | 0 ± 0 | 1 ± 1 | 0 ± 0 | 0 ± 0 | 8.33 ± 5.61 | 5.33 ± 4.84 | 1 ± 0.58 | 2 ± 2 | 7.67 ± 2.96 |
| Orbiliaceae | 7.67 ± 4.18 | 106 ± 26.76 | 79.67 ± 7.51 | 11.33 ± 3.38 | 8 ± 3.51 | 98.67 ± 20.63 | 46.67 ± 4.7 | 296.33 ± 144.47 | 114 ± 90.18 | 46.33 ± 15.6 |
| Orbiliales_norank | 14.67 ± 9.21 | 8.33 ± 4.26 | 6.33 ± 3.18 | 4.33 ± 4.33 | 5 ± 3.51 | 12 ± 4.58 | 20 ± 10.82 | 106.67 ± 45.08 | 67.67 ± 50.23 | 30.33 ± 13.69 |
| Pannariaceae | 1 ± 1 | 2.33 ± 2.33 | 6 ± 2.65 | 3 ± 0.58 | 0 ± 0 | 2 ± 1.15 | 27 ± 25.03 | 0.33 ± 0.33 | 0 ± 0 | 0.33 ± 0.33 |
| Papulosaceae | 0 ± 0 | 0 ± 0 | 0 ± 0 | 0 ± 0 | 0 ± 0 | 0 ± 0 | 0 ± 0 | 1.33 ± 1.33 | 0 ± 0 | 0 ± 0 |
| Paraglomeraceae | 0 ± 0 | 27.67 ± 13.35 | 0 ± 0 | 19.67 ± 1.45 | 0.33 ± 0.33 | 0.33 ± 0.33 | 7.67 ± 4.81 | 5.67 ± 3.84 | 7.67 ± 1.76 | 27.33 ± 7.88 |
| Peniophoraceae | 0 ± 0 | 0 ± 0 | 0.33 ± 0.33 | 2 ± 2 | 0 ± 0 | 7.33 ± 5.04 | 1.33 ± 0.67 | 1.33 ± 1.33 | 0 ± 0 | 5.67 ± 3.48 |
| Periconiaceae | 6.67 ± 0.88 | 8.67 ± 3.38 | 14.67 ± 6.01 | 2 ± 0.58 | 26 ± 12.22 | 16.33 ± 4.91 | 7.67 ± 2.67 | 80 ± 39.02 | 34 ± 19.5 | 7.33 ± 2.67 |
| Pervetustaceae | 0.33 ± 0.33 | 0 ± 0 | 0.67 ± 0.67 | 0 ± 0 | 2 ± 0.58 | 0.67 ± 0.67 | 0.33 ± 0.33 | 22.67 ± 11.57 | 16 ± 16 | 0 ± 0 |
| Pezizaceae | 6.33 ± 6.33 | 0 ± 0 | 2.67 ± 2.19 | 0 ± 0 | 1 ± 0.58 | 1.33 ± 1.33 | 0 ± 0 | 2.33 ± 1.2 | 0 ± 0 | 0.33 ± 0.33 |
| Pezizales_norank | 0.33 ± 0.33 | 0 ± 0 | 3.67 ± 3.18 | 0.33 ± 0.33 | 0.33 ± 0.33 | 2.67 ± 1.76 | 0.33 ± 0.33 | 0 ± 0 | 0 ± 0 | 1.67 ± 1.67 |
| Pezizellaceae | 0 ± 0 | 0 ± 0 | 0 ± 0 | 0 ± 0 | 0 ± 0 | 0 ± 0 | 0 ± 0 | 0 ± 0 | 0.67 ± 0.67 | 0 ± 0 |
| Phaeococcomycetaceae | 1.67 ± 1.67 | 0 ± 0 | 0 ± 0 | 0 ± 0 | 0 ± 0 | 0 ± 0 | 0 ± 0 | 0 ± 0 | 0 ± 0 | 0 ± 0 |
| Phaeomoniellaceae | 1 ± 0.58 | 3 ± 2.52 | 1.67 ± 0.33 | 0 ± 0 | 0 ± 0 | 33.33 ± 20.85 | 4.67 ± 2.91 | 6.67 ± 3.76 | 2.67 ± 1.2 | 1.67 ± 0.88 |
| Phaeosphaeriaceae | 15.33 ± 3.48 | 114.67 ± 50.32 | 15.67 ± 1.33 | 8.67 ± 4.63 | 15.33 ± 7.69 | 35 ± 4.04 | 52 ± 23.71 | 57.33 ± 34.95 | 29.67 ± 23.67 | 47 ± 11.02 |
| Phaeotremellaceae | 0 ± 0 | 0 ± 0 | 0 ± 0 | 0 ± 0 | 5.67 ± 5.67 | 0.33 ± 0.33 | 0 ± 0 | 0 ± 0 | 0.67 ± 0.67 | 0.67 ± 0.67 |
| Phanerochaetaceae | 0 ± 0 | 0 ± 0 | 0.67 ± 0.67 | 0.33 ± 0.33 | 0 ± 0 | 0 ± 0 | 0 ± 0 | 0 ± 0 | 0 ± 0 | 0 ± 0 |
| Phomatosporaceae | 0.67 ± 0.67 | 0 ± 0 | 13.67 ± 2.33 | 1.33 ± 0.33 | 0.67 ± 0.67 | 14.67 ± 3.33 | 3 ± 2.08 | 3.67 ± 1.76 | 0.33 ± 0.33 | 13 ± 11.53 |
| Physciaceae | 0 ± 0 | 0 ± 0 | 0 ± 0 | 0 ± 0 | 0 ± 0 | 0 ± 0 | 0 ± 0 | 0 ± 0 | 0 ± 0 | 2 ± 2 |
| Physodermataceae | 0 ± 0 | 0 ± 0 | 105.67 ± 25.77 | 1.33 ± 0.88 | 2.33 ± 1.2 | 33.33 ± 27.95 | 0.33 ± 0.33 | 0.33 ± 0.33 | 0 ± 0 | 2.33 ± 2.33 |
| Piptocephalidaceae | 0.67 ± 0.67 | 0 ± 0 | 0 ± 0 | 0 ± 0 | 0.33 ± 0.33 | 0.33 ± 0.33 | 0.67 ± 0.67 | 0 ± 0 | 0.33 ± 0.33 | 0 ± 0 |
| Piskurozymaceae | 1.33 ± 0.88 | 186.33 ± 57.55 | 487.33 ± 309.99 | 49.67 ± 28.94 | 2 ± 0.58 | 113.33 ± 101.83 | 37.67 ± 16.13 | 103.33 ± 60.41 | 92 ± 86 | 173 ± 88.19 |
| Pisorisporiaceae | 0 ± 0 | 0 ± 0 | 0.67 ± 0.67 | 0 ± 0 | 0 ± 0 | 4 ± 2.31 | 4 ± 4 | 4.67 ± 4.67 | 1.67 ± 1.2 | 0 ± 0 |
| Plectosphaerellaceae | 26.67 ± 5.21 | 157.33 ± 66.14 | 567 ± 75.03 | 161.33 ± 25.72 | 205.33 ± 37.36 | 1630.33 ± 424.43 | 463.67 ± 59.04 | 138.33 ± 70.01 | 76 ± 69.51 | 1342.33 ± 1089.54 |
| Pleosporaceae | 0.67 ± 0.67 | 9 ± 4.93 | 22 ± 12.58 | 1.33 ± 0.88 | 1.33 ± 0.33 | 22 ± 8.19 | 7.33 ± 1.45 | 107 ± 57.5 | 76.67 ± 73.67 | 8.33 ± 5.84 |
| Pleosporales_norank | 7.33 ± 2.33 | 5.67 ± 5.17 | 96 ± 3.61 | 1 ± 0.58 | 11 ± 2.08 | 136.33 ± 105.57 | 20 ± 5.51 | 6.33 ± 1.33 | 2 ± 1.15 | 47 ± 12.49 |
| Pleurotheciaceae | 0 ± 0 | 0 ± 0 | 0.67 ± 0.33 | 0 ± 0 | 0.33 ± 0.33 | 1.33 ± 1.33 | 2.33 ± 1.45 | 0 ± 0 | 0.67 ± 0.67 | 1.33 ± 1.33 |
| Ploettnerulaceae | 28.33 ± 20.63 | 26.67 ± 3.18 | 5.67 ± 1.67 | 2.33 ± 0.88 | 7.67 ± 2.6 | 15.33 ± 6.74 | 19 ± 1 | 121 ± 82.24 | 57.67 ± 57.17 | 7 ± 2 |
| Pluteaceae | 0.33 ± 0.33 | 0 ± 0 | 15.67 ± 13.72 | 0.67 ± 0.67 | 0.33 ± 0.33 | 7.33 ± 6.84 | 2 ± 1.53 | 0.33 ± 0.33 | 1 ± 0.58 | 0 ± 0 |
| Polyporaceae | 39.67 ± 6.17 | 22.33 ± 0.33 | 27.33 ± 9.61 | 12.67 ± 6.17 | 17 ± 3.21 | 20 ± 6.93 | 41 ± 19.97 | 35 ± 15.62 | 14.33 ± 11.39 | 38.67 ± 14.05 |
| Polyporales_norank | 0 ± 0 | 0 ± 0 | 0 ± 0 | 1 ± 0.58 | 0.33 ± 0.33 | 0 ± 0 | 1.67 ± 1.2 | 0 ± 0 | 0 ± 0 | 1.67 ± 1.67 |
| Porodiplodiaceae | 23 ± 5.51 | 0 ± 0 | 20 ± 4.93 | 0.67 ± 0.33 | 7 ± 4.04 | 8.33 ± 4.33 | 34.67 ± 28.67 | 2.33 ± 1.2 | 0.67 ± 0.67 | 3 ± 0.58 |
| Psathyrellaceae | 10.33 ± 5.21 | 2.33 ± 1.45 | 24.67 ± 3.18 | 1 ± 0.58 | 5 ± 0.58 | 18 ± 7.77 | 19.33 ± 9.84 | 4 ± 3.51 | 7 ± 3.79 | 7 ± 4.16 |
| Pseudeurotiaceae | 1.33 ± 0.33 | 43.33 ± 22.15 | 7.67 ± 3.84 | 151.67 ± 35.32 | 1.33 ± 0.88 | 27.33 ± 5.17 | 95.67 ± 52.99 | 0.33 ± 0.33 | 3 ± 2.52 | 6.67 ± 2.91 |
| Pseudoperisporiaceae | 0 ± 0 | 0 ± 0 | 0 ± 0 | 0 ± 0 | 0 ± 0 | 0 ± 0 | 0 ± 0 | 0 ± 0 | 1 ± 1 | 0 ± 0 |
| Pterulaceae | 0 ± 0 | 0 ± 0 | 0 ± 0 | 0 ± 0 | 0 ± 0 | 0 ± 0 | 1 ± 1 | 1 ± 1 | 0 ± 0 | 0.33 ± 0.33 |
| Pyrenochaetopsidaceae | 83 ± 41.58 | 248 ± 105.84 | 16.67 ± 11.22 | 34.67 ± 12.33 | 148.67 ± 108.79 | 95.67 ± 12.35 | 312.33 ± 180.51 | 129.33 ± 63.26 | 75 ± 56.24 | 33 ± 4.36 |
| Pyronemataceae | 50.67 ± 20.21 | 17 ± 3 | 36 ± 17.5 | 270.33 ± 51.13 | 14.67 ± 4.67 | 30 ± 7.51 | 116.33 ± 19.68 | 498.67 ± 414.2 | 575.33 ± 306.77 | 33.33 ± 20.54 |
| Pyxidiophoraceae | 1.33 ± 1.33 | 0 ± 0 | 2.33 ± 1.2 | 0.67 ± 0.67 | 0.67 ± 0.67 | 41 ± 22.14 | 0.33 ± 0.33 | 1.33 ± 1.33 | 1.33 ± 1.33 | 1.67 ± 0.88 |
| Ramalinaceae | 0.67 ± 0.67 | 0 ± 0 | 0.67 ± 0.67 | 3.33 ± 3.33 | 0.33 ± 0.33 | 0 ± 0 | 0.67 ± 0.67 | 0.67 ± 0.67 | 8 ± 6.11 | 0.33 ± 0.33 |
| Ramicandelaberaceae | 0 ± 0 | 0 ± 0 | 0 ± 0 | 0 ± 0 | 0 ± 0 | 0 ± 0 | 0 ± 0 | 0 ± 0 | 3.67 ± 3.67 | 0 ± 0 |
| Rhizocarpaceae | 0 ± 0 | 0.33 ± 0.33 | 0 ± 0 | 0 ± 0 | 0 ± 0 | 0 ± 0 | 0 ± 0 | 0 ± 0 | 0 ± 0 | 0 ± 0 |
| Rhizophlyctidaceae | 4.33 ± 4.33 | 0 ± 0 | 1.33 ± 0.88 | 0.33 ± 0.33 | 0.33 ± 0.33 | 0.33 ± 0.33 | 0 ± 0 | 0 ± 0 | 29 ± 29 | 5.33 ± 3.18 |
| Rhizophydiaceae | 0 ± 0 | 0 ± 0 | 0 ± 0 | 0 ± 0 | 0 ± 0 | 0 ± 0 | 0 ± 0 | 2 ± 1 | 1.67 ± 1.67 | 0 ± 0 |
| Rhizophydiales_norank | 0 ± 0 | 0 ± 0 | 0.67 ± 0.67 | 6.33 ± 6.33 | 0 ± 0 | 0.33 ± 0.33 | 4.33 ± 3.33 | 0.67 ± 0.67 | 0 ± 0 | 0 ± 0 |
| Rhynchogastremataceae | 39.67 ± 25.98 | 9.67 ± 4.81 | 27.67 ± 20.27 | 5 ± 3.51 | 32 ± 1 | 11.67 ± 3.53 | 11 ± 1.73 | 140 ± 108.68 | 32.67 ± 29.67 | 21 ± 6.24 |
| Russulaceae | 0 ± 0 | 0 ± 0 | 5.67 ± 1.86 | 1 ± 1 | 0 ± 0 | 0 ± 0 | 24 ± 24 | 3249.67 ± 3192.17 | 9204 ± 4897.44 | 27.33 ± 13.68 |
| Rutstroemiaceae | 0.33 ± 0.33 | 0 ± 0 | 0 ± 0 | 0 ± 0 | 4.33 ± 1.86 | 1.33 ± 1.33 | 0.33 ± 0.33 | 0 ± 0 | 0 ± 0 | 3 ± 1.53 |
| Saccharataceae | 0 ± 0 | 0 ± 0 | 0 ± 0 | 0.33 ± 0.33 | 0.67 ± 0.67 | 0 ± 0 | 0 ± 0 | 0 ± 0 | 0 ± 0 | 0 ± 0 |
| Saccotheciaceae | 0.33 ± 0.33 | 0 ± 0 | 1 ± 1 | 0 ± 0 | 0 ± 0 | 0.33 ± 0.33 | 0 ± 0 | 3 ± 1.73 | 0 ± 0 | 0 ± 0 |
| Sanchytriaceae | 0 ± 0 | 0 ± 0 | 0 ± 0 | 0 ± 0 | 0 ± 0 | 0 ± 0 | 0 ± 0 | 0 ± 0 | 0.67 ± 0.67 | 0 ± 0 |
| Sarcosomataceae | 0 ± 0 | 0 ± 0 | 0.33 ± 0.33 | 0 ± 0 | 0 ± 0 | 0.33 ± 0.33 | 0 ± 0 | 0 ± 0 | 0 ± 0 | 0 ± 0 |
| Sarocladiaceae | 1.33 ± 0.88 | 39.33 ± 6.84 | 80 ± 15.5 | 14.67 ± 6.06 | 8.33 ± 4.33 | 16.67 ± 12.78 | 16.33 ± 8.37 | 28 ± 23.54 | 11 ± 5.57 | 12.67 ± 4.26 |
| Sarrameanales_norank | 0 ± 0 | 0 ± 0 | 0 ± 0 | 0 ± 0 | 0 ± 0 | 0 ± 0 | 0 ± 0 | 0.67 ± 0.67 | 1.67 ± 1.67 | 0 ± 0 |
| Savoryellaceae | 0.33 ± 0.33 | 0 ± 0 | 11.67 ± 4.37 | 0.33 ± 0.33 | 0.33 ± 0.33 | 13.33 ± 9.94 | 3 ± 2.08 | 2.67 ± 2.67 | 0 ± 0 | 2 ± 1.15 |
| Schizoparmaceae | 0 ± 0 | 0 ± 0 | 0 ± 0 | 0 ± 0 | 0 ± 0 | 0 ± 0 | 0 ± 0 | 1.33 ± 1.33 | 0 ± 0 | 0 ± 0 |
| Schizophyllaceae | 2 ± 2 | 0.33 ± 0.33 | 0 ± 0 | 0 ± 0 | 0.33 ± 0.33 | 0 ± 0 | 0 ± 0 | 0 ± 0 | 0 ± 0 | 0.33 ± 0.33 |
| Sclerodermataceae | 0 ± 0 | 0 ± 0 | 0 ± 0 | 0 ± 0 | 0 ± 0 | 4 ± 2.65 | 4.67 ± 4.67 | 84.67 ± 84.67 | 247 ± 141.46 | 0.33 ± 0.33 |
| Sclerotiniaceae | 0 ± 0 | 0.67 ± 0.67 | 9.33 ± 3.53 | 1.33 ± 0.88 | 0.33 ± 0.33 | 11.67 ± 6.69 | 4.67 ± 3.28 | 73.33 ± 46.46 | 12.67 ± 11.68 | 4.33 ± 1.86 |
| Scortechiniaceae | 1 ± 1 | 0 ± 0 | 0 ± 0 | 0 ± 0 | 0 ± 0 | 0 ± 0 | 0 ± 0 | 0 ± 0 | 0 ± 0 | 0 ± 0 |
| Sebacinaceae | 0.33 ± 0.33 | 1 ± 0.58 | 32 ± 13.08 | 0.33 ± 0.33 | 0.33 ± 0.33 | 6.67 ± 4.81 | 2.67 ± 1.45 | 30 ± 14.47 | 15.67 ± 15.17 | 2.33 ± 1.45 |
| Sebacinales_norank | 0 ± 0 | 0 ± 0 | 0 ± 0 | 0 ± 0 | 0 ± 0 | 0 ± 0 | 0 ± 0 | 0 ± 0 | 0.67 ± 0.67 | 0 ± 0 |
| Septobasidiaceae | 0.33 ± 0.33 | 0 ± 0 | 2.67 ± 2.67 | 0 ± 0 | 5.33 ± 3.93 | 0.33 ± 0.33 | 0.33 ± 0.33 | 0 ± 0 | 0.33 ± 0.33 | 3.67 ± 2.03 |
| Serendipitaceae | 1.33 ± 0.88 | 4.33 ± 2.96 | 4.67 ± 4.67 | 2 ± 0.58 | 5.67 ± 3.48 | 2 ± 1.15 | 1.33 ± 0.67 | 23.67 ± 2.91 | 7 ± 1.53 | 355.67 ± 167.3 |
| Sirobasidiaceae | 0.33 ± 0.33 | 0.33 ± 0.33 | 18.33 ± 18.33 | 0 ± 0 | 0 ± 0 | 1.33 ± 0.67 | 0 ± 0 | 0 ± 0 | 0 ± 0 | 0.67 ± 0.67 |
| Sonoraphlyctidaceae | 0 ± 0 | 9.67 ± 9.67 | 0 ± 0 | 0.67 ± 0.67 | 0 ± 0 | 0.33 ± 0.33 | 2 ± 2 | 0 ± 0 | 0 ± 0 | 0 ± 0 |
| Sordariaceae | 2.33 ± 0.33 | 18 ± 8.72 | 6.67 ± 6.67 | 1.67 ± 0.67 | 0 ± 0 | 17.67 ± 8.95 | 2.67 ± 1.76 | 1.67 ± 0.88 | 14.33 ± 10.35 | 9 ± 9 |
| Sordariales_norank | 67.67 ± 15.62 | 0.67 ± 0.67 | 14.67 ± 3.84 | 1.67 ± 1.2 | 62.67 ± 13.98 | 13 ± 7.51 | 7.33 ± 5.46 | 7.67 ± 5.78 | 0 ± 0 | 0.67 ± 0.67 |
| Sordariomycetes_norank | 14.67 ± 6.74 | 174.33 ± 54.66 | 1370 ± 1059.57 | 56.33 ± 11.29 | 38 ± 2.65 | 2705.33 ± 1171.43 | 824 ± 545.74 | 116 ± 48.81 | 74.67 ± 27.97 | 145.67 ± 18.98 |
| Spizellomycetaceae | 0 ± 0 | 0 ± 0 | 2 ± 1.15 | 0 ± 0 | 0 ± 0 | 0.33 ± 0.33 | 1 ± 1 | 0 ± 0 | 0.33 ± 0.33 | 0.33 ± 0.33 |
| Sporidesmiaceae | 0 ± 0 | 2 ± 2 | 1.67 ± 1.67 | 8.67 ± 3.38 | 0.33 ± 0.33 | 0 ± 0 | 12.33 ± 10.33 | 410.33 ± 207.96 | 219 ± 213 | 9.67 ± 6.12 |
| Sporidiobolaceae | 9 ± 9 | 2.33 ± 0.33 | 13 ± 8 | 5 ± 2.31 | 6 ± 0 | 6.67 ± 3.18 | 9.67 ± 2.03 | 5.67 ± 2.85 | 1.67 ± 0.33 | 19.33 ± 7.88 |
| Sporocadaceae | 6.33 ± 0.33 | 28.67 ± 7.75 | 303.67 ± 26.77 | 10 ± 3.61 | 5.33 ± 1.67 | 85 ± 63.1 | 52 ± 20.66 | 331.33 ± 158.04 | 101.67 ± 79.17 | 21.33 ± 0.33 |
| Sporormiaceae | 0.67 ± 0.33 | 54.67 ± 13.3 | 28.67 ± 5.36 | 1.33 ± 1.33 | 2 ± 1.53 | 8 ± 4.51 | 6 ± 3.21 | 1.67 ± 1.67 | 0.33 ± 0.33 | 1.67 ± 1.2 |
| Stachybotryaceae | 21.67 ± 7.75 | 64.67 ± 32.85 | 51.67 ± 5.49 | 8 ± 4.51 | 201 ± 14.22 | 63 ± 7.37 | 96.33 ± 58.62 | 42 ± 18.25 | 30 ± 4.04 | 132.67 ± 80.42 |
| Steccherinaceae | 0 ± 0 | 0 ± 0 | 0 ± 0 | 0 ± 0 | 0 ± 0 | 0 ± 0 | 0 ± 0 | 0 ± 0 | 0 ± 0 | 1.67 ± 1.67 |
| Stereaceae | 0 ± 0 | 0 ± 0 | 3.67 ± 3.67 | 0.33 ± 0.33 | 0 ± 0 | 0 ± 0 | 0 ± 0 | 0 ± 0 | 0 ± 0 | 0 ± 0 |
| Stictidaceae | 0 ± 0 | 2 ± 1.15 | 0 ± 0 | 0 ± 0 | 0 ± 0 | 0 ± 0 | 0 ± 0 | 0 ± 0 | 0 ± 0 | 0 ± 0 |
| Stilbosporaceae | 0 ± 0 | 0.33 ± 0.33 | 1 ± 0.58 | 0 ± 0 | 0.67 ± 0.33 | 97.67 ± 54.08 | 3.67 ± 2.03 | 0.67 ± 0.67 | 0 ± 0 | 12.67 ± 8.29 |
| Strigulaceae | 0 ± 0 | 0 ± 0 | 0 ± 0 | 0 ± 0 | 1.33 ± 1.33 | 0 ± 0 | 0 ± 0 | 0 ± 0 | 0 ± 0 | 0 ± 0 |
| Strophariaceae | 3.33 ± 2.85 | 0 ± 0 | 0.67 ± 0.67 | 0 ± 0 | 0.67 ± 0.67 | 12.33 ± 6.17 | 0.67 ± 0.67 | 8.33 ± 3.67 | 3 ± 1.53 | 3 ± 3 |
| Sulcatisporaceae | 0 ± 0 | 0 ± 0 | 1.33 ± 1.33 | 0.33 ± 0.33 | 0 ± 0 | 0.67 ± 0.33 | 1.33 ± 1.33 | 0 ± 0 | 0.33 ± 0.33 | 2 ± 0.58 |
| Symmetrosporaceae | 0 ± 0 | 0 ± 0 | 13.33 ± 6.01 | 0 ± 0 | 0.33 ± 0.33 | 0 ± 0 | 0 ± 0 | 0.33 ± 0.33 | 0.33 ± 0.33 | 4.33 ± 2.4 |
| Sympoventuriaceae | 1.33 ± 1.33 | 14.33 ± 3.48 | 0.33 ± 0.33 | 1 ± 0.58 | 0 ± 0 | 0 ± 0 | 2.67 ± 2.67 | 51.67 ± 45.17 | 14.67 ± 9.94 | 2.33 ± 1.2 |
| Taphrinaceae | 0 ± 0 | 0 ± 0 | 0 ± 0 | 0 ± 0 | 1 ± 1 | 1 ± 1 | 6.33 ± 6.33 | 0 ± 0 | 0 ± 0 | 1.33 ± 1.33 |
| Teichosporaceae | 0 ± 0 | 0 ± 0 | 0 ± 0 | 0 ± 0 | 0 ± 0 | 0 ± 0 | 0 ± 0 | 4.67 ± 4.67 | 0.67 ± 0.67 | 2.67 ± 2.67 |
| Teratosphaeriaceae | 5.67 ± 0.33 | 684.33 ± 137.03 | 23.33 ± 7.88 | 69.67 ± 18.85 | 1.33 ± 0.67 | 22 ± 9.45 | 155.33 ± 46.82 | 108.33 ± 92.33 | 208.33 ± 154.44 | 594 ± 196.46 |
| Tetragoniomycetaceae | 0 ± 0 | 0 ± 0 | 0 ± 0 | 0 ± 0 | 0 ± 0 | 0 ± 0 | 1 ± 1 | 0 ± 0 | 0 ± 0 | 0 ± 0 |
| Tetraplosphaeriaceae | 0 ± 0 | 0 ± 0 | 0 ± 0 | 0.33 ± 0.33 | 1.67 ± 0.88 | 0 ± 0 | 0.67 ± 0.67 | 7 ± 4.04 | 1.33 ± 1.33 | 0 ± 0 |
| Thelebolaceae | 2.67 ± 1.76 | 1 ± 0.58 | 1132.33 ± 579.9 | 9.33 ± 3.18 | 8.67 ± 2.91 | 142.33 ± 109.22 | 41 ± 23.39 | 1.33 ± 0.88 | 1.33 ± 0.88 | 1.67 ± 1.67 |
| Thelephoraceae | 0.33 ± 0.33 | 2.33 ± 1.33 | 27.67 ± 6.44 | 4.33 ± 2.6 | 3.33 ± 1.45 | 17.67 ± 4.67 | 12 ± 7.51 | 557.67 ± 545.68 | 512 ± 259.25 | 13.33 ± 4.48 |
| Thermoascaceae | 0 ± 0 | 0 ± 0 | 7 ± 4.04 | 0 ± 0 | 1.67 ± 1.2 | 42 ± 14.8 | 11 ± 10.02 | 0.33 ± 0.33 | 2 ± 0.58 | 0.67 ± 0.33 |
| Thyridariaceae | 0 ± 0 | 0.33 ± 0.33 | 2.67 ± 1.76 | 0 ± 0 | 3 ± 1.73 | 8 ± 4.36 | 14.67 ± 11.79 | 0 ± 0 | 0 ± 0 | 6.67 ± 2.73 |
| Tilachlidiaceae | 0 ± 0 | 0 ± 0 | 0.33 ± 0.33 | 0.33 ± 0.33 | 0 ± 0 | 5.67 ± 2.96 | 1 ± 0.58 | 4 ± 4 | 0 ± 0 | 0 ± 0 |
| Togniniaceae | 0 ± 0 | 0 ± 0 | 0.67 ± 0.67 | 0 ± 0 | 0.67 ± 0.33 | 0 ± 0 | 0 ± 0 | 0 ± 0 | 0 ± 0 | 0 ± 0 |
| Torulaceae | 5 ± 2.08 | 0.33 ± 0.33 | 2.33 ± 1.33 | 1 ± 0 | 1 ± 0.58 | 15.67 ± 5.55 | 10.33 ± 4.06 | 14.67 ± 6.98 | 24 ± 23.5 | 9 ± 3.06 |
| Trematosphaeriaceae | 0 ± 0 | 0 ± 0 | 0 ± 0 | 0 ± 0 | 0 ± 0 | 0.67 ± 0.67 | 0 ± 0 | 0 ± 0 | 0 ± 0 | 0 ± 0 |
| Tremellaceae | 22.67 ± 8.84 | 54.33 ± 46.94 | 22.33 ± 8.21 | 1.67 ± 0.88 | 3.33 ± 1.45 | 1 ± 0.58 | 11 ± 10.02 | 0 ± 0 | 1.67 ± 1.67 | 1.33 ± 0.88 |
| Tremellodendropsidaceae | 0 ± 0 | 0 ± 0 | 0 ± 0 | 0 ± 0 | 0 ± 0 | 0 ± 0 | 0 ± 0 | 0 ± 0 | 0 ± 0 | 0.67 ± 0.67 |
| Trichocomaceae | 1.67 ± 1.2 | 216 ± 26.66 | 18.67 ± 5.93 | 18 ± 4.73 | 0.33 ± 0.33 | 7.67 ± 0.33 | 67.33 ± 23.7 | 85.67 ± 43.07 | 39.33 ± 36.35 | 8.33 ± 2.91 |
| Tricholomataceae | 31 ± 16.65 | 1.67 ± 1.67 | 15.67 ± 10.37 | 1 ± 1 | 3.67 ± 2.03 | 5 ± 2.52 | 9.33 ± 2.4 | 57 ± 16.2 | 16 ± 15.5 | 18.33 ± 16.37 |
| Trichomeriaceae | 6 ± 1.73 | 627 ± 185.15 | 12.67 ± 2.6 | 35.33 ± 15.3 | 2 ± 1 | 13 ± 3 | 128.67 ± 42.48 | 144 ± 64.12 | 151.67 ± 122.32 | 1641.67 ± 802.65 |
| Trichomonascaceae | 0 ± 0 | 0 ± 0 | 0 ± 0 | 0 ± 0 | 0 ± 0 | 1.33 ± 0.88 | 1.33 ± 1.33 | 0 ± 0 | 0 ± 0 | 0 ± 0 |
| Trichosphaeriaceae | 7.67 ± 2.33 | 182.33 ± 169.38 | 53.33 ± 10.11 | 13.33 ± 6.89 | 8.67 ± 3.76 | 48.67 ± 17.17 | 81.33 ± 34.62 | 63 ± 35.93 | 14.67 ± 12.17 | 12 ± 5 |
| Trichosporonaceae | 16.33 ± 7.54 | 0 ± 0 | 7.67 ± 3.18 | 89 ± 17.69 | 605.33 ± 261.02 | 20.67 ± 12.2 | 104.67 ± 52.45 | 5.33 ± 4.84 | 4.33 ± 2.33 | 7.67 ± 4.98 |
| Trimorphomycetaceae | 9.67 ± 4.37 | 2386 ± 1287.1 | 246.33 ± 80.48 | 1158.67 ± 79.59 | 13.33 ± 2.91 | 163 ± 54.98 | 771 ± 282.37 | 275 ± 85.5 | 334.33 ± 87.19 | 5976.33 ± 1482.18 |
| Tubeufiaceae | 1.67 ± 0.33 | 0 ± 0 | 0 ± 0 | 0 ± 0 | 0 ± 0 | 0 ± 0 | 0 ± 0 | 0 ± 0 | 0 ± 0 | 0 ± 0 |
| Typhulaceae | 1.67 ± 0.67 | 0.67 ± 0.67 | 13.67 ± 6.44 | 13.67 ± 6.69 | 1 ± 1 | 0.33 ± 0.33 | 2.33 ± 1.86 | 0 ± 0 | 0.33 ± 0.33 | 3.67 ± 3.67 |
| Umbilicariaceae | 2 ± 1 | 0 ± 0 | 1.33 ± 0.88 | 0.67 ± 0.67 | 0.67 ± 0.67 | 0.33 ± 0.33 | 0.33 ± 0.33 | 0 ± 0 | 2.67 ± 2.67 | 12.33 ± 5.24 |
| Unclassified | 619.67 ± 282.27 | 1611.33 ± 506.5 | 971.33 ± 162.73 | 19219 ± 1922.36 | 1293.67 ± 101.41 | 1133.33 ± 159.31 | 7377.67 ± 2745.31 | 2392 ± 1032.17 | 2165 ± 1800.38 | 437.33 ± 83.59 |
| Ustilaginaceae | 0 ± 0 | 0 ± 0 | 1 ± 1 | 0 ± 0 | 0.33 ± 0.33 | 0.33 ± 0.33 | 0 ± 0 | 1.67 ± 1.67 | 0.33 ± 0.33 | 3.67 ± 3.67 |
| Valsaceae | 0 ± 0 | 0 ± 0 | 0 ± 0 | 0.33 ± 0.33 | 0.67 ± 0.67 | 0 ± 0 | 2 ± 2 | 0 ± 0 | 0 ± 0 | 2 ± 1.15 |
| Venturiaceae | 0 ± 0 | 0.33 ± 0.33 | 50.33 ± 6.57 | 5 ± 3.06 | 0.33 ± 0.33 | 7 ± 4.73 | 4.33 ± 2.33 | 1 ± 1 | 0.33 ± 0.33 | 0 ± 0 |
| Verrucariaceae | 0.67 ± 0.67 | 5.67 ± 3.84 | 14 ± 8.39 | 156.33 ± 73.75 | 6.67 ± 2.33 | 11.67 ± 10.17 | 91.33 ± 20.51 | 3018 ± 2983 | 1379.33 ± 1249.98 | 12.33 ± 5.49 |
| Xenospadicoidaceae | 0 ± 0 | 0 ± 0 | 0 ± 0 | 0 ± 0 | 0 ± 0 | 0.67 ± 0.67 | 0 ± 0 | 0 ± 0 | 0 ± 0 | 0 ± 0 |
| Xylariaceae | 1 ± 0 | 0 ± 0 | 11 ± 5.86 | 1.67 ± 1.2 | 8.67 ± 1.67 | 10.33 ± 1.67 | 11 ± 8.54 | 43.33 ± 38.44 | 7.67 ± 5.36 | 6 ± 1.53 |
| Xylariales_norank | 1.67 ± 0.33 | 0 ± 0 | 0 ± 0 | 1.33 ± 1.33 | 1.33 ± 0.33 | 3.67 ± 2.19 | 1 ± 0.58 | 6.33 ± 2.4 | 5 ± 3.61 | 13 ± 11.02 |
| Zoopagaceae | 0 ± 0 | 0 ± 0 | 0 ± 0 | 0 ± 0 | 0.33 ± 0.33 | 0.67 ± 0.67 | 0 ± 0 | 0.33 ± 0.33 | 0 ± 0 | 0 ± 0 |

**Table S13.** Abundance of Genus.

| Genus | F_S_1 | F_S_2 | F_T_1 | F_T_2 | N_A_1 | N_A_2 | N_B_1 | N_B_2 | M1 | M2 |
| --- | --- | --- | --- | --- | --- | --- | --- | --- | --- | --- |
| Aaosphaeria | 1.67 ± 1.2 | 5.33 ± 5.33 | 25.33 ± 5.84 | 0.67 ± 0.67 | 0.33 ± 0.33 | 13.33 ± 8.95 | 2 ± 1.53 | 1.33 ± 0.88 | 1.67 ± 0.88 | 5.67 ± 0.88 |
| Abrothallus | 0 ± 0 | 0 ± 0 | 13.33 ± 13.33 | 0 ± 0 | 0.67 ± 0.67 | 1 ± 0.58 | 2.67 ± 1.45 | 0 ± 0 | 0 ± 0 | 0 ± 0 |
| Acanthostigma | 0 ± 0 | 0 ± 0 | 0 ± 0 | 0 ± 0 | 0 ± 0 | 0 ± 0 | 0 ± 0 | 0 ± 0 | 0 ± 0 | 0 ± 0 |
| Acaulium | 0 ± 0 | 2.67 ± 2.67 | 0.67 ± 0.67 | 1 ± 0.58 | 0 ± 0 | 38 ± 23.81 | 1.67 ± 0.88 | 0 ± 0 | 0 ± 0 | 0 ± 0 |
| Acaulopage | 0 ± 0 | 0 ± 0 | 0 ± 0 | 0 ± 0 | 0.33 ± 0.33 | 0.67 ± 0.67 | 0 ± 0 | 0.33 ± 0.33 | 0 ± 0 | 0 ± 0 |
| Acephala | 0 ± 0 | 0 ± 0 | 0 ± 0 | 0 ± 0 | 0 ± 0 | 0 ± 0 | 0 ± 0 | 2.67 ± 1.45 | 0 ± 0 | 0 ± 0 |
| Achroceratosphaeria | 0 ± 0 | 0 ± 0 | 0.67 ± 0.67 | 0 ± 0 | 0 ± 0 | 4 ± 2.31 | 4 ± 4 | 4.67 ± 4.67 | 1.67 ± 1.2 | 0 ± 0 |
| Achroiostachys | 0.33 ± 0.33 | 7 ± 7 | 3.33 ± 1.76 | 0 ± 0 | 4 ± 0.58 | 3 ± 2.52 | 0.67 ± 0.67 | 0 ± 0 | 0 ± 0 | 0 ± 0 |
| Acremonium | 10.67 ± 3.28 | 73.67 ± 26.41 | 31.33 ± 8.65 | 27 ± 3.06 | 40.67 ± 11.86 | 438.33 ± 211.65 | 188.67 ± 118.67 | 63 ± 23.69 | 97.67 ± 77.74 | 110.33 ± 43.99 |
| Acrocalymma | 2.67 ± 1.76 | 2 ± 1.53 | 3 ± 2.08 | 2 ± 2 | 9 ± 4.04 | 6.67 ± 1.33 | 2.67 ± 2.19 | 13.67 ± 6.12 | 11.33 ± 9.33 | 6.67 ± 1.76 |
| Acrodictys | 0 ± 0 | 0 ± 0 | 0 ± 0 | 0 ± 0 | 0 ± 0 | 0 ± 0 | 0.67 ± 0.67 | 0 ± 0 | 0 ± 0 | 0 ± 0 |
| Acrodontium | 0 ± 0 | 0 ± 0 | 0 ± 0 | 0 ± 0 | 0 ± 0 | 1 ± 1 | 0 ± 0 | 0 ± 0 | 0 ± 0 | 0 ± 0 |
| Acrogenospora | 0 ± 0 | 0 ± 0 | 0 ± 0 | 0 ± 0 | 0 ± 0 | 0 ± 0 | 7.67 ± 7.67 | 0 ± 0 | 0.33 ± 0.33 | 0.33 ± 0.33 |
| Acrophialophora | 0 ± 0 | 0.67 ± 0.67 | 1 ± 1 | 0 ± 0 | 0.67 ± 0.67 | 0.33 ± 0.33 | 1 ± 0.58 | 0 ± 0 | 0 ± 0 | 0 ± 0 |
| Aculeata | 0 ± 0 | 0 ± 0 | 0 ± 0 | 0.33 ± 0.33 | 0 ± 0 | 0 ± 0 | 0.67 ± 0.67 | 0 ± 0 | 0 ± 0 | 0 ± 0 |
| Agonimia | 0 ± 0 | 0.33 ± 0.33 | 8 ± 7.51 | 1.33 ± 0.88 | 0 ± 0 | 8 ± 8 | 17.67 ± 14.19 | 2998.67 ± 2972.17 | 1357.33 ± 1242.24 | 8.67 ± 4.37 |
| Agrocybe | 0 ± 0 | 0 ± 0 | 0 ± 0 | 0 ± 0 | 0 ± 0 | 0.33 ± 0.33 | 0 ± 0 | 0 ± 0 | 0 ± 0 | 0 ± 0 |
| Alatospora | 0.67 ± 0.67 | 0 ± 0 | 28.33 ± 8.88 | 0.67 ± 0.67 | 3.67 ± 0.67 | 4.33 ± 3.38 | 0.67 ± 0.33 | 13 ± 11.53 | 5.33 ± 4.84 | 0 ± 0 |
| Albifimbria | 0 ± 0 | 1.67 ± 1.67 | 39.67 ± 10.99 | 0 ± 0 | 0.33 ± 0.33 | 7 ± 3.06 | 2 ± 1.53 | 5.67 ± 3.67 | 0.33 ± 0.33 | 27 ± 8.08 |
| Aleurodiscus | 0 ± 0 | 0 ± 0 | 3.67 ± 3.67 | 0.33 ± 0.33 | 0 ± 0 | 0 ± 0 | 0 ± 0 | 0 ± 0 | 0 ± 0 | 0 ± 0 |
| Alfaria | 0.33 ± 0.33 | 14.33 ± 6.69 | 0.33 ± 0.33 | 1.33 ± 0.88 | 0.33 ± 0.33 | 0.33 ± 0.33 | 4 ± 2.65 | 0 ± 0 | 0.33 ± 0.33 | 0.67 ± 0.67 |
| Alloclavaria | 0 ± 0 | 0 ± 0 | 6.33 ± 2.19 | 0 ± 0 | 0 ± 0 | 0 ± 0 | 0.33 ± 0.33 | 0 ± 0 | 0 ± 0 | 0 ± 0 |
| Alnicola | 0 ± 0 | 0 ± 0 | 0 ± 0 | 0 ± 0 | 0 ± 0 | 1 ± 1 | 0 ± 0 | 0 ± 0 | 0 ± 0 | 3 ± 3 |
| Alternaria | 0.67 ± 0.67 | 0.33 ± 0.33 | 6.67 ± 2.73 | 0.67 ± 0.67 | 1.33 ± 0.33 | 13 ± 6.51 | 6.67 ± 1.76 | 106 ± 56.93 | 76.67 ± 73.67 | 6 ± 3.51 |
| Amanita | 0 ± 0 | 0 ± 0 | 0 ± 0 | 0 ± 0 | 0 ± 0 | 0 ± 0 | 0.67 ± 0.67 | 2.33 ± 2.33 | 10.33 ± 6.74 | 0 ± 0 |
| Amauroascus | 0 ± 0 | 0 ± 0 | 0 ± 0 | 0 ± 0 | 0 ± 0 | 0 ± 0 | 0.33 ± 0.33 | 0 ± 0 | 0 ± 0 | 0 ± 0 |
| Ambispora | 0 ± 0 | 1 ± 1 | 0 ± 0 | 0 ± 0 | 0 ± 0 | 0 ± 0 | 0 ± 0 | 0 ± 0 | 0 ± 0 | 0 ± 0 |
| Amesia | 1 ± 0.58 | 0 ± 0 | 16.33 ± 6.23 | 1.33 ± 0.88 | 0.67 ± 0.67 | 25.67 ± 6.39 | 33.67 ± 28.81 | 76.67 ± 73.18 | 12 ± 5.57 | 5 ± 1.15 |
| Amoeboradix | 0 ± 0 | 0 ± 0 | 0 ± 0 | 0 ± 0 | 0 ± 0 | 0 ± 0 | 0 ± 0 | 0 ± 0 | 0.67 ± 0.67 | 0 ± 0 |
| Ampelomyces | 0 ± 0 | 0 ± 0 | 5.67 ± 5.17 | 0.33 ± 0.33 | 0 ± 0 | 0 ± 0 | 0.33 ± 0.33 | 0 ± 0 | 0 ± 0 | 0 ± 0 |
| Amphinema | 0 ± 0 | 0 ± 0 | 1 ± 1 | 0 ± 0 | 0 ± 0 | 0.33 ± 0.33 | 0 ± 0 | 0 ± 0 | 0 ± 0 | 0 ± 0 |
| Amphirosellinia | 0 ± 0 | 0 ± 0 | 0.67 ± 0.67 | 0 ± 0 | 0 ± 0 | 0 ± 0 | 0 ± 0 | 0 ± 0 | 0 ± 0 | 0 ± 0 |
| Amylocorticiellum | 0 ± 0 | 0 ± 0 | 0 ± 0 | 0 ± 0 | 0 ± 0 | 0 ± 0 | 1.33 ± 1.33 | 0 ± 0 | 0 ± 0 | 0 ± 0 |
| Amylocorticium | 0 ± 0 | 0 ± 0 | 0 ± 0 | 1 ± 1 | 0 ± 0 | 0 ± 0 | 0 ± 0 | 0.33 ± 0.33 | 0 ± 0 | 0 ± 0 |
| Annulohypoxylon | 0 ± 0 | 0.33 ± 0.33 | 0.33 ± 0.33 | 0.67 ± 0.67 | 0 ± 0 | 0 ± 0 | 0 ± 0 | 0 ± 0 | 0 ± 0 | 0 ± 0 |
| Anthopsis | 0 ± 0 | 0 ± 0 | 0.33 ± 0.33 | 0 ± 0 | 0 ± 0 | 2 ± 2 | 0.67 ± 0.33 | 0 ± 0 | 0 ± 0 | 0 ± 0 |
| Anthostoma | 10 ± 5.13 | 0 ± 0 | 1.33 ± 0.88 | 0 ± 0 | 1 ± 0.58 | 0 ± 0 | 0 ± 0 | 0 ± 0 | 0 ± 0 | 0 ± 0 |
| Anthostomella | 0 ± 0 | 0 ± 0 | 3.67 ± 3.67 | 0 ± 0 | 0 ± 0 | 0 ± 0 | 0.33 ± 0.33 | 0 ± 0 | 0 ± 0 | 0 ± 0 |
| Aphanocladium | 0 ± 0 | 2 ± 2 | 0 ± 0 | 0.33 ± 0.33 | 0.33 ± 0.33 | 0.67 ± 0.67 | 2 ± 1.53 | 4 ± 2 | 1.67 ± 1.2 | 1.67 ± 1.67 |
| Aphanophora | 0 ± 0 | 0 ± 0 | 0 ± 0 | 0.67 ± 0.67 | 0 ± 0 | 0 ± 0 | 0.67 ± 0.67 | 26 ± 13.01 | 17 ± 16.5 | 5 ± 4.51 |
| Apiosordaria | 0 ± 0 | 0.33 ± 0.33 | 0.67 ± 0.67 | 2.67 ± 2.67 | 8.67 ± 4.63 | 27.67 ± 21.4 | 4 ± 2 | 58.67 ± 35.3 | 7 ± 7 | 1 ± 1 |
| Apiotrichum | 0 ± 0 | 0 ± 0 | 0 ± 0 | 5 ± 3.61 | 0 ± 0 | 0 ± 0 | 1.33 ± 0.88 | 0 ± 0 | 0 ± 0 | 1 ± 1 |
| Apodus | 1.33 ± 1.33 | 0 ± 0 | 51.67 ± 9.21 | 1.67 ± 0.33 | 0 ± 0 | 7.67 ± 5.7 | 4.33 ± 2.96 | 1 ± 0.58 | 0 ± 0 | 1.67 ± 1.67 |
| Apophysomyces | 0 ± 0 | 0 ± 0 | 2 ± 1.15 | 0 ± 0 | 0 ± 0 | 0 ± 0 | 0 ± 0 | 0 ± 0 | 0 ± 0 | 0 ± 0 |
| Arachnotheca | 0 ± 0 | 0 ± 0 | 0 ± 0 | 0 ± 0 | 0 ± 0 | 0 ± 0 | 2.67 ± 2.67 | 1 ± 0.58 | 2.33 ± 2.33 | 0.67 ± 0.67 |
| Archaeorhizomyces | 24282.67 ± 1343.46 | 1902 ± 1079.9 | 687.67 ± 78.27 | 1637.67 ± 112.48 | 22225.33 ± 741.27 | 622 ± 333.52 | 2052.33 ± 929.68 | 892 ± 866.53 | 2692 ± 1372.29 | 105 ± 66.68 |
| Archaeospora | 0 ± 0 | 8.33 ± 4.33 | 0 ± 0 | 1.67 ± 0.88 | 0 ± 0 | 1 ± 1 | 1.67 ± 1.2 | 0 ± 0 | 1 ± 1 | 2.33 ± 1.2 |
| Aristastoma | 132 ± 126.51 | 3.67 ± 3.67 | 0.67 ± 0.33 | 0 ± 0 | 13 ± 11.53 | 1.33 ± 1.33 | 1.33 ± 0.67 | 0.33 ± 0.33 | 0 ± 0 | 23 ± 20.01 |
| Arizonaphlyctis | 0.33 ± 0.33 | 3.67 ± 3.67 | 0 ± 0 | 0.67 ± 0.67 | 0 ± 0 | 0 ± 0 | 0 ± 0 | 0 ± 0 | 0 ± 0 | 0 ± 0 |
| Arrhenia | 1.33 ± 1.33 | 1.33 ± 1.33 | 1 ± 1 | 0.67 ± 0.67 | 0 ± 0 | 1 ± 0.58 | 0.33 ± 0.33 | 18.33 ± 18.33 | 0.67 ± 0.33 | 0 ± 0 |
| Arthopyrenia | 0 ± 0 | 0 ± 0 | 0.67 ± 0.67 | 0 ± 0 | 0 ± 0 | 0 ± 0 | 0 ± 0 | 0 ± 0 | 0 ± 0 | 0 ± 0 |
| Arthrinium | 0.33 ± 0.33 | 0 ± 0 | 0.67 ± 0.67 | 0 ± 0 | 0 ± 0 | 0 ± 0 | 13.33 ± 13.33 | 14 ± 9.07 | 8 ± 7.51 | 8.67 ± 1.86 |
| Arthrobotrys | 1.33 ± 1.33 | 10.33 ± 5.9 | 2 ± 0.58 | 0.33 ± 0.33 | 0 ± 0 | 20.33 ± 11.84 | 1 ± 0.58 | 44 ± 41.51 | 6.33 ± 2.91 | 4.33 ± 2.96 |
| Arthrocladium | 0.67 ± 0.67 | 0 ± 0 | 1 ± 1 | 0 ± 0 | 1.33 ± 0.33 | 1.33 ± 0.67 | 0 ± 0 | 4.67 ± 2.6 | 3.67 ± 3.67 | 2 ± 1.15 |
| Arthropsis | 0.67 ± 0.67 | 25.67 ± 12.39 | 0.33 ± 0.33 | 6 ± 1.73 | 0 ± 0 | 3 ± 2.08 | 13 ± 10.15 | 1 ± 1 | 3.67 ± 2.73 | 0 ± 0 |
| Articulospora | 0.67 ± 0.33 | 0 ± 0 | 10.33 ± 4.37 | 0.33 ± 0.33 | 0.67 ± 0.33 | 6.67 ± 3.53 | 7 ± 0.58 | 8.33 ± 5.61 | 3 ± 1.73 | 5.33 ± 2.33 |
| Arxiella | 7.67 ± 3.71 | 674.67 ± 633.94 | 88 ± 67.55 | 30 ± 24.7 | 3.33 ± 1.45 | 9.33 ± 7.42 | 285.33 ± 279.84 | 77.67 ± 38.4 | 55.67 ± 54.17 | 45.67 ± 20.43 |
| Aschersonia | 0 ± 0 | 0 ± 0 | 0 ± 0 | 0 ± 0 | 0 ± 0 | 0.33 ± 0.33 | 0 ± 0 | 0 ± 0 | 0 ± 0 | 0 ± 0 |
| Ascitendus | 0 ± 0 | 0 ± 0 | 0 ± 0 | 0 ± 0 | 0 ± 0 | 2.33 ± 1.45 | 1.67 ± 1.67 | 0 ± 0 | 0 ± 0 | 0 ± 0 |
| Ascobolus | 0 ± 0 | 0 ± 0 | 0 ± 0 | 0 ± 0 | 0 ± 0 | 1.67 ± 1.67 | 0 ± 0 | 0 ± 0 | 0 ± 0 | 0 ± 0 |
| Ascodesmis | 0 ± 0 | 1 ± 0.58 | 0 ± 0 | 0 ± 0 | 0.33 ± 0.33 | 0 ± 0 | 0 ± 0 | 2.33 ± 2.33 | 1.67 ± 1.2 | 0 ± 0 |
| Ascorhizoctonia | 14.67 ± 9.39 | 0.33 ± 0.33 | 0 ± 0 | 0 ± 0 | 0.33 ± 0.33 | 0 ± 0 | 0.33 ± 0.33 | 0 ± 0 | 0 ± 0 | 0 ± 0 |
| Ascosphaera | 0 ± 0 | 0 ± 0 | 0 ± 0 | 0 ± 0 | 0 ± 0 | 0 ± 0 | 0 ± 0 | 4 ± 2.65 | 0 ± 0 | 0 ± 0 |
| Ascotaiwania | 0.33 ± 0.33 | 0 ± 0 | 11.67 ± 4.37 | 0.33 ± 0.33 | 0.33 ± 0.33 | 13.33 ± 9.94 | 3 ± 2.08 | 2.67 ± 2.67 | 0 ± 0 | 1.67 ± 0.88 |
| Aspergillus | 3.33 ± 0.67 | 15.33 ± 6.69 | 48 ± 4.73 | 5.33 ± 0.67 | 2.33 ± 0.88 | 108.67 ± 69.44 | 11.67 ± 4.63 | 79.67 ± 39.43 | 26 ± 24.52 | 489.67 ± 465.7 |
| Astraeus | 0 ± 0 | 0 ± 0 | 0.67 ± 0.67 | 0 ± 0 | 0 ± 0 | 0 ± 0 | 0 ± 0 | 0 ± 0 | 0 ± 0 | 0 ± 0 |
| Astrocystis | 0 ± 0 | 0 ± 0 | 1.33 ± 1.33 | 0 ± 0 | 0 ± 0 | 0 ± 0 | 1.33 ± 0.88 | 0 ± 0 | 0 ± 0 | 0.33 ± 0.33 |
| Atractiella | 1 ± 1 | 281.67 ± 149.45 | 0 ± 0 | 19.67 ± 5.84 | 0 ± 0 | 0.67 ± 0.67 | 53.67 ± 38.37 | 2.33 ± 2.33 | 0 ± 0 | 0.33 ± 0.33 |
| Atractium | 0.67 ± 0.33 | 0 ± 0 | 3.67 ± 1.76 | 3 ± 1.73 | 11.67 ± 3.84 | 1 ± 0 | 2.33 ± 1.2 | 0 ± 0 | 0 ± 0 | 1.67 ± 0.88 |
| Atractospora | 0 ± 0 | 0 ± 0 | 6 ± 3.06 | 4.67 ± 3.67 | 0 ± 0 | 49.67 ± 27.91 | 5 ± 2.89 | 23.67 ± 23.67 | 0.33 ± 0.33 | 44 ± 21.36 |
| Atrocalyx | 0 ± 0 | 0 ± 0 | 0 ± 0 | 0 ± 0 | 0 ± 0 | 130 ± 118.63 | 1 ± 1 | 0 ± 0 | 0 ± 0 | 2 ± 2 |
| Aureobasidium | 0.33 ± 0.33 | 0 ± 0 | 1 ± 1 | 0 ± 0 | 0 ± 0 | 0.33 ± 0.33 | 0 ± 0 | 3 ± 1.73 | 0 ± 0 | 0 ± 0 |
| Auriculoscypha | 0.33 ± 0.33 | 0 ± 0 | 0 ± 0 | 0 ± 0 | 5.33 ± 3.93 | 0 ± 0 | 0.33 ± 0.33 | 0 ± 0 | 0 ± 0 | 0 ± 0 |
| Austroafricana | 0 ± 0 | 0 ± 0 | 1.33 ± 1.33 | 0 ± 0 | 0 ± 0 | 0 ± 0 | 1 ± 1 | 0 ± 0 | 0 ± 0 | 0 ± 0 |
| Auxarthron | 1 ± 0.58 | 0 ± 0 | 0 ± 0 | 0.33 ± 0.33 | 2.33 ± 0.88 | 0 ± 0 | 3 ± 2.52 | 0.67 ± 0.67 | 0 ± 0 | 10.67 ± 5.7 |
| Bacidia | 0 ± 0 | 0 ± 0 | 0 ± 0 | 0 ± 0 | 0 ± 0 | 0 ± 0 | 0 ± 0 | 0 ± 0 | 0 ± 0 | 0.33 ± 0.33 |
| Bacidina | 0.67 ± 0.67 | 0 ± 0 | 0.67 ± 0.67 | 3.33 ± 3.33 | 0.33 ± 0.33 | 0 ± 0 | 0.67 ± 0.67 | 0.67 ± 0.67 | 8 ± 6.11 | 0 ± 0 |
| Bacillicladium | 0 ± 0 | 0 ± 0 | 5 ± 4.04 | 0 ± 0 | 0 ± 0 | 0.33 ± 0.33 | 0 ± 0 | 0 ± 0 | 0 ± 0 | 0 ± 0 |
| Bactrodesmium | 0 ± 0 | 0 ± 0 | 0 ± 0 | 0 ± 0 | 0 ± 0 | 0 ± 0 | 1.67 ± 1.67 | 3.33 ± 3.33 | 0 ± 0 | 3 ± 1.73 |
| Bagliettoa | 0 ± 0 | 0 ± 0 | 0 ± 0 | 0 ± 0 | 0 ± 0 | 0 ± 0 | 0.67 ± 0.67 | 0.67 ± 0.67 | 2.33 ± 1.45 | 0 ± 0 |
| Bagnisiella | 0 ± 0 | 0 ± 0 | 0 ± 0 | 0 ± 0 | 0 ± 0 | 0 ± 0 | 0 ± 0 | 1.67 ± 1.67 | 0 ± 0 | 0 ± 0 |
| Banksiophoma | 0 ± 0 | 0 ± 0 | 0 ± 0 | 0 ± 0 | 0 ± 0 | 0.33 ± 0.33 | 0.33 ± 0.33 | 0 ± 0 | 0.67 ± 0.33 | 0 ± 0 |
| Bannoa | 0 ± 0 | 0 ± 0 | 3.33 ± 3.33 | 0 ± 0 | 0 ± 0 | 0 ± 0 | 1 ± 0.58 | 0 ± 0 | 0 ± 0 | 0 ± 0 |
| Basidioascus | 0 ± 0 | 0 ± 0 | 4.67 ± 2.19 | 0 ± 0 | 1.33 ± 0.88 | 0.33 ± 0.33 | 0 ± 0 | 0 ± 0 | 0 ± 0 | 0 ± 0 |
| Basidiobolus | 0 ± 0 | 0 ± 0 | 0 ± 0 | 0 ± 0 | 2.33 ± 2.33 | 0 ± 0 | 13.67 ± 13.67 | 0 ± 0 | 1.33 ± 0.88 | 0 ± 0 |
| Batcheloromyces | 0 ± 0 | 6 ± 2.08 | 0 ± 0 | 0.67 ± 0.67 | 0 ± 0 | 0 ± 0 | 2 ± 0.58 | 13 ± 13 | 67.67 ± 58.42 | 55.33 ± 37.24 |
| Beauveria | 19 ± 10 | 6.33 ± 2.73 | 106 ± 6.03 | 155 ± 76.27 | 24.33 ± 10.73 | 98 ± 38.28 | 223 ± 18.25 | 41 ± 19.09 | 25 ± 19.55 | 137.67 ± 29.18 |
| Beltrania | 0 ± 0 | 0 ± 0 | 0 ± 0 | 0 ± 0 | 0 ± 0 | 5.67 ± 5.67 | 1 ± 1 | 1.33 ± 1.33 | 1 ± 1 | 6.67 ± 5.17 |
| Beltraniella | 0 ± 0 | 0 ± 0 | 0 ± 0 | 0 ± 0 | 0 ± 0 | 0 ± 0 | 0 ± 0 | 0 ± 0 | 0 ± 0 | 0 ± 0 |
| Berkleasmium | 1.67 ± 0.33 | 0 ± 0 | 0 ± 0 | 0 ± 0 | 0 ± 0 | 0 ± 0 | 0 ± 0 | 0 ± 0 | 0 ± 0 | 0 ± 0 |
| Betamyces | 0 ± 0 | 2.67 ± 2.67 | 2.33 ± 0.67 | 1.33 ± 0.67 | 0 ± 0 | 1.33 ± 1.33 | 0.67 ± 0.67 | 0.33 ± 0.33 | 0 ± 0 | 0 ± 0 |
| Bettsia | 0 ± 0 | 0.33 ± 0.33 | 0 ± 0 | 10.33 ± 2.4 | 0.67 ± 0.67 | 0 ± 0 | 2 ± 1 | 0 ± 0 | 0 ± 0 | 0 ± 0 |
| Beverwykella | 0 ± 0 | 0 ± 0 | 0 ± 0 | 0 ± 0 | 0.33 ± 0.33 | 0.33 ± 0.33 | 0.33 ± 0.33 | 0 ± 0 | 0 ± 0 | 0 ± 0 |
| Biappendiculispora | 0 ± 0 | 7.67 ± 3.48 | 0 ± 0 | 0.33 ± 0.33 | 0 ± 0 | 0 ± 0 | 0.67 ± 0.67 | 0 ± 0 | 0 ± 0 | 0 ± 0 |
| Biatriospora | 0 ± 0 | 0 ± 0 | 0 ± 0 | 0 ± 0 | 0 ± 0 | 30 ± 16.52 | 0 ± 0 | 0 ± 0 | 0 ± 0 | 0 ± 0 |
| Bipolaris | 0 ± 0 | 6.67 ± 5.7 | 0 ± 0 | 0.33 ± 0.33 | 0 ± 0 | 1 ± 1 | 0.33 ± 0.33 | 0 ± 0 | 0 ± 0 | 0 ± 0 |
| Bloxamia | 0 ± 0 | 0.33 ± 0.33 | 0 ± 0 | 2 ± 1.15 | 0 ± 0 | 4.33 ± 2.96 | 18.67 ± 15.67 | 1 ± 0.58 | 2.67 ± 2.67 | 0.33 ± 0.33 |
| Bolbitius | 0 ± 0 | 0 ± 0 | 0.67 ± 0.67 | 0 ± 0 | 0 ± 0 | 0 ± 0 | 0 ± 0 | 0 ± 0 | 0 ± 0 | 0 ± 0 |
| Boletus | 0 ± 0 | 0 ± 0 | 0 ± 0 | 0 ± 0 | 0 ± 0 | 0 ± 0 | 0 ± 0 | 0 ± 0 | 0 ± 0 | 2 ± 2 |
| Botryoderma | 0 ± 0 | 1.33 ± 0.88 | 0 ± 0 | 0.67 ± 0.67 | 0 ± 0 | 0.33 ± 0.33 | 3 ± 3 | 0 ± 0 | 0.33 ± 0.33 | 7 ± 6.51 |
| Botryohypochnus | 0 ± 0 | 0 ± 0 | 0 ± 0 | 0 ± 0 | 0 ± 0 | 0 ± 0 | 0 ± 0 | 3.33 ± 3.33 | 8.67 ± 5.55 | 0 ± 0 |
| Botryosphaeria | 0 ± 0 | 0 ± 0 | 0 ± 0 | 0.33 ± 0.33 | 0 ± 0 | 0 ± 0 | 0.67 ± 0.67 | 1.33 ± 0.88 | 1 ± 0.58 | 0 ± 0 |
| Botryotrichum | 3.67 ± 3.67 | 0 ± 0 | 3.67 ± 3.67 | 0 ± 0 | 0 ± 0 | 13.67 ± 8.09 | 21.33 ± 20.34 | 3 ± 1 | 0.33 ± 0.33 | 22 ± 22 |
| Botrytis | 0 ± 0 | 0 ± 0 | 7.33 ± 4.33 | 1 ± 1 | 0 ± 0 | 9 ± 4.93 | 2.67 ± 2.19 | 73 ± 46.14 | 12.67 ± 11.68 | 0 ± 0 |
| Boubovia | 2.33 ± 1.33 | 0.67 ± 0.67 | 0 ± 0 | 0 ± 0 | 0 ± 0 | 2 ± 1.53 | 0 ± 0 | 0.33 ± 0.33 | 0 ± 0 | 0 ± 0 |
| Brachyphoris | 0 ± 0 | 0 ± 0 | 0 ± 0 | 0 ± 0 | 0 ± 0 | 0 ± 0 | 0 ± 0 | 3 ± 1.53 | 0.33 ± 0.33 | 0 ± 0 |
| Bryochiton | 0 ± 0 | 0 ± 0 | 0 ± 0 | 0 ± 0 | 0 ± 0 | 0 ± 0 | 0 ± 0 | 0 ± 0 | 1 ± 1 | 0 ± 0 |
| Buckleyzyma | 0.33 ± 0.33 | 0 ± 0 | 0 ± 0 | 0 ± 0 | 0 ± 0 | 0.33 ± 0.33 | 0.67 ± 0.67 | 1.33 ± 1.33 | 0 ± 0 | 0.33 ± 0.33 |
| Bullera | 1.67 ± 1.67 | 0 ± 0 | 22.33 ± 22.33 | 0 ± 0 | 0.33 ± 0.33 | 0.67 ± 0.67 | 2 ± 1.15 | 0 ± 0 | 0 ± 0 | 0.33 ± 0.33 |
| Bulleribasidium | 1 ± 1 | 0.67 ± 0.33 | 40 ± 24.11 | 35 ± 25.06 | 0.33 ± 0.33 | 2 ± 1.53 | 12 ± 2 | 0.33 ± 0.33 | 0 ± 0 | 8 ± 5.29 |
| Burgoa | 1.67 ± 1.67 | 0 ± 0 | 0 ± 0 | 0 ± 0 | 0 ± 0 | 0 ± 0 | 0 ± 0 | 0 ± 0 | 0 ± 0 | 0 ± 0 |
| Byssochlamys | 0 ± 0 | 0 ± 0 | 0.67 ± 0.33 | 0 ± 0 | 0 ± 0 | 0.67 ± 0.67 | 8.33 ± 8.33 | 0.33 ± 0.33 | 1.67 ± 0.88 | 0 ± 0 |
| Byssocorticium | 0 ± 0 | 0.67 ± 0.67 | 0 ± 0 | 0 ± 0 | 0 ± 0 | 0 ± 0 | 0 ± 0 | 0 ± 0 | 0 ± 0 | 0 ± 0 |
| Byssonectria | 0 ± 0 | 3.67 ± 1.45 | 0.33 ± 0.33 | 0 ± 0 | 0 ± 0 | 0 ± 0 | 0.33 ± 0.33 | 0 ± 0 | 0 ± 0 | 5.33 ± 4.84 |
| Cadophora | 7.33 ± 0.88 | 17.67 ± 11.22 | 7.67 ± 1.33 | 1.67 ± 0.33 | 0.33 ± 0.33 | 4.67 ± 4.18 | 28.33 ± 23.88 | 57 ± 33.42 | 14.33 ± 6.96 | 35.67 ± 20.5 |
| Cainia | 0 ± 0 | 0 ± 0 | 0 ± 0 | 0 ± 0 | 0.33 ± 0.33 | 0 ± 0 | 0 ± 0 | 0.33 ± 0.33 | 0 ± 0 | 0 ± 0 |
| Calcarisporiella | 0 ± 0 | 0 ± 0 | 0 ± 0 | 0 ± 0 | 0 ± 0 | 0 ± 0 | 0 ± 0 | 0 ± 0 | 0 ± 0 | 1.33 ± 0.88 |
| Calocera | 0.67 ± 0.67 | 0 ± 0 | 0 ± 0 | 0 ± 0 | 0 ± 0 | 0 ± 0 | 0 ± 0 | 0 ± 0 | 0 ± 0 | 0 ± 0 |
| Calonectria | 0 ± 0 | 0 ± 0 | 0 ± 0 | 2.67 ± 1.45 | 0 ± 0 | 0 ± 0 | 1 ± 1 | 0 ± 0 | 0 ± 0 | 0 ± 0 |
| Calvolachnella | 4 ± 0.58 | 0 ± 0 | 0 ± 0 | 0 ± 0 | 0 ± 0 | 0 ± 0 | 3.67 ± 3.67 | 0 ± 0 | 0 ± 0 | 0 ± 0 |
| Calycellina | 0 ± 0 | 0 ± 0 | 0.33 ± 0.33 | 1.33 ± 0.33 | 0 ± 0 | 0 ± 0 | 0.33 ± 0.33 | 0 ± 0 | 0 ± 0 | 0 ± 0 |
| Calycina | 0 ± 0 | 0 ± 0 | 0 ± 0 | 0 ± 0 | 0 ± 0 | 0 ± 0 | 0 ± 0 | 0 ± 0 | 0.67 ± 0.67 | 0 ± 0 |
| Calyptella | 22 ± 12.42 | 0.33 ± 0.33 | 1.33 ± 1.33 | 0 ± 0 | 2 ± 1.15 | 1 ± 0.58 | 0 ± 0 | 0 ± 0 | 0 ± 0 | 0 ± 0 |
| Camaropella | 0 ± 0 | 0 ± 0 | 0 ± 0 | 0 ± 0 | 0 ± 0 | 0 ± 0 | 0 ± 0 | 0 ± 0 | 0 ± 0 | 0 ± 0 |
| Camposporium | 0 ± 0 | 0 ± 0 | 4 ± 4 | 0 ± 0 | 0 ± 0 | 0 ± 0 | 0 ± 0 | 1 ± 0.58 | 0 ± 0 | 71.67 ± 69.67 |
| Camptophora | 0 ± 0 | 0.33 ± 0.33 | 0 ± 0 | 1 ± 1 | 0 ± 0 | 0 ± 0 | 1 ± 0.58 | 11.67 ± 5.46 | 9 ± 9 | 11 ± 5.69 |
| Campylocarpon | 1 ± 0.58 | 0 ± 0 | 0 ± 0 | 0 ± 0 | 0 ± 0 | 0 ± 0 | 0 ± 0 | 0 ± 0 | 0 ± 0 | 0 ± 0 |
| Canalisporium | 0 ± 0 | 0 ± 0 | 0 ± 0 | 0 ± 0 | 0 ± 0 | 1.67 ± 1.67 | 0 ± 0 | 0 ± 0 | 0 ± 0 | 0 ± 0 |
| Candelariella | 0 ± 0 | 0 ± 0 | 0 ± 0 | 0 ± 0 | 0 ± 0 | 0 ± 0 | 0 ± 0 | 0 ± 0 | 2.33 ± 2.33 | 0 ± 0 |
| Candida | 0 ± 0 | 0 ± 0 | 0 ± 0 | 0 ± 0 | 0 ± 0 | 0.33 ± 0.33 | 0 ± 0 | 0 ± 0 | 0.67 ± 0.67 | 0 ± 0 |
| Capnobotryella | 0 ± 0 | 0 ± 0 | 0 ± 0 | 0 ± 0 | 0 ± 0 | 0 ± 0 | 0 ± 0 | 0 ± 0 | 0 ± 0 | 0.33 ± 0.33 |
| Capnodium | 0 ± 0 | 0 ± 0 | 1.33 ± 1.33 | 0 ± 0 | 0 ± 0 | 0 ± 0 | 0 ± 0 | 0 ± 0 | 0 ± 0 | 0 ± 0 |
| Capronia | 0.33 ± 0.33 | 22 ± 20.52 | 0 ± 0 | 6 ± 6 | 0.33 ± 0.33 | 2 ± 1 | 6 ± 3.06 | 15.33 ± 3.48 | 31 ± 5.13 | 7.33 ± 7.33 |
| Castanediella | 1 ± 0.58 | 0 ± 0 | 0 ± 0 | 0 ± 0 | 0 ± 0 | 0 ± 0 | 0 ± 0 | 0.33 ± 0.33 | 1 ± 1 | 0 ± 0 |
| Cataractispora | 0.33 ± 0.33 | 20.67 ± 1.76 | 0 ± 0 | 0 ± 0 | 0 ± 0 | 0.67 ± 0.33 | 2.33 ± 1.86 | 0 ± 0 | 0 ± 0 | 0 ± 0 |
| Catenophlyctis | 0 ± 0 | 0 ± 0 | 0 ± 0 | 0 ± 0 | 0 ± 0 | 0.67 ± 0.67 | 0 ± 0 | 0 ± 0 | 0 ± 0 | 0.67 ± 0.67 |
| Celerioriella | 1 ± 0.58 | 0 ± 0 | 0 ± 0 | 0 ± 0 | 0 ± 0 | 0 ± 0 | 0 ± 0 | 0 ± 0 | 0 ± 0 | 0 ± 0 |
| Cenangiopsis | 0 ± 0 | 0 ± 0 | 0 ± 0 | 0 ± 0 | 0 ± 0 | 0.67 ± 0.67 | 0 ± 0 | 0 ± 0 | 0 ± 0 | 0 ± 0 |
| Cenococcum | 0 ± 0 | 0 ± 0 | 0 ± 0 | 2.67 ± 1.76 | 0 ± 0 | 0 ± 0 | 1 ± 0.58 | 0.33 ± 0.33 | 1 ± 0.58 | 30 ± 22.59 |
| Cephaliophora | 0 ± 0 | 0 ± 0 | 0 ± 0 | 0 ± 0 | 1.67 ± 1.67 | 0 ± 0 | 0 ± 0 | 0 ± 0 | 0 ± 0 | 0 ± 0 |
| Cephalosporium | 0 ± 0 | 0 ± 0 | 0 ± 0 | 0 ± 0 | 0 ± 0 | 0.67 ± 0.67 | 0 ± 0 | 0 ± 0 | 0 ± 0 | 0 ± 0 |
| Cephalotrichiella | 0 ± 0 | 0 ± 0 | 0 ± 0 | 0 ± 0 | 0 ± 0 | 0 ± 0 | 0 ± 0 | 0 ± 0 | 0 ± 0 | 0.33 ± 0.33 |
| Ceramothyrium | 0 ± 0 | 8.67 ± 4.67 | 0.67 ± 0.67 | 1.33 ± 0.67 | 0 ± 0 | 1 ± 1 | 2.33 ± 2.33 | 22.67 ± 22.17 | 3.67 ± 3.67 | 15.33 ± 9.26 |
| Ceratobasidium | 43.67 ± 4.33 | 1.33 ± 1.33 | 0.67 ± 0.33 | 15 ± 5.29 | 215.33 ± 78.96 | 4 ± 1.53 | 113.67 ± 79.69 | 12.33 ± 8.35 | 1.33 ± 0.88 | 28.67 ± 12.35 |
| Cercophora | 11 ± 4.62 | 3.33 ± 1.33 | 535 ± 433.81 | 75.33 ± 34.57 | 54.33 ± 6.84 | 140 ± 60.04 | 166.67 ± 41.32 | 76.67 ± 56.3 | 4.67 ± 3.28 | 18.67 ± 16.17 |
| Cercospora | 0 ± 0 | 0 ± 0 | 0 ± 0 | 1.67 ± 1.2 | 0 ± 0 | 0 ± 0 | 1 ± 0.58 | 1 ± 0.58 | 1 ± 1 | 0 ± 0 |
| Cercosporella | 0 ± 0 | 0 ± 0 | 0 ± 0 | 0 ± 0 | 0.67 ± 0.67 | 0 ± 0 | 0 ± 0 | 0 ± 0 | 0 ± 0 | 0 ± 0 |
| Chaetomella | 0 ± 0 | 0 ± 0 | 0 ± 0 | 0 ± 0 | 0 ± 0 | 0 ± 0 | 0 ± 0 | 0 ± 0 | 0 ± 0 | 0.67 ± 0.67 |
| Chaetomidium | 0 ± 0 | 0 ± 0 | 0 ± 0 | 0 ± 0 | 0 ± 0 | 0 ± 0 | 0 ± 0 | 0 ± 0 | 0 ± 0 | 3.33 ± 3.33 |
| Chaetomium | 3.33 ± 1.33 | 117.33 ± 36.93 | 791.33 ± 421.6 | 18.33 ± 4.91 | 6.67 ± 0.88 | 229.33 ± 93.6 | 95.33 ± 48.77 | 67.33 ± 32.77 | 12.67 ± 10.17 | 51 ± 18.23 |
| Chaetospermum | 0 ± 0 | 0 ± 0 | 0 ± 0 | 0 ± 0 | 0 ± 0 | 0 ± 0 | 0 ± 0 | 0 ± 0 | 0.67 ± 0.67 | 0 ± 0 |
| Chaetosphaeria | 0 ± 0 | 0 ± 0 | 2.33 ± 2.33 | 0 ± 0 | 3.67 ± 3.67 | 4.33 ± 4.33 | 0 ± 0 | 0 ± 0 | 0 ± 0 | 1.67 ± 1.67 |
| Chaetosphaeronema | 0 ± 0 | 3.33 ± 2.4 | 0 ± 0 | 0 ± 0 | 0.33 ± 0.33 | 0 ± 0 | 0 ± 0 | 0 ± 0 | 0 ± 0 | 0 ± 0 |
| Chalara | 2.33 ± 1.86 | 9.67 ± 3.71 | 89.67 ± 29.98 | 2.67 ± 1.33 | 2.33 ± 1.45 | 26.33 ± 15.86 | 21.67 ± 11.46 | 20 ± 12.66 | 9 ± 8.5 | 181 ± 162.51 |
| Cheilymenia | 10.33 ± 3.48 | 0 ± 0 | 0 ± 0 | 0 ± 0 | 5.67 ± 4.18 | 0 ± 0 | 0.33 ± 0.33 | 0 ± 0 | 0 ± 0 | 0 ± 0 |
| Cheirosporium | 0 ± 0 | 0 ± 0 | 0 ± 0 | 0 ± 0 | 0 ± 0 | 0 ± 0 | 4.33 ± 4.33 | 0 ± 0 | 0 ± 0 | 1.33 ± 0.88 |
| Chlamydocillium | 0 ± 0 | 0 ± 0 | 0 ± 0 | 0 ± 0 | 0 ± 0 | 0.67 ± 0.67 | 0 ± 0 | 0 ± 0 | 0 ± 0 | 0 ± 0 |
| Chloridium | 0.67 ± 0.33 | 4.33 ± 1.86 | 174 ± 71.59 | 13 ± 7.64 | 6.33 ± 1.86 | 109.67 ± 28.87 | 12.33 ± 7.88 | 2.33 ± 0.88 | 2.33 ± 2.33 | 6.67 ± 1.45 |
| Chrysosporium | 0 ± 0 | 0 ± 0 | 0 ± 0 | 0 ± 0 | 0.67 ± 0.33 | 0.67 ± 0.67 | 5.33 ± 5.33 | 0 ± 0 | 0.33 ± 0.33 | 1.67 ± 1.67 |
| Chytriomyces | 0 ± 0 | 0 ± 0 | 0.67 ± 0.67 | 0 ± 0 | 0 ± 0 | 0 ± 0 | 0 ± 0 | 0 ± 0 | 0 ± 0 | 0 ± 0 |
| Ciboria | 0 ± 0 | 0 ± 0 | 0 ± 0 | 0.33 ± 0.33 | 0.33 ± 0.33 | 0.33 ± 0.33 | 2 ± 1.15 | 0 ± 0 | 0 ± 0 | 0 ± 0 |
| Ciliolarina | 0 ± 0 | 0 ± 0 | 37.67 ± 14.19 | 1.67 ± 0.88 | 0 ± 0 | 7.33 ± 4.67 | 1.67 ± 0.88 | 0 ± 0 | 0.33 ± 0.33 | 0 ± 0 |
| Circinotrichum | 0 ± 0 | 0 ± 0 | 0.33 ± 0.33 | 0 ± 0 | 0.67 ± 0.33 | 0 ± 0 | 0.33 ± 0.33 | 33 ± 32.5 | 3.33 ± 1.67 | 0 ± 0 |
| Cladobotryum | 0 ± 0 | 0 ± 0 | 2.67 ± 2.67 | 0 ± 0 | 0.67 ± 0.33 | 4 ± 2.08 | 4.33 ± 4.33 | 0 ± 0 | 0.33 ± 0.33 | 2.67 ± 2.19 |
| Cladophialophora | 13.33 ± 4.37 | 771 ± 308.07 | 524.67 ± 114.81 | 162.33 ± 32.67 | 1.67 ± 0.33 | 147 ± 110.53 | 284.33 ± 60.83 | 223.33 ± 53.7 | 193.33 ± 40.04 | 203.67 ± 75.49 |
| Cladorrhinum | 0.33 ± 0.33 | 1.33 ± 0.33 | 4.33 ± 2.4 | 4.33 ± 3.84 | 4.67 ± 2.03 | 24.33 ± 16.83 | 16.33 ± 10.9 | 32.67 ± 28.67 | 0 ± 0 | 1 ± 1 |
| Cladosporium | 81 ± 60.51 | 114.67 ± 67.85 | 497.33 ± 296.47 | 56 ± 21.63 | 34 ± 7.81 | 230 ± 69.87 | 220 ± 75.02 | 187.67 ± 90.46 | 78 ± 62.74 | 234 ± 93.72 |
| Claroideoglomus | 0 ± 0 | 1.33 ± 1.33 | 0.33 ± 0.33 | 0 ± 0 | 3 ± 1.15 | 0.33 ± 0.33 | 1 ± 0.58 | 1.33 ± 0.33 | 1 ± 0.58 | 0 ± 0 |
| Classicula | 0 ± 0 | 0 ± 0 | 0 ± 0 | 0.67 ± 0.67 | 2 ± 1.53 | 0 ± 0 | 1 ± 1 | 0.33 ± 0.33 | 0 ± 0 | 0 ± 0 |
| Claussenomyces | 0.33 ± 0.33 | 68.67 ± 43.73 | 8.33 ± 6.84 | 6.67 ± 4.67 | 0 ± 0 | 3.67 ± 2.03 | 13.33 ± 9.06 | 1.67 ± 0.67 | 3.33 ± 2.85 | 57.67 ± 53.17 |
| Clavaria | 772.33 ± 165.84 | 30.67 ± 20.34 | 1 ± 0.58 | 6.67 ± 3.67 | 37.33 ± 4.91 | 4.33 ± 2.85 | 17 ± 8.5 | 506 ± 503 | 68.67 ± 41.91 | 5.67 ± 1.76 |
| Clavariopsis | 0 ± 0 | 0 ± 0 | 0 ± 0 | 0 ± 0 | 0 ± 0 | 0 ± 0 | 0 ± 0 | 0 ± 0 | 0 ± 0 | 1.33 ± 1.33 |
| Claviceps | 1.67 ± 1.2 | 0 ± 0 | 0 ± 0 | 0 ± 0 | 0 ± 0 | 0 ± 0 | 0 ± 0 | 0 ± 0 | 0 ± 0 | 1.67 ± 1.67 |
| Clavicorona | 101 ± 17.06 | 1 ± 0.58 | 13 ± 5.29 | 27.67 ± 13.93 | 11.67 ± 4.18 | 5.67 ± 2.85 | 37.33 ± 22.88 | 0.33 ± 0.33 | 0.67 ± 0.67 | 1 ± 1 |
| Clavulina | 2 ± 1 | 6.67 ± 1.76 | 4 ± 1.73 | 230.67 ± 98.38 | 0.33 ± 0.33 | 2.67 ± 2.19 | 99.33 ± 51.56 | 0 ± 0 | 0 ± 0 | 0 ± 0 |
| Clitocybe | 0 ± 0 | 0 ± 0 | 6.67 ± 6.67 | 0 ± 0 | 0 ± 0 | 0 ± 0 | 0.33 ± 0.33 | 0 ± 0 | 0 ± 0 | 1 ± 1 |
| Clitopilus | 1 ± 0 | 173.33 ± 69.34 | 48.67 ± 25.44 | 45.67 ± 33.53 | 1 ± 1 | 7.33 ± 3.71 | 32.33 ± 17.37 | 26.33 ± 8.25 | 44.33 ± 4.48 | 251.67 ± 109.04 |
| Clonostachys | 35.67 ± 6.36 | 174.67 ± 70.59 | 145 ± 96.5 | 18.67 ± 6.89 | 8.67 ± 3.18 | 54 ± 11.37 | 47.33 ± 18.56 | 178.33 ± 88.74 | 60.33 ± 57.84 | 11.33 ± 2.67 |
| Clypeosphaeria | 0 ± 0 | 0 ± 0 | 0 ± 0 | 0 ± 0 | 0 ± 0 | 0 ± 0 | 0 ± 0 | 1 ± 1 | 0 ± 0 | 0 ± 0 |
| Codinaea | 0.33 ± 0.33 | 43.67 ± 7.06 | 0 ± 0 | 4.33 ± 3.84 | 0 ± 0 | 0 ± 0 | 4.33 ± 2.33 | 2.33 ± 1.86 | 2.33 ± 2.33 | 0 ± 0 |
| Colacogloea | 0 ± 0 | 0 ± 0 | 0 ± 0 | 0 ± 0 | 0 ± 0 | 0 ± 0 | 0 ± 0 | 1.33 ± 1.33 | 0 ± 0 | 1.33 ± 1.33 |
| Coleophoma | 0 ± 0 | 0 ± 0 | 0 ± 0 | 0 ± 0 | 0 ± 0 | 0 ± 0 | 0 ± 0 | 1 ± 1 | 0 ± 0 | 0 ± 0 |
| Collariella | 0 ± 0 | 0 ± 0 | 0 ± 0 | 0 ± 0 | 0 ± 0 | 0 ± 0 | 0 ± 0 | 0 ± 0 | 0 ± 0 | 0 ± 0 |
| Collarina | 0 ± 0 | 0 ± 0 | 7.33 ± 4.37 | 0.67 ± 0.67 | 0.33 ± 0.33 | 1 ± 1 | 9 ± 8.5 | 1 ± 0.58 | 2.33 ± 1.45 | 1.33 ± 0.33 |
| Colletotrichum | 0 ± 0 | 8.33 ± 0.88 | 11 ± 1 | 13 ± 3.79 | 0.33 ± 0.33 | 70 ± 29.5 | 11.67 ± 3.18 | 2.67 ± 2.19 | 5.67 ± 2.03 | 21 ± 10.54 |
| Collophorina | 0 ± 0 | 0 ± 0 | 0 ± 0 | 4.33 ± 2.33 | 0 ± 0 | 0 ± 0 | 0.33 ± 0.33 | 0.33 ± 0.33 | 0 ± 0 | 1.33 ± 1.33 |
| Confertobasidium | 0 ± 0 | 0 ± 0 | 0 ± 0 | 0 ± 0 | 0 ± 0 | 0.67 ± 0.67 | 0 ± 0 | 0 ± 0 | 0 ± 0 | 0 ± 0 |
| Coniella | 0 ± 0 | 0 ± 0 | 0 ± 0 | 0 ± 0 | 0 ± 0 | 0 ± 0 | 0 ± 0 | 1.33 ± 1.33 | 0 ± 0 | 0 ± 0 |
| Coniochaeta | 0 ± 0 | 24.33 ± 8.99 | 5.67 ± 3.71 | 1 ± 1 | 3.33 ± 1.86 | 29.33 ± 12.55 | 2 ± 1.53 | 3.33 ± 1.2 | 1.67 ± 1.67 | 4 ± 3.06 |
| Coniophora | 0 ± 0 | 0 ± 0 | 0 ± 0 | 0 ± 0 | 0 ± 0 | 0 ± 0 | 0 ± 0 | 0 ± 0 | 0.33 ± 0.33 | 0 ± 0 |
| Conioscypha | 0 ± 0 | 1 ± 1 | 0 ± 0 | 0 ± 0 | 0 ± 0 | 0 ± 0 | 0 ± 0 | 0 ± 0 | 1.33 ± 1.33 | 0 ± 0 |
| Coniosporium | 2 ± 2 | 64.33 ± 40.24 | 0 ± 0 | 1 ± 0.58 | 0 ± 0 | 1.33 ± 1.33 | 5.67 ± 5.17 | 1.67 ± 1.2 | 4.33 ± 2.19 | 57.67 ± 50.78 |
| Coniothyrium | 2.67 ± 0.88 | 2.67 ± 1.67 | 43 ± 11.93 | 6.67 ± 2.96 | 1.33 ± 0.67 | 42.67 ± 13.78 | 35.67 ± 21.5 | 4.67 ± 2.4 | 5.67 ± 4.7 | 14 ± 5 |
| Conlarium | 0 ± 0 | 8.33 ± 2.73 | 1 ± 1 | 0.33 ± 0.33 | 0 ± 0 | 0 ± 0 | 1.33 ± 1.33 | 0 ± 0 | 0.33 ± 0.33 | 0 ± 0 |
| Conocybe | 0 ± 0 | 0 ± 0 | 4.67 ± 2.33 | 0 ± 0 | 0 ± 0 | 1.67 ± 1.67 | 0 ± 0 | 0 ± 0 | 0 ± 0 | 0 ± 0 |
| Coprinellus | 0.67 ± 0.33 | 0.33 ± 0.33 | 18.33 ± 1.2 | 0.33 ± 0.33 | 3.67 ± 1.33 | 17.33 ± 8.25 | 15.33 ± 5.84 | 3 ± 3 | 5 ± 4.51 | 0.33 ± 0.33 |
| Coprinopsis | 1.67 ± 0.88 | 1.67 ± 1.67 | 0 ± 0 | 0 ± 0 | 0.67 ± 0.33 | 0.33 ± 0.33 | 0.33 ± 0.33 | 0.33 ± 0.33 | 0 ± 0 | 1.33 ± 1.33 |
| Coprinus | 0 ± 0 | 0 ± 0 | 0 ± 0 | 0 ± 0 | 0 ± 0 | 2 ± 2 | 0 ± 0 | 0 ± 0 | 0 ± 0 | 0 ± 0 |
| Cordana | 0 ± 0 | 0 ± 0 | 22.67 ± 4.18 | 0 ± 0 | 0 ± 0 | 10 ± 3.06 | 4.33 ± 2.4 | 5 ± 1.73 | 16.33 ± 11.29 | 10 ± 1.53 |
| Cordyceps | 0.67 ± 0.33 | 1.67 ± 1.2 | 7.33 ± 1.45 | 24 ± 10.41 | 7 ± 2.89 | 37.33 ± 9.33 | 71 ± 38.5 | 24.67 ± 15.41 | 8.33 ± 6.84 | 39.33 ± 9.6 |
| Corinectria | 0 ± 0 | 0 ± 0 | 0 ± 0 | 0 ± 0 | 0 ± 0 | 0.67 ± 0.67 | 0 ± 0 | 0 ± 0 | 0 ± 0 | 0 ± 0 |
| Corticium | 0.33 ± 0.33 | 1.33 ± 0.67 | 5.67 ± 1.2 | 18.33 ± 4.18 | 0 ± 0 | 0.33 ± 0.33 | 11.33 ± 5.93 | 0 ± 0 | 0.67 ± 0.67 | 0 ± 0 |
| Cortinarius | 0 ± 0 | 0 ± 0 | 0 ± 0 | 0 ± 0 | 0 ± 0 | 0 ± 0 | 0 ± 0 | 0 ± 0 | 0 ± 0 | 7 ± 7 |
| Corynascella | 0 ± 0 | 0.33 ± 0.33 | 0 ± 0 | 0 ± 0 | 0 ± 0 | 1 ± 1 | 0.67 ± 0.67 | 0 ± 0 | 0 ± 0 | 0 ± 0 |
| Corynespora | 0.33 ± 0.33 | 0 ± 0 | 0 ± 0 | 0 ± 0 | 1.33 ± 1.33 | 0 ± 0 | 0 ± 0 | 0 ± 0 | 0 ± 0 | 0 ± 0 |
| Cosmospora | 0 ± 0 | 0 ± 0 | 0.33 ± 0.33 | 0.33 ± 0.33 | 0 ± 0 | 2 ± 1.15 | 1 ± 1 | 0 ± 0 | 0 ± 0 | 0.67 ± 0.67 |
| Cotylidia | 0 ± 0 | 8.67 ± 4.91 | 0 ± 0 | 0.33 ± 0.33 | 0 ± 0 | 0 ± 0 | 1 ± 0.58 | 0 ± 0 | 0 ± 0 | 0 ± 0 |
| Crassiclypeus | 0 ± 0 | 0 ± 0 | 0.33 ± 0.33 | 0 ± 0 | 0 ± 0 | 0 ± 0 | 0 ± 0 | 0 ± 0 | 0 ± 0 | 0 ± 0 |
| Creosphaeria | 0 ± 0 | 0 ± 0 | 0 ± 0 | 0 ± 0 | 0 ± 0 | 5 ± 2.52 | 3.67 ± 3.18 | 1.33 ± 1.33 | 0 ± 0 | 0.67 ± 0.67 |
| Crepidotus | 0 ± 0 | 0 ± 0 | 0 ± 0 | 0 ± 0 | 0 ± 0 | 0 ± 0 | 0 ± 0 | 0.33 ± 0.33 | 1.67 ± 1.67 | 0 ± 0 |
| Crustoderma | 0 ± 0 | 0 ± 0 | 1 ± 1 | 0 ± 0 | 0 ± 0 | 0.33 ± 0.33 | 0 ± 0 | 0 ± 0 | 0 ± 0 | 0 ± 0 |
| Cryomyces | 0 ± 0 | 0 ± 0 | 0 ± 0 | 0 ± 0 | 0 ± 0 | 0 ± 0 | 0 ± 0 | 3 ± 3 | 0 ± 0 | 0 ± 0 |
| Cryptococcus | 3 ± 1.53 | 1.33 ± 0.88 | 4.67 ± 1.45 | 15.67 ± 11.67 | 70 ± 29.5 | 8 ± 0 | 7 ± 1.53 | 16.67 ± 7.84 | 15.33 ± 12.84 | 310.33 ± 65.79 |
| Cryptocoryneum | 0 ± 0 | 0.33 ± 0.33 | 0 ± 0 | 0 ± 0 | 0 ± 0 | 0 ± 0 | 0.33 ± 0.33 | 0 ± 0 | 0 ± 0 | 0 ± 0 |
| Cryptodiscus | 0 ± 0 | 0 ± 0 | 0 ± 0 | 0 ± 0 | 0 ± 0 | 0 ± 0 | 0 ± 0 | 0 ± 0 | 0 ± 0 | 0 ± 0 |
| Cryptosphaeria | 0 ± 0 | 0 ± 0 | 0 ± 0 | 0 ± 0 | 0 ± 0 | 0 ± 0 | 0.67 ± 0.67 | 0 ± 0 | 0 ± 0 | 0 ± 0 |
| Cryptosporiopsis | 0.33 ± 0.33 | 3 ± 3 | 1 ± 0.58 | 3.67 ± 1.86 | 0 ± 0 | 1.33 ± 0.33 | 3.67 ± 1.76 | 0.67 ± 0.67 | 0.33 ± 0.33 | 1 ± 1 |
| Curreya | 0 ± 0 | 0 ± 0 | 0 ± 0 | 0.33 ± 0.33 | 0 ± 0 | 1.67 ± 1.67 | 1.33 ± 1.33 | 0.33 ± 0.33 | 1.33 ± 1.33 | 1.33 ± 1.33 |
| Curvibasidium | 0 ± 0 | 0 ± 0 | 0 ± 0 | 0 ± 0 | 0 ± 0 | 0.33 ± 0.33 | 0.33 ± 0.33 | 0 ± 0 | 0 ± 0 | 0.33 ± 0.33 |
| Curvularia | 0 ± 0 | 1.33 ± 1.33 | 1 ± 1 | 0 ± 0 | 0 ± 0 | 0.33 ± 0.33 | 0 ± 0 | 0.67 ± 0.33 | 0 ± 0 | 2.33 ± 2.33 |
| Cutaneotrichosporon | 0 ± 0 | 0 ± 0 | 0 ± 0 | 0.33 ± 0.33 | 0.33 ± 0.33 | 0.33 ± 0.33 | 0.33 ± 0.33 | 0 ± 0 | 1.67 ± 1.67 | 5.67 ± 5.67 |
| Cylindrocarpon | 49.67 ± 22.42 | 111.67 ± 47.37 | 591.67 ± 84.36 | 35 ± 13.45 | 92.33 ± 4.91 | 590.33 ± 58.94 | 877.67 ± 745.1 | 43.33 ± 23.48 | 18.67 ± 10.73 | 133.67 ± 8.25 |
| Cylindrocladiella | 0.33 ± 0.33 | 0.33 ± 0.33 | 4.33 ± 4.33 | 1.33 ± 1.33 | 7.67 ± 1.86 | 23 ± 14.64 | 27 ± 17.21 | 0 ± 0 | 0 ± 0 | 4.67 ± 4.67 |
| Cylindrodendrum | 0 ± 0 | 0 ± 0 | 0 ± 0 | 0 ± 0 | 0 ± 0 | 0 ± 0 | 0.33 ± 0.33 | 0 ± 0 | 1.33 ± 0.88 | 1 ± 1 |
| Cylindrosympodium | 0 ± 0 | 0 ± 0 | 0 ± 0 | 0 ± 0 | 0 ± 0 | 0 ± 0 | 0 ± 0 | 0 ± 0 | 0 ± 0 | 0 ± 0 |
| Cyphellophora | 19.67 ± 9.24 | 1.33 ± 0.88 | 17.33 ± 10.35 | 3 ± 1.73 | 6.33 ± 1.86 | 29.67 ± 5.24 | 25.67 ± 11.05 | 164 ± 107.82 | 78.67 ± 72.69 | 13.67 ± 6.64 |
| Cyphellophoriella | 0 ± 0 | 0 ± 0 | 0 ± 0 | 0.33 ± 0.33 | 0 ± 0 | 0 ± 0 | 0 ± 0 | 0 ± 0 | 0 ± 0 | 0 ± 0 |
| Cystobasidium | 0 ± 0 | 0 ± 0 | 0 ± 0 | 0 ± 0 | 0 ± 0 | 0.67 ± 0.33 | 0 ± 0 | 0 ± 0 | 0 ± 0 | 1 ± 1 |
| Cystofilobasidium | 0 ± 0 | 0 ± 0 | 39 ± 12.42 | 1 ± 0.58 | 2.67 ± 2.19 | 7 ± 5.51 | 37.67 ± 36.17 | 31.33 ± 19.81 | 5.67 ± 4.18 | 16.67 ± 8.35 |
| Cystostereum | 0 ± 0 | 0 ± 0 | 0 ± 0 | 0 ± 0 | 0 ± 0 | 0 ± 0 | 2.33 ± 2.33 | 0 ± 0 | 0 ± 0 | 0 ± 0 |
| Dactylaria | 1.67 ± 1.2 | 19 ± 7.77 | 60.67 ± 14.45 | 3.33 ± 0.67 | 2 ± 1.53 | 39.67 ± 16.33 | 18.33 ± 3.84 | 80 ± 45.65 | 32.67 ± 22.18 | 4.67 ± 2.67 |
| Dactylella | 0 ± 0 | 0 ± 0 | 2 ± 1.53 | 0 ± 0 | 0.33 ± 0.33 | 2.67 ± 1.45 | 0 ± 0 | 105.33 ± 53.14 | 55 ± 55 | 0.67 ± 0.33 |
| Dactylellina | 0 ± 0 | 0 ± 0 | 0 ± 0 | 0 ± 0 | 0 ± 0 | 0 ± 0 | 0 ± 0 | 0 ± 0 | 0 ± 0 | 0.67 ± 0.67 |
| Dactylonectria | 106.67 ± 19.36 | 21 ± 5.2 | 891 ± 51.1 | 127.33 ± 31.76 | 547 ± 169.61 | 1218 ± 157.57 | 745.67 ± 488.02 | 154.33 ± 37.71 | 141 ± 55.59 | 428.33 ± 93.68 |
| Daldinia | 0 ± 0 | 0 ± 0 | 0 ± 0 | 0.67 ± 0.67 | 0 ± 0 | 0 ± 0 | 0.67 ± 0.67 | 1 ± 1 | 0.33 ± 0.33 | 0 ± 0 |
| Davidhawksworthia | 0 ± 0 | 0 ± 0 | 2.33 ± 2.33 | 0 ± 0 | 0 ± 0 | 1 ± 1 | 0 ± 0 | 0 ± 0 | 0 ± 0 | 0 ± 0 |
| Debaryomyces | 0 ± 0 | 0 ± 0 | 0 ± 0 | 0 ± 0 | 0 ± 0 | 0 ± 0 | 0 ± 0 | 1.33 ± 1.33 | 0 ± 0 | 0 ± 0 |
| Deconica | 0 ± 0 | 0 ± 0 | 0 ± 0 | 0 ± 0 | 0 ± 0 | 0.67 ± 0.67 | 0 ± 0 | 2 ± 2 | 0 ± 0 | 0 ± 0 |
| Degelia | 1 ± 1 | 2.33 ± 2.33 | 6 ± 2.65 | 3 ± 0.58 | 0 ± 0 | 2 ± 1.15 | 27 ± 25.03 | 0.33 ± 0.33 | 0 ± 0 | 0.33 ± 0.33 |
| Delastria | 0.33 ± 0.33 | 0 ± 0 | 2 ± 2 | 0 ± 0 | 0.67 ± 0.33 | 0 ± 0 | 0 ± 0 | 1.33 ± 1.33 | 0 ± 0 | 0.33 ± 0.33 |
| Dendroclathra | 0 ± 0 | 0 ± 0 | 6.67 ± 4.7 | 0 ± 0 | 2.67 ± 2.19 | 2.67 ± 2.67 | 0.33 ± 0.33 | 0 ± 0 | 0 ± 0 | 0 ± 0 |
| Dendrosporium | 0 ± 0 | 0 ± 0 | 0 ± 0 | 0 ± 0 | 0 ± 0 | 0 ± 0 | 0 ± 0 | 5.67 ± 4.7 | 5.67 ± 5.67 | 0 ± 0 |
| Dendryphion | 3.67 ± 1.76 | 0.33 ± 0.33 | 1 ± 0.58 | 0.67 ± 0.33 | 1 ± 0.58 | 11 ± 2.08 | 5 ± 2.52 | 3 ± 3 | 0.33 ± 0.33 | 5.33 ± 2.73 |
| Densocarpa | 0 ± 0 | 0 ± 0 | 7.67 ± 5.36 | 0.33 ± 0.33 | 0 ± 0 | 1.33 ± 1.33 | 21 ± 21 | 0 ± 0 | 4 ± 4 | 0 ± 0 |
| Dermea | 0.67 ± 0.67 | 0 ± 0 | 3 ± 1.53 | 3.33 ± 3.33 | 4.67 ± 1.2 | 31 ± 13 | 16.33 ± 8.21 | 0 ± 0 | 0 ± 0 | 0 ± 0 |
| Dermoloma | 0 ± 0 | 0 ± 0 | 0 ± 0 | 0 ± 0 | 0 ± 0 | 0 ± 0 | 0 ± 0 | 0 ± 0 | 0 ± 0 | 15.67 ± 15.67 |
| Derxomyces | 0 ± 0 | 0 ± 0 | 0.67 ± 0.67 | 0 ± 0 | 0 ± 0 | 1.67 ± 1.67 | 0 ± 0 | 0.33 ± 0.33 | 0 ± 0 | 0.33 ± 0.33 |
| Devriesia | 5.67 ± 0.33 | 678.33 ± 135.68 | 22 ± 8.5 | 68.67 ± 17.95 | 1.33 ± 0.67 | 14 ± 11.02 | 152.33 ± 47.14 | 92.33 ± 79.35 | 136 ± 91.98 | 528.67 ± 233.92 |
| Dialonectria | 0 ± 0 | 0 ± 0 | 0 ± 0 | 0 ± 0 | 3 ± 3 | 0 ± 0 | 0 ± 0 | 0 ± 0 | 0 ± 0 | 0 ± 0 |
| Diaporthe | 1 ± 1 | 8 ± 7.02 | 11.33 ± 11.33 | 0.33 ± 0.33 | 0 ± 0 | 0.67 ± 0.67 | 3 ± 3 | 0 ± 0 | 0.67 ± 0.33 | 97 ± 95 |
| Dictyochaeta | 0 ± 0 | 0 ± 0 | 0 ± 0 | 0 ± 0 | 0 ± 0 | 0 ± 0 | 0 ± 0 | 0 ± 0 | 0 ± 0 | 0 ± 0 |
| Dictyocheirospora | 0 ± 0 | 0 ± 0 | 1.67 ± 1.67 | 0 ± 0 | 0 ± 0 | 1 ± 1 | 0.67 ± 0.67 | 1 ± 1 | 0 ± 0 | 0 ± 0 |
| Dictyosporella | 0 ± 0 | 0.67 ± 0.67 | 0 ± 0 | 0 ± 0 | 0 ± 0 | 0 ± 0 | 0.33 ± 0.33 | 0 ± 0 | 0 ± 0 | 0.67 ± 0.67 |
| Dictyosporium | 0 ± 0 | 0.67 ± 0.67 | 0 ± 0 | 0.67 ± 0.67 | 0 ± 0 | 0 ± 0 | 0 ± 0 | 0 ± 0 | 0 ± 0 | 0 ± 0 |
| Dicyma | 0 ± 0 | 0 ± 0 | 0 ± 0 | 1.33 ± 1.33 | 0.67 ± 0.67 | 0 ± 0 | 0 ± 0 | 0 ± 0 | 0 ± 0 | 0 ± 0 |
| Didymella | 59.67 ± 21.14 | 26 ± 7.23 | 292.33 ± 132.23 | 102 ± 76.57 | 156.33 ± 48.53 | 733.67 ± 398.31 | 432 ± 86.05 | 269.67 ± 196.63 | 201.67 ± 197.17 | 87.33 ± 3.18 |
| Dioszegia | 0.67 ± 0.33 | 0 ± 0 | 9.33 ± 6.17 | 1.67 ± 0.88 | 5 ± 2 | 6.67 ± 3.18 | 2 ± 2 | 5 ± 4.51 | 2.33 ± 2.33 | 11.33 ± 5.55 |
| Discosia | 0 ± 0 | 0 ± 0 | 24.67 ± 21.22 | 0.33 ± 0.33 | 0.33 ± 0.33 | 12.67 ± 12.17 | 0.67 ± 0.67 | 22.67 ± 14.62 | 2 ± 2 | 0 ± 0 |
| Distoseptispora | 0 ± 0 | 0 ± 0 | 0 ± 0 | 0 ± 0 | 0 ± 0 | 0 ± 0 | 0 ± 0 | 0 ± 0 | 0 ± 0 | 0.33 ± 0.33 |
| Diversispora | 0 ± 0 | 0.67 ± 0.67 | 0 ± 0 | 0 ± 0 | 0 ± 0 | 0 ± 0 | 0.33 ± 0.33 | 0 ± 0 | 0 ± 0 | 0 ± 0 |
| Dokmaia | 2 ± 1 | 0 ± 0 | 6 ± 1.53 | 0 ± 0 | 0 ± 0 | 4.67 ± 4.67 | 0 ± 0 | 2.33 ± 1.86 | 0 ± 0 | 0.33 ± 0.33 |
| Dominikia | 1 ± 0.58 | 0 ± 0 | 0 ± 0 | 7.67 ± 3.93 | 8.33 ± 2.85 | 0 ± 0 | 1.67 ± 0.33 | 8.67 ± 7.69 | 33.67 ± 21.26 | 40 ± 13.86 |
| Donkioporia | 0.33 ± 0.33 | 13.33 ± 4.41 | 0.33 ± 0.33 | 0 ± 0 | 0 ± 0 | 0 ± 0 | 1.33 ± 0.88 | 0 ± 0 | 0 ± 0 | 0 ± 0 |
| Doratomyces | 0 ± 0 | 0 ± 0 | 22.33 ± 7.69 | 2 ± 1.53 | 0 ± 0 | 12.67 ± 2.67 | 95.67 ± 90.17 | 2.33 ± 0.33 | 1.67 ± 0.88 | 3.33 ± 2.4 |
| Dothiorella | 0.67 ± 0.67 | 0 ± 0 | 0 ± 0 | 0.67 ± 0.67 | 34 ± 2.89 | 4.67 ± 1.45 | 1.33 ± 0.88 | 4 ± 3.06 | 1.67 ± 1.67 | 0 ± 0 |
| Drechslera | 0 ± 0 | 0.67 ± 0.67 | 0 ± 0 | 0 ± 0 | 0 ± 0 | 0 ± 0 | 0.33 ± 0.33 | 0 ± 0 | 0 ± 0 | 0 ± 0 |
| Drechslerella | 0 ± 0 | 0 ± 0 | 11 ± 11 | 0.33 ± 0.33 | 0 ± 0 | 7 ± 5.13 | 1.33 ± 1.33 | 30 ± 19.29 | 2.33 ± 2.33 | 11.33 ± 7.88 |
| Ectophoma | 0 ± 0 | 0 ± 0 | 0 ± 0 | 0 ± 0 | 0 ± 0 | 0 ± 0 | 0 ± 0 | 0.33 ± 0.33 | 0 ± 0 | 0 ± 0 |
| Efibula | 0 ± 0 | 12.67 ± 2.91 | 0 ± 0 | 0 ± 0 | 0 ± 0 | 0 ± 0 | 0.67 ± 0.67 | 0 ± 0 | 0 ± 0 | 0 ± 0 |
| Eichleriella | 0 ± 0 | 0.33 ± 0.33 | 0 ± 0 | 6.67 ± 0.88 | 0.67 ± 0.67 | 0 ± 0 | 2.67 ± 1.76 | 0 ± 0 | 0 ± 0 | 0 ± 0 |
| Elaphomyces | 0 ± 0 | 0 ± 0 | 0 ± 0 | 0.67 ± 0.67 | 0 ± 0 | 0 ± 0 | 0.67 ± 0.67 | 0 ± 0 | 0 ± 0 | 0 ± 0 |
| Elsinoe | 3 ± 2.08 | 0 ± 0 | 0 ± 0 | 0 ± 0 | 0.67 ± 0.67 | 0.67 ± 0.67 | 0 ± 0 | 0.33 ± 0.33 | 0 ± 0 | 3.67 ± 3.67 |
| Emericellopsis | 0 ± 0 | 0.33 ± 0.33 | 15.33 ± 8.41 | 0 ± 0 | 0.33 ± 0.33 | 2.67 ± 1.76 | 2.33 ± 1.45 | 8.33 ± 1.67 | 5.33 ± 4.84 | 4.33 ± 3.84 |
| Encoelia | 0 ± 0 | 0 ± 0 | 0 ± 0 | 0 ± 0 | 0 ± 0 | 0 ± 0 | 0 ± 0 | 0 ± 0 | 0 ± 0 | 1.33 ± 1.33 |
| Endosporium | 0 ± 0 | 0 ± 0 | 0 ± 0 | 0.33 ± 0.33 | 0 ± 0 | 0 ± 0 | 0 ± 0 | 0 ± 0 | 0.67 ± 0.67 | 3.67 ± 2.33 |
| Entoloma | 11 ± 11 | 8.33 ± 3.28 | 3.33 ± 2.85 | 1.67 ± 1.2 | 3.33 ± 2.85 | 6.67 ± 1.2 | 13.33 ± 9.84 | 2.67 ± 0.88 | 1.67 ± 0.88 | 1.33 ± 1.33 |
| Entorrhiza | 0 ± 0 | 54 ± 30.89 | 0 ± 0 | 2.67 ± 2.19 | 0 ± 0 | 0.33 ± 0.33 | 6.33 ± 6.33 | 0 ± 0 | 0 ± 0 | 0 ± 0 |
| Entosordaria | 0 ± 0 | 0 ± 0 | 0 ± 0 | 0 ± 0 | 0 ± 0 | 1 ± 1 | 0 ± 0 | 0 ± 0 | 0 ± 0 | 0 ± 0 |
| Entrophospora | 25 ± 6.03 | 170.33 ± 62.03 | 40.33 ± 14.77 | 219.33 ± 46.84 | 53 ± 19.63 | 56 ± 12.42 | 128.33 ± 55.71 | 117 ± 34.02 | 77 ± 2.65 | 172.33 ± 37.12 |
| Eocronartium | 0.33 ± 0.33 | 0.67 ± 0.33 | 0 ± 0 | 5.67 ± 2.4 | 13 ± 3.61 | 0 ± 0 | 2 ± 2 | 89 ± 49.74 | 120 ± 119 | 0.67 ± 0.33 |
| Epibryon | 0 ± 0 | 0.33 ± 0.33 | 0 ± 0 | 0 ± 0 | 0 ± 0 | 0 ± 0 | 0.33 ± 0.33 | 0.67 ± 0.67 | 3.33 ± 2.85 | 0 ± 0 |
| Epichloe | 0 ± 0 | 0 ± 0 | 0 ± 0 | 0 ± 0 | 0 ± 0 | 0 ± 0 | 0 ± 0 | 0 ± 0 | 0 ± 0 | 0 ± 0 |
| Epicoccum | 24.33 ± 13.86 | 159.67 ± 67.85 | 109.67 ± 25.85 | 30.33 ± 5.24 | 41 ± 2.65 | 45.33 ± 33.86 | 55.67 ± 16.97 | 84.67 ± 20.58 | 14.33 ± 2.96 | 211.33 ± 36.67 |
| Epicoleosporium | 0 ± 0 | 0 ± 0 | 81 ± 23.12 | 1.33 ± 1.33 | 0 ± 0 | 20.33 ± 16.9 | 10.33 ± 5.84 | 0 ± 0 | 0 ± 0 | 2.33 ± 1.86 |
| Erythrobasidium | 0 ± 0 | 0 ± 0 | 0.33 ± 0.33 | 0 ± 0 | 0.33 ± 0.33 | 0.67 ± 0.67 | 1 ± 1 | 2.67 ± 1.76 | 0 ± 0 | 3.67 ± 2.03 |
| Escovopsioides | 4.67 ± 2.91 | 0.33 ± 0.33 | 0.33 ± 0.33 | 0 ± 0 | 1 ± 0.58 | 0 ± 0 | 0.33 ± 0.33 | 0 ± 0 | 0 ± 0 | 0 ± 0 |
| Escovopsis | 0 ± 0 | 0 ± 0 | 0 ± 0 | 0 ± 0 | 0.67 ± 0.67 | 2.33 ± 1.86 | 0 ± 0 | 0 ± 0 | 0 ± 0 | 0 ± 0 |
| Eucasphaeria | 0 ± 0 | 0 ± 0 | 0 ± 0 | 0.67 ± 0.33 | 0 ± 0 | 0 ± 0 | 2 ± 1.15 | 2 ± 2 | 0.33 ± 0.33 | 5.33 ± 4.84 |
| Eutypella | 0 ± 0 | 0 ± 0 | 1.33 ± 0.88 | 0.67 ± 0.33 | 0.33 ± 0.33 | 8 ± 4.16 | 2 ± 1.53 | 0 ± 0 | 0 ± 0 | 1.67 ± 0.88 |
| Exidia | 0 ± 0 | 2.33 ± 1.2 | 0 ± 0 | 0 ± 0 | 1.33 ± 1.33 | 0 ± 0 | 0.67 ± 0.33 | 0 ± 0 | 0.33 ± 0.33 | 3 ± 3 |
| Exobasidium | 0 ± 0 | 0 ± 0 | 0 ± 0 | 0 ± 0 | 0 ± 0 | 0 ± 0 | 0 ± 0 | 0.67 ± 0.67 | 0 ± 0 | 0 ± 0 |
| Exophiala | 48.67 ± 4.26 | 76.33 ± 24.13 | 358.33 ± 121.07 | 58 ± 30.27 | 73 ± 38.97 | 188.33 ± 32.42 | 134 ± 43.49 | 1134.33 ± 712.23 | 832.67 ± 804.67 | 54 ± 17.04 |
| Farysia | 0 ± 0 | 0 ± 0 | 0 ± 0 | 0 ± 0 | 0 ± 0 | 0 ± 0 | 0 ± 0 | 1.67 ± 1.67 | 0.33 ± 0.33 | 0 ± 0 |
| Fellozyma | 0 ± 0 | 0 ± 0 | 0.33 ± 0.33 | 0 ± 0 | 0 ± 0 | 0.67 ± 0.67 | 0 ± 0 | 0 ± 0 | 0 ± 0 | 0 ± 0 |
| Fibroporia | 0 ± 0 | 0 ± 0 | 0 ± 0 | 1 ± 0.58 | 0.33 ± 0.33 | 0 ± 0 | 1.67 ± 1.2 | 0 ± 0 | 0 ± 0 | 1.67 ± 1.67 |
| Fibulobasidium | 0.33 ± 0.33 | 0.33 ± 0.33 | 0 ± 0 | 0 ± 0 | 0 ± 0 | 0.67 ± 0.67 | 0 ± 0 | 0 ± 0 | 0 ± 0 | 0 ± 0 |
| Fibulochlamys | 0 ± 0 | 0.67 ± 0.67 | 0 ± 0 | 0.67 ± 0.67 | 0 ± 0 | 0 ± 0 | 0 ± 0 | 0 ± 0 | 1 ± 0.58 | 0 ± 0 |
| Fibulomyces | 0 ± 0 | 0 ± 0 | 0 ± 0 | 0 ± 0 | 0 ± 0 | 0 ± 0 | 0 ± 0 | 0.33 ± 0.33 | 1 ± 0 | 23 ± 19.04 |
| Filobasidium | 0.67 ± 0.33 | 0 ± 0 | 0.67 ± 0.67 | 0.33 ± 0.33 | 0 ± 0 | 5.33 ± 2.67 | 0.67 ± 0.33 | 0.33 ± 0.33 | 1.33 ± 0.88 | 1.33 ± 1.33 |
| Fimetariella | 0 ± 0 | 0 ± 0 | 3.67 ± 3.67 | 0 ± 0 | 0 ± 0 | 0 ± 0 | 1.33 ± 0.88 | 0 ± 0 | 0 ± 0 | 0 ± 0 |
| Flabellascoma | 0 ± 0 | 0 ± 0 | 0 ± 0 | 0.33 ± 0.33 | 0 ± 0 | 0.33 ± 0.33 | 3 ± 1.73 | 5.67 ± 5.67 | 0.33 ± 0.33 | 1 ± 1 |
| Flagellospora | 1.67 ± 1.67 | 0 ± 0 | 0 ± 0 | 0 ± 0 | 0 ± 0 | 0 ± 0 | 0 ± 0 | 0 ± 0 | 0 ± 0 | 0 ± 0 |
| Fonsecazyma | 0 ± 0 | 0 ± 0 | 0 ± 0 | 0 ± 0 | 0 ± 0 | 0 ± 0 | 0 ± 0 | 0 ± 0 | 0 ± 0 | 0 ± 0 |
| Funneliformis | 1.67 ± 0.33 | 27.33 ± 3.84 | 0 ± 0 | 0.33 ± 0.33 | 1.67 ± 0.88 | 0 ± 0 | 2.67 ± 1.45 | 0 ± 0 | 0 ± 0 | 1 ± 1 |
| Fusariella | 0 ± 0 | 0 ± 0 | 7 ± 2 | 0 ± 0 | 0 ± 0 | 3.33 ± 2.03 | 0.33 ± 0.33 | 2.33 ± 2.33 | 0.33 ± 0.33 | 1 ± 1 |
| Fusarium | 491.67 ± 96.07 | 7093.67 ± 1573.51 | 626.33 ± 90.89 | 852.33 ± 238.25 | 434 ± 146.53 | 3643 ± 1395.64 | 2530 ± 889.88 | 1749.67 ± 803.87 | 570.67 ± 322.67 | 2943.33 ± 600.77 |
| Fusicolla | 3 ± 1.53 | 0.67 ± 0.67 | 64.33 ± 11.85 | 3.67 ± 1.2 | 8.33 ± 1.2 | 257.33 ± 124.86 | 10.67 ± 2.03 | 18.33 ± 9.33 | 1.33 ± 0.88 | 7.33 ± 5.04 |
| Fusiconidium | 0 ± 0 | 0 ± 0 | 0 ± 0 | 0 ± 0 | 0 ± 0 | 0 ± 0 | 0 ± 0 | 0 ± 0 | 0 ± 0 | 3.67 ± 0.67 |
| Fusidium | 6.33 ± 2.33 | 15.33 ± 11.57 | 33 ± 21.01 | 5.33 ± 3.84 | 14.67 ± 3.28 | 304.33 ± 143.91 | 14.33 ± 4.41 | 98 ± 73.98 | 38.33 ± 37.34 | 34.67 ± 12.14 |
| Gaeumannomycella | 0 ± 0 | 0 ± 0 | 0.33 ± 0.33 | 0 ± 0 | 0 ± 0 | 0 ± 0 | 0.33 ± 0.33 | 0 ± 0 | 0 ± 0 | 0 ± 0 |
| Gaeumannomyces | 0 ± 0 | 0 ± 0 | 0 ± 0 | 0 ± 0 | 0 ± 0 | 0 ± 0 | 0.33 ± 0.33 | 0 ± 0 | 0 ± 0 | 0 ± 0 |
| Gamsia | 0.33 ± 0.33 | 0 ± 0 | 0.33 ± 0.33 | 0.33 ± 0.33 | 0.33 ± 0.33 | 1 ± 0.58 | 4.67 ± 4.18 | 0.33 ± 0.33 | 1 ± 0.58 | 1.33 ± 0.88 |
| Gamsylella | 0 ± 0 | 0 ± 0 | 0 ± 0 | 0 ± 0 | 0 ± 0 | 0.67 ± 0.67 | 0 ± 0 | 0 ± 0 | 0 ± 0 | 0 ± 0 |
| Ganoderma | 39.33 ± 5.93 | 0.67 ± 0.33 | 26.33 ± 9.94 | 11.33 ± 6.36 | 17 ± 3.21 | 13 ± 4.04 | 33 ± 15.95 | 34.33 ± 16.22 | 14.33 ± 11.39 | 4.67 ± 4.18 |
| Geastrumia | 0 ± 0 | 0 ± 0 | 0 ± 0 | 0 ± 0 | 0 ± 0 | 0 ± 0 | 0 ± 0 | 0 ± 0 | 0 ± 0 | 1 ± 1 |
| Gelasinospora | 0.67 ± 0.67 | 0 ± 0 | 0 ± 0 | 0 ± 0 | 0 ± 0 | 0 ± 0 | 0 ± 0 | 0 ± 0 | 0 ± 0 | 0 ± 0 |
| Geminibasidium | 0.67 ± 0.67 | 4.33 ± 1.2 | 765.67 ± 183.51 | 3.67 ± 0.88 | 18 ± 6.51 | 270.33 ± 201.88 | 10 ± 4.16 | 0 ± 0 | 0 ± 0 | 7.33 ± 3.53 |
| Genolevuria | 0 ± 0 | 0 ± 0 | 0 ± 0 | 1 ± 1 | 0 ± 0 | 0 ± 0 | 0 ± 0 | 0 ± 0 | 0 ± 0 | 0.33 ± 0.33 |
| Geoglossum | 24 ± 19.08 | 0.33 ± 0.33 | 4 ± 4 | 0.33 ± 0.33 | 2.67 ± 1.76 | 0 ± 0 | 31 ± 30.01 | 10 ± 10 | 5.33 ± 3.33 | 0 ± 0 |
| Gibellulopsis | 1.67 ± 0.67 | 38.67 ± 24.46 | 13.33 ± 11.33 | 12.67 ± 5.67 | 12.67 ± 3.48 | 80 ± 39.37 | 19 ± 4.36 | 2.67 ± 1.76 | 7.33 ± 7.33 | 72 ± 40.81 |
| Gliocladiopsis | 0 ± 0 | 0 ± 0 | 25 ± 9.71 | 0 ± 0 | 0.33 ± 0.33 | 13.67 ± 12.2 | 3 ± 2.52 | 0 ± 0 | 0 ± 0 | 0 ± 0 |
| Gliocladium | 0 ± 0 | 0 ± 0 | 1 ± 1 | 0.67 ± 0.33 | 0 ± 0 | 4.67 ± 0.88 | 2.33 ± 0.67 | 0 ± 0 | 0 ± 0 | 0.67 ± 0.67 |
| Gliomastix | 1.33 ± 0.67 | 0 ± 0 | 13.33 ± 5.81 | 2 ± 0.58 | 14.33 ± 2.19 | 20.33 ± 8.95 | 20 ± 17.5 | 43 ± 9.85 | 7.33 ± 3.18 | 10.67 ± 3.67 |
| Gloeoporus | 0 ± 0 | 0 ± 0 | 0 ± 0 | 0 ± 0 | 0 ± 0 | 0 ± 0 | 0.67 ± 0.67 | 0 ± 0 | 0 ± 0 | 0 ± 0 |
| Gloeosporium | 0 ± 0 | 0 ± 0 | 0 ± 0 | 1 ± 1 | 0 ± 0 | 0.33 ± 0.33 | 0 ± 0 | 0 ± 0 | 0 ± 0 | 0 ± 0 |
| Glomerella | 0 ± 0 | 0 ± 0 | 1 ± 1 | 0 ± 0 | 0 ± 0 | 1.33 ± 0.88 | 0.33 ± 0.33 | 0 ± 0 | 0.67 ± 0.67 | 1 ± 1 |
| Glomus | 19.67 ± 5.81 | 157 ± 38.08 | 1 ± 0.58 | 52 ± 16.56 | 61.33 ± 18.28 | 9 ± 3.06 | 38.67 ± 17.7 | 39.33 ± 8.33 | 46.33 ± 14.45 | 118 ± 38.08 |
| Glutinoglossum | 0 ± 0 | 0 ± 0 | 0 ± 0 | 0 ± 0 | 0 ± 0 | 0 ± 0 | 0 ± 0 | 5 ± 5 | 0.67 ± 0.33 | 0 ± 0 |
| Glutinomyces | 0 ± 0 | 59.33 ± 28.59 | 58.67 ± 38.19 | 16.33 ± 10.4 | 0 ± 0 | 25.33 ± 21.84 | 16.33 ± 13.35 | 197.33 ± 180.38 | 16.33 ± 4.33 | 182.67 ± 55.17 |
| Golovinomyces | 9.67 ± 4.18 | 0.67 ± 0.33 | 24 ± 11.68 | 9.67 ± 6.89 | 0.33 ± 0.33 | 16.67 ± 5.81 | 13.67 ± 6.06 | 45.67 ± 25.41 | 9.33 ± 9.33 | 17.67 ± 4.67 |
| Gonatophragmium | 0 ± 0 | 0 ± 0 | 0 ± 0 | 0 ± 0 | 2 ± 2 | 0.67 ± 0.67 | 0 ± 0 | 0 ± 0 | 0 ± 0 | 0 ± 0 |
| Gongronella | 0 ± 0 | 0 ± 0 | 0 ± 0 | 0 ± 0 | 0 ± 0 | 0 ± 0 | 0 ± 0 | 0 ± 0 | 0 ± 0 | 6 ± 6 |
| Gorgomyces | 0 ± 0 | 0 ± 0 | 5 ± 2.65 | 0.33 ± 0.33 | 0 ± 0 | 0 ± 0 | 0 ± 0 | 0.33 ± 0.33 | 1 ± 1 | 0 ± 0 |
| Graphium | 0 ± 0 | 1 ± 0.58 | 0.33 ± 0.33 | 1.67 ± 1.2 | 4.33 ± 2.33 | 14.67 ± 4.18 | 10 ± 4.16 | 0 ± 0 | 0 ± 0 | 0.33 ± 0.33 |
| Groenewaldozyma | 0 ± 0 | 0 ± 0 | 0 ± 0 | 0 ± 0 | 0 ± 0 | 1.33 ± 0.88 | 1.33 ± 1.33 | 0 ± 0 | 0 ± 0 | 0 ± 0 |
| Grubyella | 0 ± 0 | 0.67 ± 0.33 | 1.33 ± 0.88 | 0 ± 0 | 0 ± 0 | 0 ± 0 | 1 ± 0.58 | 9.33 ± 9.33 | 2.67 ± 2.19 | 1 ± 0 |
| Gymnoascus | 0 ± 0 | 0 ± 0 | 0 ± 0 | 0 ± 0 | 0.67 ± 0.67 | 0 ± 0 | 0 ± 0 | 0 ± 0 | 0 ± 0 | 0 ± 0 |
| Gyoerffyella | 0 ± 0 | 2 ± 1.15 | 0 ± 0 | 0.67 ± 0.33 | 0 ± 0 | 0.33 ± 0.33 | 1.67 ± 1.67 | 0 ± 0 | 0 ± 0 | 0 ± 0 |
| Hamamotoa | 0.33 ± 0.33 | 0 ± 0 | 0 ± 0 | 1 ± 1 | 0 ± 0 | 0 ± 0 | 0 ± 0 | 0 ± 0 | 0 ± 0 | 0 ± 0 |
| Hamatocanthoscypha | 0 ± 0 | 0 ± 0 | 0 ± 0 | 0.33 ± 0.33 | 0 ± 0 | 0 ± 0 | 0 ± 0 | 0 ± 0 | 0 ± 0 | 0 ± 0 |
| Hannaella | 8.33 ± 5.36 | 21.67 ± 4.18 | 158.33 ± 113.86 | 11 ± 1.15 | 46.67 ± 24.21 | 23 ± 14.73 | 34.33 ± 9.61 | 1.33 ± 1.33 | 1 ± 0 | 98.33 ± 32.2 |
| Haradamyces | 0 ± 0 | 0 ± 0 | 0 ± 0 | 0 ± 0 | 0 ± 0 | 0 ± 0 | 0 ± 0 | 0.33 ± 0.33 | 0 ± 0 | 4.33 ± 1.86 |
| Harposporium | 0 ± 0 | 0.67 ± 0.67 | 0 ± 0 | 0 ± 0 | 0 ± 0 | 0 ± 0 | 1 ± 1 | 0 ± 0 | 0 ± 0 | 0 ± 0 |
| Hebeloma | 0 ± 0 | 0 ± 0 | 1.33 ± 0.88 | 0 ± 0 | 0 ± 0 | 0 ± 0 | 0.33 ± 0.33 | 0 ± 0 | 0.67 ± 0.67 | 0 ± 0 |
| Helicodendron | 0 ± 0 | 0 ± 0 | 0 ± 0 | 0 ± 0 | 0 ± 0 | 0 ± 0 | 0 ± 0 | 0 ± 0 | 0 ± 0 | 2 ± 1.53 |
| Helicoubisia | 0 ± 0 | 10.67 ± 2.96 | 0 ± 0 | 0.67 ± 0.33 | 0 ± 0 | 0 ± 0 | 1.33 ± 0.88 | 0 ± 0 | 0.33 ± 0.33 | 0 ± 0 |
| Helminthosporium | 0 ± 0 | 0 ± 0 | 0 ± 0 | 0 ± 0 | 0.33 ± 0.33 | 0 ± 0 | 0 ± 0 | 0 ± 0 | 0 ± 0 | 1 ± 1 |
| Hemileucoglossum | 0 ± 0 | 0 ± 0 | 0 ± 0 | 0 ± 0 | 0 ± 0 | 0.33 ± 0.33 | 0.33 ± 0.33 | 78.67 ± 77.17 | 402.33 ± 349.73 | 1.33 ± 0.67 |
| Hermatomyces | 0 ± 0 | 0 ± 0 | 0 ± 0 | 0 ± 0 | 0 ± 0 | 0 ± 0 | 0 ± 0 | 0 ± 0 | 0 ± 0 | 5.33 ± 2.91 |
| Herpotrichia | 0.67 ± 0.67 | 0 ± 0 | 1 ± 1 | 0 ± 0 | 1.67 ± 0.88 | 1.33 ± 1.33 | 0 ± 0 | 0 ± 0 | 0 ± 0 | 0 ± 0 |
| Heterochaete | 0 ± 0 | 0 ± 0 | 0 ± 0 | 0 ± 0 | 0 ± 0 | 2 ± 2 | 0.33 ± 0.33 | 0 ± 0 | 0 ± 0 | 2.33 ± 1.86 |
| Heterosphaeria | 0 ± 0 | 0 ± 0 | 0 ± 0 | 0 ± 0 | 0 ± 0 | 0.33 ± 0.33 | 0.67 ± 0.67 | 0 ± 0 | 0 ± 0 | 0 ± 0 |
| Hirsutella | 0 ± 0 | 1 ± 1 | 2 ± 0.58 | 1 ± 0.58 | 0.33 ± 0.33 | 7.33 ± 4.48 | 1.33 ± 0.88 | 1 ± 1 | 2 ± 1.53 | 4.67 ± 4.67 |
| Hobus | 0 ± 0 | 0 ± 0 | 0.33 ± 0.33 | 0 ± 0 | 0 ± 0 | 0 ± 0 | 0 ± 0 | 0 ± 0 | 0 ± 0 | 0 ± 0 |
| Hodophilus | 1.33 ± 0.67 | 2 ± 1.53 | 0 ± 0 | 0 ± 0 | 0 ± 0 | 0 ± 0 | 0.67 ± 0.67 | 0 ± 0 | 0 ± 0 | 0 ± 0 |
| Holocotylon | 0 ± 0 | 0 ± 0 | 0 ± 0 | 0.67 ± 0.67 | 0 ± 0 | 0 ± 0 | 0 ± 0 | 0 ± 0 | 0 ± 0 | 0 ± 0 |
| Hormiactis | 0.67 ± 0.67 | 0 ± 0 | 0 ± 0 | 0 ± 0 | 5.67 ± 3.18 | 4.67 ± 2.6 | 3 ± 2.52 | 0.33 ± 0.33 | 0.33 ± 0.33 | 0.33 ± 0.33 |
| Humicola | 16.67 ± 2.33 | 3094.33 ± 728.64 | 58 ± 8.08 | 166.67 ± 49.83 | 31 ± 14.5 | 198.33 ± 37.21 | 973.67 ± 189.02 | 165.67 ± 77.8 | 20 ± 2.65 | 160.33 ± 12.98 |
| Humicolopsis | 0 ± 0 | 0 ± 0 | 0 ± 0 | 0 ± 0 | 0 ± 0 | 1 ± 1 | 0 ± 0 | 0 ± 0 | 0 ± 0 | 0 ± 0 |
| Hyalorbilia | 4.67 ± 3.71 | 74.67 ± 22.06 | 3.33 ± 1.33 | 7.33 ± 2.33 | 5.33 ± 2.19 | 25 ± 11.59 | 26 ± 4.73 | 31.33 ± 26.83 | 17 ± 9.29 | 22.67 ± 12.33 |
| Hyaloscypha | 0 ± 0 | 0.33 ± 0.33 | 0 ± 0 | 0 ± 0 | 0 ± 0 | 0 ± 0 | 0 ± 0 | 0 ± 0 | 0 ± 0 | 0.33 ± 0.33 |
| Hydropisphaera | 0 ± 0 | 0 ± 0 | 3.67 ± 1.86 | 0 ± 0 | 0 ± 0 | 0.33 ± 0.33 | 0 ± 0 | 72.33 ± 66.37 | 15 ± 14.01 | 0.67 ± 0.33 |
| Hygrocybe | 265.33 ± 18.98 | 2 ± 1 | 3.33 ± 0.88 | 0.33 ± 0.33 | 16 ± 0.58 | 5.33 ± 2.67 | 4.33 ± 2.4 | 0.33 ± 0.33 | 0 ± 0 | 2.67 ± 2.67 |
| Hymenogaster | 0 ± 0 | 0 ± 0 | 1 ± 0.58 | 4.33 ± 3.38 | 0 ± 0 | 11 ± 4.58 | 22 ± 14.01 | 6.33 ± 2.19 | 1.67 ± 0.67 | 2 ± 2 |
| Hymenoscyphus | 0 ± 0 | 0 ± 0 | 0.67 ± 0.67 | 2.67 ± 1.76 | 0 ± 0 | 84.67 ± 72.81 | 1.67 ± 0.88 | 0 ± 0 | 0 ± 0 | 1 ± 0.58 |
| Hyphodermella | 0 ± 0 | 0 ± 0 | 0.67 ± 0.67 | 0.33 ± 0.33 | 0 ± 0 | 0 ± 0 | 0 ± 0 | 0 ± 0 | 0 ± 0 | 0 ± 0 |
| Hyphodontia | 0 ± 0 | 2 ± 1.15 | 2 ± 1.53 | 38 ± 27.06 | 0 ± 0 | 3.67 ± 0.88 | 17.67 ± 11.41 | 0 ± 0 | 0 ± 0 | 0 ± 0 |
| Hypholoma | 0 ± 0 | 0 ± 0 | 0 ± 0 | 0 ± 0 | 0 ± 0 | 0.67 ± 0.67 | 0 ± 0 | 5.33 ± 2.91 | 0 ± 0 | 0 ± 0 |
| Hypochnicium | 0 ± 0 | 0 ± 0 | 0.33 ± 0.33 | 0 ± 0 | 0 ± 0 | 0 ± 0 | 0 ± 0 | 0 ± 0 | 0 ± 0 | 0 ± 0 |
| Hypocreopsis | 0 ± 0 | 0 ± 0 | 0 ± 0 | 0 ± 0 | 0 ± 0 | 8.67 ± 5.21 | 7 ± 6.51 | 0.67 ± 0.67 | 0 ± 0 | 0 ± 0 |
| Hypomyces | 3.67 ± 1.67 | 0 ± 0 | 13 ± 2.31 | 1.67 ± 0.33 | 13 ± 12 | 26 ± 3.21 | 8.33 ± 2.33 | 1 ± 1 | 3.67 ± 2.73 | 1.67 ± 1.67 |
| Hypoxylon | 0 ± 0 | 0 ± 0 | 0 ± 0 | 0 ± 0 | 1 ± 1 | 0 ± 0 | 0 ± 0 | 0 ± 0 | 0 ± 0 | 1.67 ± 1.67 |
| Idriella | 1.33 ± 0.88 | 0 ± 0 | 48.33 ± 23.15 | 4 ± 1.53 | 22.67 ± 2.03 | 104.33 ± 40.55 | 36.67 ± 24.73 | 73.67 ± 39.77 | 32.67 ± 32.17 | 20 ± 5.03 |
| Ijuhya | 10.67 ± 3.53 | 0.33 ± 0.33 | 2.67 ± 0.33 | 0 ± 0 | 8.67 ± 1.2 | 2 ± 1 | 0.33 ± 0.33 | 2 ± 2 | 1.67 ± 1.67 | 0 ± 0 |
| Ilyonectria | 43.67 ± 27.29 | 1 ± 0 | 198 ± 74.22 | 24.67 ± 7.22 | 110.67 ± 27.71 | 213.67 ± 90.27 | 157.67 ± 117.27 | 58 ± 23.67 | 50.33 ± 23.85 | 55.33 ± 31.14 |
| Immersidiscosia | 0 ± 0 | 0 ± 0 | 2.67 ± 2.67 | 0 ± 0 | 0.33 ± 0.33 | 1.33 ± 1.33 | 0 ± 0 | 0 ± 0 | 0 ± 0 | 0 ± 0 |
| Incrucipulum | 0 ± 0 | 0 ± 0 | 0 ± 0 | 0.67 ± 0.33 | 0 ± 0 | 0 ± 0 | 0.67 ± 0.33 | 0 ± 0 | 0.33 ± 0.33 | 1.33 ± 1.33 |
| Infundichalara | 0 ± 0 | 0 ± 0 | 0 ± 0 | 0 ± 0 | 0 ± 0 | 0 ± 0 | 0 ± 0 | 6.67 ± 3.76 | 8.33 ± 8.33 | 0.33 ± 0.33 |
| Inocybe | 2.33 ± 2.33 | 0.33 ± 0.33 | 57.67 ± 22.64 | 0 ± 0 | 0 ± 0 | 9 ± 1.73 | 2 ± 1 | 0.67 ± 0.67 | 0.33 ± 0.33 | 12.33 ± 9.4 |
| Isaria | 0 ± 0 | 0 ± 0 | 2 ± 1 | 0.67 ± 0.67 | 0 ± 0 | 0.67 ± 0.33 | 0 ± 0 | 0 ± 0 | 0 ± 0 | 0 ± 0 |
| Ischnoderma | 0 ± 0 | 0 ± 0 | 2.67 ± 2.19 | 0 ± 0 | 0 ± 0 | 0 ± 0 | 0 ± 0 | 0 ± 0 | 0 ± 0 | 0 ± 0 |
| Jattaea | 0 ± 0 | 2 ± 2 | 0.33 ± 0.33 | 0 ± 0 | 0.67 ± 0.67 | 3.67 ± 3.67 | 1 ± 1 | 0.33 ± 0.33 | 0 ± 0 | 474 ± 474 |
| Jugulospora | 0 ± 0 | 0 ± 0 | 0 ± 0 | 0 ± 0 | 0 ± 0 | 0 ± 0 | 1.67 ± 1.67 | 0 ± 0 | 0 ± 0 | 0 ± 0 |
| Junewangia | 5.67 ± 3.48 | 116 ± 47.26 | 0 ± 0 | 10.33 ± 2.03 | 1.67 ± 1.2 | 1 ± 1 | 15.33 ± 7.33 | 2 ± 1 | 7.67 ± 6.17 | 9.33 ± 5.81 |
| Juxtiphoma | 0.67 ± 0.67 | 0 ± 0 | 0 ± 0 | 0.33 ± 0.33 | 12.33 ± 1.2 | 2.33 ± 2.33 | 1.33 ± 0.67 | 0.67 ± 0.67 | 0 ± 0 | 11 ± 9.07 |
| Karstenula | 0 ± 0 | 0 ± 0 | 0 ± 0 | 0 ± 0 | 0 ± 0 | 0 ± 0 | 1 ± 1 | 0 ± 0 | 0 ± 0 | 0 ± 0 |
| Kavinia | 0 ± 0 | 0 ± 0 | 0 ± 0 | 0 ± 0 | 0 ± 0 | 0 ± 0 | 0 ± 0 | 1.67 ± 1.2 | 4 ± 3.51 | 53 ± 53 |
| Keratinophyton | 0 ± 0 | 0 ± 0 | 0 ± 0 | 0 ± 0 | 0 ± 0 | 0.33 ± 0.33 | 0 ± 0 | 0 ± 0 | 2 ± 1.53 | 9 ± 1 |
| Kiflimonium | 0 ± 0 | 4.67 ± 2.6 | 0 ± 0 | 0 ± 0 | 0 ± 0 | 0.33 ± 0.33 | 0.33 ± 0.33 | 1.67 ± 0.88 | 0.67 ± 0.67 | 30.33 ± 7.26 |
| Knufia | 4 ± 1 | 627 ± 185.15 | 2 ± 1.53 | 35 ± 15.53 | 0.67 ± 0.67 | 2.67 ± 0.33 | 114 ± 45.08 | 76.33 ± 22.36 | 83 ± 55.24 | 1616.33 ± 783.76 |
| Kockovaella | 0 ± 0 | 0 ± 0 | 0 ± 0 | 0.33 ± 0.33 | 0 ± 0 | 0 ± 0 | 0 ± 0 | 1.33 ± 0.88 | 0 ± 0 | 0.67 ± 0.67 |
| Kondoa | 0 ± 0 | 0 ± 0 | 0.33 ± 0.33 | 0 ± 0 | 0 ± 0 | 0 ± 0 | 0 ± 0 | 0.33 ± 0.33 | 0 ± 0 | 4 ± 0.58 |
| Koorchaloma | 0 ± 0 | 7.67 ± 6.23 | 0 ± 0 | 0.33 ± 0.33 | 0 ± 0 | 0 ± 0 | 0.67 ± 0.67 | 0 ± 0 | 0 ± 0 | 0 ± 0 |
| Kretzschmaria | 0 ± 0 | 0 ± 0 | 0 ± 0 | 0 ± 0 | 0 ± 0 | 0 ± 0 | 1 ± 1 | 3.33 ± 3.33 | 0 ± 0 | 0 ± 0 |
| Kurtzmanomyces | 1.67 ± 1.67 | 0 ± 0 | 0.33 ± 0.33 | 1 ± 0.58 | 0.33 ± 0.33 | 1.33 ± 0.88 | 1.33 ± 1.33 | 8 ± 6.11 | 0 ± 0 | 0 ± 0 |
| Lachnellula | 0.33 ± 0.33 | 0 ± 0 | 25 ± 15.01 | 0 ± 0 | 0.67 ± 0.67 | 11.67 ± 10.68 | 0.33 ± 0.33 | 0 ± 0 | 0 ± 0 | 0 ± 0 |
| Lachnum | 0 ± 0 | 1 ± 0.58 | 0 ± 0 | 0 ± 0 | 1.67 ± 1.67 | 0 ± 0 | 0.33 ± 0.33 | 0 ± 0 | 1 ± 1 | 0 ± 0 |
| Lactarius | 0 ± 0 | 0 ± 0 | 2 ± 2 | 0 ± 0 | 0 ± 0 | 0 ± 0 | 0 ± 0 | 1.67 ± 1.67 | 0 ± 0 | 0 ± 0 |
| Laetisaria | 0 ± 0 | 0 ± 0 | 0 ± 0 | 0 ± 0 | 0 ± 0 | 0 ± 0 | 0 ± 0 | 5 ± 4.51 | 15 ± 15 | 0 ± 0 |
| Lamprospora | 0 ± 0 | 0.67 ± 0.67 | 0 ± 0 | 0 ± 0 | 0 ± 0 | 0 ± 0 | 0 ± 0 | 0 ± 0 | 0 ± 0 | 0 ± 0 |
| Lanzia | 0.33 ± 0.33 | 0 ± 0 | 0 ± 0 | 0 ± 0 | 4.33 ± 1.86 | 1.33 ± 1.33 | 0.33 ± 0.33 | 0 ± 0 | 0 ± 0 | 3 ± 1.53 |
| Lasionectria | 0 ± 0 | 0 ± 0 | 4 ± 1.15 | 0.33 ± 0.33 | 0.33 ± 0.33 | 2.33 ± 2.33 | 0 ± 0 | 4 ± 4 | 0 ± 0 | 0 ± 0 |
| Lasiosphaeria | 0.67 ± 0.33 | 6.33 ± 5.33 | 12 ± 7.21 | 17 ± 11.5 | 0 ± 0 | 5.33 ± 3.93 | 7 ± 4.16 | 72.67 ± 41.42 | 16.67 ± 11.2 | 18.67 ± 6.96 |
| Latorua | 0 ± 0 | 0 ± 0 | 0 ± 0 | 0 ± 0 | 0.33 ± 0.33 | 0 ± 0 | 0.33 ± 0.33 | 0.67 ± 0.67 | 0 ± 0 | 0 ± 0 |
| Lecanicillium | 33.33 ± 13.22 | 16.67 ± 10.27 | 2 ± 2 | 9 ± 3.21 | 14.67 ± 0.67 | 16.67 ± 7.26 | 18.33 ± 3.84 | 18.67 ± 11.2 | 9.33 ± 6.98 | 6.67 ± 2.67 |
| Lecophagus | 0 ± 0 | 0 ± 0 | 1.33 ± 1.33 | 0 ± 0 | 0.33 ± 0.33 | 1 ± 1 | 1 ± 0.58 | 0 ± 0 | 0 ± 0 | 0 ± 0 |
| Lectera | 0 ± 0 | 23 ± 13.65 | 0 ± 0 | 0.67 ± 0.67 | 0 ± 0 | 1.33 ± 1.33 | 0.33 ± 0.33 | 0 ± 0 | 0 ± 0 | 0 ± 0 |
| Leiosphaerella | 0 ± 0 | 0 ± 0 | 0.33 ± 0.33 | 0 ± 0 | 0 ± 0 | 4.33 ± 3.84 | 0.67 ± 0.67 | 0 ± 0 | 0 ± 0 | 0 ± 0 |
| Leiothecium | 0 ± 0 | 0 ± 0 | 1 ± 1 | 0.67 ± 0.33 | 0 ± 0 | 0 ± 0 | 0 ± 0 | 0 ± 0 | 0 ± 0 | 0.67 ± 0.67 |
| Leohumicola | 0 ± 0 | 24.33 ± 11.26 | 3 ± 1.73 | 10.33 ± 3.84 | 0.33 ± 0.33 | 0.67 ± 0.67 | 9 ± 5.86 | 0.67 ± 0.33 | 2 ± 1.53 | 1.67 ± 1.67 |
| Lepiota | 0 ± 0 | 0.33 ± 0.33 | 0 ± 0 | 0 ± 0 | 0 ± 0 | 0 ± 0 | 4 ± 4 | 11.33 ± 2.19 | 2297.67 ± 2286.67 | 2.33 ± 1.45 |
| Lepista | 0 ± 0 | 0 ± 0 | 0.33 ± 0.33 | 0 ± 0 | 0 ± 0 | 0.33 ± 0.33 | 3 ± 3 | 1 ± 1 | 0 ± 0 | 0 ± 0 |
| Lepteutypa | 0 ± 0 | 0 ± 0 | 0 ± 0 | 0 ± 0 | 0 ± 0 | 1 ± 1 | 0 ± 0 | 0 ± 0 | 0 ± 0 | 0 ± 0 |
| Leptodiscella | 2.33 ± 1.86 | 6.33 ± 2.85 | 22 ± 8.5 | 3 ± 2.08 | 3 ± 1 | 6.33 ± 3.38 | 8 ± 2.65 | 0.33 ± 0.33 | 2.33 ± 1.86 | 6.33 ± 3.53 |
| Leptodontidium | 1 ± 1 | 38 ± 18.33 | 358 ± 109.15 | 17 ± 12.58 | 2 ± 0.58 | 117 ± 104.71 | 21 ± 10.15 | 220.33 ± 108.55 | 133 ± 99.65 | 299 ± 115.68 |
| Leptosphaeria | 0 ± 0 | 6.33 ± 2.6 | 0 ± 0 | 7.33 ± 4.67 | 0.67 ± 0.33 | 11.67 ± 7.06 | 4 ± 2.52 | 1 ± 1 | 0 ± 0 | 0.33 ± 0.33 |
| Leptosphaerulina | 25 ± 6.11 | 1586 ± 1059.82 | 5 ± 2.52 | 91 ± 74.01 | 4 ± 2.08 | 38.33 ± 19.81 | 88.33 ± 64.05 | 0 ± 0 | 1 ± 1 | 2.33 ± 1.45 |
| Leucoglossum | 7 ± 2.52 | 0 ± 0 | 0.33 ± 0.33 | 0 ± 0 | 9 ± 5.57 | 2.33 ± 2.33 | 0.33 ± 0.33 | 0 ± 0 | 0 ± 0 | 0 ± 0 |
| Leucosporidium | 0 ± 0 | 11.33 ± 8.51 | 11.33 ± 11.33 | 1 ± 1 | 0 ± 0 | 4.67 ± 4.67 | 1 ± 1 | 1 ± 1 | 0.33 ± 0.33 | 0 ± 0 |
| Liberomyces | 0 ± 0 | 0 ± 0 | 0 ± 0 | 0 ± 0 | 0 ± 0 | 0 ± 0 | 0 ± 0 | 8 ± 4.62 | 1.67 ± 1.67 | 0 ± 0 |
| Linderina | 0 ± 0 | 0 ± 0 | 2.33 ± 2.33 | 0 ± 0 | 0 ± 0 | 0 ± 0 | 0.33 ± 0.33 | 0 ± 0 | 0 ± 0 | 0 ± 0 |
| Lipomyces | 0 ± 0 | 0 ± 0 | 0.33 ± 0.33 | 2 ± 1.15 | 0 ± 0 | 0.33 ± 0.33 | 2 ± 1.15 | 5 ± 2.89 | 5.33 ± 3.93 | 4.67 ± 3.28 |
| Lomentospora | 0 ± 0 | 0 ± 0 | 0 ± 0 | 0 ± 0 | 0 ± 0 | 0 ± 0 | 0 ± 0 | 0 ± 0 | 0 ± 0 | 1.33 ± 1.33 |
| Longicollum | 0 ± 0 | 0 ± 0 | 0 ± 0 | 0 ± 0 | 0 ± 0 | 0.33 ± 0.33 | 0 ± 0 | 0 ± 0 | 0 ± 0 | 0 ± 0 |
| Lopadostoma | 0 ± 0 | 0 ± 0 | 0 ± 0 | 0 ± 0 | 0 ± 0 | 0 ± 0 | 0 ± 0 | 0 ± 0 | 0 ± 0 | 0 ± 0 |
| Lophiostoma | 2.33 ± 1.86 | 0.67 ± 0.67 | 20.67 ± 8.41 | 5.33 ± 2.6 | 4 ± 2 | 7 ± 3.61 | 27 ± 16.26 | 6 ± 1.53 | 3.67 ± 1.76 | 3.67 ± 0.67 |
| Lophiotrema | 0.67 ± 0.67 | 0 ± 0 | 0 ± 0 | 0 ± 0 | 0 ± 0 | 0 ± 0 | 0.67 ± 0.67 | 12.33 ± 5.67 | 3 ± 1.73 | 0 ± 0 |
| Lulworthia | 7.67 ± 5.17 | 0 ± 0 | 3 ± 3 | 0 ± 0 | 15.33 ± 8.45 | 3.67 ± 3.18 | 0.67 ± 0.67 | 1 ± 1 | 0 ± 0 | 0 ± 0 |
| Lylea | 0 ± 0 | 0 ± 0 | 0 ± 0 | 0 ± 0 | 0 ± 0 | 1.67 ± 1.67 | 0 ± 0 | 0 ± 0 | 0 ± 0 | 0 ± 0 |
| Macroconia | 0 ± 0 | 0.67 ± 0.67 | 1 ± 1 | 0 ± 0 | 0 ± 0 | 0 ± 0 | 0.67 ± 0.67 | 3 ± 3 | 1.67 ± 0.67 | 0 ± 0 |
| Macrophoma | 0 ± 0 | 0 ± 0 | 0 ± 0 | 0 ± 0 | 0 ± 0 | 0.33 ± 0.33 | 0.67 ± 0.67 | 0 ± 0 | 0 ± 0 | 0 ± 0 |
| Macrophomina | 0 ± 0 | 1.33 ± 0.88 | 0 ± 0 | 0.33 ± 0.33 | 0 ± 0 | 0 ± 0 | 0 ± 0 | 0 ± 0 | 0 ± 0 | 0 ± 0 |
| Madurella | 0 ± 0 | 0 ± 0 | 0 ± 0 | 0 ± 0 | 0 ± 0 | 2 ± 1.53 | 0 ± 0 | 0 ± 0 | 0 ± 0 | 0 ± 0 |
| Magnaporthiopsis | 0 ± 0 | 1 ± 1 | 0 ± 0 | 0 ± 0 | 0 ± 0 | 0 ± 0 | 0 ± 0 | 0 ± 0 | 0 ± 0 | 0 ± 0 |
| Magnibotryascoma | 0 ± 0 | 0 ± 0 | 0 ± 0 | 0 ± 0 | 0 ± 0 | 0 ± 0 | 0 ± 0 | 0 ± 0 | 0 ± 0 | 2.67 ± 2.67 |
| Malassezia | 0 ± 0 | 0 ± 0 | 2.33 ± 1.45 | 0.67 ± 0.67 | 0 ± 0 | 0.67 ± 0.67 | 2.67 ± 2.19 | 0 ± 0 | 1.33 ± 0.88 | 2.67 ± 1.33 |
| Malbranchea | 0 ± 0 | 0 ± 0 | 0 ± 0 | 0 ± 0 | 0 ± 0 | 0 ± 0 | 0 ± 0 | 0 ± 0 | 0 ± 0 | 1.33 ± 0.88 |
| Marasmiellus | 0 ± 0 | 0 ± 0 | 0 ± 0 | 0 ± 0 | 0 ± 0 | 0 ± 0 | 0 ± 0 | 0 ± 0 | 0.33 ± 0.33 | 20 ± 8.14 |
| Marasmius | 0 ± 0 | 1.33 ± 1.33 | 181.67 ± 28.5 | 1.33 ± 0.88 | 0.33 ± 0.33 | 44.33 ± 34.82 | 3 ± 1.73 | 0 ± 0 | 0 ± 0 | 0.67 ± 0.33 |
| Mariannaea | 2.33 ± 1.86 | 120.67 ± 43.03 | 5.67 ± 4.26 | 34.67 ± 10.2 | 7.67 ± 3.71 | 54 ± 21.17 | 45.67 ± 21.62 | 48.33 ± 16.13 | 21 ± 5.77 | 35.33 ± 12.41 |
| Martininia | 0 ± 0 | 0 ± 0 | 0.33 ± 0.33 | 0 ± 0 | 0 ± 0 | 2 ± 2 | 0 ± 0 | 0 ± 0 | 0 ± 0 | 0 ± 0 |
| Massarina | 2.67 ± 1.2 | 0.33 ± 0.33 | 0 ± 0 | 3.67 ± 0.67 | 27 ± 1.15 | 20.33 ± 3.67 | 39.33 ± 27.49 | 3.33 ± 2.33 | 5.67 ± 2.85 | 6.67 ± 1.76 |
| Matsushimaea | 0 ± 0 | 0 ± 0 | 0 ± 0 | 0 ± 0 | 0 ± 0 | 0 ± 0 | 0 ± 0 | 0 ± 0 | 0 ± 0 | 3.33 ± 2.85 |
| Medicopsis | 0 ± 0 | 0 ± 0 | 0 ± 0 | 0 ± 0 | 0 ± 0 | 0 ± 0 | 2 ± 2 | 0.33 ± 0.33 | 0 ± 0 | 0 ± 0 |
| Melanconiella | 11.33 ± 7.36 | 50.67 ± 35.47 | 1 ± 0.58 | 4.67 ± 3.67 | 5.33 ± 2.85 | 2.67 ± 1.67 | 26 ± 13.8 | 9 ± 4.51 | 5 ± 3.61 | 13.33 ± 8.35 |
| Melanocarpus | 0 ± 0 | 0 ± 0 | 0 ± 0 | 0 ± 0 | 0.33 ± 0.33 | 0 ± 0 | 0 ± 0 | 0 ± 0 | 0 ± 0 | 0 ± 0 |
| Melanocucurbitaria | 0 ± 0 | 0 ± 0 | 0 ± 0 | 0 ± 0 | 0 ± 0 | 0 ± 0 | 1 ± 1 | 0 ± 0 | 0 ± 0 | 0 ± 0 |
| Melanomma | 0 ± 0 | 0 ± 0 | 0 ± 0 | 0 ± 0 | 0 ± 0 | 0 ± 0 | 2 ± 2 | 0 ± 0 | 0 ± 0 | 0 ± 0 |
| Melanophyllum | 0 ± 0 | 0 ± 0 | 0 ± 0 | 0 ± 0 | 0.33 ± 0.33 | 0 ± 0 | 2.33 ± 2.33 | 0 ± 0 | 0.33 ± 0.33 | 0 ± 0 |
| Meliniomyces | 0.33 ± 0.33 | 11.67 ± 9.21 | 10 ± 5.13 | 3.67 ± 2.19 | 0 ± 0 | 1.33 ± 0.88 | 1.67 ± 1.2 | 0.33 ± 0.33 | 0 ± 0 | 34.67 ± 29.34 |
| Memnoniella | 7.67 ± 1.76 | 0 ± 0 | 0.67 ± 0.67 | 2 ± 2 | 156 ± 23.64 | 20.67 ± 9.21 | 12 ± 3.51 | 4 ± 3.51 | 2.33 ± 2.33 | 20 ± 16.17 |
| Merimbla | 0 ± 0 | 0 ± 0 | 0 ± 0 | 0 ± 0 | 0 ± 0 | 0 ± 0 | 0 ± 0 | 0 ± 0 | 0 ± 0 | 0.67 ± 0.67 |
| Metapochonia | 0 ± 0 | 0.67 ± 0.67 | 0.67 ± 0.33 | 0.33 ± 0.33 | 0.33 ± 0.33 | 6.33 ± 4.1 | 16.67 ± 14.71 | 9.33 ± 6.98 | 7.33 ± 6.84 | 0.67 ± 0.67 |
| Metarhizium | 69 ± 17.5 | 31.33 ± 20.88 | 813 ± 470.41 | 138.67 ± 58.54 | 85 ± 19.63 | 601.67 ± 176.62 | 260 ± 91.51 | 503 ± 262.73 | 248.67 ± 223.35 | 644.33 ± 207.95 |
| Microascus | 1 ± 0.58 | 1.67 ± 1.2 | 1.33 ± 1.33 | 0 ± 0 | 0.33 ± 0.33 | 0.67 ± 0.33 | 3.67 ± 2.03 | 0 ± 0 | 0 ± 0 | 0 ± 0 |
| Microcera | 0 ± 0 | 0 ± 0 | 0 ± 0 | 0 ± 0 | 0 ± 0 | 0 ± 0 | 0.33 ± 0.33 | 0 ± 0 | 0 ± 0 | 3.33 ± 1.2 |
| Microdiplodia | 1.67 ± 0.88 | 0 ± 0 | 93.67 ± 35.67 | 7 ± 3.21 | 3.33 ± 1.33 | 32 ± 24.01 | 11 ± 8.19 | 0 ± 0 | 0 ± 0 | 1 ± 1 |
| Microdochium | 2.33 ± 1.2 | 0 ± 0 | 14.33 ± 9.06 | 1 ± 0.58 | 12 ± 7.51 | 3 ± 1.53 | 2.67 ± 1.2 | 7.67 ± 4.33 | 18.67 ± 18.67 | 1.67 ± 1.67 |
| Micronematobotrys | 0.67 ± 0.67 | 0 ± 0 | 0 ± 0 | 0 ± 0 | 0 ± 0 | 0 ± 0 | 0 ± 0 | 0 ± 0 | 0 ± 0 | 0 ± 0 |
| Microscypha | 2 ± 1.53 | 0.33 ± 0.33 | 0 ± 0 | 0 ± 0 | 0.67 ± 0.67 | 0 ± 0 | 0 ± 0 | 0 ± 0 | 0 ± 0 | 0 ± 0 |
| Microsphaeropsis | 0.67 ± 0.67 | 0 ± 0 | 58.67 ± 8.41 | 0 ± 0 | 1.67 ± 0.67 | 118 ± 92.12 | 8.67 ± 3.28 | 0 ± 0 | 0.33 ± 0.33 | 11.67 ± 4.26 |
| Microthelia | 0 ± 0 | 0 ± 0 | 0 ± 0 | 0 ± 0 | 0 ± 0 | 5 ± 4.51 | 0.33 ± 0.33 | 0 ± 0 | 0 ± 0 | 0 ± 0 |
| Miniancora | 0 ± 0 | 0.33 ± 0.33 | 0 ± 0 | 0 ± 0 | 0 ± 0 | 0 ± 0 | 0 ± 0 | 0.67 ± 0.67 | 0.67 ± 0.67 | 0 ± 0 |
| Minimedusa | 0 ± 0 | 0.33 ± 0.33 | 0.33 ± 0.33 | 0.33 ± 0.33 | 1.33 ± 0.67 | 17 ± 13.53 | 2.67 ± 1.33 | 0 ± 0 | 0 ± 0 | 1.67 ± 1.67 |
| Minimelanolocus | 0.33 ± 0.33 | 6.67 ± 2.96 | 0 ± 0 | 2.33 ± 1.45 | 0 ± 0 | 0.67 ± 0.67 | 2 ± 1.53 | 0.33 ± 0.33 | 1.67 ± 0.88 | 0 ± 0 |
| Minimidochium | 0 ± 0 | 5.33 ± 3.33 | 0 ± 0 | 0 ± 0 | 0 ± 0 | 0 ± 0 | 1.33 ± 1.33 | 0 ± 0 | 0 ± 0 | 0 ± 0 |
| Minutisphaera | 0 ± 0 | 0 ± 0 | 0 ± 0 | 0 ± 0 | 0 ± 0 | 0 ± 0 | 0 ± 0 | 0 ± 0 | 0 ± 0 | 0 ± 0 |
| Mirandina | 2 ± 2 | 1 ± 1 | 93.67 ± 44.29 | 3.33 ± 1.33 | 4.67 ± 2.33 | 13.67 ± 7.22 | 5.33 ± 2.4 | 60 ± 37.51 | 153.67 ± 153.17 | 15 ± 14.01 |
| Modicella | 0 ± 0 | 0 ± 0 | 0 ± 0 | 1 ± 1 | 0 ± 0 | 0 ± 0 | 0.67 ± 0.67 | 0 ± 0 | 0 ± 0 | 0 ± 0 |
| Moesziomyces | 0 ± 0 | 0 ± 0 | 0 ± 0 | 0 ± 0 | 0 ± 0 | 0 ± 0 | 0 ± 0 | 0 ± 0 | 0 ± 0 | 3.67 ± 3.67 |
| Mollisia | 0 ± 0 | 0 ± 0 | 0 ± 0 | 0 ± 0 | 0 ± 0 | 1.33 ± 1.33 | 0 ± 0 | 5.33 ± 2.91 | 2.67 ± 0.88 | 5.67 ± 0.33 |
| Mollisina | 0 ± 0 | 0 ± 0 | 0 ± 0 | 0 ± 0 | 0 ± 0 | 0 ± 0 | 0 ± 0 | 0 ± 0 | 0 ± 0 | 0 ± 0 |
| Monacrosporium | 0 ± 0 | 2 ± 1 | 0.33 ± 0.33 | 0 ± 0 | 0.33 ± 0.33 | 0.67 ± 0.67 | 0 ± 0 | 2.67 ± 0.88 | 0.33 ± 0.33 | 0.67 ± 0.33 |
| Monilinia | 0 ± 0 | 0.67 ± 0.67 | 0 ± 0 | 0 ± 0 | 0 ± 0 | 0 ± 0 | 0 ± 0 | 0 ± 0 | 0 ± 0 | 0 ± 0 |
| Monocillium | 0 ± 0 | 0 ± 0 | 3 ± 3 | 24.33 ± 9.21 | 0 ± 0 | 12.67 ± 7.69 | 11.33 ± 7.54 | 13 ± 6.43 | 4.33 ± 4.33 | 0.67 ± 0.67 |
| Monodictys | 0.33 ± 0.33 | 0 ± 0 | 5.67 ± 5.67 | 0 ± 0 | 8.67 ± 2.19 | 0.33 ± 0.33 | 3.33 ± 3.33 | 0 ± 0 | 0 ± 0 | 17.33 ± 17.33 |
| Monosporascus | 0 ± 0 | 0 ± 0 | 1.33 ± 1.33 | 0.33 ± 0.33 | 0.33 ± 0.33 | 0 ± 0 | 1 ± 0.58 | 0 ± 0 | 0 ± 0 | 0 ± 0 |
| Montagnula | 0 ± 0 | 0 ± 0 | 0 ± 0 | 0 ± 0 | 0 ± 0 | 0 ± 0 | 0.33 ± 0.33 | 0.67 ± 0.67 | 0.33 ± 0.33 | 0.67 ± 0.67 |
| Mortierella | 193.67 ± 67.35 | 1320.67 ± 111.76 | 2725.67 ± 397.85 | 1745 ± 935.94 | 517.67 ± 116.12 | 5523 ± 1415.37 | 3386.67 ± 1034.61 | 1718.33 ± 754.42 | 1544 ± 1175.52 | 2783 ± 423.62 |
| Mrakia | 1 ± 0.58 | 1 ± 1 | 33.67 ± 14.86 | 0.33 ± 0.33 | 1.33 ± 0.67 | 21.33 ± 7.75 | 3 ± 0.58 | 28.33 ± 25.87 | 0.33 ± 0.33 | 2.67 ± 2.67 |
| Muscinupta | 0 ± 0 | 0 ± 0 | 0.33 ± 0.33 | 0 ± 0 | 0 ± 0 | 0.33 ± 0.33 | 0 ± 0 | 8 ± 7.51 | 0.67 ± 0.67 | 0.67 ± 0.33 |
| Muscodor | 0 ± 0 | 0 ± 0 | 0 ± 0 | 0 ± 0 | 0 ± 0 | 0 ± 0 | 0 ± 0 | 0.33 ± 0.33 | 0.67 ± 0.67 | 0 ± 0 |
| Musicillium | 0 ± 0 | 0 ± 0 | 2 ± 2 | 0.33 ± 0.33 | 0 ± 0 | 0 ± 0 | 0.33 ± 0.33 | 0 ± 0 | 0 ± 0 | 1.67 ± 1.67 |
| Myceliophthora | 0 ± 0 | 0 ± 0 | 0 ± 0 | 0 ± 0 | 0 ± 0 | 0 ± 0 | 0 ± 0 | 0 ± 0 | 0 ± 0 | 0 ± 0 |
| Mycena | 0.67 ± 0.33 | 13 ± 10.15 | 2.67 ± 2.67 | 4 ± 2.08 | 0 ± 0 | 0 ± 0 | 1.33 ± 0.67 | 0 ± 0 | 0.67 ± 0.67 | 14.67 ± 13.68 |
| Mycocentrospora | 0 ± 0 | 0 ± 0 | 0.67 ± 0.67 | 8.33 ± 6.84 | 0 ± 0 | 0 ± 0 | 2.67 ± 2.67 | 0 ± 0 | 0 ± 0 | 0 ± 0 |
| Mycogone | 0 ± 0 | 0 ± 0 | 0 ± 0 | 0 ± 0 | 0 ± 0 | 4.33 ± 4.33 | 0 ± 0 | 0 ± 0 | 0 ± 0 | 0 ± 0 |
| Mycoleptodiscus | 0 ± 0 | 0.67 ± 0.67 | 4.33 ± 1.76 | 1.67 ± 1.67 | 0 ± 0 | 0.67 ± 0.67 | 1 ± 1 | 0 ± 0 | 0 ± 0 | 0 ± 0 |
| Mycosphaerella | 2 ± 2 | 0.33 ± 0.33 | 2 ± 1.15 | 1.67 ± 1.67 | 1.33 ± 0.88 | 1 ± 0.58 | 8.33 ± 2.96 | 16.67 ± 15.19 | 4 ± 3 | 2 ± 1 |
| Mycothermus | 0 ± 0 | 0 ± 0 | 0 ± 0 | 0 ± 0 | 0 ± 0 | 6.33 ± 4.91 | 1.33 ± 0.88 | 0 ± 0 | 0 ± 0 | 2.67 ± 2.67 |
| Myriangium | 0 ± 0 | 0 ± 0 | 0 ± 0 | 0 ± 0 | 0 ± 0 | 0 ± 0 | 0 ± 0 | 0 ± 0 | 0 ± 0 | 0.33 ± 0.33 |
| Myriococcum | 0 ± 0 | 0 ± 0 | 0 ± 0 | 0 ± 0 | 0 ± 0 | 1.33 ± 0.67 | 0 ± 0 | 0 ± 0 | 0 ± 0 | 0 ± 0 |
| Myriodontium | 0 ± 0 | 0 ± 0 | 0 ± 0 | 0.67 ± 0.67 | 0 ± 0 | 1 ± 0.58 | 1 ± 1 | 0 ± 0 | 0 ± 0 | 0 ± 0 |
| Myrmecridium | 16.67 ± 4.81 | 167 ± 159.5 | 1.67 ± 0.33 | 11.33 ± 11.33 | 2.33 ± 1.45 | 3.33 ± 1.45 | 17 ± 12 | 9.67 ± 5.93 | 2 ± 1.53 | 5.67 ± 0.33 |
| Myrothecium | 0.67 ± 0.67 | 27.67 ± 20.2 | 4 ± 2.52 | 2 ± 2 | 0.33 ± 0.33 | 7.33 ± 1.2 | 42.33 ± 36.89 | 14.33 ± 7.97 | 1.67 ± 0.33 | 2.33 ± 1.2 |
| Myxospora | 2 ± 1.15 | 0 ± 0 | 0 ± 0 | 0 ± 0 | 0.67 ± 0.67 | 2 ± 1 | 0.33 ± 0.33 | 0 ± 0 | 0 ± 0 | 0.33 ± 0.33 |
| Naganishia | 0 ± 0 | 1 ± 1 | 0 ± 0 | 0 ± 0 | 0 ± 0 | 0 ± 0 | 0 ± 0 | 0 ± 0 | 0 ± 0 | 0 ± 0 |
| Nectria | 13.67 ± 13.17 | 2.67 ± 1.67 | 82 ± 33.78 | 4.67 ± 3.67 | 7.67 ± 2.85 | 41.67 ± 15.98 | 13 ± 3 | 66.33 ± 34.84 | 23.33 ± 23.33 | 5.67 ± 3.71 |
| Nectricladiella | 0 ± 0 | 0 ± 0 | 0 ± 0 | 0 ± 0 | 0 ± 0 | 9.67 ± 9.17 | 0 ± 0 | 0 ± 0 | 0 ± 0 | 0 ± 0 |
| Nemania | 0 ± 0 | 0 ± 0 | 0 ± 0 | 0 ± 0 | 0 ± 0 | 0.33 ± 0.33 | 0 ± 0 | 0 ± 0 | 0 ± 0 | 1.67 ± 1.2 |
| Neoascochyta | 0 ± 0 | 0 ± 0 | 0 ± 0 | 0 ± 0 | 0 ± 0 | 3 ± 1.15 | 5.67 ± 4.18 | 0 ± 0 | 0 ± 0 | 1.67 ± 0.33 |
| Neoascotaiwania | 0 ± 0 | 0 ± 0 | 0 ± 0 | 0 ± 0 | 0 ± 0 | 0 ± 0 | 0 ± 0 | 0 ± 0 | 0 ± 0 | 0.33 ± 0.33 |
| Neobulgaria | 0 ± 0 | 0 ± 0 | 0 ± 0 | 0 ± 0 | 0 ± 0 | 0 ± 0 | 0 ± 0 | 0 ± 0 | 0 ± 0 | 0 ± 0 |
| Neoceratosperma | 0.33 ± 0.33 | 0 ± 0 | 0 ± 0 | 0 ± 0 | 0 ± 0 | 0 ± 0 | 0 ± 0 | 0 ± 0 | 0.33 ± 0.33 | 0 ± 0 |
| Neocucurbitaria | 0 ± 0 | 45.33 ± 22.73 | 0 ± 0 | 1 ± 0.58 | 0 ± 0 | 0 ± 0 | 7.67 ± 3.84 | 0 ± 0 | 0 ± 0 | 0 ± 0 |
| Neodactylaria | 0 ± 0 | 0.33 ± 0.33 | 0.33 ± 0.33 | 5.67 ± 5.67 | 0.33 ± 0.33 | 0 ± 0 | 3.67 ± 3.67 | 0 ± 0 | 0 ± 0 | 0 ± 0 |
| Neodendryphiella | 0 ± 0 | 0 ± 0 | 0.67 ± 0.67 | 0 ± 0 | 0 ± 0 | 0 ± 0 | 0 ± 0 | 0 ± 0 | 0 ± 0 | 0 ± 0 |
| Neodevriesia | 0 ± 0 | 0 ± 0 | 1.67 ± 1.67 | 1 ± 1 | 0 ± 0 | 1 ± 0.58 | 1.67 ± 0.33 | 9 ± 4.73 | 5.33 ± 5.33 | 0.33 ± 0.33 |
| Neofabraea | 0 ± 0 | 0 ± 0 | 15.67 ± 12.33 | 0.33 ± 0.33 | 0 ± 0 | 6.33 ± 4.91 | 0.67 ± 0.67 | 0 ± 0 | 0 ± 0 | 5 ± 3.61 |
| Neofusicoccum | 4.33 ± 4.33 | 0 ± 0 | 0 ± 0 | 0 ± 0 | 0.33 ± 0.33 | 0 ± 0 | 0 ± 0 | 4 ± 2.08 | 2.67 ± 0.67 | 0.33 ± 0.33 |
| Neohygrocybe | 0.33 ± 0.33 | 0 ± 0 | 1.67 ± 1.2 | 0 ± 0 | 0 ± 0 | 0 ± 0 | 0 ± 0 | 0 ± 0 | 0 ± 0 | 0 ± 0 |
| Neoidriella | 0 ± 0 | 1 ± 1 | 0 ± 0 | 0 ± 0 | 0 ± 0 | 0 ± 0 | 0 ± 0 | 0 ± 0 | 0 ± 0 | 0 ± 0 |
| Neomassarina | 0.67 ± 0.33 | 0 ± 0 | 0 ± 0 | 0.33 ± 0.33 | 0 ± 0 | 0 ± 0 | 1 ± 0.58 | 1.33 ± 0.33 | 0 ± 0 | 0.67 ± 0.67 |
| Neonectria | 118.33 ± 31.75 | 13 ± 8.02 | 1.67 ± 1.2 | 25.67 ± 8.41 | 17 ± 8.5 | 8 ± 6.51 | 23.67 ± 3.48 | 5.67 ± 1.86 | 0.33 ± 0.33 | 33 ± 14.8 |
| Neopestalotiopsis | 4.33 ± 0.88 | 17.67 ± 10.67 | 71 ± 25.7 | 4.67 ± 1.86 | 2.33 ± 0.88 | 21.67 ± 13.72 | 36.33 ± 21.61 | 222.67 ± 108.11 | 60.67 ± 48.67 | 14 ± 1.73 |
| Neopyrenochaeta | 2.33 ± 2.33 | 4 ± 3.06 | 0 ± 0 | 0 ± 0 | 8.33 ± 1.76 | 22.67 ± 8.33 | 4.33 ± 1.33 | 64.33 ± 38.2 | 20 ± 20 | 1 ± 0 |
| Neurospora | 0 ± 0 | 0 ± 0 | 0 ± 0 | 0 ± 0 | 0 ± 0 | 0 ± 0 | 0 ± 0 | 0 ± 0 | 0 ± 0 | 0 ± 0 |
| Niesslia | 0 ± 0 | 0 ± 0 | 0.33 ± 0.33 | 3.67 ± 3.67 | 0 ± 0 | 1 ± 1 | 3.33 ± 1.86 | 0 ± 0 | 0.33 ± 0.33 | 0 ± 0 |
| Nigrograna | 0 ± 0 | 0 ± 0 | 3.33 ± 3.33 | 0.33 ± 0.33 | 0.33 ± 0.33 | 15.67 ± 6.36 | 11.33 ± 10.35 | 3 ± 3 | 1 ± 1 | 1 ± 0.58 |
| Nigrospora | 7.67 ± 2.33 | 174.67 ± 171.68 | 53.33 ± 10.11 | 13 ± 7.09 | 8.67 ± 3.76 | 48.67 ± 17.17 | 80.67 ± 34.17 | 63 ± 35.93 | 14.67 ± 12.17 | 12 ± 5 |
| Ochroconis | 1.33 ± 1.33 | 12 ± 5.69 | 0.33 ± 0.33 | 1 ± 0.58 | 0 ± 0 | 0 ± 0 | 2.67 ± 2.67 | 11.67 ± 7.31 | 11 ± 6.43 | 1 ± 1 |
| Oculimacula | 28.33 ± 20.63 | 26.67 ± 3.18 | 5.67 ± 1.67 | 2.33 ± 0.88 | 7.67 ± 2.6 | 15.33 ± 6.74 | 19 ± 1 | 121 ± 82.24 | 57.67 ± 57.17 | 7 ± 2 |
| Oidiodendron | 0 ± 0 | 0.33 ± 0.33 | 2.67 ± 2.67 | 7 ± 3 | 0 ± 0 | 5.67 ± 1.76 | 6.33 ± 2.96 | 25.33 ± 8.67 | 11.33 ± 6.57 | 13.67 ± 3.28 |
| Oliveonia | 130.33 ± 29.36 | 6 ± 5.51 | 21 ± 17.09 | 0.33 ± 0.33 | 37.67 ± 13.92 | 5.33 ± 4.84 | 6.67 ± 3.18 | 5.33 ± 2.73 | 4.67 ± 2.33 | 2.67 ± 1.76 |
| Olpidium | 37 ± 8.5 | 75.67 ± 10.17 | 3.33 ± 2.4 | 12.33 ± 2.85 | 19.33 ± 3.84 | 255.33 ± 126.65 | 45 ± 11 | 2 ± 1.15 | 2.67 ± 1.76 | 13.67 ± 11.26 |
| Omphalina | 4.33 ± 2.4 | 0 ± 0 | 0 ± 0 | 0 ± 0 | 0 ± 0 | 0 ± 0 | 0.33 ± 0.33 | 37.67 ± 25.39 | 15.33 ± 15.33 | 0 ± 0 |
| Oncopodiella | 2.67 ± 2.67 | 4.33 ± 1.76 | 0 ± 0 | 0 ± 0 | 0.33 ± 0.33 | 2.67 ± 2.67 | 1 ± 0.58 | 3.33 ± 3.33 | 6 ± 6 | 0.67 ± 0.67 |
| Operculomyces | 0 ± 0 | 0 ± 0 | 0.67 ± 0.67 | 6.33 ± 6.33 | 0 ± 0 | 0.33 ± 0.33 | 4.33 ± 3.33 | 0.67 ± 0.67 | 0 ± 0 | 0 ± 0 |
| Ophiocordyceps | 5.67 ± 4.26 | 0.33 ± 0.33 | 1.33 ± 0.88 | 0.33 ± 0.33 | 2 ± 1.53 | 5 ± 2.52 | 7.67 ± 7.67 | 8 ± 5.29 | 1.33 ± 0.88 | 0 ± 0 |
| Ophiosphaerella | 0.33 ± 0.33 | 0 ± 0 | 0.33 ± 0.33 | 0.33 ± 0.33 | 0 ± 0 | 0 ± 0 | 17.33 ± 17.33 | 3.33 ± 2.85 | 0 ± 0 | 0 ± 0 |
| Orbilia | 0 ± 0 | 0 ± 0 | 0.33 ± 0.33 | 0 ± 0 | 0 ± 0 | 2.67 ± 1.76 | 0 ± 0 | 0 ± 0 | 0 ± 0 | 1.33 ± 1.33 |
| Ovatospora | 0 ± 0 | 0 ± 0 | 1.33 ± 1.33 | 0 ± 0 | 0 ± 0 | 0 ± 0 | 0 ± 0 | 0 ± 0 | 0 ± 0 | 0 ± 0 |
| Paecilomyces | 1 ± 1 | 21 ± 16.62 | 18.33 ± 5.55 | 14.33 ± 8.25 | 1.67 ± 1.2 | 64.33 ± 21.76 | 13 ± 5.57 | 14.67 ± 7.88 | 4.33 ± 2.85 | 2.33 ± 0.33 |
| Papiliotrema | 39.67 ± 25.98 | 9.67 ± 4.81 | 27.67 ± 20.27 | 5 ± 3.51 | 32 ± 1 | 11.67 ± 3.53 | 11 ± 1.73 | 140 ± 108.68 | 32.67 ± 29.67 | 21 ± 6.24 |
| Paracamarosporium | 0 ± 0 | 0 ± 0 | 0 ± 0 | 1.33 ± 0.88 | 0 ± 0 | 0.67 ± 0.67 | 0.33 ± 0.33 | 0 ± 0 | 0 ± 0 | 0.33 ± 0.33 |
| Paracladophialophora | 0 ± 0 | 0 ± 0 | 0 ± 0 | 0 ± 0 | 0 ± 0 | 0 ± 0 | 0 ± 0 | 0.67 ± 0.67 | 3 ± 0.58 | 0 ± 0 |
| Paraconiothyrium | 0 ± 0 | 0 ± 0 | 3.67 ± 2.33 | 0 ± 0 | 0 ± 0 | 3.67 ± 1.76 | 1.67 ± 0.88 | 0 ± 0 | 0.33 ± 0.33 | 0 ± 0 |
| Paracremonium | 0 ± 0 | 0 ± 0 | 1.33 ± 0.67 | 0.67 ± 0.67 | 5.33 ± 1.45 | 8.33 ± 2.4 | 5 ± 2.89 | 1 ± 0.58 | 1.67 ± 1.2 | 0.67 ± 0.67 |
| Parafabraea | 0 ± 0 | 0 ± 0 | 0 ± 0 | 0 ± 0 | 0 ± 0 | 1 ± 1 | 0 ± 0 | 0 ± 0 | 0 ± 0 | 0 ± 0 |
| Paraglomus | 0 ± 0 | 27.67 ± 13.35 | 0 ± 0 | 19.67 ± 1.45 | 0.33 ± 0.33 | 0.33 ± 0.33 | 7.67 ± 4.81 | 5.67 ± 3.84 | 7.67 ± 1.76 | 27.33 ± 7.88 |
| Paramicrosporidium | 1.33 ± 0.88 | 0 ± 0 | 0 ± 0 | 0 ± 0 | 0 ± 0 | 0.33 ± 0.33 | 0 ± 0 | 0 ± 0 | 0 ± 0 | 0 ± 0 |
| Paramicrothyrium | 0.67 ± 0.67 | 0 ± 0 | 1.67 ± 0.33 | 0 ± 0 | 0.67 ± 0.67 | 2.67 ± 1.33 | 0 ± 0 | 2.67 ± 2.67 | 0 ± 0 | 0 ± 0 |
| Paramyrothecium | 8 ± 6.56 | 12.33 ± 6.69 | 0.33 ± 0.33 | 0.67 ± 0.67 | 27.67 ± 11.33 | 7.33 ± 2.19 | 7 ± 3.06 | 13.67 ± 10.2 | 24 ± 6.51 | 0.67 ± 0.67 |
| Paraphaeosphaeria | 0.67 ± 0.67 | 0 ± 0 | 0 ± 0 | 0 ± 0 | 0.67 ± 0.67 | 8.33 ± 2.03 | 2.33 ± 1.45 | 28.67 ± 14.53 | 20.67 ± 18.68 | 0.67 ± 0.33 |
| Paraphoma | 7.67 ± 0.67 | 11 ± 7.21 | 0.67 ± 0.67 | 2.33 ± 0.67 | 11.33 ± 5.49 | 8 ± 2.52 | 13 ± 4.58 | 19 ± 9.61 | 10.33 ± 9.35 | 11.67 ± 8.21 |
| Paraphysoderma | 0 ± 0 | 0 ± 0 | 105.67 ± 25.77 | 1.33 ± 0.88 | 2.33 ± 1.2 | 33.33 ± 27.95 | 0.33 ± 0.33 | 0.33 ± 0.33 | 0 ± 0 | 2.33 ± 2.33 |
| Parapleurotheciopsis | 0 ± 0 | 0 ± 0 | 0 ± 0 | 0 ± 0 | 0 ± 0 | 2.33 ± 2.33 | 0 ± 0 | 0 ± 0 | 0 ± 0 | 0 ± 0 |
| Parascedosporium | 0 ± 0 | 0 ± 0 | 0 ± 0 | 0 ± 0 | 0 ± 0 | 2 ± 2 | 2 ± 2 | 2 ± 2 | 0 ± 0 | 1 ± 0.58 |
| Parasola | 7.33 ± 4.06 | 0.33 ± 0.33 | 5 ± 1.15 | 0 ± 0 | 0.67 ± 0.67 | 0 ± 0 | 0 ± 0 | 0 ± 0 | 0 ± 0 | 0 ± 0 |
| Parastagonospora | 0 ± 0 | 0 ± 0 | 0 ± 0 | 0 ± 0 | 0.33 ± 0.33 | 0.67 ± 0.67 | 0 ± 0 | 0 ± 0 | 0 ± 0 | 0 ± 0 |
| Parathyridaria | 0 ± 0 | 0.33 ± 0.33 | 2.67 ± 1.76 | 0 ± 0 | 2 ± 1.53 | 2.67 ± 0.88 | 11 ± 8.19 | 0 ± 0 | 0 ± 0 | 4 ± 3 |
| Passalora | 0 ± 0 | 0 ± 0 | 0 ± 0 | 0 ± 0 | 0 ± 0 | 0 ± 0 | 0 ± 0 | 0 ± 0 | 0 ± 0 | 0 ± 0 |
| Paurocotylis | 0 ± 0 | 0 ± 0 | 0.67 ± 0.67 | 0 ± 0 | 0 ± 0 | 0 ± 0 | 0 ± 0 | 0 ± 0 | 0 ± 0 | 0 ± 0 |
| Penicillifer | 0 ± 0 | 5 ± 5 | 10 ± 5.13 | 2.67 ± 1.2 | 3.67 ± 3.67 | 33 ± 13.8 | 16.67 ± 8.29 | 0 ± 0 | 0.67 ± 0.67 | 10 ± 4.93 |
| Penicillium | 113 ± 64.13 | 523.33 ± 195.16 | 160.33 ± 17.57 | 114.67 ± 38.71 | 77.33 ± 19.89 | 381.67 ± 50.86 | 549.67 ± 152.19 | 1557.33 ± 784.16 | 612.33 ± 568.34 | 253.67 ± 115.25 |
| Peniophora | 0 ± 0 | 0 ± 0 | 0.33 ± 0.33 | 2 ± 2 | 0 ± 0 | 7.33 ± 5.04 | 1.33 ± 0.67 | 1.33 ± 1.33 | 0 ± 0 | 5.67 ± 3.48 |
| Perenniporia | 0 ± 0 | 2.67 ± 1.76 | 0 ± 0 | 0.67 ± 0.67 | 0 ± 0 | 0.33 ± 0.33 | 5.33 ± 5.33 | 0.67 ± 0.67 | 0 ± 0 | 34 ± 10.39 |
| Periconia | 6.67 ± 0.88 | 8.67 ± 3.38 | 14.67 ± 6.01 | 2 ± 0.58 | 26 ± 12.22 | 16.33 ± 4.91 | 7.67 ± 2.67 | 80 ± 39.02 | 34 ± 19.5 | 7.33 ± 2.67 |
| Periglandula | 0 ± 0 | 2.33 ± 2.33 | 0 ± 0 | 0.33 ± 0.33 | 0 ± 0 | 0.67 ± 0.67 | 1.67 ± 1.67 | 1.67 ± 0.88 | 0 ± 0 | 0.33 ± 0.33 |
| Pervetustus | 0.33 ± 0.33 | 0 ± 0 | 0.67 ± 0.67 | 0 ± 0 | 2 ± 0.58 | 0.67 ± 0.67 | 0.33 ± 0.33 | 22.67 ± 11.57 | 16 ± 16 | 0 ± 0 |
| Pestalotiopsis | 1.67 ± 0.88 | 2.67 ± 1.2 | 14.67 ± 0.88 | 3.67 ± 1.76 | 1.67 ± 0.67 | 14.33 ± 8.35 | 10 ± 1 | 64 ± 32.08 | 38.33 ± 28.83 | 6.33 ± 1.2 |
| Petrakia | 0.33 ± 0.33 | 0 ± 0 | 0 ± 0 | 0.67 ± 0.33 | 0 ± 0 | 0 ± 0 | 1.67 ± 0.88 | 2.33 ± 1.86 | 11 ± 11 | 0.33 ± 0.33 |
| Petriella | 0 ± 0 | 0 ± 0 | 0 ± 0 | 0 ± 0 | 0 ± 0 | 0 ± 0 | 0.67 ± 0.67 | 1.67 ± 1.67 | 0 ± 0 | 0 ± 0 |
| Pezicula | 1 ± 0.58 | 0 ± 0 | 7.67 ± 3.71 | 0 ± 0 | 0 ± 0 | 0.67 ± 0.33 | 1.33 ± 1.33 | 5.67 ± 3.18 | 1.33 ± 1.33 | 3.33 ± 2.85 |
| Peziza | 0 ± 0 | 0 ± 0 | 0.67 ± 0.33 | 0 ± 0 | 0 ± 0 | 1 ± 1 | 0 ± 0 | 0 ± 0 | 0 ± 0 | 0 ± 0 |
| Pezizella | 4 ± 2.52 | 0 ± 0 | 1.67 ± 0.88 | 11 ± 9.5 | 1.33 ± 0.88 | 90.67 ± 45.32 | 14.33 ± 11.92 | 0 ± 0 | 0 ± 0 | 0.33 ± 0.33 |
| Phacidiella | 0 ± 0 | 0 ± 0 | 0 ± 0 | 0 ± 0 | 0 ± 0 | 1 ± 1 | 0 ± 0 | 0 ± 0 | 0 ± 0 | 0 ± 0 |
| Phaeoacremonium | 0 ± 0 | 0 ± 0 | 0.67 ± 0.67 | 0 ± 0 | 0.67 ± 0.33 | 0 ± 0 | 0 ± 0 | 0 ± 0 | 0 ± 0 | 0 ± 0 |
| Phaeococcomyces | 1.67 ± 1.67 | 0 ± 0 | 0 ± 0 | 0 ± 0 | 0 ± 0 | 0 ± 0 | 0 ± 0 | 0 ± 0 | 0 ± 0 | 0 ± 0 |
| Phaeoisaria | 0 ± 0 | 0 ± 0 | 0 ± 0 | 0 ± 0 | 0.33 ± 0.33 | 0 ± 0 | 1.67 ± 1.67 | 0 ± 0 | 0 ± 0 | 1.33 ± 1.33 |
| Phaeomoniella | 0 ± 0 | 3 ± 2.52 | 1.67 ± 0.33 | 0 ± 0 | 0 ± 0 | 33.33 ± 20.85 | 4.67 ± 2.91 | 6.67 ± 3.76 | 2.67 ± 1.2 | 1.67 ± 0.88 |
| Phaeophyscia | 0 ± 0 | 0 ± 0 | 0 ± 0 | 0 ± 0 | 0 ± 0 | 0 ± 0 | 0 ± 0 | 0 ± 0 | 0 ± 0 | 2 ± 2 |
| Phaeosphaeria | 0 ± 0 | 57.33 ± 19.55 | 0 ± 0 | 2.33 ± 1.86 | 0 ± 0 | 14 ± 8.74 | 5 ± 4.04 | 9.33 ± 7.88 | 7.67 ± 7.67 | 0 ± 0 |
| Phaeosphaeriopsis | 5 ± 2.65 | 41.33 ± 26.12 | 6.67 ± 3.71 | 2.67 ± 2.67 | 0.67 ± 0.67 | 3 ± 0.58 | 7 ± 3.21 | 20.67 ± 16.29 | 3 ± 1.15 | 14 ± 3.06 |
| Phaeothecoidea | 0 ± 0 | 0 ± 0 | 0 ± 0 | 0 ± 0 | 0 ± 0 | 0 ± 0 | 0 ± 0 | 2 ± 2 | 0 ± 0 | 0 ± 0 |
| Phaeotremella | 0 ± 0 | 0 ± 0 | 0 ± 0 | 0 ± 0 | 5.67 ± 5.67 | 0.33 ± 0.33 | 0 ± 0 | 0 ± 0 | 0.67 ± 0.67 | 0.67 ± 0.67 |
| Phellinocrescentia | 0 ± 0 | 0 ± 0 | 0 ± 0 | 0 ± 0 | 0 ± 0 | 0 ± 0 | 0 ± 0 | 0.67 ± 0.67 | 0 ± 0 | 0 ± 0 |
| Phialea | 0.33 ± 0.33 | 0 ± 0 | 3.67 ± 3.18 | 0.33 ± 0.33 | 0.33 ± 0.33 | 2.67 ± 1.76 | 0.33 ± 0.33 | 0 ± 0 | 0 ± 0 | 1.67 ± 1.67 |
| Phialemoniopsis | 0 ± 0 | 0 ± 0 | 0 ± 0 | 0 ± 0 | 0 ± 0 | 0 ± 0 | 0.67 ± 0.67 | 0 ± 0 | 0 ± 0 | 0 ± 0 |
| Phialemonium | 0 ± 0 | 0.33 ± 0.33 | 4.33 ± 3.38 | 3.67 ± 2.19 | 1.33 ± 0.33 | 30.33 ± 14.26 | 8.33 ± 4.18 | 2.67 ± 2.19 | 1 ± 1 | 3.67 ± 3.18 |
| Phialocephala | 2.67 ± 1.33 | 0 ± 0 | 2.67 ± 1.2 | 0 ± 0 | 1.33 ± 0.88 | 2 ± 1.53 | 1.33 ± 1.33 | 1.33 ± 0.33 | 0.33 ± 0.33 | 13 ± 6.35 |
| Phialomyces | 0 ± 0 | 0 ± 0 | 0 ± 0 | 0 ± 0 | 0 ± 0 | 0 ± 0 | 0 ± 0 | 0 ± 0 | 0 ± 0 | 4.67 ± 2.91 |
| Phialophora | 56.67 ± 18.98 | 3.33 ± 0.88 | 102.67 ± 7.22 | 58.33 ± 19.06 | 57.67 ± 13.2 | 66.67 ± 42.73 | 133.67 ± 32.67 | 441.33 ± 218.7 | 118.67 ± 114.17 | 16 ± 2.65 |
| Phlebia | 0 ± 0 | 0 ± 0 | 0.33 ± 0.33 | 0 ± 0 | 0 ± 0 | 0 ± 0 | 0 ± 0 | 0 ± 0 | 0 ± 0 | 0 ± 0 |
| Phlebiella | 0.33 ± 0.33 | 0 ± 0 | 0 ± 0 | 0 ± 0 | 0 ± 0 | 1 ± 1 | 0 ± 0 | 0 ± 0 | 0 ± 0 | 8.67 ± 8.67 |
| Phloeomana | 0 ± 0 | 0.33 ± 0.33 | 0 ± 0 | 0 ± 0 | 0 ± 0 | 3.67 ± 3.67 | 2 ± 2 | 0 ± 0 | 0 ± 0 | 0.33 ± 0.33 |
| Phlyctema | 0 ± 0 | 0 ± 0 | 0 ± 0 | 0 ± 0 | 0 ± 0 | 1 ± 1 | 0 ± 0 | 0 ± 0 | 0.33 ± 0.33 | 0 ± 0 |
| Pholiota | 0 ± 0 | 0 ± 0 | 0 ± 0 | 0 ± 0 | 0 ± 0 | 0 ± 0 | 0 ± 0 | 0 ± 0 | 3 ± 1.53 | 0 ± 0 |
| Pholiotina | 0 ± 0 | 0 ± 0 | 0 ± 0 | 0 ± 0 | 0 ± 0 | 0 ± 0 | 0.33 ± 0.33 | 0 ± 0 | 0 ± 0 | 0 ± 0 |
| Phoma | 22.33 ± 7.88 | 51.67 ± 18.11 | 367.67 ± 68.02 | 10.67 ± 3.71 | 75.33 ± 41.43 | 150.67 ± 68.08 | 40.67 ± 11.78 | 52.67 ± 21.54 | 47.67 ± 41.19 | 59 ± 6.81 |
| Phomatospora | 0.67 ± 0.67 | 0 ± 0 | 13.67 ± 2.33 | 1.33 ± 0.33 | 0.67 ± 0.67 | 14.67 ± 3.33 | 3 ± 2.08 | 3.67 ± 1.76 | 0.33 ± 0.33 | 13 ± 11.53 |
| Phomopsis | 0 ± 0 | 0 ± 0 | 0 ± 0 | 0.33 ± 0.33 | 0.67 ± 0.67 | 0 ± 0 | 2 ± 2 | 0 ± 0 | 0 ± 0 | 2 ± 1.15 |
| Phragmocephala | 0.67 ± 0.67 | 0.33 ± 0.33 | 8.67 ± 6.33 | 0 ± 0 | 0.33 ± 0.33 | 2 ± 0.58 | 2 ± 1.53 | 0 ± 0 | 1.33 ± 1.33 | 0 ± 0 |
| Picipes | 0 ± 0 | 5.67 ± 4.7 | 0 ± 0 | 0.67 ± 0.33 | 0 ± 0 | 0 ± 0 | 1.33 ± 1.33 | 0 ± 0 | 0 ± 0 | 0 ± 0 |
| Pilidium | 0 ± 0 | 29 ± 3.79 | 2.33 ± 2.33 | 0 ± 0 | 0 ± 0 | 0.67 ± 0.67 | 3.67 ± 2.03 | 3.67 ± 2.73 | 7 ± 7 | 0 ± 0 |
| Piloderma | 0 ± 0 | 0 ± 0 | 0 ± 0 | 0 ± 0 | 0 ± 0 | 0 ± 0 | 0 ± 0 | 0 ± 0 | 0 ± 0 | 0 ± 0 |
| Piskurozyma | 0 ± 0 | 0 ± 0 | 2.33 ± 1.45 | 0 ± 0 | 0 ± 0 | 0.33 ± 0.33 | 0 ± 0 | 3.67 ± 1.86 | 8.67 ± 8.67 | 0.67 ± 0.67 |
| Pithomyces | 289.33 ± 265.36 | 393.33 ± 180.07 | 49 ± 28.05 | 39.67 ± 25 | 53.67 ± 33.79 | 53.33 ± 16.83 | 129.67 ± 82.66 | 19.67 ± 11.72 | 30.33 ± 28.83 | 13 ± 6.81 |
| Placopyrenium | 0 ± 0 | 5 ± 3.61 | 3.67 ± 3.67 | 110.33 ± 49.33 | 0.33 ± 0.33 | 0 ± 0 | 53 ± 26.51 | 1.33 ± 1.33 | 0.33 ± 0.33 | 0.67 ± 0.67 |
| Plectania | 0 ± 0 | 0 ± 0 | 0.33 ± 0.33 | 0 ± 0 | 0 ± 0 | 0.33 ± 0.33 | 0 ± 0 | 0 ± 0 | 0 ± 0 | 0 ± 0 |
| Plectosphaerella | 23.33 ± 5.49 | 29 ± 8.72 | 535 ± 60.06 | 128 ± 25.06 | 167.67 ± 41.9 | 1163.33 ± 237.98 | 305.33 ± 33.01 | 125 ± 63.69 | 63 ± 60.52 | 1191.67 ± 1006.1 |
| Plenodomus | 0 ± 0 | 0 ± 0 | 0 ± 0 | 0.33 ± 0.33 | 0 ± 0 | 0 ± 0 | 0 ± 0 | 0 ± 0 | 0 ± 0 | 0 ± 0 |
| Pleotrichocladium | 0 ± 0 | 2.67 ± 1.2 | 0 ± 0 | 4.67 ± 1.76 | 2 ± 1.15 | 5.67 ± 3.71 | 8.67 ± 4.7 | 11 ± 6.81 | 11.67 ± 1.86 | 57.67 ± 15.71 |
| Pleuroascus | 0.33 ± 0.33 | 9.33 ± 3.28 | 7.33 ± 3.93 | 151 ± 35.59 | 1 ± 0.58 | 26.33 ± 4.81 | 89 ± 56.93 | 0 ± 0 | 0 ± 0 | 3.67 ± 1.86 |
| Pleurotheciella | 0 ± 0 | 0 ± 0 | 0.67 ± 0.33 | 0 ± 0 | 0 ± 0 | 1.33 ± 1.33 | 0.67 ± 0.67 | 0 ± 0 | 0.67 ± 0.67 | 0 ± 0 |
| Pluteus | 0.33 ± 0.33 | 0 ± 0 | 15.67 ± 13.72 | 0.33 ± 0.33 | 0.33 ± 0.33 | 7 ± 7 | 2 ± 1.53 | 0.33 ± 0.33 | 1 ± 0.58 | 0 ± 0 |
| Poaceascoma | 0.33 ± 0.33 | 48.33 ± 16.76 | 0 ± 0 | 2 ± 0.58 | 0 ± 0 | 2 ± 1.53 | 12 ± 7.94 | 90 ± 84.56 | 4 ± 1.73 | 4.33 ± 3.84 |
| Pochonia | 0 ± 0 | 47.67 ± 19.53 | 51.33 ± 7.88 | 54.67 ± 24.52 | 8 ± 2.89 | 20.33 ± 2.73 | 84.33 ± 17.74 | 30 ± 15.01 | 22.33 ± 11.39 | 212.33 ± 75.17 |
| Podospora | 17 ± 11 | 22 ± 8.14 | 7742 ± 493.3 | 100.33 ± 23.67 | 65 ± 19.92 | 2119.33 ± 1472.53 | 303.33 ± 30.12 | 40 ± 6 | 51.67 ± 18.41 | 1164.33 ± 396.09 |
| Polycephalomyces | 0 ± 0 | 0 ± 0 | 0 ± 0 | 0 ± 0 | 3.67 ± 2.03 | 0.67 ± 0.67 | 0 ± 0 | 0 ± 0 | 0 ± 0 | 0.67 ± 0.67 |
| Polyphilus | 1.33 ± 0.88 | 32.67 ± 20.5 | 82.33 ± 50.63 | 21.67 ± 9.94 | 2 ± 0.58 | 77 ± 7.02 | 37.33 ± 11.17 | 2.67 ± 1.45 | 5 ± 0 | 9.33 ± 5.84 |
| Polyporus | 0 ± 0 | 0 ± 0 | 0.67 ± 0.67 | 0 ± 0 | 0 ± 0 | 0 ± 0 | 0 ± 0 | 0 ± 0 | 0 ± 0 | 0 ± 0 |
| Porodiplodia | 23 ± 5.51 | 0 ± 0 | 20 ± 4.93 | 0.67 ± 0.33 | 7 ± 4.04 | 8.33 ± 4.33 | 34.67 ± 28.67 | 2.33 ± 1.2 | 0.67 ± 0.67 | 3 ± 0.58 |
| Praetumpfia | 0 ± 0 | 0 ± 0 | 0 ± 0 | 0 ± 0 | 0 ± 0 | 0 ± 0 | 1 ± 1 | 0 ± 0 | 0 ± 0 | 0 ± 0 |
| Preussia | 0.67 ± 0.33 | 46.67 ± 16.56 | 28.67 ± 5.36 | 1 ± 1 | 1.33 ± 0.88 | 8 ± 4.51 | 4.67 ± 2.33 | 1.67 ± 1.67 | 0.33 ± 0.33 | 1.67 ± 1.2 |
| Protocrea | 0 ± 0 | 0 ± 0 | 1.67 ± 1.67 | 0 ± 0 | 0 ± 0 | 2.33 ± 2.33 | 0 ± 0 | 2.67 ± 1.45 | 0 ± 0 | 0 ± 0 |
| Protoventuria | 0 ± 0 | 0.33 ± 0.33 | 50.33 ± 6.57 | 5 ± 3.06 | 0.33 ± 0.33 | 7 ± 4.73 | 4.33 ± 2.33 | 1 ± 1 | 0.33 ± 0.33 | 0 ± 0 |
| Psathyrella | 0.67 ± 0.67 | 0 ± 0 | 1.33 ± 1.33 | 0.67 ± 0.67 | 0 ± 0 | 0.33 ± 0.33 | 3.67 ± 3.67 | 0.67 ± 0.67 | 2 ± 1.53 | 5.33 ± 2.6 |
| Pseudaleuria | 7.33 ± 2.91 | 0 ± 0 | 1.67 ± 1.67 | 0 ± 0 | 4.33 ± 1.86 | 6 ± 3.21 | 0 ± 0 | 3.33 ± 2.85 | 0 ± 0 | 3.33 ± 3.33 |
| Pseudeurotium | 1 ± 0.58 | 34 ± 22.11 | 0.33 ± 0.33 | 0.33 ± 0.33 | 0.33 ± 0.33 | 0.33 ± 0.33 | 4.33 ± 2.19 | 0.33 ± 0.33 | 2.67 ± 2.19 | 3 ± 1.53 |
| Pseudoanungitea | 0 ± 0 | 0 ± 0 | 0 ± 0 | 0 ± 0 | 0 ± 0 | 1.33 ± 0.33 | 0 ± 0 | 0 ± 0 | 2 ± 2 | 1 ± 0.58 |
| Pseudoboubovia | 0 ± 0 | 0 ± 0 | 0 ± 0 | 0 ± 0 | 0 ± 0 | 0 ± 0 | 0 ± 0 | 27.67 ± 15.88 | 11 ± 11 | 0 ± 0 |
| Pseudocatenomycopsis | 1 ± 1 | 0 ± 0 | 0 ± 0 | 0 ± 0 | 0 ± 0 | 0 ± 0 | 0 ± 0 | 0 ± 0 | 0 ± 0 | 0 ± 0 |
| Pseudocercospora | 0 ± 0 | 0 ± 0 | 6 ± 6 | 0.67 ± 0.67 | 5 ± 2 | 0 ± 0 | 1 ± 0.58 | 0.67 ± 0.33 | 7.33 ± 6.84 | 1 ± 0.58 |
| Pseudoclathrosphaerina | 0 ± 0 | 0 ± 0 | 0 ± 0 | 0 ± 0 | 0 ± 0 | 0 ± 0 | 0 ± 0 | 2.33 ± 1.2 | 0 ± 0 | 0 ± 0 |
| Pseudocoleophoma | 0.67 ± 0.67 | 0.33 ± 0.33 | 0.33 ± 0.33 | 0.67 ± 0.67 | 3.33 ± 1.33 | 10.67 ± 1.76 | 5.33 ± 2.03 | 18 ± 10.79 | 11.33 ± 11.33 | 13.67 ± 7.22 |
| Pseudocosmospora | 0 ± 0 | 0 ± 0 | 0.67 ± 0.67 | 0 ± 0 | 0 ± 0 | 9.33 ± 4.81 | 2.33 ± 1.86 | 0 ± 0 | 0.33 ± 0.33 | 0.67 ± 0.67 |
| Pseudodictyosporium | 0 ± 0 | 0 ± 0 | 0 ± 0 | 0 ± 0 | 0 ± 0 | 0 ± 0 | 0 ± 0 | 1.67 ± 1.67 | 1 ± 1 | 0 ± 0 |
| Pseudogymnoascus | 0 ± 0 | 0 ± 0 | 0 ± 0 | 0.33 ± 0.33 | 0 ± 0 | 0.67 ± 0.67 | 2.33 ± 2.33 | 0 ± 0 | 0.33 ± 0.33 | 0 ± 0 |
| Pseudohyphozyma | 0 ± 0 | 0 ± 0 | 0.67 ± 0.67 | 0 ± 0 | 0 ± 0 | 0 ± 0 | 0 ± 0 | 0 ± 0 | 0 ± 0 | 0 ± 0 |
| Pseudomeria | 0 ± 0 | 0 ± 0 | 0 ± 0 | 0 ± 0 | 0 ± 0 | 0 ± 0 | 0 ± 0 | 0 ± 0 | 0 ± 0 | 0 ± 0 |
| Pseudoophiobolus | 0 ± 0 | 0.67 ± 0.67 | 0 ± 0 | 0 ± 0 | 0 ± 0 | 0 ± 0 | 0.33 ± 0.33 | 0 ± 0 | 0 ± 0 | 0 ± 0 |
| Pseudopestalotiopsis | 0.33 ± 0.33 | 0 ± 0 | 190.33 ± 32.43 | 1.33 ± 0.88 | 0.67 ± 0.33 | 35 ± 27.84 | 2.33 ± 1.2 | 0.67 ± 0.67 | 0 ± 0 | 0 ± 0 |
| Pseudopithomyces | 0.33 ± 0.33 | 9 ± 4.93 | 1.67 ± 1.67 | 0.33 ± 0.33 | 0 ± 0 | 0.33 ± 0.33 | 2.33 ± 2.33 | 0 ± 0 | 0 ± 0 | 0 ± 0 |
| Pseudopyricularia | 0 ± 0 | 0 ± 0 | 0 ± 0 | 0 ± 0 | 0 ± 0 | 0 ± 0 | 0 ± 0 | 0 ± 0 | 0 ± 0 | 0.33 ± 0.33 |
| Pseudorobillarda | 2 ± 1.53 | 0 ± 0 | 0 ± 0 | 0 ± 0 | 0 ± 0 | 0 ± 0 | 0 ± 0 | 0 ± 0 | 0 ± 0 | 0 ± 0 |
| Pseudosigmoidea | 0 ± 0 | 0 ± 0 | 0 ± 0 | 1 ± 1 | 0 ± 0 | 0 ± 0 | 2.33 ± 0.88 | 0 ± 0 | 0 ± 0 | 4.33 ± 2.96 |
| Pseudospiropes | 0 ± 0 | 0 ± 0 | 1.67 ± 1.67 | 0 ± 0 | 0 ± 0 | 1.33 ± 1.33 | 0 ± 0 | 7.33 ± 7.33 | 0 ± 0 | 0 ± 0 |
| Pseudoteratosphaeria | 0 ± 0 | 0 ± 0 | 0 ± 0 | 0 ± 0 | 0 ± 0 | 0 ± 0 | 0 ± 0 | 1 ± 1 | 4.67 ± 4.18 | 3 ± 3 |
| Pseudozyma | 0 ± 0 | 0 ± 0 | 1 ± 1 | 0 ± 0 | 0.33 ± 0.33 | 0.33 ± 0.33 | 0 ± 0 | 0 ± 0 | 0 ± 0 | 0 ± 0 |
| Psilocybe | 3.33 ± 2.85 | 0 ± 0 | 0.67 ± 0.67 | 0 ± 0 | 0.67 ± 0.67 | 0 ± 0 | 0 ± 0 | 0.33 ± 0.33 | 0 ± 0 | 3 ± 3 |
| Psoroglaena | 0 ± 0 | 0 ± 0 | 0 ± 0 | 3 ± 3 | 0.67 ± 0.33 | 0.67 ± 0.67 | 2 ± 1.53 | 1.33 ± 0.88 | 6.33 ± 2.03 | 0 ± 0 |
| Pterula | 0 ± 0 | 0 ± 0 | 0 ± 0 | 0 ± 0 | 0 ± 0 | 0 ± 0 | 1 ± 1 | 0 ± 0 | 0 ± 0 | 0 ± 0 |
| Pulvinula | 15 ± 5.77 | 0 ± 0 | 3.67 ± 3.67 | 1 ± 0.58 | 2 ± 0.58 | 0.67 ± 0.67 | 6.67 ± 5.7 | 457 ± 432.02 | 552 ± 320.86 | 1.33 ± 0.33 |
| Purpureocillium | 20.67 ± 2.96 | 166.33 ± 51.16 | 114.33 ± 31.23 | 66.67 ± 31.92 | 68.33 ± 22.82 | 184.33 ± 50.21 | 301 ± 108.17 | 1269.67 ± 937.48 | 737.33 ± 655.96 | 212 ± 66.61 |
| Pycnidiophora | 0 ± 0 | 8 ± 3.61 | 0 ± 0 | 0.33 ± 0.33 | 0 ± 0 | 0 ± 0 | 1.33 ± 1.33 | 0 ± 0 | 0 ± 0 | 0 ± 0 |
| Pyrenochaeta | 9.67 ± 5.17 | 8 ± 3.46 | 32.33 ± 6.67 | 24.33 ± 17.89 | 52.33 ± 34.19 | 56.67 ± 7.88 | 59.67 ± 41.53 | 8.33 ± 6.36 | 16.33 ± 2.33 | 73 ± 9.45 |
| Pyrenochaetopsis | 83 ± 41.58 | 248 ± 105.84 | 16.67 ± 11.22 | 34.67 ± 12.33 | 148.67 ± 108.79 | 95.67 ± 12.35 | 312.33 ± 180.51 | 129.33 ± 63.26 | 75 ± 56.24 | 33 ± 4.36 |
| Pyrenophora | 0 ± 0 | 0 ± 0 | 12.33 ± 12.33 | 0.33 ± 0.33 | 0 ± 0 | 6.67 ± 6.67 | 0 ± 0 | 0.33 ± 0.33 | 0 ± 0 | 0 ± 0 |
| Pyricularia | 0 ± 0 | 0 ± 0 | 0 ± 0 | 0 ± 0 | 0 ± 0 | 0 ± 0 | 0 ± 0 | 2.67 ± 2.67 | 0 ± 0 | 0 ± 0 |
| Pyrigemmula | 0.33 ± 0.33 | 8 ± 2.52 | 1.33 ± 1.33 | 1.33 ± 0.33 | 0.67 ± 0.67 | 3.33 ± 3.33 | 2.67 ± 2.19 | 0 ± 0 | 0 ± 0 | 0 ± 0 |
| Pyxidiophora | 1.33 ± 1.33 | 0 ± 0 | 2.33 ± 1.2 | 0.67 ± 0.67 | 0.67 ± 0.67 | 41 ± 22.14 | 0.33 ± 0.33 | 1.33 ± 1.33 | 1.33 ± 1.33 | 1.67 ± 0.88 |
| Quadricrura | 0 ± 0 | 0 ± 0 | 0 ± 0 | 0 ± 0 | 1.67 ± 0.88 | 0 ± 0 | 0.33 ± 0.33 | 3.67 ± 3.67 | 0.33 ± 0.33 | 0 ± 0 |
| Rachicladosporium | 0.33 ± 0.33 | 0 ± 0 | 0 ± 0 | 0 ± 0 | 0 ± 0 | 0.33 ± 0.33 | 0 ± 0 | 1.33 ± 0.67 | 1.33 ± 1.33 | 6.33 ± 4.91 |
| Racocetra | 24.33 ± 7.45 | 4.33 ± 1.86 | 5.67 ± 5.17 | 21.67 ± 18.17 | 44.33 ± 8.17 | 14 ± 10.15 | 10 ± 3.51 | 54.33 ± 31.62 | 22.33 ± 19.84 | 18 ± 6.51 |
| Radulomyces | 0 ± 0 | 0 ± 0 | 0 ± 0 | 0 ± 0 | 0 ± 0 | 0 ± 0 | 0 ± 0 | 1 ± 1 | 0 ± 0 | 0.33 ± 0.33 |
| Ragnhildiana | 0 ± 0 | 0 ± 0 | 0 ± 0 | 0 ± 0 | 0 ± 0 | 0 ± 0 | 1 ± 1 | 115.67 ± 91.81 | 34.33 ± 33.83 | 2 ± 1.53 |
| Ramariopsis | 16.67 ± 9.02 | 2 ± 1.53 | 42.67 ± 8.41 | 46 ± 23.18 | 0.67 ± 0.33 | 14 ± 8.08 | 24.33 ± 7.84 | 0 ± 0 | 0.67 ± 0.67 | 6.33 ± 6.33 |
| Ramgea | 2.67 ± 1.76 | 1 ± 0.58 | 1115.67 ± 569.77 | 9.33 ± 3.18 | 7.67 ± 2.03 | 138.33 ± 105.29 | 40 ± 23.09 | 0.33 ± 0.33 | 1 ± 1 | 1.67 ± 1.67 |
| Ramicandelaber | 0 ± 0 | 0 ± 0 | 0 ± 0 | 0 ± 0 | 0 ± 0 | 0 ± 0 | 0 ± 0 | 0 ± 0 | 3.67 ± 3.67 | 0 ± 0 |
| Ramichloridium | 0.67 ± 0.67 | 0 ± 0 | 0 ± 0 | 0.67 ± 0.67 | 0 ± 0 | 0 ± 0 | 1 ± 1 | 4.67 ± 0.33 | 4 ± 1.15 | 0 ± 0 |
| Ramophialophora | 67.67 ± 15.62 | 0.67 ± 0.67 | 13.33 ± 3.48 | 1.33 ± 1.33 | 62.33 ± 14.31 | 11 ± 8.19 | 6.33 ± 5.36 | 0 ± 0 | 0 ± 0 | 0.67 ± 0.67 |
| Ramulariopsis | 0 ± 0 | 0.67 ± 0.33 | 41.67 ± 17.7 | 1 ± 1 | 0 ± 0 | 5 ± 3.61 | 0 ± 0 | 2.33 ± 2.33 | 0 ± 0 | 0.67 ± 0.67 |
| Rasamsonia | 0 ± 0 | 0 ± 0 | 0 ± 0 | 0 ± 0 | 0 ± 0 | 0 ± 0 | 0 ± 0 | 0 ± 0 | 0.33 ± 0.33 | 0 ± 0 |
| Remersonia | 0 ± 0 | 0 ± 0 | 0 ± 0 | 0 ± 0 | 0 ± 0 | 0 ± 0 | 0 ± 0 | 7.67 ± 5.78 | 0 ± 0 | 0 ± 0 |
| Repetobasidium | 0 ± 0 | 0 ± 0 | 0 ± 0 | 0 ± 0 | 0 ± 0 | 0 ± 0 | 0.33 ± 0.33 | 7.67 ± 4.06 | 0.33 ± 0.33 | 0 ± 0 |
| Rhexodenticula | 0 ± 0 | 0.67 ± 0.33 | 0 ± 0 | 0 ± 0 | 0.33 ± 0.33 | 0 ± 0 | 0 ± 0 | 0.33 ± 0.33 | 0 ± 0 | 0 ± 0 |
| Rhinocladiella | 0.33 ± 0.33 | 4 ± 4 | 8 ± 2.89 | 0.67 ± 0.33 | 0.67 ± 0.67 | 18.33 ± 7.22 | 27 ± 25.01 | 1 ± 0.58 | 1.67 ± 0.88 | 1 ± 0.58 |
| Rhizocarpon | 0 ± 0 | 0.33 ± 0.33 | 0 ± 0 | 0 ± 0 | 0 ± 0 | 0 ± 0 | 0 ± 0 | 0 ± 0 | 0 ± 0 | 0 ± 0 |
| Rhizoctonia | 13.33 ± 6.96 | 0 ± 0 | 117.33 ± 111.37 | 0.33 ± 0.33 | 4.67 ± 1.86 | 4 ± 3.06 | 7 ± 3.61 | 2 ± 2 | 1.33 ± 1.33 | 2 ± 1.15 |
| Rhizophagus | 18 ± 7 | 171.67 ± 28.29 | 0 ± 0 | 8 ± 3.21 | 4.33 ± 1.33 | 0.33 ± 0.33 | 22.33 ± 11.46 | 2.33 ± 1.2 | 3.67 ± 0.88 | 3.33 ± 1.2 |
| Rhizophlyctis | 4.33 ± 4.33 | 0 ± 0 | 1.33 ± 0.88 | 0.33 ± 0.33 | 0.33 ± 0.33 | 0.33 ± 0.33 | 0 ± 0 | 0 ± 0 | 29 ± 29 | 5.33 ± 3.18 |
| Rhizophydium | 0 ± 0 | 0 ± 0 | 0 ± 0 | 0 ± 0 | 0 ± 0 | 0 ± 0 | 0 ± 0 | 2 ± 1 | 1.67 ± 1.67 | 0 ± 0 |
| Rhizopycnis | 0.67 ± 0.67 | 0 ± 0 | 0.33 ± 0.33 | 0 ± 0 | 0 ± 0 | 6 ± 3.79 | 0 ± 0 | 0 ± 0 | 0 ± 0 | 0 ± 0 |
| Rhizoscyphus | 0 ± 0 | 1.33 ± 0.88 | 0 ± 0 | 5.33 ± 5.33 | 0 ± 0 | 0 ± 0 | 1.67 ± 1.67 | 0 ± 0 | 0 ± 0 | 0 ± 0 |
| Rhodosporidiobolus | 9 ± 9 | 2.33 ± 0.33 | 8 ± 6.11 | 0 ± 0 | 6 ± 0 | 1.67 ± 1.2 | 1.33 ± 1.33 | 1.67 ± 1.2 | 1 ± 0.58 | 6 ± 1 |
| Rhodotorula | 0 ± 0 | 0 ± 0 | 2.67 ± 1.76 | 0.33 ± 0.33 | 0 ± 0 | 3.33 ± 2.03 | 5.33 ± 3.93 | 4 ± 2.31 | 0.67 ± 0.67 | 13.33 ± 7.8 |
| Rigidoporus | 0.33 ± 0.33 | 0 ± 0 | 0 ± 0 | 0 ± 0 | 4.33 ± 1.86 | 0 ± 0 | 0 ± 0 | 24.67 ± 24.67 | 1.67 ± 1.67 | 0 ± 0 |
| Robillarda | 0 ± 0 | 8 ± 3.79 | 0 ± 0 | 0 ± 0 | 0 ± 0 | 0 ± 0 | 0.67 ± 0.67 | 0 ± 0 | 0 ± 0 | 1 ± 1 |
| Roesleria | 0 ± 0 | 0 ± 0 | 0 ± 0 | 0 ± 0 | 0 ± 0 | 0 ± 0 | 0.33 ± 0.33 | 0 ± 0 | 0 ± 0 | 0 ± 0 |
| Rosasphaeria | 0 ± 0 | 0 ± 0 | 0 ± 0 | 0 ± 0 | 0 ± 0 | 0 ± 0 | 0 ± 0 | 0 ± 0 | 0 ± 0 | 0 ± 0 |
| Rosellinia | 0 ± 0 | 0 ± 0 | 0 ± 0 | 0 ± 0 | 0 ± 0 | 0.33 ± 0.33 | 0 ± 0 | 0 ± 0 | 1.67 ± 1.67 | 0 ± 0 |
| Roseodiscus | 0 ± 0 | 0 ± 0 | 0 ± 0 | 0 ± 0 | 0 ± 0 | 0 ± 0 | 0 ± 0 | 32.33 ± 23.05 | 11.33 ± 5.84 | 0 ± 0 |
| Rotiferophthora | 0 ± 0 | 0 ± 0 | 0 ± 0 | 0 ± 0 | 0 ± 0 | 0 ± 0 | 1.33 ± 1.33 | 0 ± 0 | 0 ± 0 | 0 ± 0 |
| Roussoella | 0 ± 0 | 0 ± 0 | 0 ± 0 | 0 ± 0 | 1 ± 0.58 | 5.33 ± 3.93 | 3.67 ± 3.67 | 0 ± 0 | 0 ± 0 | 2.67 ± 0.67 |
| Rugosomyces | 3.33 ± 1.45 | 0 ± 0 | 0 ± 0 | 0 ± 0 | 1.67 ± 0.88 | 2.67 ± 2.19 | 3 ± 1.73 | 0 ± 0 | 0 ± 0 | 0.33 ± 0.33 |
| Russula | 0 ± 0 | 0 ± 0 | 3.67 ± 2.19 | 1 ± 1 | 0 ± 0 | 0 ± 0 | 24 ± 24 | 3248 ± 3193.01 | 9204 ± 4897.44 | 27.33 ± 13.68 |
| Saccharata | 0 ± 0 | 0 ± 0 | 0 ± 0 | 0.33 ± 0.33 | 0.67 ± 0.67 | 0 ± 0 | 0 ± 0 | 0 ± 0 | 0 ± 0 | 0 ± 0 |
| Sagenomella | 0 ± 0 | 5.67 ± 5.67 | 0 ± 0 | 1 ± 1 | 0 ± 0 | 2.33 ± 1.45 | 6 ± 3.06 | 0.33 ± 0.33 | 0 ± 0 | 3.33 ± 3.33 |
| Saitozyma | 9.67 ± 4.37 | 2386 ± 1287.1 | 246.33 ± 80.48 | 1156.67 ± 78.75 | 13.33 ± 2.91 | 163 ± 54.98 | 771 ± 282.37 | 275 ± 85.5 | 334.33 ± 87.19 | 5976.33 ± 1482.18 |
| Sakaguchia | 0 ± 0 | 0 ± 0 | 0.33 ± 0.33 | 0 ± 0 | 0 ± 0 | 0 ± 0 | 0 ± 0 | 0 ± 0 | 0 ± 0 | 0 ± 0 |
| Sarcinomyces | 0 ± 0 | 0 ± 0 | 7.33 ± 3.84 | 0 ± 0 | 0 ± 0 | 0.33 ± 0.33 | 1.33 ± 0.67 | 0 ± 0 | 0 ± 0 | 0 ± 0 |
| Sarcodon | 0 ± 0 | 0 ± 0 | 0 ± 0 | 0 ± 0 | 0.33 ± 0.33 | 0 ± 0 | 0 ± 0 | 0 ± 0 | 0 ± 0 | 0 ± 0 |
| Sarcopodium | 0 ± 0 | 0 ± 0 | 6.67 ± 0.88 | 0.33 ± 0.33 | 0.33 ± 0.33 | 6.33 ± 6.33 | 0 ± 0 | 0 ± 0 | 0 ± 0 | 0 ± 0 |
| Sarea | 0.67 ± 0.33 | 4 ± 2.08 | 7 ± 3.46 | 1 ± 0.58 | 0 ± 0 | 4.67 ± 4.67 | 3.67 ± 0.88 | 64 ± 59.53 | 6.33 ± 5.36 | 1 ± 1 |
| Sarocladium | 1.33 ± 0.88 | 39.33 ± 6.84 | 80 ± 15.5 | 14.67 ± 6.06 | 8.33 ± 4.33 | 16.67 ± 12.78 | 16.33 ± 8.37 | 28 ± 23.54 | 11 ± 5.57 | 12.67 ± 4.26 |
| Scedosporium | 0 ± 0 | 0 ± 0 | 0 ± 0 | 0 ± 0 | 0 ± 0 | 4.67 ± 3.28 | 0 ± 0 | 1.33 ± 0.88 | 0 ± 0 | 1.67 ± 1.67 |
| Schaereria | 0 ± 0 | 0 ± 0 | 0 ± 0 | 0 ± 0 | 0 ± 0 | 0 ± 0 | 0 ± 0 | 0.67 ± 0.67 | 1.67 ± 1.67 | 0 ± 0 |
| Schizophyllum | 2 ± 2 | 0.33 ± 0.33 | 0 ± 0 | 0 ± 0 | 0.33 ± 0.33 | 0 ± 0 | 0 ± 0 | 0 ± 0 | 0 ± 0 | 0.33 ± 0.33 |
| Schizothecium | 0.67 ± 0.67 | 488 ± 303 | 2 ± 1.15 | 10.33 ± 2.85 | 0.33 ± 0.33 | 4.67 ± 2.19 | 77.33 ± 59.69 | 10.33 ± 10.33 | 3 ± 3 | 3 ± 2.52 |
| Schizoxylon | 0 ± 0 | 2 ± 1.15 | 0 ± 0 | 0 ± 0 | 0 ± 0 | 0 ± 0 | 0 ± 0 | 0 ± 0 | 0 ± 0 | 0 ± 0 |
| Scleroderma | 0 ± 0 | 0 ± 0 | 0 ± 0 | 0 ± 0 | 0 ± 0 | 4 ± 2.65 | 4.67 ± 4.67 | 84.67 ± 84.67 | 247 ± 141.46 | 0.33 ± 0.33 |
| Sclerogaster | 0 ± 0 | 0 ± 0 | 0 ± 0 | 0 ± 0 | 0.67 ± 0.67 | 0 ± 0 | 0 ± 0 | 0 ± 0 | 0 ± 0 | 0 ± 0 |
| Scleropezicula | 2.67 ± 2.67 | 0 ± 0 | 0 ± 0 | 0 ± 0 | 0.33 ± 0.33 | 1 ± 1 | 0.33 ± 0.33 | 0 ± 0 | 0.33 ± 0.33 | 0 ± 0 |
| Scleroramularia | 0 ± 0 | 0 ± 0 | 0 ± 0 | 1.33 ± 0.67 | 0 ± 0 | 0 ± 0 | 0.33 ± 0.33 | 0 ± 0 | 0 ± 0 | 0 ± 0 |
| Sclerotinia | 0 ± 0 | 0 ± 0 | 1.67 ± 1.67 | 0 ± 0 | 0 ± 0 | 0.33 ± 0.33 | 0 ± 0 | 0 ± 0 | 0 ± 0 | 0 ± 0 |
| Scolecobasidium | 0 ± 0 | 0 ± 0 | 0 ± 0 | 0 ± 0 | 0 ± 0 | 0 ± 0 | 0 ± 0 | 0 ± 0 | 0 ± 0 | 1 ± 1 |
| Scopulariopsis | 0 ± 0 | 0 ± 0 | 0 ± 0 | 0 ± 0 | 0 ± 0 | 0.67 ± 0.67 | 0.67 ± 0.67 | 0.33 ± 0.33 | 1.67 ± 1.67 | 0 ± 0 |
| Scytalidium | 0 ± 0 | 2 ± 1.15 | 17 ± 12.5 | 0.67 ± 0.33 | 2 ± 1 | 32 ± 8.39 | 1.33 ± 0.88 | 0 ± 0 | 0.33 ± 0.33 | 12.67 ± 3.18 |
| Sebacina | 0.33 ± 0.33 | 1 ± 0.58 | 32 ± 13.08 | 0.33 ± 0.33 | 0.33 ± 0.33 | 6.67 ± 4.81 | 2.67 ± 1.45 | 30 ± 14.47 | 15.67 ± 15.17 | 2.33 ± 1.45 |
| Seimatosporium | 0 ± 0 | 0.33 ± 0.33 | 0 ± 0 | 0 ± 0 | 0 ± 0 | 0 ± 0 | 0.67 ± 0.67 | 21.33 ± 11.55 | 0.33 ± 0.33 | 0 ± 0 |
| Seiridium | 0 ± 0 | 0 ± 0 | 0 ± 0 | 0 ± 0 | 0 ± 0 | 0 ± 0 | 1.33 ± 0.88 | 0 ± 0 | 0.33 ± 0.33 | 0 ± 0 |
| Selenodriella | 1.33 ± 0.88 | 22.67 ± 14.85 | 0 ± 0 | 1 ± 1 | 0.33 ± 0.33 | 0 ± 0 | 5.33 ± 2.6 | 0 ± 0 | 0 ± 0 | 1.33 ± 1.33 |
| Sepedonium | 0 ± 0 | 0 ± 0 | 8 ± 7.51 | 1 ± 1 | 0 ± 0 | 1 ± 1 | 0 ± 0 | 0 ± 0 | 0 ± 0 | 0 ± 0 |
| Septobasidium | 0 ± 0 | 0 ± 0 | 2.67 ± 2.67 | 0 ± 0 | 0 ± 0 | 0.33 ± 0.33 | 0 ± 0 | 0 ± 0 | 0.33 ± 0.33 | 3.67 ± 2.03 |
| Septofusidium | 0 ± 0 | 0 ± 0 | 0.33 ± 0.33 | 0 ± 0 | 0 ± 0 | 5.67 ± 2.96 | 0 ± 0 | 0 ± 0 | 0 ± 0 | 0 ± 0 |
| Septoglomus | 2.33 ± 0.33 | 0 ± 0 | 0.33 ± 0.33 | 2 ± 1.53 | 7.33 ± 4.84 | 0 ± 0 | 0.33 ± 0.33 | 6 ± 2.31 | 5 ± 2 | 67.33 ± 30.6 |
| Septoria | 5.33 ± 2.85 | 0.67 ± 0.67 | 93.33 ± 6.84 | 11.33 ± 2.96 | 17.67 ± 3.38 | 85.67 ± 44.73 | 101.33 ± 22.98 | 147.67 ± 114.36 | 12.67 ± 11.2 | 52.67 ± 19.1 |
| Septoriella | 1.67 ± 0.33 | 0 ± 0 | 3.33 ± 0.33 | 0 ± 0 | 0 ± 0 | 4.67 ± 3.71 | 0.33 ± 0.33 | 0.67 ± 0.67 | 0 ± 0 | 6.33 ± 1.86 |
| Serendipita | 1.33 ± 0.88 | 4.33 ± 2.96 | 4.67 ± 4.67 | 2 ± 0.58 | 5.67 ± 3.48 | 2 ± 1.15 | 1.33 ± 0.67 | 23.67 ± 2.91 | 7 ± 1.53 | 355.67 ± 167.3 |
| Setophaeosphaeria | 0.67 ± 0.33 | 0.33 ± 0.33 | 4.67 ± 3.28 | 0 ± 0 | 2.67 ± 2.19 | 3.67 ± 1.86 | 7.67 ± 7.17 | 4.33 ± 2.85 | 7 ± 6.03 | 12 ± 5.51 |
| Setophoma | 0 ± 0 | 0 ± 0 | 0 ± 0 | 1 ± 1 | 0 ± 0 | 0.67 ± 0.67 | 1 ± 1 | 0 ± 0 | 0 ± 0 | 3 ± 2.08 |
| Shiraia | 0 ± 0 | 0 ± 0 | 0 ± 0 | 0 ± 0 | 0 ± 0 | 0 ± 0 | 0 ± 0 | 0 ± 0 | 0 ± 0 | 4.67 ± 4.67 |
| Sigarispora | 0 ± 0 | 0 ± 0 | 0 ± 0 | 0 ± 0 | 0 ± 0 | 1.67 ± 1.2 | 0 ± 0 | 0 ± 0 | 0 ± 0 | 0 ± 0 |
| Simocybe | 0.67 ± 0.67 | 0 ± 0 | 0.33 ± 0.33 | 0 ± 0 | 0 ± 0 | 0.33 ± 0.33 | 0 ± 0 | 0.33 ± 0.33 | 2.67 ± 2.67 | 0 ± 0 |
| Simplicillium | 0 ± 0 | 0.33 ± 0.33 | 0.67 ± 0.67 | 0.67 ± 0.33 | 1.67 ± 0.88 | 1 ± 0.58 | 4.67 ± 2.03 | 9.33 ± 5.21 | 18.67 ± 16.67 | 10 ± 3.46 |
| Sirastachys | 0 ± 0 | 0 ± 0 | 0.33 ± 0.33 | 0 ± 0 | 0 ± 0 | 1 ± 0.58 | 18.33 ± 18.33 | 0 ± 0 | 0 ± 0 | 0.67 ± 0.33 |
| Sirobasidium | 0 ± 0 | 0 ± 0 | 18.33 ± 18.33 | 0 ± 0 | 0 ± 0 | 0.67 ± 0.67 | 0 ± 0 | 0 ± 0 | 0 ± 0 | 0.67 ± 0.67 |
| Sirotrema | 0 ± 0 | 0 ± 0 | 0 ± 0 | 0 ± 0 | 0 ± 0 | 0 ± 0 | 0 ± 0 | 0 ± 0 | 1.67 ± 1.67 | 0 ± 0 |
| Sistotrema | 0 ± 0 | 0 ± 0 | 0 ± 0 | 6.33 ± 2.33 | 0 ± 0 | 0 ± 0 | 1.67 ± 1.2 | 0 ± 0 | 0 ± 0 | 0 ± 0 |
| Slimacomyces | 0 ± 0 | 131 ± 74.33 | 1.33 ± 0.67 | 3.67 ± 1.76 | 0.33 ± 0.33 | 0.67 ± 0.67 | 20 ± 18.52 | 0 ± 0 | 0 ± 0 | 0 ± 0 |
| Slooffia | 0 ± 0 | 0 ± 0 | 0 ± 0 | 0 ± 0 | 0 ± 0 | 0 ± 0 | 0 ± 0 | 15 ± 10.02 | 6 ± 6 | 0 ± 0 |
| Solicoccozyma | 1.33 ± 0.88 | 186.33 ± 57.55 | 485 ± 308.55 | 49.67 ± 28.94 | 2 ± 0.58 | 113 ± 102 | 37.67 ± 16.13 | 99.67 ± 59.12 | 83.33 ± 77.33 | 172.33 ± 87.52 |
| Sonoraphlyctis | 0 ± 0 | 9.67 ± 9.67 | 0 ± 0 | 0.67 ± 0.67 | 0 ± 0 | 0.33 ± 0.33 | 2 ± 2 | 0 ± 0 | 0 ± 0 | 0 ± 0 |
| Sordaria | 1.67 ± 0.88 | 18 ± 8.72 | 6.67 ± 6.67 | 1.67 ± 0.67 | 0 ± 0 | 17.67 ± 8.95 | 2.67 ± 1.76 | 1.67 ± 0.88 | 14.33 ± 10.35 | 9 ± 9 |
| Spadicoides | 0 ± 0 | 0 ± 0 | 0 ± 0 | 0 ± 0 | 0 ± 0 | 0.67 ± 0.67 | 0 ± 0 | 0 ± 0 | 0 ± 0 | 0 ± 0 |
| Sphaceloma | 0 ± 0 | 0 ± 0 | 0 ± 0 | 0 ± 0 | 0 ± 0 | 1.33 ± 1.33 | 0 ± 0 | 0 ± 0 | 0 ± 0 | 0 ± 0 |
| Sphacelotheca | 0 ± 0 | 0 ± 0 | 0 ± 0 | 0 ± 0 | 0 ± 0 | 0 ± 0 | 0.33 ± 0.33 | 0 ± 0 | 0 ± 0 | 0 ± 0 |
| Sphaeropsis | 0 ± 0 | 0 ± 0 | 0 ± 0 | 0 ± 0 | 0 ± 0 | 0 ± 0 | 0 ± 0 | 0 ± 0 | 0 ± 0 | 0.67 ± 0.67 |
| Sphaerosporella | 0.33 ± 0.33 | 11.67 ± 4.67 | 22 ± 12.42 | 269 ± 50.34 | 2.33 ± 1.2 | 20 ± 3.46 | 87.67 ± 44.86 | 10.33 ± 5.33 | 8.33 ± 1.45 | 23.33 ± 23.33 |
| Sphaerostilbella | 0 ± 0 | 0 ± 0 | 0 ± 0 | 0.33 ± 0.33 | 0 ± 0 | 2.33 ± 1.86 | 0 ± 0 | 0 ± 0 | 0 ± 0 | 0 ± 0 |
| Sphaerulina | 0 ± 0 | 0 ± 0 | 0.33 ± 0.33 | 0 ± 0 | 0.33 ± 0.33 | 0 ± 0 | 0 ± 0 | 0 ± 0 | 0 ± 0 | 10 ± 4.16 |
| Spirosphaera | 8 ± 8 | 0 ± 0 | 0 ± 0 | 0 ± 0 | 1.33 ± 1.33 | 0.33 ± 0.33 | 0.33 ± 0.33 | 8.67 ± 6.33 | 1.67 ± 1.2 | 7.67 ± 4.26 |
| Spissiomyces | 0 ± 0 | 0 ± 0 | 4 ± 4 | 0 ± 0 | 0 ± 0 | 0 ± 0 | 0 ± 0 | 0 ± 0 | 0 ± 0 | 0 ± 0 |
| Spizellomyces | 0 ± 0 | 0 ± 0 | 2 ± 1.15 | 0 ± 0 | 0 ± 0 | 0.33 ± 0.33 | 1 ± 1 | 0 ± 0 | 0.33 ± 0.33 | 0.33 ± 0.33 |
| Sporidesmium | 0 ± 0 | 2 ± 2 | 1.67 ± 1.67 | 8.67 ± 3.38 | 0.33 ± 0.33 | 0 ± 0 | 12.33 ± 10.33 | 410.33 ± 207.96 | 219 ± 213 | 9.67 ± 6.12 |
| Sporobolomyces | 0 ± 0 | 0 ± 0 | 2.33 ± 0.67 | 4.67 ± 2.33 | 1.33 ± 1.33 | 1.67 ± 1.67 | 3 ± 1.73 | 0 ± 0 | 0 ± 0 | 1 ± 1 |
| Sporoschisma | 0 ± 0 | 0 ± 0 | 0 ± 0 | 0 ± 0 | 0 ± 0 | 0 ± 0 | 0 ± 0 | 0 ± 0 | 0.33 ± 0.33 | 0 ± 0 |
| Sporothrix | 0 ± 0 | 0 ± 0 | 0.33 ± 0.33 | 0 ± 0 | 0 ± 0 | 8.33 ± 5.61 | 5.33 ± 4.84 | 0.67 ± 0.67 | 2 ± 2 | 0 ± 0 |
| Stachybotryna | 0 ± 0 | 0 ± 0 | 0 ± 0 | 0 ± 0 | 0 ± 0 | 0 ± 0 | 0 ± 0 | 0 ± 0 | 4.33 ± 4.33 | 0 ± 0 |
| Stachybotrys | 0.67 ± 0.33 | 0 ± 0 | 0 ± 0 | 0 ± 0 | 0 ± 0 | 2.33 ± 1.45 | 0.33 ± 0.33 | 3.67 ± 3.67 | 0 ± 0 | 2 ± 2 |
| Stagonospora | 8.33 ± 7.33 | 1 ± 1 | 11.67 ± 6.89 | 2 ± 0.58 | 4.67 ± 2.91 | 4 ± 2.52 | 5.33 ± 3.93 | 13.33 ± 11.39 | 5.33 ± 4.37 | 12 ± 2.52 |
| Staphylotrichum | 0.67 ± 0.33 | 1.33 ± 0.67 | 0 ± 0 | 1.33 ± 0.67 | 2.33 ± 1.86 | 3 ± 2.08 | 7 ± 4.51 | 17 ± 13.58 | 0.33 ± 0.33 | 3.33 ± 3.33 |
| Staurothele | 0 ± 0 | 0 ± 0 | 0 ± 0 | 0 ± 0 | 0 ± 0 | 0 ± 0 | 0 ± 0 | 14.67 ± 10.27 | 0 ± 0 | 0 ± 0 |
| Steccherinum | 0 ± 0 | 0 ± 0 | 0 ± 0 | 0 ± 0 | 0 ± 0 | 0 ± 0 | 0 ± 0 | 0 ± 0 | 0 ± 0 | 1.67 ± 1.67 |
| Stemphylium | 0 ± 0 | 0 ± 0 | 2 ± 1.15 | 0 ± 0 | 0 ± 0 | 1 ± 1 | 0 ± 0 | 0 ± 0 | 0 ± 0 | 0 ± 0 |
| Stephanonectria | 0.67 ± 0.33 | 0 ± 0 | 1.33 ± 0.88 | 0.33 ± 0.33 | 0.33 ± 0.33 | 1.33 ± 1.33 | 0.33 ± 0.33 | 8.67 ± 6.33 | 3 ± 2.52 | 15 ± 11 |
| Stilbella | 6.33 ± 3.93 | 4.67 ± 4.18 | 2 ± 1.15 | 0.67 ± 0.67 | 1.33 ± 0.88 | 0 ± 0 | 1 ± 1 | 9.67 ± 4.84 | 10 ± 10 | 0 ± 0 |
| Stilbocrea | 0.33 ± 0.33 | 0 ± 0 | 0 ± 0 | 0 ± 0 | 2.67 ± 1.45 | 1.33 ± 1.33 | 0.67 ± 0.67 | 5.67 ± 4.26 | 4.33 ± 4.33 | 0 ± 0 |
| Stilbospora | 0 ± 0 | 0.33 ± 0.33 | 1 ± 0.58 | 0 ± 0 | 0.67 ± 0.33 | 97.67 ± 54.08 | 3.67 ± 2.03 | 0.67 ± 0.67 | 0 ± 0 | 12.67 ± 8.29 |
| Stomiopeltis | 0 ± 0 | 0 ± 0 | 0 ± 0 | 0 ± 0 | 0 ± 0 | 2 ± 2 | 0 ± 0 | 0 ± 0 | 0 ± 0 | 0 ± 0 |
| Strattonia | 0 ± 0 | 0 ± 0 | 0 ± 0 | 0.67 ± 0.67 | 0 ± 0 | 0 ± 0 | 0 ± 0 | 0.67 ± 0.67 | 0 ± 0 | 9 ± 5.86 |
| Strelitziana | 1.33 ± 1.33 | 1.67 ± 1.67 | 1 ± 1 | 2 ± 1.53 | 0 ± 0 | 1.33 ± 0.88 | 3 ± 1.53 | 1.33 ± 0.88 | 1.33 ± 1.33 | 16.33 ± 7.54 |
| Striatibotrys | 1.33 ± 0.88 | 0 ± 0 | 3 ± 2.52 | 2 ± 1.15 | 10 ± 4.73 | 11.67 ± 4.18 | 8.33 ± 4.37 | 0.33 ± 0.33 | 1.33 ± 0.67 | 78.33 ± 77.83 |
| Striaticonidium | 0.67 ± 0.67 | 1.67 ± 0.33 | 0 ± 0 | 0 ± 0 | 1.67 ± 1.67 | 0.33 ± 0.33 | 1 ± 0.58 | 0.33 ± 0.33 | 0 ± 0 | 0 ± 0 |
| Strigula | 0 ± 0 | 0 ± 0 | 0 ± 0 | 0 ± 0 | 1.33 ± 1.33 | 0 ± 0 | 0 ± 0 | 0 ± 0 | 0 ± 0 | 0 ± 0 |
| Stromatonectria | 0.67 ± 0.67 | 5.67 ± 5.67 | 0 ± 0 | 0 ± 0 | 2.67 ± 2.67 | 0 ± 0 | 2.33 ± 1.2 | 0 ± 0 | 0 ± 0 | 0 ± 0 |
| Suberoteratosphaeria | 0 ± 0 | 0 ± 0 | 0 ± 0 | 0 ± 0 | 0 ± 0 | 0.67 ± 0.67 | 0 ± 0 | 0 ± 0 | 0 ± 0 | 0 ± 0 |
| Subramaniomyces | 0 ± 0 | 0 ± 0 | 0 ± 0 | 0 ± 0 | 0 ± 0 | 0 ± 0 | 0.33 ± 0.33 | 0 ± 0 | 0 ± 0 | 0 ± 0 |
| Subramaniula | 0 ± 0 | 0 ± 0 | 0 ± 0 | 0 ± 0 | 0 ± 0 | 0.33 ± 0.33 | 0 ± 0 | 0 ± 0 | 0 ± 0 | 0 ± 0 |
| Subulicystidium | 13.33 ± 7.84 | 1 ± 0.58 | 25 ± 12.01 | 22 ± 11.24 | 471.33 ± 420.91 | 68 ± 22.27 | 110.67 ± 2.4 | 13.33 ± 6.69 | 10.33 ± 8.84 | 7.67 ± 2.03 |
| Sugitazyma | 0 ± 0 | 0 ± 0 | 0 ± 0 | 2 ± 2 | 0 ± 0 | 0 ± 0 | 0 ± 0 | 0 ± 0 | 0 ± 0 | 0 ± 0 |
| Suillellus | 0 ± 0 | 0 ± 0 | 0.33 ± 0.33 | 0 ± 0 | 0 ± 0 | 0 ± 0 | 0 ± 0 | 0 ± 0 | 0 ± 0 | 0 ± 0 |
| Sulcatispora | 0 ± 0 | 0 ± 0 | 1.33 ± 1.33 | 0.33 ± 0.33 | 0 ± 0 | 0.67 ± 0.33 | 1.33 ± 1.33 | 0 ± 0 | 0.33 ± 0.33 | 2 ± 0.58 |
| Symmetrospora | 0 ± 0 | 0 ± 0 | 13.33 ± 6.01 | 0 ± 0 | 0.33 ± 0.33 | 0 ± 0 | 0 ± 0 | 0.33 ± 0.33 | 0.33 ± 0.33 | 4.33 ± 2.4 |
| Syncephalis | 0.67 ± 0.67 | 0 ± 0 | 0 ± 0 | 0 ± 0 | 0.33 ± 0.33 | 0.33 ± 0.33 | 0.67 ± 0.67 | 0 ± 0 | 0.33 ± 0.33 | 0 ± 0 |
| Synnemadiella | 0.67 ± 0.67 | 0 ± 0 | 0 ± 0 | 0 ± 0 | 0 ± 0 | 0 ± 0 | 0 ± 0 | 0 ± 0 | 0 ± 0 | 0 ± 0 |
| Talaromyces | 1.67 ± 1.2 | 210.33 ± 22.85 | 18.67 ± 5.93 | 17 ± 4.62 | 0.33 ± 0.33 | 5.33 ± 1.45 | 61.33 ± 26.44 | 85.33 ± 42.83 | 39 ± 36.51 | 5 ± 2 |
| Taphrina | 0 ± 0 | 0 ± 0 | 0 ± 0 | 0 ± 0 | 1 ± 1 | 1 ± 1 | 6.33 ± 6.33 | 0 ± 0 | 0 ± 0 | 1.33 ± 1.33 |
| Tausonia | 0.33 ± 0.33 | 0.67 ± 0.33 | 86 ± 55.52 | 1.33 ± 0.88 | 1 ± 0.58 | 26.33 ± 2.96 | 86 ± 75 | 4 ± 2.52 | 6.67 ± 4.81 | 8.33 ± 4.84 |
| Teichospora | 0 ± 0 | 0 ± 0 | 0 ± 0 | 0 ± 0 | 0 ± 0 | 0 ± 0 | 0 ± 0 | 4.67 ± 4.67 | 0.67 ± 0.67 | 0 ± 0 |
| Tephrocybella | 0 ± 0 | 0 ± 0 | 1.67 ± 0.33 | 0 ± 0 | 0 ± 0 | 0.67 ± 0.67 | 0 ± 0 | 0 ± 0 | 0 ± 0 | 0 ± 0 |
| Terfezia | 6 ± 6 | 0 ± 0 | 0 ± 0 | 0 ± 0 | 0.33 ± 0.33 | 0.33 ± 0.33 | 0 ± 0 | 1 ± 1 | 0 ± 0 | 0 ± 0 |
| Tetracladium | 12 ± 2.52 | 3 ± 3 | 47.67 ± 2.96 | 7.67 ± 4.18 | 38.33 ± 7.06 | 148 ± 40.55 | 70 ± 54.03 | 721 ± 564.26 | 92 ± 88.02 | 8.33 ± 1.76 |
| Tetragoniomyces | 0 ± 0 | 0 ± 0 | 0 ± 0 | 0 ± 0 | 0 ± 0 | 0 ± 0 | 1 ± 1 | 0 ± 0 | 0 ± 0 | 0 ± 0 |
| Tetraploa | 0 ± 0 | 0 ± 0 | 0 ± 0 | 0 ± 0 | 0 ± 0 | 0 ± 0 | 0 ± 0 | 1 ± 1 | 0 ± 0 | 0 ± 0 |
| Tetraplosphaeria | 0 ± 0 | 0 ± 0 | 0 ± 0 | 0.33 ± 0.33 | 0 ± 0 | 0 ± 0 | 0.33 ± 0.33 | 2.33 ± 2.33 | 1 ± 1 | 0 ± 0 |
| Thelebolus | 0 ± 0 | 0 ± 0 | 16.67 ± 11.61 | 0 ± 0 | 1 ± 1 | 4 ± 4 | 1 ± 0.58 | 1 ± 1 | 0.33 ± 0.33 | 0 ± 0 |
| Thelephora | 0 ± 0 | 0 ± 0 | 0 ± 0 | 0 ± 0 | 0 ± 0 | 6.67 ± 6.17 | 0 ± 0 | 0 ± 0 | 0 ± 0 | 0 ± 0 |
| Thelonectria | 2 ± 1 | 1.67 ± 0.88 | 133.67 ± 18.98 | 5.33 ± 1.67 | 2 ± 0 | 59.67 ± 43.23 | 9 ± 5.51 | 18.67 ± 9.33 | 4 ± 3.51 | 6.67 ± 2.6 |
| Thermoascus | 0 ± 0 | 0 ± 0 | 0.33 ± 0.33 | 0 ± 0 | 0 ± 0 | 0 ± 0 | 0.67 ± 0.67 | 0 ± 0 | 0.33 ± 0.33 | 0.33 ± 0.33 |
| Thozetella | 0.33 ± 0.33 | 10.33 ± 6.74 | 0.67 ± 0.33 | 43.33 ± 37.35 | 0 ± 0 | 0.33 ± 0.33 | 21 ± 16.26 | 0.33 ± 0.33 | 0 ± 0 | 9 ± 8.5 |
| Thyronectria | 1.33 ± 0.88 | 3.33 ± 2.85 | 20.67 ± 6.36 | 6 ± 4.58 | 4.33 ± 1.45 | 38.33 ± 12.77 | 16.33 ± 9.21 | 0.33 ± 0.33 | 1.67 ± 0.88 | 4.67 ± 0.88 |
| Thysanorea | 0 ± 0 | 6 ± 4.51 | 0 ± 0 | 2 ± 2 | 0 ± 0 | 0 ± 0 | 5.67 ± 5.17 | 0.67 ± 0.33 | 5 ± 3.06 | 29.33 ± 23.05 |
| Tilachlidium | 0 ± 0 | 0 ± 0 | 0 ± 0 | 0.33 ± 0.33 | 0 ± 0 | 0 ± 0 | 1 ± 0.58 | 4 ± 4 | 0 ± 0 | 0 ± 0 |
| Tolypocladium | 0.67 ± 0.33 | 134.33 ± 33.55 | 21.67 ± 5.81 | 49 ± 19.67 | 2.33 ± 1.86 | 13.67 ± 9.21 | 79.33 ± 40.68 | 68.33 ± 55.63 | 45 ± 27.3 | 6.67 ± 2.19 |
| Tomentella | 0.33 ± 0.33 | 2.33 ± 1.33 | 27.67 ± 6.44 | 4.33 ± 2.6 | 3 ± 1.53 | 11 ± 1.53 | 12 ± 7.51 | 557.67 ± 545.68 | 512 ± 259.25 | 13.33 ± 4.48 |
| Torrubiella | 0 ± 0 | 0 ± 0 | 0 ± 0 | 0.67 ± 0.67 | 1 ± 1 | 0 ± 0 | 0 ± 0 | 0 ± 0 | 0 ± 0 | 0 ± 0 |
| Torula | 1.33 ± 0.33 | 0 ± 0 | 1.33 ± 0.88 | 0.33 ± 0.33 | 0 ± 0 | 4.67 ± 4.67 | 5.33 ± 4.84 | 11.67 ± 6.69 | 23.67 ± 23.67 | 3.67 ± 0.33 |
| Toxicocladosporium | 0 ± 0 | 0 ± 0 | 0 ± 0 | 0 ± 0 | 0 ± 0 | 0 ± 0 | 0 ± 0 | 0 ± 0 | 2.33 ± 2.33 | 0 ± 0 |
| Trechispora | 2 ± 1 | 29.67 ± 10.17 | 12.33 ± 5.24 | 174.67 ± 136.56 | 1.67 ± 0.88 | 8 ± 4.73 | 97.67 ± 59.61 | 33.67 ± 9.28 | 19.33 ± 2.19 | 2814 ± 2726.27 |
| Trematophoma | 0 ± 0 | 0 ± 0 | 0.33 ± 0.33 | 0 ± 0 | 1.33 ± 0.67 | 2.67 ± 2.19 | 0.33 ± 0.33 | 0 ± 0 | 0 ± 0 | 0 ± 0 |
| Trematosphaeria | 0 ± 0 | 0 ± 0 | 0 ± 0 | 0 ± 0 | 0 ± 0 | 0.67 ± 0.67 | 0 ± 0 | 0 ± 0 | 0 ± 0 | 0 ± 0 |
| Tremella | 22.67 ± 8.84 | 54.33 ± 46.94 | 22.33 ± 8.21 | 1.67 ± 0.88 | 3.33 ± 1.45 | 1 ± 0.58 | 11 ± 10.02 | 0 ± 0 | 0 ± 0 | 1.33 ± 0.88 |
| Tremellodendropsis | 0 ± 0 | 0 ± 0 | 0 ± 0 | 0 ± 0 | 0 ± 0 | 0 ± 0 | 0 ± 0 | 0 ± 0 | 0 ± 0 | 0.67 ± 0.67 |
| Triadelphia | 0 ± 0 | 0 ± 0 | 0 ± 0 | 0 ± 0 | 0 ± 0 | 16.33 ± 11.86 | 1.67 ± 1.67 | 0 ± 0 | 0 ± 0 | 0 ± 0 |
| Tricellula | 0.67 ± 0.33 | 1 ± 1 | 0.67 ± 0.67 | 0.67 ± 0.67 | 0.33 ± 0.33 | 2.33 ± 1.86 | 3.33 ± 2.85 | 10.33 ± 6.01 | 10.33 ± 9.35 | 1.67 ± 0.88 |
| Trichocladium | 5.33 ± 2.03 | 6 ± 2 | 1337.67 ± 1052.3 | 38 ± 9.45 | 29.33 ± 4.48 | 2581.33 ± 1188.62 | 776.33 ± 550.39 | 31 ± 13.53 | 8.67 ± 3.53 | 108.67 ± 15.5 |
| Trichoderma | 7.67 ± 1.45 | 366.67 ± 133.38 | 119 ± 42.15 | 62.33 ± 27.34 | 2 ± 1.15 | 64.33 ± 37.37 | 133.67 ± 66.27 | 71.33 ± 25.43 | 105 ± 65 | 78.33 ± 7.45 |
| Trichoglossum | 11.33 ± 8.41 | 0 ± 0 | 8 ± 5.69 | 0 ± 0 | 0.67 ± 0.33 | 0 ± 0 | 0 ± 0 | 0 ± 0 | 0 ± 0 | 2 ± 2 |
| Tricholoma | 0 ± 0 | 0 ± 0 | 0 ± 0 | 0.33 ± 0.33 | 0 ± 0 | 0 ± 0 | 2 ± 1.15 | 0 ± 0 | 0 ± 0 | 1.33 ± 1.33 |
| Trichomerium | 1.33 ± 1.33 | 0 ± 0 | 4.67 ± 1.2 | 0.33 ± 0.33 | 0 ± 0 | 8.67 ± 2.73 | 14.67 ± 8.21 | 63 ± 52.32 | 65 ± 63.51 | 23.33 ± 18.48 |
| Trichophaea | 0 ± 0 | 0 ± 0 | 0 ± 0 | 0 ± 0 | 0 ± 0 | 0 ± 0 | 0 ± 0 | 0 ± 0 | 0 ± 0 | 0 ± 0 |
| Trichosporiella | 0.33 ± 0.33 | 0 ± 0 | 5.33 ± 1.76 | 0 ± 0 | 0.67 ± 0.33 | 1 ± 0.58 | 0 ± 0 | 18.33 ± 18.33 | 0.33 ± 0.33 | 0.33 ± 0.33 |
| Trichosporon | 16.33 ± 7.54 | 0 ± 0 | 7.33 ± 3.33 | 83.67 ± 14.31 | 605 ± 261.1 | 19.33 ± 11.57 | 103 ± 51.64 | 5.33 ± 4.84 | 2.67 ± 2.67 | 1 ± 1 |
| Trichothecium | 0 ± 0 | 0 ± 0 | 0 ± 0 | 0.67 ± 0.67 | 0.33 ± 0.33 | 54 ± 31.94 | 18.67 ± 18.67 | 0.67 ± 0.67 | 6.67 ± 4.41 | 0.67 ± 0.67 |
| Trichurus | 0 ± 0 | 0 ± 0 | 0 ± 0 | 0 ± 0 | 0 ± 0 | 0.67 ± 0.67 | 1.67 ± 0.67 | 0.33 ± 0.33 | 0 ± 0 | 1.33 ± 1.33 |
| Tricladium | 1.67 ± 1.67 | 0 ± 0 | 11.67 ± 3.71 | 0 ± 0 | 0 ± 0 | 4.67 ± 3.71 | 2 ± 1.53 | 0 ± 0 | 0 ± 0 | 0 ± 0 |
| Trimorphomyces | 0 ± 0 | 0 ± 0 | 0 ± 0 | 0 ± 0 | 0 ± 0 | 0 ± 0 | 0 ± 0 | 0 ± 0 | 0 ± 0 | 0 ± 0 |
| Truncatella | 0 ± 0 | 0 ± 0 | 0.33 ± 0.33 | 0 ± 0 | 0 ± 0 | 0 ± 0 | 0 ± 0 | 0 ± 0 | 0 ± 0 | 0 ± 0 |
| Tubaria | 0 ± 0 | 0 ± 0 | 0 ± 0 | 0 ± 0 | 0 ± 0 | 11 ± 5.57 | 0.67 ± 0.67 | 0.67 ± 0.67 | 0 ± 0 | 0 ± 0 |
| Tumularia | 0 ± 0 | 0 ± 0 | 0 ± 0 | 0 ± 0 | 0 ± 0 | 0.67 ± 0.33 | 0 ± 0 | 0 ± 0 | 0 ± 0 | 0 ± 0 |
| Tygervalleyomyces | 1.67 ± 0.67 | 0.67 ± 0.67 | 13.67 ± 6.44 | 13.67 ± 6.69 | 1 ± 1 | 0.33 ± 0.33 | 2.33 ± 1.86 | 0 ± 0 | 0.33 ± 0.33 | 3.67 ± 3.67 |
| Tympanis | 0 ± 0 | 0 ± 0 | 0 ± 0 | 0 ± 0 | 1.67 ± 1.2 | 0 ± 0 | 0 ± 0 | 0 ± 0 | 0 ± 0 | 0 ± 0 |
| Udeniozyma | 6.67 ± 5.67 | 7.67 ± 3.53 | 1.67 ± 1.67 | 10.67 ± 3.33 | 1.33 ± 1.33 | 0.67 ± 0.67 | 5 ± 2.65 | 29 ± 27.02 | 4 ± 2.08 | 0.33 ± 0.33 |
| Ugola | 0.33 ± 0.33 | 2 ± 1.53 | 0 ± 0 | 0 ± 0 | 0.33 ± 0.33 | 0 ± 0 | 0 ± 0 | 0 ± 0 | 0.67 ± 0.33 | 2 ± 0.58 |
| Umbilicaria | 2 ± 1 | 0 ± 0 | 1.33 ± 0.88 | 0.67 ± 0.67 | 0.67 ± 0.67 | 0.33 ± 0.33 | 0.33 ± 0.33 | 0 ± 0 | 2.67 ± 2.67 | 12.33 ± 5.24 |
| Uncispora | 0 ± 0 | 0 ± 0 | 0.67 ± 0.67 | 0 ± 0 | 0 ± 0 | 0.33 ± 0.33 | 0.67 ± 0.67 | 0.33 ± 0.33 | 2.33 ± 2.33 | 0 ± 0 |
| Unclassified | 619.67 ± 282.27 | 1611.33 ± 506.5 | 971.33 ± 162.73 | 19219 ± 1922.36 | 1293.67 ± 101.41 | 1133.33 ± 159.31 | 7377.67 ± 2745.31 | 2392 ± 1032.17 | 2165 ± 1800.38 | 437.33 ± 83.59 |
| Ustilaginoidea | 3.67 ± 2.19 | 3.33 ± 2.85 | 1.33 ± 0.88 | 1 ± 0.58 | 1 ± 0.58 | 8 ± 3.61 | 11.67 ± 9.74 | 9.33 ± 2.73 | 0.67 ± 0.67 | 1.67 ± 1.2 |
| Valsonectria | 0 ± 0 | 0 ± 0 | 0 ± 0 | 0 ± 0 | 0 ± 0 | 0.67 ± 0.67 | 0 ± 0 | 0 ± 0 | 0 ± 0 | 0 ± 0 |
| Vanderbylia | 0 ± 0 | 0 ± 0 | 0 ± 0 | 0 ± 0 | 0 ± 0 | 6.67 ± 3.67 | 0 ± 0 | 0 ± 0 | 0 ± 0 | 0 ± 0 |
| Vanrija | 0 ± 0 | 0 ± 0 | 0.33 ± 0.33 | 0 ± 0 | 0 ± 0 | 1 ± 1 | 0 ± 0 | 0 ± 0 | 0 ± 0 | 0 ± 0 |
| Vararia | 4 ± 1 | 0 ± 0 | 0 ± 0 | 0 ± 0 | 0.33 ± 0.33 | 0 ± 0 | 0 ± 0 | 0 ± 0 | 0 ± 0 | 0 ± 0 |
| Varicosporellopsis | 0 ± 0 | 0 ± 0 | 0.67 ± 0.67 | 0 ± 0 | 0 ± 0 | 8.33 ± 4.06 | 2.33 ± 2.33 | 4 ± 4 | 0 ± 0 | 0.33 ± 0.33 |
| Velutarina | 0 ± 0 | 0 ± 0 | 0 ± 0 | 0 ± 0 | 0 ± 0 | 0 ± 0 | 0 ± 0 | 1.67 ± 1.67 | 3.67 ± 3.67 | 0 ± 0 |
| Vermiconia | 0 ± 0 | 0 ± 0 | 0 ± 0 | 0 ± 0 | 0 ± 0 | 0 ± 0 | 1.33 ± 1.33 | 0 ± 0 | 0 ± 0 | 0 ± 0 |
| Vermiculariopsiella | 0.33 ± 0.33 | 24.33 ± 18.34 | 0 ± 0 | 1 ± 1 | 0 ± 0 | 3 ± 1.53 | 2 ± 1.53 | 0 ± 0 | 0 ± 0 | 0 ± 0 |
| Vermispora | 14.67 ± 9.21 | 8.33 ± 4.26 | 5 ± 2.89 | 4.33 ± 4.33 | 4.67 ± 3.67 | 11 ± 5.57 | 19 ± 11.37 | 106.67 ± 45.08 | 67.67 ± 50.23 | 30.33 ± 13.69 |
| Veronaea | 4 ± 2.08 | 0.33 ± 0.33 | 28.67 ± 15.71 | 1 ± 1 | 1.33 ± 0.88 | 5.33 ± 2.91 | 6.33 ± 3.84 | 7.33 ± 3.33 | 0.67 ± 0.33 | 1.33 ± 0.33 |
| Veronaeopsis | 0 ± 0 | 2.33 ± 2.33 | 0 ± 0 | 0 ± 0 | 0 ± 0 | 0 ± 0 | 0 ± 0 | 40 ± 38.02 | 3.67 ± 3.67 | 0.33 ± 0.33 |
| Verrucaria | 0.67 ± 0.67 | 0.33 ± 0.33 | 2.33 ± 1.86 | 41.67 ± 22.91 | 5.67 ± 2.4 | 3 ± 2.52 | 18 ± 8.39 | 1.33 ± 0.88 | 13 ± 7 | 3 ± 2.52 |
| Verticillium | 0 ± 0 | 0 ± 0 | 1 ± 0.58 | 0 ± 0 | 2 ± 1.15 | 3.67 ± 2.19 | 1.33 ± 0.88 | 19.33 ± 18.84 | 2 ± 2 | 0 ± 0 |
| Vestigium | 0 ± 0 | 0 ± 0 | 0 ± 0 | 0 ± 0 | 0 ± 0 | 0 ± 0 | 0 ± 0 | 0 ± 0 | 0 ± 0 | 0.67 ± 0.67 |
| Vishniacozyma | 6.67 ± 5.17 | 1.67 ± 1.67 | 10.67 ± 2.03 | 13 ± 2.52 | 33 ± 9.07 | 26.67 ± 4.84 | 44.67 ± 27.67 | 18 ± 11.53 | 25.33 ± 23.84 | 305.33 ± 127.96 |
| Volutella | 4.67 ± 2.67 | 5.67 ± 3.48 | 46.33 ± 11.72 | 11.33 ± 4.7 | 23.67 ± 11.61 | 447.33 ± 196.41 | 190.67 ± 166.67 | 402.33 ± 199.54 | 177.33 ± 154.09 | 31.33 ± 12.24 |
| Volvariella | 0 ± 0 | 0 ± 0 | 0 ± 0 | 0.33 ± 0.33 | 0 ± 0 | 0.33 ± 0.33 | 0 ± 0 | 0 ± 0 | 0 ± 0 | 0 ± 0 |
| Vrystaatia | 0 ± 0 | 0.67 ± 0.33 | 0 ± 0 | 0 ± 0 | 0 ± 0 | 0 ± 0 | 0 ± 0 | 0 ± 0 | 0 ± 0 | 0 ± 0 |
| Waitea | 0 ± 0 | 0 ± 0 | 0 ± 0 | 0 ± 0 | 0 ± 0 | 0 ± 0 | 0 ± 0 | 0 ± 0 | 0 ± 0 | 1.67 ± 0.88 |
| Wardomyces | 0 ± 0 | 0 ± 0 | 21 ± 20.5 | 0 ± 0 | 0 ± 0 | 1.67 ± 1.67 | 0.33 ± 0.33 | 0 ± 0 | 0 ± 0 | 0 ± 0 |
| Wardomycopsis | 0 ± 0 | 0 ± 0 | 0 ± 0 | 0 ± 0 | 0 ± 0 | 0 ± 0 | 2.33 ± 2.33 | 0 ± 0 | 0 ± 0 | 0 ± 0 |
| Westerdykella | 0 ± 0 | 0 ± 0 | 0 ± 0 | 0 ± 0 | 0.67 ± 0.67 | 0 ± 0 | 0 ± 0 | 0 ± 0 | 0 ± 0 | 0 ± 0 |
| Whalleya | 0 ± 0 | 0 ± 0 | 1 ± 1 | 0 ± 0 | 0 ± 0 | 0 ± 0 | 0 ± 0 | 0.33 ± 0.33 | 0 ± 0 | 0 ± 0 |
| Wojnowiciella | 0 ± 0 | 0 ± 0 | 0 ± 0 | 0 ± 0 | 0 ± 0 | 0 ± 0 | 0 ± 0 | 0 ± 0 | 1 ± 0.58 | 0 ± 0 |
| Wongia | 0 ± 0 | 0 ± 0 | 0 ± 0 | 0 ± 0 | 0 ± 0 | 0 ± 0 | 0 ± 0 | 1.33 ± 1.33 | 0 ± 0 | 0 ± 0 |
| Xenoacremonium | 2.33 ± 2.33 | 164.33 ± 104.14 | 0 ± 0 | 12 ± 9.5 | 0 ± 0 | 2.67 ± 0.88 | 55.67 ± 37.88 | 34.67 ± 23.31 | 10 ± 10 | 0.67 ± 0.33 |
| Xenochalara | 0 ± 0 | 0 ± 0 | 0 ± 0 | 0 ± 0 | 0 ± 0 | 0 ± 0 | 0 ± 0 | 0.67 ± 0.33 | 0.33 ± 0.33 | 0 ± 0 |
| Xenophacidiella | 0 ± 0 | 0 ± 0 | 0 ± 0 | 0 ± 0 | 0 ± 0 | 6.33 ± 6.33 | 0 ± 0 | 0 ± 0 | 0 ± 0 | 0.33 ± 0.33 |
| Xenoramularia | 0 ± 0 | 0 ± 0 | 0 ± 0 | 0 ± 0 | 0 ± 0 | 0.33 ± 0.33 | 4.67 ± 4.67 | 0 ± 0 | 0 ± 0 | 0 ± 0 |
| Xenoteratosphaeria | 0 ± 0 | 0 ± 0 | 0 ± 0 | 0.33 ± 0.33 | 0 ± 0 | 0 ± 0 | 0 ± 0 | 0 ± 0 | 0 ± 0 | 6.33 ± 6.33 |
| Xepicula | 0 ± 0 | 0 ± 0 | 0 ± 0 | 0 ± 0 | 0 ± 0 | 0 ± 0 | 0 ± 0 | 0 ± 0 | 0 ± 0 | 0.67 ± 0.67 |
| Xylaria | 1 ± 0 | 0 ± 0 | 4 ± 1 | 1.67 ± 1.2 | 8 ± 2 | 4.67 ± 3.28 | 4.33 ± 3.38 | 4 ± 2.31 | 2 ± 2 | 3.33 ± 1.76 |
| Xylohypha | 0.33 ± 0.33 | 5.67 ± 5.67 | 13 ± 5.51 | 2 ± 1 | 0 ± 0 | 2.33 ± 2.33 | 3.67 ± 2.03 | 0.33 ± 0.33 | 0.33 ± 0.33 | 0.67 ± 0.33 |
| Zasmidium | 0 ± 0 | 0 ± 0 | 7 ± 5.13 | 0 ± 0 | 0 ± 0 | 5.33 ± 1.45 | 0 ± 0 | 0 ± 0 | 0 ± 0 | 0 ± 0 |
| Zopfiella | 10.67 ± 8.17 | 0 ± 0 | 104.67 ± 30.53 | 7.33 ± 3.18 | 10.33 ± 4.91 | 67.33 ± 22.75 | 21.67 ± 1.45 | 25.33 ± 23.36 | 6.67 ± 6.67 | 16.67 ± 12.02 |
| Zygosporium | 0 ± 0 | 0 ± 0 | 0 ± 0 | 0 ± 0 | 0.67 ± 0.33 | 0 ± 0 | 0 ± 0 | 6 ± 2.65 | 2 ± 2 | 12 ± 10.54 |
| Zymoseptoria | 0 ± 0 | 0 ± 0 | 0 ± 0 | 0 ± 0 | 0 ± 0 | 0 ± 0 | 1 ± 1 | 0 ± 0 | 0 ± 0 | 1 ± 0.58 |

**Table S14. Abundance of species.**

| Species | FS1 | FS2 | FT1 | FT2 | NA1 | NA2 | NB1 | NB2 | M1 | M2 |
| --- | --- | --- | --- | --- | --- | --- | --- | --- | --- | --- |
| Aaosphaeria arxii | 1.67 ± 1.2 | 5.33 ± 5.33 | 25.33 ± 5.84 | 0.67 ± 0.67 | 0.33 ± 0.33 | 13.33 ± 8.95 | 2 ± 1.53 | 1.33 ± 0.88 | 1.67 ± 0.88 | 5.67 ± 0.88 |
| Abrothallus suecicus | 0 ± 0 | 0 ± 0 | 0 ± 0 | 0 ± 0 | 0 ± 0 | 0 ± 0 | 1.67 ± 1.67 | 0 ± 0 | 0 ± 0 | 0 ± 0 |
| Abrothallus usneae | 0 ± 0 | 0 ± 0 | 13.33 ± 13.33 | 0 ± 0 | 0.67 ± 0.67 | 1 ± 0.58 | 1 ± 1 | 0 ± 0 | 0 ± 0 | 0 ± 0 |
| Acanthostigma perpusillum | 0 ± 0 | 0 ± 0 | 0 ± 0 | 0 ± 0 | 0 ± 0 | 0 ± 0 | 0 ± 0 | 0 ± 0 | 0 ± 0 | 0 ± 0 |
| Acaulium albonigrescens | 0 ± 0 | 0 ± 0 | 0 ± 0 | 0 ± 0 | 0 ± 0 | 0 ± 0 | 0 ± 0 | 0 ± 0 | 0 ± 0 | 0 ± 0 |
| Acaulium sp. 1 RJ2014 | 0 ± 0 | 2.67 ± 2.67 | 0.67 ± 0.67 | 1 ± 0.58 | 0 ± 0 | 38 ± 23.81 | 1.67 ± 0.88 | 0 ± 0 | 0 ± 0 | 0 ± 0 |
| Acaulopage dichotoma | 0 ± 0 | 0 ± 0 | 0 ± 0 | 0 ± 0 | 0 ± 0 | 0 ± 0 | 0 ± 0 | 0 ± 0 | 0 ± 0 | 0 ± 0 |
| Acaulopage tetraceros | 0 ± 0 | 0 ± 0 | 0 ± 0 | 0 ± 0 | 0.33 ± 0.33 | 0.67 ± 0.67 | 0 ± 0 | 0.33 ± 0.33 | 0 ± 0 | 0 ± 0 |
| Acephala applanata | 0 ± 0 | 0 ± 0 | 0 ± 0 | 0 ± 0 | 0 ± 0 | 0 ± 0 | 0 ± 0 | 2.67 ± 1.45 | 0 ± 0 | 0 ± 0 |
| Achroceratosphaeria potamia | 0 ± 0 | 0 ± 0 | 0.67 ± 0.67 | 0 ± 0 | 0 ± 0 | 4 ± 2.31 | 4 ± 4 | 4.67 ± 4.67 | 1.67 ± 1.2 | 0 ± 0 |
| Achroiostachys humicola | 0.33 ± 0.33 | 7 ± 7 | 3.33 ± 1.76 | 0 ± 0 | 4 ± 0.58 | 3 ± 2.52 | 0.67 ± 0.67 | 0 ± 0 | 0 ± 0 | 0 ± 0 |
| Acremonium antarcticum | 1 ± 0.58 | 13.67 ± 13.67 | 2 ± 0.58 | 8.67 ± 3.93 | 11.67 ± 5.36 | 149 ± 84.32 | 24.33 ± 4.63 | 0 ± 0 | 0.67 ± 0.67 | 52.67 ± 30.17 |
| Acremonium asperulatum | 0 ± 0 | 0 ± 0 | 0 ± 0 | 0 ± 0 | 0 ± 0 | 0 ± 0 | 0 ± 0 | 2.67 ± 2.67 | 0 ± 0 | 0.33 ± 0.33 |
| Acremonium camptosporum | 2 ± 1.15 | 0 ± 0 | 0 ± 0 | 0 ± 0 | 0.67 ± 0.67 | 0 ± 0 | 0 ± 0 | 36.33 ± 20.51 | 53.33 ± 53.33 | 3.33 ± 2.33 |
| Acremonium charticola | 0 ± 0 | 0 ± 0 | 0 ± 0 | 0.33 ± 0.33 | 1 ± 1 | 0 ± 0 | 0.33 ± 0.33 | 0 ± 0 | 19.67 ± 19.67 | 0.33 ± 0.33 |
| Acremonium cucurbitacearum | 0 ± 0 | 0 ± 0 | 0 ± 0 | 0 ± 0 | 0 ± 0 | 0 ± 0 | 0 ± 0 | 0 ± 0 | 0.33 ± 0.33 | 5 ± 2.08 |
| Acremonium distortum | 0 ± 0 | 0 ± 0 | 1.67 ± 1.67 | 0 ± 0 | 0 ± 0 | 2 ± 2 | 0 ± 0 | 0 ± 0 | 0 ± 0 | 0 ± 0 |
| Acremonium egyptiacum | 0 ± 0 | 0 ± 0 | 0 ± 0 | 0 ± 0 | 2.33 ± 0.33 | 0 ± 0 | 3.67 ± 2.33 | 0 ± 0 | 0 ± 0 | 2.67 ± 1.76 |
| Acremonium furcatum | 0.67 ± 0.67 | 53 ± 17.21 | 14.33 ± 3.84 | 11 ± 1.15 | 13.33 ± 4.33 | 233 ± 104.31 | 114.33 ± 77.88 | 4.33 ± 0.88 | 3.33 ± 1.2 | 19.33 ± 9.84 |
| Acremonium fusidioides | 0 ± 0 | 0 ± 0 | 0 ± 0 | 0 ± 0 | 1 ± 0.58 | 0 ± 0 | 0 ± 0 | 1.33 ± 0.88 | 0 ± 0 | 0 ± 0 |
| Acremonium luzulae | 0 ± 0 | 0 ± 0 | 0 ± 0 | 0 ± 0 | 4.33 ± 2.6 | 0.33 ± 0.33 | 0 ± 0 | 0 ± 0 | 0 ± 0 | 0 ± 0 |
| Acremonium persicinum | 0 ± 0 | 0 ± 0 | 0 ± 0 | 0 ± 0 | 0.33 ± 0.33 | 1 ± 1 | 1.33 ± 0.67 | 1 ± 1 | 1.33 ± 0.67 | 0 ± 0 |
| Acremonium psammosporum | 0 ± 0 | 0.33 ± 0.33 | 0 ± 0 | 2 ± 1 | 0 ± 0 | 0 ± 0 | 3.67 ± 0.88 | 0 ± 0 | 0.33 ± 0.33 | 6.33 ± 2.33 |
| Acremonium pteridii | 0.33 ± 0.33 | 0 ± 0 | 8.33 ± 6.84 | 0.67 ± 0.67 | 0.33 ± 0.33 | 17.67 ± 8.45 | 5.67 ± 5.17 | 1.67 ± 1.2 | 3.33 ± 1.76 | 7 ± 5.13 |
| Acremonium roseolum | 0 ± 0 | 6.33 ± 2.91 | 0 ± 0 | 0.67 ± 0.67 | 0 ± 0 | 0 ± 0 | 0 ± 0 | 0 ± 0 | 0 ± 0 | 0 ± 0 |
| Acremonium rutilum | 0 ± 0 | 0 ± 0 | 1.33 ± 1.33 | 0 ± 0 | 2 ± 2 | 0 ± 0 | 0 ± 0 | 0 ± 0 | 0 ± 0 | 0 ± 0 |
| Acremonium sordidulum | 0 ± 0 | 0 ± 0 | 0 ± 0 | 0.67 ± 0.67 | 0 ± 0 | 0 ± 0 | 0 ± 0 | 0 ± 0 | 0 ± 0 | 0 ± 0 |
| Acremonium sp. (in: Ascomycota) | 6 ± 3.06 | 0 ± 0 | 1 ± 1 | 0 ± 0 | 1.33 ± 0.88 | 0.33 ± 0.33 | 0 ± 0 | 1.67 ± 0.88 | 0 ± 0 | 2 ± 1.15 |
| Acremonium sp. 0609ALT47Q6-KH | 0 ± 0 | 0 ± 0 | 0 ± 0 | 0.67 ± 0.67 | 0 ± 0 | 6.33 ± 4.91 | 4.67 ± 4.67 | 0.67 ± 0.67 | 0 ± 0 | 2 ± 1.53 |
| Acremonium sp. BRO-2013 | 0.33 ± 0.33 | 0 ± 0 | 0 ± 0 | 0 ± 0 | 0 ± 0 | 0 ± 0 | 0 ± 0 | 0 ± 0 | 0.67 ± 0.67 | 0 ± 0 |
| Acremonium sp. DWS6m3 | 0.33 ± 0.33 | 0 ± 0 | 0 ± 0 | 0 ± 0 | 0.67 ± 0.67 | 0 ± 0 | 0 ± 0 | 9 ± 4.93 | 7.67 ± 7.67 | 0 ± 0 |
| Acremonium sp. F9A015 | 0 ± 0 | 0 ± 0 | 0 ± 0 | 0 ± 0 | 0 ± 0 | 1.67 ± 0.88 | 0 ± 0 | 0 ± 0 | 0 ± 0 | 1.67 ± 1.67 |
| Acremonium sp. HF12701 | 0 ± 0 | 0 ± 0 | 0 ± 0 | 0 ± 0 | 0.67 ± 0.33 | 0 ± 0 | 0 ± 0 | 0 ± 0 | 0 ± 0 | 0 ± 0 |
| Acremonium sp. KM1p | 0 ± 0 | 0.33 ± 0.33 | 0.67 ± 0.33 | 2.33 ± 1.86 | 1 ± 0.58 | 26.33 ± 14.88 | 26.67 ± 21.67 | 3.67 ± 3.67 | 6.33 ± 4.48 | 1.67 ± 0.88 |
| Acremonium sp. MJ35 | 0 ± 0 | 0 ± 0 | 0 ± 0 | 0 ± 0 | 0 ± 0 | 0 ± 0 | 0.67 ± 0.67 | 0 ± 0 | 0 ± 0 | 0 ± 0 |
| Acremonium sp. TR080 | 0 ± 0 | 0 ± 0 | 2 ± 2 | 0 ± 0 | 0 ± 0 | 0 ± 0 | 0 ± 0 | 0 ± 0 | 0 ± 0 | 1.33 ± 1.33 |
| Acremonium sp. r293 | 0 ± 0 | 0 ± 0 | 0 ± 0 | 0 ± 0 | 0 ± 0 | 0.67 ± 0.67 | 0.33 ± 0.33 | 0.67 ± 0.33 | 0.67 ± 0.67 | 4.67 ± 4.67 |
| Acremonium variecolor | 0 ± 0 | 0 ± 0 | 0 ± 0 | 0 ± 0 | 0 ± 0 | 0 ± 0 | 0 ± 0 | 0 ± 0 | 0 ± 0 | 0 ± 0 |
| Acremonium vitellinum | 0 ± 0 | 0 ± 0 | 0 ± 0 | 0 ± 0 | 0 ± 0 | 0 ± 0 | 3 ± 3 | 0 ± 0 | 0 ± 0 | 0 ± 0 |
| Acrocalymma sp. | 1.33 ± 1.33 | 2 ± 1.53 | 3 ± 2.08 | 2 ± 2 | 0.33 ± 0.33 | 6.67 ± 1.33 | 2.67 ± 2.19 | 13.33 ± 6.39 | 10.67 ± 9.68 | 5.67 ± 1.2 |
| Acrocalymma vagum | 1.33 ± 0.67 | 0 ± 0 | 0 ± 0 | 0 ± 0 | 3 ± 2.52 | 0 ± 0 | 0 ± 0 | 0.33 ± 0.33 | 0.67 ± 0.67 | 1 ± 0.58 |
| Acrocalymma walkeri | 0 ± 0 | 0 ± 0 | 0 ± 0 | 0 ± 0 | 5.67 ± 1.86 | 0 ± 0 | 0 ± 0 | 0 ± 0 | 0 ± 0 | 0 ± 0 |
| Acrodictys sp. | 0 ± 0 | 0 ± 0 | 0 ± 0 | 0 ± 0 | 0 ± 0 | 0 ± 0 | 0.67 ± 0.67 | 0 ± 0 | 0 ± 0 | 0 ± 0 |
| Acrodontium antarcticum | 0 ± 0 | 0 ± 0 | 0 ± 0 | 0 ± 0 | 0 ± 0 | 0.33 ± 0.33 | 0 ± 0 | 0 ± 0 | 0 ± 0 | 0 ± 0 |
| Acrodontium hydnicola | 0 ± 0 | 0 ± 0 | 0 ± 0 | 0 ± 0 | 0 ± 0 | 0.67 ± 0.67 | 0 ± 0 | 0 ± 0 | 0 ± 0 | 0 ± 0 |
| Acrogenospora carmichaeliana | 0 ± 0 | 0 ± 0 | 0 ± 0 | 0 ± 0 | 0 ± 0 | 0 ± 0 | 7.67 ± 7.67 | 0 ± 0 | 0.33 ± 0.33 | 0.33 ± 0.33 |
| Acrophialophora levis | 0 ± 0 | 0.67 ± 0.67 | 1 ± 1 | 0 ± 0 | 0 ± 0 | 0.33 ± 0.33 | 0 ± 0 | 0 ± 0 | 0 ± 0 | 0 ± 0 |
| Acrophialophora nainiana | 0 ± 0 | 0 ± 0 | 0 ± 0 | 0 ± 0 | 0.67 ± 0.67 | 0 ± 0 | 1 ± 0.58 | 0 ± 0 | 0 ± 0 | 0 ± 0 |
| Aculeata aquatica | 0 ± 0 | 0 ± 0 | 0 ± 0 | 0.33 ± 0.33 | 0 ± 0 | 0 ± 0 | 0.67 ± 0.67 | 0 ± 0 | 0 ± 0 | 0 ± 0 |
| Agonimia allobata | 0 ± 0 | 0.33 ± 0.33 | 8 ± 7.51 | 1.33 ± 0.88 | 0 ± 0 | 8 ± 8 | 17.67 ± 14.19 | 2998.67 ± 2972.17 | 1357.33 ± 1242.24 | 8.67 ± 4.37 |
| Agrocybe praecox | 0 ± 0 | 0 ± 0 | 0 ± 0 | 0 ± 0 | 0 ± 0 | 0.33 ± 0.33 | 0 ± 0 | 0 ± 0 | 0 ± 0 | 0 ± 0 |
| Alatospora flagellata | 0.67 ± 0.67 | 0 ± 0 | 7.33 ± 3.71 | 0.67 ± 0.67 | 0 ± 0 | 2.67 ± 2.67 | 0.67 ± 0.33 | 13 ± 11.53 | 5.33 ± 4.84 | 0 ± 0 |
| Alatospora pulchella | 0 ± 0 | 0 ± 0 | 21 ± 8.66 | 0 ± 0 | 3.67 ± 0.67 | 1.67 ± 0.88 | 0 ± 0 | 0 ± 0 | 0 ± 0 | 0 ± 0 |
| Albifimbria verrucaria | 0 ± 0 | 1.67 ± 1.67 | 39.67 ± 10.99 | 0 ± 0 | 0.33 ± 0.33 | 7 ± 3.06 | 2 ± 1.53 | 5.67 ± 3.67 | 0.33 ± 0.33 | 27 ± 8.08 |
| Aleurodiscus sp. MG266 | 0 ± 0 | 0 ± 0 | 3.67 ± 3.67 | 0.33 ± 0.33 | 0 ± 0 | 0 ± 0 | 0 ± 0 | 0 ± 0 | 0 ± 0 | 0 ± 0 |
| Alfaria terrestris | 0.33 ± 0.33 | 11.33 ± 5.55 | 0.33 ± 0.33 | 1 ± 0.58 | 0 ± 0 | 0 ± 0 | 4 ± 2.65 | 0 ± 0 | 0 ± 0 | 0 ± 0 |
| Alfaria vitis | 0 ± 0 | 3 ± 3 | 0 ± 0 | 0.33 ± 0.33 | 0.33 ± 0.33 | 0.33 ± 0.33 | 0 ± 0 | 0 ± 0 | 0.33 ± 0.33 | 0.67 ± 0.67 |
| Alloclavaria purpurea | 0 ± 0 | 0 ± 0 | 6.33 ± 2.19 | 0 ± 0 | 0 ± 0 | 0 ± 0 | 0.33 ± 0.33 | 0 ± 0 | 0 ± 0 | 0 ± 0 |
| Alnicola sp. | 0 ± 0 | 0 ± 0 | 0 ± 0 | 0 ± 0 | 0 ± 0 | 1 ± 1 | 0 ± 0 | 0 ± 0 | 0 ± 0 | 0 ± 0 |
| Alnicola umbrina | 0 ± 0 | 0 ± 0 | 0 ± 0 | 0 ± 0 | 0 ± 0 | 0 ± 0 | 0 ± 0 | 0 ± 0 | 0 ± 0 | 3 ± 3 |
| Alternaria chlamydospora | 0 ± 0 | 0 ± 0 | 0.33 ± 0.33 | 0 ± 0 | 0 ± 0 | 1.67 ± 1.67 | 0 ± 0 | 0 ± 0 | 0 ± 0 | 0 ± 0 |
| Alternaria longissima | 0 ± 0 | 0 ± 0 | 0 ± 0 | 0 ± 0 | 0 ± 0 | 0 ± 0 | 0 ± 0 | 0 ± 0 | 0.33 ± 0.33 | 0 ± 0 |
| Alternaria sp. | 0.67 ± 0.67 | 0.33 ± 0.33 | 6.33 ± 2.85 | 0.67 ± 0.67 | 1.33 ± 0.33 | 9.67 ± 5.78 | 6.33 ± 2.03 | 16.67 ± 8.41 | 3.33 ± 2.03 | 4 ± 2.08 |
| Alternaria sp. R60.1 | 0 ± 0 | 0 ± 0 | 0 ± 0 | 0 ± 0 | 0 ± 0 | 1.67 ± 1.67 | 0.33 ± 0.33 | 89.33 ± 48.79 | 73 ± 71.5 | 2 ± 1.53 |
| Amanita ceciliae | 0 ± 0 | 0 ± 0 | 0 ± 0 | 0 ± 0 | 0 ± 0 | 0 ± 0 | 0.67 ± 0.67 | 2.33 ± 2.33 | 10.33 ± 6.74 | 0 ± 0 |
| Amauroascus volatilis-patellis | 0 ± 0 | 0 ± 0 | 0 ± 0 | 0 ± 0 | 0 ± 0 | 0 ± 0 | 0.33 ± 0.33 | 0 ± 0 | 0 ± 0 | 0 ± 0 |
| Ambispora leptoticha | 0 ± 0 | 1 ± 1 | 0 ± 0 | 0 ± 0 | 0 ± 0 | 0 ± 0 | 0 ± 0 | 0 ± 0 | 0 ± 0 | 0 ± 0 |
| Ambispora sp.1 SL-2017 | 0 ± 0 | 0 ± 0 | 0 ± 0 | 0 ± 0 | 0 ± 0 | 0 ± 0 | 0 ± 0 | 0 ± 0 | 0 ± 0 | 0 ± 0 |
| Amesia gelasinospora | 0 ± 0 | 0 ± 0 | 0 ± 0 | 0 ± 0 | 0 ± 0 | 0 ± 0 | 0.67 ± 0.67 | 0 ± 0 | 0 ± 0 | 0 ± 0 |
| Amesia nigricolor | 1 ± 0.58 | 0 ± 0 | 16.33 ± 6.23 | 1.33 ± 0.88 | 0.67 ± 0.67 | 25.67 ± 6.39 | 33 ± 29.09 | 76.67 ± 73.18 | 12 ± 5.57 | 5 ± 1.15 |
| Amoeboradix gromovi | 0 ± 0 | 0 ± 0 | 0 ± 0 | 0 ± 0 | 0 ± 0 | 0 ± 0 | 0 ± 0 | 0 ± 0 | 0.67 ± 0.67 | 0 ± 0 |
| Ampelomyces sp. | 0 ± 0 | 0 ± 0 | 5.67 ± 5.17 | 0.33 ± 0.33 | 0 ± 0 | 0 ± 0 | 0.33 ± 0.33 | 0 ± 0 | 0 ± 0 | 0 ± 0 |
| Amphinema sp. (in: Fungi) | 0 ± 0 | 0 ± 0 | 1 ± 1 | 0 ± 0 | 0 ± 0 | 0.33 ± 0.33 | 0 ± 0 | 0 ± 0 | 0 ± 0 | 0 ± 0 |
| Amphirosellinia nigrospora | 0 ± 0 | 0 ± 0 | 0.67 ± 0.67 | 0 ± 0 | 0 ± 0 | 0 ± 0 | 0 ± 0 | 0 ± 0 | 0 ± 0 | 0 ± 0 |
| Amylocorticiellum sp. UC2022882 | 0 ± 0 | 0 ± 0 | 0 ± 0 | 0 ± 0 | 0 ± 0 | 0 ± 0 | 1.33 ± 1.33 | 0 ± 0 | 0 ± 0 | 0 ± 0 |
| Amylocorticium cebennense | 0 ± 0 | 0 ± 0 | 0 ± 0 | 1 ± 1 | 0 ± 0 | 0 ± 0 | 0 ± 0 | 0.33 ± 0.33 | 0 ± 0 | 0 ± 0 |
| Annulohypoxylon multiforme | 0 ± 0 | 0.33 ± 0.33 | 0.33 ± 0.33 | 0.67 ± 0.67 | 0 ± 0 | 0 ± 0 | 0 ± 0 | 0 ± 0 | 0 ± 0 | 0 ± 0 |
| Anthopsis catenata | 0 ± 0 | 0 ± 0 | 0.33 ± 0.33 | 0 ± 0 | 0 ± 0 | 2 ± 2 | 0.67 ± 0.33 | 0 ± 0 | 0 ± 0 | 0 ± 0 |
| Anthostoma decipiens | 10 ± 5.13 | 0 ± 0 | 1.33 ± 0.88 | 0 ± 0 | 1 ± 0.58 | 0 ± 0 | 0 ± 0 | 0 ± 0 | 0 ± 0 | 0 ± 0 |
| Anthostomella sp. | 0 ± 0 | 0 ± 0 | 3.67 ± 3.67 | 0 ± 0 | 0 ± 0 | 0 ± 0 | 0.33 ± 0.33 | 0 ± 0 | 0 ± 0 | 0 ± 0 |
| Aphanocladium album | 0 ± 0 | 2 ± 2 | 0 ± 0 | 0.33 ± 0.33 | 0.33 ± 0.33 | 0.67 ± 0.67 | 2 ± 1.53 | 4 ± 2 | 1.67 ± 1.2 | 1.67 ± 1.67 |
| Aphanophora eugeniae | 0 ± 0 | 0 ± 0 | 0 ± 0 | 0.67 ± 0.67 | 0 ± 0 | 0 ± 0 | 0.67 ± 0.67 | 26 ± 13.01 | 17 ± 16.5 | 5 ± 4.51 |
| Apiosordaria microcarpa | 0 ± 0 | 0.33 ± 0.33 | 0.67 ± 0.67 | 2.67 ± 2.67 | 6 ± 2.52 | 27.33 ± 21.07 | 3.67 ± 1.67 | 58.67 ± 35.3 | 7 ± 7 | 0 ± 0 |
| Apiosordaria verruculosa | 0 ± 0 | 0 ± 0 | 0 ± 0 | 0 ± 0 | 2.67 ± 2.67 | 0.33 ± 0.33 | 0.33 ± 0.33 | 0 ± 0 | 0 ± 0 | 1 ± 1 |
| Apiotrichum dehoogii | 0 ± 0 | 0 ± 0 | 0 ± 0 | 4 ± 4 | 0 ± 0 | 0 ± 0 | 0.67 ± 0.33 | 0 ± 0 | 0 ± 0 | 1 ± 1 |
| Apiotrichum scarabaeorum | 0 ± 0 | 0 ± 0 | 0 ± 0 | 1 ± 1 | 0 ± 0 | 0 ± 0 | 0.67 ± 0.67 | 0 ± 0 | 0 ± 0 | 0 ± 0 |
| Apodus deciduus | 0 ± 0 | 0 ± 0 | 51.67 ± 9.21 | 1 ± 0.58 | 0 ± 0 | 7.33 ± 5.9 | 4 ± 2.65 | 0 ± 0 | 0 ± 0 | 0 ± 0 |
| Apodus oryzae | 1.33 ± 1.33 | 0 ± 0 | 0 ± 0 | 0.67 ± 0.67 | 0 ± 0 | 0.33 ± 0.33 | 0.33 ± 0.33 | 1 ± 0.58 | 0 ± 0 | 1.67 ± 1.67 |
| Apophysomyces ossiformis | 0 ± 0 | 0 ± 0 | 2 ± 1.15 | 0 ± 0 | 0 ± 0 | 0 ± 0 | 0 ± 0 | 0 ± 0 | 0 ± 0 | 0 ± 0 |
| Arachnotheca glomerata | 0 ± 0 | 0 ± 0 | 0 ± 0 | 0 ± 0 | 0 ± 0 | 0 ± 0 | 2.67 ± 2.67 | 1 ± 0.58 | 2.33 ± 2.33 | 0.67 ± 0.67 |
| Archaeorhizomyces borealis | 15795.33 ± 817.23 | 1854 ± 1099.36 | 433.33 ± 36.7 | 1593 ± 121.01 | 16041 ± 465.61 | 450.33 ± 227.46 | 1787 ± 812.71 | 892 ± 866.53 | 2691.67 ± 1372.06 | 100 ± 64.31 |
| Archaeorhizomyces finlayi | 8487.33 ± 898.88 | 48 ± 41.5 | 254.33 ± 73.67 | 44.67 ± 31.17 | 6184.33 ± 287.56 | 171.67 ± 107.44 | 265.33 ± 131.51 | 0 ± 0 | 0.33 ± 0.33 | 5 ± 2.89 |
| Archaeospora schenckii | 0 ± 0 | 0 ± 0 | 0 ± 0 | 0 ± 0 | 0 ± 0 | 0 ± 0 | 0 ± 0 | 0 ± 0 | 1 ± 1 | 0 ± 0 |
| Archaeospora sp. | 0 ± 0 | 3.33 ± 0.67 | 0 ± 0 | 1 ± 1 | 0 ± 0 | 0 ± 0 | 0.33 ± 0.33 | 0 ± 0 | 0 ± 0 | 2.33 ± 1.2 |
| Archaeospora sp. PODO7.3 | 0 ± 0 | 0 ± 0 | 0 ± 0 | 0 ± 0 | 0 ± 0 | 1 ± 1 | 0 ± 0 | 0 ± 0 | 0 ± 0 | 0 ± 0 |
| Archaeospora sp. isa33 | 0 ± 0 | 2 ± 2 | 0 ± 0 | 0.67 ± 0.67 | 0 ± 0 | 0 ± 0 | 0.33 ± 0.33 | 0 ± 0 | 0 ± 0 | 0 ± 0 |
| Archaeospora trappei | 0 ± 0 | 3 ± 3 | 0 ± 0 | 0 ± 0 | 0 ± 0 | 0 ± 0 | 1 ± 1 | 0 ± 0 | 0 ± 0 | 0 ± 0 |
| Aristastoma oeconomicum | 132 ± 126.51 | 3.67 ± 3.67 | 0.67 ± 0.33 | 0 ± 0 | 13 ± 11.53 | 1.33 ± 1.33 | 1.33 ± 0.67 | 0.33 ± 0.33 | 0 ± 0 | 23 ± 20.01 |
| Arizonaphlyctis lemmonensis | 0.33 ± 0.33 | 3.67 ± 3.67 | 0 ± 0 | 0.67 ± 0.67 | 0 ± 0 | 0 ± 0 | 0 ± 0 | 0 ± 0 | 0 ± 0 | 0 ± 0 |
| Arrhenia acerosa | 0 ± 0 | 1.33 ± 1.33 | 0.33 ± 0.33 | 0 ± 0 | 0 ± 0 | 0 ± 0 | 0 ± 0 | 0 ± 0 | 0 ± 0 | 0 ± 0 |
| Arrhenia subglobispora | 1.33 ± 1.33 | 0 ± 0 | 0.67 ± 0.67 | 0.67 ± 0.67 | 0 ± 0 | 1 ± 0.58 | 0.33 ± 0.33 | 18.33 ± 18.33 | 0.67 ± 0.33 | 0 ± 0 |
| Arthopyrenia sp. | 0 ± 0 | 0 ± 0 | 0.67 ± 0.67 | 0 ± 0 | 0 ± 0 | 0 ± 0 | 0 ± 0 | 0 ± 0 | 0 ± 0 | 0 ± 0 |
| Arthrinium arundinis | 0.33 ± 0.33 | 0 ± 0 | 0.67 ± 0.67 | 0 ± 0 | 0 ± 0 | 0 ± 0 | 7.67 ± 7.67 | 10.33 ± 6.74 | 5.67 ± 5.17 | 6 ± 1.53 |
| Arthrinium hydei | 0 ± 0 | 0 ± 0 | 0 ± 0 | 0 ± 0 | 0 ± 0 | 0 ± 0 | 0 ± 0 | 0 ± 0 | 1.33 ± 1.33 | 0 ± 0 |
| Arthrinium phaeospermum | 0 ± 0 | 0 ± 0 | 0 ± 0 | 0 ± 0 | 0 ± 0 | 0 ± 0 | 0 ± 0 | 3 ± 2.52 | 1 ± 1 | 0 ± 0 |
| Arthrinium sp. AX147 | 0 ± 0 | 0 ± 0 | 0 ± 0 | 0 ± 0 | 0 ± 0 | 0 ± 0 | 5.67 ± 5.67 | 0.67 ± 0.67 | 0 ± 0 | 2.67 ± 1.76 |
| Arthrobotrys gephyropaga | 0 ± 0 | 3.67 ± 2.67 | 0 ± 0 | 0 ± 0 | 0 ± 0 | 0 ± 0 | 0.33 ± 0.33 | 41.33 ± 40.83 | 3.67 ± 3.18 | 0 ± 0 |
| Arthrobotrys oligospora | 0.33 ± 0.33 | 0.67 ± 0.67 | 2 ± 0.58 | 0 ± 0 | 0 ± 0 | 13 ± 9.29 | 0 ± 0 | 2 ± 1 | 1 ± 0.58 | 0.33 ± 0.33 |
| Arthrobotrys thaumasia | 0 ± 0 | 6 ± 3.51 | 0 ± 0 | 0.33 ± 0.33 | 0 ± 0 | 0.67 ± 0.67 | 0.33 ± 0.33 | 0.67 ± 0.67 | 1.67 ± 1.67 | 4 ± 2.65 |
| Arthrobotrys xiangyunensis | 1 ± 1 | 0 ± 0 | 0 ± 0 | 0 ± 0 | 0 ± 0 | 6.67 ± 3.53 | 0.33 ± 0.33 | 0 ± 0 | 0 ± 0 | 0 ± 0 |
| Arthrocladium caudatum | 0.67 ± 0.67 | 0 ± 0 | 1 ± 1 | 0 ± 0 | 1.33 ± 0.33 | 1.33 ± 0.67 | 0 ± 0 | 4.67 ± 2.6 | 3.67 ± 3.67 | 2 ± 1.15 |
| Arthropsis truncata | 0.67 ± 0.67 | 25.67 ± 12.39 | 0.33 ± 0.33 | 6 ± 1.73 | 0 ± 0 | 3 ± 2.08 | 13 ± 10.15 | 1 ± 1 | 3.67 ± 2.73 | 0 ± 0 |
| Articulospora proliferata | 0.67 ± 0.33 | 0 ± 0 | 10.33 ± 4.37 | 0.33 ± 0.33 | 0.67 ± 0.33 | 6.67 ± 3.53 | 7 ± 0.58 | 8.33 ± 5.61 | 3 ± 1.73 | 5.33 ± 2.33 |
| Arxiella dolichandrae | 0 ± 0 | 1.33 ± 1.33 | 0 ± 0 | 0 ± 0 | 0 ± 0 | 0 ± 0 | 0.33 ± 0.33 | 1.33 ± 1.33 | 3.67 ± 3.18 | 0 ± 0 |
| Arxiella terrestris | 7.67 ± 3.71 | 673.33 ± 634.64 | 88 ± 67.55 | 30 ± 24.7 | 3.33 ± 1.45 | 9.33 ± 7.42 | 285 ± 280.01 | 76.33 ± 37.83 | 52 ± 51 | 45.67 ± 20.43 |
| Aschersonia sp. | 0 ± 0 | 0 ± 0 | 0 ± 0 | 0 ± 0 | 0 ± 0 | 0.33 ± 0.33 | 0 ± 0 | 0 ± 0 | 0 ± 0 | 0 ± 0 |
| Ascitendus austriacus | 0 ± 0 | 0 ± 0 | 0 ± 0 | 0 ± 0 | 0 ± 0 | 2.33 ± 1.45 | 1.67 ± 1.67 | 0 ± 0 | 0 ± 0 | 0 ± 0 |
| Ascobolus crenulatus | 0 ± 0 | 0 ± 0 | 0 ± 0 | 0 ± 0 | 0 ± 0 | 1.67 ± 1.67 | 0 ± 0 | 0 ± 0 | 0 ± 0 | 0 ± 0 |
| Ascodesmis sphaerospora | 0 ± 0 | 1 ± 0.58 | 0 ± 0 | 0 ± 0 | 0.33 ± 0.33 | 0 ± 0 | 0 ± 0 | 2.33 ± 2.33 | 1.67 ± 1.2 | 0 ± 0 |
| Ascorhizoctonia sp. ZC-W-2-2 | 14.67 ± 9.39 | 0.33 ± 0.33 | 0 ± 0 | 0 ± 0 | 0.33 ± 0.33 | 0 ± 0 | 0.33 ± 0.33 | 0 ± 0 | 0 ± 0 | 0 ± 0 |
| Ascosphaera aggregata | 0 ± 0 | 0 ± 0 | 0 ± 0 | 0 ± 0 | 0 ± 0 | 0 ± 0 | 0 ± 0 | 4 ± 2.65 | 0 ± 0 | 0 ± 0 |
| Ascotaiwania sawadae | 0.33 ± 0.33 | 0 ± 0 | 11.67 ± 4.37 | 0.33 ± 0.33 | 0.33 ± 0.33 | 13.33 ± 9.94 | 3 ± 2.08 | 2.67 ± 2.67 | 0 ± 0 | 1.67 ± 0.88 |
| Aspergillus aculeatus | 0 ± 0 | 0 ± 0 | 0 ± 0 | 0 ± 0 | 0 ± 0 | 0 ± 0 | 0 ± 0 | 0.67 ± 0.33 | 0.33 ± 0.33 | 0.67 ± 0.67 |
| Aspergillus alliaceus | 0 ± 0 | 0 ± 0 | 0 ± 0 | 0 ± 0 | 0 ± 0 | 0 ± 0 | 0 ± 0 | 0 ± 0 | 0.67 ± 0.33 | 15.67 ± 7.06 |
| Aspergillus asperescens | 0 ± 0 | 0 ± 0 | 0 ± 0 | 0 ± 0 | 0 ± 0 | 0.67 ± 0.67 | 0 ± 0 | 0 ± 0 | 0 ± 0 | 0 ± 0 |
| Aspergillus bicolor | 0 ± 0 | 0 ± 0 | 0 ± 0 | 0 ± 0 | 0 ± 0 | 0 ± 0 | 0 ± 0 | 0 ± 0 | 0 ± 0 | 0 ± 0 |
| Aspergillus brunneouniseriatus | 0 ± 0 | 0 ± 0 | 0 ± 0 | 0 ± 0 | 0.67 ± 0.33 | 0 ± 0 | 0 ± 0 | 0 ± 0 | 0 ± 0 | 0 ± 0 |
| Aspergillus cejpii | 0 ± 0 | 0 ± 0 | 0 ± 0 | 0 ± 0 | 0.67 ± 0.67 | 0 ± 0 | 0 ± 0 | 0 ± 0 | 0 ± 0 | 0 ± 0 |
| Aspergillus felis | 0 ± 0 | 0 ± 0 | 0.33 ± 0.33 | 0 ± 0 | 0 ± 0 | 0.67 ± 0.67 | 0 ± 0 | 0 ± 0 | 0.67 ± 0.67 | 0 ± 0 |
| Aspergillus flavipes | 0 ± 0 | 11.67 ± 6.39 | 0 ± 0 | 1.33 ± 1.33 | 0.33 ± 0.33 | 0 ± 0 | 4.33 ± 4.33 | 17.67 ± 8.84 | 0.67 ± 0.67 | 1 ± 1 |
| Aspergillus inflatus | 0 ± 0 | 0 ± 0 | 0 ± 0 | 2.67 ± 1.33 | 0 ± 0 | 0 ± 0 | 1 ± 0.58 | 0 ± 0 | 0 ± 0 | 0 ± 0 |
| Aspergillus japonicus | 0 ± 0 | 0 ± 0 | 0 ± 0 | 0 ± 0 | 0 ± 0 | 1.67 ± 1.67 | 0 ± 0 | 0 ± 0 | 0 ± 0 | 0 ± 0 |
| Aspergillus luteovirescens | 0 ± 0 | 0 ± 0 | 0 ± 0 | 0 ± 0 | 0 ± 0 | 0 ± 0 | 0 ± 0 | 0 ± 0 | 0.67 ± 0.67 | 0 ± 0 |
| Aspergillus nidulans | 0 ± 0 | 0 ± 0 | 0.33 ± 0.33 | 0 ± 0 | 0 ± 0 | 81.67 ± 77.18 | 0 ± 0 | 0 ± 0 | 0.67 ± 0.67 | 471 ± 471 |
| Aspergillus penicillioides | 0 ± 0 | 0 ± 0 | 0.67 ± 0.67 | 0 ± 0 | 0 ± 0 | 1.33 ± 0.67 | 0 ± 0 | 1.33 ± 1.33 | 0 ± 0 | 0 ± 0 |
| Aspergillus proliferans | 0 ± 0 | 0 ± 0 | 0 ± 0 | 0.67 ± 0.67 | 0 ± 0 | 0 ± 0 | 0 ± 0 | 0 ± 0 | 0 ± 0 | 1 ± 1 |
| Aspergillus purpureus | 0 ± 0 | 0 ± 0 | 44.33 ± 6.06 | 0.33 ± 0.33 | 0 ± 0 | 19.67 ± 16.29 | 2 ± 1 | 0 ± 0 | 0 ± 0 | 0 ± 0 |
| Aspergillus sclerotiorum | 0 ± 0 | 3.33 ± 2.33 | 0 ± 0 | 0 ± 0 | 0 ± 0 | 0 ± 0 | 0.33 ± 0.33 | 0 ± 0 | 0 ± 0 | 0 ± 0 |
| Aspergillus sp. | 0 ± 0 | 0 ± 0 | 0.67 ± 0.67 | 0 ± 0 | 0 ± 0 | 0.67 ± 0.67 | 0 ± 0 | 0.67 ± 0.67 | 0 ± 0 | 0 ± 0 |
| Aspergillus sp. BMP3043 | 3.33 ± 0.67 | 0.33 ± 0.33 | 0 ± 0 | 0.33 ± 0.33 | 0.67 ± 0.33 | 0.33 ± 0.33 | 4 ± 2.52 | 59.33 ± 31.06 | 22.33 ± 22.33 | 0.33 ± 0.33 |
| Aspergillus uvarum | 0 ± 0 | 0 ± 0 | 0 ± 0 | 0 ± 0 | 0 ± 0 | 0.67 ± 0.67 | 0 ± 0 | 0 ± 0 | 0 ± 0 | 0 ± 0 |
| Aspergillus versicolor | 0 ± 0 | 0 ± 0 | 1.67 ± 0.88 | 0 ± 0 | 0 ± 0 | 1 ± 1 | 0 ± 0 | 0 ± 0 | 0 ± 0 | 0 ± 0 |
| Aspergillus vitricola | 0 ± 0 | 0 ± 0 | 0 ± 0 | 0 ± 0 | 0 ± 0 | 0.33 ± 0.33 | 0 ± 0 | 0 ± 0 | 0 ± 0 | 0 ± 0 |
| Astraeus hygrometricus | 0 ± 0 | 0 ± 0 | 0.67 ± 0.67 | 0 ± 0 | 0 ± 0 | 0 ± 0 | 0 ± 0 | 0 ± 0 | 0 ± 0 | 0 ± 0 |
| Astrocystis sublimbata | 0 ± 0 | 0 ± 0 | 1.33 ± 1.33 | 0 ± 0 | 0 ± 0 | 0 ± 0 | 1.33 ± 0.88 | 0 ± 0 | 0 ± 0 | 0.33 ± 0.33 |
| Atractiella rhizophila | 1 ± 1 | 281.67 ± 149.45 | 0 ± 0 | 19.67 ± 5.84 | 0 ± 0 | 0.67 ± 0.67 | 53.67 ± 38.37 | 0 ± 0 | 0 ± 0 | 0.33 ± 0.33 |
| Atractiella sp. | 0 ± 0 | 0 ± 0 | 0 ± 0 | 0 ± 0 | 0 ± 0 | 0 ± 0 | 0 ± 0 | 2.33 ± 2.33 | 0 ± 0 | 0 ± 0 |
| Atractium crassum | 0.67 ± 0.33 | 0 ± 0 | 3.67 ± 1.76 | 3 ± 1.73 | 11.67 ± 3.84 | 1 ± 0 | 2.33 ± 1.2 | 0 ± 0 | 0 ± 0 | 1.67 ± 0.88 |
| Atractospora decumbens | 0 ± 0 | 0 ± 0 | 6 ± 3.06 | 4.67 ± 3.67 | 0 ± 0 | 49.67 ± 27.91 | 5 ± 2.89 | 0 ± 0 | 0 ± 0 | 43.67 ± 21.42 |
| Atractospora verruculosa | 0 ± 0 | 0 ± 0 | 0 ± 0 | 0 ± 0 | 0 ± 0 | 0 ± 0 | 0 ± 0 | 23.67 ± 23.67 | 0.33 ± 0.33 | 0.33 ± 0.33 |
| Atrocalyx bambusae | 0 ± 0 | 0 ± 0 | 0 ± 0 | 0 ± 0 | 0 ± 0 | 130 ± 118.63 | 1 ± 1 | 0 ± 0 | 0 ± 0 | 2 ± 2 |
| Aureobasidium leucospermi | 0.33 ± 0.33 | 0 ± 0 | 1 ± 1 | 0 ± 0 | 0 ± 0 | 0.33 ± 0.33 | 0 ± 0 | 3 ± 1.73 | 0 ± 0 | 0 ± 0 |
| Auriculoscypha anacardiicola | 0.33 ± 0.33 | 0 ± 0 | 0 ± 0 | 0 ± 0 | 5.33 ± 3.93 | 0 ± 0 | 0.33 ± 0.33 | 0 ± 0 | 0 ± 0 | 0 ± 0 |
| Austroafricana associata | 0 ± 0 | 0 ± 0 | 1.33 ± 1.33 | 0 ± 0 | 0 ± 0 | 0 ± 0 | 1 ± 1 | 0 ± 0 | 0 ± 0 | 0 ± 0 |
| Auxarthron alboluteum | 0 ± 0 | 0 ± 0 | 0 ± 0 | 0 ± 0 | 0 ± 0 | 0 ± 0 | 0 ± 0 | 0 ± 0 | 0 ± 0 | 2 ± 1.15 |
| Auxarthron kuehnii | 1 ± 0.58 | 0 ± 0 | 0 ± 0 | 0.33 ± 0.33 | 2.33 ± 0.88 | 0 ± 0 | 0 ± 0 | 0 ± 0 | 0 ± 0 | 4 ± 1.73 |
| Auxarthron sp. | 0 ± 0 | 0 ± 0 | 0 ± 0 | 0 ± 0 | 0 ± 0 | 0 ± 0 | 3 ± 2.52 | 0.67 ± 0.67 | 0 ± 0 | 4.67 ± 4.18 |
| Bacidia sp. WSL DF72 | 0 ± 0 | 0 ± 0 | 0 ± 0 | 0 ± 0 | 0 ± 0 | 0 ± 0 | 0 ± 0 | 0 ± 0 | 0 ± 0 | 0 ± 0 |
| Bacidia vermifera | 0 ± 0 | 0 ± 0 | 0 ± 0 | 0 ± 0 | 0 ± 0 | 0 ± 0 | 0 ± 0 | 0 ± 0 | 0 ± 0 | 0.33 ± 0.33 |
| Bacidina flavoleprosa | 0 ± 0 | 0 ± 0 | 0.67 ± 0.67 | 0 ± 0 | 0 ± 0 | 0 ± 0 | 0 ± 0 | 0.67 ± 0.67 | 8 ± 6.11 | 0 ± 0 |
| Bacidina mendax | 0 ± 0 | 0 ± 0 | 0 ± 0 | 3.33 ± 3.33 | 0 ± 0 | 0 ± 0 | 0.67 ± 0.67 | 0 ± 0 | 0 ± 0 | 0 ± 0 |
| Bacidina sulphurella | 0.67 ± 0.67 | 0 ± 0 | 0 ± 0 | 0 ± 0 | 0.33 ± 0.33 | 0 ± 0 | 0 ± 0 | 0 ± 0 | 0 ± 0 | 0 ± 0 |
| Bacillicladium lobatum | 0 ± 0 | 0 ± 0 | 5 ± 4.04 | 0 ± 0 | 0 ± 0 | 0.33 ± 0.33 | 0 ± 0 | 0 ± 0 | 0 ± 0 | 0 ± 0 |
| Bactrodesmium longisporum | 0 ± 0 | 0 ± 0 | 0 ± 0 | 0 ± 0 | 0 ± 0 | 0 ± 0 | 1.67 ± 1.67 | 0 ± 0 | 0 ± 0 | 2 ± 2 |
| Bactrodesmium obovatum | 0 ± 0 | 0 ± 0 | 0 ± 0 | 0 ± 0 | 0 ± 0 | 0 ± 0 | 0 ± 0 | 3.33 ± 3.33 | 0 ± 0 | 1 ± 1 |
| Bagliettoa baldensis | 0 ± 0 | 0 ± 0 | 0 ± 0 | 0 ± 0 | 0 ± 0 | 0 ± 0 | 0 ± 0 | 0.67 ± 0.67 | 2.33 ± 1.45 | 0 ± 0 |
| Bagliettoa cazzae | 0 ± 0 | 0 ± 0 | 0 ± 0 | 0 ± 0 | 0 ± 0 | 0 ± 0 | 0.67 ± 0.67 | 0 ± 0 | 0 ± 0 | 0 ± 0 |
| Bagliettoa marmorea | 0 ± 0 | 0 ± 0 | 0 ± 0 | 0 ± 0 | 0 ± 0 | 0 ± 0 | 0 ± 0 | 0 ± 0 | 0 ± 0 | 0 ± 0 |
| Bagnisiella sp. CBS 513.84 | 0 ± 0 | 0 ± 0 | 0 ± 0 | 0 ± 0 | 0 ± 0 | 0 ± 0 | 0 ± 0 | 1.67 ± 1.67 | 0 ± 0 | 0 ± 0 |
| Banksiophoma australiensis | 0 ± 0 | 0 ± 0 | 0 ± 0 | 0 ± 0 | 0 ± 0 | 0.33 ± 0.33 | 0.33 ± 0.33 | 0 ± 0 | 0.67 ± 0.33 | 0 ± 0 |
| Bannoa hahajimensis | 0 ± 0 | 0 ± 0 | 3.33 ± 3.33 | 0 ± 0 | 0 ± 0 | 0 ± 0 | 1 ± 0.58 | 0 ± 0 | 0 ± 0 | 0 ± 0 |
| Basidioascus persicus | 0 ± 0 | 0 ± 0 | 0 ± 0 | 0 ± 0 | 0 ± 0 | 0.33 ± 0.33 | 0 ± 0 | 0 ± 0 | 0 ± 0 | 0 ± 0 |
| Basidioascus undulatus | 0 ± 0 | 0 ± 0 | 4.67 ± 2.19 | 0 ± 0 | 1.33 ± 0.88 | 0 ± 0 | 0 ± 0 | 0 ± 0 | 0 ± 0 | 0 ± 0 |
| Basidiobolus haptosporus | 0 ± 0 | 0 ± 0 | 0 ± 0 | 0 ± 0 | 0 ± 0 | 0 ± 0 | 13.67 ± 13.67 | 0 ± 0 | 0.33 ± 0.33 | 0 ± 0 |
| Basidiobolus ranarum | 0 ± 0 | 0 ± 0 | 0 ± 0 | 0 ± 0 | 2.33 ± 2.33 | 0 ± 0 | 0 ± 0 | 0 ± 0 | 0 ± 0 | 0 ± 0 |
| Basidiobolus sp. ARSEF 5903 | 0 ± 0 | 0 ± 0 | 0 ± 0 | 0 ± 0 | 0 ± 0 | 0 ± 0 | 0 ± 0 | 0 ± 0 | 1 ± 1 | 0 ± 0 |
| Batcheloromyces leucadendri | 0 ± 0 | 6 ± 2.08 | 0 ± 0 | 0.67 ± 0.67 | 0 ± 0 | 0 ± 0 | 2 ± 0.58 | 13 ± 13 | 67.67 ± 58.42 | 55.33 ± 37.24 |
| Beauveria bassiana | 15.33 ± 7.84 | 5.67 ± 2.4 | 96.33 ± 10.53 | 101.33 ± 48.34 | 14.67 ± 8.69 | 38.33 ± 33.35 | 122 ± 15.95 | 26.67 ± 11.14 | 14 ± 10.02 | 2 ± 1 |
| Beauveria brongniartii | 0 ± 0 | 0.33 ± 0.33 | 1.67 ± 1.67 | 1 ± 0.58 | 0 ± 0 | 1.67 ± 1.2 | 0 ± 0 | 0.67 ± 0.67 | 0 ± 0 | 15.67 ± 4.67 |
| Beauveria felina | 0 ± 0 | 0 ± 0 | 0 ± 0 | 0.67 ± 0.67 | 0 ± 0 | 1.67 ± 1.67 | 0 ± 0 | 0 ± 0 | 0 ± 0 | 0.33 ± 0.33 |
| Beauveria tenella | 3.67 ± 2.19 | 0.33 ± 0.33 | 8 ± 3.46 | 52 ± 26.84 | 9.67 ± 3.84 | 56.33 ± 10.09 | 101 ± 17.04 | 13.67 ± 7.54 | 11 ± 9.54 | 119.67 ± 25.83 |
| Beltrania sp. 2 RV-2015 | 0 ± 0 | 0 ± 0 | 0 ± 0 | 0 ± 0 | 0 ± 0 | 5.67 ± 5.67 | 1 ± 1 | 1.33 ± 1.33 | 1 ± 1 | 6.67 ± 5.17 |
| Beltraniella sp. 37.2.1 | 0 ± 0 | 0 ± 0 | 0 ± 0 | 0 ± 0 | 0 ± 0 | 0 ± 0 | 0 ± 0 | 0 ± 0 | 0 ± 0 | 0 ± 0 |
| Berkleasmium sp. X18 | 1.67 ± 0.33 | 0 ± 0 | 0 ± 0 | 0 ± 0 | 0 ± 0 | 0 ± 0 | 0 ± 0 | 0 ± 0 | 0 ± 0 | 0 ± 0 |
| Betamyces americaemeridionalis | 0 ± 0 | 0 ± 0 | 2.33 ± 0.67 | 0.67 ± 0.67 | 0 ± 0 | 1.33 ± 1.33 | 0.67 ± 0.67 | 0 ± 0 | 0 ± 0 | 0 ± 0 |
| Betamyces sp. PL 173 | 0 ± 0 | 2.67 ± 2.67 | 0 ± 0 | 0.67 ± 0.67 | 0 ± 0 | 0 ± 0 | 0 ± 0 | 0.33 ± 0.33 | 0 ± 0 | 0 ± 0 |
| Bettsia alvei | 0 ± 0 | 0.33 ± 0.33 | 0 ± 0 | 10.33 ± 2.4 | 0.67 ± 0.67 | 0 ± 0 | 2 ± 1 | 0 ± 0 | 0 ± 0 | 0 ± 0 |
| Bettsia fastidia | 0 ± 0 | 0 ± 0 | 0 ± 0 | 0 ± 0 | 0 ± 0 | 0 ± 0 | 0 ± 0 | 0 ± 0 | 0 ± 0 | 0 ± 0 |
| Beverwykella pulmonaria | 0 ± 0 | 0 ± 0 | 0 ± 0 | 0 ± 0 | 0.33 ± 0.33 | 0.33 ± 0.33 | 0.33 ± 0.33 | 0 ± 0 | 0 ± 0 | 0 ± 0 |
| Biappendiculispora japonica | 0 ± 0 | 7.67 ± 3.48 | 0 ± 0 | 0.33 ± 0.33 | 0 ± 0 | 0 ± 0 | 0.67 ± 0.67 | 0 ± 0 | 0 ± 0 | 0 ± 0 |
| Biatriospora sp. | 0 ± 0 | 0 ± 0 | 0 ± 0 | 0 ± 0 | 0 ± 0 | 29.67 ± 16.5 | 0 ± 0 | 0 ± 0 | 0 ± 0 | 0 ± 0 |
| Biatriospora sp. 1 NV-2015 | 0 ± 0 | 0 ± 0 | 0 ± 0 | 0 ± 0 | 0 ± 0 | 0.33 ± 0.33 | 0 ± 0 | 0 ± 0 | 0 ± 0 | 0 ± 0 |
| Bipolaris axonopicola | 0 ± 0 | 6.67 ± 5.7 | 0 ± 0 | 0.33 ± 0.33 | 0 ± 0 | 0 ± 0 | 0.33 ± 0.33 | 0 ± 0 | 0 ± 0 | 0 ± 0 |
| Bipolaris sorokiniana | 0 ± 0 | 0 ± 0 | 0 ± 0 | 0 ± 0 | 0 ± 0 | 1 ± 1 | 0 ± 0 | 0 ± 0 | 0 ± 0 | 0 ± 0 |
| Bloxamia sp. TNS:F24589 | 0 ± 0 | 0.33 ± 0.33 | 0 ± 0 | 2 ± 1.15 | 0 ± 0 | 3 ± 1.73 | 18.33 ± 15.84 | 1 ± 0.58 | 2.67 ± 2.67 | 0.33 ± 0.33 |
| Bloxamia truncata | 0 ± 0 | 0 ± 0 | 0 ± 0 | 0 ± 0 | 0 ± 0 | 1.33 ± 1.33 | 0.33 ± 0.33 | 0 ± 0 | 0 ± 0 | 0 ± 0 |
| Bolbitius bisporus | 0 ± 0 | 0 ± 0 | 0.67 ± 0.67 | 0 ± 0 | 0 ± 0 | 0 ± 0 | 0 ± 0 | 0 ± 0 | 0 ± 0 | 0 ± 0 |
| Boletus pallidus | 0 ± 0 | 0 ± 0 | 0 ± 0 | 0 ± 0 | 0 ± 0 | 0 ± 0 | 0 ± 0 | 0 ± 0 | 0 ± 0 | 2 ± 2 |
| Botryoderma lateritium | 0 ± 0 | 1.33 ± 0.88 | 0 ± 0 | 0.67 ± 0.67 | 0 ± 0 | 0.33 ± 0.33 | 3 ± 3 | 0 ± 0 | 0.33 ± 0.33 | 7 ± 6.51 |
| Botryohypochnus aff. isabellinus UC2023036 | 0 ± 0 | 0 ± 0 | 0 ± 0 | 0 ± 0 | 0 ± 0 | 0 ± 0 | 0 ± 0 | 3.33 ± 3.33 | 8.67 ± 5.55 | 0 ± 0 |
| Botryosphaeria cf. protearum CBS 119220 | 0 ± 0 | 0 ± 0 | 0 ± 0 | 0.33 ± 0.33 | 0 ± 0 | 0 ± 0 | 0.67 ± 0.67 | 0.33 ± 0.33 | 1 ± 0.58 | 0 ± 0 |
| Botryosphaeria sinensis | 0 ± 0 | 0 ± 0 | 0 ± 0 | 0 ± 0 | 0 ± 0 | 0 ± 0 | 0 ± 0 | 1 ± 1 | 0 ± 0 | 0 ± 0 |
| Botryotrichum piluliferum | 3.67 ± 3.67 | 0 ± 0 | 3.67 ± 3.67 | 0 ± 0 | 0 ± 0 | 13.67 ± 8.09 | 21.33 ± 20.34 | 3 ± 1 | 0.33 ± 0.33 | 22 ± 22 |
| Botrytis cinerea | 0 ± 0 | 0 ± 0 | 7.33 ± 4.33 | 1 ± 1 | 0 ± 0 | 9 ± 4.93 | 2.67 ± 2.19 | 73 ± 46.14 | 12.67 ± 11.68 | 0 ± 0 |
| Boubovia vermiphila | 2.33 ± 1.33 | 0.67 ± 0.67 | 0 ± 0 | 0 ± 0 | 0 ± 0 | 2 ± 1.53 | 0 ± 0 | 0.33 ± 0.33 | 0 ± 0 | 0 ± 0 |
| Brachyphoris sp. 'tenuifusaria' | 0 ± 0 | 0 ± 0 | 0 ± 0 | 0 ± 0 | 0 ± 0 | 0 ± 0 | 0 ± 0 | 3 ± 1.53 | 0.33 ± 0.33 | 0 ± 0 |
| Bryochiton sp. PW-2014 | 0 ± 0 | 0 ± 0 | 0 ± 0 | 0 ± 0 | 0 ± 0 | 0 ± 0 | 0 ± 0 | 0 ± 0 | 1 ± 1 | 0 ± 0 |
| Buckleyzyma aurantiaca | 0.33 ± 0.33 | 0 ± 0 | 0 ± 0 | 0 ± 0 | 0 ± 0 | 0.33 ± 0.33 | 0 ± 0 | 1.33 ± 1.33 | 0 ± 0 | 0.33 ± 0.33 |
| Buckleyzyma phyllomatis | 0 ± 0 | 0 ± 0 | 0 ± 0 | 0 ± 0 | 0 ± 0 | 0 ± 0 | 0.67 ± 0.67 | 0 ± 0 | 0 ± 0 | 0 ± 0 |
| Bullera alba | 1.67 ± 1.67 | 0 ± 0 | 22.33 ± 22.33 | 0 ± 0 | 0.33 ± 0.33 | 0.67 ± 0.67 | 2 ± 1.15 | 0 ± 0 | 0 ± 0 | 0.33 ± 0.33 |
| Bulleribasidium pseudovariabile | 0 ± 0 | 0 ± 0 | 0 ± 0 | 0 ± 0 | 0 ± 0 | 0 ± 0 | 0 ± 0 | 0 ± 0 | 0 ± 0 | 1 ± 1 |
| Bulleribasidium variabile | 1 ± 1 | 0 ± 0 | 40 ± 24.11 | 0.33 ± 0.33 | 0.33 ± 0.33 | 0.67 ± 0.33 | 1.67 ± 0.88 | 0 ± 0 | 0 ± 0 | 5.33 ± 2.91 |
| Bulleribasidium wuzhishanense | 0 ± 0 | 0.67 ± 0.33 | 0 ± 0 | 34.67 ± 24.73 | 0 ± 0 | 1.33 ± 1.33 | 10.33 ± 1.45 | 0.33 ± 0.33 | 0 ± 0 | 1.67 ± 1.67 |
| Burgoa anomala | 1.67 ± 1.67 | 0 ± 0 | 0 ± 0 | 0 ± 0 | 0 ± 0 | 0 ± 0 | 0 ± 0 | 0 ± 0 | 0 ± 0 | 0 ± 0 |
| Byssochlamys spectabilis | 0 ± 0 | 0 ± 0 | 0.67 ± 0.33 | 0 ± 0 | 0 ± 0 | 0.67 ± 0.67 | 0 ± 0 | 0 ± 0 | 0 ± 0 | 0 ± 0 |
| Byssochlamys verrucosa | 0 ± 0 | 0 ± 0 | 0 ± 0 | 0 ± 0 | 0 ± 0 | 0 ± 0 | 8.33 ± 8.33 | 0.33 ± 0.33 | 1.67 ± 0.88 | 0 ± 0 |
| Byssocorticium sp. HA6 | 0 ± 0 | 0.67 ± 0.67 | 0 ± 0 | 0 ± 0 | 0 ± 0 | 0 ± 0 | 0 ± 0 | 0 ± 0 | 0 ± 0 | 0 ± 0 |
| Byssonectria fusispora | 0 ± 0 | 3.67 ± 1.45 | 0.33 ± 0.33 | 0 ± 0 | 0 ± 0 | 0 ± 0 | 0.33 ± 0.33 | 0 ± 0 | 0 ± 0 | 5.33 ± 4.84 |
| Cadophora orchidicola | 0.33 ± 0.33 | 16.67 ± 10.27 | 5 ± 1.73 | 1.67 ± 0.33 | 0 ± 0 | 3.67 ± 3.67 | 24.67 ± 20.74 | 3.33 ± 1.67 | 2 ± 1 | 21.67 ± 12.41 |
| Cadophora sp. | 7 ± 0.58 | 1 ± 1 | 2.67 ± 0.88 | 0 ± 0 | 0.33 ± 0.33 | 1 ± 0.58 | 3.67 ± 3.18 | 53.67 ± 32.3 | 12.33 ± 6.17 | 14 ± 8.08 |
| Cainia desmazieri | 0 ± 0 | 0 ± 0 | 0 ± 0 | 0 ± 0 | 0.33 ± 0.33 | 0 ± 0 | 0 ± 0 | 0.33 ± 0.33 | 0 ± 0 | 0 ± 0 |
| Calcarisporiella sp. NUH37 | 0 ± 0 | 0 ± 0 | 0 ± 0 | 0 ± 0 | 0 ± 0 | 0 ± 0 | 0 ± 0 | 0 ± 0 | 0 ± 0 | 1.33 ± 0.88 |
| Calocera cornea | 0.67 ± 0.67 | 0 ± 0 | 0 ± 0 | 0 ± 0 | 0 ± 0 | 0 ± 0 | 0 ± 0 | 0 ± 0 | 0 ± 0 | 0 ± 0 |
| Calonectria colhounii | 0 ± 0 | 0 ± 0 | 0 ± 0 | 2.67 ± 1.45 | 0 ± 0 | 0 ± 0 | 1 ± 1 | 0 ± 0 | 0 ± 0 | 0 ± 0 |
| Calvolachnella guaviyunis | 4 ± 0.58 | 0 ± 0 | 0 ± 0 | 0 ± 0 | 0 ± 0 | 0 ± 0 | 3.67 ± 3.67 | 0 ± 0 | 0 ± 0 | 0 ± 0 |
| Calycellina populina | 0 ± 0 | 0 ± 0 | 0.33 ± 0.33 | 1.33 ± 0.33 | 0 ± 0 | 0 ± 0 | 0.33 ± 0.33 | 0 ± 0 | 0 ± 0 | 0 ± 0 |
| Calycina marina | 0 ± 0 | 0 ± 0 | 0 ± 0 | 0 ± 0 | 0 ± 0 | 0 ± 0 | 0 ± 0 | 0 ± 0 | 0.67 ± 0.67 | 0 ± 0 |
| Calyptella capula | 0 ± 0 | 0 ± 0 | 1.33 ± 1.33 | 0 ± 0 | 0 ± 0 | 0 ± 0 | 0 ± 0 | 0 ± 0 | 0 ± 0 | 0 ± 0 |
| Calyptella sp. | 0 ± 0 | 0 ± 0 | 0 ± 0 | 0 ± 0 | 0 ± 0 | 0.67 ± 0.67 | 0 ± 0 | 0 ± 0 | 0 ± 0 | 0 ± 0 |
| Calyptella sp. Aronsen120826/2 | 22 ± 12.42 | 0.33 ± 0.33 | 0 ± 0 | 0 ± 0 | 2 ± 1.15 | 0.33 ± 0.33 | 0 ± 0 | 0 ± 0 | 0 ± 0 | 0 ± 0 |
| Camaropella pugillus | 0 ± 0 | 0 ± 0 | 0 ± 0 | 0 ± 0 | 0 ± 0 | 0 ± 0 | 0 ± 0 | 0 ± 0 | 0 ± 0 | 0 ± 0 |
| Camposporium ramosum | 0 ± 0 | 0 ± 0 | 4 ± 4 | 0 ± 0 | 0 ± 0 | 0 ± 0 | 0 ± 0 | 1 ± 0.58 | 0 ± 0 | 71.67 ± 69.67 |
| Camptophora schimae | 0 ± 0 | 0.33 ± 0.33 | 0 ± 0 | 1 ± 1 | 0 ± 0 | 0 ± 0 | 1 ± 0.58 | 11.67 ± 5.46 | 9 ± 9 | 11 ± 5.69 |
| Campylocarpon fasciculare | 1 ± 0.58 | 0 ± 0 | 0 ± 0 | 0 ± 0 | 0 ± 0 | 0 ± 0 | 0 ± 0 | 0 ± 0 | 0 ± 0 | 0 ± 0 |
| Canalisporium sp. SS03732 | 0 ± 0 | 0 ± 0 | 0 ± 0 | 0 ± 0 | 0 ± 0 | 1.67 ± 1.67 | 0 ± 0 | 0 ± 0 | 0 ± 0 | 0 ± 0 |
| Candelariella aurella | 0 ± 0 | 0 ± 0 | 0 ± 0 | 0 ± 0 | 0 ± 0 | 0 ± 0 | 0 ± 0 | 0 ± 0 | 2.33 ± 2.33 | 0 ± 0 |
| Candida tropicalis | 0 ± 0 | 0 ± 0 | 0 ± 0 | 0 ± 0 | 0 ± 0 | 0.33 ± 0.33 | 0 ± 0 | 0 ± 0 | 0.67 ± 0.67 | 0 ± 0 |
| Capnobotryella sp. MA 3619 | 0 ± 0 | 0 ± 0 | 0 ± 0 | 0 ± 0 | 0 ± 0 | 0 ± 0 | 0 ± 0 | 0 ± 0 | 0 ± 0 | 0 ± 0 |
| Capnobotryella sp. MA 4674 | 0 ± 0 | 0 ± 0 | 0 ± 0 | 0 ± 0 | 0 ± 0 | 0 ± 0 | 0 ± 0 | 0 ± 0 | 0 ± 0 | 0.33 ± 0.33 |
| Capnodium sp. | 0 ± 0 | 0 ± 0 | 1.33 ± 1.33 | 0 ± 0 | 0 ± 0 | 0 ± 0 | 0 ± 0 | 0 ± 0 | 0 ± 0 | 0 ± 0 |
| Capronia pilosella | 0 ± 0 | 0 ± 0 | 0 ± 0 | 0 ± 0 | 0 ± 0 | 0 ± 0 | 0 ± 0 | 2 ± 2 | 0 ± 0 | 0 ± 0 |
| Capronia sp. 94003b | 0 ± 0 | 0 ± 0 | 0 ± 0 | 0 ± 0 | 0 ± 0 | 1.67 ± 0.88 | 0 ± 0 | 0 ± 0 | 0 ± 0 | 0 ± 0 |
| Capronia sp. 96003a | 0 ± 0 | 22 ± 20.52 | 0 ± 0 | 6 ± 6 | 0 ± 0 | 0 ± 0 | 3.33 ± 3.33 | 7 ± 6.51 | 24 ± 12.06 | 0 ± 0 |
| Capronia sp. BRO-2013 | 0.33 ± 0.33 | 0 ± 0 | 0 ± 0 | 0 ± 0 | 0.33 ± 0.33 | 0.33 ± 0.33 | 2.67 ± 2.67 | 6.33 ± 4.37 | 7 ± 7 | 7.33 ± 7.33 |
| Castanediella couratarii | 1 ± 0.58 | 0 ± 0 | 0 ± 0 | 0 ± 0 | 0 ± 0 | 0 ± 0 | 0 ± 0 | 0 ± 0 | 0 ± 0 | 0 ± 0 |
| Castanediella sp. | 0 ± 0 | 0 ± 0 | 0 ± 0 | 0 ± 0 | 0 ± 0 | 0 ± 0 | 0 ± 0 | 0.33 ± 0.33 | 1 ± 1 | 0 ± 0 |
| Cataractispora appendiculata | 0.33 ± 0.33 | 20.67 ± 1.76 | 0 ± 0 | 0 ± 0 | 0 ± 0 | 0.67 ± 0.33 | 2.33 ± 1.86 | 0 ± 0 | 0 ± 0 | 0 ± 0 |
| Catenophlyctis variabilis | 0 ± 0 | 0 ± 0 | 0 ± 0 | 0 ± 0 | 0 ± 0 | 0.67 ± 0.67 | 0 ± 0 | 0 ± 0 | 0 ± 0 | 0.67 ± 0.67 |
| Celerioriella petrophiles | 1 ± 0.58 | 0 ± 0 | 0 ± 0 | 0 ± 0 | 0 ± 0 | 0 ± 0 | 0 ± 0 | 0 ± 0 | 0 ± 0 | 0 ± 0 |
| Cenangiopsis sp. KL376 | 0 ± 0 | 0 ± 0 | 0 ± 0 | 0 ± 0 | 0 ± 0 | 0.67 ± 0.67 | 0 ± 0 | 0 ± 0 | 0 ± 0 | 0 ± 0 |
| Cenococcum sp. | 0 ± 0 | 0 ± 0 | 0 ± 0 | 2.67 ± 1.76 | 0 ± 0 | 0 ± 0 | 1 ± 0.58 | 0.33 ± 0.33 | 1 ± 0.58 | 30 ± 22.59 |
| Cephaliophora tropica | 0 ± 0 | 0 ± 0 | 0 ± 0 | 0 ± 0 | 1.67 ± 1.67 | 0 ± 0 | 0 ± 0 | 0 ± 0 | 0 ± 0 | 0 ± 0 |
| Cephalosporium sp. BRO-2013 | 0 ± 0 | 0 ± 0 | 0 ± 0 | 0 ± 0 | 0 ± 0 | 0.67 ± 0.67 | 0 ± 0 | 0 ± 0 | 0 ± 0 | 0 ± 0 |
| Cephalotrichiella penicillata | 0 ± 0 | 0 ± 0 | 0 ± 0 | 0 ± 0 | 0 ± 0 | 0 ± 0 | 0 ± 0 | 0 ± 0 | 0 ± 0 | 0.33 ± 0.33 |
| Ceramothyrium linnaeae | 0 ± 0 | 8.67 ± 4.67 | 0.67 ± 0.67 | 1.33 ± 0.67 | 0 ± 0 | 1 ± 1 | 2.33 ± 2.33 | 22.67 ± 22.17 | 3.67 ± 3.67 | 15.33 ± 9.26 |
| Ceratobasidium cornigerum | 13.33 ± 3.84 | 0 ± 0 | 0 ± 0 | 0 ± 0 | 1 ± 0.58 | 0 ± 0 | 0 ± 0 | 0 ± 0 | 0 ± 0 | 0 ± 0 |
| Ceratobasidium sp. | 20.33 ± 5.61 | 0 ± 0 | 0.33 ± 0.33 | 7 ± 3.06 | 131 ± 67.99 | 2.33 ± 1.45 | 103.67 ± 84.76 | 3.33 ± 0.33 | 0.33 ± 0.33 | 12.33 ± 1.86 |
| Ceratobasidium sp. 4 JS-2017 | 4 ± 1.53 | 1.33 ± 1.33 | 0.33 ± 0.33 | 0.33 ± 0.33 | 82.33 ± 14.95 | 0.33 ± 0.33 | 4.67 ± 2.6 | 0 ± 0 | 0 ± 0 | 0 ± 0 |
| Ceratobasidium sp. AG-I | 0 ± 0 | 0 ± 0 | 0 ± 0 | 7.67 ± 6.23 | 0 ± 0 | 0.67 ± 0.33 | 5.33 ± 5.33 | 0 ± 0 | 0 ± 0 | 0 ± 0 |
| Ceratobasidium sp. AG-S | 0 ± 0 | 0 ± 0 | 0 ± 0 | 0 ± 0 | 0 ± 0 | 0 ± 0 | 0 ± 0 | 0.33 ± 0.33 | 0 ± 0 | 0 ± 0 |
| Ceratobasidium sp. CalS1-2 | 0 ± 0 | 0 ± 0 | 0 ± 0 | 0 ± 0 | 0 ± 0 | 0.67 ± 0.67 | 0 ± 0 | 0 ± 0 | 0 ± 0 | 0 ± 0 |
| Ceratobasidium sp. FN7 | 6 ± 3.79 | 0 ± 0 | 0 ± 0 | 0 ± 0 | 1 ± 1 | 0 ± 0 | 0 ± 0 | 0.33 ± 0.33 | 0.33 ± 0.33 | 0 ± 0 |
| Ceratobasidium sp. G3 | 0 ± 0 | 0 ± 0 | 0 ± 0 | 0 ± 0 | 0 ± 0 | 0 ± 0 | 0 ± 0 | 8.33 ± 8.33 | 0.67 ± 0.67 | 16.33 ± 10.53 |
| Cercophora acanthigera | 0 ± 0 | 0 ± 0 | 0 ± 0 | 0 ± 0 | 0 ± 0 | 0 ± 0 | 0 ± 0 | 0 ± 0 | 0 ± 0 | 6 ± 5 |
| Cercophora coprophila | 10.67 ± 4.63 | 2 ± 1.15 | 473.33 ± 434.34 | 19 ± 3.06 | 51.33 ± 6.89 | 126.67 ± 53.45 | 142.33 ± 49.75 | 64 ± 61 | 2.33 ± 1.2 | 11.67 ± 10.67 |
| Cercophora sp. CIM1_17 | 0 ± 0 | 0 ± 0 | 4.33 ± 4.33 | 1.67 ± 1.67 | 0 ± 0 | 0.33 ± 0.33 | 1 ± 1 | 0 ± 0 | 0 ± 0 | 0 ± 0 |
| Cercophora sp. TMS-2011 | 0 ± 0 | 0 ± 0 | 44.67 ± 11.2 | 1 ± 1 | 0.33 ± 0.33 | 7 ± 4.73 | 1.33 ± 0.67 | 10 ± 9.5 | 2.33 ± 2.33 | 0.33 ± 0.33 |
| Cercophora sparsa | 0.33 ± 0.33 | 1.33 ± 0.67 | 1 ± 0.58 | 53.67 ± 34.16 | 2.67 ± 1.2 | 4 ± 3.51 | 21.33 ± 14.89 | 0 ± 0 | 0 ± 0 | 0 ± 0 |
| Cercophora sulphurella | 0 ± 0 | 0 ± 0 | 3 ± 1.73 | 0 ± 0 | 0 ± 0 | 0 ± 0 | 0.33 ± 0.33 | 0 ± 0 | 0 ± 0 | 0 ± 0 |
| Cercophora thailandica | 0 ± 0 | 0 ± 0 | 8.67 ± 4.1 | 0 ± 0 | 0 ± 0 | 2 ± 1 | 0.33 ± 0.33 | 0 ± 0 | 0 ± 0 | 0.67 ± 0.67 |
| Cercophora vinosa | 0 ± 0 | 0 ± 0 | 0 ± 0 | 0 ± 0 | 0 ± 0 | 0 ± 0 | 0 ± 0 | 2.67 ± 2.67 | 0 ± 0 | 0 ± 0 |
| Cercospora asparagi | 0 ± 0 | 0 ± 0 | 0 ± 0 | 1 ± 0.58 | 0 ± 0 | 0 ± 0 | 0.67 ± 0.67 | 0 ± 0 | 0 ± 0 | 0 ± 0 |
| Cercospora dichondrae | 0 ± 0 | 0 ± 0 | 0 ± 0 | 0.67 ± 0.67 | 0 ± 0 | 0 ± 0 | 0.33 ± 0.33 | 1 ± 0.58 | 1 ± 1 | 0 ± 0 |
| Cercosporella dolichandrae | 0 ± 0 | 0 ± 0 | 0 ± 0 | 0 ± 0 | 0.67 ± 0.67 | 0 ± 0 | 0 ± 0 | 0 ± 0 | 0 ± 0 | 0 ± 0 |
| Chaetomella cf. raphigera G28 | 0 ± 0 | 0 ± 0 | 0 ± 0 | 0 ± 0 | 0 ± 0 | 0 ± 0 | 0 ± 0 | 0 ± 0 | 0 ± 0 | 0.67 ± 0.67 |
| Chaetomidium leptoderma | 0 ± 0 | 0 ± 0 | 0 ± 0 | 0 ± 0 | 0 ± 0 | 0 ± 0 | 0 ± 0 | 0 ± 0 | 0 ± 0 | 3.33 ± 3.33 |
| Chaetomium capillare | 0 ± 0 | 0 ± 0 | 5 ± 2.52 | 0 ± 0 | 0 ± 0 | 0.67 ± 0.67 | 0 ± 0 | 0 ± 0 | 0 ± 0 | 0 ± 0 |
| Chaetomium crispatum | 0 ± 0 | 1 ± 0.58 | 767.67 ± 426.57 | 11.33 ± 4.37 | 2.67 ± 0.67 | 180.33 ± 106.77 | 34.67 ± 10.4 | 1.67 ± 1.2 | 9.33 ± 9.33 | 3.33 ± 3.33 |
| Chaetomium homopilatum | 1.33 ± 0.33 | 42 ± 14.84 | 7.67 ± 6.67 | 2 ± 1 | 2 ± 1 | 6.67 ± 3.84 | 5.33 ± 2.96 | 13.33 ± 12.35 | 1 ± 1 | 3 ± 0.58 |
| Chaetomium mareoticum | 0 ± 0 | 0 ± 0 | 0 ± 0 | 0 ± 0 | 0 ± 0 | 0 ± 0 | 0 ± 0 | 0 ± 0 | 0 ± 0 | 0.67 ± 0.67 |
| Chaetomium perlucidum | 0 ± 0 | 0 ± 0 | 0 ± 0 | 0 ± 0 | 0 ± 0 | 0.67 ± 0.67 | 0 ± 0 | 0 ± 0 | 0 ± 0 | 0 ± 0 |
| Chaetomium seminudum | 0.67 ± 0.67 | 0.33 ± 0.33 | 0 ± 0 | 0.67 ± 0.67 | 0 ± 0 | 0 ± 0 | 1.67 ± 0.88 | 32.33 ± 14.95 | 0.67 ± 0.33 | 12.33 ± 10.84 |
| Chaetomium sp. | 0 ± 0 | 16 ± 4.51 | 3 ± 2.08 | 2.67 ± 1.45 | 1.67 ± 0.33 | 8.67 ± 7.67 | 32 ± 27.01 | 15 ± 6.24 | 1.33 ± 0.33 | 25 ± 6.56 |
| Chaetomium sp. 1 RJ2014 | 0 ± 0 | 0 ± 0 | 0 ± 0 | 0 ± 0 | 0 ± 0 | 25 ± 11.02 | 1 ± 1 | 0 ± 0 | 0 ± 0 | 0 ± 0 |
| Chaetomium sp. 2 RJ2014 | 0 ± 0 | 0 ± 0 | 0 ± 0 | 0 ± 0 | 0 ± 0 | 0 ± 0 | 2.33 ± 2.33 | 0 ± 0 | 0 ± 0 | 0 ± 0 |
| Chaetomium sp. F26 | 0 ± 0 | 0 ± 0 | 0 ± 0 | 0 ± 0 | 0 ± 0 | 0 ± 0 | 0.67 ± 0.67 | 0 ± 0 | 0 ± 0 | 0 ± 0 |
| Chaetomium sp. SS5 | 0 ± 0 | 0 ± 0 | 6.33 ± 2.96 | 0 ± 0 | 0 ± 0 | 3.33 ± 1.2 | 8.33 ± 6.89 | 0.33 ± 0.33 | 0 ± 0 | 0 ± 0 |
| Chaetomium subspirale | 1.33 ± 0.88 | 55.33 ± 54.34 | 1.67 ± 1.67 | 1.67 ± 0.88 | 0.33 ± 0.33 | 1.67 ± 0.88 | 9.33 ± 5.24 | 1 ± 0 | 0.33 ± 0.33 | 0 ± 0 |
| Chaetomium subspirilliferum | 0 ± 0 | 0 ± 0 | 0 ± 0 | 0 ± 0 | 0 ± 0 | 2 ± 2 | 0 ± 0 | 0 ± 0 | 0 ± 0 | 1.67 ± 1.67 |
| Chaetomium succineum | 0 ± 0 | 2.67 ± 2.67 | 0 ± 0 | 0 ± 0 | 0 ± 0 | 0.33 ± 0.33 | 0 ± 0 | 3.67 ± 3.67 | 0 ± 0 | 5 ± 2.65 |
| Chaetospermum chaetosporum | 0 ± 0 | 0 ± 0 | 0 ± 0 | 0 ± 0 | 0 ± 0 | 0 ± 0 | 0 ± 0 | 0 ± 0 | 0.67 ± 0.67 | 0 ± 0 |
| Chaetosphaeria fusiformis | 0 ± 0 | 0 ± 0 | 0 ± 0 | 0 ± 0 | 0 ± 0 | 0 ± 0 | 0 ± 0 | 0 ± 0 | 0 ± 0 | 1 ± 1 |
| Chaetosphaeria myriocarpa | 0 ± 0 | 0 ± 0 | 1.33 ± 1.33 | 0 ± 0 | 3.67 ± 3.67 | 2.67 ± 2.67 | 0 ± 0 | 0 ± 0 | 0 ± 0 | 0.67 ± 0.67 |
| Chaetosphaeria sp. KRCF730 | 0 ± 0 | 0 ± 0 | 1 ± 1 | 0 ± 0 | 0 ± 0 | 1.67 ± 1.67 | 0 ± 0 | 0 ± 0 | 0 ± 0 | 0 ± 0 |
| Chaetosphaeronema sp. | 0 ± 0 | 3.33 ± 2.4 | 0 ± 0 | 0 ± 0 | 0.33 ± 0.33 | 0 ± 0 | 0 ± 0 | 0 ± 0 | 0 ± 0 | 0 ± 0 |
| Chalara hyalocuspica | 0 ± 0 | 0 ± 0 | 0 ± 0 | 0 ± 0 | 0 ± 0 | 0 ± 0 | 0.33 ± 0.33 | 3.33 ± 1.76 | 1 ± 1 | 175.33 ± 163.84 |
| Chalara sp. | 2.33 ± 1.86 | 7 ± 5.13 | 0 ± 0 | 0.33 ± 0.33 | 2 ± 1.15 | 1.33 ± 1.33 | 16 ± 13.53 | 16.33 ± 11.29 | 7.33 ± 7.33 | 0 ± 0 |
| Chalara sp. NK388 | 0 ± 0 | 0 ± 0 | 1.33 ± 0.88 | 0 ± 0 | 0 ± 0 | 0 ± 0 | 0 ± 0 | 0 ± 0 | 0 ± 0 | 0 ± 0 |
| Chalara sp. TMS-2011 | 0 ± 0 | 0.33 ± 0.33 | 88.33 ± 29.33 | 2 ± 1.15 | 0.33 ± 0.33 | 25 ± 14.53 | 4.33 ± 2.33 | 0.33 ± 0.33 | 0.67 ± 0.33 | 2.33 ± 2.33 |
| Chalara sp. YTH-2012b | 0 ± 0 | 2.33 ± 1.86 | 0 ± 0 | 0.33 ± 0.33 | 0 ± 0 | 0 ± 0 | 1 ± 1 | 0 ± 0 | 0 ± 0 | 3.33 ± 1.45 |
| Cheilymenia pulcherrima | 10.33 ± 3.48 | 0 ± 0 | 0 ± 0 | 0 ± 0 | 0.67 ± 0.33 | 0 ± 0 | 0 ± 0 | 0 ± 0 | 0 ± 0 | 0 ± 0 |
| Cheilymenia stercoraria | 0 ± 0 | 0 ± 0 | 0 ± 0 | 0 ± 0 | 5 ± 4.04 | 0 ± 0 | 0.33 ± 0.33 | 0 ± 0 | 0 ± 0 | 0 ± 0 |
| Cheirosporium triseriale | 0 ± 0 | 0 ± 0 | 0 ± 0 | 0 ± 0 | 0 ± 0 | 0 ± 0 | 4.33 ± 4.33 | 0 ± 0 | 0 ± 0 | 1.33 ± 0.88 |
| Chlamydocillium cyanophilum | 0 ± 0 | 0 ± 0 | 0 ± 0 | 0 ± 0 | 0 ± 0 | 0.67 ± 0.67 | 0 ± 0 | 0 ± 0 | 0 ± 0 | 0 ± 0 |
| Chloridium botryoideum | 0.67 ± 0.33 | 0 ± 0 | 7.67 ± 2.33 | 0 ± 0 | 4.67 ± 0.67 | 0.33 ± 0.33 | 0 ± 0 | 0 ± 0 | 0 ± 0 | 0 ± 0 |
| Chloridium sp. | 0 ± 0 | 4 ± 2.08 | 165 ± 71.84 | 11 ± 5.69 | 1.67 ± 1.2 | 108.67 ± 28.59 | 11.67 ± 7.26 | 0.67 ± 0.67 | 1.33 ± 1.33 | 6 ± 1.53 |
| Chloridium sp. KO-2013 | 0 ± 0 | 0 ± 0 | 0.33 ± 0.33 | 0.33 ± 0.33 | 0 ± 0 | 0.33 ± 0.33 | 0.33 ± 0.33 | 0 ± 0 | 0 ± 0 | 0 ± 0 |
| Chloridium sp. TMS-2011 | 0 ± 0 | 0 ± 0 | 0 ± 0 | 0.33 ± 0.33 | 0 ± 0 | 0.33 ± 0.33 | 0 ± 0 | 0 ± 0 | 0 ± 0 | 0 ± 0 |
| Chloridium virescens | 0 ± 0 | 0.33 ± 0.33 | 1 ± 1 | 1.33 ± 1.33 | 0 ± 0 | 0 ± 0 | 0.33 ± 0.33 | 1.67 ± 0.33 | 1 ± 1 | 0.67 ± 0.33 |
| Chrysosporium articulatum | 0 ± 0 | 0 ± 0 | 0 ± 0 | 0 ± 0 | 0 ± 0 | 0 ± 0 | 0 ± 0 | 0 ± 0 | 0.33 ± 0.33 | 0 ± 0 |
| Chrysosporium keratinophilum | 0 ± 0 | 0 ± 0 | 0 ± 0 | 0 ± 0 | 0 ± 0 | 0.67 ± 0.67 | 0 ± 0 | 0 ± 0 | 0 ± 0 | 0 ± 0 |
| Chrysosporium pannicola | 0 ± 0 | 0 ± 0 | 0 ± 0 | 0 ± 0 | 0 ± 0 | 0 ± 0 | 1.33 ± 1.33 | 0 ± 0 | 0 ± 0 | 0 ± 0 |
| Chrysosporium sp. | 0 ± 0 | 0 ± 0 | 0 ± 0 | 0 ± 0 | 0.67 ± 0.33 | 0 ± 0 | 0 ± 0 | 0 ± 0 | 0 ± 0 | 0 ± 0 |
| Chrysosporium sp. 14PA12 | 0 ± 0 | 0 ± 0 | 0 ± 0 | 0 ± 0 | 0 ± 0 | 0 ± 0 | 2.67 ± 2.67 | 0 ± 0 | 0 ± 0 | 1.67 ± 1.67 |
| Chrysosporium sp. OUCMBIII101004 | 0 ± 0 | 0 ± 0 | 0 ± 0 | 0 ± 0 | 0 ± 0 | 0 ± 0 | 1.33 ± 1.33 | 0 ± 0 | 0 ± 0 | 0 ± 0 |
| Chytriomyces confervae | 0 ± 0 | 0 ± 0 | 0.67 ± 0.67 | 0 ± 0 | 0 ± 0 | 0 ± 0 | 0 ± 0 | 0 ± 0 | 0 ± 0 | 0 ± 0 |
| Ciboria aestivalis | 0 ± 0 | 0 ± 0 | 0 ± 0 | 0.33 ± 0.33 | 0.33 ± 0.33 | 0.33 ± 0.33 | 0.67 ± 0.67 | 0 ± 0 | 0 ± 0 | 0 ± 0 |
| Ciboria shiraiana | 0 ± 0 | 0 ± 0 | 0 ± 0 | 0 ± 0 | 0 ± 0 | 0 ± 0 | 1.33 ± 1.33 | 0 ± 0 | 0 ± 0 | 0 ± 0 |
| Ciliolarina ligniseda | 0 ± 0 | 0 ± 0 | 26.67 ± 10.84 | 0.33 ± 0.33 | 0 ± 0 | 3.67 ± 1.86 | 1 ± 0.58 | 0 ± 0 | 0 ± 0 | 0 ± 0 |
| Ciliolarina pinicola | 0 ± 0 | 0 ± 0 | 11 ± 6.66 | 1.33 ± 0.67 | 0 ± 0 | 3.67 ± 3.18 | 0.67 ± 0.67 | 0 ± 0 | 0.33 ± 0.33 | 0 ± 0 |
| Circinotrichum cycadis | 0 ± 0 | 0 ± 0 | 0.33 ± 0.33 | 0 ± 0 | 0.67 ± 0.33 | 0 ± 0 | 0.33 ± 0.33 | 33 ± 32.5 | 2.67 ± 1.45 | 0 ± 0 |
| Circinotrichum maculiforme | 0 ± 0 | 0 ± 0 | 0 ± 0 | 0 ± 0 | 0 ± 0 | 0 ± 0 | 0 ± 0 | 0 ± 0 | 0 ± 0 | 0 ± 0 |
| Circinotrichum sinense | 0 ± 0 | 0 ± 0 | 0 ± 0 | 0 ± 0 | 0 ± 0 | 0 ± 0 | 0 ± 0 | 0 ± 0 | 0.67 ± 0.67 | 0 ± 0 |
| Cladobotryum apiculatum | 0 ± 0 | 0 ± 0 | 0 ± 0 | 0 ± 0 | 0 ± 0 | 0 ± 0 | 1.67 ± 1.67 | 0 ± 0 | 0 ± 0 | 0 ± 0 |
| Cladobotryum protrusum | 0 ± 0 | 0 ± 0 | 2.67 ± 2.67 | 0 ± 0 | 0.67 ± 0.33 | 4 ± 2.08 | 2.67 ± 2.67 | 0 ± 0 | 0.33 ± 0.33 | 2.67 ± 2.19 |
| Cladophialophora chaetospira | 1.67 ± 0.88 | 40.33 ± 16.68 | 134.67 ± 80.2 | 8.33 ± 3.84 | 0.33 ± 0.33 | 20 ± 10.26 | 24 ± 7.51 | 29.33 ± 19.4 | 6.33 ± 5.36 | 6 ± 4.58 |
| Cladophialophora minutissima | 0 ± 0 | 9 ± 8.02 | 0 ± 0 | 2 ± 2 | 0 ± 0 | 0 ± 0 | 1 ± 1 | 7.67 ± 5.78 | 4.67 ± 1.45 | 25.33 ± 18.77 |
| Cladophialophora sp. | 0 ± 0 | 12.67 ± 7.22 | 0 ± 0 | 0.67 ± 0.67 | 0 ± 0 | 0.33 ± 0.33 | 5.33 ± 2.73 | 0 ± 0 | 0 ± 0 | 1.67 ± 1.67 |
| Cladophialophora sp. K101008-4-7 | 0 ± 0 | 0.67 ± 0.67 | 9.33 ± 2.73 | 0 ± 0 | 0 ± 0 | 3.67 ± 3.67 | 1 ± 1 | 0 ± 0 | 0 ± 0 | 0 ± 0 |
| Cladophialophora sp. KO-groupE 2014 | 7.33 ± 2.4 | 449.33 ± 174.82 | 371.33 ± 63.65 | 136.67 ± 35.38 | 0.67 ± 0.33 | 117.33 ± 95.11 | 225.33 ± 47.81 | 180.33 ± 40.14 | 158 ± 32.52 | 164.33 ± 67.46 |
| Cladophialophora sp. KO-groupF 2014 | 0 ± 0 | 0 ± 0 | 0 ± 0 | 0 ± 0 | 0 ± 0 | 0.67 ± 0.67 | 0 ± 0 | 0 ± 0 | 0 ± 0 | 0 ± 0 |
| Cladophialophora sp. KO-groupH 2014 | 4.33 ± 1.45 | 256.67 ± 109.11 | 1.67 ± 0.88 | 14.67 ± 6.23 | 0.67 ± 0.67 | 3.33 ± 2.4 | 27.33 ± 18.27 | 5 ± 5 | 21.67 ± 14.05 | 2 ± 1.53 |
| Cladophialophora sp. KO-groupM 2014 | 0 ± 0 | 0 ± 0 | 0 ± 0 | 0 ± 0 | 0 ± 0 | 0 ± 0 | 0 ± 0 | 0 ± 0 | 0 ± 0 | 1 ± 1 |
| Cladophialophora sp. KO-groupP 2014 | 0 ± 0 | 2.33 ± 2.33 | 0 ± 0 | 0 ± 0 | 0 ± 0 | 0 ± 0 | 0 ± 0 | 0 ± 0 | 0 ± 0 | 0 ± 0 |
| Cladophialophora sp. L3-3-NN-2016 | 0 ± 0 | 0 ± 0 | 0 ± 0 | 0 ± 0 | 0 ± 0 | 0 ± 0 | 0 ± 0 | 0 ± 0 | 0.33 ± 0.33 | 0.33 ± 0.33 |
| Cladophialophora sp. TS-2016 | 0 ± 0 | 0 ± 0 | 7.67 ± 3.33 | 0 ± 0 | 0 ± 0 | 1.67 ± 0.88 | 0.33 ± 0.33 | 1 ± 1 | 2.33 ± 2.33 | 3 ± 3 |
| Cladorrhinum australe | 0 ± 0 | 0.33 ± 0.33 | 0 ± 0 | 0 ± 0 | 0 ± 0 | 0 ± 0 | 0 ± 0 | 0 ± 0 | 0 ± 0 | 0 ± 0 |
| Cladorrhinum brunnescens | 0.33 ± 0.33 | 1 ± 0 | 4.33 ± 2.4 | 4.33 ± 3.84 | 2.33 ± 1.33 | 24 ± 17.06 | 16.33 ± 10.9 | 30.67 ± 29.67 | 0 ± 0 | 0.33 ± 0.33 |
| Cladorrhinum bulbillosum | 0 ± 0 | 0 ± 0 | 0 ± 0 | 0 ± 0 | 0 ± 0 | 0 ± 0 | 0 ± 0 | 0.67 ± 0.67 | 0 ± 0 | 0 ± 0 |
| Cladorrhinum flexuosum | 0 ± 0 | 0 ± 0 | 0 ± 0 | 0 ± 0 | 2.33 ± 2.33 | 0 ± 0 | 0 ± 0 | 1.33 ± 1.33 | 0 ± 0 | 0 ± 0 |
| Cladorrhinum samala | 0 ± 0 | 0 ± 0 | 0 ± 0 | 0 ± 0 | 0 ± 0 | 0.33 ± 0.33 | 0 ± 0 | 0 ± 0 | 0 ± 0 | 0.67 ± 0.67 |
| Cladosporium dominicanum | 0 ± 0 | 0 ± 0 | 0 ± 0 | 0 ± 0 | 0 ± 0 | 0.33 ± 0.33 | 0.67 ± 0.67 | 0 ± 0 | 0 ± 0 | 1.33 ± 1.33 |
| Cladosporium nigrellum | 0 ± 0 | 0 ± 0 | 0 ± 0 | 0 ± 0 | 0 ± 0 | 0 ± 0 | 0 ± 0 | 0 ± 0 | 0 ± 0 | 0 ± 0 |
| Cladosporium perangustum | 7 ± 4.51 | 6.67 ± 3.53 | 58.33 ± 40.35 | 7.67 ± 2.85 | 8.67 ± 1.2 | 36.67 ± 10.53 | 34.33 ± 16.97 | 59 ± 27.68 | 22.33 ± 17.42 | 48.33 ± 26.81 |
| Cladosporium sinense | 0.33 ± 0.33 | 0.67 ± 0.33 | 1.33 ± 1.33 | 1.33 ± 1.33 | 0.67 ± 0.33 | 4.67 ± 2.67 | 4.33 ± 4.33 | 0.33 ± 0.33 | 0 ± 0 | 0 ± 0 |
| Cladosporium sp. | 72.33 ± 54.35 | 107.33 ± 64.2 | 437.67 ± 254.88 | 47 ± 18.03 | 24.67 ± 6.39 | 188.33 ± 58.84 | 180.67 ± 58.6 | 128.33 ± 63.73 | 55.67 ± 45.32 | 184.33 ± 69.5 |
| Cladosporium sp. FA-A2 | 1.33 ± 1.33 | 0 ± 0 | 0 ± 0 | 0 ± 0 | 0 ± 0 | 0 ± 0 | 0 ± 0 | 0 ± 0 | 0 ± 0 | 0 ± 0 |
| Claroideoglomus cf. drummondii | 0 ± 0 | 0 ± 0 | 0 ± 0 | 0 ± 0 | 0 ± 0 | 0.33 ± 0.33 | 0 ± 0 | 0 ± 0 | 0 ± 0 | 0 ± 0 |
| Claroideoglomus claroideum | 0 ± 0 | 0 ± 0 | 0.33 ± 0.33 | 0 ± 0 | 3 ± 1.15 | 0 ± 0 | 1 ± 0.58 | 0.67 ± 0.33 | 1 ± 0.58 | 0 ± 0 |
| Claroideoglomus hanlinii | 0 ± 0 | 0.67 ± 0.67 | 0 ± 0 | 0 ± 0 | 0 ± 0 | 0 ± 0 | 0 ± 0 | 0 ± 0 | 0 ± 0 | 0 ± 0 |
| Claroideoglomus sp. 1 RS-2017 | 0 ± 0 | 0.67 ± 0.67 | 0 ± 0 | 0 ± 0 | 0 ± 0 | 0 ± 0 | 0 ± 0 | 0.67 ± 0.67 | 0 ± 0 | 0 ± 0 |
| Classicula sinensis | 0 ± 0 | 0 ± 0 | 0 ± 0 | 0.67 ± 0.67 | 2 ± 1.53 | 0 ± 0 | 1 ± 1 | 0.33 ± 0.33 | 0 ± 0 | 0 ± 0 |
| Claussenomyces atrovirens | 0 ± 0 | 0 ± 0 | 0 ± 0 | 0 ± 0 | 0 ± 0 | 0 ± 0 | 0 ± 0 | 0 ± 0 | 0 ± 0 | 0.67 ± 0.67 |
| Claussenomyces cf. hydnicola H.B. 9975 | 0 ± 0 | 0 ± 0 | 0 ± 0 | 0 ± 0 | 0 ± 0 | 1.33 ± 1.33 | 0 ± 0 | 0 ± 0 | 0 ± 0 | 1.33 ± 1.33 |
| Claussenomyces sp. | 0 ± 0 | 0.33 ± 0.33 | 8 ± 7.02 | 1.67 ± 0.33 | 0 ± 0 | 2.33 ± 2.33 | 0.33 ± 0.33 | 0.67 ± 0.67 | 0 ± 0 | 0.67 ± 0.67 |
| Claussenomyces sp. HB9300b | 0.33 ± 0.33 | 67.33 ± 43.95 | 0 ± 0 | 1.67 ± 1.2 | 0 ± 0 | 0 ± 0 | 11 ± 7.77 | 0.67 ± 0.33 | 3 ± 3 | 54.33 ± 53.83 |
| Claussenomyces sp. PDD 55517 | 0 ± 0 | 1 ± 0.58 | 0.33 ± 0.33 | 3.33 ± 3.33 | 0 ± 0 | 0 ± 0 | 2 ± 1.53 | 0.33 ± 0.33 | 0.33 ± 0.33 | 0.67 ± 0.67 |
| Clavaria asterospora | 733.33 ± 168.33 | 5.33 ± 4.84 | 0.33 ± 0.33 | 1 ± 1 | 33.67 ± 5.36 | 2 ± 1.53 | 3.67 ± 1.2 | 496.67 ± 493.67 | 50.67 ± 37.32 | 1.67 ± 0.88 |
| Clavaria citrinorubra | 0 ± 0 | 0 ± 0 | 0.67 ± 0.67 | 5.33 ± 4.37 | 0 ± 0 | 0 ± 0 | 0.33 ± 0.33 | 0 ± 0 | 0 ± 0 | 0 ± 0 |
| Clavaria falcata | 4.33 ± 2.19 | 0 ± 0 | 0 ± 0 | 0 ± 0 | 0 ± 0 | 2 ± 1.53 | 10.67 ± 10.67 | 0 ± 0 | 0 ± 0 | 2.33 ± 1.2 |
| Clavaria flavipes | 0 ± 0 | 1.33 ± 0.33 | 0 ± 0 | 0 ± 0 | 0 ± 0 | 0.33 ± 0.33 | 0.67 ± 0.67 | 0 ± 0 | 0 ± 0 | 1 ± 1 |
| Clavaria fragilis | 0 ± 0 | 0 ± 0 | 0 ± 0 | 0 ± 0 | 0 ± 0 | 0 ± 0 | 0 ± 0 | 1 ± 1 | 4.33 ± 2.19 | 0 ± 0 |
| Clavaria globospora | 0.33 ± 0.33 | 0 ± 0 | 0 ± 0 | 0 ± 0 | 1.33 ± 0.67 | 0 ± 0 | 0 ± 0 | 0 ± 0 | 0 ± 0 | 0 ± 0 |
| Clavaria greletii | 0 ± 0 | 0 ± 0 | 0 ± 0 | 0 ± 0 | 0 ± 0 | 0 ± 0 | 0 ± 0 | 0 ± 0 | 0 ± 0 | 0.67 ± 0.67 |
| Clavaria greletoides | 0 ± 0 | 24 ± 15.31 | 0 ± 0 | 0 ± 0 | 0 ± 0 | 0 ± 0 | 0 ± 0 | 0 ± 0 | 0 ± 0 | 0 ± 0 |
| Clavaria sp. SAV1992 (BRA) | 0 ± 0 | 0 ± 0 | 0 ± 0 | 0.33 ± 0.33 | 0 ± 0 | 0 ± 0 | 1.67 ± 1.2 | 4 ± 4 | 11 ± 6.08 | 0 ± 0 |
| Clavaria tenuipes | 25.67 ± 2.73 | 0 ± 0 | 0 ± 0 | 0 ± 0 | 1.33 ± 0.33 | 0 ± 0 | 0 ± 0 | 4.33 ± 4.33 | 2.67 ± 1.33 | 0 ± 0 |
| Clavaria ypsilondia | 8.67 ± 2.03 | 0 ± 0 | 0 ± 0 | 0 ± 0 | 1 ± 0.58 | 0 ± 0 | 0 ± 0 | 0 ± 0 | 0 ± 0 | 0 ± 0 |
| Clavariopsis aquatica | 0 ± 0 | 0 ± 0 | 0 ± 0 | 0 ± 0 | 0 ± 0 | 0 ± 0 | 0 ± 0 | 0 ± 0 | 0 ± 0 | 1.33 ± 1.33 |
| Claviceps panicoidearum | 1.67 ± 1.2 | 0 ± 0 | 0 ± 0 | 0 ± 0 | 0 ± 0 | 0 ± 0 | 0 ± 0 | 0 ± 0 | 0 ± 0 | 0 ± 0 |
| Claviceps paspali | 0 ± 0 | 0 ± 0 | 0 ± 0 | 0 ± 0 | 0 ± 0 | 0 ± 0 | 0 ± 0 | 0 ± 0 | 0 ± 0 | 1.67 ± 1.67 |
| Clavicorona taxophila | 101 ± 17.06 | 1 ± 0.58 | 13 ± 5.29 | 27.67 ± 13.93 | 11.67 ± 4.18 | 5.67 ± 2.85 | 37.33 ± 22.88 | 0.33 ± 0.33 | 0.67 ± 0.67 | 1 ± 1 |
| Clavulina sp. | 0 ± 0 | 6.67 ± 1.76 | 4 ± 1.73 | 230.67 ± 98.38 | 0.33 ± 0.33 | 2.67 ± 2.19 | 99.33 ± 51.56 | 0 ± 0 | 0 ± 0 | 0 ± 0 |
| Clavulina sp. OSC 1064248 | 2 ± 1 | 0 ± 0 | 0 ± 0 | 0 ± 0 | 0 ± 0 | 0 ± 0 | 0 ± 0 | 0 ± 0 | 0 ± 0 | 0 ± 0 |
| Clitocybe sp. 4 PA-2015 | 0 ± 0 | 0 ± 0 | 0 ± 0 | 0 ± 0 | 0 ± 0 | 0 ± 0 | 0 ± 0 | 0 ± 0 | 0 ± 0 | 1 ± 1 |
| Clitocybe trulliformis | 0 ± 0 | 0 ± 0 | 6.67 ± 6.67 | 0 ± 0 | 0 ± 0 | 0 ± 0 | 0.33 ± 0.33 | 0 ± 0 | 0 ± 0 | 0 ± 0 |
| Clitopilus kamaka | 0.33 ± 0.33 | 1.67 ± 0.67 | 16.67 ± 5.7 | 26.67 ± 26.17 | 0 ± 0 | 2.33 ± 2.33 | 11.33 ± 6.84 | 0.67 ± 0.67 | 2 ± 0 | 202 ± 81.96 |
| Clitopilus prunulus | 0.67 ± 0.33 | 171.67 ± 68.86 | 32 ± 28.51 | 19 ± 8.39 | 1 ± 1 | 5 ± 2.89 | 21 ± 10.6 | 25.67 ± 8.41 | 42.33 ± 4.48 | 49 ± 28.73 |
| Clitopilus sp. K6 | 0 ± 0 | 0 ± 0 | 0 ± 0 | 0 ± 0 | 0 ± 0 | 0 ± 0 | 0 ± 0 | 0 ± 0 | 0 ± 0 | 0.67 ± 0.67 |
| Clonostachys rogersoniana | 16 ± 1.53 | 0 ± 0 | 0 ± 0 | 0 ± 0 | 1 ± 0 | 0 ± 0 | 1 ± 1 | 0.67 ± 0.33 | 0 ± 0 | 4 ± 2.31 |
| Clonostachys rosea | 5.67 ± 2.19 | 16.33 ± 6.77 | 39.33 ± 22.4 | 2.67 ± 1.76 | 0.33 ± 0.33 | 18 ± 2.89 | 11.67 ± 2.73 | 22.67 ± 10.4 | 8.33 ± 7.84 | 2.67 ± 0.33 |
| Clonostachys sp. | 12.33 ± 3.38 | 158 ± 64.16 | 105.67 ± 74.18 | 12 ± 5.29 | 7.33 ± 3.18 | 35 ± 8.62 | 30.67 ± 13.2 | 41.33 ± 19.67 | 16 ± 14.5 | 4.33 ± 0.88 |
| Clonostachys sp. ATT061 | 0 ± 0 | 0 ± 0 | 0 ± 0 | 0 ± 0 | 0 ± 0 | 0 ± 0 | 0.33 ± 0.33 | 8 ± 6.11 | 6 ± 6 | 0 ± 0 |
| Clonostachys sp. CWG1(1) | 1.67 ± 1.67 | 0.33 ± 0.33 | 0 ± 0 | 4 ± 3.51 | 0 ± 0 | 0.33 ± 0.33 | 3.67 ± 3.18 | 105.67 ± 56.97 | 30 ± 29.5 | 0 ± 0 |
| Clonostachys sp. MIAE01299 | 0 ± 0 | 0 ± 0 | 0 ± 0 | 0 ± 0 | 0 ± 0 | 0.67 ± 0.33 | 0 ± 0 | 0 ± 0 | 0 ± 0 | 0.33 ± 0.33 |
| Clypeosphaeria mamillana | 0 ± 0 | 0 ± 0 | 0 ± 0 | 0 ± 0 | 0 ± 0 | 0 ± 0 | 0 ± 0 | 1 ± 1 | 0 ± 0 | 0 ± 0 |
| Codinaea acaciae | 0.33 ± 0.33 | 43.67 ± 7.06 | 0 ± 0 | 4.33 ± 3.84 | 0 ± 0 | 0 ± 0 | 4.33 ± 2.33 | 2.33 ± 1.86 | 2.33 ± 2.33 | 0 ± 0 |
| Colacogloea falcata | 0 ± 0 | 0 ± 0 | 0 ± 0 | 0 ± 0 | 0 ± 0 | 0 ± 0 | 0 ± 0 | 1.33 ± 1.33 | 0 ± 0 | 0 ± 0 |
| Colacogloea retinophila | 0 ± 0 | 0 ± 0 | 0 ± 0 | 0 ± 0 | 0 ± 0 | 0 ± 0 | 0 ± 0 | 0 ± 0 | 0 ± 0 | 1.33 ± 1.33 |
| Coleophoma ericicola | 0 ± 0 | 0 ± 0 | 0 ± 0 | 0 ± 0 | 0 ± 0 | 0 ± 0 | 0 ± 0 | 1 ± 1 | 0 ± 0 | 0 ± 0 |
| Collariella gracilis | 0 ± 0 | 0 ± 0 | 0 ± 0 | 0 ± 0 | 0 ± 0 | 0 ± 0 | 0 ± 0 | 0 ± 0 | 0 ± 0 | 0 ± 0 |
| Collarina aurantiaca | 0 ± 0 | 0 ± 0 | 7.33 ± 4.37 | 0.67 ± 0.67 | 0.33 ± 0.33 | 1 ± 1 | 9 ± 8.5 | 1 ± 0.58 | 2.33 ± 1.45 | 1.33 ± 0.33 |
| Colletotrichum acutatum | 0 ± 0 | 0 ± 0 | 0 ± 0 | 0.33 ± 0.33 | 0 ± 0 | 4 ± 4 | 3.33 ± 2.4 | 0.33 ± 0.33 | 0 ± 0 | 0.33 ± 0.33 |
| Colletotrichum boninense | 0 ± 0 | 0.33 ± 0.33 | 8 ± 2.52 | 0.33 ± 0.33 | 0 ± 0 | 1.67 ± 1.67 | 0 ± 0 | 0 ± 0 | 0 ± 0 | 0 ± 0 |
| Colletotrichum cliviicola | 0 ± 0 | 0 ± 0 | 0.67 ± 0.67 | 0 ± 0 | 0 ± 0 | 0 ± 0 | 0 ± 0 | 0 ± 0 | 0 ± 0 | 0 ± 0 |
| Colletotrichum coccodes | 0 ± 0 | 0 ± 0 | 0 ± 0 | 0 ± 0 | 0 ± 0 | 1 ± 1 | 0 ± 0 | 0 ± 0 | 0 ± 0 | 0 ± 0 |
| Colletotrichum gloeosporioides | 0 ± 0 | 0 ± 0 | 0 ± 0 | 6.33 ± 2.4 | 0.33 ± 0.33 | 10.33 ± 9.35 | 2.33 ± 1.45 | 2.33 ± 2.33 | 5 ± 2.65 | 5 ± 2.52 |
| Colletotrichum godetiae | 0 ± 0 | 0 ± 0 | 0 ± 0 | 0 ± 0 | 0 ± 0 | 0 ± 0 | 3 ± 3 | 0 ± 0 | 0.33 ± 0.33 | 0.33 ± 0.33 |
| Colletotrichum higginsianum | 0 ± 0 | 0.67 ± 0.67 | 0 ± 0 | 0 ± 0 | 0 ± 0 | 0 ± 0 | 0 ± 0 | 0 ± 0 | 0 ± 0 | 1.67 ± 1.67 |
| Colletotrichum pisi | 0 ± 0 | 0 ± 0 | 0 ± 0 | 0 ± 0 | 0 ± 0 | 1.33 ± 0.67 | 0 ± 0 | 0 ± 0 | 0 ± 0 | 0 ± 0 |
| Colletotrichum queenslandicum | 0 ± 0 | 0 ± 0 | 2 ± 2 | 0 ± 0 | 0 ± 0 | 0 ± 0 | 0 ± 0 | 0 ± 0 | 0 ± 0 | 0 ± 0 |
| Colletotrichum sp. CP-CC026 | 0 ± 0 | 0 ± 0 | 0 ± 0 | 0 ± 0 | 0 ± 0 | 0 ± 0 | 0 ± 0 | 0 ± 0 | 0 ± 0 | 3.67 ± 3.18 |
| Colletotrichum tofieldiae | 0 ± 0 | 0 ± 0 | 0.33 ± 0.33 | 0 ± 0 | 0 ± 0 | 50.67 ± 23.84 | 1 ± 0.58 | 0 ± 0 | 0.33 ± 0.33 | 9 ± 3.51 |
| Colletotrichum truncatum | 0 ± 0 | 7.33 ± 0.67 | 0 ± 0 | 0 ± 0 | 0 ± 0 | 1 ± 1 | 1.33 ± 0.67 | 0 ± 0 | 0 ± 0 | 0 ± 0 |
| Colletotrichum xanthorrhoeae | 0 ± 0 | 0 ± 0 | 0 ± 0 | 6 ± 1.53 | 0 ± 0 | 0 ± 0 | 0.67 ± 0.67 | 0 ± 0 | 0 ± 0 | 1 ± 1 |
| Collophorina paarla | 0 ± 0 | 0 ± 0 | 0 ± 0 | 4.33 ± 2.33 | 0 ± 0 | 0 ± 0 | 0.33 ± 0.33 | 0.33 ± 0.33 | 0 ± 0 | 1.33 ± 1.33 |
| Confertobasidium sp. UC2022936 | 0 ± 0 | 0 ± 0 | 0 ± 0 | 0 ± 0 | 0 ± 0 | 0.67 ± 0.67 | 0 ± 0 | 0 ± 0 | 0 ± 0 | 0 ± 0 |
| Coniella diplodiopsis | 0 ± 0 | 0 ± 0 | 0 ± 0 | 0 ± 0 | 0 ± 0 | 0 ± 0 | 0 ± 0 | 1.33 ± 1.33 | 0 ± 0 | 0 ± 0 |
| Coniochaeta gigantospora | 0 ± 0 | 0 ± 0 | 0 ± 0 | 0 ± 0 | 0 ± 0 | 0 ± 0 | 0 ± 0 | 0 ± 0 | 0 ± 0 | 0.67 ± 0.67 |
| Coniochaeta lignicola | 0 ± 0 | 2 ± 1 | 0 ± 0 | 0.67 ± 0.67 | 0 ± 0 | 0 ± 0 | 0.67 ± 0.67 | 0 ± 0 | 0 ± 0 | 0 ± 0 |
| Coniochaeta rosae | 0 ± 0 | 21.33 ± 8.69 | 0 ± 0 | 0.33 ± 0.33 | 0.33 ± 0.33 | 1 ± 1 | 1 ± 0.58 | 0 ± 0 | 0 ± 0 | 0 ± 0 |
| Coniochaeta sp. | 0 ± 0 | 0 ± 0 | 1 ± 0.58 | 0 ± 0 | 0.33 ± 0.33 | 21.33 ± 12.72 | 0 ± 0 | 0 ± 0 | 0 ± 0 | 0 ± 0 |
| Coniochaeta sp. I179 | 0 ± 0 | 0.33 ± 0.33 | 2.33 ± 1.86 | 0 ± 0 | 0.33 ± 0.33 | 4.67 ± 1.86 | 0.33 ± 0.33 | 1.67 ± 1.67 | 0 ± 0 | 2 ± 1.15 |
| Coniochaeta sp. I95 | 0 ± 0 | 0 ± 0 | 2.33 ± 2.33 | 0 ± 0 | 0 ± 0 | 2.33 ± 1.2 | 0 ± 0 | 0 ± 0 | 0 ± 0 | 0.33 ± 0.33 |
| Coniochaeta sp. NG_p46 | 0 ± 0 | 0.67 ± 0.67 | 0 ± 0 | 0 ± 0 | 2.33 ± 1.86 | 0 ± 0 | 0 ± 0 | 0 ± 0 | 0 ± 0 | 1 ± 1 |
| Coniochaeta verticillata | 0 ± 0 | 0 ± 0 | 0 ± 0 | 0 ± 0 | 0 ± 0 | 0 ± 0 | 0 ± 0 | 1.67 ± 1.2 | 1.67 ± 1.67 | 0 ± 0 |
| Coniophora puteana | 0 ± 0 | 0 ± 0 | 0 ± 0 | 0 ± 0 | 0 ± 0 | 0 ± 0 | 0 ± 0 | 0 ± 0 | 0.33 ± 0.33 | 0 ± 0 |
| Conioscypha bambusicola | 0 ± 0 | 0 ± 0 | 0 ± 0 | 0 ± 0 | 0 ± 0 | 0 ± 0 | 0 ± 0 | 0 ± 0 | 1.33 ± 1.33 | 0 ± 0 |
| Conioscypha varia | 0 ± 0 | 1 ± 1 | 0 ± 0 | 0 ± 0 | 0 ± 0 | 0 ± 0 | 0 ± 0 | 0 ± 0 | 0 ± 0 | 0 ± 0 |
| Coniosporium sp. Sigrf15 | 0 ± 0 | 64.33 ± 40.24 | 0 ± 0 | 1 ± 0.58 | 0 ± 0 | 1.33 ± 1.33 | 5.67 ± 5.17 | 1.67 ± 1.2 | 4.33 ± 2.19 | 57.67 ± 50.78 |
| Coniosporium uncinatum | 2 ± 2 | 0 ± 0 | 0 ± 0 | 0 ± 0 | 0 ± 0 | 0 ± 0 | 0 ± 0 | 0 ± 0 | 0 ± 0 | 0 ± 0 |
| Coniothyrium crepinianum | 0 ± 0 | 2 ± 1.53 | 30.67 ± 6.06 | 0 ± 0 | 0.67 ± 0.33 | 19 ± 12 | 5 ± 2.52 | 2 ± 1 | 0 ± 0 | 0 ± 0 |
| Coniothyrium insitivum | 0 ± 0 | 0 ± 0 | 0 ± 0 | 0.67 ± 0.67 | 0 ± 0 | 0 ± 0 | 0 ± 0 | 0 ± 0 | 0 ± 0 | 0 ± 0 |
| Coniothyrium sp. | 0 ± 0 | 0 ± 0 | 0.33 ± 0.33 | 0 ± 0 | 0 ± 0 | 0 ± 0 | 0.33 ± 0.33 | 0 ± 0 | 0 ± 0 | 3 ± 2.52 |
| Coniothyrium sp. FF-2011 | 0 ± 0 | 0 ± 0 | 0 ± 0 | 0 ± 0 | 0 ± 0 | 0 ± 0 | 0 ± 0 | 0 ± 0 | 0.67 ± 0.67 | 0 ± 0 |
| Coniothyrium sp. TS-2016 | 2.67 ± 0.88 | 0.67 ± 0.33 | 12 ± 6.51 | 6 ± 2.89 | 0.67 ± 0.33 | 23.67 ± 2.4 | 30.33 ± 18.66 | 2.67 ± 1.45 | 5 ± 5 | 11 ± 4.04 |
| Conlarium sp. V1-1.2-NN-2016 | 0 ± 0 | 8.33 ± 2.73 | 1 ± 1 | 0.33 ± 0.33 | 0 ± 0 | 0 ± 0 | 1.33 ± 1.33 | 0 ± 0 | 0.33 ± 0.33 | 0 ± 0 |
| Conocybe juniana | 0 ± 0 | 0 ± 0 | 0 ± 0 | 0 ± 0 | 0 ± 0 | 1 ± 1 | 0 ± 0 | 0 ± 0 | 0 ± 0 | 0 ± 0 |
| Conocybe moseri | 0 ± 0 | 0 ± 0 | 4.67 ± 2.33 | 0 ± 0 | 0 ± 0 | 0 ± 0 | 0 ± 0 | 0 ± 0 | 0 ± 0 | 0 ± 0 |
| Conocybe rickenii | 0 ± 0 | 0 ± 0 | 0 ± 0 | 0 ± 0 | 0 ± 0 | 0.67 ± 0.67 | 0 ± 0 | 0 ± 0 | 0 ± 0 | 0 ± 0 |
| Coprinellus aff. radians | 0 ± 0 | 0 ± 0 | 1 ± 0.58 | 0 ± 0 | 0 ± 0 | 0.33 ± 0.33 | 0 ± 0 | 0 ± 0 | 0 ± 0 | 0 ± 0 |
| Coprinellus angulatus | 0 ± 0 | 0 ± 0 | 0.33 ± 0.33 | 0 ± 0 | 0 ± 0 | 1.67 ± 1.2 | 0 ± 0 | 0 ± 0 | 0 ± 0 | 0 ± 0 |
| Coprinellus micaceus | 0.33 ± 0.33 | 0 ± 0 | 0 ± 0 | 0 ± 0 | 0.33 ± 0.33 | 3 ± 2.08 | 5 ± 4.51 | 2.33 ± 2.33 | 0.67 ± 0.67 | 0 ± 0 |
| Coprinellus radians | 0.33 ± 0.33 | 0 ± 0 | 0 ± 0 | 0 ± 0 | 0.67 ± 0.67 | 0.33 ± 0.33 | 0 ± 0 | 0 ± 0 | 0.33 ± 0.33 | 0 ± 0 |
| Coprinellus sp. | 0 ± 0 | 0 ± 0 | 0.33 ± 0.33 | 0.33 ± 0.33 | 2.67 ± 1.2 | 3 ± 2.52 | 8.33 ± 0.33 | 0 ± 0 | 0 ± 0 | 0.33 ± 0.33 |
| Coprinellus sp. L21_2 | 0 ± 0 | 0 ± 0 | 16.67 ± 0.67 | 0 ± 0 | 0 ± 0 | 9 ± 5.69 | 0.67 ± 0.33 | 0.67 ± 0.67 | 2.67 ± 2.67 | 0 ± 0 |
| Coprinellus verrucispermus | 0 ± 0 | 0.33 ± 0.33 | 0 ± 0 | 0 ± 0 | 0 ± 0 | 0 ± 0 | 1.33 ± 1.33 | 0 ± 0 | 1.33 ± 1.33 | 0 ± 0 |
| Coprinopsis atramentaria | 0 ± 0 | 0 ± 0 | 0 ± 0 | 0 ± 0 | 0 ± 0 | 0 ± 0 | 0.33 ± 0.33 | 0.33 ± 0.33 | 0 ± 0 | 0.67 ± 0.67 |
| Coprinopsis cinerea | 0 ± 0 | 1.67 ± 1.67 | 0 ± 0 | 0 ± 0 | 0.33 ± 0.33 | 0.33 ± 0.33 | 0 ± 0 | 0 ± 0 | 0 ± 0 | 0.33 ± 0.33 |
| Coprinopsis udicola | 1.67 ± 0.88 | 0 ± 0 | 0 ± 0 | 0 ± 0 | 0.33 ± 0.33 | 0 ± 0 | 0 ± 0 | 0 ± 0 | 0 ± 0 | 0.33 ± 0.33 |
| Coprinus cortinatus | 0 ± 0 | 0 ± 0 | 0 ± 0 | 0 ± 0 | 0 ± 0 | 2 ± 2 | 0 ± 0 | 0 ± 0 | 0 ± 0 | 0 ± 0 |
| Cordana bisbyi | 0 ± 0 | 0 ± 0 | 0 ± 0 | 0 ± 0 | 0 ± 0 | 2 ± 2 | 0 ± 0 | 0 ± 0 | 0 ± 0 | 0.33 ± 0.33 |
| Cordana mercadiana | 0 ± 0 | 0 ± 0 | 0 ± 0 | 0 ± 0 | 0 ± 0 | 0 ± 0 | 0 ± 0 | 0 ± 0 | 0 ± 0 | 1.67 ± 1.2 |
| Cordana terrestris | 0 ± 0 | 0 ± 0 | 22.67 ± 4.18 | 0 ± 0 | 0 ± 0 | 8 ± 4.16 | 4.33 ± 2.4 | 5 ± 1.73 | 16.33 ± 11.29 | 8 ± 0.58 |
| Cordyceps amoene-rosea | 0 ± 0 | 1.33 ± 1.33 | 1.33 ± 1.33 | 11.67 ± 7.45 | 0 ± 0 | 0 ± 0 | 19.67 ± 6.67 | 0.33 ± 0.33 | 0 ± 0 | 0 ± 0 |
| Cordyceps cicadae | 0 ± 0 | 0 ± 0 | 1.67 ± 1.67 | 1 ± 0.58 | 0 ± 0 | 3.67 ± 3.67 | 2.33 ± 0.67 | 0 ± 0 | 0 ± 0 | 0.67 ± 0.67 |
| Cordyceps farinosa | 0 ± 0 | 0 ± 0 | 0 ± 0 | 0.33 ± 0.33 | 1.33 ± 0.67 | 13.33 ± 9.61 | 5.33 ± 3.53 | 7.67 ± 7.17 | 1.67 ± 1.67 | 2.33 ± 1.2 |
| Cordyceps fumosorosea | 0 ± 0 | 0 ± 0 | 4.33 ± 2.85 | 0.33 ± 0.33 | 0.67 ± 0.33 | 12 ± 7.09 | 12.67 ± 7.69 | 16.67 ± 8.82 | 6 ± 5.51 | 11 ± 1.53 |
| Cordyceps javanica | 0.33 ± 0.33 | 0.33 ± 0.33 | 0 ± 0 | 10.33 ± 3.38 | 2 ± 1.15 | 5.67 ± 3.18 | 10.33 ± 1.76 | 0 ± 0 | 0.33 ± 0.33 | 22 ± 9.07 |
| Cordyceps militaris | 0 ± 0 | 0 ± 0 | 0 ± 0 | 0 ± 0 | 0 ± 0 | 0 ± 0 | 0 ± 0 | 0 ± 0 | 0 ± 0 | 3 ± 1.53 |
| Cordyceps sp. HN-D1 | 0 ± 0 | 0 ± 0 | 0 ± 0 | 0 ± 0 | 0 ± 0 | 0 ± 0 | 0.33 ± 0.33 | 0 ± 0 | 0 ± 0 | 0 ± 0 |
| Cordyceps sp. XSD-72 | 0 ± 0 | 0 ± 0 | 0 ± 0 | 0.33 ± 0.33 | 0 ± 0 | 2.33 ± 1.45 | 20 ± 19.5 | 0 ± 0 | 0.33 ± 0.33 | 0.33 ± 0.33 |
| Cordyceps takaomontana | 0.33 ± 0.33 | 0 ± 0 | 0 ± 0 | 0 ± 0 | 3 ± 1.53 | 0.33 ± 0.33 | 0.33 ± 0.33 | 0 ± 0 | 0 ± 0 | 0 ± 0 |
| Corinectria fuckeliana | 0 ± 0 | 0 ± 0 | 0 ± 0 | 0 ± 0 | 0 ± 0 | 0.67 ± 0.67 | 0 ± 0 | 0 ± 0 | 0 ± 0 | 0 ± 0 |
| Corticium confine | 0.33 ± 0.33 | 0 ± 0 | 5.67 ± 1.2 | 0 ± 0 | 0 ± 0 | 0.33 ± 0.33 | 1.33 ± 0.67 | 0 ± 0 | 0.67 ± 0.67 | 0 ± 0 |
| Corticium sp. (in: Fungi) | 0 ± 0 | 1.33 ± 0.67 | 0 ± 0 | 18.33 ± 4.18 | 0 ± 0 | 0 ± 0 | 10 ± 5.29 | 0 ± 0 | 0 ± 0 | 0 ± 0 |
| Cortinarius distans | 0 ± 0 | 0 ± 0 | 0 ± 0 | 0 ± 0 | 0 ± 0 | 0 ± 0 | 0 ± 0 | 0 ± 0 | 0 ± 0 | 7 ± 7 |
| Corynascella humicola | 0 ± 0 | 0.33 ± 0.33 | 0 ± 0 | 0 ± 0 | 0 ± 0 | 1 ± 1 | 0.67 ± 0.67 | 0 ± 0 | 0 ± 0 | 0 ± 0 |
| Corynespora cassiicola | 0.33 ± 0.33 | 0 ± 0 | 0 ± 0 | 0 ± 0 | 1.33 ± 1.33 | 0 ± 0 | 0 ± 0 | 0 ± 0 | 0 ± 0 | 0 ± 0 |
| Cosmospora cymosa | 0 ± 0 | 0 ± 0 | 0.33 ± 0.33 | 0.33 ± 0.33 | 0 ± 0 | 0.67 ± 0.67 | 0.33 ± 0.33 | 0 ± 0 | 0 ± 0 | 0 ± 0 |
| Cosmospora gigas | 0 ± 0 | 0 ± 0 | 0 ± 0 | 0 ± 0 | 0 ± 0 | 1.33 ± 1.33 | 0.67 ± 0.67 | 0 ± 0 | 0 ± 0 | 0 ± 0 |
| Cosmospora viridescens | 0 ± 0 | 0 ± 0 | 0 ± 0 | 0 ± 0 | 0 ± 0 | 0 ± 0 | 0 ± 0 | 0 ± 0 | 0 ± 0 | 0.67 ± 0.67 |
| Cotylidia carpatica | 0 ± 0 | 8.67 ± 4.91 | 0 ± 0 | 0.33 ± 0.33 | 0 ± 0 | 0 ± 0 | 1 ± 0.58 | 0 ± 0 | 0 ± 0 | 0 ± 0 |
| Crassiclypeus aquaticus | 0 ± 0 | 0 ± 0 | 0.33 ± 0.33 | 0 ± 0 | 0 ± 0 | 0 ± 0 | 0 ± 0 | 0 ± 0 | 0 ± 0 | 0 ± 0 |
| Creosphaeria sassafras | 0 ± 0 | 0 ± 0 | 0 ± 0 | 0 ± 0 | 0 ± 0 | 5 ± 2.52 | 3.67 ± 3.18 | 1.33 ± 1.33 | 0 ± 0 | 0.67 ± 0.67 |
| Crepidotus malachioides | 0 ± 0 | 0 ± 0 | 0 ± 0 | 0 ± 0 | 0 ± 0 | 0 ± 0 | 0 ± 0 | 0.33 ± 0.33 | 1.67 ± 1.67 | 0 ± 0 |
| Crustoderma corneum | 0 ± 0 | 0 ± 0 | 1 ± 1 | 0 ± 0 | 0 ± 0 | 0.33 ± 0.33 | 0 ± 0 | 0 ± 0 | 0 ± 0 | 0 ± 0 |
| Cryomyces antarcticus | 0 ± 0 | 0 ± 0 | 0 ± 0 | 0 ± 0 | 0 ± 0 | 0 ± 0 | 0 ± 0 | 3 ± 3 | 0 ± 0 | 0 ± 0 |
| Cryptococcus sp. | 0 ± 0 | 0 ± 0 | 0 ± 0 | 0 ± 0 | 0 ± 0 | 0 ± 0 | 0 ± 0 | 0 ± 0 | 0 ± 0 | 0.67 ± 0.67 |
| Cryptococcus sp. 2 IA06 | 0.33 ± 0.33 | 1.33 ± 0.88 | 0.33 ± 0.33 | 0 ± 0 | 0 ± 0 | 0 ± 0 | 0.33 ± 0.33 | 7.67 ± 4.33 | 0 ± 0 | 2.33 ± 1.2 |
| Cryptococcus sp. 2 TMS-2011 | 0.33 ± 0.33 | 0 ± 0 | 0.33 ± 0.33 | 0.33 ± 0.33 | 1.67 ± 1.2 | 0.33 ± 0.33 | 0 ± 0 | 6.67 ± 4.41 | 0 ± 0 | 0 ± 0 |
| Cryptococcus sp. DMKU-CP250 | 0 ± 0 | 0 ± 0 | 0 ± 0 | 0 ± 0 | 5.33 ± 3.18 | 0.67 ± 0.67 | 0 ± 0 | 0 ± 0 | 0 ± 0 | 0 ± 0 |
| Cryptococcus sp. DMKU-SP423 | 0 ± 0 | 0 ± 0 | 0 ± 0 | 0 ± 0 | 0 ± 0 | 0 ± 0 | 0 ± 0 | 0 ± 0 | 0 ± 0 | 1.33 ± 1.33 |
| Cryptococcus sp. GT-333 | 0 ± 0 | 0 ± 0 | 0 ± 0 | 6.67 ± 6.17 | 0 ± 0 | 2.67 ± 1.76 | 0.67 ± 0.67 | 1.67 ± 1.67 | 9 ± 9 | 0 ± 0 |
| Cryptococcus sp. GT-388 | 0 ± 0 | 0 ± 0 | 0 ± 0 | 0 ± 0 | 1.33 ± 0.88 | 0.67 ± 0.67 | 0 ± 0 | 0 ± 0 | 3.33 ± 3.33 | 0 ± 0 |
| Cryptococcus sp. KY-763 | 2.33 ± 1.45 | 0 ± 0 | 0.67 ± 0.33 | 6.33 ± 3.38 | 61.67 ± 24.73 | 3 ± 1.53 | 5.67 ± 1.33 | 0.67 ± 0.33 | 3 ± 0.58 | 306 ± 67.64 |
| Cryptococcus sp. LCF-27 | 0 ± 0 | 0 ± 0 | 0 ± 0 | 2.33 ± 1.86 | 0 ± 0 | 0 ± 0 | 0.33 ± 0.33 | 0 ± 0 | 0 ± 0 | 0 ± 0 |
| Cryptococcus sp. S-27 | 0 ± 0 | 0 ± 0 | 2.33 ± 2.33 | 0 ± 0 | 0 ± 0 | 0.67 ± 0.67 | 0 ± 0 | 0 ± 0 | 0 ± 0 | 0 ± 0 |
| Cryptococcus sp. YKS 2004 | 0 ± 0 | 0 ± 0 | 1 ± 1 | 0 ± 0 | 0 ± 0 | 0 ± 0 | 0 ± 0 | 0 ± 0 | 0 ± 0 | 0 ± 0 |
| Cryptocoryneum condensatum | 0 ± 0 | 0.33 ± 0.33 | 0 ± 0 | 0 ± 0 | 0 ± 0 | 0 ± 0 | 0.33 ± 0.33 | 0 ± 0 | 0 ± 0 | 0 ± 0 |
| Cryptodiscus epicladonia | 0 ± 0 | 0 ± 0 | 0 ± 0 | 0 ± 0 | 0 ± 0 | 0 ± 0 | 0 ± 0 | 0 ± 0 | 0 ± 0 | 0 ± 0 |
| Cryptosphaeria subcutanea | 0 ± 0 | 0 ± 0 | 0 ± 0 | 0 ± 0 | 0 ± 0 | 0 ± 0 | 0.67 ± 0.67 | 0 ± 0 | 0 ± 0 | 0 ± 0 |
| Cryptosporiopsis sp. CBS 433.75 | 0.33 ± 0.33 | 3 ± 3 | 1 ± 0.58 | 3.67 ± 1.86 | 0 ± 0 | 1.33 ± 0.33 | 3.67 ± 1.76 | 0.67 ± 0.67 | 0.33 ± 0.33 | 1 ± 1 |
| Curreya sp. OUCMBI101084 | 0 ± 0 | 0 ± 0 | 0 ± 0 | 0.33 ± 0.33 | 0 ± 0 | 1.67 ± 1.67 | 1.33 ± 1.33 | 0.33 ± 0.33 | 1.33 ± 1.33 | 1.33 ± 1.33 |
| Curvibasidium cygneicollum | 0 ± 0 | 0 ± 0 | 0 ± 0 | 0 ± 0 | 0 ± 0 | 0.33 ± 0.33 | 0.33 ± 0.33 | 0 ± 0 | 0 ± 0 | 0.33 ± 0.33 |
| Curvularia coatesiae | 0 ± 0 | 1.33 ± 1.33 | 0 ± 0 | 0 ± 0 | 0 ± 0 | 0 ± 0 | 0 ± 0 | 0 ± 0 | 0 ± 0 | 0 ± 0 |
| Curvularia lunata | 0 ± 0 | 0 ± 0 | 1 ± 1 | 0 ± 0 | 0 ± 0 | 0.33 ± 0.33 | 0 ± 0 | 0.67 ± 0.33 | 0 ± 0 | 2.33 ± 2.33 |
| Cutaneotrichosporon moniliiforme | 0 ± 0 | 0 ± 0 | 0 ± 0 | 0.33 ± 0.33 | 0.33 ± 0.33 | 0.33 ± 0.33 | 0.33 ± 0.33 | 0 ± 0 | 1.67 ± 1.67 | 5.67 ± 5.67 |
| Cylindrocarpon sp. | 49 ± 22.19 | 111.33 ± 47.69 | 506 ± 78.89 | 32.67 ± 12.35 | 91.33 ± 4.91 | 552.33 ± 54.39 | 823.67 ± 696.1 | 43.33 ± 23.48 | 18 ± 11.02 | 129.33 ± 9.74 |
| Cylindrocarpon sp. FKI-4602 | 0.33 ± 0.33 | 0 ± 0 | 3.67 ± 0.67 | 1.67 ± 1.67 | 0.67 ± 0.33 | 5.33 ± 1.2 | 51.67 ± 50.17 | 0 ± 0 | 0.67 ± 0.67 | 3 ± 1.53 |
| Cylindrocarpon sp. PB1-R7-A Lr | 0.33 ± 0.33 | 0.33 ± 0.33 | 82 ± 6 | 0.67 ± 0.67 | 0.33 ± 0.33 | 32.67 ± 14.4 | 2.33 ± 1.45 | 0 ± 0 | 0 ± 0 | 1.33 ± 1.33 |
| Cylindrocladiella peruviana | 0.33 ± 0.33 | 0.33 ± 0.33 | 4.33 ± 4.33 | 1.33 ± 1.33 | 7.67 ± 1.86 | 23 ± 14.64 | 27 ± 17.21 | 0 ± 0 | 0 ± 0 | 4.67 ± 4.67 |
| Cylindrodendrum hubeiense | 0 ± 0 | 0 ± 0 | 0 ± 0 | 0 ± 0 | 0 ± 0 | 0 ± 0 | 0.33 ± 0.33 | 0 ± 0 | 1.33 ± 0.88 | 1 ± 1 |
| Cylindrosympodium variabile | 0 ± 0 | 0 ± 0 | 0 ± 0 | 0 ± 0 | 0 ± 0 | 0 ± 0 | 0 ± 0 | 0 ± 0 | 0 ± 0 | 0 ± 0 |
| Cyphellophora eucalypti | 0 ± 0 | 0 ± 0 | 0 ± 0 | 0 ± 0 | 0.67 ± 0.33 | 1.67 ± 1.67 | 0 ± 0 | 0 ± 0 | 0 ± 0 | 0 ± 0 |
| Cyphellophora europaea | 0 ± 0 | 0 ± 0 | 0.67 ± 0.67 | 0 ± 0 | 0.67 ± 0.33 | 3.33 ± 1.67 | 5.33 ± 3.33 | 1.67 ± 0.88 | 2.67 ± 1.45 | 2.33 ± 2.33 |
| Cyphellophora fusarioides | 0 ± 0 | 0 ± 0 | 0 ± 0 | 0 ± 0 | 0 ± 0 | 0 ± 0 | 0 ± 0 | 0.67 ± 0.67 | 8 ± 8 | 0 ± 0 |
| Cyphellophora gamsii | 13.67 ± 8.74 | 0.67 ± 0.33 | 0.33 ± 0.33 | 0.67 ± 0.33 | 2 ± 1.15 | 3 ± 0.58 | 5 ± 2.31 | 118 ± 101.24 | 29.67 ± 28.18 | 5.33 ± 3.84 |
| Cyphellophora guyanensis | 0 ± 0 | 0 ± 0 | 0 ± 0 | 0 ± 0 | 0 ± 0 | 0 ± 0 | 0 ± 0 | 2 ± 2 | 0 ± 0 | 0 ± 0 |
| Cyphellophora jingdongensis | 0 ± 0 | 0 ± 0 | 0 ± 0 | 0.33 ± 0.33 | 0 ± 0 | 0 ± 0 | 0.33 ± 0.33 | 9 ± 5.57 | 1.67 ± 1.2 | 0 ± 0 |
| Cyphellophora laciniata | 3.67 ± 2.19 | 0 ± 0 | 2.67 ± 0.88 | 0 ± 0 | 0.67 ± 0.33 | 11 ± 7.64 | 1.33 ± 1.33 | 5.67 ± 2.6 | 12.33 ± 12.33 | 0 ± 0 |
| Cyphellophora musae | 0 ± 0 | 0 ± 0 | 0 ± 0 | 0 ± 0 | 0 ± 0 | 0 ± 0 | 1.67 ± 1.67 | 0 ± 0 | 0 ± 0 | 0 ± 0 |
| Cyphellophora olivacea | 0 ± 0 | 0 ± 0 | 0 ± 0 | 0.33 ± 0.33 | 0 ± 0 | 0 ± 0 | 0 ± 0 | 0 ± 0 | 0 ± 0 | 0 ± 0 |
| Cyphellophora oxyspora | 0 ± 0 | 0 ± 0 | 0 ± 0 | 0 ± 0 | 0.67 ± 0.67 | 0 ± 0 | 0 ± 0 | 0 ± 0 | 0 ± 0 | 0 ± 0 |
| Cyphellophora pauciseptata | 0 ± 0 | 0.67 ± 0.67 | 13 ± 11.5 | 0 ± 0 | 1.33 ± 1.33 | 4.67 ± 2.19 | 0.67 ± 0.33 | 11.33 ± 9.84 | 6 ± 6 | 0 ± 0 |
| Cyphellophora pluriseptata | 0 ± 0 | 0 ± 0 | 0 ± 0 | 0 ± 0 | 0 ± 0 | 0 ± 0 | 0 ± 0 | 6 ± 3.46 | 2.33 ± 2.33 | 0 ± 0 |
| Cyphellophora sessilis | 2.33 ± 1.2 | 0 ± 0 | 0.67 ± 0.33 | 1.67 ± 1.2 | 0.33 ± 0.33 | 4.67 ± 2.19 | 9 ± 6.03 | 9.67 ± 3.28 | 16 ± 13.5 | 6 ± 3.21 |
| Cyphellophora sp. | 0 ± 0 | 0 ± 0 | 0 ± 0 | 0 ± 0 | 0 ± 0 | 1.33 ± 0.67 | 2.33 ± 2.33 | 0 ± 0 | 0 ± 0 | 0 ± 0 |
| Cyphellophoriella pruni | 0 ± 0 | 0 ± 0 | 0 ± 0 | 0.33 ± 0.33 | 0 ± 0 | 0 ± 0 | 0 ± 0 | 0 ± 0 | 0 ± 0 | 0 ± 0 |
| Cystobasidium calyptogenae | 0 ± 0 | 0 ± 0 | 0 ± 0 | 0 ± 0 | 0 ± 0 | 0.67 ± 0.33 | 0 ± 0 | 0 ± 0 | 0 ± 0 | 0 ± 0 |
| Cystobasidium minutum | 0 ± 0 | 0 ± 0 | 0 ± 0 | 0 ± 0 | 0 ± 0 | 0 ± 0 | 0 ± 0 | 0 ± 0 | 0 ± 0 | 1 ± 1 |
| Cystofilobasidium capitatum | 0 ± 0 | 0 ± 0 | 36.33 ± 10.99 | 0.67 ± 0.33 | 2 ± 2 | 6 ± 4.51 | 37.67 ± 36.17 | 31.33 ± 19.81 | 5.67 ± 4.18 | 15.33 ± 7.86 |
| Cystofilobasidium infirmominiatum | 0 ± 0 | 0 ± 0 | 2.67 ± 1.45 | 0.33 ± 0.33 | 0.67 ± 0.33 | 1 ± 1 | 0 ± 0 | 0 ± 0 | 0 ± 0 | 1.33 ± 1.33 |
| Cystostereum murrayi | 0 ± 0 | 0 ± 0 | 0 ± 0 | 0 ± 0 | 0 ± 0 | 0 ± 0 | 2.33 ± 2.33 | 0 ± 0 | 0 ± 0 | 0 ± 0 |
| Dactylaria acaciae | 1.33 ± 1.33 | 0 ± 0 | 0.67 ± 0.67 | 0 ± 0 | 0.33 ± 0.33 | 21.67 ± 19.7 | 4 ± 1.53 | 16.33 ± 14.86 | 1.67 ± 1.67 | 0.67 ± 0.67 |
| Dactylaria appendiculata | 0.33 ± 0.33 | 19 ± 7.77 | 51.33 ± 11.78 | 2.67 ± 0.67 | 0 ± 0 | 10.67 ± 7.86 | 6 ± 3.79 | 2.33 ± 2.33 | 13.33 ± 6.69 | 3.33 ± 3.33 |
| Dactylaria dimorphospora | 0 ± 0 | 0 ± 0 | 6 ± 4.04 | 0 ± 0 | 1.67 ± 1.2 | 1 ± 0.58 | 0.33 ± 0.33 | 59.67 ± 33.02 | 15.33 ± 14.84 | 0 ± 0 |
| Dactylaria fragilis | 0 ± 0 | 0 ± 0 | 0 ± 0 | 0 ± 0 | 0 ± 0 | 0 ± 0 | 0 ± 0 | 0 ± 0 | 0.33 ± 0.33 | 0.67 ± 0.67 |
| Dactylaria sp. 20417 | 0 ± 0 | 0 ± 0 | 2.67 ± 1.2 | 0.67 ± 0.67 | 0 ± 0 | 6.33 ± 4.91 | 8 ± 7.51 | 1.67 ± 1.2 | 2 ± 2 | 0 ± 0 |
| Dactylella oxyspora | 0 ± 0 | 0 ± 0 | 2 ± 1.53 | 0 ± 0 | 0.33 ± 0.33 | 2.67 ± 1.45 | 0 ± 0 | 0 ± 0 | 0 ± 0 | 0 ± 0 |
| Dactylella tenuifusaria | 0 ± 0 | 0 ± 0 | 0 ± 0 | 0 ± 0 | 0 ± 0 | 0 ± 0 | 0 ± 0 | 105.33 ± 53.14 | 55 ± 55 | 0.67 ± 0.33 |
| Dactylellina parvicollis | 0 ± 0 | 0 ± 0 | 0 ± 0 | 0 ± 0 | 0 ± 0 | 0 ± 0 | 0 ± 0 | 0 ± 0 | 0 ± 0 | 0.67 ± 0.67 |
| Dactylonectria alcacerensis | 72 ± 13.86 | 7.67 ± 2.33 | 485 ± 115.77 | 55.67 ± 13.59 | 378.33 ± 149.07 | 1009.67 ± 266.78 | 675.67 ± 493.01 | 73.33 ± 29.49 | 50.33 ± 38.34 | 76.33 ± 30.68 |
| Dactylonectria pauciseptata | 10.67 ± 1.86 | 12.33 ± 6.36 | 129.67 ± 66.87 | 66 ± 22.23 | 91.67 ± 18.52 | 61.33 ± 52.41 | 55.67 ± 3.33 | 53 ± 8 | 55.67 ± 19.38 | 349.67 ± 65.95 |
| Dactylonectria vitis | 24 ± 7.21 | 1 ± 0.58 | 276.33 ± 20.54 | 5.67 ± 0.67 | 77 ± 22.37 | 147 ± 60.56 | 14.33 ± 7.26 | 28 ± 15.14 | 35 ± 34 | 2.33 ± 1.2 |
| Daldinia concentrica | 0 ± 0 | 0 ± 0 | 0 ± 0 | 0.67 ± 0.67 | 0 ± 0 | 0 ± 0 | 0 ± 0 | 0 ± 0 | 0 ± 0 | 0 ± 0 |
| Daldinia sp. | 0 ± 0 | 0 ± 0 | 0 ± 0 | 0 ± 0 | 0 ± 0 | 0 ± 0 | 0.67 ± 0.67 | 1 ± 1 | 0.33 ± 0.33 | 0 ± 0 |
| Davidhawksworthia ilicicola | 0 ± 0 | 0 ± 0 | 2.33 ± 2.33 | 0 ± 0 | 0 ± 0 | 1 ± 1 | 0 ± 0 | 0 ± 0 | 0 ± 0 | 0 ± 0 |
| Debaryomyces hansenii | 0 ± 0 | 0 ± 0 | 0 ± 0 | 0 ± 0 | 0 ± 0 | 0 ± 0 | 0 ± 0 | 1.33 ± 1.33 | 0 ± 0 | 0 ± 0 |
| Deconica coprophila | 0 ± 0 | 0 ± 0 | 0 ± 0 | 0 ± 0 | 0 ± 0 | 0.67 ± 0.67 | 0 ± 0 | 0 ± 0 | 0 ± 0 | 0 ± 0 |
| Deconica sp. 471 | 0 ± 0 | 0 ± 0 | 0 ± 0 | 0 ± 0 | 0 ± 0 | 0 ± 0 | 0 ± 0 | 2 ± 2 | 0 ± 0 | 0 ± 0 |
| Degelia plumbea | 1 ± 1 | 2.33 ± 2.33 | 6 ± 2.65 | 3 ± 0.58 | 0 ± 0 | 2 ± 1.15 | 27 ± 25.03 | 0.33 ± 0.33 | 0 ± 0 | 0.33 ± 0.33 |
| Delastria sp. | 0.33 ± 0.33 | 0 ± 0 | 2 ± 2 | 0 ± 0 | 0.67 ± 0.33 | 0 ± 0 | 0 ± 0 | 0 ± 0 | 0 ± 0 | 0.33 ± 0.33 |
| Delastria sp. AH39200 | 0 ± 0 | 0 ± 0 | 0 ± 0 | 0 ± 0 | 0 ± 0 | 0 ± 0 | 0 ± 0 | 1.33 ± 1.33 | 0 ± 0 | 0 ± 0 |
| Dendroclathra lignicola | 0 ± 0 | 0 ± 0 | 6.67 ± 4.7 | 0 ± 0 | 2.67 ± 2.19 | 2.67 ± 2.67 | 0.33 ± 0.33 | 0 ± 0 | 0 ± 0 | 0 ± 0 |
| Dendrosporium sp. 1 RB-2011 | 0 ± 0 | 0 ± 0 | 0 ± 0 | 0 ± 0 | 0 ± 0 | 0 ± 0 | 0 ± 0 | 5.67 ± 4.7 | 5.67 ± 5.67 | 0 ± 0 |
| Dendryphion comosum | 3.67 ± 1.76 | 0.33 ± 0.33 | 1 ± 0.58 | 0.67 ± 0.33 | 1 ± 0.58 | 11 ± 2.08 | 4.33 ± 1.86 | 3 ± 3 | 0.33 ± 0.33 | 5.33 ± 2.73 |
| Dendryphion nanum | 0 ± 0 | 0 ± 0 | 0 ± 0 | 0 ± 0 | 0 ± 0 | 0 ± 0 | 0.67 ± 0.67 | 0 ± 0 | 0 ± 0 | 0 ± 0 |
| Densocarpa crocea | 0 ± 0 | 0 ± 0 | 7.67 ± 5.36 | 0.33 ± 0.33 | 0 ± 0 | 1.33 ± 1.33 | 21 ± 21 | 0 ± 0 | 4 ± 4 | 0 ± 0 |
| Dermea prunastri | 0 ± 0 | 0 ± 0 | 2.33 ± 1.86 | 0 ± 0 | 0 ± 0 | 10.33 ± 9.84 | 4.33 ± 4.33 | 0 ± 0 | 0 ± 0 | 0 ± 0 |
| Dermea viburni | 0.67 ± 0.67 | 0 ± 0 | 0.67 ± 0.33 | 3.33 ± 3.33 | 4.67 ± 1.2 | 20.67 ± 6.57 | 12 ± 7.57 | 0 ± 0 | 0 ± 0 | 0 ± 0 |
| Dermoloma sp. SAV4094 | 0 ± 0 | 0 ± 0 | 0 ± 0 | 0 ± 0 | 0 ± 0 | 0 ± 0 | 0 ± 0 | 0 ± 0 | 0 ± 0 | 15.67 ± 15.67 |
| Derxomyces mrakii | 0 ± 0 | 0 ± 0 | 0.67 ± 0.67 | 0 ± 0 | 0 ± 0 | 1.67 ± 1.67 | 0 ± 0 | 0.33 ± 0.33 | 0 ± 0 | 0.33 ± 0.33 |
| Devriesia americana | 0 ± 0 | 4.67 ± 2.33 | 0 ± 0 | 0.33 ± 0.33 | 0 ± 0 | 0 ± 0 | 3 ± 3 | 0 ± 0 | 0 ± 0 | 0 ± 0 |
| Devriesia pseudoamericana | 0 ± 0 | 0.33 ± 0.33 | 0 ± 0 | 0 ± 0 | 0 ± 0 | 0.33 ± 0.33 | 0 ± 0 | 0.33 ± 0.33 | 0 ± 0 | 0.33 ± 0.33 |
| Devriesia sp. GSH2_8 | 1 ± 1 | 0 ± 0 | 0 ± 0 | 0 ± 0 | 0 ± 0 | 0 ± 0 | 0.33 ± 0.33 | 0.67 ± 0.67 | 0 ± 0 | 0 ± 0 |
| Devriesia strelitziicola | 4.67 ± 0.88 | 673.33 ± 135.57 | 22 ± 8.5 | 68.33 ± 17.65 | 1.33 ± 0.67 | 13.67 ± 11.2 | 149 ± 47.34 | 91.33 ± 79.37 | 136 ± 91.98 | 528.33 ± 233.87 |
| Dialonectria episphaeria | 0 ± 0 | 0 ± 0 | 0 ± 0 | 0 ± 0 | 3 ± 3 | 0 ± 0 | 0 ± 0 | 0 ± 0 | 0 ± 0 | 0 ± 0 |
| Diaporthe columnaris | 0 ± 0 | 0 ± 0 | 0 ± 0 | 0 ± 0 | 0 ± 0 | 0 ± 0 | 3 ± 3 | 0 ± 0 | 0.67 ± 0.33 | 97 ± 95 |
| Diaporthe phaseolorum | 0 ± 0 | 0 ± 0 | 11.33 ± 11.33 | 0.33 ± 0.33 | 0 ± 0 | 0.67 ± 0.67 | 0 ± 0 | 0 ± 0 | 0 ± 0 | 0 ± 0 |
| Diaporthe sp. C2c7 | 0 ± 0 | 8 ± 7.02 | 0 ± 0 | 0 ± 0 | 0 ± 0 | 0 ± 0 | 0 ± 0 | 0 ± 0 | 0 ± 0 | 0 ± 0 |
| Diaporthe sp. CZ-2018 | 1 ± 1 | 0 ± 0 | 0 ± 0 | 0 ± 0 | 0 ± 0 | 0 ± 0 | 0 ± 0 | 0 ± 0 | 0 ± 0 | 0 ± 0 |
| Dictyochaeta sp. RJ-2015 | 0 ± 0 | 0 ± 0 | 0 ± 0 | 0 ± 0 | 0 ± 0 | 0 ± 0 | 0 ± 0 | 0 ± 0 | 0 ± 0 | 0 ± 0 |
| Dictyocheirospora indica | 0 ± 0 | 0 ± 0 | 1.67 ± 1.67 | 0 ± 0 | 0 ± 0 | 1 ± 1 | 0.67 ± 0.67 | 1 ± 1 | 0 ± 0 | 0 ± 0 |
| Dictyosporella thailandensis | 0 ± 0 | 0.67 ± 0.67 | 0 ± 0 | 0 ± 0 | 0 ± 0 | 0 ± 0 | 0.33 ± 0.33 | 0 ± 0 | 0 ± 0 | 0.67 ± 0.67 |
| Dictyosporium sp. 19VA07 | 0 ± 0 | 0.67 ± 0.67 | 0 ± 0 | 0.67 ± 0.67 | 0 ± 0 | 0 ± 0 | 0 ± 0 | 0 ± 0 | 0 ± 0 | 0 ± 0 |
| Dicyma pulvinata | 0 ± 0 | 0 ± 0 | 0 ± 0 | 1.33 ± 1.33 | 0.67 ± 0.67 | 0 ± 0 | 0 ± 0 | 0 ± 0 | 0 ± 0 | 0 ± 0 |
| Didymella glomerata | 42 ± 23.3 | 25 ± 6.35 | 148.33 ± 62.77 | 95 ± 72.08 | 148.67 ± 47.13 | 681.67 ± 378.27 | 409 ± 74.8 | 268 ± 195 | 198.67 ± 194.17 | 81.67 ± 3.48 |
| Didymella hellebori | 0.33 ± 0.33 | 0.33 ± 0.33 | 139.67 ± 130.73 | 4.33 ± 1.86 | 1 ± 0.58 | 21.33 ± 3.93 | 4.33 ± 2.96 | 0.67 ± 0.67 | 2.67 ± 2.67 | 5.67 ± 4.26 |
| Didymella tanaceti | 17.33 ± 11.02 | 0.67 ± 0.67 | 4.33 ± 3.84 | 2.67 ± 2.67 | 6.67 ± 2.6 | 30.67 ± 20.28 | 18.67 ± 9.49 | 1 ± 1 | 0.33 ± 0.33 | 0 ± 0 |
| Dioszegia changbaiensis | 0 ± 0 | 0 ± 0 | 0 ± 0 | 0 ± 0 | 0 ± 0 | 0.67 ± 0.67 | 0 ± 0 | 0 ± 0 | 0 ± 0 | 0 ± 0 |
| Dioszegia hungarica | 0 ± 0 | 0 ± 0 | 0 ± 0 | 0 ± 0 | 4 ± 2.08 | 0 ± 0 | 0 ± 0 | 0 ± 0 | 0 ± 0 | 0 ± 0 |
| Dioszegia patagonica | 0.33 ± 0.33 | 0 ± 0 | 0 ± 0 | 0 ± 0 | 0 ± 0 | 0 ± 0 | 0 ± 0 | 0 ± 0 | 0 ± 0 | 0 ± 0 |
| Dioszegia sp. YA-2016 | 0 ± 0 | 0 ± 0 | 0 ± 0 | 0 ± 0 | 0 ± 0 | 0 ± 0 | 0 ± 0 | 1.33 ± 1.33 | 0 ± 0 | 0.33 ± 0.33 |
| Dioszegia takashimae | 0 ± 0 | 0 ± 0 | 0 ± 0 | 0.67 ± 0.67 | 0 ± 0 | 2.67 ± 2.67 | 0 ± 0 | 0 ± 0 | 0 ± 0 | 2.33 ± 1.86 |
| Dioszegia xingshanensis | 0 ± 0 | 0 ± 0 | 0 ± 0 | 0 ± 0 | 1 ± 0.58 | 1 ± 0.58 | 2 ± 2 | 3.67 ± 3.18 | 2.33 ± 2.33 | 7 ± 3.46 |
| Dioszegia zsoltii | 0.33 ± 0.33 | 0 ± 0 | 9.33 ± 6.17 | 1 ± 0.58 | 0 ± 0 | 2.33 ± 0.88 | 0 ± 0 | 0 ± 0 | 0 ± 0 | 1.67 ± 1.2 |
| Discosia pseudoartocreas | 0 ± 0 | 0 ± 0 | 24.67 ± 21.22 | 0.33 ± 0.33 | 0.33 ± 0.33 | 12.67 ± 12.17 | 0.67 ± 0.67 | 22.67 ± 14.62 | 2 ± 2 | 0 ± 0 |
| Distoseptispora suoluoensis | 0 ± 0 | 0 ± 0 | 0 ± 0 | 0 ± 0 | 0 ± 0 | 0 ± 0 | 0 ± 0 | 0 ± 0 | 0 ± 0 | 0.33 ± 0.33 |
| Diversispora sp. SR2 | 0 ± 0 | 0 ± 0 | 0 ± 0 | 0 ± 0 | 0 ± 0 | 0 ± 0 | 0.33 ± 0.33 | 0 ± 0 | 0 ± 0 | 0 ± 0 |
| Diversispora versiformis | 0 ± 0 | 0.67 ± 0.67 | 0 ± 0 | 0 ± 0 | 0 ± 0 | 0 ± 0 | 0 ± 0 | 0 ± 0 | 0 ± 0 | 0 ± 0 |
| Dokmaia sp. 1 TMS-2011 | 0 ± 0 | 0 ± 0 | 5 ± 1.15 | 0 ± 0 | 0 ± 0 | 1.33 ± 1.33 | 0 ± 0 | 2.33 ± 1.86 | 0 ± 0 | 0 ± 0 |
| Dokmaia sp. 2 TMS-2011 | 0 ± 0 | 0 ± 0 | 0 ± 0 | 0 ± 0 | 0 ± 0 | 3.33 ± 3.33 | 0 ± 0 | 0 ± 0 | 0 ± 0 | 0 ± 0 |
| Dokmaia sp. 46a | 2 ± 1 | 0 ± 0 | 1 ± 1 | 0 ± 0 | 0 ± 0 | 0 ± 0 | 0 ± 0 | 0 ± 0 | 0 ± 0 | 0.33 ± 0.33 |
| Dominikia aurea | 0.33 ± 0.33 | 0 ± 0 | 0 ± 0 | 0 ± 0 | 0 ± 0 | 0 ± 0 | 0 ± 0 | 0 ± 0 | 0 ± 0 | 0 ± 0 |
| Dominikia bernensis | 0.67 ± 0.33 | 0 ± 0 | 0 ± 0 | 7.67 ± 3.93 | 2.67 ± 1.33 | 0 ± 0 | 1.33 ± 0.33 | 6.33 ± 6.33 | 16.33 ± 10.9 | 2.67 ± 1.45 |
| Dominikia difficilevidera | 0 ± 0 | 0 ± 0 | 0 ± 0 | 0 ± 0 | 4 ± 2.08 | 0 ± 0 | 0 ± 0 | 0 ± 0 | 0 ± 0 | 0 ± 0 |
| Dominikia disticha | 0 ± 0 | 0 ± 0 | 0 ± 0 | 0 ± 0 | 0 ± 0 | 0 ± 0 | 0 ± 0 | 1.33 ± 1.33 | 14.33 ± 9.02 | 1.33 ± 0.88 |
| Dominikia duoreactiva | 0 ± 0 | 0 ± 0 | 0 ± 0 | 0 ± 0 | 0 ± 0 | 0 ± 0 | 0.33 ± 0.33 | 0.67 ± 0.67 | 0 ± 0 | 36 ± 12.74 |
| Dominikia indica | 0 ± 0 | 0 ± 0 | 0 ± 0 | 0 ± 0 | 1.67 ± 0.88 | 0 ± 0 | 0 ± 0 | 0 ± 0 | 0 ± 0 | 0 ± 0 |
| Dominikia iranica | 0 ± 0 | 0 ± 0 | 0 ± 0 | 0 ± 0 | 0 ± 0 | 0 ± 0 | 0 ± 0 | 0.33 ± 0.33 | 2.33 ± 1.45 | 0 ± 0 |
| Dominikia lithuanica | 0 ± 0 | 0 ± 0 | 0 ± 0 | 0 ± 0 | 0 ± 0 | 0 ± 0 | 0 ± 0 | 0 ± 0 | 0.67 ± 0.67 | 0 ± 0 |
| Donkioporia expansa | 0.33 ± 0.33 | 13.33 ± 4.41 | 0.33 ± 0.33 | 0 ± 0 | 0 ± 0 | 0 ± 0 | 1.33 ± 0.88 | 0 ± 0 | 0 ± 0 | 0 ± 0 |
| Doratomyces sp. 02NH10 | 0 ± 0 | 0 ± 0 | 22.33 ± 7.69 | 2 ± 1.53 | 0 ± 0 | 12.67 ± 2.67 | 95.67 ± 90.17 | 2.33 ± 0.33 | 1.67 ± 0.88 | 3.33 ± 2.4 |
| Dothiorella rosulata | 0 ± 0 | 0 ± 0 | 0 ± 0 | 0.33 ± 0.33 | 0.33 ± 0.33 | 4 ± 1.53 | 0 ± 0 | 0 ± 0 | 0 ± 0 | 0 ± 0 |
| Dothiorella viticola | 0.67 ± 0.67 | 0 ± 0 | 0 ± 0 | 0.33 ± 0.33 | 33.67 ± 2.91 | 0.67 ± 0.67 | 1.33 ± 0.88 | 4 ± 3.06 | 1.67 ± 1.67 | 0 ± 0 |
| Drechslera sp. | 0 ± 0 | 0.67 ± 0.67 | 0 ± 0 | 0 ± 0 | 0 ± 0 | 0 ± 0 | 0.33 ± 0.33 | 0 ± 0 | 0 ± 0 | 0 ± 0 |
| Drechslerella dactyloides | 0 ± 0 | 0 ± 0 | 11 ± 11 | 0.33 ± 0.33 | 0 ± 0 | 7 ± 5.13 | 1.33 ± 1.33 | 30 ± 19.29 | 2.33 ± 2.33 | 11.33 ± 7.88 |
| Ectophoma pomi | 0 ± 0 | 0 ± 0 | 0 ± 0 | 0 ± 0 | 0 ± 0 | 0 ± 0 | 0 ± 0 | 0.33 ± 0.33 | 0 ± 0 | 0 ± 0 |
| Efibula tuberculata | 0 ± 0 | 12.67 ± 2.91 | 0 ± 0 | 0 ± 0 | 0 ± 0 | 0 ± 0 | 0.67 ± 0.67 | 0 ± 0 | 0 ± 0 | 0 ± 0 |
| Eichleriella alliciens | 0 ± 0 | 0.33 ± 0.33 | 0 ± 0 | 0.67 ± 0.67 | 0.67 ± 0.67 | 0 ± 0 | 0 ± 0 | 0 ± 0 | 0 ± 0 | 0 ± 0 |
| Eichleriella flavida | 0 ± 0 | 0 ± 0 | 0 ± 0 | 6 ± 1.53 | 0 ± 0 | 0 ± 0 | 2.67 ± 1.76 | 0 ± 0 | 0 ± 0 | 0 ± 0 |
| Elaphomyces virgatosporus | 0 ± 0 | 0 ± 0 | 0 ± 0 | 0.67 ± 0.67 | 0 ± 0 | 0 ± 0 | 0.67 ± 0.67 | 0 ± 0 | 0 ± 0 | 0 ± 0 |
| Elsinoe diospyri | 3 ± 2.08 | 0 ± 0 | 0 ± 0 | 0 ± 0 | 0.67 ± 0.67 | 0 ± 0 | 0 ± 0 | 0 ± 0 | 0 ± 0 | 0 ± 0 |
| Elsinoe eucalyptigena | 0 ± 0 | 0 ± 0 | 0 ± 0 | 0 ± 0 | 0 ± 0 | 0 ± 0 | 0 ± 0 | 0 ± 0 | 0 ± 0 | 0 ± 0 |
| Elsinoe heveae | 0 ± 0 | 0 ± 0 | 0 ± 0 | 0 ± 0 | 0 ± 0 | 0 ± 0 | 0 ± 0 | 0.33 ± 0.33 | 0 ± 0 | 3.33 ± 3.33 |
| Elsinoe phaseoli | 0 ± 0 | 0 ± 0 | 0 ± 0 | 0 ± 0 | 0 ± 0 | 0.67 ± 0.67 | 0 ± 0 | 0 ± 0 | 0 ± 0 | 0.33 ± 0.33 |
| Emericellopsis maritima | 0 ± 0 | 0.33 ± 0.33 | 0 ± 0 | 0 ± 0 | 0 ± 0 | 0 ± 0 | 0.67 ± 0.67 | 0 ± 0 | 0 ± 0 | 0 ± 0 |
| Emericellopsis microspora | 0 ± 0 | 0 ± 0 | 0 ± 0 | 0 ± 0 | 0 ± 0 | 2 ± 2 | 0.67 ± 0.67 | 7 ± 3 | 5 ± 5 | 4 ± 4 |
| Emericellopsis stolkiae | 0 ± 0 | 0 ± 0 | 15.33 ± 8.41 | 0 ± 0 | 0.33 ± 0.33 | 0.67 ± 0.67 | 1 ± 1 | 1.33 ± 1.33 | 0.33 ± 0.33 | 0.33 ± 0.33 |
| Encoelia furfuracea | 0 ± 0 | 0 ± 0 | 0 ± 0 | 0 ± 0 | 0 ± 0 | 0 ± 0 | 0 ± 0 | 0 ± 0 | 0 ± 0 | 1.33 ± 1.33 |
| Endosporium sp. | 0 ± 0 | 0 ± 0 | 0 ± 0 | 0.33 ± 0.33 | 0 ± 0 | 0 ± 0 | 0 ± 0 | 0 ± 0 | 0.67 ± 0.67 | 3.67 ± 2.33 |
| Entoloma abortivum | 0 ± 0 | 0 ± 0 | 0 ± 0 | 0 ± 0 | 0 ± 0 | 1 ± 1 | 0 ± 0 | 0.33 ± 0.33 | 0 ± 0 | 0 ± 0 |
| Entoloma aprile | 0 ± 0 | 0 ± 0 | 0 ± 0 | 0 ± 0 | 0 ± 0 | 0 ± 0 | 0.33 ± 0.33 | 1 ± 1 | 0.67 ± 0.67 | 0 ± 0 |
| Entoloma byssisedum | 0 ± 0 | 0 ± 0 | 0.33 ± 0.33 | 0 ± 0 | 0 ± 0 | 3.33 ± 0.33 | 10.67 ± 9.68 | 1 ± 1 | 0 ± 0 | 0 ± 0 |
| Entoloma chalybaeum | 0 ± 0 | 0 ± 0 | 0 ± 0 | 0 ± 0 | 0 ± 0 | 0.67 ± 0.67 | 0 ± 0 | 0 ± 0 | 0 ± 0 | 0 ± 0 |
| Entoloma graphitipes | 11 ± 11 | 0 ± 0 | 0 ± 0 | 0 ± 0 | 1.67 ± 1.2 | 0 ± 0 | 0.33 ± 0.33 | 0 ± 0 | 0 ± 0 | 0 ± 0 |
| Entoloma juncinum | 0 ± 0 | 7.67 ± 3.38 | 3 ± 3 | 0 ± 0 | 0 ± 0 | 0.67 ± 0.67 | 1 ± 1 | 0.33 ± 0.33 | 1 ± 1 | 0.67 ± 0.67 |
| Entoloma mirum | 0 ± 0 | 0 ± 0 | 0 ± 0 | 0 ± 0 | 0 ± 0 | 0 ± 0 | 0 ± 0 | 0 ± 0 | 0 ± 0 | 0.67 ± 0.67 |
| Entoloma percoelestinum | 0 ± 0 | 0 ± 0 | 0 ± 0 | 0 ± 0 | 1.67 ± 1.67 | 0 ± 0 | 0 ± 0 | 0 ± 0 | 0 ± 0 | 0 ± 0 |
| Entoloma pulchellum | 0 ± 0 | 0 ± 0 | 0 ± 0 | 0 ± 0 | 0 ± 0 | 0 ± 0 | 0.67 ± 0.67 | 0 ± 0 | 0 ± 0 | 0 ± 0 |
| Entoloma sp. 2 AK-2012 | 0 ± 0 | 0 ± 0 | 0 ± 0 | 0 ± 0 | 0 ± 0 | 1 ± 1 | 0 ± 0 | 0 ± 0 | 0 ± 0 | 0 ± 0 |
| Entoloma undatum | 0 ± 0 | 0.67 ± 0.33 | 0 ± 0 | 1.67 ± 1.2 | 0 ± 0 | 0 ± 0 | 0.33 ± 0.33 | 0 ± 0 | 0 ± 0 | 0 ± 0 |
| Entorrhiza sp. HUV 21857 | 0 ± 0 | 54 ± 30.89 | 0 ± 0 | 2.67 ± 2.19 | 0 ± 0 | 0.33 ± 0.33 | 6.33 ± 6.33 | 0 ± 0 | 0 ± 0 | 0 ± 0 |
| Entosordaria quercina | 0 ± 0 | 0 ± 0 | 0 ± 0 | 0 ± 0 | 0 ± 0 | 1 ± 1 | 0 ± 0 | 0 ± 0 | 0 ± 0 | 0 ± 0 |
| Entrophospora infrequens | 24.67 ± 5.84 | 170.33 ± 62.03 | 36 ± 12.58 | 219.33 ± 46.84 | 53 ± 19.63 | 43.67 ± 8.67 | 126.33 ± 57.16 | 107.67 ± 36.83 | 58.67 ± 5.78 | 142.33 ± 48.34 |
| Entrophospora sp. | 0 ± 0 | 0 ± 0 | 1.67 ± 1.67 | 0 ± 0 | 0 ± 0 | 7.33 ± 6.36 | 1.33 ± 1.33 | 4.67 ± 4.18 | 9.67 ± 3.38 | 24.33 ± 12.44 |
| Entrophospora sp. JJ61 | 0.33 ± 0.33 | 0 ± 0 | 2.33 ± 2.33 | 0 ± 0 | 0 ± 0 | 5 ± 5 | 0.33 ± 0.33 | 1 ± 1 | 0 ± 0 | 5 ± 3.61 |
| Entrophospora sp. shylm120 | 0 ± 0 | 0 ± 0 | 0.33 ± 0.33 | 0 ± 0 | 0 ± 0 | 0 ± 0 | 0.33 ± 0.33 | 3.67 ± 2.03 | 8.67 ± 8.67 | 0.67 ± 0.67 |
| Eocronartium sp. I12F-02262 | 0.33 ± 0.33 | 0.67 ± 0.33 | 0 ± 0 | 5.67 ± 2.4 | 13 ± 3.61 | 0 ± 0 | 2 ± 2 | 89 ± 49.74 | 120 ± 119 | 0.67 ± 0.33 |
| Epibryon bryophilum | 0 ± 0 | 0.33 ± 0.33 | 0 ± 0 | 0 ± 0 | 0 ± 0 | 0 ± 0 | 0.33 ± 0.33 | 0.67 ± 0.67 | 3.33 ± 2.85 | 0 ± 0 |
| Epichloe pampeana | 0 ± 0 | 0 ± 0 | 0 ± 0 | 0 ± 0 | 0 ± 0 | 0 ± 0 | 0 ± 0 | 0 ± 0 | 0 ± 0 | 0 ± 0 |
| Epicoccum nigrum | 22.67 ± 13.25 | 27.67 ± 14.17 | 106.67 ± 27.49 | 24.33 ± 3.93 | 40.33 ± 2.6 | 43 ± 32.02 | 40.33 ± 12.25 | 84.67 ± 20.58 | 14.33 ± 2.96 | 211.33 ± 36.67 |
| Epicoccum plurivorum | 0.67 ± 0.67 | 22 ± 20.03 | 1 ± 0 | 1 ± 1 | 0.33 ± 0.33 | 1 ± 0.58 | 6.33 ± 6.33 | 0 ± 0 | 0 ± 0 | 0 ± 0 |
| Epicoccum sp. | 1 ± 0.58 | 110 ± 59.48 | 2 ± 2 | 5 ± 3.51 | 0.33 ± 0.33 | 1.33 ± 1.33 | 9 ± 9 | 0 ± 0 | 0 ± 0 | 0 ± 0 |
| Epicoleosporium ramularioides | 0 ± 0 | 0 ± 0 | 81 ± 23.12 | 1.33 ± 1.33 | 0 ± 0 | 20.33 ± 16.9 | 10.33 ± 5.84 | 0 ± 0 | 0 ± 0 | 2.33 ± 1.86 |
| Erythrobasidium hasegawianum | 0 ± 0 | 0 ± 0 | 0 ± 0 | 0 ± 0 | 0 ± 0 | 0 ± 0 | 0 ± 0 | 0 ± 0 | 0 ± 0 | 1.33 ± 1.33 |
| Erythrobasidium sp. FF-2011 | 0 ± 0 | 0 ± 0 | 0 ± 0 | 0 ± 0 | 0.33 ± 0.33 | 0 ± 0 | 0 ± 0 | 1 ± 1 | 0 ± 0 | 2.33 ± 1.2 |
| Erythrobasidium yunnanense | 0 ± 0 | 0 ± 0 | 0.33 ± 0.33 | 0 ± 0 | 0 ± 0 | 0.67 ± 0.67 | 1 ± 1 | 1.67 ± 0.88 | 0 ± 0 | 0 ± 0 |
| Escovopsioides nivea | 2.67 ± 2.67 | 0 ± 0 | 0.33 ± 0.33 | 0 ± 0 | 1 ± 0.58 | 0 ± 0 | 0.33 ± 0.33 | 0 ± 0 | 0 ± 0 | 0 ± 0 |
| Escovopsioides sp. | 2 ± 1.15 | 0.33 ± 0.33 | 0 ± 0 | 0 ± 0 | 0 ± 0 | 0 ± 0 | 0 ± 0 | 0 ± 0 | 0 ± 0 | 0 ± 0 |
| Escovopsis weberi | 0 ± 0 | 0 ± 0 | 0 ± 0 | 0 ± 0 | 0.67 ± 0.67 | 2.33 ± 1.86 | 0 ± 0 | 0 ± 0 | 0 ± 0 | 0 ± 0 |
| Eucasphaeria capensis | 0 ± 0 | 0 ± 0 | 0 ± 0 | 0.67 ± 0.33 | 0 ± 0 | 0 ± 0 | 2 ± 1.15 | 2 ± 2 | 0.33 ± 0.33 | 5.33 ± 4.84 |
| Eutypella scoparia | 0 ± 0 | 0 ± 0 | 1.33 ± 0.88 | 0.67 ± 0.33 | 0.33 ± 0.33 | 8 ± 4.16 | 2 ± 1.53 | 0 ± 0 | 0 ± 0 | 1.67 ± 0.88 |
| Exidia candida | 0 ± 0 | 0 ± 0 | 0 ± 0 | 0 ± 0 | 0 ± 0 | 0 ± 0 | 0 ± 0 | 0 ± 0 | 0 ± 0 | 0 ± 0 |
| Exidia glandulosa | 0 ± 0 | 2.33 ± 1.2 | 0 ± 0 | 0 ± 0 | 1.33 ± 1.33 | 0 ± 0 | 0.67 ± 0.33 | 0 ± 0 | 0.33 ± 0.33 | 3 ± 3 |
| Exobasidium pulchrum | 0 ± 0 | 0 ± 0 | 0 ± 0 | 0 ± 0 | 0 ± 0 | 0 ± 0 | 0 ± 0 | 0.67 ± 0.67 | 0 ± 0 | 0 ± 0 |
| Exophiala angulospora | 0 ± 0 | 0 ± 0 | 1 ± 1 | 0 ± 0 | 0 ± 0 | 0.67 ± 0.67 | 0 ± 0 | 0 ± 0 | 2 ± 2 | 0 ± 0 |
| Exophiala aquamarina | 2.33 ± 2.33 | 0 ± 0 | 0 ± 0 | 0 ± 0 | 0 ± 0 | 0 ± 0 | 0 ± 0 | 0 ± 0 | 0 ± 0 | 1 ± 1 |
| Exophiala attenuata | 0 ± 0 | 0 ± 0 | 2 ± 1.53 | 0 ± 0 | 0 ± 0 | 0.33 ± 0.33 | 1.67 ± 1.67 | 0 ± 0 | 0 ± 0 | 0 ± 0 |
| Exophiala brunnea | 0 ± 0 | 0 ± 0 | 5 ± 3.21 | 0.33 ± 0.33 | 0 ± 0 | 0 ± 0 | 0 ± 0 | 0 ± 0 | 0 ± 0 | 0.67 ± 0.67 |
| Exophiala cancerae | 5 ± 2.65 | 7 ± 6.51 | 1 ± 1 | 0 ± 0 | 0 ± 0 | 0 ± 0 | 0.67 ± 0.67 | 8.33 ± 4.18 | 26 ± 26 | 0.67 ± 0.33 |
| Exophiala equina | 38 ± 2.08 | 44 ± 10.02 | 200.67 ± 128.17 | 24.67 ± 13.54 | 71.67 ± 39.1 | 66.67 ± 26.35 | 47 ± 10.6 | 318.67 ± 152.66 | 134.67 ± 113.7 | 42 ± 13.87 |
| Exophiala jeanselmei | 0 ± 0 | 0 ± 0 | 0 ± 0 | 0 ± 0 | 0 ± 0 | 0 ± 0 | 0 ± 0 | 0.33 ± 0.33 | 0.67 ± 0.67 | 0 ± 0 |
| Exophiala lecanii-corni | 0 ± 0 | 0 ± 0 | 0 ± 0 | 0.33 ± 0.33 | 0 ± 0 | 8.33 ± 6.01 | 0 ± 0 | 0 ± 0 | 0 ± 0 | 0.33 ± 0.33 |
| Exophiala mesophila | 0 ± 0 | 0 ± 0 | 0 ± 0 | 0 ± 0 | 0 ± 0 | 0 ± 0 | 0 ± 0 | 0 ± 0 | 0 ± 0 | 0.67 ± 0.67 |
| Exophiala salmonis | 0 ± 0 | 3 ± 3 | 0 ± 0 | 0 ± 0 | 0 ± 0 | 0 ± 0 | 0.33 ± 0.33 | 0 ± 0 | 0 ± 0 | 0 ± 0 |
| Exophiala sp. | 1.67 ± 1.2 | 4.67 ± 0.88 | 144.67 ± 45.06 | 30 ± 17.67 | 1 ± 0 | 60.67 ± 30.02 | 70.33 ± 31.14 | 146.67 ± 72 | 69.33 ± 66.34 | 6 ± 3 |
| Exophiala sp. 2 TS-2016 | 0 ± 0 | 0.33 ± 0.33 | 0.67 ± 0.67 | 1.33 ± 0.33 | 0.33 ± 0.33 | 51 ± 23.52 | 6 ± 2.65 | 11.67 ± 5.9 | 0 ± 0 | 0.67 ± 0.67 |
| Exophiala sp. 4-11c | 0 ± 0 | 0 ± 0 | 0 ± 0 | 0 ± 0 | 0 ± 0 | 0 ± 0 | 0 ± 0 | 0 ± 0 | 0 ± 0 | 0 ± 0 |
| Exophiala sp. ATT135 | 0 ± 0 | 0 ± 0 | 0.33 ± 0.33 | 0 ± 0 | 0 ± 0 | 0.67 ± 0.67 | 0 ± 0 | 0 ± 0 | 0 ± 0 | 0 ± 0 |
| Exophiala sp. CPC 12171 | 0 ± 0 | 0 ± 0 | 0 ± 0 | 0 ± 0 | 0 ± 0 | 0 ± 0 | 0 ± 0 | 0.67 ± 0.67 | 0 ± 0 | 0 ± 0 |
| Exophiala sp. NH512 | 0 ± 0 | 0.33 ± 0.33 | 0 ± 0 | 0 ± 0 | 0 ± 0 | 0 ± 0 | 0 ± 0 | 0 ± 0 | 0 ± 0 | 0 ± 0 |
| Exophiala sp. Ppf18 | 1.67 ± 0.67 | 17 ± 11.59 | 3 ± 1.73 | 1.33 ± 0.88 | 0 ± 0 | 0 ± 0 | 8 ± 5.69 | 648 ± 567.57 | 599.33 ± 595.33 | 2 ± 1.15 |
| Exophiala xenobiotica | 0 ± 0 | 0 ± 0 | 0 ± 0 | 0 ± 0 | 0 ± 0 | 0 ± 0 | 0 ± 0 | 0 ± 0 | 0.67 ± 0.67 | 0 ± 0 |
| Farysia acheniorum | 0 ± 0 | 0 ± 0 | 0 ± 0 | 0 ± 0 | 0 ± 0 | 0 ± 0 | 0 ± 0 | 1.67 ± 1.67 | 0.33 ± 0.33 | 0 ± 0 |
| Fellozyma inositophila | 0 ± 0 | 0 ± 0 | 0.33 ± 0.33 | 0 ± 0 | 0 ± 0 | 0.67 ± 0.67 | 0 ± 0 | 0 ± 0 | 0 ± 0 | 0 ± 0 |
| Fibroporia vaillantii | 0 ± 0 | 0 ± 0 | 0 ± 0 | 1 ± 0.58 | 0.33 ± 0.33 | 0 ± 0 | 1.67 ± 1.2 | 0 ± 0 | 0 ± 0 | 1.67 ± 1.67 |
| Fibulobasidium murrhardtense | 0.33 ± 0.33 | 0.33 ± 0.33 | 0 ± 0 | 0 ± 0 | 0 ± 0 | 0.67 ± 0.67 | 0 ± 0 | 0 ± 0 | 0 ± 0 | 0 ± 0 |
| Fibulochlamys chilensis | 0 ± 0 | 0.67 ± 0.67 | 0 ± 0 | 0.67 ± 0.67 | 0 ± 0 | 0 ± 0 | 0 ± 0 | 0 ± 0 | 1 ± 0.58 | 0 ± 0 |
| Fibulomyces fusoideus | 0 ± 0 | 0 ± 0 | 0 ± 0 | 0 ± 0 | 0 ± 0 | 0 ± 0 | 0 ± 0 | 0.33 ± 0.33 | 1 ± 0 | 23 ± 19.04 |
| Filobasidium magnum | 0.67 ± 0.33 | 0 ± 0 | 0.67 ± 0.67 | 0.33 ± 0.33 | 0 ± 0 | 4.33 ± 2.33 | 0.67 ± 0.33 | 0.33 ± 0.33 | 1.33 ± 0.88 | 1.33 ± 1.33 |
| Filobasidium wieringae | 0 ± 0 | 0 ± 0 | 0 ± 0 | 0 ± 0 | 0 ± 0 | 1 ± 1 | 0 ± 0 | 0 ± 0 | 0 ± 0 | 0 ± 0 |
| Fimetariella rabenhorstii | 0 ± 0 | 0 ± 0 | 3.67 ± 3.67 | 0 ± 0 | 0 ± 0 | 0 ± 0 | 1.33 ± 0.88 | 0 ± 0 | 0 ± 0 | 0 ± 0 |
| Flabellascoma cycadicola | 0 ± 0 | 0 ± 0 | 0 ± 0 | 0.33 ± 0.33 | 0 ± 0 | 0.33 ± 0.33 | 3 ± 1.73 | 5.67 ± 5.67 | 0.33 ± 0.33 | 1 ± 1 |
| Flagellospora curvula | 1.67 ± 1.67 | 0 ± 0 | 0 ± 0 | 0 ± 0 | 0 ± 0 | 0 ± 0 | 0 ± 0 | 0 ± 0 | 0 ± 0 | 0 ± 0 |
| Fonsecazyma tronadorensis | 0 ± 0 | 0 ± 0 | 0 ± 0 | 0 ± 0 | 0 ± 0 | 0 ± 0 | 0 ± 0 | 0 ± 0 | 0 ± 0 | 0 ± 0 |
| Funneliformis caledonium | 0.67 ± 0.33 | 0 ± 0 | 0 ± 0 | 0 ± 0 | 1 ± 1 | 0 ± 0 | 0 ± 0 | 0 ± 0 | 0 ± 0 | 0 ± 0 |
| Funneliformis mosseae | 1 ± 0.58 | 27.33 ± 3.84 | 0 ± 0 | 0.33 ± 0.33 | 0.67 ± 0.67 | 0 ± 0 | 2.67 ± 1.45 | 0 ± 0 | 0 ± 0 | 1 ± 1 |
| Fusariella hughesii | 0 ± 0 | 0 ± 0 | 7 ± 2 | 0 ± 0 | 0 ± 0 | 3.33 ± 2.03 | 0.33 ± 0.33 | 2.33 ± 2.33 | 0.33 ± 0.33 | 1 ± 1 |
| Fusarium albidum | 0.33 ± 0.33 | 0 ± 0 | 0 ± 0 | 0 ± 0 | 5 ± 3.06 | 0.33 ± 0.33 | 0.33 ± 0.33 | 0 ± 0 | 0 ± 0 | 0 ± 0 |
| Fusarium brachygibbosum | 0 ± 0 | 0 ± 0 | 0 ± 0 | 0.33 ± 0.33 | 0 ± 0 | 5.67 ± 5.67 | 0 ± 0 | 0 ± 0 | 0 ± 0 | 4.67 ± 4.67 |
| Fusarium cf. solani | 45.67 ± 20.27 | 84.67 ± 47.75 | 55 ± 18.9 | 27 ± 17.01 | 21.33 ± 3.93 | 161.33 ± 20.67 | 120 ± 23.64 | 84.67 ± 53.41 | 53.67 ± 50.67 | 66.67 ± 25.83 |
| Fusarium ciliatum | 0 ± 0 | 0 ± 0 | 11.67 ± 3.84 | 0 ± 0 | 0 ± 0 | 3 ± 1.73 | 4.33 ± 2.4 | 675.67 ± 641.95 | 26.67 ± 23.67 | 4.33 ± 2.6 |
| Fusarium culmorum | 0 ± 0 | 0 ± 0 | 0 ± 0 | 0 ± 0 | 0.67 ± 0.33 | 1 ± 1 | 1.33 ± 0.88 | 0 ± 0 | 0 ± 0 | 0 ± 0 |
| Fusarium cuneirostrum | 0.33 ± 0.33 | 0 ± 0 | 0 ± 0 | 0 ± 0 | 0 ± 0 | 0.33 ± 0.33 | 0.67 ± 0.33 | 0 ± 0 | 0 ± 0 | 0 ± 0 |
| Fusarium decemcellulare | 0 ± 0 | 0 ± 0 | 1 ± 1 | 3 ± 2 | 0 ± 0 | 11.33 ± 6.74 | 3.67 ± 2.19 | 0 ± 0 | 0 ± 0 | 1.33 ± 0.88 |
| Fusarium fujikuroi | 1.67 ± 0.67 | 6 ± 0.58 | 7.67 ± 3.53 | 6 ± 2.31 | 9.67 ± 3.84 | 96 ± 50.77 | 36.67 ± 15.39 | 1.33 ± 1.33 | 0.33 ± 0.33 | 3 ± 1.53 |
| Fusarium graminearum | 6.67 ± 3.48 | 34.67 ± 33.67 | 101 ± 50.36 | 5.33 ± 3.93 | 74.33 ± 66.42 | 51.67 ± 48.68 | 12 ± 5.86 | 0.33 ± 0.33 | 9.33 ± 8.84 | 6.67 ± 0.33 |
| Fusarium illudens | 0 ± 0 | 0 ± 0 | 1 ± 1 | 0 ± 0 | 0 ± 0 | 5 ± 2.65 | 0 ± 0 | 0 ± 0 | 0 ± 0 | 0 ± 0 |
| Fusarium incarnatum | 4 ± 1.53 | 18.67 ± 12.17 | 17.67 ± 10.17 | 7 ± 6.51 | 12.67 ± 7.17 | 22.33 ± 10.59 | 28.33 ± 13.35 | 2 ± 1 | 1.67 ± 1.2 | 29.67 ± 11.46 |
| Fusarium lichenicola | 0.33 ± 0.33 | 0 ± 0 | 1 ± 1 | 0 ± 0 | 0.67 ± 0.33 | 1 ± 0.58 | 0 ± 0 | 0 ± 0 | 0 ± 0 | 0 ± 0 |
| Fusarium nematophilum | 39.33 ± 4.41 | 0.67 ± 0.33 | 19.33 ± 8.82 | 0.33 ± 0.33 | 66.67 ± 37.88 | 165.67 ± 113.52 | 10.33 ± 5.36 | 3 ± 3 | 0 ± 0 | 1 ± 1 |
| Fusarium oxysporum | 9.33 ± 3.18 | 46 ± 18.19 | 2.67 ± 1.45 | 18 ± 9.45 | 8 ± 2.31 | 45.67 ± 22.81 | 37 ± 6.66 | 6 ± 1 | 1.67 ± 0.33 | 17 ± 3.21 |
| Fusarium phaseoli | 0 ± 0 | 0 ± 0 | 0 ± 0 | 0 ± 0 | 0 ± 0 | 0.67 ± 0.67 | 0 ± 0 | 0 ± 0 | 0 ± 0 | 0 ± 0 |
| Fusarium polyphialidicum | 1.33 ± 0.88 | 2 ± 0.58 | 2 ± 1.53 | 0.67 ± 0.33 | 3.33 ± 1.45 | 2.67 ± 1.67 | 4 ± 2.08 | 0 ± 0 | 0.67 ± 0.33 | 7.33 ± 3.38 |
| Fusarium sambucinum | 0 ± 0 | 0.67 ± 0.33 | 22.67 ± 5.84 | 7.33 ± 4.67 | 6.33 ± 2.33 | 15.67 ± 5.7 | 21.33 ± 10.91 | 0 ± 0 | 0 ± 0 | 8.67 ± 5.55 |
| Fusarium sarcochroum | 0 ± 0 | 0.33 ± 0.33 | 0.67 ± 0.67 | 0.33 ± 0.33 | 1.33 ± 1.33 | 1 ± 0.58 | 1 ± 1 | 0 ± 0 | 0 ± 0 | 15.33 ± 6.74 |
| Fusarium solani | 50.33 ± 9.13 | 34.33 ± 21.53 | 161 ± 12.5 | 28 ± 7.02 | 25.67 ± 9.21 | 276.33 ± 86.64 | 82.33 ± 27.09 | 427.33 ± 221.77 | 140.67 ± 136.17 | 45.33 ± 12.41 |
| Fusarium sp. | 324.67 ± 66.99 | 6865 ± 1538.04 | 218.67 ± 95.06 | 743.67 ± 225.47 | 193.67 ± 22.06 | 2723.67 ± 1210.46 | 2154 ± 832.26 | 544.33 ± 238.85 | 332 ± 99.53 | 2719.33 ± 610.5 |
| Fusarium sp. 14012 | 0 ± 0 | 0 ± 0 | 0 ± 0 | 0 ± 0 | 0.33 ± 0.33 | 0.33 ± 0.33 | 1.67 ± 0.88 | 1 ± 1 | 0 ± 0 | 0 ± 0 |
| Fusarium sp. CF13 | 0 ± 0 | 0 ± 0 | 0 ± 0 | 1.67 ± 0.88 | 1.33 ± 1.33 | 0 ± 0 | 1.67 ± 1.2 | 0 ± 0 | 0 ± 0 | 6 ± 4.58 |
| Fusarium sp. F-ZH | 0 ± 0 | 0.33 ± 0.33 | 0 ± 0 | 3.33 ± 1.33 | 0.33 ± 0.33 | 20.33 ± 11.84 | 3 ± 1.73 | 0 ± 0 | 0 ± 0 | 5.67 ± 2.19 |
| Fusarium sp. JCM 28004 | 0 ± 0 | 0 ± 0 | 0.67 ± 0.67 | 0 ± 0 | 0 ± 0 | 0.33 ± 0.33 | 0.33 ± 0.33 | 0 ± 0 | 0 ± 0 | 0 ± 0 |
| Fusarium sp. SS-R6 | 7.67 ± 5.36 | 0 ± 0 | 2.33 ± 2.33 | 0 ± 0 | 1.67 ± 0.67 | 23 ± 10.39 | 3.33 ± 1.86 | 4 ± 4 | 4 ± 4 | 0 ± 0 |
| Fusarium sublunatum | 0 ± 0 | 0.33 ± 0.33 | 0.33 ± 0.33 | 0.33 ± 0.33 | 1 ± 0.58 | 8.67 ± 7.22 | 2.67 ± 1.45 | 0 ± 0 | 0 ± 0 | 1.33 ± 1.33 |
| Fusarium verticillioides | 0 ± 0 | 0 ± 0 | 0 ± 0 | 0 ± 0 | 0 ± 0 | 0 ± 0 | 0 ± 0 | 0 ± 0 | 0 ± 0 | 0 ± 0 |
| Fusicolla acetilerea | 0 ± 0 | 0.33 ± 0.33 | 3.67 ± 3.67 | 0 ± 0 | 0 ± 0 | 15.33 ± 2.85 | 1.33 ± 0.88 | 0 ± 0 | 0 ± 0 | 0.33 ± 0.33 |
| Fusicolla aquaeductuum | 0 ± 0 | 0 ± 0 | 0 ± 0 | 1 ± 1 | 0 ± 0 | 0.33 ± 0.33 | 0.67 ± 0.67 | 0 ± 0 | 0 ± 0 | 0 ± 0 |
| Fusicolla melogrammae | 0 ± 0 | 0 ± 0 | 2.67 ± 2.67 | 0 ± 0 | 0 ± 0 | 1 ± 1 | 0 ± 0 | 14.33 ± 7.26 | 1 ± 1 | 0.33 ± 0.33 |
| Fusicolla ossicola | 3 ± 1.53 | 0.33 ± 0.33 | 56.33 ± 13.28 | 2.67 ± 0.33 | 8.33 ± 1.2 | 240 ± 122.46 | 8.67 ± 2.91 | 4 ± 2.08 | 0.33 ± 0.33 | 6.33 ± 4.1 |
| Fusicolla sp. SKS-2018a | 0 ± 0 | 0 ± 0 | 1.67 ± 0.88 | 0 ± 0 | 0 ± 0 | 0.67 ± 0.67 | 0 ± 0 | 0 ± 0 | 0 ± 0 | 0.33 ± 0.33 |
| Fusiconidium lycopodiellae | 0 ± 0 | 0 ± 0 | 0 ± 0 | 0 ± 0 | 0 ± 0 | 0 ± 0 | 0 ± 0 | 0 ± 0 | 0 ± 0 | 3.67 ± 0.67 |
| Fusidium griseum | 6.33 ± 2.33 | 15.33 ± 11.57 | 33 ± 21.01 | 5.33 ± 3.84 | 14.67 ± 3.28 | 304.33 ± 143.91 | 14.33 ± 4.41 | 98 ± 73.98 | 38.33 ± 37.34 | 34.67 ± 12.14 |
| Gaeumannomycella caricis | 0 ± 0 | 0 ± 0 | 0.33 ± 0.33 | 0 ± 0 | 0 ± 0 | 0 ± 0 | 0.33 ± 0.33 | 0 ± 0 | 0 ± 0 | 0 ± 0 |
| Gaeumannomyces tritici | 0 ± 0 | 0 ± 0 | 0 ± 0 | 0 ± 0 | 0 ± 0 | 0 ± 0 | 0.33 ± 0.33 | 0 ± 0 | 0 ± 0 | 0 ± 0 |
| Gamsia columbina | 0.33 ± 0.33 | 0 ± 0 | 0.33 ± 0.33 | 0.33 ± 0.33 | 0.33 ± 0.33 | 1 ± 0.58 | 4.67 ± 4.18 | 0.33 ± 0.33 | 1 ± 0.58 | 1.33 ± 0.88 |
| Gamsylella phymatopaga | 0 ± 0 | 0 ± 0 | 0 ± 0 | 0 ± 0 | 0 ± 0 | 0.67 ± 0.67 | 0 ± 0 | 0 ± 0 | 0 ± 0 | 0 ± 0 |
| Ganoderma gibbosum | 1.33 ± 0.33 | 0.33 ± 0.33 | 18.67 ± 4.81 | 10.33 ± 5.36 | 6.67 ± 1.86 | 11.33 ± 3.48 | 21.33 ± 8.82 | 32.67 ± 14.88 | 14 ± 11.53 | 2 ± 1.53 |
| Ganoderma lucidum | 0 ± 0 | 0 ± 0 | 1 ± 1 | 1 ± 1 | 0 ± 0 | 0.67 ± 0.33 | 10.33 ± 7.88 | 1.67 ± 1.67 | 0.33 ± 0.33 | 2.67 ± 2.67 |
| Ganoderma sp. BJ17 | 38 ± 6.24 | 0.33 ± 0.33 | 6.67 ± 5.7 | 0 ± 0 | 10.33 ± 4.18 | 1 ± 1 | 1.33 ± 0.67 | 0 ± 0 | 0 ± 0 | 0 ± 0 |
| Geastrumia polystigmatis | 0 ± 0 | 0 ± 0 | 0 ± 0 | 0 ± 0 | 0 ± 0 | 0 ± 0 | 0 ± 0 | 0 ± 0 | 0 ± 0 | 1 ± 1 |
| Gelasinospora heterospora | 0.67 ± 0.67 | 0 ± 0 | 0 ± 0 | 0 ± 0 | 0 ± 0 | 0 ± 0 | 0 ± 0 | 0 ± 0 | 0 ± 0 | 0 ± 0 |
| Geminibasidium donsium | 0.67 ± 0.67 | 4.33 ± 1.2 | 765.67 ± 183.51 | 3.67 ± 0.88 | 18 ± 6.51 | 270.33 ± 201.88 | 10 ± 4.16 | 0 ± 0 | 0 ± 0 | 7.33 ± 3.53 |
| Genolevuria amylolytica | 0 ± 0 | 0 ± 0 | 0 ± 0 | 1 ± 1 | 0 ± 0 | 0 ± 0 | 0 ± 0 | 0 ± 0 | 0 ± 0 | 0.33 ± 0.33 |
| Genolevuria bromeliarum | 0 ± 0 | 0 ± 0 | 0 ± 0 | 0 ± 0 | 0 ± 0 | 0 ± 0 | 0 ± 0 | 0 ± 0 | 0 ± 0 | 0 ± 0 |
| Geoglossum dunense | 0 ± 0 | 0 ± 0 | 0 ± 0 | 0 ± 0 | 0 ± 0 | 0 ± 0 | 0 ± 0 | 6 ± 6 | 4.33 ± 3.38 | 0 ± 0 |
| Geoglossum fallax | 0 ± 0 | 0 ± 0 | 0 ± 0 | 0 ± 0 | 0 ± 0 | 0 ± 0 | 30.33 ± 30.33 | 0 ± 0 | 1 ± 0.58 | 0 ± 0 |
| Geoglossum umbratile | 0 ± 0 | 0 ± 0 | 4 ± 4 | 0.33 ± 0.33 | 0 ± 0 | 0 ± 0 | 0.67 ± 0.67 | 0 ± 0 | 0 ± 0 | 0 ± 0 |
| Geoglossum variabilisporum | 0 ± 0 | 0 ± 0 | 0 ± 0 | 0 ± 0 | 0.67 ± 0.67 | 0 ± 0 | 0 ± 0 | 0 ± 0 | 0 ± 0 | 0 ± 0 |
| Geoglossum vleugelianum | 24 ± 19.08 | 0.33 ± 0.33 | 0 ± 0 | 0 ± 0 | 2 ± 1.15 | 0 ± 0 | 0 ± 0 | 4 ± 4 | 0 ± 0 | 0 ± 0 |
| Gibellulopsis nigrescens | 1.67 ± 0.67 | 38.67 ± 24.46 | 13.33 ± 11.33 | 12.67 ± 5.67 | 12.67 ± 3.48 | 80 ± 39.37 | 19 ± 4.36 | 2.67 ± 1.76 | 7.33 ± 7.33 | 72 ± 40.81 |
| Gliocladiopsis sp. | 0 ± 0 | 0 ± 0 | 25 ± 9.71 | 0 ± 0 | 0.33 ± 0.33 | 13.67 ± 12.2 | 0.33 ± 0.33 | 0 ± 0 | 0 ± 0 | 0 ± 0 |
| Gliocladiopsis tenuis | 0 ± 0 | 0 ± 0 | 0 ± 0 | 0 ± 0 | 0 ± 0 | 0 ± 0 | 2.67 ± 2.67 | 0 ± 0 | 0 ± 0 | 0 ± 0 |
| Gliocladium cibotii | 0 ± 0 | 0 ± 0 | 1 ± 1 | 0.67 ± 0.33 | 0 ± 0 | 4.67 ± 0.88 | 2.33 ± 0.67 | 0 ± 0 | 0 ± 0 | 0.67 ± 0.67 |
| Gliomastix masseei | 0.33 ± 0.33 | 0 ± 0 | 0 ± 0 | 0.67 ± 0.67 | 0 ± 0 | 15 ± 9.29 | 0 ± 0 | 0 ± 0 | 0 ± 0 | 0 ± 0 |
| Gliomastix murorum | 1 ± 0.58 | 0 ± 0 | 13.33 ± 5.81 | 1.33 ± 0.33 | 14.33 ± 2.19 | 5.33 ± 2.33 | 20 ± 17.5 | 43 ± 9.85 | 7.33 ± 3.18 | 10.67 ± 3.67 |
| Gloeoporus dichrous | 0 ± 0 | 0 ± 0 | 0 ± 0 | 0 ± 0 | 0 ± 0 | 0 ± 0 | 0 ± 0 | 0 ± 0 | 0 ± 0 | 0 ± 0 |
| Gloeoporus orientalis | 0 ± 0 | 0 ± 0 | 0 ± 0 | 0 ± 0 | 0 ± 0 | 0 ± 0 | 0.67 ± 0.67 | 0 ± 0 | 0 ± 0 | 0 ± 0 |
| Gloeosporium orbiculare | 0 ± 0 | 0 ± 0 | 0 ± 0 | 1 ± 1 | 0 ± 0 | 0.33 ± 0.33 | 0 ± 0 | 0 ± 0 | 0 ± 0 | 0 ± 0 |
| Glomerella sp. CS64-1 | 0 ± 0 | 0 ± 0 | 1 ± 1 | 0 ± 0 | 0 ± 0 | 1.33 ± 0.88 | 0.33 ± 0.33 | 0 ± 0 | 0.67 ± 0.67 | 1 ± 1 |
| Glomus aggregatum | 0.67 ± 0.33 | 0 ± 0 | 0 ± 0 | 0 ± 0 | 6.33 ± 3.38 | 0 ± 0 | 0 ± 0 | 0 ± 0 | 0 ± 0 | 0 ± 0 |
| Glomus sp. | 1.33 ± 0.88 | 0 ± 0 | 0.33 ± 0.33 | 8.33 ± 5.04 | 0 ± 0 | 0.67 ± 0.67 | 1 ± 1 | 1 ± 0.58 | 0.67 ± 0.33 | 25.67 ± 10.84 |
| Glomus sp. 0151 | 0 ± 0 | 0.33 ± 0.33 | 0 ± 0 | 0.67 ± 0.67 | 0 ± 0 | 0 ± 0 | 0 ± 0 | 0 ± 0 | 0 ± 0 | 0 ± 0 |
| Glomus sp. 027 | 0 ± 0 | 0.67 ± 0.67 | 0 ± 0 | 0 ± 0 | 0 ± 0 | 0 ± 0 | 0 ± 0 | 0 ± 0 | 0 ± 0 | 0 ± 0 |
| Glomus sp. 0504 | 0.67 ± 0.33 | 0 ± 0 | 0 ± 0 | 0 ± 0 | 4.67 ± 1.2 | 0 ± 0 | 2.33 ± 2.33 | 0 ± 0 | 0 ± 0 | 0 ± 0 |
| Glomus sp. 0512 | 0 ± 0 | 0 ± 0 | 0 ± 0 | 0 ± 0 | 3.33 ± 1.86 | 0 ± 0 | 0.33 ± 0.33 | 0 ± 0 | 0 ± 0 | 0 ± 0 |
| Glomus sp. 0523 | 0 ± 0 | 0 ± 0 | 0 ± 0 | 1.33 ± 1.33 | 1.33 ± 0.33 | 1 ± 0.58 | 0 ± 0 | 5.33 ± 2.19 | 4 ± 2.52 | 15 ± 6.43 |
| Glomus sp. 1 SL-2017 | 0 ± 0 | 0 ± 0 | 0 ± 0 | 0.33 ± 0.33 | 8.33 ± 3.76 | 0 ± 0 | 0.67 ± 0.67 | 0 ± 0 | 0 ± 0 | 0 ± 0 |
| Glomus sp. 11 ZHNL-2013f | 0.67 ± 0.33 | 0 ± 0 | 0 ± 0 | 0 ± 0 | 0 ± 0 | 0.67 ± 0.67 | 0 ± 0 | 0 ± 0 | 0 ± 0 | 0 ± 0 |
| Glomus sp. 11 ZHNL-2013l | 5 ± 3.51 | 0 ± 0 | 0 ± 0 | 0 ± 0 | 0.33 ± 0.33 | 1 ± 1 | 0 ± 0 | 0 ± 0 | 0 ± 0 | 0 ± 0 |
| Glomus sp. 11 ZHNL-2013o | 0 ± 0 | 0.33 ± 0.33 | 0 ± 0 | 15.33 ± 3.84 | 0.33 ± 0.33 | 0.67 ± 0.67 | 3 ± 2.52 | 0 ± 0 | 0 ± 0 | 0 ± 0 |
| Glomus sp. 11 ZHNL-2013r | 6.33 ± 1.33 | 0 ± 0 | 0 ± 0 | 1.67 ± 1.2 | 1 ± 0.58 | 0.33 ± 0.33 | 0.67 ± 0.67 | 4 ± 2 | 1 ± 0.58 | 0.33 ± 0.33 |
| Glomus sp. 16 SUN-2011 | 0 ± 0 | 0 ± 0 | 0 ± 0 | 0 ± 0 | 0.33 ± 0.33 | 0.33 ± 0.33 | 0.33 ± 0.33 | 0 ± 0 | 0 ± 0 | 0 ± 0 |
| Glomus sp. 17 SUN-2011 | 0 ± 0 | 2.67 ± 1.45 | 0 ± 0 | 0 ± 0 | 0 ± 0 | 0 ± 0 | 0 ± 0 | 0.33 ± 0.33 | 0 ± 0 | 0 ± 0 |
| Glomus sp. 1a4.2-2 | 0 ± 0 | 104.33 ± 45.62 | 0 ± 0 | 3.67 ± 0.67 | 1 ± 1 | 0 ± 0 | 22.33 ± 13.96 | 0 ± 0 | 0 ± 0 | 0 ± 0 |
| Glomus sp. 2 SL-2017 | 0.33 ± 0.33 | 27 ± 5.29 | 0 ± 0 | 11.33 ± 4.91 | 0 ± 0 | 0 ± 0 | 2.33 ± 0.88 | 9 ± 9 | 29 ± 15.72 | 4 ± 1.15 |
| Glomus sp. 26 ZHNL-2013b | 0 ± 0 | 0.33 ± 0.33 | 0 ± 0 | 0.67 ± 0.67 | 0 ± 0 | 0.67 ± 0.67 | 0.67 ± 0.67 | 0 ± 0 | 0 ± 0 | 0 ± 0 |
| Glomus sp. 6 SUN-2011 | 0 ± 0 | 0 ± 0 | 0 ± 0 | 0 ± 0 | 0 ± 0 | 0 ± 0 | 0 ± 0 | 0 ± 0 | 0 ± 0 | 17.67 ± 9.17 |
| Glomus sp. 6 ZHNL-2013b | 1.33 ± 0.88 | 0 ± 0 | 0 ± 0 | 0 ± 0 | 0 ± 0 | 0.33 ± 0.33 | 0 ± 0 | 0 ± 0 | 0 ± 0 | 0 ± 0 |
| Glomus sp. 6 ZHNL-2013c | 0 ± 0 | 0 ± 0 | 0 ± 0 | 0 ± 0 | 1 ± 0.58 | 0 ± 0 | 0.33 ± 0.33 | 0 ± 0 | 0 ± 0 | 0 ± 0 |
| Glomus sp. 6 ZHNL-2013d | 0 ± 0 | 5.33 ± 2.19 | 0 ± 0 | 0 ± 0 | 2.33 ± 1.2 | 0 ± 0 | 0.33 ± 0.33 | 0 ± 0 | 0.33 ± 0.33 | 0.67 ± 0.67 |
| Glomus sp. 7 SUN-2011 | 0 ± 0 | 0 ± 0 | 0 ± 0 | 0 ± 0 | 0 ± 0 | 0 ± 0 | 0 ± 0 | 0.33 ± 0.33 | 0 ± 0 | 0 ± 0 |
| Glomus sp. 7 ZHNL-2013c | 0 ± 0 | 0 ± 0 | 0 ± 0 | 0 ± 0 | 0 ± 0 | 0 ± 0 | 0 ± 0 | 2 ± 1.15 | 0 ± 0 | 0 ± 0 |
| Glomus sp. 7 ZHNL-2013d | 1 ± 0.58 | 0 ± 0 | 0 ± 0 | 0 ± 0 | 9.67 ± 7.22 | 0 ± 0 | 0 ± 0 | 0 ± 0 | 0 ± 0 | 0 ± 0 |
| Glomus sp. 8 SUN-2011 | 0 ± 0 | 0 ± 0 | 0 ± 0 | 0 ± 0 | 0 ± 0 | 0 ± 0 | 0 ± 0 | 4.33 ± 4.33 | 1.33 ± 0.88 | 0 ± 0 |
| Glomus sp. 8 ZHNL-2013i | 0.33 ± 0.33 | 0 ± 0 | 0 ± 0 | 0 ± 0 | 0 ± 0 | 0 ± 0 | 0 ± 0 | 0 ± 0 | 0 ± 0 | 0 ± 0 |
| Glomus sp. 8 ZHNL-2013k | 0 ± 0 | 0 ± 0 | 0 ± 0 | 0.33 ± 0.33 | 0.33 ± 0.33 | 1.33 ± 0.88 | 0 ± 0 | 0.67 ± 0.67 | 0 ± 0 | 0 ± 0 |
| Glomus sp. 9 SUN-2011 | 0.67 ± 0.33 | 2.67 ± 0.33 | 0.33 ± 0.33 | 2.33 ± 0.33 | 14.67 ± 4.06 | 1.67 ± 1.67 | 3.33 ± 1.2 | 6.33 ± 2.19 | 7.67 ± 2.4 | 4 ± 0.58 |
| Glomus sp. Bad Sachsa | 0.67 ± 0.67 | 0 ± 0 | 0.33 ± 0.33 | 2 ± 0.58 | 6.33 ± 1.86 | 0.33 ± 0.33 | 1 ± 1 | 6 ± 1.15 | 2.33 ± 0.67 | 50.33 ± 19.63 |
| Glomus sp. JB-2018a | 0 ± 0 | 0 ± 0 | 0 ± 0 | 2.33 ± 0.88 | 0 ± 0 | 0 ± 0 | 0 ± 0 | 0 ± 0 | 0 ± 0 | 0 ± 0 |
| Glomus sp. N10.2 | 0.33 ± 0.33 | 13.33 ± 2.91 | 0 ± 0 | 1 ± 0 | 0 ± 0 | 0 ± 0 | 0 ± 0 | 0 ± 0 | 0 ± 0 | 0 ± 0 |
| Glomus sp. W3349 | 0 ± 0 | 0 ± 0 | 0 ± 0 | 0 ± 0 | 0 ± 0 | 0 ± 0 | 0 ± 0 | 0 ± 0 | 0 ± 0 | 0.33 ± 0.33 |
| Glomus tetrastratosum | 0.33 ± 0.33 | 0 ± 0 | 0 ± 0 | 0.67 ± 0.33 | 0 ± 0 | 0 ± 0 | 0 ± 0 | 0 ± 0 | 0 ± 0 | 0 ± 0 |
| Glutinoglossum pseudoglutinosum | 0 ± 0 | 0 ± 0 | 0 ± 0 | 0 ± 0 | 0 ± 0 | 0 ± 0 | 0 ± 0 | 5 ± 5 | 0.67 ± 0.33 | 0 ± 0 |
| Glutinomyces brunneus | 0 ± 0 | 0 ± 0 | 41 ± 35.04 | 0.33 ± 0.33 | 0 ± 0 | 19 ± 17.04 | 0 ± 0 | 0 ± 0 | 0 ± 0 | 0 ± 0 |
| Glutinomyces vulgaris | 0 ± 0 | 59.33 ± 28.59 | 17.67 ± 4.48 | 16 ± 10.07 | 0 ± 0 | 6.33 ± 4.91 | 16.33 ± 13.35 | 197.33 ± 180.38 | 16.33 ± 4.33 | 182.67 ± 55.17 |
| Golovinomyces sordidus | 9.67 ± 4.18 | 0.67 ± 0.33 | 24 ± 11.68 | 9.67 ± 6.89 | 0.33 ± 0.33 | 16.67 ± 5.81 | 13.67 ± 6.06 | 45.67 ± 25.41 | 9.33 ± 9.33 | 17.67 ± 4.67 |
| Gonatophragmium epilobii | 0 ± 0 | 0 ± 0 | 0 ± 0 | 0 ± 0 | 2 ± 2 | 0.67 ± 0.67 | 0 ± 0 | 0 ± 0 | 0 ± 0 | 0 ± 0 |
| Gongronella sp. OTU093 AN-2016 | 0 ± 0 | 0 ± 0 | 0 ± 0 | 0 ± 0 | 0 ± 0 | 0 ± 0 | 0 ± 0 | 0 ± 0 | 0 ± 0 | 6 ± 6 |
| Gorgomyces honrubiae | 0 ± 0 | 0 ± 0 | 5 ± 2.65 | 0.33 ± 0.33 | 0 ± 0 | 0 ± 0 | 0 ± 0 | 0.33 ± 0.33 | 1 ± 1 | 0 ± 0 |
| Graphium basitruncatum | 0 ± 0 | 0.33 ± 0.33 | 0.33 ± 0.33 | 0 ± 0 | 0 ± 0 | 6 ± 2.65 | 0 ± 0 | 0 ± 0 | 0 ± 0 | 0.33 ± 0.33 |
| Graphium sp. HF12719 | 0 ± 0 | 0.67 ± 0.67 | 0 ± 0 | 1.67 ± 1.2 | 4.33 ± 2.33 | 8.67 ± 6.33 | 10 ± 4.16 | 0 ± 0 | 0 ± 0 | 0 ± 0 |
| Groenewaldozyma salmanticensis | 0 ± 0 | 0 ± 0 | 0 ± 0 | 0 ± 0 | 0 ± 0 | 1.33 ± 0.88 | 1.33 ± 1.33 | 0 ± 0 | 0 ± 0 | 0 ± 0 |
| Grubyella aurea | 0 ± 0 | 0.67 ± 0.33 | 1.33 ± 0.88 | 0 ± 0 | 0 ± 0 | 0 ± 0 | 1 ± 0.58 | 9.33 ± 9.33 | 2.67 ± 2.19 | 1 ± 0 |
| Gymnoascus reesii | 0 ± 0 | 0 ± 0 | 0 ± 0 | 0 ± 0 | 0.67 ± 0.67 | 0 ± 0 | 0 ± 0 | 0 ± 0 | 0 ± 0 | 0 ± 0 |
| Gyoerffyella rotula | 0 ± 0 | 2 ± 1.15 | 0 ± 0 | 0.67 ± 0.33 | 0 ± 0 | 0.33 ± 0.33 | 1.67 ± 1.67 | 0 ± 0 | 0 ± 0 | 0 ± 0 |
| Hamamotoa lignophila | 0.33 ± 0.33 | 0 ± 0 | 0 ± 0 | 1 ± 1 | 0 ± 0 | 0 ± 0 | 0 ± 0 | 0 ± 0 | 0 ± 0 | 0 ± 0 |
| Hamatocanthoscypha sp. | 0 ± 0 | 0 ± 0 | 0 ± 0 | 0.33 ± 0.33 | 0 ± 0 | 0 ± 0 | 0 ± 0 | 0 ± 0 | 0 ± 0 | 0 ± 0 |
| Hannaella coprosmae | 0.67 ± 0.33 | 5.67 ± 0.88 | 2.33 ± 1.2 | 0.33 ± 0.33 | 1.33 ± 0.88 | 2 ± 1.15 | 3 ± 1.73 | 0 ± 0 | 0 ± 0 | 5.67 ± 0.88 |
| Hannaella kunmingensis | 1.67 ± 0.88 | 3.33 ± 2.03 | 48 ± 37.9 | 1.33 ± 0.67 | 6.33 ± 1.2 | 6.33 ± 4.37 | 7.33 ± 1.33 | 0 ± 0 | 0.33 ± 0.33 | 23.33 ± 9.21 |
| Hannaella luteola | 0 ± 0 | 0 ± 0 | 24 ± 16.09 | 0 ± 0 | 0.67 ± 0.33 | 4.67 ± 4.18 | 0.67 ± 0.67 | 0 ± 0 | 0 ± 0 | 3 ± 2.52 |
| Hannaella oryzae | 5 ± 3.61 | 0.67 ± 0.67 | 30.33 ± 13.28 | 0 ± 0 | 6.67 ± 2.91 | 5 ± 4.04 | 2.67 ± 0.88 | 1.33 ± 1.33 | 0.33 ± 0.33 | 12.33 ± 4.81 |
| Hannaella pagnoccae | 0 ± 0 | 0 ± 0 | 0 ± 0 | 0.33 ± 0.33 | 0 ± 0 | 0 ± 0 | 0 ± 0 | 0 ± 0 | 0 ± 0 | 0.33 ± 0.33 |
| Hannaella sinensis | 0 ± 0 | 0 ± 0 | 1 ± 1 | 0 ± 0 | 0 ± 0 | 0 ± 0 | 0 ± 0 | 0 ± 0 | 0 ± 0 | 0 ± 0 |
| Hannaella surugaensis | 1 ± 1 | 0 ± 0 | 2.33 ± 1.33 | 8.67 ± 1.2 | 30 ± 19.04 | 1.67 ± 1.2 | 16.33 ± 9.61 | 0 ± 0 | 0.33 ± 0.33 | 50 ± 20.31 |
| Hannaella zeae | 0 ± 0 | 12 ± 4.51 | 50.33 ± 48.35 | 0.33 ± 0.33 | 1.67 ± 1.2 | 3.33 ± 0.33 | 4.33 ± 0.67 | 0 ± 0 | 0 ± 0 | 3.67 ± 2.67 |
| Haradamyces foliicola | 0 ± 0 | 0 ± 0 | 0 ± 0 | 0 ± 0 | 0 ± 0 | 0 ± 0 | 0 ± 0 | 0.33 ± 0.33 | 0 ± 0 | 4.33 ± 1.86 |
| Harposporium bysmatosporum | 0 ± 0 | 0.67 ± 0.67 | 0 ± 0 | 0 ± 0 | 0 ± 0 | 0 ± 0 | 1 ± 1 | 0 ± 0 | 0 ± 0 | 0 ± 0 |
| Hebeloma cavipes | 0 ± 0 | 0 ± 0 | 0 ± 0 | 0 ± 0 | 0 ± 0 | 0 ± 0 | 0 ± 0 | 0 ± 0 | 0.67 ± 0.67 | 0 ± 0 |
| Hebeloma theobrominum | 0 ± 0 | 0 ± 0 | 1.33 ± 0.88 | 0 ± 0 | 0 ± 0 | 0 ± 0 | 0.33 ± 0.33 | 0 ± 0 | 0 ± 0 | 0 ± 0 |
| Helicodendron articulatum | 0 ± 0 | 0 ± 0 | 0 ± 0 | 0 ± 0 | 0 ± 0 | 0 ± 0 | 0 ± 0 | 0 ± 0 | 0 ± 0 | 2 ± 1.53 |
| Helicoubisia coronata | 0 ± 0 | 10.67 ± 2.96 | 0 ± 0 | 0.67 ± 0.33 | 0 ± 0 | 0 ± 0 | 1.33 ± 0.88 | 0 ± 0 | 0.33 ± 0.33 | 0 ± 0 |
| Helminthosporium velutinum | 0 ± 0 | 0 ± 0 | 0 ± 0 | 0 ± 0 | 0.33 ± 0.33 | 0 ± 0 | 0 ± 0 | 0 ± 0 | 0 ± 0 | 1 ± 1 |
| Hemileucoglossum pusillum | 0 ± 0 | 0 ± 0 | 0 ± 0 | 0 ± 0 | 0 ± 0 | 0.33 ± 0.33 | 0.33 ± 0.33 | 78.67 ± 77.17 | 402.33 ± 349.73 | 1.33 ± 0.67 |
| Hermatomyces sphaericus | 0 ± 0 | 0 ± 0 | 0 ± 0 | 0 ± 0 | 0 ± 0 | 0 ± 0 | 0 ± 0 | 0 ± 0 | 0 ± 0 | 5.33 ± 2.91 |
| Herpotrichia juniperi | 0.67 ± 0.67 | 0 ± 0 | 1 ± 1 | 0 ± 0 | 0 ± 0 | 1.33 ± 1.33 | 0 ± 0 | 0 ± 0 | 0 ± 0 | 0 ± 0 |
| Herpotrichia parasitica | 0 ± 0 | 0 ± 0 | 0 ± 0 | 0 ± 0 | 1.67 ± 0.88 | 0 ± 0 | 0 ± 0 | 0 ± 0 | 0 ± 0 | 0 ± 0 |
| Heterochaete sp. 2 KUC20130725-37 | 0 ± 0 | 0 ± 0 | 0 ± 0 | 0 ± 0 | 0 ± 0 | 2 ± 2 | 0.33 ± 0.33 | 0 ± 0 | 0 ± 0 | 2.33 ± 1.86 |
| Heterosphaeria patella | 0 ± 0 | 0 ± 0 | 0 ± 0 | 0 ± 0 | 0 ± 0 | 0.33 ± 0.33 | 0.67 ± 0.67 | 0 ± 0 | 0 ± 0 | 0 ± 0 |
| Hirsutella rhossiliensis | 0 ± 0 | 0 ± 0 | 0 ± 0 | 0 ± 0 | 0 ± 0 | 1 ± 1 | 0 ± 0 | 0 ± 0 | 0 ± 0 | 0 ± 0 |
| Hirsutella sp. NBRC 103844 | 0 ± 0 | 0 ± 0 | 0 ± 0 | 0 ± 0 | 0 ± 0 | 0 ± 0 | 0 ± 0 | 1 ± 1 | 1.67 ± 1.67 | 0 ± 0 |
| Hirsutella sp. NHJ12699.02 | 0 ± 0 | 0 ± 0 | 0 ± 0 | 0 ± 0 | 0 ± 0 | 0 ± 0 | 0 ± 0 | 0 ± 0 | 0 ± 0 | 1 ± 1 |
| Hirsutella strigosa | 0 ± 0 | 0 ± 0 | 1 ± 1 | 0 ± 0 | 0 ± 0 | 0 ± 0 | 0.33 ± 0.33 | 0 ± 0 | 0 ± 0 | 0 ± 0 |
| Hirsutella subulata | 0 ± 0 | 0 ± 0 | 0.33 ± 0.33 | 0 ± 0 | 0 ± 0 | 0.33 ± 0.33 | 0 ± 0 | 0 ± 0 | 0 ± 0 | 3.67 ± 3.67 |
| Hirsutella thompsonii | 0 ± 0 | 1 ± 1 | 0 ± 0 | 1 ± 0.58 | 0.33 ± 0.33 | 4.33 ± 4.33 | 1 ± 0.58 | 0 ± 0 | 0.33 ± 0.33 | 0 ± 0 |
| Hirsutella vermicola | 0 ± 0 | 0 ± 0 | 0.67 ± 0.67 | 0 ± 0 | 0 ± 0 | 1.67 ± 1.67 | 0 ± 0 | 0 ± 0 | 0 ± 0 | 0 ± 0 |
| Hobus wogradensis | 0 ± 0 | 0 ± 0 | 0.33 ± 0.33 | 0 ± 0 | 0 ± 0 | 0 ± 0 | 0 ± 0 | 0 ± 0 | 0 ± 0 | 0 ± 0 |
| Hodophilus smithii | 1.33 ± 0.67 | 0 ± 0 | 0 ± 0 | 0 ± 0 | 0 ± 0 | 0 ± 0 | 0 ± 0 | 0 ± 0 | 0 ± 0 | 0 ± 0 |
| Hodophilus variabilipes | 0 ± 0 | 2 ± 1.53 | 0 ± 0 | 0 ± 0 | 0 ± 0 | 0 ± 0 | 0.67 ± 0.67 | 0 ± 0 | 0 ± 0 | 0 ± 0 |
| Holocotylon brandegeeanum | 0 ± 0 | 0 ± 0 | 0 ± 0 | 0.67 ± 0.67 | 0 ± 0 | 0 ± 0 | 0 ± 0 | 0 ± 0 | 0 ± 0 | 0 ± 0 |
| Hormiactis candida | 0.67 ± 0.67 | 0 ± 0 | 0 ± 0 | 0 ± 0 | 5.67 ± 3.18 | 4.67 ± 2.6 | 3 ± 2.52 | 0.33 ± 0.33 | 0.33 ± 0.33 | 0.33 ± 0.33 |
| Humicola cuyabenoensis | 0.33 ± 0.33 | 108.33 ± 13.42 | 2.67 ± 1.76 | 28 ± 14.53 | 0.67 ± 0.33 | 13 ± 5.57 | 277.67 ± 237.04 | 6.67 ± 1.2 | 2.33 ± 1.45 | 5.67 ± 0.88 |
| Humicola fuscoatra | 0.33 ± 0.33 | 0 ± 0 | 3.33 ± 1.2 | 0.33 ± 0.33 | 1 ± 0.58 | 17 ± 15.52 | 1.33 ± 0.33 | 3.33 ± 1.33 | 1.33 ± 1.33 | 19.67 ± 19.67 |
| Humicola grisea | 0.67 ± 0.67 | 0 ± 0 | 35.67 ± 10.99 | 2.33 ± 1.86 | 5.67 ± 1.45 | 104 ± 57.38 | 49 ± 30.55 | 1 ± 0.58 | 0 ± 0 | 8.33 ± 1.67 |
| Humicola nigrescens | 0 ± 0 | 0 ± 0 | 0 ± 0 | 0 ± 0 | 0 ± 0 | 0 ± 0 | 0 ± 0 | 0 ± 0 | 0 ± 0 | 0 ± 0 |
| Humicola phialophoroides | 0 ± 0 | 0 ± 0 | 0 ± 0 | 0 ± 0 | 0 ± 0 | 0.33 ± 0.33 | 0.33 ± 0.33 | 0.33 ± 0.33 | 0 ± 0 | 0 ± 0 |
| Humicola sp. | 5.67 ± 0.67 | 762 ± 251.18 | 2.67 ± 2.67 | 38 ± 11.02 | 16.33 ± 14.84 | 9 ± 8.02 | 174.33 ± 81.87 | 34.33 ± 28.95 | 5.67 ± 2.85 | 44.33 ± 15.34 |
| Humicola sp. Ecu212 | 0 ± 0 | 0.33 ± 0.33 | 0 ± 0 | 0.33 ± 0.33 | 0 ± 0 | 0 ± 0 | 6.67 ± 3.76 | 0.33 ± 0.33 | 0 ± 0 | 0 ± 0 |
| Humicola sp. L-2 | 9.67 ± 1.2 | 2223.67 ± 471.15 | 13.67 ± 4.41 | 97.67 ± 36.4 | 7.33 ± 0.88 | 55 ± 20.95 | 464.33 ± 180.57 | 119.67 ± 54.85 | 10.67 ± 2.6 | 82.33 ± 6.36 |
| Humicolopsis cephalosporioides | 0 ± 0 | 0 ± 0 | 0 ± 0 | 0 ± 0 | 0 ± 0 | 1 ± 1 | 0 ± 0 | 0 ± 0 | 0 ± 0 | 0 ± 0 |
| Hyalorbilia erythrostigma | 0 ± 0 | 0 ± 0 | 0 ± 0 | 0 ± 0 | 0 ± 0 | 0 ± 0 | 0 ± 0 | 0 ± 0 | 1.33 ± 1.33 | 0 ± 0 |
| Hyalorbilia fusispora | 0.67 ± 0.67 | 15 ± 10.02 | 2 ± 0.58 | 1 ± 1 | 3.33 ± 1.45 | 4 ± 1.53 | 7 ± 6.03 | 0 ± 0 | 0.33 ± 0.33 | 0 ± 0 |
| Hyalorbilia juliae | 4 ± 3.06 | 10 ± 7.21 | 1.33 ± 0.88 | 2.33 ± 1.86 | 2 ± 1.15 | 19 ± 14.01 | 7.33 ± 3.67 | 2.67 ± 0.67 | 5 ± 2 | 19.67 ± 13.68 |
| Hyalorbilia sp. HB19 | 0 ± 0 | 49.67 ± 5.46 | 0 ± 0 | 3.67 ± 0.67 | 0 ± 0 | 0 ± 0 | 10.67 ± 5.61 | 0 ± 0 | 1 ± 1 | 0 ± 0 |
| Hyalorbilia sp. HOB-2016c | 0 ± 0 | 0 ± 0 | 0 ± 0 | 0 ± 0 | 0 ± 0 | 1 ± 1 | 0 ± 0 | 28.67 ± 27.18 | 9.33 ± 9.33 | 0 ± 0 |
| Hyalorbilia sp. HOB-2017b | 0 ± 0 | 0 ± 0 | 0 ± 0 | 0 ± 0 | 0 ± 0 | 0 ± 0 | 0 ± 0 | 0 ± 0 | 0 ± 0 | 0.67 ± 0.67 |
| Hyalorbilia sp. HOB-2017c | 0 ± 0 | 0 ± 0 | 0 ± 0 | 0.33 ± 0.33 | 0 ± 0 | 1 ± 1 | 1 ± 1 | 0 ± 0 | 0 ± 0 | 2.33 ± 2.33 |
| Hyaloscypha bicolor | 0 ± 0 | 0.33 ± 0.33 | 0 ± 0 | 0 ± 0 | 0 ± 0 | 0 ± 0 | 0 ± 0 | 0 ± 0 | 0 ± 0 | 0.33 ± 0.33 |
| Hydropisphaera erubescens | 0 ± 0 | 0 ± 0 | 3.67 ± 1.86 | 0 ± 0 | 0 ± 0 | 0.33 ± 0.33 | 0 ± 0 | 72.33 ± 66.37 | 15 ± 14.01 | 0.67 ± 0.33 |
| Hygrocybe conica | 0 ± 0 | 0 ± 0 | 3 ± 1.15 | 0.33 ± 0.33 | 0.67 ± 0.67 | 0.33 ± 0.33 | 0.67 ± 0.67 | 0 ± 0 | 0 ± 0 | 0 ± 0 |
| Hygrocybe glutinipes | 0 ± 0 | 0 ± 0 | 0 ± 0 | 0 ± 0 | 0 ± 0 | 0.67 ± 0.67 | 0 ± 0 | 0 ± 0 | 0 ± 0 | 1.67 ± 1.67 |
| Hygrocybe konradii | 264.67 ± 19.6 | 2 ± 1 | 0.33 ± 0.33 | 0 ± 0 | 13.67 ± 1.86 | 2.33 ± 2.33 | 3.67 ± 2.67 | 0.33 ± 0.33 | 0 ± 0 | 0 ± 0 |
| Hygrocybe persistens | 0.67 ± 0.67 | 0 ± 0 | 0 ± 0 | 0 ± 0 | 1.67 ± 1.67 | 0 ± 0 | 0 ± 0 | 0 ± 0 | 0 ± 0 | 0 ± 0 |
| Hygrocybe pseudoconica | 0 ± 0 | 0 ± 0 | 0 ± 0 | 0 ± 0 | 0 ± 0 | 0 ± 0 | 0 ± 0 | 0 ± 0 | 0 ± 0 | 1 ± 1 |
| Hygrocybe sp. | 0 ± 0 | 0 ± 0 | 0 ± 0 | 0 ± 0 | 0 ± 0 | 2 ± 2 | 0 ± 0 | 0 ± 0 | 0 ± 0 | 0 ± 0 |
| Hymenogaster arenarius | 0 ± 0 | 0 ± 0 | 0.33 ± 0.33 | 4.33 ± 3.38 | 0 ± 0 | 8.33 ± 4.84 | 16.67 ± 9.7 | 6 ± 2.52 | 1.67 ± 0.67 | 0.67 ± 0.67 |
| Hymenogaster niveus | 0 ± 0 | 0 ± 0 | 0 ± 0 | 0 ± 0 | 0 ± 0 | 0.67 ± 0.67 | 0 ± 0 | 0 ± 0 | 0 ± 0 | 0 ± 0 |
| Hymenogaster rubyensis | 0 ± 0 | 0 ± 0 | 0 ± 0 | 0 ± 0 | 0 ± 0 | 0 ± 0 | 0 ± 0 | 0 ± 0 | 0 ± 0 | 1.33 ± 1.33 |
| Hymenogaster sp. 1 SGT-2012 | 0 ± 0 | 0 ± 0 | 0.67 ± 0.67 | 0 ± 0 | 0 ± 0 | 2 ± 0.58 | 5.33 ± 4.37 | 0.33 ± 0.33 | 0 ± 0 | 0 ± 0 |
| Hymenoscyphus fructigenus | 0 ± 0 | 0 ± 0 | 0 ± 0 | 0 ± 0 | 0 ± 0 | 0 ± 0 | 0 ± 0 | 0 ± 0 | 0 ± 0 | 0.67 ± 0.67 |
| Hymenoscyphus monotropae | 0 ± 0 | 0 ± 0 | 0 ± 0 | 0 ± 0 | 0 ± 0 | 0 ± 0 | 0.67 ± 0.67 | 0 ± 0 | 0 ± 0 | 0 ± 0 |
| Hymenoscyphus sp. 2 FC-5110 | 0 ± 0 | 0 ± 0 | 0 ± 0 | 2.67 ± 1.76 | 0 ± 0 | 0 ± 0 | 0.67 ± 0.67 | 0 ± 0 | 0 ± 0 | 0 ± 0 |
| Hymenoscyphus sp. FC-2727 | 0 ± 0 | 0 ± 0 | 0.67 ± 0.67 | 0 ± 0 | 0 ± 0 | 84.67 ± 72.81 | 0.33 ± 0.33 | 0 ± 0 | 0 ± 0 | 0.33 ± 0.33 |
| Hyphodermella corrugata | 0 ± 0 | 0 ± 0 | 0.67 ± 0.67 | 0.33 ± 0.33 | 0 ± 0 | 0 ± 0 | 0 ± 0 | 0 ± 0 | 0 ± 0 | 0 ± 0 |
| Hyphodermella sp. | 0 ± 0 | 0 ± 0 | 0 ± 0 | 0 ± 0 | 0 ± 0 | 0 ± 0 | 0 ± 0 | 0 ± 0 | 0 ± 0 | 0 ± 0 |
| Hyphodontia pallidula | 0 ± 0 | 2 ± 1.15 | 2 ± 1.53 | 38 ± 27.06 | 0 ± 0 | 3.67 ± 0.88 | 17.67 ± 11.41 | 0 ± 0 | 0 ± 0 | 0 ± 0 |
| Hypholoma fasciculare | 0 ± 0 | 0 ± 0 | 0 ± 0 | 0 ± 0 | 0 ± 0 | 0.33 ± 0.33 | 0 ± 0 | 3.33 ± 2.85 | 0 ± 0 | 0 ± 0 |
| Hypholoma sublateritium | 0 ± 0 | 0 ± 0 | 0 ± 0 | 0 ± 0 | 0 ± 0 | 0.33 ± 0.33 | 0 ± 0 | 2 ± 1.53 | 0 ± 0 | 0 ± 0 |
| Hypochnicium cremicolor | 0 ± 0 | 0 ± 0 | 0.33 ± 0.33 | 0 ± 0 | 0 ± 0 | 0 ± 0 | 0 ± 0 | 0 ± 0 | 0 ± 0 | 0 ± 0 |
| Hypocreopsis rhododendri | 0 ± 0 | 0 ± 0 | 0 ± 0 | 0 ± 0 | 0 ± 0 | 8.67 ± 5.21 | 7 ± 6.51 | 0.67 ± 0.67 | 0 ± 0 | 0 ± 0 |
| Hypomyces armeniacus | 0 ± 0 | 0 ± 0 | 8.67 ± 2.73 | 0 ± 0 | 0 ± 0 | 8 ± 6.56 | 0.67 ± 0.67 | 1 ± 1 | 3.67 ± 2.73 | 0 ± 0 |
| Hypomyces aurantius | 0 ± 0 | 0 ± 0 | 0.67 ± 0.67 | 1.33 ± 0.33 | 0.33 ± 0.33 | 8.33 ± 4.48 | 2.67 ± 1.76 | 0 ± 0 | 0 ± 0 | 1.33 ± 1.33 |
| Hypomyces australis | 0 ± 0 | 0 ± 0 | 0 ± 0 | 0 ± 0 | 0 ± 0 | 7 ± 3.51 | 1 ± 1 | 0 ± 0 | 0 ± 0 | 0 ± 0 |
| Hypomyces cervinigenus | 0 ± 0 | 0 ± 0 | 3.67 ± 0.33 | 0 ± 0 | 0 ± 0 | 0 ± 0 | 0 ± 0 | 0 ± 0 | 0 ± 0 | 0 ± 0 |
| Hypomyces chrysospermus | 1.33 ± 1.33 | 0 ± 0 | 0 ± 0 | 0.33 ± 0.33 | 12 ± 11.5 | 1.67 ± 1.2 | 1.67 ± 1.67 | 0 ± 0 | 0 ± 0 | 0 ± 0 |
| Hypomyces peltigericola | 2.33 ± 0.33 | 0 ± 0 | 0 ± 0 | 0 ± 0 | 0 ± 0 | 0 ± 0 | 0 ± 0 | 0 ± 0 | 0 ± 0 | 0 ± 0 |
| Hypomyces rosellus | 0 ± 0 | 0 ± 0 | 0 ± 0 | 0 ± 0 | 0.67 ± 0.67 | 1 ± 0.58 | 0.33 ± 0.33 | 0 ± 0 | 0 ± 0 | 0.33 ± 0.33 |
| Hypomyces xyloboli | 0 ± 0 | 0 ± 0 | 0 ± 0 | 0 ± 0 | 0 ± 0 | 0 ± 0 | 2 ± 2 | 0 ± 0 | 0 ± 0 | 0 ± 0 |
| Hypoxylon perforatum | 0 ± 0 | 0 ± 0 | 0 ± 0 | 0 ± 0 | 0 ± 0 | 0 ± 0 | 0 ± 0 | 0 ± 0 | 0 ± 0 | 1.67 ± 1.67 |
| Hypoxylon vinosopulvinatum | 0 ± 0 | 0 ± 0 | 0 ± 0 | 0 ± 0 | 1 ± 1 | 0 ± 0 | 0 ± 0 | 0 ± 0 | 0 ± 0 | 0 ± 0 |
| Idriella cubensis | 0 ± 0 | 0 ± 0 | 0 ± 0 | 0 ± 0 | 0 ± 0 | 0 ± 0 | 0 ± 0 | 11 ± 7.77 | 1.67 ± 1.67 | 0.33 ± 0.33 |
| Idriella lunata | 1.33 ± 0.88 | 0 ± 0 | 45.67 ± 22.41 | 2 ± 1.53 | 19 ± 1.15 | 18.33 ± 4.91 | 27.67 ± 22.18 | 0.33 ± 0.33 | 0.33 ± 0.33 | 3.33 ± 1.45 |
| Idriella rara | 0 ± 0 | 0 ± 0 | 2.67 ± 1.45 | 2 ± 2 | 3.67 ± 2.73 | 86 ± 36.29 | 9 ± 2.65 | 62.33 ± 32.57 | 30.67 ± 30.17 | 16.33 ± 5.81 |
| Ijuhya corynospora | 0 ± 0 | 0 ± 0 | 0 ± 0 | 0 ± 0 | 0.67 ± 0.67 | 0 ± 0 | 0 ± 0 | 0 ± 0 | 0 ± 0 | 0 ± 0 |
| Ijuhya peristomialis | 0 ± 0 | 0 ± 0 | 0 ± 0 | 0 ± 0 | 1 ± 1 | 0 ± 0 | 0 ± 0 | 0 ± 0 | 0 ± 0 | 0 ± 0 |
| Ijuhya sp. | 0 ± 0 | 0.33 ± 0.33 | 0 ± 0 | 0 ± 0 | 1.33 ± 1.33 | 0.67 ± 0.67 | 0 ± 0 | 0 ± 0 | 0 ± 0 | 0 ± 0 |
| Ijuhya vitellina | 10.67 ± 3.53 | 0 ± 0 | 2.67 ± 0.33 | 0 ± 0 | 5.67 ± 0.88 | 1.33 ± 0.88 | 0.33 ± 0.33 | 2 ± 2 | 1.67 ± 1.67 | 0 ± 0 |
| Ilyonectria capensis | 0.33 ± 0.33 | 0 ± 0 | 0 ± 0 | 0 ± 0 | 0 ± 0 | 0 ± 0 | 0.33 ± 0.33 | 0.33 ± 0.33 | 0 ± 0 | 0 ± 0 |
| Ilyonectria coprosmae | 0 ± 0 | 0 ± 0 | 0 ± 0 | 0 ± 0 | 0 ± 0 | 0 ± 0 | 0.67 ± 0.67 | 0 ± 0 | 0 ± 0 | 0 ± 0 |
| Ilyonectria liliigena | 0.33 ± 0.33 | 0 ± 0 | 0.67 ± 0.67 | 2.67 ± 1.76 | 0.33 ± 0.33 | 4 ± 2.31 | 10 ± 8.5 | 27.67 ± 19.38 | 20 ± 19.5 | 1.67 ± 1.67 |
| Ilyonectria liriodendri | 0 ± 0 | 0.67 ± 0.33 | 18 ± 2.65 | 0.33 ± 0.33 | 0.67 ± 0.33 | 107.67 ± 91.2 | 59.67 ± 58.67 | 1.67 ± 1.67 | 9.33 ± 4.7 | 1.67 ± 0.67 |
| Ilyonectria robusta | 43 ± 27.62 | 0.33 ± 0.33 | 179.33 ± 71 | 21.67 ± 6.49 | 109 ± 27.5 | 81.33 ± 10.68 | 67 ± 34.24 | 28.33 ± 8.29 | 20.67 ± 8.69 | 50.67 ± 29.41 |
| Ilyonectria sp. | 0 ± 0 | 0 ± 0 | 0 ± 0 | 0 ± 0 | 0 ± 0 | 20.67 ± 10.97 | 20 ± 16.5 | 0 ± 0 | 0.33 ± 0.33 | 0.67 ± 0.67 |
| Ilyonectria sp. M64 | 0 ± 0 | 0 ± 0 | 0 ± 0 | 0 ± 0 | 0.67 ± 0.67 | 0 ± 0 | 0 ± 0 | 0 ± 0 | 0 ± 0 | 0.67 ± 0.33 |
| Immersidiscosia eucalypti | 0 ± 0 | 0 ± 0 | 2.67 ± 2.67 | 0 ± 0 | 0.33 ± 0.33 | 1.33 ± 1.33 | 0 ± 0 | 0 ± 0 | 0 ± 0 | 0 ± 0 |
| Incrucipulum ciliare | 0 ± 0 | 0 ± 0 | 0 ± 0 | 0.67 ± 0.33 | 0 ± 0 | 0 ± 0 | 0.67 ± 0.33 | 0 ± 0 | 0.33 ± 0.33 | 1.33 ± 1.33 |
| Infundichalara microchona | 0 ± 0 | 0 ± 0 | 0 ± 0 | 0 ± 0 | 0 ± 0 | 0 ± 0 | 0 ± 0 | 6.67 ± 3.76 | 8.33 ± 8.33 | 0.33 ± 0.33 |
| Inocybe candidipes | 0 ± 0 | 0 ± 0 | 0 ± 0 | 0 ± 0 | 0 ± 0 | 0 ± 0 | 0 ± 0 | 0.67 ± 0.67 | 0 ± 0 | 0 ± 0 |
| Inocybe ochroalba | 2.33 ± 2.33 | 0.33 ± 0.33 | 57.67 ± 22.64 | 0 ± 0 | 0 ± 0 | 5.67 ± 3.18 | 2 ± 1 | 0 ± 0 | 0.33 ± 0.33 | 10 ± 7.09 |
| Inocybe rimosa | 0 ± 0 | 0 ± 0 | 0 ± 0 | 0 ± 0 | 0 ± 0 | 0 ± 0 | 0 ± 0 | 0 ± 0 | 0 ± 0 | 2.33 ± 2.33 |
| Inocybe sp. B153 | 0 ± 0 | 0 ± 0 | 0 ± 0 | 0 ± 0 | 0 ± 0 | 3.33 ± 2.03 | 0 ± 0 | 0 ± 0 | 0 ± 0 | 0 ± 0 |
| Isaria takamizusanensis | 0 ± 0 | 0 ± 0 | 2 ± 1 | 0.67 ± 0.67 | 0 ± 0 | 0.67 ± 0.33 | 0 ± 0 | 0 ± 0 | 0 ± 0 | 0 ± 0 |
| Ischnoderma resinosum | 0 ± 0 | 0 ± 0 | 2.67 ± 2.19 | 0 ± 0 | 0 ± 0 | 0 ± 0 | 0 ± 0 | 0 ± 0 | 0 ± 0 | 0 ± 0 |
| Jattaea leucospermi | 0 ± 0 | 2 ± 2 | 0.33 ± 0.33 | 0 ± 0 | 0.67 ± 0.67 | 3.67 ± 3.67 | 1 ± 1 | 0.33 ± 0.33 | 0 ± 0 | 474 ± 474 |
| Jugulospora rotula | 0 ± 0 | 0 ± 0 | 0 ± 0 | 0 ± 0 | 0 ± 0 | 0 ± 0 | 1.67 ± 1.67 | 0 ± 0 | 0 ± 0 | 0 ± 0 |
| Junewangia globulosa | 5.67 ± 3.48 | 116 ± 47.26 | 0 ± 0 | 10.33 ± 2.03 | 1.67 ± 1.2 | 1 ± 1 | 15.33 ± 7.33 | 2 ± 1 | 7.67 ± 6.17 | 9.33 ± 5.81 |
| Juxtiphoma eupyrena | 0.67 ± 0.67 | 0 ± 0 | 0 ± 0 | 0.33 ± 0.33 | 12.33 ± 1.2 | 2.33 ± 2.33 | 1.33 ± 0.67 | 0.67 ± 0.67 | 0 ± 0 | 11 ± 9.07 |
| Karstenula sp. | 0 ± 0 | 0 ± 0 | 0 ± 0 | 0 ± 0 | 0 ± 0 | 0 ± 0 | 1 ± 1 | 0 ± 0 | 0 ± 0 | 0 ± 0 |
| Kavinia aff. alboviridis UC2022816 | 0 ± 0 | 0 ± 0 | 0 ± 0 | 0 ± 0 | 0 ± 0 | 0 ± 0 | 0 ± 0 | 1.67 ± 1.2 | 4 ± 3.51 | 53 ± 53 |
| Keratinophyton durum | 0 ± 0 | 0 ± 0 | 0 ± 0 | 0 ± 0 | 0 ± 0 | 0.33 ± 0.33 | 0 ± 0 | 0 ± 0 | 2 ± 1.53 | 9 ± 1 |
| Kiflimonium curvulum | 0 ± 0 | 4.67 ± 2.6 | 0 ± 0 | 0 ± 0 | 0 ± 0 | 0.33 ± 0.33 | 0.33 ± 0.33 | 1.67 ± 0.88 | 0.67 ± 0.67 | 30.33 ± 7.26 |
| Knufia cryptophialidica | 0 ± 0 | 0 ± 0 | 0 ± 0 | 0 ± 0 | 0 ± 0 | 0 ± 0 | 0 ± 0 | 1 ± 0.58 | 1 ± 0.58 | 0 ± 0 |
| Knufia marmoricola | 0 ± 0 | 0.67 ± 0.67 | 0 ± 0 | 0 ± 0 | 0 ± 0 | 0 ± 0 | 0 ± 0 | 0 ± 0 | 0 ± 0 | 0 ± 0 |
| Knufia perforans | 2.33 ± 1.2 | 267.33 ± 42.49 | 0 ± 0 | 17 ± 7.37 | 0.67 ± 0.67 | 1 ± 0.58 | 50.67 ± 18.59 | 16.67 ± 4.06 | 14.33 ± 3.38 | 1613 ± 781.39 |
| Knufia sp. | 1.67 ± 0.88 | 359 ± 150.81 | 2 ± 1.53 | 18 ± 8.19 | 0 ± 0 | 1.67 ± 0.33 | 63.33 ± 30.6 | 58.67 ± 25.01 | 67.67 ± 56.74 | 3.33 ± 2.4 |
| Kockovaella cucphuongensis | 0 ± 0 | 0 ± 0 | 0 ± 0 | 0.33 ± 0.33 | 0 ± 0 | 0 ± 0 | 0 ± 0 | 0.33 ± 0.33 | 0 ± 0 | 0 ± 0 |
| Kockovaella lichenicola | 0 ± 0 | 0 ± 0 | 0 ± 0 | 0 ± 0 | 0 ± 0 | 0 ± 0 | 0 ± 0 | 0 ± 0 | 0 ± 0 | 0.67 ± 0.67 |
| Kockovaella schimae | 0 ± 0 | 0 ± 0 | 0 ± 0 | 0 ± 0 | 0 ± 0 | 0 ± 0 | 0 ± 0 | 1 ± 1 | 0 ± 0 | 0 ± 0 |
| Kondoa gutianensis | 0 ± 0 | 0 ± 0 | 0.33 ± 0.33 | 0 ± 0 | 0 ± 0 | 0 ± 0 | 0 ± 0 | 0 ± 0 | 0 ± 0 | 0.67 ± 0.67 |
| Kondoa miscanthi | 0 ± 0 | 0 ± 0 | 0 ± 0 | 0 ± 0 | 0 ± 0 | 0 ± 0 | 0 ± 0 | 0.33 ± 0.33 | 0 ± 0 | 2.67 ± 0.88 |
| Kondoa sp. 'myxariophila' | 0 ± 0 | 0 ± 0 | 0 ± 0 | 0 ± 0 | 0 ± 0 | 0 ± 0 | 0 ± 0 | 0 ± 0 | 0 ± 0 | 0.67 ± 0.67 |
| Koorchaloma spartinicola | 0 ± 0 | 7.67 ± 6.23 | 0 ± 0 | 0.33 ± 0.33 | 0 ± 0 | 0 ± 0 | 0.67 ± 0.67 | 0 ± 0 | 0 ± 0 | 0 ± 0 |
| Kretzschmaria sp. AK-2 | 0 ± 0 | 0 ± 0 | 0 ± 0 | 0 ± 0 | 0 ± 0 | 0 ± 0 | 1 ± 1 | 3.33 ± 3.33 | 0 ± 0 | 0 ± 0 |
| Kurtzmanomyces insolitus | 0 ± 0 | 0 ± 0 | 0.33 ± 0.33 | 0 ± 0 | 0.33 ± 0.33 | 1 ± 1 | 0 ± 0 | 0 ± 0 | 0 ± 0 | 0 ± 0 |
| Kurtzmanomyces shapotouensis | 0 ± 0 | 0 ± 0 | 0 ± 0 | 1 ± 0.58 | 0 ± 0 | 0.33 ± 0.33 | 0 ± 0 | 0 ± 0 | 0 ± 0 | 0 ± 0 |
| Kurtzmanomyces sp. MG21 | 1.67 ± 1.67 | 0 ± 0 | 0 ± 0 | 0 ± 0 | 0 ± 0 | 0 ± 0 | 0 ± 0 | 0 ± 0 | 0 ± 0 | 0 ± 0 |
| Kurtzmanomyces tardus | 0 ± 0 | 0 ± 0 | 0 ± 0 | 0 ± 0 | 0 ± 0 | 0 ± 0 | 1.33 ± 1.33 | 8 ± 6.11 | 0 ± 0 | 0 ± 0 |
| Lachnellula subtilissima | 0.33 ± 0.33 | 0 ± 0 | 25 ± 15.01 | 0 ± 0 | 0.67 ± 0.67 | 11.67 ± 10.68 | 0.33 ± 0.33 | 0 ± 0 | 0 ± 0 | 0 ± 0 |
| Lachnum sp. | 0 ± 0 | 1 ± 0.58 | 0 ± 0 | 0 ± 0 | 0 ± 0 | 0 ± 0 | 0.33 ± 0.33 | 0 ± 0 | 0 ± 0 | 0 ± 0 |
| Lachnum sp. 2 KO-2013 | 0 ± 0 | 0 ± 0 | 0 ± 0 | 0 ± 0 | 0 ± 0 | 0 ± 0 | 0 ± 0 | 0 ± 0 | 1 ± 1 | 0 ± 0 |
| Lachnum willisii | 0 ± 0 | 0 ± 0 | 0 ± 0 | 0 ± 0 | 1.67 ± 1.67 | 0 ± 0 | 0 ± 0 | 0 ± 0 | 0 ± 0 | 0 ± 0 |
| Lactarius alboscrobiculatus | 0 ± 0 | 0 ± 0 | 2 ± 2 | 0 ± 0 | 0 ± 0 | 0 ± 0 | 0 ± 0 | 0 ± 0 | 0 ± 0 | 0 ± 0 |
| Lactarius hatsudake | 0 ± 0 | 0 ± 0 | 0 ± 0 | 0 ± 0 | 0 ± 0 | 0 ± 0 | 0 ± 0 | 1.67 ± 1.67 | 0 ± 0 | 0 ± 0 |
| Laetisaria arvalis | 0 ± 0 | 0 ± 0 | 0 ± 0 | 0 ± 0 | 0 ± 0 | 0 ± 0 | 0 ± 0 | 5 ± 4.51 | 15 ± 15 | 0 ± 0 |
| Lamprospora cf. areolata | 0 ± 0 | 0.67 ± 0.67 | 0 ± 0 | 0 ± 0 | 0 ± 0 | 0 ± 0 | 0 ± 0 | 0 ± 0 | 0 ± 0 | 0 ± 0 |
| Lanzia sp. WWYZ-2015 | 0.33 ± 0.33 | 0 ± 0 | 0 ± 0 | 0 ± 0 | 4.33 ± 1.86 | 1.33 ± 1.33 | 0.33 ± 0.33 | 0 ± 0 | 0 ± 0 | 3 ± 1.53 |
| Lasionectria oenanthicola | 0 ± 0 | 0 ± 0 | 1.33 ± 1.33 | 0 ± 0 | 0 ± 0 | 2.33 ± 2.33 | 0 ± 0 | 4 ± 4 | 0 ± 0 | 0 ± 0 |
| Lasionectria sp. DV-2018a | 0 ± 0 | 0 ± 0 | 2.67 ± 1.76 | 0.33 ± 0.33 | 0.33 ± 0.33 | 0 ± 0 | 0 ± 0 | 0 ± 0 | 0 ± 0 | 0 ± 0 |
| Lasiosphaeria lanuginosa | 0 ± 0 | 0.33 ± 0.33 | 12 ± 7.21 | 13.67 ± 11.68 | 0 ± 0 | 4.67 ± 3.28 | 6 ± 3.21 | 69.67 ± 43 | 14.33 ± 11.92 | 14.67 ± 6.36 |
| Lasiosphaeria similisorbina | 0.67 ± 0.33 | 6 ± 5.51 | 0 ± 0 | 0.33 ± 0.33 | 0 ± 0 | 0 ± 0 | 0.67 ± 0.67 | 0 ± 0 | 0 ± 0 | 0 ± 0 |
| Lasiosphaeria sorbina | 0 ± 0 | 0 ± 0 | 0 ± 0 | 1.33 ± 0.88 | 0 ± 0 | 0.67 ± 0.67 | 0 ± 0 | 0.33 ± 0.33 | 1.67 ± 1.2 | 0 ± 0 |
| Lasiosphaeria sp. SH-2018a | 0 ± 0 | 0 ± 0 | 0 ± 0 | 1.67 ± 0.88 | 0 ± 0 | 0 ± 0 | 0.33 ± 0.33 | 2.67 ± 2.19 | 0.67 ± 0.33 | 4 ± 1.15 |
| Latorua caligans | 0 ± 0 | 0 ± 0 | 0 ± 0 | 0 ± 0 | 0.33 ± 0.33 | 0 ± 0 | 0.33 ± 0.33 | 0.67 ± 0.67 | 0 ± 0 | 0 ± 0 |
| Lecanicillium antillanum | 0 ± 0 | 0 ± 0 | 0 ± 0 | 0 ± 0 | 0.33 ± 0.33 | 0 ± 0 | 2.33 ± 2.33 | 0 ± 0 | 0 ± 0 | 0 ± 0 |
| Lecanicillium dimorphum | 0 ± 0 | 0 ± 0 | 0 ± 0 | 0 ± 0 | 0 ± 0 | 0 ± 0 | 0 ± 0 | 0.33 ± 0.33 | 0 ± 0 | 0 ± 0 |
| Lecanicillium fungicola | 0 ± 0 | 0 ± 0 | 0.33 ± 0.33 | 0.67 ± 0.67 | 0 ± 0 | 0 ± 0 | 1.33 ± 1.33 | 0 ± 0 | 0.33 ± 0.33 | 0 ± 0 |
| Lecanicillium kalimantanense | 0 ± 0 | 0 ± 0 | 0 ± 0 | 1 ± 1 | 0 ± 0 | 1.67 ± 1.67 | 0.33 ± 0.33 | 0 ± 0 | 0 ± 0 | 0 ± 0 |
| Lecanicillium psalliotae | 0.33 ± 0.33 | 0 ± 0 | 1 ± 1 | 3.67 ± 1.86 | 1 ± 0 | 9 ± 4.73 | 6.67 ± 1.86 | 1.67 ± 0.33 | 2 ± 1 | 4.67 ± 2.19 |
| Lecanicillium saksenae | 31.67 ± 12.57 | 10.33 ± 4.98 | 0.67 ± 0.67 | 3 ± 1.15 | 2.33 ± 1.2 | 2.33 ± 1.2 | 3.67 ± 2.19 | 1.33 ± 0.88 | 0.33 ± 0.33 | 1.33 ± 0.88 |
| Lecanicillium sp. | 1.33 ± 0.88 | 6.33 ± 5.84 | 0 ± 0 | 0.67 ± 0.67 | 11 ± 1.15 | 3.67 ± 1.86 | 4 ± 2.31 | 15.33 ± 11.02 | 6.67 ± 6.67 | 0.67 ± 0.67 |
| Lecophagus sp. ATCC 56071 | 0 ± 0 | 0 ± 0 | 1.33 ± 1.33 | 0 ± 0 | 0.33 ± 0.33 | 1 ± 1 | 1 ± 0.58 | 0 ± 0 | 0 ± 0 | 0 ± 0 |
| Lectera colletotrichoides | 0 ± 0 | 23 ± 13.65 | 0 ± 0 | 0.67 ± 0.67 | 0 ± 0 | 1.33 ± 1.33 | 0.33 ± 0.33 | 0 ± 0 | 0 ± 0 | 0 ± 0 |
| Leiosphaerella lycopodina | 0 ± 0 | 0 ± 0 | 0.33 ± 0.33 | 0 ± 0 | 0 ± 0 | 4.33 ± 3.84 | 0.67 ± 0.67 | 0 ± 0 | 0 ± 0 | 0 ± 0 |
| Leiothecium ellipsoideum | 0 ± 0 | 0 ± 0 | 1 ± 1 | 0.67 ± 0.33 | 0 ± 0 | 0 ± 0 | 0 ± 0 | 0 ± 0 | 0 ± 0 | 0.67 ± 0.67 |
| Leohumicola minima | 0 ± 0 | 24.33 ± 11.26 | 1 ± 1 | 10.33 ± 3.84 | 0.33 ± 0.33 | 0 ± 0 | 9 ± 5.86 | 0.67 ± 0.33 | 2 ± 1.53 | 1.67 ± 1.67 |
| Leohumicola sp. DAOM 230084 | 0 ± 0 | 0 ± 0 | 2 ± 2 | 0 ± 0 | 0 ± 0 | 0.67 ± 0.67 | 0 ± 0 | 0 ± 0 | 0 ± 0 | 0 ± 0 |
| Lepiota sp. MFLU 09-0142 | 0 ± 0 | 0 ± 0 | 0 ± 0 | 0 ± 0 | 0 ± 0 | 0 ± 0 | 0.33 ± 0.33 | 0 ± 0 | 0.67 ± 0.33 | 0 ± 0 |
| Lepiota sp. Yang 1957 | 0 ± 0 | 0.33 ± 0.33 | 0 ± 0 | 0 ± 0 | 0 ± 0 | 0 ± 0 | 3.67 ± 3.67 | 11.33 ± 2.19 | 2297 ± 2286.5 | 2.33 ± 1.45 |
| Lepista nuda | 0 ± 0 | 0 ± 0 | 0.33 ± 0.33 | 0 ± 0 | 0 ± 0 | 0 ± 0 | 0 ± 0 | 0 ± 0 | 0 ± 0 | 0 ± 0 |
| Lepista panaeola | 0 ± 0 | 0 ± 0 | 0 ± 0 | 0 ± 0 | 0 ± 0 | 0 ± 0 | 0 ± 0 | 0.67 ± 0.67 | 0 ± 0 | 0 ± 0 |
| Lepista sordida | 0 ± 0 | 0 ± 0 | 0 ± 0 | 0 ± 0 | 0 ± 0 | 0.33 ± 0.33 | 3 ± 3 | 0.33 ± 0.33 | 0 ± 0 | 0 ± 0 |
| Lepteutypa sambuci | 0 ± 0 | 0 ± 0 | 0 ± 0 | 0 ± 0 | 0 ± 0 | 1 ± 1 | 0 ± 0 | 0 ± 0 | 0 ± 0 | 0 ± 0 |
| Leptodiscella africana | 1 ± 1 | 3 ± 1.53 | 6.67 ± 3.28 | 0.33 ± 0.33 | 0 ± 0 | 1.33 ± 0.88 | 4.33 ± 2.6 | 0 ± 0 | 0 ± 0 | 0 ± 0 |
| Leptodiscella chlamydospora | 0 ± 0 | 2.33 ± 2.33 | 0 ± 0 | 0 ± 0 | 0 ± 0 | 0 ± 0 | 0 ± 0 | 0 ± 0 | 0 ± 0 | 0 ± 0 |
| Leptodiscella sp. F277786 | 0.33 ± 0.33 | 0 ± 0 | 0 ± 0 | 0 ± 0 | 0 ± 0 | 2 ± 1 | 0 ± 0 | 0 ± 0 | 0 ± 0 | 3.33 ± 2.4 |
| Leptodiscella sp. FMR 10885 | 1 ± 1 | 1 ± 1 | 15.33 ± 7.69 | 2.67 ± 1.76 | 3 ± 1 | 3 ± 3 | 3.67 ± 0.67 | 0.33 ± 0.33 | 2.33 ± 1.86 | 3 ± 1.15 |
| Leptodontidium obscurum | 0.67 ± 0.67 | 0.67 ± 0.33 | 311.33 ± 64.98 | 7 ± 4.16 | 2 ± 0.58 | 95.33 ± 83.62 | 9 ± 5.51 | 0 ± 0 | 0 ± 0 | 0 ± 0 |
| Leptodontidium sp. | 0.33 ± 0.33 | 36.67 ± 18.35 | 0.33 ± 0.33 | 9 ± 7.51 | 0 ± 0 | 1 ± 1 | 10.67 ± 6.36 | 217 ± 107.57 | 128.33 ± 99.5 | 30.67 ± 15.98 |
| Leptodontidium sp. Sib5-8-1 | 0 ± 0 | 0.67 ± 0.67 | 46.33 ± 43.86 | 1 ± 1 | 0 ± 0 | 20.67 ± 20.17 | 1.33 ± 1.33 | 3.33 ± 1.33 | 4.67 ± 0.33 | 268.33 ± 101.9 |
| Leptosphaeria biglobosa | 0 ± 0 | 4 ± 3.51 | 0 ± 0 | 0 ± 0 | 0.33 ± 0.33 | 0 ± 0 | 0 ± 0 | 0 ± 0 | 0 ± 0 | 0 ± 0 |
| Leptosphaeria sclerotioides | 0 ± 0 | 0 ± 0 | 0 ± 0 | 0 ± 0 | 0 ± 0 | 1 ± 0.58 | 0.33 ± 0.33 | 0 ± 0 | 0 ± 0 | 0 ± 0 |
| Leptosphaeria sp. | 0 ± 0 | 2.33 ± 1.45 | 0 ± 0 | 0 ± 0 | 0 ± 0 | 0 ± 0 | 0.33 ± 0.33 | 0 ± 0 | 0 ± 0 | 0 ± 0 |
| Leptosphaeria sp. EF-32 | 0 ± 0 | 0 ± 0 | 0 ± 0 | 0 ± 0 | 0 ± 0 | 2.33 ± 1.86 | 0 ± 0 | 1 ± 1 | 0 ± 0 | 0 ± 0 |
| Leptosphaeria sp. LA1 | 0 ± 0 | 0 ± 0 | 0 ± 0 | 0.33 ± 0.33 | 0 ± 0 | 0 ± 0 | 0.33 ± 0.33 | 0 ± 0 | 0 ± 0 | 0 ± 0 |
| Leptosphaeria sp. OUCMBI101033 | 0 ± 0 | 0 ± 0 | 0 ± 0 | 0 ± 0 | 0 ± 0 | 0 ± 0 | 0 ± 0 | 0 ± 0 | 0 ± 0 | 0 ± 0 |
| Leptosphaeria sp. RJ-2015 | 0 ± 0 | 0 ± 0 | 0 ± 0 | 7 ± 4.73 | 0.33 ± 0.33 | 8.33 ± 7.36 | 3 ± 2.08 | 0 ± 0 | 0 ± 0 | 0.33 ± 0.33 |
| Leptosphaerulina albulae | 0 ± 0 | 0 ± 0 | 0 ± 0 | 0 ± 0 | 0 ± 0 | 0 ± 0 | 0 ± 0 | 0 ± 0 | 0 ± 0 | 0 ± 0 |
| Leptosphaerulina argentinensis | 25 ± 6.11 | 1586 ± 1059.82 | 5 ± 2.52 | 91 ± 74.01 | 4 ± 2.08 | 38.33 ± 19.81 | 88.33 ± 64.05 | 0 ± 0 | 1 ± 1 | 2.33 ± 1.45 |
| Leucoglossum leucosporum | 7 ± 2.52 | 0 ± 0 | 0.33 ± 0.33 | 0 ± 0 | 9 ± 5.57 | 2.33 ± 2.33 | 0.33 ± 0.33 | 0 ± 0 | 0 ± 0 | 0 ± 0 |
| Leucosporidium intermedium | 0 ± 0 | 11.33 ± 8.51 | 0 ± 0 | 1 ± 1 | 0 ± 0 | 0 ± 0 | 1 ± 1 | 1 ± 1 | 0 ± 0 | 0 ± 0 |
| Leucosporidium yakuticum | 0 ± 0 | 0 ± 0 | 11.33 ± 11.33 | 0 ± 0 | 0 ± 0 | 4.67 ± 4.67 | 0 ± 0 | 0 ± 0 | 0.33 ± 0.33 | 0 ± 0 |
| Liberomyces sp. M36 | 0 ± 0 | 0 ± 0 | 0 ± 0 | 0 ± 0 | 0 ± 0 | 0 ± 0 | 0 ± 0 | 8 ± 4.62 | 1.67 ± 1.67 | 0 ± 0 |
| Linderina macrospora | 0 ± 0 | 0 ± 0 | 2.33 ± 2.33 | 0 ± 0 | 0 ± 0 | 0 ± 0 | 0.33 ± 0.33 | 0 ± 0 | 0 ± 0 | 0 ± 0 |
| Lipomyces doorenjongii | 0 ± 0 | 0 ± 0 | 0.33 ± 0.33 | 2 ± 1.15 | 0 ± 0 | 0.33 ± 0.33 | 2 ± 1.15 | 5 ± 2.89 | 5.33 ± 3.93 | 4.67 ± 3.28 |
| Lomentospora prolificans | 0 ± 0 | 0 ± 0 | 0 ± 0 | 0 ± 0 | 0 ± 0 | 0 ± 0 | 0 ± 0 | 0 ± 0 | 0 ± 0 | 1.33 ± 1.33 |
| Longicollum biappendiculatum | 0 ± 0 | 0 ± 0 | 0 ± 0 | 0 ± 0 | 0 ± 0 | 0.33 ± 0.33 | 0 ± 0 | 0 ± 0 | 0 ± 0 | 0 ± 0 |
| Lopadostoma polynesium | 0 ± 0 | 0 ± 0 | 0 ± 0 | 0 ± 0 | 0 ± 0 | 0 ± 0 | 0 ± 0 | 0 ± 0 | 0 ± 0 | 0 ± 0 |
| Lophiostoma corticola | 0 ± 0 | 0.33 ± 0.33 | 1 ± 1 | 2 ± 1 | 0 ± 0 | 0 ± 0 | 3.33 ± 1.76 | 2.67 ± 2.67 | 2 ± 1 | 0 ± 0 |
| Lophiostoma sp. AK190/05 | 0 ± 0 | 0 ± 0 | 0 ± 0 | 0.33 ± 0.33 | 0 ± 0 | 0 ± 0 | 0.33 ± 0.33 | 0 ± 0 | 0 ± 0 | 0 ± 0 |
| Lophiostoma sp. F135 | 2 ± 1.53 | 0.33 ± 0.33 | 16 ± 5.86 | 2.67 ± 1.76 | 3 ± 1 | 6.67 ± 3.28 | 22 ± 18.56 | 0 ± 0 | 1 ± 0.58 | 3.67 ± 0.67 |
| Lophiostoma sp. Sigrf10 | 0.33 ± 0.33 | 0 ± 0 | 3.67 ± 1.86 | 0.33 ± 0.33 | 1 ± 1 | 0.33 ± 0.33 | 1.33 ± 0.88 | 3.33 ± 1.2 | 0.67 ± 0.33 | 0 ± 0 |
| Lophiotrema sp. 'neohysterioides' | 0.67 ± 0.67 | 0 ± 0 | 0 ± 0 | 0 ± 0 | 0 ± 0 | 0 ± 0 | 0.67 ± 0.67 | 12.33 ± 5.67 | 3 ± 1.73 | 0 ± 0 |
| Lulworthia cf. purpurea FCUL280207CF9 | 7.67 ± 5.17 | 0 ± 0 | 3 ± 3 | 0 ± 0 | 15.33 ± 8.45 | 3.67 ± 3.18 | 0.67 ± 0.67 | 1 ± 1 | 0 ± 0 | 0 ± 0 |
| Lylea tetracoila | 0 ± 0 | 0 ± 0 | 0 ± 0 | 0 ± 0 | 0 ± 0 | 1.67 ± 1.67 | 0 ± 0 | 0 ± 0 | 0 ± 0 | 0 ± 0 |
| Macroconia leptosphaeriae | 0 ± 0 | 0.67 ± 0.67 | 1 ± 1 | 0 ± 0 | 0 ± 0 | 0 ± 0 | 0.67 ± 0.67 | 3 ± 3 | 1.67 ± 0.67 | 0 ± 0 |
| Macrophoma sophoricola | 0 ± 0 | 0 ± 0 | 0 ± 0 | 0 ± 0 | 0 ± 0 | 0.33 ± 0.33 | 0.67 ± 0.67 | 0 ± 0 | 0 ± 0 | 0 ± 0 |
| Macrophomina phaseolina | 0 ± 0 | 1.33 ± 0.88 | 0 ± 0 | 0.33 ± 0.33 | 0 ± 0 | 0 ± 0 | 0 ± 0 | 0 ± 0 | 0 ± 0 | 0 ± 0 |
| Madurella sp. 'pseudomycetomatis' | 0 ± 0 | 0 ± 0 | 0 ± 0 | 0 ± 0 | 0 ± 0 | 2 ± 1.53 | 0 ± 0 | 0 ± 0 | 0 ± 0 | 0 ± 0 |
| Magnaporthiopsis meyeri-festucae | 0 ± 0 | 1 ± 1 | 0 ± 0 | 0 ± 0 | 0 ± 0 | 0 ± 0 | 0 ± 0 | 0 ± 0 | 0 ± 0 | 0 ± 0 |
| Magnibotryascoma mali | 0 ± 0 | 0 ± 0 | 0 ± 0 | 0 ± 0 | 0 ± 0 | 0 ± 0 | 0 ± 0 | 0 ± 0 | 0 ± 0 | 2.67 ± 2.67 |
| Malassezia dermatis | 0 ± 0 | 0 ± 0 | 1.33 ± 0.67 | 0 ± 0 | 0 ± 0 | 0 ± 0 | 0.67 ± 0.67 | 0 ± 0 | 0.67 ± 0.33 | 0.33 ± 0.33 |
| Malassezia globosa | 0 ± 0 | 0 ± 0 | 0.33 ± 0.33 | 0 ± 0 | 0 ± 0 | 0.33 ± 0.33 | 0 ± 0 | 0 ± 0 | 0 ± 0 | 0 ± 0 |
| Malassezia restricta | 0 ± 0 | 0 ± 0 | 0.33 ± 0.33 | 0 ± 0 | 0 ± 0 | 0 ± 0 | 0 ± 0 | 0 ± 0 | 0.67 ± 0.67 | 0.67 ± 0.67 |
| Malassezia sp. 1 MPM-2013 | 0 ± 0 | 0 ± 0 | 0.33 ± 0.33 | 0.67 ± 0.67 | 0 ± 0 | 0.33 ± 0.33 | 2 ± 1.53 | 0 ± 0 | 0 ± 0 | 1.67 ± 1.2 |
| Malbranchea flocciformis | 0 ± 0 | 0 ± 0 | 0 ± 0 | 0 ± 0 | 0 ± 0 | 0 ± 0 | 0 ± 0 | 0 ± 0 | 0 ± 0 | 1.33 ± 0.88 |
| Marasmiellus candidus | 0 ± 0 | 0 ± 0 | 0 ± 0 | 0 ± 0 | 0 ± 0 | 0 ± 0 | 0 ± 0 | 0 ± 0 | 0.33 ± 0.33 | 20 ± 8.14 |
| Marasmius brunneoaurantiacus | 0 ± 0 | 1 ± 1 | 181.67 ± 28.5 | 0.67 ± 0.33 | 0.33 ± 0.33 | 44.33 ± 34.82 | 3 ± 1.73 | 0 ± 0 | 0 ± 0 | 0 ± 0 |
| Marasmius cf. ferrugineus BRNM 724480 | 0 ± 0 | 0.33 ± 0.33 | 0 ± 0 | 0.67 ± 0.67 | 0 ± 0 | 0 ± 0 | 0 ± 0 | 0 ± 0 | 0 ± 0 | 0.67 ± 0.33 |
| Mariannaea elegans | 0.33 ± 0.33 | 63.33 ± 24.26 | 1 ± 0.58 | 13 ± 4.04 | 0.33 ± 0.33 | 14 ± 5.03 | 13.33 ± 8.45 | 5 ± 4.51 | 9.33 ± 5.49 | 24 ± 5.51 |
| Mariannaea pinicola | 0 ± 0 | 7.67 ± 7.17 | 0 ± 0 | 5 ± 2 | 0.33 ± 0.33 | 3.33 ± 1.67 | 2.33 ± 1.86 | 10.67 ± 8.69 | 1.67 ± 0.88 | 7.67 ± 6.17 |
| Mariannaea punicea | 2 ± 1.53 | 49.67 ± 14.33 | 4.67 ± 4.18 | 16.67 ± 7.69 | 7 ± 4.16 | 36.67 ± 20 | 28.33 ± 14.5 | 7.33 ± 2.6 | 3 ± 2.52 | 3.33 ± 1.2 |
| Mariannaea superimposita | 0 ± 0 | 0 ± 0 | 0 ± 0 | 0 ± 0 | 0 ± 0 | 0 ± 0 | 1.67 ± 1.67 | 25.33 ± 16.76 | 7 ± 6.51 | 0.33 ± 0.33 |
| Martininia panamaensis | 0 ± 0 | 0 ± 0 | 0.33 ± 0.33 | 0 ± 0 | 0 ± 0 | 2 ± 2 | 0 ± 0 | 0 ± 0 | 0 ± 0 | 0 ± 0 |
| Massarina rubi | 2.67 ± 1.2 | 0.33 ± 0.33 | 0 ± 0 | 3.67 ± 0.67 | 27 ± 1.15 | 20.33 ± 3.67 | 39.33 ± 27.49 | 3.33 ± 2.33 | 5.67 ± 2.85 | 6.67 ± 1.76 |
| Matsushimaea monilioides | 0 ± 0 | 0 ± 0 | 0 ± 0 | 0 ± 0 | 0 ± 0 | 0 ± 0 | 0 ± 0 | 0 ± 0 | 0 ± 0 | 3.33 ± 2.85 |
| Medicopsis romeroi | 0 ± 0 | 0 ± 0 | 0 ± 0 | 0 ± 0 | 0 ± 0 | 0 ± 0 | 2 ± 2 | 0.33 ± 0.33 | 0 ± 0 | 0 ± 0 |
| Melanconiella elegans | 11.33 ± 7.36 | 50.67 ± 35.47 | 1 ± 0.58 | 4.67 ± 3.67 | 5.33 ± 2.85 | 2.67 ± 1.67 | 26 ± 13.8 | 9 ± 4.51 | 5 ± 3.61 | 13.33 ± 8.35 |
| Melanocarpus albomyces | 0 ± 0 | 0 ± 0 | 0 ± 0 | 0 ± 0 | 0.33 ± 0.33 | 0 ± 0 | 0 ± 0 | 0 ± 0 | 0 ± 0 | 0 ± 0 |
| Melanocucurbitaria uzbekistanica | 0 ± 0 | 0 ± 0 | 0 ± 0 | 0 ± 0 | 0 ± 0 | 0 ± 0 | 1 ± 1 | 0 ± 0 | 0 ± 0 | 0 ± 0 |
| Melanomma sanguinarium | 0 ± 0 | 0 ± 0 | 0 ± 0 | 0 ± 0 | 0 ± 0 | 0 ± 0 | 2 ± 2 | 0 ± 0 | 0 ± 0 | 0 ± 0 |
| Melanophyllum haematospermum | 0 ± 0 | 0 ± 0 | 0 ± 0 | 0 ± 0 | 0.33 ± 0.33 | 0 ± 0 | 2.33 ± 2.33 | 0 ± 0 | 0.33 ± 0.33 | 0 ± 0 |
| Meliniomyces sp. ECRU075 | 0.33 ± 0.33 | 11.67 ± 9.21 | 10 ± 5.13 | 3.67 ± 2.19 | 0 ± 0 | 1.33 ± 0.88 | 1.67 ± 1.2 | 0.33 ± 0.33 | 0 ± 0 | 34.67 ± 29.34 |
| Memnoniella dichroa | 1 ± 0.58 | 0 ± 0 | 0 ± 0 | 0.33 ± 0.33 | 2.67 ± 0.88 | 13.33 ± 9.21 | 0 ± 0 | 0 ± 0 | 0 ± 0 | 0.33 ± 0.33 |
| Memnoniella ellipsoidea | 2 ± 2 | 0 ± 0 | 0 ± 0 | 0 ± 0 | 2.67 ± 2.19 | 0 ± 0 | 2 ± 1.53 | 3.67 ± 3.18 | 0 ± 0 | 19.67 ± 16.29 |
| Memnoniella longistipitata | 0 ± 0 | 0 ± 0 | 0.33 ± 0.33 | 0 ± 0 | 1.33 ± 1.33 | 0 ± 0 | 3.33 ± 3.33 | 0.33 ± 0.33 | 2.33 ± 2.33 | 0 ± 0 |
| Memnoniella pseudonilagirica | 0 ± 0 | 0 ± 0 | 0 ± 0 | 0 ± 0 | 0 ± 0 | 3 ± 2.52 | 0.33 ± 0.33 | 0 ± 0 | 0 ± 0 | 0 ± 0 |
| Memnoniella sp. MUCL 50191 | 4.67 ± 1.2 | 0 ± 0 | 0.33 ± 0.33 | 1.67 ± 1.67 | 149.33 ± 23.85 | 4.33 ± 2.96 | 6.33 ± 4.48 | 0 ± 0 | 0 ± 0 | 0 ± 0 |
| Merimbla ingelheimensis | 0 ± 0 | 0 ± 0 | 0 ± 0 | 0 ± 0 | 0 ± 0 | 0 ± 0 | 0 ± 0 | 0 ± 0 | 0 ± 0 | 0.67 ± 0.67 |
| Metapochonia bulbillosa | 0 ± 0 | 0.67 ± 0.67 | 0 ± 0 | 0.33 ± 0.33 | 0 ± 0 | 0.33 ± 0.33 | 1 ± 1 | 0.67 ± 0.67 | 0 ± 0 | 0 ± 0 |
| Metapochonia suchlasporia | 0 ± 0 | 0 ± 0 | 0.67 ± 0.33 | 0 ± 0 | 0.33 ± 0.33 | 6 ± 3.79 | 15.67 ± 13.72 | 8.67 ± 6.33 | 7.33 ± 6.84 | 0.67 ± 0.67 |
| Metarhizium anisopliae | 1 ± 0.58 | 1 ± 1 | 7.33 ± 3.84 | 48.33 ± 46.33 | 1 ± 0.58 | 139.33 ± 82.52 | 56 ± 33.56 | 0.33 ± 0.33 | 0.33 ± 0.33 | 27 ± 20.5 |
| Metarhizium carneum | 0.67 ± 0.67 | 21.33 ± 16.5 | 9 ± 4.04 | 2.33 ± 0.88 | 0.67 ± 0.67 | 8 ± 2.08 | 8.67 ± 4.81 | 6.33 ± 3.28 | 0.67 ± 0.67 | 1.67 ± 0.88 |
| Metarhizium granulomatis | 0 ± 0 | 0 ± 0 | 0 ± 0 | 0 ± 0 | 0 ± 0 | 0 ± 0 | 1 ± 1 | 0 ± 0 | 0 ± 0 | 0 ± 0 |
| Metarhizium marquandii | 62.33 ± 18.84 | 9 ± 5 | 778.33 ± 479.19 | 85.33 ± 13.87 | 36.33 ± 3.28 | 294.67 ± 10.48 | 151.33 ± 22.66 | 357.67 ± 198.61 | 155 ± 134.69 | 563 ± 190.89 |
| Metarhizium rileyi | 0 ± 0 | 0 ± 0 | 0 ± 0 | 0 ± 0 | 0 ± 0 | 0.33 ± 0.33 | 0.67 ± 0.67 | 0 ± 0 | 0 ± 0 | 0 ± 0 |
| Metarhizium sp. | 5 ± 1.53 | 0 ± 0 | 18 ± 2.89 | 2.67 ± 1.33 | 47 ± 16.2 | 158.33 ± 83.59 | 41.33 ± 30.42 | 138.67 ± 63.84 | 92.67 ± 89.19 | 49.33 ± 14.38 |
| Metarhizium sp. CW-2015 | 0 ± 0 | 0 ± 0 | 0 ± 0 | 0 ± 0 | 0 ± 0 | 0 ± 0 | 1 ± 0.58 | 0 ± 0 | 0 ± 0 | 3.33 ± 3.33 |
| Metarhizium sp. Ngs5-2 | 0 ± 0 | 0 ± 0 | 0.33 ± 0.33 | 0 ± 0 | 0 ± 0 | 1 ± 1 | 0 ± 0 | 0 ± 0 | 0 ± 0 | 0 ± 0 |
| Microascus cirrosus | 0 ± 0 | 0 ± 0 | 0 ± 0 | 0 ± 0 | 0 ± 0 | 0 ± 0 | 2.33 ± 2.33 | 0 ± 0 | 0 ± 0 | 0 ± 0 |
| Microascus desmosporus | 1 ± 0.58 | 1.67 ± 1.2 | 1.33 ± 1.33 | 0 ± 0 | 0.33 ± 0.33 | 0.67 ± 0.33 | 1.33 ± 1.33 | 0 ± 0 | 0 ± 0 | 0 ± 0 |
| Microcera coccophila | 0 ± 0 | 0 ± 0 | 0 ± 0 | 0 ± 0 | 0 ± 0 | 0 ± 0 | 0 ± 0 | 0 ± 0 | 0 ± 0 | 1.33 ± 0.88 |
| Microcera larvarum | 0 ± 0 | 0 ± 0 | 0 ± 0 | 0 ± 0 | 0 ± 0 | 0 ± 0 | 0.33 ± 0.33 | 0 ± 0 | 0 ± 0 | 2 ± 1.53 |
| Microdiplodia sp. | 1.67 ± 0.88 | 0 ± 0 | 91.67 ± 36.71 | 7 ± 3.21 | 3 ± 1 | 31.67 ± 24.17 | 11 ± 8.19 | 0 ± 0 | 0 ± 0 | 1 ± 1 |
| Microdiplodia sp. TS-2016 | 0 ± 0 | 0 ± 0 | 2 ± 2 | 0 ± 0 | 0.33 ± 0.33 | 0.33 ± 0.33 | 0 ± 0 | 0 ± 0 | 0 ± 0 | 0 ± 0 |
| Microdochium fisheri | 0 ± 0 | 0 ± 0 | 0 ± 0 | 0 ± 0 | 0 ± 0 | 0 ± 0 | 0 ± 0 | 2.33 ± 2.33 | 6.33 ± 6.33 | 0 ± 0 |
| Microdochium musae | 0 ± 0 | 0 ± 0 | 0 ± 0 | 0.67 ± 0.67 | 0 ± 0 | 0 ± 0 | 1 ± 1 | 0 ± 0 | 0 ± 0 | 0 ± 0 |
| Microdochium nivale | 2 ± 1 | 0 ± 0 | 0 ± 0 | 0 ± 0 | 0.33 ± 0.33 | 0 ± 0 | 0 ± 0 | 0 ± 0 | 0 ± 0 | 0 ± 0 |
| Microdochium sp. | 0 ± 0 | 0 ± 0 | 0 ± 0 | 0 ± 0 | 0 ± 0 | 0 ± 0 | 0 ± 0 | 0.33 ± 0.33 | 0 ± 0 | 0 ± 0 |
| Microdochium sp. KS00012 | 0.33 ± 0.33 | 0 ± 0 | 14.33 ± 9.06 | 0.33 ± 0.33 | 3 ± 2.52 | 3 ± 1.53 | 0.67 ± 0.67 | 0 ± 0 | 0 ± 0 | 0 ± 0 |
| Microdochium sp. wb587 | 0 ± 0 | 0 ± 0 | 0 ± 0 | 0 ± 0 | 8.67 ± 5.17 | 0 ± 0 | 1 ± 0.58 | 5 ± 2.52 | 12.33 ± 12.33 | 1.67 ± 1.67 |
| Micronematobotrys verrucosus | 0.67 ± 0.67 | 0 ± 0 | 0 ± 0 | 0 ± 0 | 0 ± 0 | 0 ± 0 | 0 ± 0 | 0 ± 0 | 0 ± 0 | 0 ± 0 |
| Microscypha sp. S-28 | 2 ± 1.53 | 0.33 ± 0.33 | 0 ± 0 | 0 ± 0 | 0.67 ± 0.67 | 0 ± 0 | 0 ± 0 | 0 ± 0 | 0 ± 0 | 0 ± 0 |
| Microsphaeropsis olivacea | 0 ± 0 | 0 ± 0 | 15.67 ± 7.8 | 0 ± 0 | 0 ± 0 | 15.67 ± 10.68 | 1 ± 0.58 | 0 ± 0 | 0 ± 0 | 0.33 ± 0.33 |
| Microsphaeropsis proteae | 0.67 ± 0.67 | 0 ± 0 | 43 ± 12.66 | 0 ± 0 | 1.67 ± 0.67 | 102.33 ± 81.46 | 7.67 ± 3.18 | 0 ± 0 | 0.33 ± 0.33 | 11.33 ± 3.93 |
| Microthelia verruculosa | 0 ± 0 | 0 ± 0 | 0 ± 0 | 0 ± 0 | 0 ± 0 | 5 ± 4.51 | 0.33 ± 0.33 | 0 ± 0 | 0 ± 0 | 0 ± 0 |
| Miniancora allisoniensis | 0 ± 0 | 0.33 ± 0.33 | 0 ± 0 | 0 ± 0 | 0 ± 0 | 0 ± 0 | 0 ± 0 | 0.67 ± 0.67 | 0.67 ± 0.67 | 0 ± 0 |
| Minimedusa polyspora | 0 ± 0 | 0.33 ± 0.33 | 0.33 ± 0.33 | 0.33 ± 0.33 | 1.33 ± 0.67 | 17 ± 13.53 | 2.67 ± 1.33 | 0 ± 0 | 0 ± 0 | 1.67 ± 1.67 |
| Minimelanolocus asiaticus | 0 ± 0 | 6.67 ± 2.96 | 0 ± 0 | 0.33 ± 0.33 | 0 ± 0 | 0.67 ± 0.67 | 1 ± 1 | 0 ± 0 | 1.67 ± 0.88 | 0 ± 0 |
| Minimelanolocus curvatus | 0.33 ± 0.33 | 0 ± 0 | 0 ± 0 | 2 ± 1.53 | 0 ± 0 | 0 ± 0 | 1 ± 0.58 | 0.33 ± 0.33 | 0 ± 0 | 0 ± 0 |
| Minimidochium sp. TMS-2011 | 0 ± 0 | 5.33 ± 3.33 | 0 ± 0 | 0 ± 0 | 0 ± 0 | 0 ± 0 | 1.33 ± 1.33 | 0 ± 0 | 0 ± 0 | 0 ± 0 |
| Minutisphaera aspera | 0 ± 0 | 0 ± 0 | 0 ± 0 | 0 ± 0 | 0 ± 0 | 0 ± 0 | 0 ± 0 | 0 ± 0 | 0 ± 0 | 0 ± 0 |
| Mirandina sp. 'breviphora' | 2 ± 2 | 1 ± 1 | 93.67 ± 44.29 | 3.33 ± 1.33 | 4.67 ± 2.33 | 13.67 ± 7.22 | 5.33 ± 2.4 | 60 ± 37.51 | 153.67 ± 153.17 | 15 ± 14.01 |
| Modicella malleola | 0 ± 0 | 0 ± 0 | 0 ± 0 | 1 ± 1 | 0 ± 0 | 0 ± 0 | 0 ± 0 | 0 ± 0 | 0 ± 0 | 0 ± 0 |
| Modicella reniformis | 0 ± 0 | 0 ± 0 | 0 ± 0 | 0 ± 0 | 0 ± 0 | 0 ± 0 | 0.67 ± 0.67 | 0 ± 0 | 0 ± 0 | 0 ± 0 |
| Moesziomyces antarcticus | 0 ± 0 | 0 ± 0 | 0 ± 0 | 0 ± 0 | 0 ± 0 | 0 ± 0 | 0 ± 0 | 0 ± 0 | 0 ± 0 | 3.67 ± 3.67 |
| Mollisia sp. | 0 ± 0 | 0 ± 0 | 0 ± 0 | 0 ± 0 | 0 ± 0 | 1.33 ± 1.33 | 0 ± 0 | 5.33 ± 2.91 | 2.67 ± 0.88 | 5.67 ± 0.33 |
| Mollisina uncinata | 0 ± 0 | 0 ± 0 | 0 ± 0 | 0 ± 0 | 0 ± 0 | 0 ± 0 | 0 ± 0 | 0 ± 0 | 0 ± 0 | 0 ± 0 |
| Monacrosporium megalosporum | 0 ± 0 | 2 ± 1 | 0.33 ± 0.33 | 0 ± 0 | 0.33 ± 0.33 | 0.67 ± 0.67 | 0 ± 0 | 2.67 ± 0.88 | 0.33 ± 0.33 | 0.67 ± 0.33 |
| Monilinia sp. | 0 ± 0 | 0.67 ± 0.67 | 0 ± 0 | 0 ± 0 | 0 ± 0 | 0 ± 0 | 0 ± 0 | 0 ± 0 | 0 ± 0 | 0 ± 0 |
| Monocillium bulbillosum | 0 ± 0 | 0 ± 0 | 0 ± 0 | 0.33 ± 0.33 | 0 ± 0 | 0 ± 0 | 0.67 ± 0.67 | 0 ± 0 | 0 ± 0 | 0 ± 0 |
| Monocillium mucidum | 0 ± 0 | 0 ± 0 | 0 ± 0 | 0 ± 0 | 0 ± 0 | 4.33 ± 4.33 | 0 ± 0 | 0.33 ± 0.33 | 0.33 ± 0.33 | 0 ± 0 |
| Monocillium sp. | 0 ± 0 | 0 ± 0 | 0 ± 0 | 0.33 ± 0.33 | 0 ± 0 | 0 ± 0 | 0.33 ± 0.33 | 12.67 ± 6.74 | 4 ± 4 | 0 ± 0 |
| Monocillium sp. BGE-2018c | 0 ± 0 | 0 ± 0 | 3 ± 3 | 23.67 ± 8.97 | 0 ± 0 | 8.33 ± 3.38 | 10.33 ± 7.13 | 0 ± 0 | 0 ± 0 | 0.67 ± 0.67 |
| Monodictys castaneae | 0 ± 0 | 0 ± 0 | 0 ± 0 | 0 ± 0 | 0 ± 0 | 0 ± 0 | 0 ± 0 | 0 ± 0 | 0 ± 0 | 17.33 ± 17.33 |
| Monodictys sp. | 0.33 ± 0.33 | 0 ± 0 | 0 ± 0 | 0 ± 0 | 7.33 ± 1.86 | 0 ± 0 | 3.33 ± 3.33 | 0 ± 0 | 0 ± 0 | 0 ± 0 |
| Monodictys sp. 1 JAS-2013 | 0 ± 0 | 0 ± 0 | 5.67 ± 5.67 | 0 ± 0 | 1.33 ± 0.67 | 0.33 ± 0.33 | 0 ± 0 | 0 ± 0 | 0 ± 0 | 0 ± 0 |
| Monosporascus cannonballus | 0 ± 0 | 0 ± 0 | 1.33 ± 1.33 | 0.33 ± 0.33 | 0 ± 0 | 0 ± 0 | 1 ± 0.58 | 0 ± 0 | 0 ± 0 | 0 ± 0 |
| Monosporascus ibericus | 0 ± 0 | 0 ± 0 | 0 ± 0 | 0 ± 0 | 0.33 ± 0.33 | 0 ± 0 | 0 ± 0 | 0 ± 0 | 0 ± 0 | 0 ± 0 |
| Montagnula scabiosae | 0 ± 0 | 0 ± 0 | 0 ± 0 | 0 ± 0 | 0 ± 0 | 0 ± 0 | 0.33 ± 0.33 | 0.67 ± 0.67 | 0.33 ± 0.33 | 0.67 ± 0.67 |
| Mortierella aff. gamsii | 0.33 ± 0.33 | 0 ± 0 | 2.67 ± 0.88 | 0.67 ± 0.33 | 2.67 ± 1.2 | 16.33 ± 6.36 | 6.67 ± 1.86 | 2 ± 0.58 | 0.33 ± 0.33 | 0.33 ± 0.33 |
| Mortierella alpina | 3.67 ± 0.67 | 1 ± 0.58 | 28.67 ± 5.49 | 5 ± 1.53 | 24.33 ± 3.84 | 209.67 ± 28.18 | 333.33 ± 281.37 | 852.67 ± 445.54 | 352.33 ± 333.39 | 42.33 ± 10.48 |
| Mortierella ambigua | 0 ± 0 | 0 ± 0 | 0 ± 0 | 0 ± 0 | 0 ± 0 | 0 ± 0 | 0 ± 0 | 0 ± 0 | 0 ± 0 | 0 ± 0 |
| Mortierella beljakovae | 2 ± 0.58 | 0 ± 0 | 0 ± 0 | 0 ± 0 | 0 ± 0 | 0 ± 0 | 0 ± 0 | 1.33 ± 1.33 | 1.33 ± 1.33 | 2.67 ± 2.67 |
| Mortierella calciphila | 0 ± 0 | 23.67 ± 22.17 | 0 ± 0 | 24.67 ± 15.38 | 0.33 ± 0.33 | 0 ± 0 | 7 ± 5.57 | 20 ± 18.5 | 2.33 ± 1.45 | 5 ± 1.73 |
| Mortierella capitata | 0 ± 0 | 0.33 ± 0.33 | 0 ± 0 | 0.33 ± 0.33 | 0 ± 0 | 0.67 ± 0.67 | 0 ± 0 | 0.33 ± 0.33 | 0 ± 0 | 2.67 ± 2.67 |
| Mortierella cystojenkinii | 0 ± 0 | 0 ± 0 | 0 ± 0 | 0.67 ± 0.67 | 0 ± 0 | 0 ± 0 | 0 ± 0 | 0 ± 0 | 0 ± 0 | 6 ± 3.21 |
| Mortierella echinula | 0 ± 0 | 0 ± 0 | 0 ± 0 | 0 ± 0 | 0 ± 0 | 0 ± 0 | 0 ± 0 | 0 ± 0 | 1 ± 1 | 0 ± 0 |
| Mortierella elongatula | 0 ± 0 | 0 ± 0 | 0 ± 0 | 0 ± 0 | 0 ± 0 | 1.67 ± 1.67 | 0 ± 0 | 0 ± 0 | 5 ± 5 | 0 ± 0 |
| Mortierella exigua | 0.33 ± 0.33 | 0 ± 0 | 0.33 ± 0.33 | 1.67 ± 1.67 | 6 ± 3.06 | 122.33 ± 57.14 | 9.33 ± 4.7 | 0 ± 0 | 0 ± 0 | 5 ± 5 |
| Mortierella fimbricystis | 0 ± 0 | 0 ± 0 | 0 ± 0 | 0 ± 0 | 0 ± 0 | 3.67 ± 3.67 | 0 ± 0 | 0 ± 0 | 0 ± 0 | 0 ± 0 |
| Mortierella gamsii | 0 ± 0 | 0 ± 0 | 0.33 ± 0.33 | 0 ± 0 | 0 ± 0 | 0 ± 0 | 0 ± 0 | 0 ± 0 | 0 ± 0 | 0 ± 0 |
| Mortierella gemmifera | 0 ± 0 | 0.33 ± 0.33 | 0 ± 0 | 3.33 ± 2.4 | 0 ± 0 | 0 ± 0 | 1.33 ± 0.88 | 0 ± 0 | 0 ± 0 | 0.67 ± 0.67 |
| Mortierella hyalina | 0 ± 0 | 0 ± 0 | 0 ± 0 | 0 ± 0 | 0 ± 0 | 1.33 ± 1.33 | 0.33 ± 0.33 | 11.67 ± 11.67 | 0 ± 0 | 6 ± 6 |
| Mortierella indohii | 0 ± 0 | 0 ± 0 | 0 ± 0 | 0.33 ± 0.33 | 0 ± 0 | 0 ± 0 | 0 ± 0 | 0 ± 0 | 0.67 ± 0.67 | 0 ± 0 |
| Mortierella jenkinii | 0 ± 0 | 0 ± 0 | 0 ± 0 | 0 ± 0 | 0 ± 0 | 0 ± 0 | 0 ± 0 | 0 ± 0 | 0 ± 0 | 0 ± 0 |
| Mortierella lignicola | 0 ± 0 | 5 ± 5 | 0 ± 0 | 0.33 ± 0.33 | 0.33 ± 0.33 | 0 ± 0 | 1 ± 1 | 0 ± 0 | 0 ± 0 | 0 ± 0 |
| Mortierella sp. | 14.67 ± 6.12 | 0.33 ± 0.33 | 194 ± 58.21 | 60.67 ± 34.28 | 11.67 ± 0.67 | 346 ± 59.79 | 107 ± 15.95 | 408 ± 187.55 | 774 ± 720.11 | 137.67 ± 46.77 |
| Mortierella sp. 02NH02 | 14 ± 1.15 | 3 ± 0.58 | 539.33 ± 60.42 | 13.33 ± 5.81 | 19.33 ± 5.24 | 203.67 ± 62.71 | 172 ± 69.94 | 195 ± 116.09 | 102 ± 95.51 | 16 ± 5.51 |
| Mortierella sp. 06VT02 | 0 ± 0 | 0 ± 0 | 0 ± 0 | 0 ± 0 | 0 ± 0 | 0 ± 0 | 0 ± 0 | 0.33 ± 0.33 | 9.33 ± 9.33 | 0 ± 0 |
| Mortierella sp. 17WV07 | 0 ± 0 | 0.67 ± 0.67 | 2.33 ± 1.2 | 4.67 ± 2.33 | 6.33 ± 0.88 | 43 ± 29.54 | 56 ± 43 | 1 ± 0.58 | 0.33 ± 0.33 | 2.33 ± 1.33 |
| Mortierella sp. CBS 118520 | 0 ± 0 | 0 ± 0 | 0 ± 0 | 0 ± 0 | 0 ± 0 | 0.67 ± 0.67 | 0 ± 0 | 0 ± 0 | 0 ± 0 | 0 ± 0 |
| Mortierella sp. CF15 | 76 ± 66.01 | 1.33 ± 1.33 | 0 ± 0 | 16 ± 10.07 | 5 ± 2.52 | 1.33 ± 0.88 | 5.33 ± 3.18 | 29 ± 17.04 | 22.33 ± 20.34 | 34.33 ± 24.44 |
| Mortierella sp. D3Wa | 57.33 ± 7.26 | 56 ± 19.55 | 927.67 ± 186.38 | 1281.33 ± 785.58 | 367.67 ± 103.6 | 3878.67 ± 1375.1 | 1885 ± 524.54 | 25 ± 6.24 | 53.67 ± 20.76 | 875 ± 209.93 |
| Mortierella sp. FMR13-7 | 0 ± 0 | 0 ± 0 | 0 ± 0 | 0 ± 0 | 0 ± 0 | 0.33 ± 0.33 | 0 ± 0 | 0 ± 0 | 0 ± 0 | 0 ± 0 |
| Mortierella sp. FMR23-12 | 0 ± 0 | 0 ± 0 | 0 ± 0 | 1 ± 1 | 0 ± 0 | 0 ± 0 | 0 ± 0 | 6 ± 5.03 | 7.67 ± 7.67 | 0 ± 0 |
| Mortierella sp. FSU 10557 | 0 ± 0 | 0 ± 0 | 0 ± 0 | 0.33 ± 0.33 | 0 ± 0 | 0 ± 0 | 0 ± 0 | 0 ± 0 | 0 ± 0 | 0 ± 0 |
| Mortierella sp. GW_OTU27 | 0 ± 0 | 17 ± 11.5 | 0 ± 0 | 0.33 ± 0.33 | 0 ± 0 | 0 ± 0 | 4 ± 3.06 | 0 ± 0 | 0 ± 0 | 0 ± 0 |
| Mortierella sp. JCM 28527 | 0 ± 0 | 8.33 ± 0.33 | 0 ± 0 | 0 ± 0 | 0 ± 0 | 0.67 ± 0.67 | 1.33 ± 0.67 | 0 ± 0 | 1 ± 0.58 | 0.33 ± 0.33 |
| Mortierella sp. MEL 2385001 | 10.33 ± 0.88 | 1015.33 ± 143.65 | 673 ± 206.6 | 257 ± 76.58 | 8.67 ± 2.73 | 250.33 ± 91.54 | 351.67 ± 119.51 | 100 ± 17.35 | 170 ± 54.84 | 1403.67 ± 339.72 |
| Mortierella sp. OTU094 AN-2016 | 1 ± 1 | 42 ± 8.02 | 28.33 ± 6.06 | 13.67 ± 4.91 | 0 ± 0 | 21 ± 6.56 | 17.33 ± 8.25 | 6.33 ± 1.45 | 7 ± 4.16 | 106 ± 38.97 |
| Mortierella sp. S-24 | 0 ± 0 | 0 ± 0 | 0 ± 0 | 0 ± 0 | 0 ± 0 | 0.67 ± 0.67 | 0 ± 0 | 0 ± 0 | 0 ± 0 | 13 ± 13 |
| Mortierella sp. S-25 | 0 ± 0 | 0 ± 0 | 0 ± 0 | 0 ± 0 | 0 ± 0 | 0.67 ± 0.67 | 0 ± 0 | 0 ± 0 | 0 ± 0 | 2.33 ± 2.33 |
| Mortierella sp. SA1-3 | 0 ± 0 | 0 ± 0 | 0 ± 0 | 1 ± 0.58 | 0 ± 0 | 1 ± 0.58 | 1.33 ± 0.67 | 0 ± 0 | 0 ± 0 | 2.33 ± 2.33 |
| Mortierella sp. T12 | 11.67 ± 2.03 | 1 ± 0.58 | 276 ± 9.85 | 13.67 ± 4.18 | 61.67 ± 6.17 | 378.33 ± 39.07 | 90.67 ± 14.25 | 24.33 ± 11.2 | 12.33 ± 10.33 | 47 ± 8.72 |
| Mortierella sp. TC60c | 2.33 ± 0.67 | 144 ± 88.91 | 0.33 ± 0.33 | 13.33 ± 6.01 | 0.33 ± 0.33 | 14.33 ± 12.84 | 310.67 ± 265.39 | 9.33 ± 4.41 | 2.33 ± 0.88 | 3.33 ± 1.45 |
| Mortierella sp. TR065 | 0 ± 0 | 1 ± 0.58 | 46.33 ± 5.78 | 14.33 ± 9.4 | 1.67 ± 0.67 | 23.33 ± 15.06 | 18.67 ± 7.06 | 0.67 ± 0.33 | 0 ± 0 | 11.33 ± 2.33 |
| Mortierella sp. TUFC 20030 | 0 ± 0 | 0 ± 0 | 0 ± 0 | 0.33 ± 0.33 | 1.67 ± 1.67 | 0.33 ± 0.33 | 0.67 ± 0.33 | 8.67 ± 6.33 | 15.67 ± 9.91 | 50 ± 47.52 |
| Mortierella sp. TUFC 20053 | 0 ± 0 | 0 ± 0 | 0.33 ± 0.33 | 0.33 ± 0.33 | 0 ± 0 | 0 ± 0 | 0 ± 0 | 0 ± 0 | 0 ± 0 | 0 ± 0 |
| Mortierella strangulata | 0 ± 0 | 0 ± 0 | 2.67 ± 1.33 | 0 ± 0 | 0 ± 0 | 0 ± 0 | 0 ± 0 | 0 ± 0 | 0 ± 0 | 0 ± 0 |
| Mortierella verticillata | 0 ± 0 | 0.33 ± 0.33 | 3 ± 1.73 | 16.67 ± 12.78 | 0 ± 0 | 0.67 ± 0.33 | 6 ± 4.51 | 1.33 ± 0.88 | 3.33 ± 0.33 | 6.67 ± 3.71 |
| Mortierella wolfii | 0 ± 0 | 0 ± 0 | 0.33 ± 0.33 | 0 ± 0 | 0 ± 0 | 2.33 ± 1.2 | 0 ± 0 | 0 ± 0 | 0 ± 0 | 1 ± 1 |
| Mortierella zychae | 0 ± 0 | 0 ± 0 | 0 ± 0 | 0 ± 0 | 0 ± 0 | 0 ± 0 | 0 ± 0 | 15.33 ± 14.34 | 0 ± 0 | 0 ± 0 |
| Mrakia aquatica | 0.33 ± 0.33 | 0 ± 0 | 29.67 ± 12.99 | 0 ± 0 | 0.67 ± 0.67 | 11 ± 9.5 | 2.33 ± 0.88 | 1.67 ± 1.67 | 0.33 ± 0.33 | 2.33 ± 2.33 |
| Mrakia gelida | 0.67 ± 0.33 | 1 ± 1 | 4 ± 3.51 | 0.33 ± 0.33 | 0.67 ± 0.67 | 10.33 ± 7.88 | 0.67 ± 0.67 | 26.67 ± 24.21 | 0 ± 0 | 0.33 ± 0.33 |
| Muscinupta laevis | 0 ± 0 | 0 ± 0 | 0.33 ± 0.33 | 0 ± 0 | 0 ± 0 | 0.33 ± 0.33 | 0 ± 0 | 8 ± 7.51 | 0.67 ± 0.67 | 0.67 ± 0.33 |
| Muscodor sp. N190 | 0 ± 0 | 0 ± 0 | 0 ± 0 | 0 ± 0 | 0 ± 0 | 0 ± 0 | 0 ± 0 | 0.33 ± 0.33 | 0.67 ± 0.67 | 0 ± 0 |
| Musicillium theobromae | 0 ± 0 | 0 ± 0 | 2 ± 2 | 0.33 ± 0.33 | 0 ± 0 | 0 ± 0 | 0.33 ± 0.33 | 0 ± 0 | 0 ± 0 | 1.67 ± 1.67 |
| Myceliophthora lutea | 0 ± 0 | 0 ± 0 | 0 ± 0 | 0 ± 0 | 0 ± 0 | 0 ± 0 | 0 ± 0 | 0 ± 0 | 0 ± 0 | 0 ± 0 |
| Mycena maurella | 0 ± 0 | 0 ± 0 | 0 ± 0 | 0 ± 0 | 0 ± 0 | 0 ± 0 | 0 ± 0 | 0 ± 0 | 0.67 ± 0.67 | 14.67 ± 13.68 |
| Mycena oregonensis | 0 ± 0 | 0 ± 0 | 2.67 ± 2.67 | 0 ± 0 | 0 ± 0 | 0 ± 0 | 0.67 ± 0.67 | 0 ± 0 | 0 ± 0 | 0 ± 0 |
| Mycena sp. 2 KO-2013 | 0.67 ± 0.33 | 10.33 ± 10.33 | 0 ± 0 | 2 ± 1.53 | 0 ± 0 | 0 ± 0 | 0.33 ± 0.33 | 0 ± 0 | 0 ± 0 | 0 ± 0 |
| Mycena sp. YO2-12 | 0 ± 0 | 0.33 ± 0.33 | 0 ± 0 | 2 ± 1 | 0 ± 0 | 0 ± 0 | 0.33 ± 0.33 | 0 ± 0 | 0 ± 0 | 0 ± 0 |
| Mycena stylobates | 0 ± 0 | 2.33 ± 1.86 | 0 ± 0 | 0 ± 0 | 0 ± 0 | 0 ± 0 | 0 ± 0 | 0 ± 0 | 0 ± 0 | 0 ± 0 |
| Mycocentrospora sp. | 0 ± 0 | 0 ± 0 | 0.67 ± 0.67 | 8.33 ± 6.84 | 0 ± 0 | 0 ± 0 | 2.67 ± 2.67 | 0 ± 0 | 0 ± 0 | 0 ± 0 |
| Mycogone perniciosa | 0 ± 0 | 0 ± 0 | 0 ± 0 | 0 ± 0 | 0 ± 0 | 4.33 ± 4.33 | 0 ± 0 | 0 ± 0 | 0 ± 0 | 0 ± 0 |
| Mycoleptodiscus terrestris | 0 ± 0 | 0.67 ± 0.67 | 4.33 ± 1.76 | 1.67 ± 1.67 | 0 ± 0 | 0.67 ± 0.67 | 1 ± 1 | 0 ± 0 | 0 ± 0 | 0 ± 0 |
| Mycosphaerella sp. CBS 208.94 | 0 ± 0 | 0 ± 0 | 0 ± 0 | 1.33 ± 1.33 | 0 ± 0 | 0 ± 0 | 3.33 ± 0.67 | 0 ± 0 | 0 ± 0 | 2 ± 1 |
| Mycosphaerella sp. MUCC574 | 1.67 ± 1.67 | 0.33 ± 0.33 | 1.67 ± 1.2 | 0.33 ± 0.33 | 1 ± 1 | 0 ± 0 | 2.33 ± 0.33 | 0 ± 0 | 0.33 ± 0.33 | 0 ± 0 |
| Mycosphaerella sp. PDD 105250 | 0.33 ± 0.33 | 0 ± 0 | 0.33 ± 0.33 | 0 ± 0 | 0.33 ± 0.33 | 0.67 ± 0.67 | 0 ± 0 | 16.67 ± 15.19 | 3.67 ± 3.18 | 0 ± 0 |
| Mycosphaerella sp. soF2 | 0 ± 0 | 0 ± 0 | 0 ± 0 | 0 ± 0 | 0 ± 0 | 0.33 ± 0.33 | 2.67 ± 2.67 | 0 ± 0 | 0 ± 0 | 0 ± 0 |
| Mycothermus thermophilus | 0 ± 0 | 0 ± 0 | 0 ± 0 | 0 ± 0 | 0 ± 0 | 6.33 ± 4.91 | 1.33 ± 0.88 | 0 ± 0 | 0 ± 0 | 2.67 ± 2.67 |
| Myriangium duriaei | 0 ± 0 | 0 ± 0 | 0 ± 0 | 0 ± 0 | 0 ± 0 | 0 ± 0 | 0 ± 0 | 0 ± 0 | 0 ± 0 | 0.33 ± 0.33 |
| Myriococcum praecox | 0 ± 0 | 0 ± 0 | 0 ± 0 | 0 ± 0 | 0 ± 0 | 1.33 ± 0.67 | 0 ± 0 | 0 ± 0 | 0 ± 0 | 0 ± 0 |
| Myriodontium keratinophilum | 0 ± 0 | 0 ± 0 | 0 ± 0 | 0.67 ± 0.67 | 0 ± 0 | 1 ± 0.58 | 1 ± 1 | 0 ± 0 | 0 ± 0 | 0 ± 0 |
| Myrmecridium banksiae | 0 ± 0 | 0 ± 0 | 0 ± 0 | 0 ± 0 | 0 ± 0 | 0 ± 0 | 3.33 ± 3.33 | 0 ± 0 | 0 ± 0 | 0.33 ± 0.33 |
| Myrmecridium phragmitis | 11 ± 6.24 | 0 ± 0 | 0.33 ± 0.33 | 0 ± 0 | 1.67 ± 0.88 | 0.67 ± 0.67 | 6.33 ± 5.36 | 0.33 ± 0.33 | 0 ± 0 | 1.33 ± 1.33 |
| Myrmecridium schulzeri | 5.33 ± 1.45 | 164.33 ± 159.85 | 1.33 ± 0.33 | 11 ± 11 | 0.67 ± 0.67 | 0.67 ± 0.67 | 2.33 ± 0.33 | 8 ± 4.93 | 1.67 ± 1.2 | 1.33 ± 1.33 |
| Myrmecridium sorbicola | 0 ± 0 | 0 ± 0 | 0 ± 0 | 0 ± 0 | 0 ± 0 | 1.67 ± 1.2 | 0 ± 0 | 0 ± 0 | 0 ± 0 | 2.67 ± 1.76 |
| Myrmecridium sp. TMS-2011 | 0.33 ± 0.33 | 2.67 ± 1.76 | 0 ± 0 | 0.33 ± 0.33 | 0 ± 0 | 0 ± 0 | 4.33 ± 3.33 | 1.33 ± 1.33 | 0.33 ± 0.33 | 0 ± 0 |
| Myrmecridium thailandicum | 0 ± 0 | 0 ± 0 | 0 ± 0 | 0 ± 0 | 0 ± 0 | 0.33 ± 0.33 | 0.67 ± 0.67 | 0 ± 0 | 0 ± 0 | 0 ± 0 |
| Myrothecium inundatum | 0 ± 0 | 2 ± 2 | 0 ± 0 | 0 ± 0 | 0 ± 0 | 0 ± 0 | 0 ± 0 | 0 ± 0 | 0 ± 0 | 0 ± 0 |
| Myrothecium sp. | 0.67 ± 0.67 | 25.67 ± 18.26 | 0.67 ± 0.33 | 2 ± 2 | 0.33 ± 0.33 | 5 ± 0.58 | 36 ± 31.53 | 8.33 ± 6.36 | 1.33 ± 0.33 | 1.33 ± 0.88 |
| Myrothecium sp. 08010 | 0 ± 0 | 0 ± 0 | 0 ± 0 | 0 ± 0 | 0 ± 0 | 0.33 ± 0.33 | 0 ± 0 | 6 ± 1.73 | 0.33 ± 0.33 | 0 ± 0 |
| Myrothecium sp. F129 | 0 ± 0 | 0 ± 0 | 3.33 ± 2.4 | 0 ± 0 | 0 ± 0 | 1 ± 1 | 0 ± 0 | 0 ± 0 | 0 ± 0 | 1 ± 1 |
| Myrothecium sp. REF176 | 0 ± 0 | 0 ± 0 | 0 ± 0 | 0 ± 0 | 0 ± 0 | 1 ± 1 | 6.33 ± 5.36 | 0 ± 0 | 0 ± 0 | 0 ± 0 |
| Myxospora crassiseta | 0 ± 0 | 0 ± 0 | 0 ± 0 | 0 ± 0 | 0 ± 0 | 0 ± 0 | 0.33 ± 0.33 | 0 ± 0 | 0 ± 0 | 0.33 ± 0.33 |
| Myxospora sp. 1 LL-2016 | 2 ± 1.15 | 0 ± 0 | 0 ± 0 | 0 ± 0 | 0.67 ± 0.67 | 2 ± 1 | 0 ± 0 | 0 ± 0 | 0 ± 0 | 0 ± 0 |
| Naganishia globosa | 0 ± 0 | 1 ± 1 | 0 ± 0 | 0 ± 0 | 0 ± 0 | 0 ± 0 | 0 ± 0 | 0 ± 0 | 0 ± 0 | 0 ± 0 |
| Nectria balansae | 0 ± 0 | 0.67 ± 0.67 | 0 ± 0 | 4.33 ± 3.84 | 0 ± 0 | 0.33 ± 0.33 | 3 ± 1.15 | 0 ± 0 | 0 ± 0 | 0 ± 0 |
| Nectria cinnabarina | 0 ± 0 | 0 ± 0 | 0.33 ± 0.33 | 0 ± 0 | 0 ± 0 | 0 ± 0 | 0.33 ± 0.33 | 0 ± 0 | 0 ± 0 | 0 ± 0 |
| Nectria diminuta | 0 ± 0 | 0 ± 0 | 0 ± 0 | 0 ± 0 | 0 ± 0 | 0 ± 0 | 0 ± 0 | 1.67 ± 1.67 | 0 ± 0 | 1.67 ± 1.67 |
| Nectria pseudopeziza | 0.33 ± 0.33 | 0 ± 0 | 0.67 ± 0.67 | 0 ± 0 | 5 ± 2.52 | 3 ± 2.52 | 0.33 ± 0.33 | 27.67 ± 21.06 | 2 ± 2 | 0.33 ± 0.33 |
| Nectria pseudotrichia | 0 ± 0 | 0 ± 0 | 0 ± 0 | 0 ± 0 | 0 ± 0 | 0 ± 0 | 1.33 ± 1.33 | 14 ± 7 | 3.67 ± 3.67 | 0 ± 0 |
| Nectria sp. CBS 125498 | 0 ± 0 | 0 ± 0 | 0 ± 0 | 0 ± 0 | 0 ± 0 | 0 ± 0 | 0 ± 0 | 0 ± 0 | 0 ± 0 | 0 ± 0 |
| Nectria sp. ICMP 13358 | 0 ± 0 | 0 ± 0 | 0 ± 0 | 0 ± 0 | 0 ± 0 | 0.33 ± 0.33 | 0 ± 0 | 0 ± 0 | 0 ± 0 | 2 ± 2 |
| Nectria sp. OTU041 AN-2016 | 13.33 ± 13.33 | 2 ± 1 | 81 ± 33.86 | 0.33 ± 0.33 | 2.67 ± 2.67 | 38 ± 18.19 | 8 ± 1.73 | 23 ± 12.1 | 17.67 ± 17.67 | 1.67 ± 0.33 |
| Nectricladiella sp. | 0 ± 0 | 0 ± 0 | 0 ± 0 | 0 ± 0 | 0 ± 0 | 9.67 ± 9.17 | 0 ± 0 | 0 ± 0 | 0 ± 0 | 0 ± 0 |
| Nemania sp. 1-NN-2017 | 0 ± 0 | 0 ± 0 | 0 ± 0 | 0 ± 0 | 0 ± 0 | 0 ± 0 | 0 ± 0 | 0 ± 0 | 0 ± 0 | 1 ± 0.58 |
| Nemania sp. H5-8-NN-2016 | 0 ± 0 | 0 ± 0 | 0 ± 0 | 0 ± 0 | 0 ± 0 | 0.33 ± 0.33 | 0 ± 0 | 0 ± 0 | 0 ± 0 | 0.67 ± 0.67 |
| Neoascochyta sp. | 0 ± 0 | 0 ± 0 | 0 ± 0 | 0 ± 0 | 0 ± 0 | 0 ± 0 | 4.67 ± 4.67 | 0 ± 0 | 0 ± 0 | 1.67 ± 0.33 |
| Neoascochyta sp. OTU023 AN-2016 | 0 ± 0 | 0 ± 0 | 0 ± 0 | 0 ± 0 | 0 ± 0 | 3 ± 1.15 | 1 ± 0.58 | 0 ± 0 | 0 ± 0 | 0 ± 0 |
| Neoascotaiwania terrestris | 0 ± 0 | 0 ± 0 | 0 ± 0 | 0 ± 0 | 0 ± 0 | 0 ± 0 | 0 ± 0 | 0 ± 0 | 0 ± 0 | 0.33 ± 0.33 |
| Neobulgaria sp. | 0 ± 0 | 0 ± 0 | 0 ± 0 | 0 ± 0 | 0 ± 0 | 0 ± 0 | 0 ± 0 | 0 ± 0 | 0 ± 0 | 0 ± 0 |
| Neoceratosperma alsophilae | 0.33 ± 0.33 | 0 ± 0 | 0 ± 0 | 0 ± 0 | 0 ± 0 | 0 ± 0 | 0 ± 0 | 0 ± 0 | 0.33 ± 0.33 | 0 ± 0 |
| Neocucurbitaria rhamnicola | 0 ± 0 | 45.33 ± 22.73 | 0 ± 0 | 1 ± 0.58 | 0 ± 0 | 0 ± 0 | 7.67 ± 3.84 | 0 ± 0 | 0 ± 0 | 0 ± 0 |
| Neodactylaria obpyriformis | 0 ± 0 | 0.33 ± 0.33 | 0.33 ± 0.33 | 5.67 ± 5.67 | 0.33 ± 0.33 | 0 ± 0 | 3.67 ± 3.67 | 0 ± 0 | 0 ± 0 | 0 ± 0 |
| Neodendryphiella mali | 0 ± 0 | 0 ± 0 | 0.67 ± 0.67 | 0 ± 0 | 0 ± 0 | 0 ± 0 | 0 ± 0 | 0 ± 0 | 0 ± 0 | 0 ± 0 |
| Neodevriesia coryneliae | 0 ± 0 | 0 ± 0 | 0 ± 0 | 0 ± 0 | 0 ± 0 | 0 ± 0 | 0 ± 0 | 0 ± 0 | 1 ± 1 | 0 ± 0 |
| Neodevriesia knoxdaviesii | 0 ± 0 | 0 ± 0 | 0.33 ± 0.33 | 0 ± 0 | 0 ± 0 | 0.67 ± 0.67 | 1.33 ± 0.67 | 7.67 ± 4.63 | 4 ± 4 | 0.33 ± 0.33 |
| Neodevriesia lagerstroemiae | 0 ± 0 | 0 ± 0 | 1.33 ± 1.33 | 1 ± 1 | 0 ± 0 | 0.33 ± 0.33 | 0.33 ± 0.33 | 1.33 ± 1.33 | 0.33 ± 0.33 | 0 ± 0 |
| Neofabraea inaequalis | 0 ± 0 | 0 ± 0 | 15 ± 12.12 | 0.33 ± 0.33 | 0 ± 0 | 5.33 ± 4.84 | 0.67 ± 0.67 | 0 ± 0 | 0 ± 0 | 4.67 ± 3.28 |
| Neofabraea malicorticis | 0 ± 0 | 0 ± 0 | 0.67 ± 0.33 | 0 ± 0 | 0 ± 0 | 0.33 ± 0.33 | 0 ± 0 | 0 ± 0 | 0 ± 0 | 0.33 ± 0.33 |
| Neofabraea sp. CKS1078 | 0 ± 0 | 0 ± 0 | 0 ± 0 | 0 ± 0 | 0 ± 0 | 0.67 ± 0.67 | 0 ± 0 | 0 ± 0 | 0 ± 0 | 0 ± 0 |
| Neofusicoccum australe | 4.33 ± 4.33 | 0 ± 0 | 0 ± 0 | 0 ± 0 | 0.33 ± 0.33 | 0 ± 0 | 0 ± 0 | 2.67 ± 2.67 | 1.33 ± 0.67 | 0 ± 0 |
| Neofusicoccum parvum | 0 ± 0 | 0 ± 0 | 0 ± 0 | 0 ± 0 | 0 ± 0 | 0 ± 0 | 0 ± 0 | 1.33 ± 0.88 | 1.33 ± 1.33 | 0.33 ± 0.33 |
| Neohygrocybe nitrata | 0.33 ± 0.33 | 0 ± 0 | 1.67 ± 1.2 | 0 ± 0 | 0 ± 0 | 0 ± 0 | 0 ± 0 | 0 ± 0 | 0 ± 0 | 0 ± 0 |
| Neoidriella desertorum | 0 ± 0 | 1 ± 1 | 0 ± 0 | 0 ± 0 | 0 ± 0 | 0 ± 0 | 0 ± 0 | 0 ± 0 | 0 ± 0 | 0 ± 0 |
| Neomassarina thailandica | 0.67 ± 0.33 | 0 ± 0 | 0 ± 0 | 0.33 ± 0.33 | 0 ± 0 | 0 ± 0 | 1 ± 0.58 | 1.33 ± 0.33 | 0 ± 0 | 0.67 ± 0.67 |
| Neonectria lugdunensis | 0 ± 0 | 10.67 ± 7.69 | 1 ± 1 | 0 ± 0 | 0 ± 0 | 0 ± 0 | 0.67 ± 0.67 | 2 ± 2 | 0.33 ± 0.33 | 0 ± 0 |
| Neonectria major | 0.33 ± 0.33 | 0 ± 0 | 0 ± 0 | 0 ± 0 | 4.33 ± 4.33 | 0 ± 0 | 8 ± 7.51 | 0.33 ± 0.33 | 0 ± 0 | 10.67 ± 8.29 |
| Neonectria obtusispora | 118 ± 31.64 | 1 ± 1 | 0 ± 0 | 0.33 ± 0.33 | 9.67 ± 4.7 | 1 ± 0.58 | 1 ± 0.58 | 0 ± 0 | 0 ± 0 | 1 ± 1 |
| Neonectria sp. 1 RJ2015 | 0 ± 0 | 0 ± 0 | 0 ± 0 | 0.33 ± 0.33 | 0 ± 0 | 6.67 ± 6.67 | 0 ± 0 | 2.67 ± 2.67 | 0 ± 0 | 12.67 ± 10.17 |
| Neonectria_uncultured Neonectria | 0 ± 0 | 1.33 ± 0.88 | 0.67 ± 0.33 | 25 ± 8.62 | 3 ± 1 | 0.33 ± 0.33 | 14 ± 7.81 | 0.67 ± 0.33 | 0 ± 0 | 8.67 ± 5.36 |
| Neopestalotiopsis sp. | 4.33 ± 0.88 | 17.67 ± 10.67 | 71 ± 25.7 | 4.67 ± 1.86 | 2.33 ± 0.88 | 21.67 ± 13.72 | 36.33 ± 21.61 | 222.67 ± 108.11 | 60.67 ± 48.67 | 14 ± 1.73 |
| Neopyrenochaeta inflorescentiae | 2.33 ± 2.33 | 4 ± 3.06 | 0 ± 0 | 0 ± 0 | 8.33 ± 1.76 | 22.67 ± 8.33 | 4.33 ± 1.33 | 64.33 ± 38.2 | 20 ± 20 | 1 ± 0 |
| Neurospora calospora | 0 ± 0 | 0 ± 0 | 0 ± 0 | 0 ± 0 | 0 ± 0 | 0 ± 0 | 0 ± 0 | 0 ± 0 | 0 ± 0 | 0 ± 0 |
| Niesslia exigua | 0 ± 0 | 0 ± 0 | 0 ± 0 | 0 ± 0 | 0 ± 0 | 0 ± 0 | 1 ± 1 | 0 ± 0 | 0 ± 0 | 0 ± 0 |
| Niesslia exilis | 0 ± 0 | 0 ± 0 | 0 ± 0 | 1.33 ± 1.33 | 0 ± 0 | 0 ± 0 | 0.67 ± 0.67 | 0 ± 0 | 0.33 ± 0.33 | 0 ± 0 |
| Niesslia sp. | 0 ± 0 | 0 ± 0 | 0 ± 0 | 0 ± 0 | 0 ± 0 | 0.33 ± 0.33 | 0 ± 0 | 0 ± 0 | 0 ± 0 | 0 ± 0 |
| Niesslia sp. BGE-2018b | 0 ± 0 | 0 ± 0 | 0.33 ± 0.33 | 2.33 ± 2.33 | 0 ± 0 | 0.67 ± 0.67 | 1.67 ± 0.33 | 0 ± 0 | 0 ± 0 | 0 ± 0 |
| Nigrograna mycophila | 0 ± 0 | 0 ± 0 | 3.33 ± 3.33 | 0 ± 0 | 0.33 ± 0.33 | 14.33 ± 5.93 | 0.67 ± 0.67 | 3 ± 3 | 0.33 ± 0.33 | 0 ± 0 |
| Nigrograna obliqua | 0 ± 0 | 0 ± 0 | 0 ± 0 | 0 ± 0 | 0 ± 0 | 0 ± 0 | 1.67 ± 1.67 | 0 ± 0 | 0 ± 0 | 0.67 ± 0.67 |
| Nigrograna sp. JZ-2017a | 0 ± 0 | 0 ± 0 | 0 ± 0 | 0.33 ± 0.33 | 0 ± 0 | 1.33 ± 1.33 | 2.33 ± 2.33 | 0 ± 0 | 0 ± 0 | 0.33 ± 0.33 |
| Nigrograna thymi | 0 ± 0 | 0 ± 0 | 0 ± 0 | 0 ± 0 | 0 ± 0 | 0 ± 0 | 6.67 ± 6.67 | 0 ± 0 | 0.67 ± 0.67 | 0 ± 0 |
| Nigrospora oryzae | 2 ± 0.58 | 172 ± 170 | 19.33 ± 9.94 | 8.33 ± 7.36 | 4.33 ± 1.45 | 9 ± 6.24 | 49.33 ± 33.4 | 27 ± 15.72 | 3 ± 3 | 6.67 ± 1.45 |
| Nigrospora sp. SLM2_10 | 0.67 ± 0.67 | 0 ± 0 | 2 ± 2 | 1 ± 1 | 1 ± 1 | 12.67 ± 6.96 | 11 ± 9.54 | 4 ± 4 | 2.67 ± 0.67 | 2.67 ± 2.67 |
| Nigrospora sp. TA26-9 | 0.33 ± 0.33 | 0.67 ± 0.67 | 5.67 ± 2.19 | 1.33 ± 0.88 | 0 ± 0 | 2 ± 1 | 2.33 ± 0.33 | 6.33 ± 3.53 | 6.33 ± 5.84 | 0 ± 0 |
| Nigrospora sp. TMS-2011 | 1.67 ± 0.88 | 1 ± 0.58 | 1.33 ± 0.33 | 1 ± 1 | 0.33 ± 0.33 | 5.33 ± 4.84 | 8.67 ± 6.77 | 5.33 ± 5.33 | 0 ± 0 | 0 ± 0 |
| Nigrospora sphaerica | 3 ± 1.53 | 1 ± 1 | 25 ± 12.9 | 1.33 ± 0.33 | 3 ± 1.53 | 19.67 ± 9.39 | 9.33 ± 0.88 | 20.33 ± 12.55 | 2.67 ± 2.67 | 2.67 ± 1.45 |
| Ochroconis bacilliformis | 1 ± 1 | 0 ± 0 | 0 ± 0 | 0 ± 0 | 0 ± 0 | 0 ± 0 | 0 ± 0 | 0.67 ± 0.67 | 3.67 ± 3.67 | 0 ± 0 |
| Ochroconis constricta | 0.33 ± 0.33 | 10.33 ± 5.17 | 0 ± 0 | 0.33 ± 0.33 | 0 ± 0 | 0 ± 0 | 2 ± 2 | 0 ± 0 | 0 ± 0 | 0 ± 0 |
| Ochroconis humicola | 0 ± 0 | 1.67 ± 1.2 | 0 ± 0 | 0.67 ± 0.33 | 0 ± 0 | 0 ± 0 | 0.67 ± 0.67 | 11 ± 6.66 | 7.33 ± 3.28 | 1 ± 1 |
| Ochroconis robusta | 0 ± 0 | 0 ± 0 | 0.33 ± 0.33 | 0 ± 0 | 0 ± 0 | 0 ± 0 | 0 ± 0 | 0 ± 0 | 0 ± 0 | 0 ± 0 |
| Oculimacula aestiva | 28.33 ± 20.63 | 26.67 ± 3.18 | 5.67 ± 1.67 | 2.33 ± 0.88 | 7.67 ± 2.6 | 15.33 ± 6.74 | 19 ± 1 | 121 ± 82.24 | 57.67 ± 57.17 | 7 ± 2 |
| Oidiodendron maius | 0 ± 0 | 0.33 ± 0.33 | 2.67 ± 2.67 | 3.33 ± 2.33 | 0 ± 0 | 4.33 ± 2.6 | 2.67 ± 2.67 | 25.33 ± 8.67 | 11.33 ± 6.57 | 13.67 ± 3.28 |
| Oidiodendron periconioides | 0 ± 0 | 0 ± 0 | 0 ± 0 | 0.67 ± 0.67 | 0 ± 0 | 0 ± 0 | 0 ± 0 | 0 ± 0 | 0 ± 0 | 0 ± 0 |
| Oidiodendron sp. 15PA16 | 0 ± 0 | 0 ± 0 | 0 ± 0 | 0 ± 0 | 0 ± 0 | 1.33 ± 0.88 | 1.67 ± 1.67 | 0 ± 0 | 0 ± 0 | 0 ± 0 |
| Oidiodendron sp. L2-1-NN-2016 | 0 ± 0 | 0 ± 0 | 0 ± 0 | 3 ± 2.08 | 0 ± 0 | 0 ± 0 | 2 ± 1.15 | 0 ± 0 | 0 ± 0 | 0 ± 0 |
| Oliveonia pauxilla | 130.33 ± 29.36 | 6 ± 5.51 | 21 ± 17.09 | 0.33 ± 0.33 | 37.67 ± 13.92 | 5.33 ± 4.84 | 6.67 ± 3.18 | 5.33 ± 2.73 | 4.67 ± 2.33 | 2.67 ± 1.76 |
| Olpidium brassicae | 32.33 ± 6.67 | 61.67 ± 17.17 | 3 ± 2.52 | 9 ± 2.08 | 16 ± 4.16 | 238.33 ± 121.71 | 36 ± 14.05 | 0.67 ± 0.67 | 0 ± 0 | 11.67 ± 9.74 |
| Olpidium virulentus | 0 ± 0 | 0 ± 0 | 0 ± 0 | 0 ± 0 | 0 ± 0 | 0 ± 0 | 0 ± 0 | 0 ± 0 | 0 ± 0 | 0 ± 0 |
| Olpidium_uncultured Olpidium | 4.67 ± 3.18 | 14 ± 7 | 0.33 ± 0.33 | 3.33 ± 0.88 | 3.33 ± 0.33 | 17 ± 12.06 | 9 ± 3.21 | 1.33 ± 1.33 | 2.67 ± 1.76 | 2 ± 1.53 |
| Omphalina rustica | 4.33 ± 2.4 | 0 ± 0 | 0 ± 0 | 0 ± 0 | 0 ± 0 | 0 ± 0 | 0.33 ± 0.33 | 37.67 ± 25.39 | 15.33 ± 15.33 | 0 ± 0 |
| Oncopodiella trigonella | 2.67 ± 2.67 | 4.33 ± 1.76 | 0 ± 0 | 0 ± 0 | 0.33 ± 0.33 | 2.67 ± 2.67 | 1 ± 0.58 | 3.33 ± 3.33 | 6 ± 6 | 0.67 ± 0.67 |
| Operculomyces laminatus | 0 ± 0 | 0 ± 0 | 0.67 ± 0.67 | 6.33 ± 6.33 | 0 ± 0 | 0.33 ± 0.33 | 4.33 ± 3.33 | 0.67 ± 0.67 | 0 ± 0 | 0 ± 0 |
| Ophiocordyceps agriotidis | 0 ± 0 | 0 ± 0 | 0 ± 0 | 0 ± 0 | 0 ± 0 | 0 ± 0 | 0 ± 0 | 0.67 ± 0.67 | 0 ± 0 | 0 ± 0 |
| Ophiocordyceps issidarum | 2.67 ± 1.45 | 0.33 ± 0.33 | 0 ± 0 | 0 ± 0 | 0.33 ± 0.33 | 0 ± 0 | 0 ± 0 | 0 ± 0 | 0 ± 0 | 0 ± 0 |
| Ophiocordyceps pruinosa | 3 ± 3 | 0 ± 0 | 0 ± 0 | 0 ± 0 | 1.33 ± 1.33 | 0 ± 0 | 0 ± 0 | 0 ± 0 | 0 ± 0 | 0 ± 0 |
| Ophiocordyceps robertsii | 0 ± 0 | 0 ± 0 | 0 ± 0 | 0.33 ± 0.33 | 0 ± 0 | 2.33 ± 2.33 | 7.67 ± 7.67 | 0 ± 0 | 1 ± 0.58 | 0 ± 0 |
| Ophiocordyceps tettigonia | 0 ± 0 | 0 ± 0 | 1.33 ± 0.88 | 0 ± 0 | 0.33 ± 0.33 | 2.67 ± 2.67 | 0 ± 0 | 7.33 ± 5.46 | 0.33 ± 0.33 | 0 ± 0 |
| Ophiosphaerella agrostidis | 0.33 ± 0.33 | 0 ± 0 | 0 ± 0 | 0 ± 0 | 0 ± 0 | 0 ± 0 | 0 ± 0 | 3 ± 3 | 0 ± 0 | 0 ± 0 |
| Ophiosphaerella herpotricha | 0 ± 0 | 0 ± 0 | 0 ± 0 | 0 ± 0 | 0 ± 0 | 0 ± 0 | 17.33 ± 17.33 | 0.33 ± 0.33 | 0 ± 0 | 0 ± 0 |
| Ophiosphaerella sp. V1M4R71 | 0 ± 0 | 0 ± 0 | 0.33 ± 0.33 | 0.33 ± 0.33 | 0 ± 0 | 0 ± 0 | 0 ± 0 | 0 ± 0 | 0 ± 0 | 0 ± 0 |
| Orbilia cf. querci A731 | 0 ± 0 | 0 ± 0 | 0.33 ± 0.33 | 0 ± 0 | 0 ± 0 | 0.67 ± 0.67 | 0 ± 0 | 0 ± 0 | 0 ± 0 | 0 ± 0 |
| Orbilia dorsalis | 0 ± 0 | 0 ± 0 | 0 ± 0 | 0 ± 0 | 0 ± 0 | 0.67 ± 0.67 | 0 ± 0 | 0 ± 0 | 0 ± 0 | 0 ± 0 |
| Orbilia rubrovacuolata | 0 ± 0 | 0 ± 0 | 0 ± 0 | 0 ± 0 | 0 ± 0 | 0 ± 0 | 0 ± 0 | 0 ± 0 | 0 ± 0 | 1.33 ± 1.33 |
| Orbilia sp. HMAS 139539 | 0 ± 0 | 0 ± 0 | 0 ± 0 | 0 ± 0 | 0 ± 0 | 1.33 ± 1.33 | 0 ± 0 | 0 ± 0 | 0 ± 0 | 0 ± 0 |
| Ovatospora mollicella | 0 ± 0 | 0 ± 0 | 1.33 ± 1.33 | 0 ± 0 | 0 ± 0 | 0 ± 0 | 0 ± 0 | 0 ± 0 | 0 ± 0 | 0 ± 0 |
| Paecilomyces cf. penicillatus 1 RH-2012 | 1 ± 1 | 0 ± 0 | 0 ± 0 | 0 ± 0 | 0 ± 0 | 0 ± 0 | 0 ± 0 | 0 ± 0 | 0 ± 0 | 0 ± 0 |
| Paecilomyces divaricatus | 0 ± 0 | 0 ± 0 | 6 ± 4.04 | 0 ± 0 | 1.67 ± 1.2 | 41.33 ± 15.34 | 2 ± 2 | 0 ± 0 | 0 ± 0 | 0.33 ± 0.33 |
| Paecilomyces nostocoides | 0 ± 0 | 0 ± 0 | 0 ± 0 | 0 ± 0 | 0 ± 0 | 0 ± 0 | 0 ± 0 | 0 ± 0 | 0 ± 0 | 0 ± 0 |
| Paecilomyces sp. GZU-ECBCWS1-5 | 0 ± 0 | 1.33 ± 1.33 | 0.33 ± 0.33 | 5 ± 3.61 | 0 ± 0 | 0 ± 0 | 1.33 ± 1.33 | 2.67 ± 1.76 | 3 ± 2.52 | 0.33 ± 0.33 |
| Paecilomyces sp. KUC5016 | 0 ± 0 | 0 ± 0 | 0.67 ± 0.67 | 0 ± 0 | 0 ± 0 | 0 ± 0 | 0.67 ± 0.67 | 0 ± 0 | 0.33 ± 0.33 | 0 ± 0 |
| Paecilomyces sp. RCEF4111 | 0 ± 0 | 19.67 ± 15.3 | 6.67 ± 4.7 | 9 ± 4.93 | 0 ± 0 | 0 ± 0 | 6.67 ± 5.7 | 3 ± 1.73 | 0.67 ± 0.33 | 1.67 ± 0.33 |
| Paecilomyces sp. SC0924 | 0 ± 0 | 0 ± 0 | 1 ± 0.58 | 0.33 ± 0.33 | 0 ± 0 | 18 ± 10.12 | 1.67 ± 0.88 | 0 ± 0 | 0 ± 0 | 0 ± 0 |
| Papiliotrema aurea | 2 ± 1.53 | 2.67 ± 2.67 | 1 ± 1 | 0 ± 0 | 1.33 ± 0.88 | 1 ± 1 | 1.33 ± 1.33 | 0 ± 0 | 0 ± 0 | 0 ± 0 |
| Papiliotrema flavescens | 36.67 ± 26.74 | 0 ± 0 | 26.67 ± 19.27 | 1.33 ± 1.33 | 30 ± 0.58 | 9 ± 4.36 | 9 ± 0.58 | 137.67 ± 106.39 | 32.67 ± 29.67 | 16.33 ± 5.21 |
| Papiliotrema fuscus | 0 ± 0 | 0.33 ± 0.33 | 0 ± 0 | 2.67 ± 2.19 | 0 ± 0 | 0 ± 0 | 0 ± 0 | 0 ± 0 | 0 ± 0 | 0.67 ± 0.67 |
| Papiliotrema laurentii | 0 ± 0 | 2.33 ± 1.45 | 0 ± 0 | 0 ± 0 | 0 ± 0 | 0 ± 0 | 0 ± 0 | 0 ± 0 | 0 ± 0 | 0 ± 0 |
| Papiliotrema mangalensis | 0 ± 0 | 4.33 ± 2.19 | 0 ± 0 | 0 ± 0 | 0 ± 0 | 0 ± 0 | 0.33 ± 0.33 | 0 ± 0 | 0 ± 0 | 2.33 ± 2.33 |
| Papiliotrema nemorosus | 1 ± 0.58 | 0 ± 0 | 0 ± 0 | 0 ± 0 | 0.67 ± 0.67 | 1.67 ± 1.67 | 0 ± 0 | 2.33 ± 2.33 | 0 ± 0 | 1.67 ± 1.67 |
| Papiliotrema rajasthanensis | 0 ± 0 | 0 ± 0 | 0 ± 0 | 1 ± 0 | 0 ± 0 | 0 ± 0 | 0.33 ± 0.33 | 0 ± 0 | 0 ± 0 | 0 ± 0 |
| Paracamarosporium hawaiiense | 0 ± 0 | 0 ± 0 | 0 ± 0 | 1.33 ± 0.88 | 0 ± 0 | 0.67 ± 0.67 | 0.33 ± 0.33 | 0 ± 0 | 0 ± 0 | 0.33 ± 0.33 |
| Paracladophialophora carceris | 0 ± 0 | 0 ± 0 | 0 ± 0 | 0 ± 0 | 0 ± 0 | 0 ± 0 | 0 ± 0 | 0.67 ± 0.67 | 2.33 ± 1.2 | 0 ± 0 |
| Paracladophialophora sp. | 0 ± 0 | 0 ± 0 | 0 ± 0 | 0 ± 0 | 0 ± 0 | 0 ± 0 | 0 ± 0 | 0 ± 0 | 0.67 ± 0.67 | 0 ± 0 |
| Paraconiothyrium fuckelii | 0 ± 0 | 0 ± 0 | 0.67 ± 0.67 | 0 ± 0 | 0 ± 0 | 2.33 ± 2.33 | 0.33 ± 0.33 | 0 ± 0 | 0 ± 0 | 0 ± 0 |
| Paraconiothyrium sp. | 0 ± 0 | 0 ± 0 | 0.33 ± 0.33 | 0 ± 0 | 0 ± 0 | 1 ± 1 | 1 ± 1 | 0 ± 0 | 0 ± 0 | 0 ± 0 |
| Paraconiothyrium sp. 1 NV-2015 | 0 ± 0 | 0 ± 0 | 2.67 ± 2.67 | 0 ± 0 | 0 ± 0 | 0.33 ± 0.33 | 0.33 ± 0.33 | 0 ± 0 | 0 ± 0 | 0 ± 0 |
| Paraconiothyrium sp. CBS 194.82 | 0 ± 0 | 0 ± 0 | 0 ± 0 | 0 ± 0 | 0 ± 0 | 0 ± 0 | 0 ± 0 | 0 ± 0 | 0.33 ± 0.33 | 0 ± 0 |
| Paracremonium binnewijzendii | 0 ± 0 | 0 ± 0 | 0 ± 0 | 0 ± 0 | 0 ± 0 | 0 ± 0 | 0 ± 0 | 0.67 ± 0.67 | 1.67 ± 1.2 | 0 ± 0 |
| Paracremonium contagium | 0 ± 0 | 0 ± 0 | 0 ± 0 | 0 ± 0 | 0 ± 0 | 2 ± 2 | 0.33 ± 0.33 | 0 ± 0 | 0 ± 0 | 0 ± 0 |
| Paracremonium sp. 1 RJ2014 | 0 ± 0 | 0 ± 0 | 1.33 ± 0.67 | 0.67 ± 0.67 | 5.33 ± 1.45 | 6.33 ± 3.53 | 4.67 ± 2.91 | 0.33 ± 0.33 | 0 ± 0 | 0.67 ± 0.67 |
| Parafabraea eucalypti | 0 ± 0 | 0 ± 0 | 0 ± 0 | 0 ± 0 | 0 ± 0 | 1 ± 1 | 0 ± 0 | 0 ± 0 | 0 ± 0 | 0 ± 0 |
| Paraglomus brasilianum | 0 ± 0 | 0.67 ± 0.67 | 0 ± 0 | 0 ± 0 | 0 ± 0 | 0 ± 0 | 0.33 ± 0.33 | 4.67 ± 3.28 | 2.67 ± 1.76 | 0 ± 0 |
| Paraglomus laccatum | 0 ± 0 | 6 ± 5.03 | 0 ± 0 | 1.67 ± 0.33 | 0 ± 0 | 0 ± 0 | 1.67 ± 0.88 | 0.67 ± 0.67 | 0 ± 0 | 0 ± 0 |
| Paraglomus occultum | 0 ± 0 | 0 ± 0 | 0 ± 0 | 0 ± 0 | 0 ± 0 | 0 ± 0 | 0 ± 0 | 0 ± 0 | 0 ± 0 | 1.67 ± 1.2 |
| Paraglomus sp. 1a14.3 | 0 ± 0 | 21 ± 14.5 | 0 ± 0 | 18 ± 1.73 | 0.33 ± 0.33 | 0.33 ± 0.33 | 5.67 ± 4.7 | 0.33 ± 0.33 | 5 ± 0 | 25.67 ± 8.67 |
| Paramicrosporidium saccamoebae | 1.33 ± 0.88 | 0 ± 0 | 0 ± 0 | 0 ± 0 | 0 ± 0 | 0.33 ± 0.33 | 0 ± 0 | 0 ± 0 | 0 ± 0 | 0 ± 0 |
| Paramicrothyrium chinensis | 0.67 ± 0.67 | 0 ± 0 | 1.67 ± 0.33 | 0 ± 0 | 0.67 ± 0.67 | 2.67 ± 1.33 | 0 ± 0 | 2.67 ± 2.67 | 0 ± 0 | 0 ± 0 |
| Paramyrothecium roridum | 8 ± 6.56 | 12.33 ± 6.69 | 0.33 ± 0.33 | 0.67 ± 0.67 | 27.67 ± 11.33 | 7.33 ± 2.19 | 7 ± 3.06 | 13.67 ± 10.2 | 24 ± 6.51 | 0.67 ± 0.67 |
| Paraphaeosphaeria michotii | 0.67 ± 0.67 | 0 ± 0 | 0 ± 0 | 0 ± 0 | 0.33 ± 0.33 | 1.67 ± 0.88 | 0 ± 0 | 0 ± 0 | 0 ± 0 | 0.33 ± 0.33 |
| Paraphaeosphaeria neglecta | 0 ± 0 | 0 ± 0 | 0 ± 0 | 0 ± 0 | 0.33 ± 0.33 | 0.33 ± 0.33 | 0.67 ± 0.67 | 28.67 ± 14.53 | 20.33 ± 18.85 | 0.33 ± 0.33 |
| Paraphaeosphaeria sporulosa | 0 ± 0 | 0 ± 0 | 0 ± 0 | 0 ± 0 | 0 ± 0 | 0.67 ± 0.67 | 0 ± 0 | 0 ± 0 | 0 ± 0 | 0 ± 0 |
| Paraphaeosphaeria verruculosa | 0 ± 0 | 0 ± 0 | 0 ± 0 | 0 ± 0 | 0 ± 0 | 5.67 ± 2.85 | 1.67 ± 0.88 | 0 ± 0 | 0.33 ± 0.33 | 0 ± 0 |
| Paraphoma chlamydocopiosa | 0 ± 0 | 0 ± 0 | 0 ± 0 | 0 ± 0 | 0 ± 0 | 0 ± 0 | 0.33 ± 0.33 | 0.33 ± 0.33 | 0 ± 0 | 0 ± 0 |
| Paraphoma chrysanthemicola | 1.33 ± 0.88 | 0 ± 0 | 0 ± 0 | 1.33 ± 0.88 | 0.33 ± 0.33 | 0 ± 0 | 1.33 ± 0.88 | 6.33 ± 5.84 | 0 ± 0 | 0.67 ± 0.33 |
| Paraphoma radicina | 0 ± 0 | 1 ± 1 | 0 ± 0 | 0 ± 0 | 0 ± 0 | 0 ± 0 | 0 ± 0 | 0 ± 0 | 0 ± 0 | 0 ± 0 |
| Paraphoma sp. | 6.33 ± 0.33 | 10 ± 6.24 | 0.67 ± 0.67 | 1 ± 1 | 11 ± 5.2 | 8 ± 2.52 | 11.33 ± 4.18 | 12.33 ± 7.22 | 10.33 ± 9.35 | 11 ± 8.54 |
| Paraphysoderma sedebokerense | 0 ± 0 | 0 ± 0 | 105.67 ± 25.77 | 1.33 ± 0.88 | 2.33 ± 1.2 | 33.33 ± 27.95 | 0.33 ± 0.33 | 0.33 ± 0.33 | 0 ± 0 | 2.33 ± 2.33 |
| Parapleurotheciopsis inaequiseptata | 0 ± 0 | 0 ± 0 | 0 ± 0 | 0 ± 0 | 0 ± 0 | 2.33 ± 2.33 | 0 ± 0 | 0 ± 0 | 0 ± 0 | 0 ± 0 |
| Parascedosporium putredinis | 0 ± 0 | 0 ± 0 | 0 ± 0 | 0 ± 0 | 0 ± 0 | 2 ± 2 | 2 ± 2 | 2 ± 2 | 0 ± 0 | 1 ± 0.58 |
| Parasola leiocephala | 7.33 ± 4.06 | 0.33 ± 0.33 | 5 ± 1.15 | 0 ± 0 | 0.67 ± 0.67 | 0 ± 0 | 0 ± 0 | 0 ± 0 | 0 ± 0 | 0 ± 0 |
| Parastagonospora nodorum | 0 ± 0 | 0 ± 0 | 0 ± 0 | 0 ± 0 | 0.33 ± 0.33 | 0 ± 0 | 0 ± 0 | 0 ± 0 | 0 ± 0 | 0 ± 0 |
| Parastagonospora sp. | 0 ± 0 | 0 ± 0 | 0 ± 0 | 0 ± 0 | 0 ± 0 | 0.67 ± 0.67 | 0 ± 0 | 0 ± 0 | 0 ± 0 | 0 ± 0 |
| Parathyridaria ramulicola | 0 ± 0 | 0.33 ± 0.33 | 2 ± 2 | 0 ± 0 | 2 ± 1.53 | 2.67 ± 0.88 | 10 ± 7.64 | 0 ± 0 | 0 ± 0 | 4 ± 3 |
| Parathyridaria rosae | 0 ± 0 | 0 ± 0 | 0.67 ± 0.67 | 0 ± 0 | 0 ± 0 | 0 ± 0 | 1 ± 0.58 | 0 ± 0 | 0 ± 0 | 0 ± 0 |
| Passalora sp. EG-2013 | 0 ± 0 | 0 ± 0 | 0 ± 0 | 0 ± 0 | 0 ± 0 | 0 ± 0 | 0 ± 0 | 0 ± 0 | 0 ± 0 | 0 ± 0 |
| Paurocotylis bynumii | 0 ± 0 | 0 ± 0 | 0.67 ± 0.67 | 0 ± 0 | 0 ± 0 | 0 ± 0 | 0 ± 0 | 0 ± 0 | 0 ± 0 | 0 ± 0 |
| Penicillifer bipapillatus | 0 ± 0 | 0 ± 0 | 7.67 ± 3.93 | 0 ± 0 | 3.67 ± 3.67 | 7.67 ± 3.93 | 0.33 ± 0.33 | 0 ± 0 | 0 ± 0 | 0 ± 0 |
| Penicillifer diparietisporus | 0 ± 0 | 0 ± 0 | 2 ± 1.15 | 0.67 ± 0.67 | 0 ± 0 | 0.67 ± 0.67 | 0.67 ± 0.33 | 0 ± 0 | 0 ± 0 | 0 ± 0 |
| Penicillifer pulcher | 0 ± 0 | 0 ± 0 | 0 ± 0 | 0 ± 0 | 0 ± 0 | 0 ± 0 | 8 ± 8 | 0 ± 0 | 0 ± 0 | 0 ± 0 |
| Penicillifer sp. 1-NN-2017 | 0 ± 0 | 5 ± 5 | 0.33 ± 0.33 | 2 ± 1.53 | 0 ± 0 | 24.67 ± 13.38 | 7.67 ± 1.86 | 0 ± 0 | 0.67 ± 0.67 | 10 ± 4.93 |
| Penicillium adametzioides | 0 ± 0 | 0 ± 0 | 0 ± 0 | 0 ± 0 | 0 ± 0 | 0 ± 0 | 0 ± 0 | 1 ± 1 | 0.67 ± 0.67 | 0 ± 0 |
| Penicillium arenicola | 0 ± 0 | 0 ± 0 | 0 ± 0 | 0 ± 0 | 0 ± 0 | 0 ± 0 | 0 ± 0 | 0 ± 0 | 0 ± 0 | 0.67 ± 0.67 |
| Penicillium aurantiocandidum | 0 ± 0 | 0 ± 0 | 0 ± 0 | 0 ± 0 | 0 ± 0 | 0 ± 0 | 0 ± 0 | 0 ± 0 | 0 ± 0 | 2.67 ± 2.67 |
| Penicillium brasilianum | 0 ± 0 | 17.33 ± 4.37 | 5.67 ± 5.17 | 1.67 ± 0.88 | 0 ± 0 | 0.33 ± 0.33 | 11.33 ± 6.96 | 0 ± 0 | 0 ± 0 | 0.33 ± 0.33 |
| Penicillium brevicompactum | 0.33 ± 0.33 | 0 ± 0 | 2 ± 1.53 | 5.67 ± 2.6 | 0.67 ± 0.33 | 5.33 ± 2.03 | 18.67 ± 5.46 | 33.67 ± 28.24 | 2.67 ± 2.19 | 7 ± 3.79 |
| Penicillium cairnsense | 9.33 ± 7.36 | 0.67 ± 0.67 | 0 ± 0 | 0 ± 0 | 1.33 ± 0.88 | 0 ± 0 | 0.33 ± 0.33 | 0 ± 0 | 0 ± 0 | 0 ± 0 |
| Penicillium citreonigrum | 0 ± 0 | 0.33 ± 0.33 | 0 ± 0 | 0 ± 0 | 0 ± 0 | 0 ± 0 | 0 ± 0 | 0 ± 0 | 0 ± 0 | 0 ± 0 |
| Penicillium citrinum | 0.33 ± 0.33 | 2.67 ± 0.33 | 0.33 ± 0.33 | 0.67 ± 0.33 | 1 ± 1 | 1.67 ± 0.33 | 1 ± 0.58 | 0.33 ± 0.33 | 0.33 ± 0.33 | 1 ± 0.58 |
| Penicillium clavigerum | 0 ± 0 | 16.33 ± 7.62 | 0.33 ± 0.33 | 2.33 ± 1.45 | 0.33 ± 0.33 | 0 ± 0 | 3.33 ± 3.33 | 18 ± 16.52 | 4 ± 4 | 11.33 ± 7.13 |
| Penicillium coprobium | 0 ± 0 | 0 ± 0 | 1.33 ± 0.88 | 0 ± 0 | 0 ± 0 | 0 ± 0 | 3.33 ± 2.85 | 0.33 ± 0.33 | 1 ± 1 | 0.33 ± 0.33 |
| Penicillium copticola | 0.67 ± 0.33 | 0 ± 0 | 0.33 ± 0.33 | 0.33 ± 0.33 | 3 ± 2.08 | 1.67 ± 0.88 | 2.33 ± 0.67 | 3.67 ± 2.73 | 3.33 ± 2.4 | 4.67 ± 3.18 |
| Penicillium daleae | 0 ± 0 | 2 ± 1 | 0 ± 0 | 20.33 ± 13.78 | 0.33 ± 0.33 | 0 ± 0 | 19 ± 11.27 | 0.33 ± 0.33 | 0.33 ± 0.33 | 2.33 ± 1.33 |
| Penicillium digitatum | 56 ± 41 | 0 ± 0 | 0 ± 0 | 0 ± 0 | 4.33 ± 3.84 | 0.67 ± 0.67 | 0.67 ± 0.67 | 0 ± 0 | 0 ± 0 | 0 ± 0 |
| Penicillium dimorphosporum | 0 ± 0 | 0 ± 0 | 0 ± 0 | 0 ± 0 | 0 ± 0 | 0 ± 0 | 0 ± 0 | 0.33 ± 0.33 | 0 ± 0 | 0 ± 0 |
| Penicillium glabrum | 0.67 ± 0.67 | 0.67 ± 0.67 | 7.33 ± 4.1 | 15.33 ± 12.35 | 1.67 ± 1.67 | 8.67 ± 5.93 | 17.33 ± 9.84 | 9 ± 1.15 | 5.33 ± 2.03 | 0.67 ± 0.67 |
| Penicillium herquei | 0 ± 0 | 0 ± 0 | 2 ± 1.15 | 0.33 ± 0.33 | 0.67 ± 0.33 | 1.67 ± 1.2 | 3 ± 2.08 | 75.33 ± 38.1 | 21 ± 21 | 0.67 ± 0.67 |
| Penicillium janczewskii | 0 ± 0 | 0 ± 0 | 0.33 ± 0.33 | 1.33 ± 0.88 | 0 ± 0 | 13 ± 4.16 | 34 ± 24.58 | 6.33 ± 2.33 | 9.67 ± 4.41 | 2.33 ± 0.33 |
| Penicillium laeve | 0 ± 0 | 3 ± 3 | 0 ± 0 | 0.33 ± 0.33 | 0 ± 0 | 0 ± 0 | 1.33 ± 1.33 | 0.33 ± 0.33 | 0 ± 0 | 8.33 ± 3.48 |
| Penicillium lagena | 0 ± 0 | 0 ± 0 | 0 ± 0 | 0 ± 0 | 0 ± 0 | 1 ± 1 | 0 ± 0 | 0 ± 0 | 0 ± 0 | 0 ± 0 |
| Penicillium maclennaniae | 0 ± 0 | 11.67 ± 6.69 | 13.67 ± 5.21 | 1.67 ± 0.88 | 0 ± 0 | 9.33 ± 3.93 | 2.67 ± 1.45 | 0.67 ± 0.33 | 0.67 ± 0.67 | 0.67 ± 0.33 |
| Penicillium megasporum | 0 ± 0 | 0 ± 0 | 0.33 ± 0.33 | 0 ± 0 | 0 ± 0 | 0.67 ± 0.67 | 0 ± 0 | 0 ± 0 | 0 ± 0 | 0 ± 0 |
| Penicillium multicolor | 12 ± 4.73 | 2 ± 1.53 | 51 ± 4.73 | 8.67 ± 3.76 | 35 ± 15.04 | 239 ± 45 | 121.67 ± 43.91 | 1211.33 ± 632.65 | 487.33 ± 479.83 | 85.33 ± 49.82 |
| Penicillium nothofagi | 0 ± 0 | 0 ± 0 | 0 ± 0 | 0 ± 0 | 0 ± 0 | 0.67 ± 0.67 | 0 ± 0 | 0 ± 0 | 0 ± 0 | 0 ± 0 |
| Penicillium oxalicum | 0 ± 0 | 0 ± 0 | 0.67 ± 0.67 | 0 ± 0 | 0 ± 0 | 0 ± 0 | 0 ± 0 | 0.33 ± 0.33 | 0 ± 0 | 1 ± 0.58 |
| Penicillium paxilli | 0 ± 0 | 0 ± 0 | 0.67 ± 0.33 | 7.67 ± 4.06 | 1 ± 0.58 | 0.67 ± 0.33 | 6.33 ± 0.88 | 10.67 ± 8.29 | 1.67 ± 1.2 | 0.33 ± 0.33 |
| Penicillium pusillum | 0 ± 0 | 0 ± 0 | 4 ± 3.06 | 0 ± 0 | 0 ± 0 | 2.33 ± 2.33 | 0.67 ± 0.67 | 0 ± 0 | 0 ± 0 | 0 ± 0 |
| Penicillium raphiae | 0.33 ± 0.33 | 0.33 ± 0.33 | 8.67 ± 2.67 | 1 ± 0.58 | 0 ± 0 | 12 ± 2.08 | 31 ± 25.63 | 43 ± 23.43 | 14.33 ± 14.33 | 9.33 ± 4.26 |
| Penicillium rubidurum | 24.33 ± 11.05 | 0.33 ± 0.33 | 0 ± 0 | 0 ± 0 | 1.67 ± 1.2 | 0 ± 0 | 0 ± 0 | 0 ± 0 | 0 ± 0 | 0 ± 0 |
| Penicillium sacculum | 0.33 ± 0.33 | 341 ± 135.02 | 1 ± 0.58 | 14.67 ± 12.72 | 0.67 ± 0.33 | 2 ± 2 | 70.33 ± 55.11 | 0 ± 0 | 0 ± 0 | 0.67 ± 0.67 |
| Penicillium sanguifluum | 0 ± 0 | 0 ± 0 | 0.33 ± 0.33 | 0 ± 0 | 0.33 ± 0.33 | 16 ± 9.87 | 55.33 ± 52.84 | 3 ± 1.53 | 1 ± 0.58 | 1 ± 0.58 |
| Penicillium scabrosum | 0 ± 0 | 1 ± 0.58 | 8 ± 2.08 | 5.67 ± 4.67 | 1.33 ± 0.88 | 15 ± 3.21 | 56 ± 29.96 | 3 ± 1.53 | 2.33 ± 1.33 | 7 ± 3.51 |
| Penicillium sclerotiorum | 0 ± 0 | 0.33 ± 0.33 | 1.33 ± 0.88 | 0.33 ± 0.33 | 3 ± 1 | 2.33 ± 1.45 | 6 ± 4.04 | 9.67 ± 4.37 | 5.67 ± 5.67 | 14 ± 6.24 |
| Penicillium simplicissimum | 0.33 ± 0.33 | 2 ± 0.58 | 1 ± 0.58 | 3 ± 0.58 | 0.33 ± 0.33 | 8 ± 4.04 | 4.67 ± 3.18 | 18 ± 7.64 | 12 ± 11.5 | 2 ± 0.58 |
| Penicillium sp. | 0.33 ± 0.33 | 12.67 ± 6.69 | 43 ± 8.33 | 4 ± 2.52 | 1.67 ± 0.88 | 26 ± 18.18 | 11 ± 4.93 | 4.67 ± 2.4 | 3.67 ± 1.2 | 3.67 ± 1.33 |
| Penicillium sp. 11MA11 | 0.33 ± 0.33 | 0.33 ± 0.33 | 4.33 ± 2.96 | 4 ± 2.08 | 0.67 ± 0.67 | 4.33 ± 3.38 | 4 ± 2.08 | 8.33 ± 4.06 | 4.67 ± 3.67 | 0.67 ± 0.33 |
| Penicillium sp. 13-M-2 | 4 ± 0.58 | 0 ± 0 | 0 ± 0 | 0.33 ± 0.33 | 15.67 ± 6.44 | 0.67 ± 0.67 | 3.67 ± 2.33 | 0 ± 0 | 0 ± 0 | 0 ± 0 |
| Penicillium sp. 21 BRO-2013 | 0 ± 0 | 0.33 ± 0.33 | 1 ± 0.58 | 4.67 ± 4.18 | 0 ± 0 | 0.67 ± 0.33 | 6.33 ± 1.76 | 2.33 ± 1.2 | 0.67 ± 0.33 | 13 ± 7.23 |
| Penicillium sp. 23-M-5 | 0 ± 0 | 0 ± 0 | 0 ± 0 | 0 ± 0 | 0 ± 0 | 0 ± 0 | 1.67 ± 1.67 | 0 ± 0 | 0 ± 0 | 0 ± 0 |
| Penicillium sp. 4 B134R | 1 ± 1 | 0 ± 0 | 0 ± 0 | 0 ± 0 | 0 ± 0 | 0 ± 0 | 0 ± 0 | 0 ± 0 | 0 ± 0 | 0 ± 0 |
| Penicillium sp. DV-2018m | 0 ± 0 | 5 ± 1.15 | 0.33 ± 0.33 | 0 ± 0 | 0 ± 0 | 0.33 ± 0.33 | 4 ± 1.73 | 0.33 ± 0.33 | 0.33 ± 0.33 | 0.33 ± 0.33 |
| Penicillium sp. F35 | 0.33 ± 0.33 | 0 ± 0 | 1 ± 0.58 | 0 ± 0 | 0.33 ± 0.33 | 0 ± 0 | 0.33 ± 0.33 | 24 ± 23.5 | 0.33 ± 0.33 | 0.33 ± 0.33 |
| Penicillium sp. F40 | 0 ± 0 | 0 ± 0 | 0 ± 0 | 0 ± 0 | 0 ± 0 | 1 ± 1 | 0.33 ± 0.33 | 0 ± 0 | 0 ± 0 | 0 ± 0 |
| Penicillium sp. GHQ-18 | 0 ± 0 | 24 ± 7.57 | 0 ± 0 | 2 ± 0 | 0 ± 0 | 0 ± 0 | 6.67 ± 4.26 | 0.33 ± 0.33 | 0.33 ± 0.33 | 2.67 ± 2.67 |
| Penicillium sp. GZU-BCECYN31-2 | 0 ± 0 | 0 ± 0 | 0 ± 0 | 0 ± 0 | 0 ± 0 | 0.67 ± 0.67 | 0 ± 0 | 0 ± 0 | 0 ± 0 | 0 ± 0 |
| Penicillium sp. H21 | 0 ± 0 | 0 ± 0 | 0 ± 0 | 3.33 ± 2.4 | 0 ± 0 | 2.33 ± 0.88 | 3.33 ± 0.67 | 1.67 ± 0.88 | 0 ± 0 | 0.33 ± 0.33 |
| Penicillium sp. M21 | 0.67 ± 0.33 | 0.33 ± 0.33 | 0 ± 0 | 1 ± 1 | 1.67 ± 1.2 | 2.67 ± 1.45 | 7.33 ± 2.96 | 15.67 ± 5.9 | 7.33 ± 4.91 | 8.33 ± 4.26 |
| Penicillium sp. NWHC 23942-01-02-02 | 0 ± 0 | 0.33 ± 0.33 | 0 ± 0 | 0 ± 0 | 0 ± 0 | 0.33 ± 0.33 | 0 ± 0 | 3.33 ± 2.4 | 1.67 ± 1.67 | 1 ± 0.58 |
| Penicillium sp. NWHC 24266-03-01-01 | 1.67 ± 0.67 | 78.67 ± 42.17 | 0.33 ± 0.33 | 4.33 ± 2.33 | 0.67 ± 0.67 | 0.67 ± 0.67 | 29.67 ± 26.24 | 6.33 ± 2.67 | 5.33 ± 2.96 | 58.33 ± 28.7 |
| Penicillium sp. PSF46 | 0 ± 0 | 0 ± 0 | 0 ± 0 | 0 ± 0 | 0 ± 0 | 0 ± 0 | 0 ± 0 | 0 ± 0 | 0.67 ± 0.67 | 0 ± 0 |
| Penicillium steckii | 0 ± 0 | 0 ± 0 | 0 ± 0 | 0 ± 0 | 0 ± 0 | 0 ± 0 | 0 ± 0 | 0.67 ± 0.67 | 0 ± 0 | 1.33 ± 0.67 |
| Penicillium stoloniferum | 0 ± 0 | 0 ± 0 | 0 ± 0 | 0 ± 0 | 0 ± 0 | 0 ± 0 | 1 ± 1 | 41.33 ± 33.2 | 14 ± 14 | 0 ± 0 |
| Peniophora cf. aurantiaca UC2022951 | 0 ± 0 | 0 ± 0 | 0 ± 0 | 0 ± 0 | 0 ± 0 | 0 ± 0 | 0 ± 0 | 1.33 ± 1.33 | 0 ± 0 | 0 ± 0 |
| Peniophora cinerea | 0 ± 0 | 0 ± 0 | 0 ± 0 | 0.33 ± 0.33 | 0 ± 0 | 0 ± 0 | 0 ± 0 | 0 ± 0 | 0 ± 0 | 1.67 ± 1.67 |
| Peniophora fissilis | 0 ± 0 | 0 ± 0 | 0 ± 0 | 0 ± 0 | 0 ± 0 | 7.33 ± 5.04 | 0.67 ± 0.67 | 0 ± 0 | 0 ± 0 | 0 ± 0 |
| Peniophora sp. NDVN01 | 0 ± 0 | 0 ± 0 | 0.33 ± 0.33 | 0 ± 0 | 0 ± 0 | 0 ± 0 | 0 ± 0 | 0 ± 0 | 0 ± 0 | 2 ± 2 |
| Peniophora sp. TW06-13 | 0 ± 0 | 0 ± 0 | 0 ± 0 | 1.67 ± 1.67 | 0 ± 0 | 0 ± 0 | 0.67 ± 0.67 | 0 ± 0 | 0 ± 0 | 0 ± 0 |
| Peniophora versiformis | 0 ± 0 | 0 ± 0 | 0 ± 0 | 0 ± 0 | 0 ± 0 | 0 ± 0 | 0 ± 0 | 0 ± 0 | 0 ± 0 | 2 ± 2 |
| Perenniporia sp. | 0 ± 0 | 2.67 ± 1.76 | 0 ± 0 | 0.67 ± 0.67 | 0 ± 0 | 0.33 ± 0.33 | 5.33 ± 5.33 | 0.67 ± 0.67 | 0 ± 0 | 34 ± 10.39 |
| Periconia byssoides | 2.33 ± 0.88 | 0.67 ± 0.33 | 11.67 ± 4.84 | 1.67 ± 0.67 | 13.33 ± 12.35 | 9.33 ± 4.84 | 3.67 ± 1.2 | 10 ± 5.51 | 1.33 ± 0.88 | 4 ± 2 |
| Periconia echinochloae | 0.33 ± 0.33 | 0.33 ± 0.33 | 0 ± 0 | 0 ± 0 | 2 ± 1 | 1 ± 0.58 | 0 ± 0 | 5 ± 5 | 2.67 ± 1.76 | 0 ± 0 |
| Periconia elaeidis | 0.67 ± 0.67 | 0 ± 0 | 0 ± 0 | 0 ± 0 | 0.67 ± 0.33 | 0 ± 0 | 1.33 ± 0.88 | 0 ± 0 | 0 ± 0 | 1.33 ± 0.67 |
| Periconia macrospinosa | 2.67 ± 0.88 | 6.67 ± 3.38 | 3 ± 2 | 0 ± 0 | 10 ± 2.31 | 6 ± 2.52 | 2 ± 1.15 | 59.33 ± 28.95 | 30 ± 18.52 | 1.67 ± 1.2 |
| Periconia sp. | 0.67 ± 0.67 | 0 ± 0 | 0 ± 0 | 0 ± 0 | 0 ± 0 | 0 ± 0 | 0.67 ± 0.67 | 5 ± 5 | 0 ± 0 | 0.33 ± 0.33 |
| Periconia sp. CY191 | 0 ± 0 | 1 ± 1 | 0 ± 0 | 0.33 ± 0.33 | 0 ± 0 | 0 ± 0 | 0 ± 0 | 0.67 ± 0.67 | 0 ± 0 | 0 ± 0 |
| Periglandula sp. 1 WB-2014 | 0 ± 0 | 2.33 ± 2.33 | 0 ± 0 | 0.33 ± 0.33 | 0 ± 0 | 0.67 ± 0.67 | 1.67 ± 1.67 | 0.67 ± 0.67 | 0 ± 0 | 0.33 ± 0.33 |
| Periglandula sp. 3 WB-2014 | 0 ± 0 | 0 ± 0 | 0 ± 0 | 0 ± 0 | 0 ± 0 | 0 ± 0 | 0 ± 0 | 1 ± 1 | 0 ± 0 | 0 ± 0 |
| Pervetustus simplex | 0.33 ± 0.33 | 0 ± 0 | 0.67 ± 0.67 | 0 ± 0 | 2 ± 0.58 | 0.67 ± 0.67 | 0.33 ± 0.33 | 22.67 ± 11.57 | 16 ± 16 | 0 ± 0 |
| Pestalotiopsis cocculi | 0 ± 0 | 0 ± 0 | 0.33 ± 0.33 | 0 ± 0 | 0 ± 0 | 0.33 ± 0.33 | 1.67 ± 1.2 | 5 ± 3.21 | 1 ± 0.58 | 0 ± 0 |
| Pestalotiopsis sp. 2 TT-2017 | 1.67 ± 0.88 | 1.33 ± 1.33 | 12.67 ± 0.88 | 3.33 ± 1.86 | 1.33 ± 0.33 | 13 ± 7.02 | 7.33 ± 1.2 | 59 ± 28.88 | 37.33 ± 28.83 | 6.33 ± 1.2 |
| Pestalotiopsis sp. HC02 | 0 ± 0 | 0 ± 0 | 1 ± 0.58 | 0 ± 0 | 0.33 ± 0.33 | 1 ± 1 | 0 ± 0 | 0 ± 0 | 0 ± 0 | 0 ± 0 |
| Pestalotiopsis sp. HKUCC 8323 | 0 ± 0 | 0.67 ± 0.67 | 0 ± 0 | 0 ± 0 | 0 ± 0 | 0 ± 0 | 0 ± 0 | 0 ± 0 | 0 ± 0 | 0 ± 0 |
| Pestalotiopsis sp. SH12KPN | 0 ± 0 | 0.67 ± 0.33 | 0.67 ± 0.67 | 0.33 ± 0.33 | 0 ± 0 | 0 ± 0 | 1 ± 1 | 0 ± 0 | 0 ± 0 | 0 ± 0 |
| Petrakia echinata | 0.33 ± 0.33 | 0 ± 0 | 0 ± 0 | 0.67 ± 0.33 | 0 ± 0 | 0 ± 0 | 1.67 ± 0.88 | 2.33 ± 1.86 | 11 ± 11 | 0.33 ± 0.33 |
| Petriella guttulata | 0 ± 0 | 0 ± 0 | 0 ± 0 | 0 ± 0 | 0 ± 0 | 0 ± 0 | 0.67 ± 0.67 | 1.67 ± 1.67 | 0 ± 0 | 0 ± 0 |
| Pezicula ericae | 0 ± 0 | 0 ± 0 | 7.67 ± 3.71 | 0 ± 0 | 0 ± 0 | 0.67 ± 0.33 | 0 ± 0 | 0 ± 0 | 0 ± 0 | 2.67 ± 2.67 |
| Pezicula sp. 3 ICMP 18931 | 1 ± 0.58 | 0 ± 0 | 0 ± 0 | 0 ± 0 | 0 ± 0 | 0 ± 0 | 1.33 ± 1.33 | 5.67 ± 3.18 | 1.33 ± 1.33 | 0.67 ± 0.33 |
| Peziza sp. | 0 ± 0 | 0 ± 0 | 0 ± 0 | 0 ± 0 | 0 ± 0 | 1 ± 1 | 0 ± 0 | 0 ± 0 | 0 ± 0 | 0 ± 0 |
| Peziza varia | 0 ± 0 | 0 ± 0 | 0.67 ± 0.33 | 0 ± 0 | 0 ± 0 | 0 ± 0 | 0 ± 0 | 0 ± 0 | 0 ± 0 | 0 ± 0 |
| Pezizella discreta | 4 ± 2.52 | 0 ± 0 | 1.67 ± 0.88 | 11 ± 9.5 | 1.33 ± 0.88 | 90.67 ± 45.32 | 14.33 ± 11.92 | 0 ± 0 | 0 ± 0 | 0.33 ± 0.33 |
| Phacidiella eucalypti | 0 ± 0 | 0 ± 0 | 0 ± 0 | 0 ± 0 | 0 ± 0 | 1 ± 1 | 0 ± 0 | 0 ± 0 | 0 ± 0 | 0 ± 0 |
| Phaeoacremonium sp. | 0 ± 0 | 0 ± 0 | 0.67 ± 0.67 | 0 ± 0 | 0.67 ± 0.33 | 0 ± 0 | 0 ± 0 | 0 ± 0 | 0 ± 0 | 0 ± 0 |
| Phaeoacremonium sp. Z9 | 0 ± 0 | 0 ± 0 | 0 ± 0 | 0 ± 0 | 0 ± 0 | 0 ± 0 | 0 ± 0 | 0 ± 0 | 0 ± 0 | 0 ± 0 |
| Phaeococcomyces eucalypti | 1.67 ± 1.67 | 0 ± 0 | 0 ± 0 | 0 ± 0 | 0 ± 0 | 0 ± 0 | 0 ± 0 | 0 ± 0 | 0 ± 0 | 0 ± 0 |
| Phaeoisaria clematidis | 0 ± 0 | 0 ± 0 | 0 ± 0 | 0 ± 0 | 0.33 ± 0.33 | 0 ± 0 | 1.67 ± 1.67 | 0 ± 0 | 0 ± 0 | 1.33 ± 1.33 |
| Phaeomoniella pinifoliorum | 0 ± 0 | 3 ± 2.52 | 0 ± 0 | 0 ± 0 | 0 ± 0 | 1 ± 0.58 | 3.67 ± 3.18 | 3.67 ± 1.86 | 2.67 ± 1.2 | 1.67 ± 0.88 |
| Phaeomoniella sp. 1 ICMP 18935 | 0 ± 0 | 0 ± 0 | 0 ± 0 | 0 ± 0 | 0 ± 0 | 0 ± 0 | 0 ± 0 | 0 ± 0 | 0 ± 0 | 0 ± 0 |
| Phaeomoniella sp. 3 ICMP 18946 | 0 ± 0 | 0 ± 0 | 1.67 ± 0.33 | 0 ± 0 | 0 ± 0 | 32.33 ± 20.73 | 1 ± 1 | 0 ± 0 | 0 ± 0 | 0 ± 0 |
| Phaeomoniella sp. F2-2.2-NN-2016 | 0 ± 0 | 0 ± 0 | 0 ± 0 | 0 ± 0 | 0 ± 0 | 0 ± 0 | 0 ± 0 | 3 ± 2.08 | 0 ± 0 | 0 ± 0 |
| Phaeophyscia pyrrhophora | 0 ± 0 | 0 ± 0 | 0 ± 0 | 0 ± 0 | 0 ± 0 | 0 ± 0 | 0 ± 0 | 0 ± 0 | 0 ± 0 | 2 ± 2 |
| Phaeosphaeria sp. | 0 ± 0 | 15.33 ± 2.67 | 0 ± 0 | 0 ± 0 | 0 ± 0 | 12.33 ± 8.25 | 2 ± 1.15 | 0 ± 0 | 0 ± 0 | 0 ± 0 |
| Phaeosphaeria sp. G330 | 0 ± 0 | 0 ± 0 | 0 ± 0 | 0 ± 0 | 0 ± 0 | 1 ± 1 | 0 ± 0 | 0 ± 0 | 0 ± 0 | 0 ± 0 |
| Phaeosphaeria sp. TMS-2011 | 0 ± 0 | 42 ± 17.1 | 0 ± 0 | 2.33 ± 1.86 | 0 ± 0 | 0.67 ± 0.67 | 3 ± 3 | 9.33 ± 7.88 | 7.67 ± 7.67 | 0 ± 0 |
| Phaeosphaeriopsis sp. | 0 ± 0 | 1.33 ± 1.33 | 0 ± 0 | 0 ± 0 | 0 ± 0 | 0 ± 0 | 0 ± 0 | 0 ± 0 | 0 ± 0 | 0 ± 0 |
| Phaeosphaeriopsis sp. CBP21E | 0 ± 0 | 0 ± 0 | 2.33 ± 2.33 | 0 ± 0 | 0 ± 0 | 0 ± 0 | 0 ± 0 | 0 ± 0 | 0 ± 0 | 0 ± 0 |
| Phaeosphaeriopsis sp. TMS-2011 | 5 ± 2.65 | 40 ± 26.51 | 4.33 ± 1.45 | 2.67 ± 2.67 | 0.67 ± 0.67 | 3 ± 0.58 | 7 ± 3.21 | 20.67 ± 16.29 | 3 ± 1.15 | 14 ± 3.06 |
| Phaeothecoidea melaleuca | 0 ± 0 | 0 ± 0 | 0 ± 0 | 0 ± 0 | 0 ± 0 | 0 ± 0 | 0 ± 0 | 2 ± 2 | 0 ± 0 | 0 ± 0 |
| Phaeotremella eugeniae | 0 ± 0 | 0 ± 0 | 0 ± 0 | 0 ± 0 | 5.67 ± 5.67 | 0.33 ± 0.33 | 0 ± 0 | 0 ± 0 | 0 ± 0 | 0 ± 0 |
| Phaeotremella simplex | 0 ± 0 | 0 ± 0 | 0 ± 0 | 0 ± 0 | 0 ± 0 | 0 ± 0 | 0 ± 0 | 0 ± 0 | 0 ± 0 | 0.67 ± 0.67 |
| Phaeotremella skinneri | 0 ± 0 | 0 ± 0 | 0 ± 0 | 0 ± 0 | 0 ± 0 | 0 ± 0 | 0 ± 0 | 0 ± 0 | 0.67 ± 0.67 | 0 ± 0 |
| Phellinocrescentia guianensis | 0 ± 0 | 0 ± 0 | 0 ± 0 | 0 ± 0 | 0 ± 0 | 0 ± 0 | 0 ± 0 | 0.67 ± 0.67 | 0 ± 0 | 0 ± 0 |
| Phialea strobilina | 0.33 ± 0.33 | 0 ± 0 | 3.67 ± 3.18 | 0.33 ± 0.33 | 0.33 ± 0.33 | 2.67 ± 1.76 | 0.33 ± 0.33 | 0 ± 0 | 0 ± 0 | 1.67 ± 1.67 |
| Phialemoniopsis curvata | 0 ± 0 | 0 ± 0 | 0 ± 0 | 0 ± 0 | 0 ± 0 | 0 ± 0 | 0.67 ± 0.67 | 0 ± 0 | 0 ± 0 | 0 ± 0 |
| Phialemonium dimorphosporum | 0 ± 0 | 0 ± 0 | 0.67 ± 0.67 | 0.67 ± 0.67 | 1 ± 0.58 | 25.67 ± 14.19 | 3.33 ± 2.03 | 0 ± 0 | 0 ± 0 | 0 ± 0 |
| Phialemonium inflatum | 0 ± 0 | 0 ± 0 | 0 ± 0 | 2 ± 2 | 0.33 ± 0.33 | 0 ± 0 | 3 ± 3 | 2.33 ± 2.33 | 0 ± 0 | 0 ± 0 |
| Phialemonium obovatum | 0 ± 0 | 0 ± 0 | 1.67 ± 1.67 | 0 ± 0 | 0 ± 0 | 0.33 ± 0.33 | 0 ± 0 | 0 ± 0 | 0 ± 0 | 0 ± 0 |
| Phialemonium sp. 3 RJ2015 | 0 ± 0 | 0 ± 0 | 2 ± 2 | 0.33 ± 0.33 | 0 ± 0 | 1 ± 0.58 | 0 ± 0 | 0 ± 0 | 0 ± 0 | 0 ± 0 |
| Phialemonium sp. T004-F2-2 | 0 ± 0 | 0.33 ± 0.33 | 0 ± 0 | 0.67 ± 0.67 | 0 ± 0 | 3.33 ± 1.45 | 2 ± 2 | 0.33 ± 0.33 | 1 ± 1 | 3.67 ± 3.18 |
| Phialocephala bamuru | 0 ± 0 | 0 ± 0 | 0 ± 0 | 0 ± 0 | 0 ± 0 | 1 ± 1 | 0 ± 0 | 1 ± 0.58 | 0.33 ± 0.33 | 5.33 ± 4.84 |
| Phialocephala fluminis | 2.67 ± 1.33 | 0 ± 0 | 0 ± 0 | 0 ± 0 | 1.33 ± 0.88 | 0 ± 0 | 0 ± 0 | 0 ± 0 | 0 ± 0 | 0 ± 0 |
| Phialocephala fortinii | 0 ± 0 | 0 ± 0 | 0 ± 0 | 0 ± 0 | 0 ± 0 | 1 ± 0.58 | 1.33 ± 1.33 | 0 ± 0 | 0 ± 0 | 0 ± 0 |
| Phialocephala humicola | 0 ± 0 | 0 ± 0 | 0.67 ± 0.67 | 0 ± 0 | 0 ± 0 | 0 ± 0 | 0 ± 0 | 0.33 ± 0.33 | 0 ± 0 | 7.67 ± 2.96 |
| Phialocephala virens | 0 ± 0 | 0 ± 0 | 2 ± 1.53 | 0 ± 0 | 0 ± 0 | 0 ± 0 | 0 ± 0 | 0 ± 0 | 0 ± 0 | 0 ± 0 |
| Phialomyces macrosporus | 0 ± 0 | 0 ± 0 | 0 ± 0 | 0 ± 0 | 0 ± 0 | 0 ± 0 | 0 ± 0 | 0 ± 0 | 0 ± 0 | 4.67 ± 2.91 |
| Phialophora americana | 0 ± 0 | 0 ± 0 | 0 ± 0 | 0 ± 0 | 0 ± 0 | 0 ± 0 | 0 ± 0 | 0 ± 0 | 0 ± 0 | 1.67 ± 1.67 |
| Phialophora geniculata | 0.33 ± 0.33 | 0 ± 0 | 0 ± 0 | 0 ± 0 | 2 ± 0.58 | 0 ± 0 | 1.33 ± 1.33 | 0 ± 0 | 0 ± 0 | 0 ± 0 |
| Phialophora livistonae | 0 ± 0 | 0 ± 0 | 0 ± 0 | 0 ± 0 | 0 ± 0 | 0 ± 0 | 0 ± 0 | 0 ± 0 | 0 ± 0 | 0 ± 0 |
| Phialophora sp. (in: Eurotiomycetes) | 0.33 ± 0.33 | 0 ± 0 | 10 ± 5.77 | 0.33 ± 0.33 | 0 ± 0 | 2.33 ± 2.33 | 0.33 ± 0.33 | 0 ± 0 | 0 ± 0 | 0.33 ± 0.33 |
| Phialophora sp. 1 RJ2014 | 0 ± 0 | 0 ± 0 | 0 ± 0 | 0.33 ± 0.33 | 0 ± 0 | 0 ± 0 | 0.67 ± 0.33 | 3.33 ± 2.4 | 0 ± 0 | 0 ± 0 |
| Phialophora sp. AX146 | 0 ± 0 | 0 ± 0 | 2 ± 1.15 | 0 ± 0 | 0 ± 0 | 0 ± 0 | 0 ± 0 | 0 ± 0 | 0 ± 0 | 0 ± 0 |
| Phialophora sp. AX163 | 0 ± 0 | 0.67 ± 0.67 | 5.67 ± 2.19 | 41.33 ± 9.74 | 8.33 ± 3.76 | 31 ± 17.01 | 46.67 ± 24.04 | 2 ± 2 | 0 ± 0 | 3.67 ± 3.67 |
| Phialophora sp. CDC-B1214 | 0 ± 0 | 0 ± 0 | 0 ± 0 | 0 ± 0 | 0 ± 0 | 0 ± 0 | 0.67 ± 0.67 | 0 ± 0 | 0 ± 0 | 0 ± 0 |
| Phialophora sp. DF35 | 56 ± 19.09 | 2.67 ± 0.33 | 68.67 ± 4.37 | 16.33 ± 12.35 | 47.33 ± 9.94 | 30.33 ± 20.41 | 83.67 ± 55.5 | 435 ± 215.56 | 118.67 ± 114.17 | 10.33 ± 5.78 |
| Phialophora sp. JBS-2014a | 0 ± 0 | 0 ± 0 | 0 ± 0 | 0 ± 0 | 0 ± 0 | 0 ± 0 | 0 ± 0 | 1 ± 1 | 0 ± 0 | 0 ± 0 |
| Phialophora sp. p3901 | 0 ± 0 | 0 ± 0 | 16.33 ± 1.86 | 0 ± 0 | 0 ± 0 | 3 ± 3 | 0.33 ± 0.33 | 0 ± 0 | 0 ± 0 | 0 ± 0 |
| Phlebia livida | 0 ± 0 | 0 ± 0 | 0.33 ± 0.33 | 0 ± 0 | 0 ± 0 | 0 ± 0 | 0 ± 0 | 0 ± 0 | 0 ± 0 | 0 ± 0 |
| Phlebiella sp. 128 VM-2015 | 0.33 ± 0.33 | 0 ± 0 | 0 ± 0 | 0 ± 0 | 0 ± 0 | 1 ± 1 | 0 ± 0 | 0 ± 0 | 0 ± 0 | 8.67 ± 8.67 |
| Phloeomana speirea | 0 ± 0 | 0.33 ± 0.33 | 0 ± 0 | 0 ± 0 | 0 ± 0 | 3.67 ± 3.67 | 2 ± 2 | 0 ± 0 | 0 ± 0 | 0.33 ± 0.33 |
| Phlyctema vagabunda | 0 ± 0 | 0 ± 0 | 0 ± 0 | 0 ± 0 | 0 ± 0 | 1 ± 1 | 0 ± 0 | 0 ± 0 | 0.33 ± 0.33 | 0 ± 0 |
| Pholiota multicingulata | 0 ± 0 | 0 ± 0 | 0 ± 0 | 0 ± 0 | 0 ± 0 | 0 ± 0 | 0 ± 0 | 0 ± 0 | 3 ± 1.53 | 0 ± 0 |
| Pholiotina sp. | 0 ± 0 | 0 ± 0 | 0 ± 0 | 0 ± 0 | 0 ± 0 | 0 ± 0 | 0.33 ± 0.33 | 0 ± 0 | 0 ± 0 | 0 ± 0 |
| Phoma adonidicola | 1 ± 1 | 0 ± 0 | 3.67 ± 2.33 | 1.33 ± 0.88 | 46 ± 31.88 | 6 ± 3.79 | 5 ± 2.65 | 0.67 ± 0.33 | 0.33 ± 0.33 | 1 ± 1 |
| Phoma herbarum | 0 ± 0 | 0 ± 0 | 0.33 ± 0.33 | 0 ± 0 | 0 ± 0 | 2.33 ± 1.45 | 0 ± 0 | 0 ± 0 | 0 ± 0 | 0 ± 0 |
| Phoma sp. | 13.67 ± 7.06 | 29.33 ± 9.6 | 57.67 ± 21.8 | 4.33 ± 1.33 | 14.33 ± 8.65 | 55.33 ± 12.2 | 18 ± 3.61 | 20.67 ± 12.17 | 36.67 ± 33.17 | 40.33 ± 3.53 |
| Phoma sp. BPL2_2 | 0.67 ± 0.67 | 21.33 ± 10.27 | 22.67 ± 6.77 | 2 ± 0.58 | 1.67 ± 0.33 | 17.33 ± 5.36 | 8.33 ± 5.61 | 27.67 ± 14.19 | 10.33 ± 7.97 | 13.33 ± 11.86 |
| Phoma sp. CY107 | 0 ± 0 | 0 ± 0 | 0.67 ± 0.67 | 0 ± 0 | 0 ± 0 | 0.33 ± 0.33 | 0 ± 0 | 0 ± 0 | 0 ± 0 | 0 ± 0 |
| Phoma sp. G358 | 0.33 ± 0.33 | 0 ± 0 | 0 ± 0 | 0 ± 0 | 1.33 ± 1.33 | 0 ± 0 | 0.33 ± 0.33 | 0 ± 0 | 0 ± 0 | 0.33 ± 0.33 |
| Phoma sp. GF8C4 | 0 ± 0 | 0 ± 0 | 0 ± 0 | 0 ± 0 | 0 ± 0 | 0 ± 0 | 0 ± 0 | 0 ± 0 | 0.33 ± 0.33 | 0 ± 0 |
| Phoma sp. LK-2016 | 0 ± 0 | 0.33 ± 0.33 | 1 ± 1 | 0 ± 0 | 0.33 ± 0.33 | 3.67 ± 3.67 | 1.33 ± 0.67 | 3.33 ± 1.76 | 0 ± 0 | 4 ± 2.65 |
| Phomatospora biseriata | 0.67 ± 0.67 | 0 ± 0 | 13.67 ± 2.33 | 1.33 ± 0.33 | 0.67 ± 0.67 | 14.67 ± 3.33 | 3 ± 2.08 | 3.67 ± 1.76 | 0.33 ± 0.33 | 13 ± 11.53 |
| Phomopsis sp. | 0 ± 0 | 0 ± 0 | 0 ± 0 | 0 ± 0 | 0 ± 0 | 0 ± 0 | 0 ± 0 | 0 ± 0 | 0 ± 0 | 0 ± 0 |
| Phomopsis sp. Sn248 | 0 ± 0 | 0 ± 0 | 0 ± 0 | 0.33 ± 0.33 | 0.67 ± 0.67 | 0 ± 0 | 2 ± 2 | 0 ± 0 | 0 ± 0 | 2 ± 1.15 |
| Phragmocephala atra | 0.67 ± 0.67 | 0 ± 0 | 0 ± 0 | 0 ± 0 | 0.33 ± 0.33 | 0 ± 0 | 0 ± 0 | 0 ± 0 | 1.33 ± 1.33 | 0 ± 0 |
| Phragmocephala garethjonesii | 0 ± 0 | 0.33 ± 0.33 | 8.67 ± 6.33 | 0 ± 0 | 0 ± 0 | 2 ± 0.58 | 2 ± 1.53 | 0 ± 0 | 0 ± 0 | 0 ± 0 |
| Picipes badius | 0 ± 0 | 5.67 ± 4.7 | 0 ± 0 | 0.67 ± 0.33 | 0 ± 0 | 0 ± 0 | 1.33 ± 1.33 | 0 ± 0 | 0 ± 0 | 0 ± 0 |
| Pilidium anglicum | 0 ± 0 | 0 ± 0 | 0 ± 0 | 0 ± 0 | 0 ± 0 | 0 ± 0 | 0 ± 0 | 3.67 ± 2.73 | 4.33 ± 4.33 | 0 ± 0 |
| Pilidium sp. BRO-2013 | 0 ± 0 | 29 ± 3.79 | 2.33 ± 2.33 | 0 ± 0 | 0 ± 0 | 0.67 ± 0.67 | 3.67 ± 2.03 | 0 ± 0 | 2.67 ± 2.67 | 0 ± 0 |
| Piloderma sp. YO4-3OI | 0 ± 0 | 0 ± 0 | 0 ± 0 | 0 ± 0 | 0 ± 0 | 0 ± 0 | 0 ± 0 | 0 ± 0 | 0 ± 0 | 0 ± 0 |
| Piskurozyma silvicola | 0 ± 0 | 0 ± 0 | 2 ± 1.53 | 0 ± 0 | 0 ± 0 | 0 ± 0 | 0 ± 0 | 3.67 ± 1.86 | 8.67 ± 8.67 | 0.67 ± 0.67 |
| Piskurozyma taiwanensis | 0 ± 0 | 0 ± 0 | 0.33 ± 0.33 | 0 ± 0 | 0 ± 0 | 0.33 ± 0.33 | 0 ± 0 | 0 ± 0 | 0 ± 0 | 0 ± 0 |
| Pithomyces chartarum | 289.33 ± 265.36 | 393.33 ± 180.07 | 49 ± 28.05 | 39.67 ± 25 | 53.67 ± 33.79 | 53.33 ± 16.83 | 129.67 ± 82.66 | 19.67 ± 11.72 | 30.33 ± 28.83 | 13 ± 6.81 |
| Placopyrenium bucekii | 0 ± 0 | 5 ± 3.61 | 3.67 ± 3.67 | 110.33 ± 49.33 | 0.33 ± 0.33 | 0 ± 0 | 53 ± 26.51 | 1.33 ± 1.33 | 0.33 ± 0.33 | 0.67 ± 0.67 |
| Plectania melastoma | 0 ± 0 | 0 ± 0 | 0.33 ± 0.33 | 0 ± 0 | 0 ± 0 | 0.33 ± 0.33 | 0 ± 0 | 0 ± 0 | 0 ± 0 | 0 ± 0 |
| Plectosphaerella cucumerina | 23.33 ± 5.49 | 28.67 ± 8.41 | 535 ± 60.06 | 127.67 ± 25.21 | 163.33 ± 44.66 | 1162 ± 239.23 | 305.33 ± 33.01 | 124 ± 63.88 | 62.67 ± 60.68 | 1178.67 ± 1008.39 |
| Plectosphaerella sp. | 0 ± 0 | 0 ± 0 | 0 ± 0 | 0 ± 0 | 0 ± 0 | 0 ± 0 | 0 ± 0 | 0.67 ± 0.33 | 0.33 ± 0.33 | 12.67 ± 8.41 |
| Plectosphaerella sp. M32 | 0 ± 0 | 0.33 ± 0.33 | 0 ± 0 | 0.33 ± 0.33 | 4.33 ± 2.85 | 1.33 ± 1.33 | 0 ± 0 | 0.33 ± 0.33 | 0 ± 0 | 0.33 ± 0.33 |
| Plenodomus collinsoniae | 0 ± 0 | 0 ± 0 | 0 ± 0 | 0.33 ± 0.33 | 0 ± 0 | 0 ± 0 | 0 ± 0 | 0 ± 0 | 0 ± 0 | 0 ± 0 |
| Pleotrichocladium opacum | 0 ± 0 | 2.67 ± 1.2 | 0 ± 0 | 4.67 ± 1.76 | 2 ± 1.15 | 5.67 ± 3.71 | 8.67 ± 4.7 | 11 ± 6.81 | 11.67 ± 1.86 | 57.67 ± 15.71 |
| Pleuroascus nicholsonii | 0.33 ± 0.33 | 9.33 ± 3.28 | 7.33 ± 3.93 | 151 ± 35.59 | 1 ± 0.58 | 26.33 ± 4.81 | 89 ± 56.93 | 0 ± 0 | 0 ± 0 | 3.67 ± 1.86 |
| Pleurotheciella krabiensis | 0 ± 0 | 0 ± 0 | 0.67 ± 0.33 | 0 ± 0 | 0 ± 0 | 1.33 ± 1.33 | 0.67 ± 0.67 | 0 ± 0 | 0.67 ± 0.67 | 0 ± 0 |
| Pluteus albidus | 0 ± 0 | 0 ± 0 | 14 ± 13.5 | 0 ± 0 | 0 ± 0 | 7 ± 7 | 1.67 ± 1.67 | 0 ± 0 | 0.33 ± 0.33 | 0 ± 0 |
| Pluteus chrysaegis | 0 ± 0 | 0 ± 0 | 1 ± 1 | 0 ± 0 | 0 ± 0 | 0 ± 0 | 0 ± 0 | 0 ± 0 | 0 ± 0 | 0 ± 0 |
| Pluteus plautus | 0 ± 0 | 0 ± 0 | 0.67 ± 0.67 | 0 ± 0 | 0 ± 0 | 0 ± 0 | 0 ± 0 | 0 ± 0 | 0 ± 0 | 0 ± 0 |
| Pluteus sp. AJ838 | 0 ± 0 | 0 ± 0 | 0 ± 0 | 0.33 ± 0.33 | 0 ± 0 | 0 ± 0 | 0 ± 0 | 0 ± 0 | 0 ± 0 | 0 ± 0 |
| Pluteus sp. BAB-5245 | 0.33 ± 0.33 | 0 ± 0 | 0 ± 0 | 0 ± 0 | 0.33 ± 0.33 | 0 ± 0 | 0 ± 0 | 0 ± 0 | 0.67 ± 0.33 | 0 ± 0 |
| Pluteus variabilicolor | 0 ± 0 | 0 ± 0 | 0 ± 0 | 0 ± 0 | 0 ± 0 | 0 ± 0 | 0.33 ± 0.33 | 0.33 ± 0.33 | 0 ± 0 | 0 ± 0 |
| Poaceascoma helicoides | 0.33 ± 0.33 | 48.33 ± 16.76 | 0 ± 0 | 2 ± 0.58 | 0 ± 0 | 2 ± 1.53 | 12 ± 7.94 | 90 ± 84.56 | 4 ± 1.73 | 4.33 ± 3.84 |
| Pochonia chlamydosporia | 0 ± 0 | 2.33 ± 1.2 | 35.33 ± 15.34 | 40.33 ± 20.21 | 4.67 ± 0.88 | 12.67 ± 1.86 | 54 ± 14.29 | 16 ± 12.12 | 14 ± 10.02 | 4.33 ± 1.45 |
| Pochonia sp. | 0 ± 0 | 5 ± 3.51 | 16 ± 8.33 | 9.67 ± 5.21 | 0 ± 0 | 7.33 ± 1.33 | 24.67 ± 7.84 | 14 ± 5.86 | 7.33 ± 1.76 | 207.67 ± 74.84 |
| Pochonia sp. NBRC 104298 | 0 ± 0 | 40.33 ± 20.34 | 0 ± 0 | 4.67 ± 2.19 | 3.33 ± 2.03 | 0.33 ± 0.33 | 5.67 ± 3.67 | 0 ± 0 | 1 ± 0.58 | 0.33 ± 0.33 |
| Podospora curvicolla | 0 ± 0 | 0 ± 0 | 0 ± 0 | 0 ± 0 | 0 ± 0 | 0 ± 0 | 0 ± 0 | 0 ± 0 | 0 ± 0 | 0 ± 0 |
| Podospora curvula | 0 ± 0 | 0 ± 0 | 3.67 ± 2.33 | 0 ± 0 | 0 ± 0 | 2.33 ± 0.33 | 0.67 ± 0.67 | 0 ± 0 | 0 ± 0 | 2.67 ± 2.67 |
| Podospora didyma | 10.33 ± 9.84 | 0 ± 0 | 0 ± 0 | 0.33 ± 0.33 | 19 ± 18.5 | 0.33 ± 0.33 | 0.67 ± 0.67 | 0 ± 0 | 0 ± 0 | 0 ± 0 |
| Podospora glutinans | 0 ± 0 | 8.67 ± 4.37 | 6.33 ± 4.91 | 1.33 ± 0.88 | 0 ± 0 | 7 ± 1 | 3.67 ± 2.03 | 8.67 ± 4.06 | 15.33 ± 7.06 | 101 ± 73.51 |
| Podospora intestinacea | 0.33 ± 0.33 | 0.33 ± 0.33 | 0.33 ± 0.33 | 0 ± 0 | 0 ± 0 | 1.33 ± 1.33 | 1.67 ± 1.2 | 5.33 ± 1.2 | 1.67 ± 0.88 | 458.67 ± 310.94 |
| Podospora leporina | 0 ± 0 | 0 ± 0 | 0.67 ± 0.67 | 0 ± 0 | 0 ± 0 | 0 ± 0 | 0 ± 0 | 0 ± 0 | 0 ± 0 | 0 ± 0 |
| Podospora pauciseta | 0 ± 0 | 5.67 ± 5.67 | 0 ± 0 | 0.67 ± 0.67 | 0 ± 0 | 0.33 ± 0.33 | 2.33 ± 2.33 | 0 ± 0 | 0 ± 0 | 0 ± 0 |
| Podospora platensis | 0 ± 0 | 0 ± 0 | 9.33 ± 6.57 | 0 ± 0 | 0 ± 0 | 3.33 ± 3.33 | 0.33 ± 0.33 | 0 ± 0 | 0.33 ± 0.33 | 0 ± 0 |
| Podospora pyriformis | 3.67 ± 0.88 | 0 ± 0 | 0 ± 0 | 0 ± 0 | 0.33 ± 0.33 | 0 ± 0 | 0 ± 0 | 0.33 ± 0.33 | 0 ± 0 | 0 ± 0 |
| Podospora serotina | 0 ± 0 | 0 ± 0 | 0 ± 0 | 0 ± 0 | 0 ± 0 | 0.67 ± 0.67 | 0 ± 0 | 0 ± 0 | 0 ± 0 | 0 ± 0 |
| Podospora sp. | 1.67 ± 0.33 | 7 ± 1.15 | 7647.67 ± 540.44 | 93.33 ± 20.95 | 37.33 ± 8.65 | 2053.67 ± 1447.91 | 267.67 ± 40.34 | 24 ± 4.51 | 32.33 ± 12.67 | 594 ± 21.73 |
| Podospora sp. 1 RJ2014 | 0.67 ± 0.67 | 0.33 ± 0.33 | 74 ± 58.11 | 4.67 ± 3.28 | 8 ± 1.73 | 34.67 ± 31.67 | 25.67 ± 11.57 | 1.67 ± 1.67 | 2 ± 2 | 3.33 ± 3.33 |
| Podospora vesticola | 0.33 ± 0.33 | 0 ± 0 | 0 ± 0 | 0 ± 0 | 0.33 ± 0.33 | 15.67 ± 15.67 | 0.67 ± 0.67 | 0 ± 0 | 0 ± 0 | 4.67 ± 0.88 |
| Polycephalomyces formosus | 0 ± 0 | 0 ± 0 | 0 ± 0 | 0 ± 0 | 3.67 ± 2.03 | 0 ± 0 | 0 ± 0 | 0 ± 0 | 0 ± 0 | 0 ± 0 |
| Polycephalomyces sp. RCEF6001 | 0 ± 0 | 0 ± 0 | 0 ± 0 | 0 ± 0 | 0 ± 0 | 0.67 ± 0.67 | 0 ± 0 | 0 ± 0 | 0 ± 0 | 0.67 ± 0.67 |
| Polyphilus frankenii | 1 ± 0.58 | 0 ± 0 | 9.33 ± 5.84 | 1.67 ± 0.88 | 1 ± 0.58 | 58 ± 13.05 | 7 ± 5.57 | 0 ± 0 | 0 ± 0 | 0.33 ± 0.33 |
| Polyphilus sieberi | 0.33 ± 0.33 | 32.67 ± 20.5 | 73 ± 44.84 | 20 ± 9.61 | 1 ± 0.58 | 19 ± 6.35 | 30.33 ± 10.4 | 2.67 ± 1.45 | 5 ± 0 | 9 ± 6 |
| Polyporus arcularius | 0 ± 0 | 0 ± 0 | 0.67 ± 0.67 | 0 ± 0 | 0 ± 0 | 0 ± 0 | 0 ± 0 | 0 ± 0 | 0 ± 0 | 0 ± 0 |
| Porodiplodia livistonae | 23 ± 5.51 | 0 ± 0 | 20 ± 4.93 | 0.67 ± 0.33 | 7 ± 4.04 | 8.33 ± 4.33 | 34.67 ± 28.67 | 2.33 ± 1.2 | 0.67 ± 0.67 | 3 ± 0.58 |
| Praetumpfia obducens | 0 ± 0 | 0 ± 0 | 0 ± 0 | 0 ± 0 | 0 ± 0 | 0 ± 0 | 1 ± 1 | 0 ± 0 | 0 ± 0 | 0 ± 0 |
| Preussia africana | 0.33 ± 0.33 | 19.67 ± 4.37 | 10 ± 3.51 | 0.33 ± 0.33 | 0 ± 0 | 3.33 ± 2.4 | 2.33 ± 1.86 | 0 ± 0 | 0 ± 0 | 0 ± 0 |
| Preussia fleischhakii | 0 ± 0 | 0 ± 0 | 0 ± 0 | 0 ± 0 | 0.33 ± 0.33 | 0.33 ± 0.33 | 0 ± 0 | 0 ± 0 | 0 ± 0 | 0 ± 0 |
| Preussia funiculata | 0 ± 0 | 0 ± 0 | 1 ± 1 | 0 ± 0 | 1 ± 0.58 | 0.33 ± 0.33 | 0 ± 0 | 0 ± 0 | 0 ± 0 | 0.33 ± 0.33 |
| Preussia sp. (in: Fungi) | 0.33 ± 0.33 | 20.67 ± 11.68 | 12.67 ± 5.21 | 0.33 ± 0.33 | 0 ± 0 | 1.33 ± 1.33 | 1.67 ± 1.67 | 1.33 ± 1.33 | 0 ± 0 | 1 ± 1 |
| Preussia sp. ATT024 | 0 ± 0 | 5.33 ± 2.91 | 5 ± 2.08 | 0.33 ± 0.33 | 0 ± 0 | 1.67 ± 1.67 | 0.33 ± 0.33 | 0.33 ± 0.33 | 0 ± 0 | 0.33 ± 0.33 |
| Preussia sp. CY071 | 0 ± 0 | 1 ± 0.58 | 0 ± 0 | 0 ± 0 | 0 ± 0 | 0 ± 0 | 0.33 ± 0.33 | 0 ± 0 | 0 ± 0 | 0 ± 0 |
| Preussia sp. EAL2.5 | 0 ± 0 | 0 ± 0 | 0 ± 0 | 0 ± 0 | 0 ± 0 | 1 ± 1 | 0 ± 0 | 0 ± 0 | 0.33 ± 0.33 | 0 ± 0 |
| Protocrea farinosa | 0 ± 0 | 0 ± 0 | 1.67 ± 1.67 | 0 ± 0 | 0 ± 0 | 2.33 ± 2.33 | 0 ± 0 | 2.67 ± 1.45 | 0 ± 0 | 0 ± 0 |
| Protoventuria_uncultured Protoventuria | 0 ± 0 | 0.33 ± 0.33 | 50.33 ± 6.57 | 5 ± 3.06 | 0.33 ± 0.33 | 7 ± 4.73 | 4.33 ± 2.33 | 1 ± 1 | 0.33 ± 0.33 | 0 ± 0 |
| Psathyrella candolleana | 0 ± 0 | 0 ± 0 | 0 ± 0 | 0.67 ± 0.67 | 0 ± 0 | 0 ± 0 | 3.67 ± 3.67 | 0.67 ± 0.67 | 2 ± 1.53 | 2.33 ± 1.33 |
| Psathyrella ornatispora | 0 ± 0 | 0 ± 0 | 1.33 ± 1.33 | 0 ± 0 | 0 ± 0 | 0 ± 0 | 0 ± 0 | 0 ± 0 | 0 ± 0 | 0 ± 0 |
| Psathyrella panaeoloides | 0.67 ± 0.67 | 0 ± 0 | 0 ± 0 | 0 ± 0 | 0 ± 0 | 0.33 ± 0.33 | 0 ± 0 | 0 ± 0 | 0 ± 0 | 3 ± 3 |
| Pseudaleuria quinaultiana | 7.33 ± 2.91 | 0 ± 0 | 1.67 ± 1.67 | 0 ± 0 | 2.67 ± 0.33 | 2 ± 2 | 0 ± 0 | 3.33 ± 2.85 | 0 ± 0 | 0 ± 0 |
| Pseudaleuria sp. MF-3 | 0 ± 0 | 0 ± 0 | 0 ± 0 | 0 ± 0 | 1.67 ± 1.67 | 4 ± 3.51 | 0 ± 0 | 0 ± 0 | 0 ± 0 | 3.33 ± 3.33 |
| Pseudeurotium bakeri | 1 ± 0.58 | 34 ± 22.11 | 0.33 ± 0.33 | 0.33 ± 0.33 | 0.33 ± 0.33 | 0.33 ± 0.33 | 4.33 ± 2.19 | 0.33 ± 0.33 | 2.67 ± 2.19 | 1.67 ± 0.33 |
| Pseudeurotium sp. 01NH04 | 0 ± 0 | 0 ± 0 | 0 ± 0 | 0 ± 0 | 0 ± 0 | 0 ± 0 | 0 ± 0 | 0 ± 0 | 0 ± 0 | 0 ± 0 |
| Pseudeurotium sp. 24MN02 | 0 ± 0 | 0 ± 0 | 0 ± 0 | 0 ± 0 | 0 ± 0 | 0 ± 0 | 0 ± 0 | 0 ± 0 | 0 ± 0 | 1.33 ± 1.33 |
| Pseudoanungitea vaccinii | 0 ± 0 | 0 ± 0 | 0 ± 0 | 0 ± 0 | 0 ± 0 | 1.33 ± 0.33 | 0 ± 0 | 0 ± 0 | 2 ± 2 | 1 ± 0.58 |
| Pseudoboubovia benkertii | 0 ± 0 | 0 ± 0 | 0 ± 0 | 0 ± 0 | 0 ± 0 | 0 ± 0 | 0 ± 0 | 27.67 ± 15.88 | 11 ± 11 | 0 ± 0 |
| Pseudocatenomycopsis rothmanniae | 1 ± 1 | 0 ± 0 | 0 ± 0 | 0 ± 0 | 0 ± 0 | 0 ± 0 | 0 ± 0 | 0 ± 0 | 0 ± 0 | 0 ± 0 |
| Pseudocercospora nephrolepidicola | 0 ± 0 | 0 ± 0 | 6 ± 6 | 0 ± 0 | 0 ± 0 | 0 ± 0 | 0.33 ± 0.33 | 0.33 ± 0.33 | 7.33 ± 6.84 | 0 ± 0 |
| Pseudocercospora sp. | 0 ± 0 | 0 ± 0 | 0 ± 0 | 0 ± 0 | 0 ± 0 | 0 ± 0 | 0.33 ± 0.33 | 0 ± 0 | 0 ± 0 | 0 ± 0 |
| Pseudocercospora sp. ZJUM 11 | 0 ± 0 | 0 ± 0 | 0 ± 0 | 0.67 ± 0.67 | 5 ± 2 | 0 ± 0 | 0.33 ± 0.33 | 0.33 ± 0.33 | 0 ± 0 | 1 ± 0.58 |
| Pseudoclathrosphaerina spiralis | 0 ± 0 | 0 ± 0 | 0 ± 0 | 0 ± 0 | 0 ± 0 | 0 ± 0 | 0 ± 0 | 2.33 ± 1.2 | 0 ± 0 | 0 ± 0 |
| Pseudocoleophoma calamagrostidis | 0 ± 0 | 0 ± 0 | 0 ± 0 | 0 ± 0 | 0 ± 0 | 0.67 ± 0.33 | 0.33 ± 0.33 | 0.33 ± 0.33 | 0 ± 0 | 12 ± 8.02 |
| Pseudocoleophoma polygonicola | 0.67 ± 0.67 | 0.33 ± 0.33 | 0.33 ± 0.33 | 0.67 ± 0.67 | 3.33 ± 1.33 | 10 ± 1.53 | 5 ± 2.31 | 17.67 ± 11.05 | 11.33 ± 11.33 | 1.67 ± 1.67 |
| Pseudocosmospora rogersonii | 0 ± 0 | 0 ± 0 | 0 ± 0 | 0 ± 0 | 0 ± 0 | 0.33 ± 0.33 | 2.33 ± 1.86 | 0 ± 0 | 0.33 ± 0.33 | 0 ± 0 |
| Pseudocosmospora sp. G.J.S. 95-143 | 0 ± 0 | 0 ± 0 | 0 ± 0 | 0 ± 0 | 0 ± 0 | 0.67 ± 0.67 | 0 ± 0 | 0 ± 0 | 0 ± 0 | 0 ± 0 |
| Pseudocosmospora vilior | 0 ± 0 | 0 ± 0 | 0.67 ± 0.67 | 0 ± 0 | 0 ± 0 | 8.33 ± 4.18 | 0 ± 0 | 0 ± 0 | 0 ± 0 | 0.67 ± 0.67 |
| Pseudodictyosporium wauense | 0 ± 0 | 0 ± 0 | 0 ± 0 | 0 ± 0 | 0 ± 0 | 0 ± 0 | 0 ± 0 | 1.67 ± 1.67 | 1 ± 1 | 0 ± 0 |
| Pseudogymnoascus sp. | 0 ± 0 | 0 ± 0 | 0 ± 0 | 0.33 ± 0.33 | 0 ± 0 | 0.67 ± 0.67 | 2.33 ± 2.33 | 0 ± 0 | 0.33 ± 0.33 | 0 ± 0 |
| Pseudohyphozyma pustula | 0 ± 0 | 0 ± 0 | 0.67 ± 0.67 | 0 ± 0 | 0 ± 0 | 0 ± 0 | 0 ± 0 | 0 ± 0 | 0 ± 0 | 0 ± 0 |
| Pseudomeria mucosa | 0 ± 0 | 0 ± 0 | 0 ± 0 | 0 ± 0 | 0 ± 0 | 0 ± 0 | 0 ± 0 | 0 ± 0 | 0 ± 0 | 0 ± 0 |
| Pseudoophiobolus rosae | 0 ± 0 | 0.67 ± 0.67 | 0 ± 0 | 0 ± 0 | 0 ± 0 | 0 ± 0 | 0.33 ± 0.33 | 0 ± 0 | 0 ± 0 | 0 ± 0 |
| Pseudopestalotiopsis theae | 0.33 ± 0.33 | 0 ± 0 | 190.33 ± 32.43 | 1.33 ± 0.88 | 0.67 ± 0.33 | 35 ± 27.84 | 2.33 ± 1.2 | 0.67 ± 0.67 | 0 ± 0 | 0 ± 0 |
| Pseudopithomyces maydicus | 0.33 ± 0.33 | 9 ± 4.93 | 1.67 ± 1.67 | 0.33 ± 0.33 | 0 ± 0 | 0.33 ± 0.33 | 2.33 ± 2.33 | 0 ± 0 | 0 ± 0 | 0 ± 0 |
| Pseudopyricularia bothriochloae | 0 ± 0 | 0 ± 0 | 0 ± 0 | 0 ± 0 | 0 ± 0 | 0 ± 0 | 0 ± 0 | 0 ± 0 | 0 ± 0 | 0.33 ± 0.33 |
| Pseudorobillarda phragmitis | 2 ± 1.53 | 0 ± 0 | 0 ± 0 | 0 ± 0 | 0 ± 0 | 0 ± 0 | 0 ± 0 | 0 ± 0 | 0 ± 0 | 0 ± 0 |
| Pseudosigmoidea ibarakiensis | 0 ± 0 | 0 ± 0 | 0 ± 0 | 1 ± 1 | 0 ± 0 | 0 ± 0 | 2.33 ± 0.88 | 0 ± 0 | 0 ± 0 | 4.33 ± 2.96 |
| Pseudospiropes lotorum | 0 ± 0 | 0 ± 0 | 1.67 ± 1.67 | 0 ± 0 | 0 ± 0 | 1.33 ± 1.33 | 0 ± 0 | 7.33 ± 7.33 | 0 ± 0 | 0 ± 0 |
| Pseudoteratosphaeria perpendicularis | 0 ± 0 | 0 ± 0 | 0 ± 0 | 0 ± 0 | 0 ± 0 | 0 ± 0 | 0 ± 0 | 1 ± 1 | 4.67 ± 4.18 | 3 ± 3 |
| Pseudozyma sp. JCC207 | 0 ± 0 | 0 ± 0 | 1 ± 1 | 0 ± 0 | 0.33 ± 0.33 | 0.33 ± 0.33 | 0 ± 0 | 0 ± 0 | 0 ± 0 | 0 ± 0 |
| Psilocybe inquilina | 3.33 ± 2.85 | 0 ± 0 | 0.67 ± 0.67 | 0 ± 0 | 0.67 ± 0.67 | 0 ± 0 | 0 ± 0 | 0.33 ± 0.33 | 0 ± 0 | 3 ± 3 |
| Psoroglaena sp. BGK-2011 | 0 ± 0 | 0 ± 0 | 0 ± 0 | 3 ± 3 | 0.67 ± 0.33 | 0.67 ± 0.67 | 2 ± 1.53 | 1.33 ± 0.88 | 6.33 ± 2.03 | 0 ± 0 |
| Pterula gracilis | 0 ± 0 | 0 ± 0 | 0 ± 0 | 0 ± 0 | 0 ± 0 | 0 ± 0 | 1 ± 1 | 0 ± 0 | 0 ± 0 | 0 ± 0 |
| Pulvinula constellatio | 15 ± 5.77 | 0 ± 0 | 3.67 ± 3.67 | 1 ± 0.58 | 1.67 ± 0.67 | 0 ± 0 | 5 ± 4.04 | 449 ± 436.01 | 551.67 ± 320.57 | 0.67 ± 0.33 |
| Pulvinula niveoalba | 0 ± 0 | 0 ± 0 | 0 ± 0 | 0 ± 0 | 0.33 ± 0.33 | 0.67 ± 0.67 | 1.67 ± 1.67 | 8 ± 4.16 | 0.33 ± 0.33 | 0.67 ± 0.33 |
| Purpureocillium lavendulum | 1.33 ± 0.67 | 11.67 ± 7.31 | 6.67 ± 3.18 | 1.33 ± 1.33 | 0.33 ± 0.33 | 13.33 ± 2.33 | 31 ± 21.03 | 153.33 ± 93.53 | 52 ± 44.52 | 18.67 ± 10.11 |
| Purpureocillium lilacinum | 19.33 ± 3.18 | 154.67 ± 45.17 | 107.67 ± 31.76 | 65.33 ± 30.6 | 68 ± 23.07 | 171 ± 52.54 | 270 ± 88.69 | 1116.33 ± 845.46 | 685.33 ± 611.44 | 193.33 ± 57.68 |
| Pycnidiophora sp. caf8 | 0 ± 0 | 8 ± 3.61 | 0 ± 0 | 0.33 ± 0.33 | 0 ± 0 | 0 ± 0 | 1.33 ± 1.33 | 0 ± 0 | 0 ± 0 | 0 ± 0 |
| Pyrenochaeta nobilis | 0 ± 0 | 0 ± 0 | 0 ± 0 | 0 ± 0 | 0 ± 0 | 0 ± 0 | 42.67 ± 42.67 | 0.67 ± 0.33 | 0.33 ± 0.33 | 45.67 ± 14.4 |
| Pyrenochaeta sp. | 1.67 ± 0.33 | 0.33 ± 0.33 | 5.33 ± 2.19 | 5.67 ± 4.18 | 6.33 ± 0.88 | 23.67 ± 12.57 | 6.67 ± 3.67 | 1.33 ± 0.88 | 6 ± 4 | 23.67 ± 12.25 |
| Pyrenochaeta sp. 14009 | 0 ± 0 | 0 ± 0 | 0.67 ± 0.67 | 0 ± 0 | 0 ± 0 | 0 ± 0 | 0 ± 0 | 0 ± 0 | 0 ± 0 | 0 ± 0 |
| Pyrenochaeta sp. GCG2(1) | 0 ± 0 | 0 ± 0 | 5.33 ± 3.53 | 0.33 ± 0.33 | 0 ± 0 | 3.33 ± 3.33 | 0 ± 0 | 0 ± 0 | 1.67 ± 1.67 | 0.67 ± 0.67 |
| Pyrenochaeta sp. ZLY-2010b | 0 ± 0 | 0 ± 0 | 1 ± 1 | 0 ± 0 | 0 ± 0 | 0 ± 0 | 0 ± 0 | 0 ± 0 | 0 ± 0 | 0 ± 0 |
| Pyrenochaeta sp. shylm24 | 8 ± 5.03 | 7.67 ± 3.48 | 20 ± 4.04 | 18.33 ± 13.98 | 46 ± 33.41 | 29.67 ± 4.48 | 10.33 ± 7.54 | 6.33 ± 6.33 | 8.33 ± 4.41 | 3 ± 1.53 |
| Pyrenochaetopsis leptospora | 75.67 ± 38.27 | 41.33 ± 15.81 | 14.67 ± 10.09 | 22.33 ± 3.71 | 131 ± 93.18 | 50.67 ± 5.81 | 61.67 ± 9.87 | 49 ± 16.5 | 16.67 ± 5.55 | 25.33 ± 5.04 |
| Pyrenochaetopsis sp. | 2 ± 1.53 | 204.33 ± 91.81 | 1.33 ± 0.67 | 11.33 ± 8.88 | 15.33 ± 14.34 | 37.67 ± 15.98 | 244 ± 193.45 | 79 ± 45.65 | 58.33 ± 56.83 | 6 ± 3.21 |
| Pyrenochaetopsis sp. PG293 | 5.33 ± 2.19 | 2.33 ± 2.33 | 0.67 ± 0.67 | 1 ± 1 | 2.33 ± 1.33 | 7.33 ± 5.9 | 6.67 ± 3.76 | 1.33 ± 1.33 | 0 ± 0 | 1.67 ± 1.67 |
| Pyrenophora lolii | 0 ± 0 | 0 ± 0 | 12.33 ± 12.33 | 0.33 ± 0.33 | 0 ± 0 | 6.67 ± 6.67 | 0 ± 0 | 0.33 ± 0.33 | 0 ± 0 | 0 ± 0 |
| Pyricularia parasitica | 0 ± 0 | 0 ± 0 | 0 ± 0 | 0 ± 0 | 0 ± 0 | 0 ± 0 | 0 ± 0 | 2.67 ± 2.67 | 0 ± 0 | 0 ± 0 |
| Pyrigemmula aurantiaca | 0.33 ± 0.33 | 8 ± 2.52 | 1.33 ± 1.33 | 1.33 ± 0.33 | 0.67 ± 0.67 | 3.33 ± 3.33 | 2.67 ± 2.19 | 0 ± 0 | 0 ± 0 | 0 ± 0 |
| Pyxidiophora arvernensis | 0 ± 0 | 0 ± 0 | 0.67 ± 0.67 | 0 ± 0 | 0 ± 0 | 31 ± 15.52 | 0 ± 0 | 1.33 ± 1.33 | 1.33 ± 1.33 | 1 ± 1 |
| Pyxidiophora microspora | 1.33 ± 1.33 | 0 ± 0 | 1.67 ± 1.2 | 0.67 ± 0.67 | 0.67 ± 0.67 | 10 ± 9.02 | 0.33 ± 0.33 | 0 ± 0 | 0 ± 0 | 0.67 ± 0.67 |
| Quadricrura meridionalis | 0 ± 0 | 0 ± 0 | 0 ± 0 | 0 ± 0 | 0 ± 0 | 0 ± 0 | 0 ± 0 | 3.67 ± 3.67 | 0.33 ± 0.33 | 0 ± 0 |
| Quadricrura septentrionalis | 0 ± 0 | 0 ± 0 | 0 ± 0 | 0 ± 0 | 1.67 ± 0.88 | 0 ± 0 | 0.33 ± 0.33 | 0 ± 0 | 0 ± 0 | 0 ± 0 |
| Rachicladosporium americanum | 0 ± 0 | 0 ± 0 | 0 ± 0 | 0 ± 0 | 0 ± 0 | 0 ± 0 | 0 ± 0 | 0 ± 0 | 0 ± 0 | 4.33 ± 3.84 |
| Rachicladosporium antarcticum | 0.33 ± 0.33 | 0 ± 0 | 0 ± 0 | 0 ± 0 | 0 ± 0 | 0.33 ± 0.33 | 0 ± 0 | 0 ± 0 | 0 ± 0 | 0.67 ± 0.67 |
| Rachicladosporium cboliae | 0 ± 0 | 0 ± 0 | 0 ± 0 | 0 ± 0 | 0 ± 0 | 0 ± 0 | 0 ± 0 | 1.33 ± 0.67 | 1.33 ± 1.33 | 1.33 ± 1.33 |
| Racocetra coralloidea | 24.33 ± 7.45 | 4.33 ± 1.86 | 5.67 ± 5.17 | 21.67 ± 18.17 | 44.33 ± 8.17 | 14 ± 10.15 | 10 ± 3.51 | 54.33 ± 31.62 | 22.33 ± 19.84 | 18 ± 6.51 |
| Radulomyces confluens | 0 ± 0 | 0 ± 0 | 0 ± 0 | 0 ± 0 | 0 ± 0 | 0 ± 0 | 0 ± 0 | 1 ± 1 | 0 ± 0 | 0.33 ± 0.33 |
| Ragnhildiana diffusa | 0 ± 0 | 0 ± 0 | 0 ± 0 | 0 ± 0 | 0 ± 0 | 0 ± 0 | 1 ± 1 | 115.67 ± 91.81 | 34.33 ± 33.83 | 2 ± 1.53 |
| Ramariopsis corniculata | 0 ± 0 | 0 ± 0 | 0 ± 0 | 0 ± 0 | 0 ± 0 | 0.67 ± 0.67 | 0 ± 0 | 0 ± 0 | 0 ± 0 | 0 ± 0 |
| Ramariopsis crocea | 0 ± 0 | 0 ± 0 | 36 ± 10.44 | 0.67 ± 0.67 | 0.33 ± 0.33 | 11.67 ± 8.84 | 0 ± 0 | 0 ± 0 | 0 ± 0 | 0.67 ± 0.67 |
| Ramariopsis flavescens | 0 ± 0 | 0 ± 0 | 2 ± 1 | 0 ± 0 | 0 ± 0 | 0.33 ± 0.33 | 0 ± 0 | 0 ± 0 | 0 ± 0 | 3.33 ± 3.33 |
| Ramariopsis helvola | 0 ± 0 | 0 ± 0 | 0 ± 0 | 0 ± 0 | 0 ± 0 | 0 ± 0 | 0 ± 0 | 0 ± 0 | 0 ± 0 | 2.33 ± 2.33 |
| Ramariopsis laeticolor | 0.67 ± 0.67 | 1.67 ± 1.67 | 0 ± 0 | 44.67 ± 22.93 | 0 ± 0 | 0 ± 0 | 21.33 ± 6.33 | 0 ± 0 | 0.67 ± 0.67 | 0 ± 0 |
| Ramariopsis pulchella | 16 ± 8.96 | 0.33 ± 0.33 | 0 ± 0 | 0 ± 0 | 0 ± 0 | 0 ± 0 | 0 ± 0 | 0 ± 0 | 0 ± 0 | 0 ± 0 |
| Ramariopsis sp. | 0 ± 0 | 0 ± 0 | 4.67 ± 2.33 | 0.67 ± 0.67 | 0.33 ± 0.33 | 1.33 ± 0.67 | 3 ± 2 | 0 ± 0 | 0 ± 0 | 0 ± 0 |
| Ramgea ozimecii | 2.67 ± 1.76 | 1 ± 0.58 | 1115.67 ± 569.77 | 9.33 ± 3.18 | 7.67 ± 2.03 | 138.33 ± 105.29 | 40 ± 23.09 | 0.33 ± 0.33 | 1 ± 1 | 1.67 ± 1.67 |
| Ramicandelaber taiwanensis | 0 ± 0 | 0 ± 0 | 0 ± 0 | 0 ± 0 | 0 ± 0 | 0 ± 0 | 0 ± 0 | 0 ± 0 | 3.67 ± 3.67 | 0 ± 0 |
| Ramichloridium apiculatum | 0 ± 0 | 0 ± 0 | 0 ± 0 | 0 ± 0 | 0 ± 0 | 0 ± 0 | 0 ± 0 | 2.67 ± 1.33 | 1.33 ± 1.33 | 0 ± 0 |
| Ramichloridium brasilianum | 0.67 ± 0.67 | 0 ± 0 | 0 ± 0 | 0.67 ± 0.67 | 0 ± 0 | 0 ± 0 | 1 ± 1 | 2 ± 1.53 | 2.67 ± 1.76 | 0 ± 0 |
| Ramophialophora humicola | 67.33 ± 15.3 | 0.67 ± 0.67 | 13.33 ± 3.48 | 1.33 ± 1.33 | 57.33 ± 14.17 | 9.67 ± 8.69 | 6.33 ± 5.36 | 0 ± 0 | 0 ± 0 | 0.67 ± 0.67 |
| Ramophialophora petraea | 0.33 ± 0.33 | 0 ± 0 | 0 ± 0 | 0 ± 0 | 5 ± 1.73 | 1.33 ± 1.33 | 0 ± 0 | 0 ± 0 | 0 ± 0 | 0 ± 0 |
| Ramulariopsis gossypii | 0 ± 0 | 0.67 ± 0.33 | 41.67 ± 17.7 | 1 ± 1 | 0 ± 0 | 5 ± 3.61 | 0 ± 0 | 2.33 ± 2.33 | 0 ± 0 | 0.67 ± 0.67 |
| Rasamsonia emersonii | 0 ± 0 | 0 ± 0 | 0 ± 0 | 0 ± 0 | 0 ± 0 | 0 ± 0 | 0 ± 0 | 0 ± 0 | 0.33 ± 0.33 | 0 ± 0 |
| Remersonia sp. | 0 ± 0 | 0 ± 0 | 0 ± 0 | 0 ± 0 | 0 ± 0 | 0 ± 0 | 0 ± 0 | 7.67 ± 5.78 | 0 ± 0 | 0 ± 0 |
| Repetobasidium glaucocanum | 0 ± 0 | 0 ± 0 | 0 ± 0 | 0 ± 0 | 0 ± 0 | 0 ± 0 | 0.33 ± 0.33 | 7.67 ± 4.06 | 0.33 ± 0.33 | 0 ± 0 |
| Rhexodenticula acaciae | 0 ± 0 | 0.67 ± 0.33 | 0 ± 0 | 0 ± 0 | 0.33 ± 0.33 | 0 ± 0 | 0 ± 0 | 0.33 ± 0.33 | 0 ± 0 | 0 ± 0 |
| Rhinocladiella pyriformis | 0 ± 0 | 0 ± 0 | 0 ± 0 | 0 ± 0 | 0 ± 0 | 0 ± 0 | 0 ± 0 | 0 ± 0 | 0 ± 0 | 1 ± 0.58 |
| Rhinocladiella sp. | 0 ± 0 | 0 ± 0 | 2 ± 2 | 0 ± 0 | 0 ± 0 | 0 ± 0 | 0 ± 0 | 0 ± 0 | 0 ± 0 | 0 ± 0 |
| Rhinocladiella sp. 01001b | 0 ± 0 | 4 ± 4 | 4.67 ± 4.18 | 0.67 ± 0.33 | 0.33 ± 0.33 | 16.67 ± 8.11 | 25 ± 23.01 | 1 ± 0.58 | 0 ± 0 | 0 ± 0 |
| Rhinocladiella sp. EXP0525F | 0.33 ± 0.33 | 0 ± 0 | 0 ± 0 | 0 ± 0 | 0.33 ± 0.33 | 0 ± 0 | 0 ± 0 | 0 ± 0 | 1.67 ± 0.88 | 0 ± 0 |
| Rhinocladiella sp. TS-2016 | 0 ± 0 | 0 ± 0 | 1.33 ± 0.67 | 0 ± 0 | 0 ± 0 | 1.33 ± 1.33 | 0 ± 0 | 0 ± 0 | 0 ± 0 | 0 ± 0 |
| Rhinocladiella sp. YH-2009a | 0 ± 0 | 0 ± 0 | 0 ± 0 | 0 ± 0 | 0 ± 0 | 0.33 ± 0.33 | 2 ± 2 | 0 ± 0 | 0 ± 0 | 0 ± 0 |
| Rhizocarpon disporum | 0 ± 0 | 0.33 ± 0.33 | 0 ± 0 | 0 ± 0 | 0 ± 0 | 0 ± 0 | 0 ± 0 | 0 ± 0 | 0 ± 0 | 0 ± 0 |
| Rhizoctonia globularis | 0 ± 0 | 0 ± 0 | 112.33 ± 111.33 | 0 ± 0 | 2.33 ± 1.86 | 3.33 ± 2.4 | 5.67 ± 2.96 | 2 ± 2 | 1.33 ± 1.33 | 0.33 ± 0.33 |
| Rhizoctonia rubi | 0 ± 0 | 0 ± 0 | 0 ± 0 | 0 ± 0 | 1.33 ± 1.33 | 0 ± 0 | 0 ± 0 | 0 ± 0 | 0 ± 0 | 0 ± 0 |
| Rhizoctonia solani | 0 ± 0 | 0 ± 0 | 1 ± 1 | 0 ± 0 | 0 ± 0 | 0 ± 0 | 0 ± 0 | 0 ± 0 | 0 ± 0 | 0 ± 0 |
| Rhizoctonia sp. | 13 ± 7 | 0 ± 0 | 0.33 ± 0.33 | 0 ± 0 | 1 ± 1 | 0 ± 0 | 0 ± 0 | 0 ± 0 | 0 ± 0 | 1.33 ± 1.33 |
| Rhizoctonia sp. ATT213 | 0.33 ± 0.33 | 0 ± 0 | 3.67 ± 1.45 | 0.33 ± 0.33 | 0 ± 0 | 0.67 ± 0.67 | 0.67 ± 0.67 | 0 ± 0 | 0 ± 0 | 0 ± 0 |
| Rhizoctonia sp. C-610 | 0 ± 0 | 0 ± 0 | 0 ± 0 | 0 ± 0 | 0 ± 0 | 0 ± 0 | 0.67 ± 0.67 | 0 ± 0 | 0 ± 0 | 0.33 ± 0.33 |
| Rhizophagus clarus | 0 ± 0 | 0 ± 0 | 0 ± 0 | 1.67 ± 1.2 | 0 ± 0 | 0 ± 0 | 0 ± 0 | 0 ± 0 | 0 ± 0 | 0 ± 0 |
| Rhizophagus intraradices | 11 ± 6.11 | 39 ± 10.41 | 0 ± 0 | 0.33 ± 0.33 | 2 ± 1.15 | 0 ± 0 | 3 ± 2.08 | 2 ± 1.15 | 2.67 ± 0.67 | 0.33 ± 0.33 |
| Rhizophagus irregularis | 3.67 ± 2.03 | 11 ± 4.51 | 0 ± 0 | 1.67 ± 0.67 | 1 ± 0.58 | 0 ± 0 | 1.67 ± 1.67 | 0.33 ± 0.33 | 1 ± 0.58 | 0 ± 0 |
| Rhizophagus sp. (in: Fungi) | 2.67 ± 0.33 | 0 ± 0 | 0 ± 0 | 0 ± 0 | 1 ± 0.58 | 0 ± 0 | 0 ± 0 | 0 ± 0 | 0 ± 0 | 0 ± 0 |
| Rhizophagus sp. 1 SL-2017 | 0 ± 0 | 0 ± 0 | 0 ± 0 | 0 ± 0 | 0 ± 0 | 0 ± 0 | 0 ± 0 | 0 ± 0 | 0 ± 0 | 3 ± 1 |
| Rhizophagus sp. AT-2018 | 0 ± 0 | 0 ± 0 | 0 ± 0 | 2 ± 1.53 | 0 ± 0 | 0 ± 0 | 0.67 ± 0.33 | 0 ± 0 | 0 ± 0 | 0 ± 0 |
| Rhizophagus sp. MUCL 46100 | 0.33 ± 0.33 | 1.33 ± 1.33 | 0 ± 0 | 0 ± 0 | 0 ± 0 | 0 ± 0 | 0 ± 0 | 0 ± 0 | 0 ± 0 | 0 ± 0 |
| Rhizophagus sp. TC-2016a | 0.33 ± 0.33 | 120.33 ± 43.88 | 0 ± 0 | 2.33 ± 1.86 | 0.33 ± 0.33 | 0.33 ± 0.33 | 17 ± 8.5 | 0 ± 0 | 0 ± 0 | 0 ± 0 |
| Rhizophlyctis rosea | 4.33 ± 4.33 | 0 ± 0 | 1.33 ± 0.88 | 0.33 ± 0.33 | 0.33 ± 0.33 | 0.33 ± 0.33 | 0 ± 0 | 0 ± 0 | 29 ± 29 | 5.33 ± 3.18 |
| Rhizophydium globosum | 0 ± 0 | 0 ± 0 | 0 ± 0 | 0 ± 0 | 0 ± 0 | 0 ± 0 | 0 ± 0 | 1.33 ± 0.88 | 0.33 ± 0.33 | 0 ± 0 |
| Rhizophydium sp. JEL-385 | 0 ± 0 | 0 ± 0 | 0 ± 0 | 0 ± 0 | 0 ± 0 | 0 ± 0 | 0 ± 0 | 0.67 ± 0.67 | 1.33 ± 1.33 | 0 ± 0 |
| Rhizopycnis sp. | 0.67 ± 0.67 | 0 ± 0 | 0.33 ± 0.33 | 0 ± 0 | 0 ± 0 | 6 ± 3.79 | 0 ± 0 | 0 ± 0 | 0 ± 0 | 0 ± 0 |
| Rhizoscyphus sp. | 0 ± 0 | 1.33 ± 0.88 | 0 ± 0 | 5.33 ± 5.33 | 0 ± 0 | 0 ± 0 | 1.67 ± 1.67 | 0 ± 0 | 0 ± 0 | 0 ± 0 |
| Rhodosporidiobolus azoricus | 0 ± 0 | 2.33 ± 0.33 | 0 ± 0 | 0 ± 0 | 0 ± 0 | 0 ± 0 | 0 ± 0 | 1 ± 0.58 | 0.33 ± 0.33 | 0 ± 0 |
| Rhodosporidiobolus odoratus | 9 ± 9 | 0 ± 0 | 8 ± 6.11 | 0 ± 0 | 6 ± 0 | 1.67 ± 1.2 | 1.33 ± 1.33 | 0.67 ± 0.67 | 0.67 ± 0.33 | 6 ± 1 |
| Rhodotorula paludigena | 0 ± 0 | 0 ± 0 | 0 ± 0 | 0 ± 0 | 0 ± 0 | 0 ± 0 | 0 ± 0 | 0 ± 0 | 0 ± 0 | 1.67 ± 1.67 |
| Rhodotorula sp. AY214 | 0 ± 0 | 0 ± 0 | 0.33 ± 0.33 | 0 ± 0 | 0 ± 0 | 1.67 ± 1.67 | 0.67 ± 0.67 | 0 ± 0 | 0 ± 0 | 0 ± 0 |
| Rhodotorula sp. P2S-PDA | 0 ± 0 | 0 ± 0 | 2.33 ± 1.86 | 0.33 ± 0.33 | 0 ± 0 | 1.67 ± 0.88 | 4.67 ± 4.18 | 4 ± 2.31 | 0 ± 0 | 11.67 ± 6.39 |
| Rhodotorula sp. wF6 | 0 ± 0 | 0 ± 0 | 0 ± 0 | 0 ± 0 | 0 ± 0 | 0 ± 0 | 0 ± 0 | 0 ± 0 | 0.67 ± 0.67 | 0 ± 0 |
| Rigidoporus concrescens | 0 ± 0 | 0 ± 0 | 0 ± 0 | 0 ± 0 | 0.67 ± 0.67 | 0 ± 0 | 0 ± 0 | 24.67 ± 24.67 | 1.67 ± 1.67 | 0 ± 0 |
| Rigidoporus crocatus | 0.33 ± 0.33 | 0 ± 0 | 0 ± 0 | 0 ± 0 | 3.67 ± 2.33 | 0 ± 0 | 0 ± 0 | 0 ± 0 | 0 ± 0 | 0 ± 0 |
| Robillarda sessilis | 0 ± 0 | 8 ± 3.79 | 0 ± 0 | 0 ± 0 | 0 ± 0 | 0 ± 0 | 0.67 ± 0.67 | 0 ± 0 | 0 ± 0 | 1 ± 1 |
| Roesleria subterranea | 0 ± 0 | 0 ± 0 | 0 ± 0 | 0 ± 0 | 0 ± 0 | 0 ± 0 | 0.33 ± 0.33 | 0 ± 0 | 0 ± 0 | 0 ± 0 |
| Rosasphaeria moravica | 0 ± 0 | 0 ± 0 | 0 ± 0 | 0 ± 0 | 0 ± 0 | 0 ± 0 | 0 ± 0 | 0 ± 0 | 0 ± 0 | 0 ± 0 |
| Rosellinia aquila | 0 ± 0 | 0 ± 0 | 0 ± 0 | 0 ± 0 | 0 ± 0 | 0.33 ± 0.33 | 0 ± 0 | 0 ± 0 | 1.67 ± 1.67 | 0 ± 0 |
| Roseodiscus subcarneus | 0 ± 0 | 0 ± 0 | 0 ± 0 | 0 ± 0 | 0 ± 0 | 0 ± 0 | 0 ± 0 | 32.33 ± 23.05 | 11.33 ± 5.84 | 0 ± 0 |
| Rotiferophthora minutispora | 0 ± 0 | 0 ± 0 | 0 ± 0 | 0 ± 0 | 0 ± 0 | 0 ± 0 | 1.33 ± 1.33 | 0 ± 0 | 0 ± 0 | 0 ± 0 |
| Roussoella neopustulans | 0 ± 0 | 0 ± 0 | 0 ± 0 | 0 ± 0 | 1 ± 0.58 | 5.33 ± 3.93 | 0 ± 0 | 0 ± 0 | 0 ± 0 | 0 ± 0 |
| Roussoella solani | 0 ± 0 | 0 ± 0 | 0 ± 0 | 0 ± 0 | 0 ± 0 | 0 ± 0 | 3.67 ± 3.67 | 0 ± 0 | 0 ± 0 | 2.67 ± 0.67 |
| Rugosomyces cyanellus | 3.33 ± 1.45 | 0 ± 0 | 0 ± 0 | 0 ± 0 | 1.67 ± 0.88 | 2.67 ± 2.19 | 3 ± 1.73 | 0 ± 0 | 0 ± 0 | 0.33 ± 0.33 |
| Russula catillus | 0 ± 0 | 0 ± 0 | 1.33 ± 1.33 | 0 ± 0 | 0 ± 0 | 0 ± 0 | 6 ± 6 | 881.33 ± 865.84 | 2663.33 ± 1412.66 | 9.33 ± 5.21 |
| Russula cerolens | 0 ± 0 | 0 ± 0 | 2.33 ± 0.88 | 0 ± 0 | 0 ± 0 | 0 ± 0 | 12.67 ± 12.67 | 2000.67 ± 1966.67 | 5141 ± 2710.41 | 14 ± 7.21 |
| Russula livescens | 0 ± 0 | 0 ± 0 | 0 ± 0 | 0.33 ± 0.33 | 0 ± 0 | 0 ± 0 | 1 ± 1 | 0 ± 0 | 0 ± 0 | 0 ± 0 |
| Russula sp. SB125 | 0 ± 0 | 0 ± 0 | 0 ± 0 | 0.67 ± 0.67 | 0 ± 0 | 0 ± 0 | 0 ± 0 | 0 ± 0 | 0 ± 0 | 0 ± 0 |
| Russula sp. tjv4 | 0 ± 0 | 0 ± 0 | 0 ± 0 | 0 ± 0 | 0 ± 0 | 0 ± 0 | 4.33 ± 4.33 | 366 ± 360.51 | 1399.67 ± 782.89 | 4 ± 2.31 |
| Saccharata daviesiae | 0 ± 0 | 0 ± 0 | 0 ± 0 | 0.33 ± 0.33 | 0.67 ± 0.67 | 0 ± 0 | 0 ± 0 | 0 ± 0 | 0 ± 0 | 0 ± 0 |
| Sagenomella oligospora | 0 ± 0 | 2.33 ± 2.33 | 0 ± 0 | 0 ± 0 | 0 ± 0 | 0 ± 0 | 0 ± 0 | 0.33 ± 0.33 | 0 ± 0 | 0.33 ± 0.33 |
| Sagenomella striatispora | 0 ± 0 | 3.33 ± 3.33 | 0 ± 0 | 1 ± 1 | 0 ± 0 | 2.33 ± 1.45 | 6 ± 3.06 | 0 ± 0 | 0 ± 0 | 3 ± 3 |
| Saitozyma ninhbinhensis | 0 ± 0 | 0 ± 0 | 0 ± 0 | 0 ± 0 | 0 ± 0 | 0 ± 0 | 0 ± 0 | 0 ± 0 | 1.33 ± 1.33 | 0 ± 0 |
| Saitozyma paraflava | 0 ± 0 | 3.67 ± 3.67 | 0 ± 0 | 0 ± 0 | 0 ± 0 | 2.33 ± 2.33 | 1 ± 0.58 | 1.33 ± 1.33 | 0 ± 0 | 0 ± 0 |
| Saitozyma podzolica | 9.67 ± 4.37 | 2382.33 ± 1283.45 | 246.33 ± 80.48 | 1156.67 ± 78.75 | 13.33 ± 2.91 | 160.67 ± 56.43 | 770 ± 281.79 | 273.67 ± 85.71 | 333 ± 88.05 | 5976.33 ± 1482.18 |
| Sakaguchia lamellibrachiae | 0 ± 0 | 0 ± 0 | 0.33 ± 0.33 | 0 ± 0 | 0 ± 0 | 0 ± 0 | 0 ± 0 | 0 ± 0 | 0 ± 0 | 0 ± 0 |
| Sarcinomyces sp. MA 4787 | 0 ± 0 | 0 ± 0 | 7.33 ± 3.84 | 0 ± 0 | 0 ± 0 | 0.33 ± 0.33 | 1.33 ± 0.67 | 0 ± 0 | 0 ± 0 | 0 ± 0 |
| Sarcodon glaucopus | 0 ± 0 | 0 ± 0 | 0 ± 0 | 0 ± 0 | 0.33 ± 0.33 | 0 ± 0 | 0 ± 0 | 0 ± 0 | 0 ± 0 | 0 ± 0 |
| Sarcopodium circinatum | 0 ± 0 | 0 ± 0 | 6.67 ± 0.88 | 0.33 ± 0.33 | 0.33 ± 0.33 | 6.33 ± 6.33 | 0 ± 0 | 0 ± 0 | 0 ± 0 | 0 ± 0 |
| Sarea difformis | 0.67 ± 0.33 | 0 ± 0 | 7 ± 3.46 | 0 ± 0 | 0 ± 0 | 4.67 ± 4.67 | 1 ± 0.58 | 0 ± 0 | 0 ± 0 | 0 ± 0 |
| Sarea resinae | 0 ± 0 | 4 ± 2.08 | 0 ± 0 | 0.33 ± 0.33 | 0 ± 0 | 0 ± 0 | 0.67 ± 0.67 | 0 ± 0 | 0 ± 0 | 0 ± 0 |
| Sarea sp. C65 | 0 ± 0 | 0 ± 0 | 0 ± 0 | 0.33 ± 0.33 | 0 ± 0 | 0 ± 0 | 0.67 ± 0.33 | 64 ± 59.53 | 6.33 ± 5.36 | 1 ± 1 |
| Sarea sp. K25 | 0 ± 0 | 0 ± 0 | 0 ± 0 | 0.33 ± 0.33 | 0 ± 0 | 0 ± 0 | 1.33 ± 0.88 | 0 ± 0 | 0 ± 0 | 0 ± 0 |
| Sarocladium bacillisporum | 0 ± 0 | 0 ± 0 | 0 ± 0 | 0 ± 0 | 0.33 ± 0.33 | 0 ± 0 | 0 ± 0 | 0 ± 0 | 0 ± 0 | 0 ± 0 |
| Sarocladium hominis | 0 ± 0 | 0 ± 0 | 3 ± 2.52 | 0 ± 0 | 0.67 ± 0.67 | 0.67 ± 0.67 | 0 ± 0 | 0 ± 0 | 0 ± 0 | 0 ± 0 |
| Sarocladium mycophilum | 0.33 ± 0.33 | 34.67 ± 9.91 | 76.33 ± 17.64 | 12.33 ± 5.81 | 0 ± 0 | 13.33 ± 9.94 | 15 ± 8.08 | 19.67 ± 15.76 | 11 ± 5.57 | 7.67 ± 1.86 |
| Sarocladium strictum | 1 ± 0.58 | 4.33 ± 4.33 | 0.33 ± 0.33 | 0.67 ± 0.33 | 5.33 ± 3.84 | 1.33 ± 0.88 | 1 ± 0.58 | 8.33 ± 7.84 | 0 ± 0 | 4.33 ± 3.38 |
| Sarocladium summerbellii | 0 ± 0 | 0.33 ± 0.33 | 0.33 ± 0.33 | 0 ± 0 | 2 ± 0 | 0.33 ± 0.33 | 0 ± 0 | 0 ± 0 | 0 ± 0 | 0.67 ± 0.33 |
| Sarocladium zeae | 0 ± 0 | 0 ± 0 | 0 ± 0 | 1.67 ± 1.2 | 0 ± 0 | 1 ± 1 | 0.33 ± 0.33 | 0 ± 0 | 0 ± 0 | 0 ± 0 |
| Scedosporium apiospermum | 0 ± 0 | 0 ± 0 | 0 ± 0 | 0 ± 0 | 0 ± 0 | 2.33 ± 1.86 | 0 ± 0 | 0 ± 0 | 0 ± 0 | 0 ± 0 |
| Scedosporium aurantiacum | 0 ± 0 | 0 ± 0 | 0 ± 0 | 0 ± 0 | 0 ± 0 | 2 ± 1.15 | 0 ± 0 | 0 ± 0 | 0 ± 0 | 0.33 ± 0.33 |
| Scedosporium boydii | 0 ± 0 | 0 ± 0 | 0 ± 0 | 0 ± 0 | 0 ± 0 | 0.33 ± 0.33 | 0 ± 0 | 1.33 ± 0.88 | 0 ± 0 | 1.33 ± 1.33 |
| Schaereria fuscocinerea | 0 ± 0 | 0 ± 0 | 0 ± 0 | 0 ± 0 | 0 ± 0 | 0 ± 0 | 0 ± 0 | 0.67 ± 0.67 | 1.67 ± 1.67 | 0 ± 0 |
| Schizophyllum commune | 2 ± 2 | 0.33 ± 0.33 | 0 ± 0 | 0 ± 0 | 0.33 ± 0.33 | 0 ± 0 | 0 ± 0 | 0 ± 0 | 0 ± 0 | 0.33 ± 0.33 |
| Schizothecium carpinicola | 0 ± 0 | 0 ± 0 | 0 ± 0 | 0 ± 0 | 0 ± 0 | 0 ± 0 | 0 ± 0 | 1.67 ± 1.67 | 0 ± 0 | 0.33 ± 0.33 |
| Schizothecium curvisporum | 0 ± 0 | 0 ± 0 | 0 ± 0 | 0 ± 0 | 0 ± 0 | 0.33 ± 0.33 | 0 ± 0 | 0 ± 0 | 0 ± 0 | 0 ± 0 |
| Schizothecium glutinans | 0 ± 0 | 0 ± 0 | 1.33 ± 1.33 | 0 ± 0 | 0 ± 0 | 0.67 ± 0.33 | 0 ± 0 | 8.67 ± 8.67 | 3 ± 3 | 2.67 ± 2.19 |
| Schizothecium inaequale | 0 ± 0 | 0 ± 0 | 0 ± 0 | 0 ± 0 | 0 ± 0 | 0 ± 0 | 0.33 ± 0.33 | 0 ± 0 | 0 ± 0 | 0 ± 0 |
| Schizothecium sp. F277858 | 0.67 ± 0.67 | 488 ± 303 | 0.67 ± 0.67 | 10.33 ± 2.85 | 0.33 ± 0.33 | 3.67 ± 2.33 | 77 ± 59.91 | 0 ± 0 | 0 ± 0 | 0 ± 0 |
| Schizoxylon albescens | 0 ± 0 | 2 ± 1.15 | 0 ± 0 | 0 ± 0 | 0 ± 0 | 0 ± 0 | 0 ± 0 | 0 ± 0 | 0 ± 0 | 0 ± 0 |
| Scleroderma sp. EMF38 | 0 ± 0 | 0 ± 0 | 0 ± 0 | 0 ± 0 | 0 ± 0 | 4 ± 2.65 | 4.67 ± 4.67 | 84.67 ± 84.67 | 247 ± 141.46 | 0.33 ± 0.33 |
| Sclerogaster sp. | 0 ± 0 | 0 ± 0 | 0 ± 0 | 0 ± 0 | 0.67 ± 0.67 | 0 ± 0 | 0 ± 0 | 0 ± 0 | 0 ± 0 | 0 ± 0 |
| Scleropezicula alnicola | 2.67 ± 2.67 | 0 ± 0 | 0 ± 0 | 0 ± 0 | 0.33 ± 0.33 | 1 ± 1 | 0.33 ± 0.33 | 0 ± 0 | 0.33 ± 0.33 | 0 ± 0 |
| Scleroramularia shaanxiensis | 0 ± 0 | 0 ± 0 | 0 ± 0 | 1.33 ± 0.67 | 0 ± 0 | 0 ± 0 | 0.33 ± 0.33 | 0 ± 0 | 0 ± 0 | 0 ± 0 |
| Sclerotinia spermophila | 0 ± 0 | 0 ± 0 | 1.67 ± 1.67 | 0 ± 0 | 0 ± 0 | 0.33 ± 0.33 | 0 ± 0 | 0 ± 0 | 0 ± 0 | 0 ± 0 |
| Scolecobasidium sp. HF12231 | 0 ± 0 | 0 ± 0 | 0 ± 0 | 0 ± 0 | 0 ± 0 | 0 ± 0 | 0 ± 0 | 0 ± 0 | 0 ± 0 | 1 ± 1 |
| Scopulariopsis brevicaulis | 0 ± 0 | 0 ± 0 | 0 ± 0 | 0 ± 0 | 0 ± 0 | 0.67 ± 0.67 | 0.67 ± 0.67 | 0.33 ± 0.33 | 0 ± 0 | 0 ± 0 |
| Scopulariopsis parva | 0 ± 0 | 0 ± 0 | 0 ± 0 | 0 ± 0 | 0 ± 0 | 0 ± 0 | 0 ± 0 | 0 ± 0 | 1.67 ± 1.67 | 0 ± 0 |
| Scytalidium album | 0 ± 0 | 0 ± 0 | 1.33 ± 1.33 | 0 ± 0 | 1 ± 1 | 14.67 ± 6.89 | 1 ± 0.58 | 0 ± 0 | 0.33 ± 0.33 | 10.33 ± 4.33 |
| Scytalidium cuboideum | 0 ± 0 | 2 ± 1.15 | 12 ± 12 | 0 ± 0 | 0 ± 0 | 11.67 ± 11.67 | 0 ± 0 | 0 ± 0 | 0 ± 0 | 1.33 ± 1.33 |
| Scytalidium lignicola | 0 ± 0 | 0 ± 0 | 0 ± 0 | 0.33 ± 0.33 | 1 ± 1 | 1.67 ± 0.88 | 0 ± 0 | 0 ± 0 | 0 ± 0 | 0 ± 0 |
| Scytalidium sp. | 0 ± 0 | 0 ± 0 | 3.67 ± 0.88 | 0.33 ± 0.33 | 0 ± 0 | 4 ± 2.08 | 0.33 ± 0.33 | 0 ± 0 | 0 ± 0 | 1 ± 1 |
| Sebacina epigaea | 0 ± 0 | 0 ± 0 | 1 ± 1 | 0 ± 0 | 0 ± 0 | 0 ± 0 | 0 ± 0 | 0 ± 0 | 0 ± 0 | 0 ± 0 |
| Sebacina incrustans | 0.33 ± 0.33 | 0 ± 0 | 0 ± 0 | 0 ± 0 | 0.33 ± 0.33 | 0 ± 0 | 1.67 ± 1.2 | 0 ± 0 | 0 ± 0 | 0 ± 0 |
| Sebacina sp. | 0 ± 0 | 0.67 ± 0.67 | 24.33 ± 9.13 | 0.33 ± 0.33 | 0 ± 0 | 6 ± 4.16 | 0.67 ± 0.33 | 11.33 ± 5.04 | 9.33 ± 8.84 | 0.67 ± 0.33 |
| Sebacina sp. AA3.8 | 0 ± 0 | 0.33 ± 0.33 | 0 ± 0 | 0 ± 0 | 0 ± 0 | 0 ± 0 | 0.33 ± 0.33 | 0 ± 0 | 0 ± 0 | 0 ± 0 |
| Sebacina sp. EMF41 | 0 ± 0 | 0 ± 0 | 1.33 ± 1.33 | 0 ± 0 | 0 ± 0 | 0 ± 0 | 0 ± 0 | 0 ± 0 | 0 ± 0 | 0 ± 0 |
| Sebacina sp. Rr35 | 0 ± 0 | 0 ± 0 | 0 ± 0 | 0 ± 0 | 0 ± 0 | 0 ± 0 | 0 ± 0 | 18.33 ± 9.7 | 6.33 ± 6.33 | 0.33 ± 0.33 |
| Sebacina sp. Seb12I | 0 ± 0 | 0 ± 0 | 5.33 ± 4.84 | 0 ± 0 | 0 ± 0 | 0.67 ± 0.67 | 0 ± 0 | 0.33 ± 0.33 | 0 ± 0 | 0 ± 0 |
| Sebacina sp. UC2022810 | 0 ± 0 | 0 ± 0 | 0 ± 0 | 0 ± 0 | 0 ± 0 | 0 ± 0 | 0 ± 0 | 0 ± 0 | 0 ± 0 | 1.33 ± 1.33 |
| Seimatosporium dilophosporum | 0 ± 0 | 0.33 ± 0.33 | 0 ± 0 | 0 ± 0 | 0 ± 0 | 0 ± 0 | 0 ± 0 | 0 ± 0 | 0 ± 0 | 0 ± 0 |
| Seimatosporium pistaciae | 0 ± 0 | 0 ± 0 | 0 ± 0 | 0 ± 0 | 0 ± 0 | 0 ± 0 | 0.67 ± 0.67 | 21.33 ± 11.55 | 0.33 ± 0.33 | 0 ± 0 |
| Seiridium ceratosporum | 0 ± 0 | 0 ± 0 | 0 ± 0 | 0 ± 0 | 0 ± 0 | 0 ± 0 | 0 ± 0 | 0 ± 0 | 0 ± 0 | 0 ± 0 |
| Seiridium podocarpi | 0 ± 0 | 0 ± 0 | 0 ± 0 | 0 ± 0 | 0 ± 0 | 0 ± 0 | 0 ± 0 | 0 ± 0 | 0.33 ± 0.33 | 0 ± 0 |
| Seiridium sp. | 0 ± 0 | 0 ± 0 | 0 ± 0 | 0 ± 0 | 0 ± 0 | 0 ± 0 | 1.33 ± 0.88 | 0 ± 0 | 0 ± 0 | 0 ± 0 |
| Selenodriella cubensis | 1.33 ± 0.88 | 22.67 ± 14.85 | 0 ± 0 | 1 ± 1 | 0.33 ± 0.33 | 0 ± 0 | 5.33 ± 2.6 | 0 ± 0 | 0 ± 0 | 1.33 ± 1.33 |
| Sepedonium ampullosporum | 0 ± 0 | 0 ± 0 | 0 ± 0 | 1 ± 1 | 0 ± 0 | 0 ± 0 | 0 ± 0 | 0 ± 0 | 0 ± 0 | 0 ± 0 |
| Sepedonium chalcipori | 0 ± 0 | 0 ± 0 | 8 ± 7.51 | 0 ± 0 | 0 ± 0 | 1 ± 1 | 0 ± 0 | 0 ± 0 | 0 ± 0 | 0 ± 0 |
| Septobasidium sinuosum | 0 ± 0 | 0 ± 0 | 2.67 ± 2.67 | 0 ± 0 | 0 ± 0 | 0.33 ± 0.33 | 0 ± 0 | 0 ± 0 | 0.33 ± 0.33 | 3.67 ± 2.03 |
| Septofusidium herbarum | 0 ± 0 | 0 ± 0 | 0.33 ± 0.33 | 0 ± 0 | 0 ± 0 | 5.67 ± 2.96 | 0 ± 0 | 0 ± 0 | 0 ± 0 | 0 ± 0 |
| Septoglomus constrictum | 0.33 ± 0.33 | 0 ± 0 | 0.33 ± 0.33 | 1 ± 0.58 | 0.67 ± 0.67 | 0 ± 0 | 0 ± 0 | 4 ± 2.08 | 4 ± 1.53 | 19 ± 8.74 |
| Septoglomus furcatum | 0 ± 0 | 0 ± 0 | 0 ± 0 | 0 ± 0 | 1 ± 1 | 0 ± 0 | 0 ± 0 | 0 ± 0 | 0 ± 0 | 0 ± 0 |
| Septoglomus sp. ZS-2014 | 0 ± 0 | 0 ± 0 | 0 ± 0 | 1 ± 1 | 0 ± 0 | 0 ± 0 | 0 ± 0 | 1.67 ± 0.33 | 0.33 ± 0.33 | 48.33 ± 23.69 |
| Septoglomus viscosum | 2 ± 0 | 0 ± 0 | 0 ± 0 | 0 ± 0 | 5.67 ± 4.18 | 0 ± 0 | 0.33 ± 0.33 | 0.33 ± 0.33 | 0.67 ± 0.67 | 0 ± 0 |
| Septoria lepidiicola | 5.33 ± 2.85 | 0.67 ± 0.67 | 93.33 ± 6.84 | 11.33 ± 2.96 | 17.67 ± 3.38 | 85.67 ± 44.73 | 101.33 ± 22.98 | 147.67 ± 114.36 | 12.67 ± 11.2 | 52.67 ± 19.1 |
| Septoriella phragmitis | 1.67 ± 0.33 | 0 ± 0 | 3.33 ± 0.33 | 0 ± 0 | 0 ± 0 | 4.67 ± 3.71 | 0.33 ± 0.33 | 0.67 ± 0.67 | 0 ± 0 | 6.33 ± 1.86 |
| Serendipita herbamans | 0 ± 0 | 0 ± 0 | 0 ± 0 | 0 ± 0 | 0 ± 0 | 0 ± 0 | 0 ± 0 | 6.33 ± 6.33 | 2.33 ± 1.2 | 0.33 ± 0.33 |
| Serendipita sp. MAFF 305840 | 0 ± 0 | 0 ± 0 | 0 ± 0 | 2 ± 0.58 | 0 ± 0 | 0 ± 0 | 1.33 ± 0.67 | 0 ± 0 | 0 ± 0 | 0 ± 0 |
| Serendipita sp. MAFF 305842 | 1.33 ± 0.88 | 0 ± 0 | 0 ± 0 | 0 ± 0 | 5.67 ± 3.48 | 0.67 ± 0.67 | 0 ± 0 | 3.67 ± 2.33 | 1 ± 1 | 0 ± 0 |
| Serendipita vermifera | 0 ± 0 | 4.33 ± 2.96 | 4.67 ± 4.67 | 0 ± 0 | 0 ± 0 | 1.33 ± 1.33 | 0 ± 0 | 13.67 ± 6.84 | 3.67 ± 1.76 | 355.33 ± 167.48 |
| Setophaeosphaeria badalingensis | 0 ± 0 | 0 ± 0 | 0 ± 0 | 0 ± 0 | 0.33 ± 0.33 | 0.67 ± 0.67 | 0 ± 0 | 0 ± 0 | 3.67 ± 3.67 | 0 ± 0 |
| Setophaeosphaeria citricola | 0 ± 0 | 0 ± 0 | 0.33 ± 0.33 | 0 ± 0 | 0.33 ± 0.33 | 0 ± 0 | 6.67 ± 6.17 | 0 ± 0 | 0.67 ± 0.67 | 4.33 ± 3.84 |
| Setophaeosphaeria hemerocallidis | 0.67 ± 0.33 | 0.33 ± 0.33 | 4.33 ± 3.38 | 0 ± 0 | 2 ± 2 | 3 ± 1.53 | 1 ± 1 | 4.33 ± 2.85 | 2.67 ± 1.76 | 7.67 ± 2.33 |
| Setophoma chromolaenae | 0 ± 0 | 0 ± 0 | 0 ± 0 | 0 ± 0 | 0 ± 0 | 0 ± 0 | 0 ± 0 | 0 ± 0 | 0 ± 0 | 2.33 ± 1.45 |
| Setophoma vernoniae | 0 ± 0 | 0 ± 0 | 0 ± 0 | 1 ± 1 | 0 ± 0 | 0.67 ± 0.67 | 1 ± 1 | 0 ± 0 | 0 ± 0 | 0.67 ± 0.67 |
| Shiraia sp. SUPER-H168 | 0 ± 0 | 0 ± 0 | 0 ± 0 | 0 ± 0 | 0 ± 0 | 0 ± 0 | 0 ± 0 | 0 ± 0 | 0 ± 0 | 4.67 ± 4.67 |
[truncated: 41,613 more chars]
